# Plasma MicroRNAs as noninvasive diagnostic biomarkers in patients with Brugada syndrome

**DOI:** 10.1371/journal.pone.0261390

**Published:** 2022-05-26

**Authors:** Yoshihiro Ikeuchi, Hidenori Ochi, Chikaaki Motoda, Takehito Tokuyama, Yousaku Okubo, Sho Okamura, Syunsuke Miyauchi, Shogo Miyamoto, Yukimi Uotani, Yuko Onohara, Mika Nakashima, Rie Akiyama, Hidetoshi Tahara, Kazuaki Chayama, Yasuki Kihara, Yukiko Nakano

**Affiliations:** 1 Department of Cardiovascular Medicine, Hiroshima University Graduate School of Biomedical and Health Sciences, Hiroshima, Japan; 2 Department of Health Management, Hiroshima Red Cross Hospital & Atomic-bomb Survivors Hospital, Hiroshima, Japan; 3 Department of Cellular and Molecular Biology, Graduate School of Biomedical Sciences, Hiroshima University, Hiroshima, Japan; 4 Research Center for Hepatology and Gastroenterology, Hiroshima University, Hiroshima, Japan; 5 RIKEN Center for Integrative Medical Sciences, Yokohama, Japan; Ohio State University, UNITED STATES

## Abstract

**Background:**

Brugada syndrome (BrS) can be diagnosed by a type 1 BrS tracing in a 12-lead electrocardiogram (ECG). However, there are daily variations in the ECGs of BrS patients, which presents a challenge when diagnosing BrS. Although many susceptibility genes have been identified, the *SCN5A* gene is reportedly the main causative gene of BrS. However, most patients do not have an evidence of genetic predisposition to develop BrS. In addition, the diagnosis and risk stratification for ventricular fibrillation (VF) in patients with BrS presents some problems. Meanwhile, circulating micro RNAs (miRNAs) have drawn increased attention as potential biomarkers of various diseases. We hypothesize that circulating miRNAs may be potential diagnostic biomarkers for BrS.

**Methods:**

We enrolled 70 Japanese BrS patients and 34 controls for the screening cohort. A total of 2,555 miRNA sequences were detected using the 3D-Gene miRNAs labeling kit and 3D-Gene Human miRNAs Oligo Chip. We compared the expression of the miRNAs between the BrS patients and the controls. We validated whether the miRNA were significantly up- or downregulated in the screening cohort using RT-PCR. We also enrolled 72 Japanese BrS patients and 56 controls to replicate these miRNAs.

**Results:**

Eight miRNAs (hsa-miR-223-3p, hsa-miR-22-3p, hsa-miR-221-3p, hsa-miR-4485-5p, hsa-miR-550a-5p, hsa-miR-423-3p, hsa-miR-23a-3p, and hsa-miR-30d-5p) were downregulated, and one miRNA (hsa-miR-873-3p) was upregulated by more than 3-fold in BrS patients. The multivariate logistic regression analysis determined that hsa-miR-423-3p, hsa-miR-223-3p, and hsa-miR-23a-3p were independently associated with BrS (P < 0.0001). The AUC based on cross validation was 0.871 with a sensitivity and specificity of 83.5% and 81.1%, respectively.

**Conclusions:**

The plasma miRNAs are potential noninvasive biomarkers of BrS, and the constructed logistic model was useful for discriminating BrS.

## Introduction

Brugada syndrome (BrS) is an inherited arrhythmia that predisposes patients to developing ventricular arrhythmias and sudden cardiac death [1, 2]. BrS is characterized by right precordial ST elevation, and it is diagnosed when a type 1 Brugada ECG pattern, either spontaneously or drug induced, is recorded at least once, according to the HRS/EHRA/APHRS expert consensus statement [3]. In Japan, a type 1 Brugada ECG pattern is observed in 12 per 10,000 inhabitants; type 2 and 3 ECGs, which are not diagnostic of BrS, are much more prevalent, appearing in 58 per 10,000 inhabitants [4]. However, the ST morphologies of BrS patients have day-to-day variations. Thus, diagnosing BrS using a 12-lead ECG has remained challenging, with the true prevalence of BrS in the general population being difficult to estimate. Some patients diagnosed of arrhythmogenic right ventricular cardiomyopathy have been reported to exhibit the characteristic right precordial BrS-type ECG pattern [5]. Chevallier et al. investigated the ECG criteria for discriminating between incomplete right bundle branch block and the types 2 and 3 Brugada ECG patterns [6].

Ever since the *SCN5A* gene was reported to be the main causative gene of BrS [7], many other susceptibility genes have been identified [8]. The mutation detection of these genes range from 11% to 28% of BrS patients [9], which means that many patients with BrS do not have an evidence of genetic predisposition to develop BrS. In a recent genome-wide association study, three single-nucleotide polymorphisms of *SCN5A*, *SCN10*, and *HEY2* were reported to be associated with BrS [10]. Currently, genetic testing is not a definitive diagnosis of BrS [11].

BrS patients with a history of ventricular fibrillation (VF) are generally considered to be at high risk for VF recurrences. In contrast, the risk for VF in asymptomatic BrS patients is only 1.1%–1.5%. Nonetheless, the risk-stratification of BrS patients without a history of VF is very important [12–15]. There have been several reports regarding the prediction of VF occurrence in BrS patients using clinical characteristics or ECG parameters [14, 15]. We also reported a novel prediction method combined with clinical and ECG parameters to assess the risk for VF in BrS patients [16]. However, risk stratification of VF in BrS patients remains controversial.

MicroRNAs (miRNAs) are a class of non-coding single-stranded RNA molecules containing 17–25 nucleotides that post-transcriptionally regulate their target genes by degradation or translational repression of the complementary messenger RNAs [17]. The miRNAs modulate several physiological and pathological pathways in human diseases, including diabetes, cardiovascular diseases, and cancer [18]. Circulating miRNAs are enclosed in micro particles that remain stable in plasma [19]. They have drawn increasing attention as potential diagnostic biomarkers of various diseases, and in the cardiovascular field, circulating miRNAs have been reported as biomarkers of coronary artery disease, heart failure, cardiomyopathy, and cardiac remodeling [20]. However, the role of miRNAs as diagnostic biomarkers for BrS has not been established. We hypothesized that circulating miRNAs might act as potential diagnostic biomarkers for BrS that may assist risk stratification of VF in BrS patients.

## Methods

### Participants

Consecutive adult patients and age and gender matched healthy controls were enrolled from July 2010 through May 2015 (screening cohort: BrS, n = 70, 64 males and 6 females, mean age, 43 ± 18 years vs. control, n = 34, 27 males and 7 females, mean age, 41 ± 11 years) and from June 2015 through August 2019 (validation cohort, BrS, n = 72, 69 males and 3 females, mean age, 46 ± 4 years vs. control, n = 56, 49 males and 7 females, mean age, 45 ± 8 years) from Hiroshima University Hospital. All participants were unrelated Japanese individuals. A diagnosis of BrS was made when the 12-lead ECG showed ST-segment elevation with a type-1 morphology of ≥2 mm in ≥1 right precordial lead either spontaneously or after a provocative drug test (intravenous administration of a Class I antiarrhythmic) in the absence of structural heart disease [3] The Institutional Ethics Committee of the Graduate School of Biomedical Science at Hiroshima University approved all human tissue usage procedures. All participants provided written informed consent.

### Sequence analysis of *SCN5A* and genotyping for screening BrS patients

Genomic DNA was extracted from leukocytes using a QIAamp DNA Blood Mini Kit (QIAGEN, Hilden, Germany) according to the standard protocol in all screening BrS patients. Using Go Taq (Promega, Madison, WI, USA), all coding regions of *SCN5A* were amplified by a polymerase chain reaction (PCR) from 2.5 ng of genomic DNA using our original primers. These amplified coding regions were then sequenced using an ABI PRISM 310 Genetic Analyzer (Applied Biosystems, Foster City, CA, USA) to identify mutations and polymorphisms.

### miRNA expression analysis using microarray

The total RNA was extracted from individual plasma samples using the 3D-Gene RNA Extraction Reagent from a liquid sample kit (Toray Industries, Inc., Kanagawa, Japan). A total of 2,555 miRNA sequences were detected using the 3D-Gene miRNA Labeling kit and 3D-Gene Human miRNA Oligo Chip (Toray Industries, Inc, Kanagawa, Japan).

After a quality check, we analyzed 574 miRNAs which had no missing data.

We used NormFinder [21], which is an algorithm for identifying the optimal normalization gene among candidates, and selected miR-149–3p as a normalizer to analyze miRNA because stability value by NormFinder was good (0.076) and stability of qRT-PCR measurements was also good. We compared expression of the 574 mRNAs between BrS and controls. We also compared expression of the 574 mRNAs between symptomatic and asymptomatic BrS. The miRNA data are shown in S2 Table in S1 File.

### Pathway analysis

We performed a pathway analysis of miRNAs using DIANA mirPath (ver. 3), which showed significantly different expressions between the BrS patients and controls.

### RNA isolation and quantitative reverse transcription PCR

For replication of selected miRNAs as a result of the microarray data, we performed quantitative reverse transcription-PCR (qRT-PCR) analysis. The levels of miRNA expression were investigated using TaqMan advanced cDNA synthesis kit (A28007, Thermo Fisher Scientific), TaqMan fast advanced Master Mix (#4444557, Thermo Fisher Scientific), and TaqMan advanced microRNA assay (Thermo Fisher Scientific, San Jose, CA, USA) kit. The expression levels were normalized to that of hsa-miR-149–3p.

### Statistical analysis

The normally distributed continuous variables are presented as mean ± standard deviation. For miRNA array-based analyses, the log_2_ ratio of each plasma miRNA after normalization as calculated by the ratio of the BrS patients to the controls, or BrS patients with VF events to BrS patients without VF events. The unpaired Student’s t-test was used in the microarray data or qRT-PCR data to evaluate the differences between the two groups. In addition, The false discovery rate (FDR) by the Benjamini–Hochberg test was used. We combined the FDR criterion (<0.1) with the fold change (>3) to select a list of differentially expressed miRNAs in the BrS compared with the controls.

The receiver-operating characteristic (ROC) curves and the areas under the ROC curve (AUC) were used to assess the feasibility of using plasma miRNAs as diagnostic tools to detect those with BrS among the controls. The relationship between significant down- or upregulated miRNAs in BrS was analyzed using cluster analysis. Based on the factors deemed significant by the univariate analysis, multivariate analysis was performed using logistic regression with stepwise forward selection in the total BrS patients and control subjects. The odds ratios and 95% confidence intervals were calculated for the control subjects.

The predictive value of the BrS was assessed, and the performance of the logistic model was analyzed by the ROC analysis. We performed cross-validation of the logistic model using the leave-one-out cross-validation.

All statistical analyses were conducted using the R3.3.1 and the JMP statistical packages (version 13, SAS Institute, Cary, NC).

## Results

### Difference of miRNA expression using the microarray between BrS patients and controls

**Fig 1:**
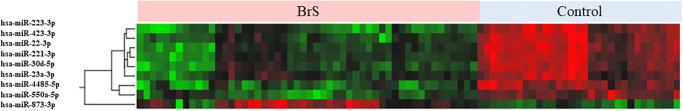
Heat map of hierarchical clustering analysis based on the significantly changed miRNA expression between BrS patients and controls. Some BrS patients showed similar miRNA expression to those of the controls, but there were some notable differences between the BrS patients and the controls. hsa-miR-873–3p and the other miRNAs formed different clusters.

Based on the results of the 3D gene microarray analysis, 8 miRNAs (hsa-miR-223–3p, hsa-miR-22–3p, hsa-miR-221–3p, hsa-miR-4485–5p, hsa-miR-550a-5p, hsa-miR-423–3p, hsa-miR-23a-3p, and hsa-miR-30d-5p) were significantly downregulated while one miRNA (hsa-miR-873–3p) was significantly upregulated by more than 3-fold in BrS patients. (Table 1) The miRNAs hsa-miR-223–3p, hsa-miR-22–3p, hsa-miR-221–3p, hsa-miR-4485–5p, hsa-miR-423–3p, and hsa-miR-23a-3p were closely related and had strong positive correlations with each other. Meanwhile, the miRNAs hsa-miR-550a-5p and hsa-miR-873–3p had moderate positive and weak negative correlations with the other miRNAs, respectively (S2 Table in S1 File). Fig 1 shows a heat map of the hierarchical clustering analysis based on the significantly changed miRNA expressions between BrS patients and controls. Although some of the BrS patients showed similar miRNA expressions to those of the controls, there were some notable differences. hsa-miR-873–3p and the other miRNAs formed different clusters.

**Table 1:** Difference of miRNA expression between BrS patients and controls in the screening and replication cohorts.

	Screening Cohort						Replication Cohort			
	3D-Gene® data			qRT-PCR validation			qRT-PCR			Regulation
miRNA	P-value*	Corrected P-value	Fold change	P-value*	Corrected P-value	q-value#	P-value*	Corrected P-value	q-value#	
hsa-miR-223–3p	6.47x10^-16^	3.71x10^-13^	6.11	1.12x10^-5^	1.11x10^-4^	4.568E-05	0.027	0.162	0.0324	Down
hsa-miR-22–3p	3.67x10^-22^	2.11x10^-19^	5.83	7.59x10^-4^	6.83x10^-3^	0.0013662	0.012	0.072	0.018	Down
hsa-miR-221–3p	3.75x10^-19^	2.15x10^-16^	5.54	2.03x10^-5^	1.83x10^-4^	4.568E-05	4.70x10^-3^	0.028	0.0141	Down
hsa-miR-4485–5p	2.85x10^-20^	1.63x10^-17^	4.35	0.077	0.693	0.099				Down
hsa-miR-550a-5p	2.04x10^-20^	1.17x10^-17^	3.97	0.250	2.250	0.25				Down
hsa-miR-423–3p	2.66x10^-20^	1.53x10^-17^	3.71	9.25x10^-6^	8.32x10^-5^	4.568E-05	7.71x10^-4^	4.62x10^-3^	0.004626	Down
hsa-miR-23a-3p	9.71x10^-13^	5.57x10^-10^	3.63	7.37x10^-3^	0.066	0.011055	0.036	0.216	0.036	Down
hsa-miR-30d-5p	8.67x10^-18^	4.97x10^-15^	3.44	1.89x10^-5^	1.70x10^-5^	4.568E-05	0.012	0.072	0.018	Down
hsa-miR-873–3p	1.18x10^-12^	6.77x10^-9^	3.05	0.137	1.233	0.154125				UP

* unpaired Student’s t-test,

^#^FDR (false discovery rate) by the Benjamini–Hochberg test, Corrected P-value is after Bonferonni correction

### *SCN5A* mutations and miRNAs in BrS patients with and without VF events

*SCN5A* mutations were detected in two BrS patients with VF events (R893C and IVS21+1 G>A) and in four BrS patients without VF events (N782T, G1420R, H278R, and N740del). The frequency of the *SCN5A* mutation was similar in the BrS patients with and without VF events (10% vs. 9.3%, respectively). There were no significantly up or down regulated miRNA among the BrS patients with or without VF events. The hsa-miR-6880–5p in the microarray was more downregulated in BrS patients with VF events than in the BrS patients without VF events and showing the lowest P value, but it was not significant.

### Validation of the significant miRNAs using qRT-PCR in the screening cohort and replication cohort

The miRNAs miR-22–3P, miR-221–3p, miR-23a-3p, miR-30d-5p, miR-423a-3p, and miR-223–3P were significantly more downregulated in BrS patients compared with the controls after qRT-PCR variation (Table 1). In the replication cohort, the miRNAs miR-22–3P, miR-221–3p, miR-23a-3p, miR-30d-5p, miR-423a-3p, and miR-223–3P were also significantly more downregulated in BrS patients compared with the controls. (Table 1).

### ROC curves and the AUC of the significant miRNAs

**Fig 2:**
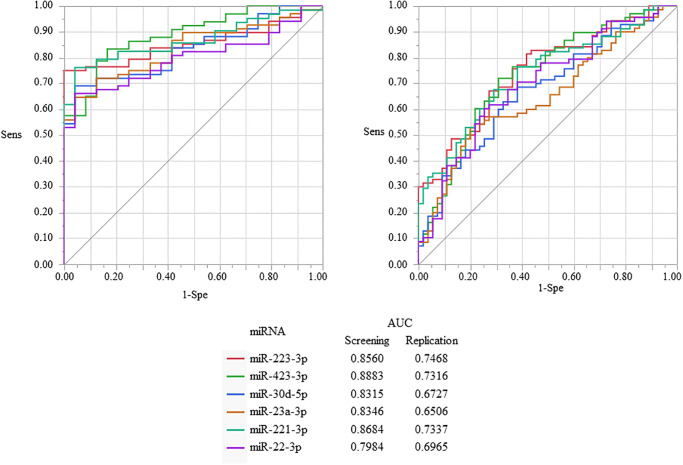
Receiver-operating characteristic (ROC) curves and the area under the ROC curve (AUC) of the significant miRNAs. The AUC of all miRNAs showing significantly different expressions between the BrS patients and the controls in the screening cohort was more than 0.8. The AUC of miR-423–3p was the highest at 0.8883. The AUC of the significant miRNAs in the replication cohort was lower than those in the screening cohort.

The AUC of all miRNAs in the screening cohort were more than 0.8. The AUC of miR-423–3p was the highest at 0.8883. The AUC of the significant miRNAs were lower in the replication cohort than those in the screening cohort. (Fig 2).

### Pathway analysis of the significant miRNAs

The pathway analysis results indicated that hsa-miR-22–3P, hsa-miR-221–3p, hsa-miR-23a-3p, hsa-miR-30d-5p, and hsa-miR-423a-3p were important for the function of the adherens junction pathway (P = 5.06 x 10^-5^), whereas hsa-miR-223–3p was not involved in the adherens junction pathway.

### Construction of the BrS prediction model

**Fig 3:**
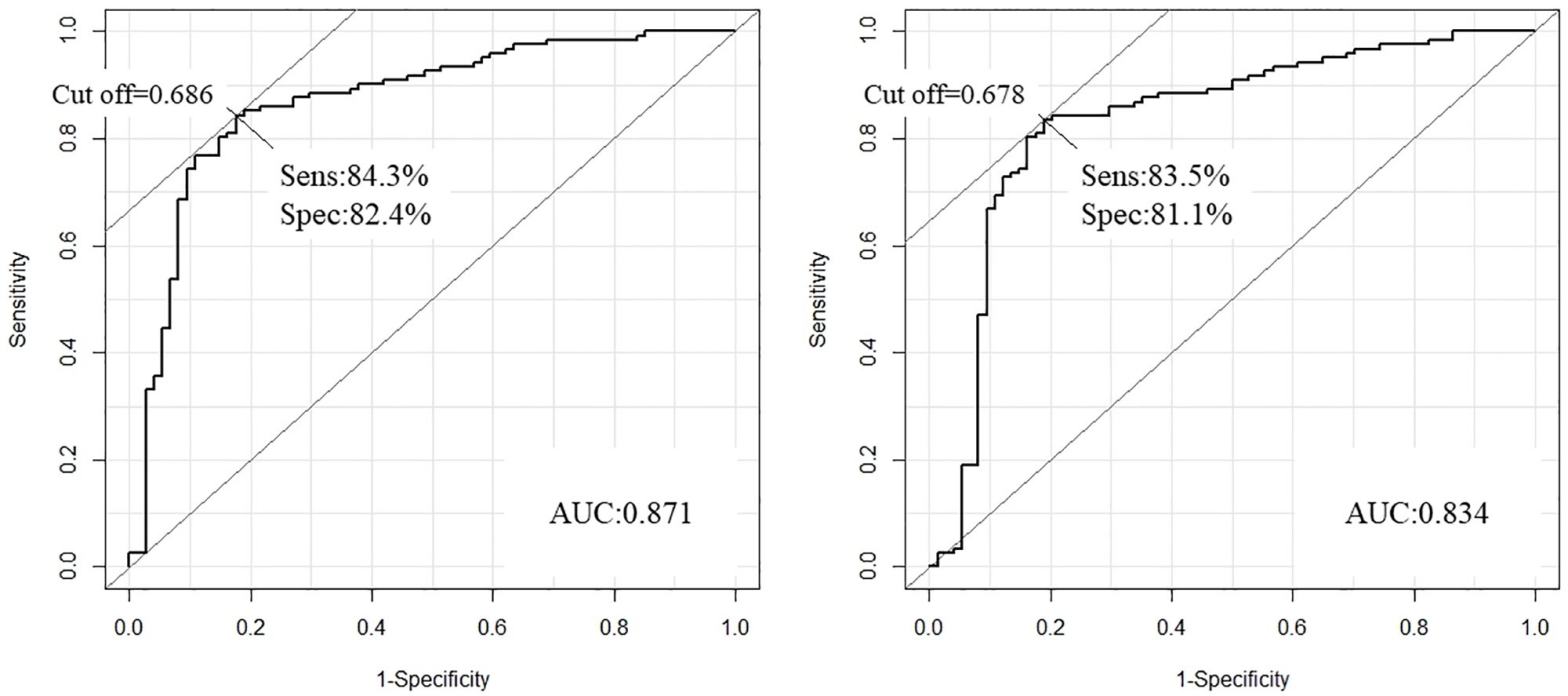
ROC curves of the BrS prediction model using miR-23a-3p, miR-423a-3p, and miR-223–3P. The miR-23a-3p, miR-423a-3p, and miR-223–3P remained as independent predictors for BrS patients. The ROC curve using these three miRNAs showed good discrimination of the BrS patients from the controls with an area under the curve (AUC) of 0.871 and a sensitivity and specificity of 84.3% and 82.4%, respectively (Fig 3, left). Internal validation was performed by the leave-one-out cross-validation technique. The AUC based on cross-validation was 0.834 with a sensitivity and specificity of 83.5% and 81.1%, respectively.

Multivariate analysis was performed for 142 Japanese BrS subjects and the 90 controls with significant miRNAs in the unpaired Student’s t-test, and a P level of 0.05 was deemed significant (Table 2). miR-23a-3p, miR-423a-3p, and miR-223–3P remained as the independent predictors for BrS patients. We performed ROC analysis using these three miRNAs (miR-23a-3p, miR-423a-3p, and miR-223–3P). The ROC curve showed good discrimination of the BrS patients from the control with an AUC of 0.871 and sensitivity and specificity of 84.3% and 82.4%, respectively (Fig 3, left). Internal validation was performed with the leave-one-out cross-validation technique. The AUC based on the cross-validation was 0.834 with a sensitivity and specificity of 83.5% and 81.1%, respectively (Fig 3, right).

**Table 2:** Multivariate analysis of significant miRNAs expression in univariate analysis between BrS patients and controls.

miRNA	BrS	Control	Odds ratio	95% CI	Multivariate P-value
hsa-miR-223–3p	0.75 ± 0.25	3.16 ± 0.53	0.62	0.41–0.93	0.0225
hsa-miR-22–3p	0.51 ± 0.16	2.12 ± 0.22			0.6933
hsa-miR-221–3p	1.89 ± 0.65	6.74 ± 0.88			0.6848
hsa-miR-423–3p	2.46 ± 0.71	9.15 ± 0.96	0.79	0.69–0.94	0.0011
hsa-miR-23a-3p	2.43 ± 0.90	13.4 ± 1.20	0.82	0.71–0.94	0.0005
hsa-miR-30d-5p	1.01 ± 0.34	4.82 ± 0.46			0.1018

## Discussion

In this study, the miRNA microarray showed that 8 circulating miRNAs were upregulated (>3 fold), and one circulating miRNA was downregulated (>3 fold) in BrS patients compared with the controls. After the-PCR validation in the screening and the replication cohorts, we confirmed that 6 miRNAs (miR-22–3P, miR-221–3p, miR-23a-3p, miR-30d-5p, miR-423a-3p, and miR-223–3P) downregulated significantly in BrS patients compared with the controls. Among them, miR-23a-3p, miR-423a-3p, and miR-223–3P were independent predictors and potential biomarkers of BrS. We could establish a logistic model consisting of these miRNAs to discriminate BrS patients from non-BrS controls and obtain a satisfactory AUC in the ROC analysis. Unfortunately, we could not find a significant biomarker of BrS with a high risk for VF.

A previous study has reported that miR-223 was upregulated in the heart after myocardial infarction and was related to arrhythmogenesis [22]. miR-223–3p is complementary to the coding sequence of *KCND2* that encodes KV4.2 and I_to_. Co-transfection of luciferase-Kcnd2 and miR-223–3p suppressed luciferase activity. miR 223–3p also regulates KCND2 and cardiac action potential [22, 23]. Furthermore, a decrease-of-I_Na_ and an elevated I_to_ could induce the ventricular cardiomyocytes in patients with BrS, causing proarrhythmic changes [24, 25]. The downregulation of miR-223–3p may relate to the pathogenesis of BrS.

As a result of the pathway analysis, the other significant miRNAs were related to the adherens junction. There are intimate relationships between the desmosomes, adherens junctions, gap junctions, and sodium channel complexes. Adherens junctions combine with desmosomal proteins to form the area composita. Voltage-gated sodium-channel (*NaV 1.5*) complexes are located in the area composita along with cadherin 2, one of the adherens junction, boundary of gap junction. The *NaV1.5* interact with connexin-43 (Cx43), ankyrin-G, and PKP2 [26, 27]. A mutation in the Na_v_1.5 protein reportedly blocked interaction with *ankyrin-G* binding that disrupts the surface expression of Na_v_1.5 in cardiomyocytes, resulting in BrS [28]. Loss of expression of PKP2 in adult and in neonatal rat ventricular myocytes led to a shift in voltage-dependent inactivation properties of the voltage-gated sodium channel Na_V_1.5 [29]. PKP2-dependent disorders of sodium current have been reported to lead to phenotype-related BrS [30]. The *PKP2* gene mutation was reported to associated with arrhythmogenic right ventricular cardiomyopathy (ARVC) and BrS [31].

There had been no established biomarker for BrS. However, cardiac actin, skeletal actin, keratin, and Cx43 identified from the serum of BrS patients were recently reported as biomarkers of BrS [32]. These close links provide support for the overlap of two diseases based on intercalated disc disorders, ARVC and BrS.

Non-coding RNAs are known to perform post-transcriptional regulation of genes and proteins expression [17]. It was revealed that the production of connexin is also modified by epigenetic actions [33]. Connexin levels were reported to be controlled post-transcriptionally by miR-221–3p and miR-23a-3p [34]. The downregulation of the miRNAs associated with the adherens junction may also relate to the pathogenesis of BrS.

Risk stratification in BrS remains unresolved. A lot of ECG or clinical parameters were reported and combination of some parameters were reported to be useful, but they were insufficient [12–16]. We reported that BrS patients with *SCN5A* mutation showed poor prognosis [35]. However, the prognosis cannot be estimated by that alone, and the genetic test is not so simple. Therefore, we wondered if miRNA could be a biomarker capable of stratifying the risk of BrS, but could not detect miRNA with a significant difference between symptomatic and asymptomatic BrS. It may be due to the small number of symptomatic and asymptomatic BrS, so we would like to increase the number of cases and reexamine in the future.

This study has some limitations. Currently, these miRNAs are only biomarkers. Further investigation using cardiac muscles of BrS patients is needed. In order to minimize the waste of the training dataset and to avoid overfitting, leave-one-out cross-validation was performed after combining both the screening and replication cohort. The prediction performance (AUC) in the cross-validation was a comparably high value of 0.834, demonstrating the lack of serious overfitting, although further external validation is still desirable. We must validate the association between BrS and these miRNAs in a larger sample of cases and controls. Additionally, in future studies, I would like to consider miRNAs as a biomarker for distinguishing between Brugada-type ECG cases and true Brugada syndrome patients.

In conclusion, we identified the potential biomarkers of BrS using circulating miRNAs. Most patients with BrS do not have a genetic predisposition, and we also cannot apply genetics to diagnose for BrS. Hence, these miRNAs may be useful in diagnosing BrS.

## Supporting information

S1 File (PDF)
