# Supplementary material for: Plasma MicroRNAs as noninvasive diagnostic biomarkers in patients with Brugada syndrome
Source: PLoS One. 2022 May 26;17(5):e0261390. doi: 10.1371/journal.pone.0261390 (PMC9135283; doi:10.1371/journal.pone.0261390)
Supplement: S1 File — (PDF) [file pone.0261390.s001.pdf]

Supporting Information

Supplementary Table 1. Array Data of miRNAs in BrS and Controls

ID	hsa-miR-103a-3p	hsa-miR-1181	hsa-miR-1185-1	hsa-miR-1185-2	hsa-miR-1193	hsa-miR-1199-3	hsa-miR-1199-5	hsa-miR-1203	hsa-miR-1207-5	hsa-miR-1224-3	hsa-miR-1224-5	hsa-miR-1225-3	hsa-miR-1225-5
0_P673	-2.499908882	-4.261370021	-3.50466997	-4.049590686	-4.905633245	-5.767828447	-3.30450867	-1.922757317	-2.32632821	-3.671216697	-4.608765648	-3.902501905	-1.253564915
0_P674	-1.732672532	-3.680220809	-3.617821991	-4.514010332	-4.432216594	-5.073902077	-4.053555983	-2.35651581	-2.14060392	-3.989643645	-4.719532986	-3.4058699	-1.90135842
0_P675	-2.146050128	-3.923490118	-3.636884001	-4.45445332	-4.414702717	-5.53361794	-3.866729358	-2.461904845	-2.193894437	-3.782013244	-4.949631914	-3.560606026	-1.567313259
0_P679	-2.675446261	-4.13622796	-3.065883166	-3.783981518	-4.503570205	-5.575408584	-4.097236005	-2.811630512	-2.065511732	-3.698197253	-4.640394408	-3.722651016	-1.573401259
0_P681	-2.712327319	-4.111110769	-3.192992765	-4.016008896	-4.540785016	-5.498005008	-3.314796723	-2.225391356	-2.155809019	-3.251583728	-4.669460137	-3.559743784	-1.421513391
0_P682	-2.347485344	-3.770502747	-3.671218618	-3.947273807	-4.214834461	-5.265299503	-3.786301128	-2.519022213	-2.125106363	-3.62754622	-4.495937823	-3.168118638	-1.979427422
0_P684	-1.230099943	-3.826214284	-3.268895672	-3.958045888	-4.52263487	-5.425617938	-3.706809929	-2.517509112	-2.206249883	-3.556659502	-4.631135612	-3.423604796	-1.724054954
0_P687	-2.51035	-3.800436937	-3.330598163	-3.862856469	-4.238913299	-5.350385742	-4.012555356	-2.597290126	-2.141964718	-3.890951431	-4.820804391	-3.270302897	-2.021982772
0_P688	-2.537516759	-3.932736043	-3.492085688	-3.904884746	-4.403731701	-5.306411944	-3.983222394	-2.592910115	-2.125570348	-3.903162378	-4.780494486	-3.558030062	-1.816247189
0_P692	-3.307765946	-3.801479634	-3.341687772	-3.716967471	-4.064232188	-5.096092977	-4.029529318	-2.616180588	-2.206058247	-3.745415496	-4.53466751	-3.256269654	-2.015010006
0_P698	-1.814164669	-4.145292546	-2.660446958	-3.720592326	-4.399379309	-5.656898401	-4.119593791	-2.901382517	-2.137217423	-4.123327131	-4.901316518	-3.432435413	-1.971933059
0_P699	-4.792920191	-4.069687934	-3.533689738	-4.567978354	-4.77774244	-5.697214066	-3.836652515	-2.186885936	-2.369269827	-4.088998162	-5.225414958	-3.756060389	-1.899045288
0_P700	-5.57190438	-4.206227374	-3.706618952	-4.712769929	-4.348654566	-5.977621871	-4.27315406	-2.850016621	-2.092784273	-4.098901266	-5.139736563	-3.868818786	-2.163422637
0_P701	-4.478703808	-3.997811592	-3.625726014	-4.363099946	-4.441707763	-5.479856034	-3.972939554	-2.605603275	-2.472068521	-3.93273919	-5.313265088	-3.521510814	-1.836301577
0_P702	-2.812504698	-3.708416029	-3.694507858	-4.679491848	-4.476058105	-5.30004644	-3.940929421	-2.018148597	-2.435129743	-4.207417578	-5.132577014	-3.343187915	-2.111788104
0_P706	-3.74592203	-3.74873069	-3.260917457	-4.195089083	-4.326600156	-5.438856785	-4.348349152	-2.400266997	-2.148729824	-4.084383738	-4.851148231	-3.277687667	-1.809559966
0_P708	-2.985004559	-3.594281601	-3.966755352	-4.294227072	-4.381244909	-5.171166974	-3.95146293	-1.935106642	-2.521978795	-4.118344835	-4.899780131	-3.0582496	-2.185632716
0_P710	-1.512597383	-4.027424123	-3.812790552	-4.800347811	-4.271076362	-5.907895031	-4.39059476	-2.695080063	-2.225921549	-4.264230667	-5.325546391	-3.097877627	-2.31658595
0_P711	-3.299690124	-4.193210832	-3.036275948	-3.96609618	-4.556303625	-5.828333179	-4.0600949	-2.531392797	-2.38444124	-3.929007762	-4.937060108	-3.549211326	-2.021588055
0_P712	-1.712301925	-3.850596096	-3.514670531	-3.927734128	-4.338446404	-5.37840037	-4.047491209	-2.580403001	-2.290383698	-4.079317068	-4.87754041	-3.259790412	-2.036777937
0_P714	-1.945135145	-3.60644297	-4.130123513	-4.917897812	-4.48075914	-5.132100725	-3.65225648	-1.965687288	-2.426667193	-4.236703702	-5.032603332	-3.274252358	-1.984472955
0_P715	-3.060888902	-3.642093967	-4.050591543	-4.939130261	-4.399839462	-5.290336206	-3.501549412	-1.727288439	-2.506995511	-4.106664904	-5.144559633	-3.278379158	-1.883060791
0_P723	-2.997636699	-4.098503418	-2.95196556	-4.212532315	-4.243559296	-5.777266811	-3.906797934	-2.590701093	-2.04921491	-4.015593265	-5.077694332	-3.534464252	-1.961318262
0_P725	-4.856410148	-4.07269379	-2.661002363	-3.899171303	-4.566250799	-5.888059303	-3.369546928	-1.947440799	-2.443576021	-3.645228125	-4.971229789	-3.569399649	-1.623522254
0_P727	-4.119498219	-3.691441929	-3.675228338	-4.515167946	-4.310427177	-5.355232496	-3.74161176	-2.229971972	-2.375684867	-3.982441189	-4.92809375	-2.972525552	-1.839653772
0_P728	-4.461184506	-4.167774085	-3.414552953	-3.669351941	-4.671628059	-5.940898365	-2.974909332	-1.732847585	-2.387717322	-3.490833253	-4.963259205	-3.524419768	-1.492083031
0_P729	-4.223436466	-4.301039818	-3.811728768	-4.603445095	-4.498653683	-5.872105091	-4.070505438	-2.977281845	-2.315622279	-3.918144774	-5.01601637	-3.647643997	-2.294597614
0_P730	-3.872860609	-4.434823202	-3.581344425	-4.52650869	-4.955461618	-6.163819654	-3.484875625	-2.440760905	-2.576956657	-3.833464009	-5.167029562	-3.96475935	-1.899293514
0_P772	-3.261055778	-4.385349802	-4.065039912	-4.293895628	-4.987053766	-5.970875847	-3.633362197	-1.692501978	-2.461973381	-3.858430654	-5.335927792	-3.647371431	-1.959841422
0_P804	-4.328351888	-4.132763745	-3.820420244	-4.581252078	-4.337128111	-5.645497029	-4.05457791	-2.661942935	-2.202972707	-3.688520016	-4.92296146	-3.484307514	-1.883884829
0_P811	-2.236596825	-4.158579088	-3.059220516	-3.766511375	-4.393866583	-5.701269463	-4.251296011	-3.086985717	-2.163241723	-4.040301007	-4.719778523	-3.5590726	-2.178670785
0_P1021	-5.411417365	-5.320722693	-4.233644427	-5.196525864	-5.071112009	-6.97218397	-4.644214496	-3.119912928	-2.804543952	-4.521211472	-5.772117714	-4.883601211	-2.826149224
1_P12	-5.259062796	-3.989847895	-3.705554296	-4.614821604	-4.11340925	-5.439197555	-4.428229637	-2.933552692	-2.650987989	-4.380334342	-4.861869934	-2.690054136	-2.405441383
1_P16	-7.192427151	-3.831209456	-3.705385878	-4.41603609	-4.200163292	-5.162605631	-4.253781767	-3.32129144	-2.234806086	-4.199795489	-4.812702548	-2.085906725	-2.533998562
1_P59	-6.253528386	-4.063619434	-4.268865386	-4.999485851	-3.949985342	-5.904069618	-4.477866076	-3.221975895	-2.718344423	-4.041001829	-5.350335685	-3.196559305	-2.020046055
1_P105	-4.18681822	-3.678280163	-4.991933585	-5.133929565	-4.703262907	-5.196264585	-3.352302825	-2.201727883	-2.410252808	-3.780606287	-4.672614016	-2.891775469	-1.663615757
1_P139	-3.418818536	-3.655169426	-4.421037516	-5.071954645	-4.330136906	-6.491768714	-4.532026752	-2.404450751	-2.953543443	-4.371034726	-4.646763633	-1.512567096	-2.42420972
1_P148	-3.808624606	-3.559209264	-4.305122339	-5.444097386	-4.013589399	-5.566102511	-4.158889612	-2.812619842	-2.360938995	-4.249889339	-4.855753302	-2.167576205	-2.41151753
1_P149	-4.526170961	-4.113794749	-4.023861114	-4.910549092	-4.236153547	-5.844302542	-4.600271365	-3.326095234	-2.486273488	-4.018086004	-5.208522063	-2.944955982	-1.935458382
1_P150	-4.660018733	-3.743425923	-4.318534095	-4.7062027	-4.066820409	-5.675383776	-4.019050248	-1.776481946	-2.943185391	-4.056680299	-4.547720255	-2.395314847	-2.650272955
1_P153	-4.733760804	-3.867845673	-4.47274638	-5.396487296	-4.441619449	-5.957264621	-3.254722685	-2.00814838	-2.494510263	-4.009748851	-4.985097102	-3.140321496	-1.794895931
1_P156	-2.927101565	-4.404398552	-4.075931506	-4.946239557	-4.217180017	-5.296633083	-4.786263275	-4.284344952	-2.226925843	-4.383287397	-4.849581914	-2.21831017	-2.445563349
1_P167	-4.571889294	-4.652943212	-4.246010595	-4.805440206	-4.478185502	-5.996306021	-5.266186239	-4.46126466	-2.773143985	-4.813977146	-4.9466421	-2.522749365	-3.067922848
1_P172	-7.264493023	-4.363432824	-2.904662301	-3.572665552	-4.577084296	-6.001338042	-2.673573719	-1.882548177	-2.112587503	-3.304457196	-4.744300663	-3.278818858	-0.574913067
1_P174	-4.812207416	-4.0018928	-4.604632117	-5.017427873	-4.194710147	-5.469381803	-4.805312393	-3.630075896	-2.494971045	-4.374606929	-4.854723268	-1.927111215	-2.449892377
1_P177	-4.316808776	-3.892779088	-4.430956196	-5.378865081	-3.990295288	-5.509634475	-4.309798497	-3.148110096	-2.568013599	-4.300554919	-5.24206578	-2.625495305	-2.059928068
1_P178	-4.761462551	-3.901754351	-4.045037126	-4.66512732	-4.570705598	-5.841388215	-3.732001705	-3.10576317	-2.475631355	-3.867845498	-5.217334644	-3.143802034	-1.261970256

1_P179	-3.719677347	-4.101167374	-3.849040473	-4.419357108	-4.214376952	-5.508089883	-4.944454217	-3.589166288	-2.51385776	-4.372463968	-5.05545355	-2.616440167	-2.374793899
1_P183	-4.247767237	-3.255660984	-4.205085061	-4.822483975	-3.801896201	-4.657638241	-4.137853316	-2.632611168	-2.60395761	-4.650420761	-3.910297461	-1.441946524	-2.126054314
1_P191	-4.122644408	-3.490732687	-4.142398905	-4.769899284	-4.167813938	-5.098319733	-4.008156717	-2.292693746	-2.50648653	-4.092032116	-4.571662843	-2.61996632	-1.914278804
1_P192	-6.55778616	-4.755688418	-2.533956943	-3.595681026	-4.833282689	-6.115291904	-3.273971276	-2.278843725	-2.097910889	-3.430331072	-4.905966214	-3.699205319	-0.866535604
1_P194	-6.841456043	-4.545353447	-3.74087506	-4.149532119	-4.58493258	-6.095844391	-3.129978983	-2.231973411	-2.931082463	-3.864145235	-5.146569645	-3.485763318	-1.905219199
1_P196	-3.652476944	-3.763754319	-4.158530993	-4.604272943	-3.958386338	-4.767137667	-4.636154451	-3.271908552	-2.708887509	-4.452553935	-4.00934164	-1.410973128	-2.722703312
1_P200	-5.399158142	-3.133473484	-3.554469949	-3.343388657	-4.07437062	-4.608885218	-3.839045905	-2.441427309	-2.591950482	-3.426911429	-3.236682629	-1.887350745	-2.0708053
1_P204	-4.005132599	-4.179210674	-3.904867406	-4.490523566	-4.578660564	-5.9417469	-3.88312249	-2.716647202	-2.677174333	-3.937905599	-5.022576565	-2.981588494	-2.240599916
1_P206	-8.549658377	-3.76010508	-3.781848439	-3.818797612	-4.158135941	-5.323900261	-4.162093346	-3.323124358	-2.396115202	-3.976225598	-4.989958903	-1.506084675	-2.323356163
1_P212	-5.62199577	-4.219730713	-4.12235694	-4.474189392	-4.139640452	-5.406014172	-4.622162254	-3.736634314	-2.32875286	-4.295475175	-4.925419633	-2.176872689	-2.408501481
1_P217	-3.573402903	-4.520431994	-4.45277004	-5.93293317	-3.745976307	-5.530588652	-4.312799054	-3.378083727	-2.737481751	-4.500582449	-5.091178143	-2.471086339	-2.836346718
1_P218	-4.530997703	-4.441623469	-4.280354613	-4.892015993	-3.932749903	-5.563522104	-4.208337631	-3.96898495	-2.518871172	-4.240767586	-4.368159492	-2.790785288	-1.858233234
1_P230	-6.276223973	-4.537582557	-4.570784271	-4.992517623	-5.026516782	-6.398825928	-3.141463355	-1.78627998	-2.767167845	-4.294186394	-5.33656536	-3.533228192	-2.342018332
1_P238	-6.526044796	-4.493259977	-4.288835337	-4.639778486	-4.395127755	-6.20405233	-4.375185619	-4.109845426	-2.89960045	-4.773772518	-4.822706267	-2.180064477	-2.357811569
1_P248	-5.486273347	-4.242097257	-3.839984561	-4.240274655	-3.981668379	-5.788693955	-5.3710692	-3.542700842	-2.968954409	-4.594897172	-4.413101598	-2.154497188	-2.691218852
1_P263	-3.035216749	-3.793339491	-4.530650639	-5.109904336	-4.42388137	-5.892751888	-3.972038565	-2.562169457	-2.767856419	-4.326645591	-4.431166193	-2.084188195	-2.239238489
1_P328	-2.374015554	-3.835030307	-4.647892318	-5.184004809	-4.501968664	-5.737554808	-4.325739185	-2.950108403	-2.581933563	-4.083784765	-5.004079906	-2.185190536	-2.173759983
1_P341	-6.09668457	-4.42115497	-3.831098961	-4.423483444	-4.906603865	-6.078255284	-2.951423367	-1.75306498	-2.597413585	-3.421673655	-5.01849615	-3.563336394	-1.742218759
1_P395	-8.517369866	-3.914082169	-3.544587831	-3.751208484	-4.274382676	-5.510007128	-4.374663755	-3.297408536	-2.355777621	-3.906661797	-5.0663663	-3.07379504	-1.657451907
1_P530	-2.539258228	-4.237677012	-4.82452087	-5.604265936	-3.881874979	-5.482581388	-4.978161653	-3.897585581	-2.600386559	-4.878680244	-5.13990031	-2.157891662	-2.734786918
1_P608	-4.037916143	-3.991095414	-4.108795687	-4.333502191	-4.359161111	-5.393767081	-3.941351169	-2.378391722	-2.261937935	-3.846482543	-4.871137662	-3.240561076	-1.728898558
1_P1040	-2.265313743	-3.998183837	-3.576612442	-4.175289832	-4.225837648	-5.63593613	-4.613861182	-3.007727331	-2.098558332	-4.558024404	-3.836773796	-3.772551227	-2.093174811
1_P891	-3.814693665	-4.926735335	-3.532168319	-4.231195457	-4.727375467	-6.424908989	-4.783094427	-4.053434437	-2.189355177	-4.519057579	-4.72398453	-4.099856653	-2.362256858
1_P917	-4.180892035	-3.938930586	-4.086239603	-4.728241949	-4.363642002	-6.13458727	-4.538003764	-2.975735158	-2.57082116	-4.958859271	-4.890563369	-2.441225076	-2.39764699
1_P986	-3.142124175	-4.280305882	-3.844544926	-4.849406212	-4.35125489	-5.426756003	-4.554627516	-3.069580505	-2.315396772	-4.314506816	-4.961572331	-3.472676045	-2.247447666
1_P1031	-4.024561887	-3.324147935	-4.107740783	-4.756927019	-4.010462782	-5.622496444	-4.193610765	-2.589442606	-2.604101799	-4.024031018	-4.355710423	-2.69080294	-2.189493248
1_P1032	-4.392704309	-4.048393904	-4.35125357	-4.839540527	-3.770698007	-5.51290967	-4.190810928	-2.204171958	-2.404363326	-4.320163946	-3.667319613	-4.005109316	-2.514260636
1_P1033	-4.976848954	-3.962637361	-4.620442399	-4.963289232	-4.293919881	-5.72964653	-3.69367755	-1.652001074	-2.528970891	-4.283088304	-3.813681302	-4.052857895	-2.41163036
1_P1034	-3.122147066	-4.023271231	-4.051484264	-5.003152028	-4.29368635	-5.595824588	-4.30168479	-2.822834753	-2.236295129	-4.407219669	-3.890302428	-3.72246098	-2.133015678
1_P1035	-2.541688028	-3.834089392	-3.699261112	-4.146699926	-4.188235325	-5.354360541	-4.110825501	-3.052767154	-2.363476356	-4.114279381	-4.635225979	-3.225527493	-2.16548976
1_P1038	-4.246456463	-3.910333115	-3.872794602	-4.505551016	-3.972988874	-5.599229077	-4.601416056	-3.088917341	-2.225754334	-4.635263143	-3.912458261	-3.326901043	-2.438624705
1_P1039	-3.497989099	-3.988344513	-4.236160469	-4.744017838	-4.257386043	-5.310656703	-4.118319529	-2.039892566	-2.517988957	-4.418253132	-4.763256982	-3.574853362	-2.562650594
1_P1041	-1.572137932	-3.937659489	-3.764674623	-4.2374249	-4.09544581	-5.380945641	-4.429098578	-2.969680748	-2.318946328	-4.45243011	-3.859422178	-3.559254818	-2.318966034
1_P1042	-2.882756401	-3.87306785	-3.628125415	-4.273496613	-3.921403376	-5.338155129	-4.504190802	-2.879747714	-2.160160114	-4.475692279	-3.738388804	-3.875509143	-2.23321269
2_P29	-4.978852636	-4.38901105	-4.294822022	-5.290342322	-4.111880429	-5.967645475	-4.753407433	-3.690755955	-2.479189844	-4.426581684	-5.314031103	-2.904211732	-2.642000963
2_P138	-7.581609251	-3.985986364	-3.594277879	-3.921779268	-4.134931057	-5.452083084	-3.956700073	-3.040753238	-2.542352347	-4.070715113	-4.976003858	-2.445522842	-1.960625001
2_P152	-3.388890151	-4.096609535	-3.935752914	-4.666876897	-4.135841639	-4.910995173	-4.568970016	-3.406987787	-2.706899648	-4.501475035	-4.49386206	-1.843003562	-2.425224967
2_P154	-3.837150718	-3.670385294	-3.941876832	-4.501826573	-4.002210946	-5.687881957	-4.59118972	-3.328263394	-2.576557178	-4.306970624	-4.259428519	-1.698958175	-2.437499244
2_P155	-6.167360235	-3.646022791	-4.383455857	-4.787219348	-4.057400322	-5.560393631	-4.411024987	-3.079928125	-2.690425933	-4.319342104	-4.673569627	-1.981551633	-2.500508341
2_P182	-6.486292391	-3.458854135	-3.255341066	-4.052731407	-3.63630069	-5.302832803	-3.992586712	-2.436385059	-2.701524249	-3.79938151	-4.497077618	-2.202047576	-2.360007557
2_P199	-5.235476168	-3.933387585	-4.050515981	-4.555632016	-3.810472294	-5.330350928	-4.158081034	-3.539396086	-2.410586339	-4.310712773	-4.657041086	-1.933939739	-2.30869904
2_P219	-5.661325584	-4.716638084	-4.05251172	-4.519632103	-4.230976998	-5.760392408	-4.629883123	-4.392042889	-2.716327931	-4.322695121	-5.226461955	-3.101919996	-2.217986637
2_P239	-5.107367127	-4.770892852	-3.838534009	-4.528345316	-4.016652063	-5.931634288	-4.746018501	-4.394817836	-2.172736469	-4.743101985	-4.941825972	-2.813524682	-2.292844108
2_P246	-6.149153542	-5.000873032	-4.340450789	-4.834278619	-4.87454621	-6.522153749	-4.78599033	-2.962006825	-2.839018688	-4.661939615	-5.253658264	-3.316597386	-2.844761969
2_P257	-6.749923692	-4.668779739	-4.282647329	-5.005944071	-4.848654529	-6.15428813	-3.67645342	-2.163920497	-3.07880498	-4.971028187	-5.175845188	-3.671425447	-2.436032607
2_P259	-3.612551248	-4.36971211	-4.980162849	-5.490132275	-4.650254746	-6.197352584	-4.101198729	-3.500489329	-2.867421051	-4.644277701	-4.805525745	-2.061973735	-2.562088381
2_P271	-5.38357976	-3.205232196	-3.788758012	-4.263053872	-4.217158169	-5.517281426	-3.917070589	-2.20734797	-2.947088167	-4.431179817	-3.83111527	-1.757865687	-2.149781556
2_P312	-3.475279606	-4.608513347	-3.877247746	-4.972231412	-4.301975471	-6.184675212	-4.859507382	-4.553854543	-2.344973554	-4.454156972	-5.087745013	-2.803560167	-2.527330402
2_P515	-3.355805729	-4.060075428	-4.096291935	-4.838166893	-3.903221242	-4.872758417	-4.31934804	-3.067075648	-2.6861928	-4.301604638	-4.381528953	-1.963805374	-2.443587131

2_P1037	-2.766348426	-3.841184567	-3.787064747	-4.439027874	-3.980457827	-5.217263916	-4.032564593	-2.711195707	-2.385283058	-4.215118629	-3.591639564	-3.421551523	-2.179549571
2_P1028	-2.600086908	-3.816368797	-3.850250869	-4.403165383	-3.722624218	-5.214310832	-4.10360442	-3.293523222	-2.111398675	-4.093408266	-3.457177448	-3.413708353	-2.349598644
2_P1029	-2.352015878	-3.826499431	-4.109955493	-4.332194544	-3.891771773	-5.237581702	-3.989080866	-3.327951036	-2.146008528	-4.183306588	-3.560539429	-3.165105507	-2.155660798
2_P1030	-2.842898895	-3.888580172	-4.239870883	-5.010924296	-4.227357648	-5.726738064	-4.141990085	-2.997574241	-2.284255171	-4.357656979	-3.896743908	-3.572196801	-2.205663606
2_P1036	-2.711289347	-4.265891093	-3.605440714	-4.292608037	-4.261332633	-5.843871528	-4.297632113	-3.034809648	-2.252704557	-4.302047525	-3.956804569	-3.875945064	-2.142780115

hsa-miR-1226-5	hsa-miR-1227-5	hsa-miR-1228-3	hsa-miR-1229-3	hsa-miR-1229-5	hsa-miR-1231	hsa-miR-1233-3	hsa-miR-1234-3	hsa-miR-1236-3	hsa-miR-1236-5	hsa-miR-1237-3	hsa-miR-1238-3	hsa-miR-1238-5	hsa-miR-1246
-6.346560522	0.316111696	-2.780744332	-3.680516066	-0.979993756	-3.652106267	-4.798229308	-4.552504563	-4.423925197	-5.140053594	-3.910002013	-4.035594136	-2.989812476	-1.0892144
-5.885523915	0.539205728	-2.996631241	-3.950738291	-1.293263172	-2.75190657	-5.038130231	-4.614546198	-4.528529027	-4.989864026	-4.048654974	-4.452172263	-3.464432508	-3.34870587
-5.365903	0.378317399	-2.907146446	-3.90596077	-1.092697807	-2.648172403	-4.745521835	-4.537635726	-4.608879255	-4.462045274	-3.985036296	-4.257192874	-3.370008723	-2.640627891
-5.515352751	0.512549224	-2.926267711	-3.822011208	-0.957856966	-2.773525937	-5.022258004	-4.599438033	-4.570721629	-3.988154662	-3.979463761	-4.124359245	-3.212687413	-4.698490226
-5.823838133	0.457192881	-2.542039608	-3.644367885	-0.739033112	-2.952978497	-4.645727848	-4.308260505	-4.342035433	-5.124993722	-3.622647367	-3.710969988	-2.903142038	-2.172880452
-5.826545748	0.210718482	-2.736835512	-3.648850095	-1.216772653	-2.77407736	-5.048608915	-4.396177697	-4.79721257	-4.740613162	-3.91415174	-4.034807175	-3.293771564	-3.112972143
-5.467194224	0.492789386	-2.60219538	-3.583169158	-0.946308751	-3.064750987	-4.910134019	-4.41378562	-4.506917027	-4.132928188	-3.656720012	-3.950295666	-2.990437254	-2.444624864
-5.375095971	0.329713658	-2.949254294	-3.840511437	-1.415345271	-2.654770885	-4.850916635	-4.535210376	-4.507226387	-4.28857676	-4.123576861	-4.291452045	-3.422437215	-4.19981554
-5.756370783	0.254438212	-3.026664246	-3.948206175	-1.334570605	-2.741760302	-5.143486072	-4.860972951	-5.002766407	-4.708679506	-4.102658148	-4.249926567	-3.44349893	-3.503462207
-5.549484881	0.368126892	-2.852710942	-3.817926532	-1.511481517	-2.745742828	-4.959610849	-4.724886729	-4.598323709	-4.506604767	-4.00595627	-4.188316079	-3.422221115	-3.598875005
-5.445774659	0.424759741	-2.987844711	-4.333882489	-1.428196067	-2.803492551	-4.859584302	-4.687317807	-4.925239322	-4.338390816	-4.109623068	-4.394707952	-3.277412142	-5.223689049
-5.676131726	0.339573743	-3.252514741	-4.366528224	-1.089290751	-2.91391723	-5.341793012	-5.090646772	-4.799147504	-4.514085627	-4.30475835	-4.556075966	-3.692721938	-3.154265803
-5.803701901	0.210809923	-3.335484338	-4.539769225	-1.57314559	-2.989652509	-5.339696823	-5.02689998	-4.908711423	-4.487690855	-4.304895978	-4.583015446	-3.668664344	-3.411089607
-5.660856161	0.305856162	-2.973989394	-4.319644321	-1.386976926	-2.860386911	-4.925151864	-4.71554597	-4.845254149	-4.033798212	-4.149752406	-4.460923583	-3.597438372	-6.178850183
-6.032189742	0.459579505	-3.18005702	-4.401385443	-1.670518793	-2.87873167	-5.255514654	-4.877203275	-4.945896881	-4.570453622	-4.317557926	-4.501297504	-3.629285025	-3.515373449
-4.996692934	0.355810078	-3.241405034	-4.402940537	-1.563625988	-2.459876264	-5.163952686	-4.864053912	-4.994124577	-4.079513389	-4.218684292	-4.517567736	-3.518157161	-3.605230374
-5.649231746	0.320496332	-3.176889873	-4.460758697	-1.712184499	-2.729100532	-5.140351608	-4.819028772	-4.91924341	-4.04552441	-4.131777874	-4.484096214	-3.597010704	-2.979171533
-5.415489244	0.336258013	-3.092721707	-4.585358805	-1.524403582	-2.794566499	-4.968438993	-4.753493131	-4.819985854	-3.43370537	-4.24604127	-4.763588798	-4.056693701	-2.758341726
-5.785195488	0.414110117	-3.063305944	-4.39518171	-1.257064076	-3.032793605	-4.773602767	-4.631636142	-4.821790117	-4.384853695	-4.225323409	-4.331975244	-3.365437831	-2.269987918
-5.630308861	0.42058196	-3.141558886	-4.370420857	-1.501989392	-2.756050673	-5.201499415	-4.859916928	-4.914664867	-3.864940302	-4.217961114	-4.415162022	-3.43523972	-2.699015143
-5.771246245	0.315008302	-3.14018895	-4.496450847	-1.742345586	-2.977262496	-5.251495526	-4.651391611	-4.935339259	-4.334172636	-4.156933169	-4.518923592	-3.50801917	-1.88951415
-5.807524158	0.540799966	-3.203655637	-4.563984116	-1.647161566	-2.592908441	-5.207791377	-4.797089521	-4.993569729	-4.534818525	-4.248738424	-4.595512273	-3.60674151	-3.529338529
-5.897886305	0.439813096	-3.019373358	-4.348969408	-1.068903336	-2.659400897	-5.066405438	-4.660969685	-4.810198005	-4.573308846	-4.100338278	-4.31296719	-3.491249861	-3.839049787
-5.441596177	0.661510854	-2.818971179	-3.841016277	-0.775102885	-2.956563621	-4.748113509	-4.622131465	-4.286413232	-4.042743352	-3.822348752	-3.996775369	-2.837064283	-3.023002372
-5.132891055	0.343517618	-3.10364848	-4.422792337	-1.445612204	-2.549957046	-5.138270431	-4.8041829	-4.926953782	-3.613775749	-4.255788305	-4.483748982	-3.604425091	-4.918350151
-5.722279664	0.184227491	-2.635981806	-3.937303369	-0.914387391	-2.894497352	-4.8576643	-4.526846126	-4.375079823	-4.215098035	-3.79505403	-3.97024305	-2.906319156	-2.888021417
-6.257794862	0.231218027	-3.277288483	-4.275101015	-1.579899197	-3.126155721	-5.402193626	-5.085860368	-4.984351298	-3.942525793	-4.505881285	-4.561174918	-3.874247124	-4.323654133
-6.205542207	0.363979809	-3.050532633	-4.153926125	-1.19693094	-3.382750658	-5.044468953	-4.770767565	-4.459997951	-4.004304846	-4.003973189	-4.319249794	-3.462180302	-3.391932438
-6.655675096	0.253572024	-2.840094654	-3.878228069	-1.551421311	-3.345334647	-5.073809519	-4.838009317	-4.912012263	-5.49935412	-4.172899315	-4.401610976	-3.885740622	-3.407034548
-5.965039079	0.210940571	-3.048219165	-3.925036241	-1.299579569	-2.599723864	-5.116774168	-4.771616598	-4.463391232	-4.128007994	-3.963404554	-4.312973535	-3.605656432	-4.421277184
-5.499292435	0.311045879	-3.19436046	-4.194819908	-1.402995994	-2.665654239	-5.163154118	-4.916408154	-4.640121875	-3.746603272	-4.117199689	-4.359716525	-3.37848367	-4.298099879
-9.112205787	0.11615107	-3.781182344	-4.786253622	-1.942048374	-3.997547936	-5.83180731	-5.51750611	-5.084379375	-3.3094989	-4.700300489	-5.031324116	-4.202595361	-5.360264774
-5.357812461	0.17415884	-3.361497171	-4.389801792	-2.247029146	-2.457822423	-5.507152777	-5.078611215	-5.136948881	-2.973363723	-4.423726761	-4.589367539	-3.695642218	-5.021537976
-5.182840813	0.557366021	-3.296246687	-4.519301296	-2.068267226	-1.674706735	-5.254033708	-5.054962226	-5.360387258	-3.700174585	-4.210116106	-4.350953134	-3.815160624	-7.776622799
-5.581120295	0.364695704	-3.315931488	-4.812199725	-2.122865271	-2.590588738	-5.24950865	-5.141744	-5.277261305	-3.586889819	-4.157087669	-4.739743687	-4.375018922	-6.708496657
-5.582594706	0.443565071	-2.811994013	-4.03928287	-2.117485928	-2.205761553	-4.953729797	-4.633993056	-4.846953591	-4.366145268	-3.665432668	-4.026416623	-3.847103942	-3.620561441
-5.888442111	0.272194219	-3.294595715	-5.22757793	-2.332542351	-2.632875937	-5.677291672	-4.416291832	-6.694697304	-4.000028822	-4.414502954	-4.660157497	-4.073221801	-3.265328374
-5.163228084	0.463727551	-3.001383662	-4.290531056	-2.023696158	-2.210731766	-4.757842691	-4.573774084	-4.735418638	-3.928121105	-3.88144514	-4.405172507	-4.151445371	-5.7798701
-4.970396538	0.540572299	-3.019081895	-4.117077579	-1.593839007	-2.435082641	-4.802871302	-4.746918141	-4.788989805	-3.498990283	-3.996370357	-4.367513326	-3.473321197	-5.549614248
-6.08869298	0.301810609	-2.98682015	-4.362399621	-2.49593178	-2.817113252	-5.017286595	-4.62953104	-4.933969734	-4.724433823	-4.131648678	-4.599115036	-4.10108911	-3.102820947
-5.380870158	0.233893036	-2.908376941	-4.170773395	-1.843174601	-3.100675615	-4.802440777	-4.658019745	-4.782525725	-3.970774473	-3.873722519	-4.383263964	-3.91455559	-2.452453428
-5.370470291	0.553664984	-3.239504117	-4.442872408	-1.755142593	-2.119679505	-5.147778252	-4.761897987	-4.954670408	-2.525286468	-4.114984641	-4.799218825	-4.065091011	-5.048816503
-5.804475561	0.212152448	-3.504678771	-4.647213437	-2.261263315	-2.218747158	-5.421255188	-5.358236737	-5.475453373	-3.027054869	-4.582716182	-5.019469899	-3.979338103	-4.26214217
-6.558398964	0.531384849	-2.128819076	-3.728189334	-0.908598946	-3.444610057	-4.345891532	-4.08869846	-4.087191786	-5.60193751	-3.309220734	-3.360965666	-3.023241936	-0.436921805
-4.907226172	0.459636796	-3.264972998	-4.461621077	-1.966150292	-1.939230104	-5.289424371	-5.044678788	-5.341945795	-3.715311359	-4.367400909	-4.689650547	-3.868456052	-4.681660697
-4.801410928	0.356721932	-3.146396646	-4.407458917	-2.162708088	-2.098154148	-5.17680895	-5.138948941	-5.154424913	-3.795160794	-4.21195111	-4.588879962	-4.569579172	-4.414576196
-4.657488808	0.53322891	-2.886074433	-4.150183679	-1.646773135	-2.467516608	-4.922269112	-4.728184886	-4.787652953	-3.666647561	-3.891565018	-4.161516217	-3.594381243	-3.198190533

-5.301272769	0.414670882	-3.279987808	-4.367177267	-1.713088069	-2.010076993	-5.049396826	-4.96560017	-4.786129793	-3.153253786	-4.069710271	-4.756997344	-3.907241452	-4.425276126
-5.390460964	0.648802282	-3.069926887	-4.608743085	-2.325310254	-2.039998868	-5.367578816	-4.701291785	-5.558522442	-3.919093369	-4.313795637	-4.587624349	-3.478919157	-2.980818829
-5.16532242	0.529203527	-3.079635109	-4.083321787	-2.101035467	-2.394782498	-4.794757364	-4.516504153	-4.674214294	-3.35403456	-3.986247228	-4.525061163	-3.71468468	-4.624208777
-6.641278185	0.84397344	-2.465286568	-3.819587492	-0.792461805	-3.071182481	-4.371176753	-4.177630789	-3.955836742	-4.407244716	-3.218393733	-3.742715633	-2.836032447	-0.269271324
-6.419086105	0.450491415	-2.835242939	-4.11082422	-1.440658975	-3.032872484	-4.995756022	-4.978565678	-4.803738228	-3.949919206	-3.967365636	-4.099519011	-3.643296412	-2.072782984
-5.346536845	0.577921253	-3.400752023	-4.421619955	-1.851206421	-2.172356614	-5.126629331	-4.546350257	-4.458809548	-3.041313474	-3.818910791	-4.859341405	-3.18610374	-3.42046078
-4.93272311	0.594764873	-2.656566747	-4.002411071	-2.297511641	-2.268922339	-5.561016354	-3.814705723	-5.298200517	-4.055592246	-3.504203918	-4.971208648	-2.913770299	-3.847255196
-5.731923473	0.441703251	-2.83691538	-4.147630765	-1.867543657	-2.889026305	-5.01023701	-4.869275227	-5.028565831	-3.944797442	-4.108866126	-3.97232096	-3.776347941	-2.384715338
-5.352544988	0.574928924	-3.131405322	-4.104126891	-2.061795356	-1.875819086	-5.037714675	-4.810320062	-4.932884407	-2.865574813	-4.085236209	-4.568091129	-3.908263589	-7.358633905
-4.931897578	0.507157638	-3.244331185	-4.313210222	-1.975127191	-2.006061618	-5.048027058	-5.090210098	-4.983204735	-2.768446438	-4.108925136	-4.743828038	-3.956863263	-4.564384831
-5.669693022	0.389477412	-3.252660637	-4.393950348	-1.71066107	-2.757380789	-5.295769113	-4.8246942	-5.142194508	-2.334705749	-4.344679395	-5.103924416	-3.998350192	-3.622571425
-4.694646214	0.363820797	-3.303603404	-4.39744321	-1.942365358	-2.479087623	-5.217010761	-5.062390578	-5.186830667	-2.761147782	-4.333270856	-4.419207187	-3.652167261	-5.318887348
-6.398991815	0.253945565	-3.174872913	-4.610654677	-2.248734287	-3.180131355	-4.930135474	-5.097580685	-5.084080952	-4.877933566	-4.4034034	-4.707312726	-4.503956198	-3.485211466
-4.997942022	0.308038239	-3.404328769	-4.797487413	-2.399918981	-2.770317909	-5.117651655	-5.038045532	-5.042572809	-2.804101628	-4.522284907	-5.123112507	-3.839430095	-6.259461704
-5.279713375	0.270959823	-3.643593514	-5.040174443	-2.403742063	-2.782973492	-5.203333564	-4.270158333	-5.251088547	-3.52096729	-4.15131714	-5.541630917	-3.759854674	-4.745517885
-4.932762818	0.292349982	-3.220190147	-4.33740762	-2.156011548	-2.423646796	-5.666434783	-4.958276031	-5.337802638	-3.751311713	-4.314905191	-5.117099741	-3.770903815	-4.477535642
-5.378467856	0.297109639	-2.974468311	-4.43770525	-1.927229827	-2.475547467	-4.971793956	-4.508271976	-4.824388774	-4.471105278	-3.881273767	-4.623899591	-3.989022722	-4.44167211
-6.753502167	0.550363165	-2.525004477	-3.582748273	-1.68789516	-3.168783599	-4.624833269	-4.566137466	-4.817365196	-4.900633198	-3.743104042	-3.941363019	-3.82419223	-1.722793531
-4.780061442	0.567177402	-2.921809618	-4.225770082	-1.952429623	-1.804134115	-5.108032843	-4.991543823	-4.841239119	-3.836176342	-3.970257889	-4.285812922	-3.660038021	-5.573112297
-5.444310167	0.175683439	-3.533119465	-4.743019829	-2.10089171	-2.029037596	-5.797614367	-5.118028899	-5.4586987	-2.885823297	-4.599367802	-5.124428177	-4.036595569	-4.037530081
-5.638236923	0.281202628	-2.961569331	-3.909933369	-1.712783337	-2.74660493	-5.042580812	-4.832792186	-4.704672632	-4.414389921	-4.05536188	-4.319572611	-3.727872234	-1.992471029
-5.90270422	-0.030700458	-3.814537158	-4.612809808	-2.103414638	-2.883667899	-5.967218226	-5.327372868	-5.389366278	-3.668682121	-4.750902361	-4.875377756	-3.471260131	-4.142493769
-6.000897272	0.035201627	-3.774295704	-4.808137338	-1.832009452	-2.590521495	-5.613118182	-5.559742212	-5.107613903	-3.158607025	-4.603252153	-5.041745634	-4.137701186	-6.288616099
-6.355201896	0.253365913	-3.947666815	-5.035479815	-2.25824792	-2.082579477	-6.842770927	-5.957581008	-5.986724709	-3.550247576	-5.036829186	-5.607498902	-3.930805484	-6.159890175
-6.437278011	0.170891329	-3.527560236	-4.590628925	-1.760435389	-2.515808355	-5.565836939	-5.338190032	-5.320467245	-3.202851626	-4.574367972	-4.773116034	-4.18248337	-6.097197443
-4.956968754	0.129626349	-3.097645993	-4.455654009	-2.054059451	-2.581792743	-5.188387256	-4.16940893	-5.24569479	-4.209286099	-4.16473267	-4.770444066	-3.585507845	-4.797489998
-6.346911318	-0.080514337	-3.647440853	-4.397445234	-2.104551573	-2.949518625	-5.863057153	-4.972876672	-5.20541743	-5.096805028	-4.985351184	-4.870017954	-3.056533454	-3.524231161
-6.812817836	-0.025785606	-3.533934625	-4.523071175	-2.19610561	-2.914556969	-5.763620452	-5.332026454	-5.353698005	-5.272723809	-4.767416322	-4.738764526	-3.381439252	-3.150951127
-5.90307146	0.04216142	-3.613998296	-4.472429029	-1.887886425	-2.783386736	-5.54721817	-5.081863202	-5.290405182	-3.775448405	-4.68959819	-4.744850157	-3.552728808	-4.245365886
-5.580663891	-0.162009793	-3.247826125	-4.495454484	-1.947571845	-2.59547735	-5.342364148	-4.784624819	-4.962689443	-3.525662062	-4.228665399	-4.664156984	-3.639552319	-4.813759093
-5.599519991	0.067824158	-3.664908038	-4.580506169	-2.030539167	-2.55064166	-5.721565589	-4.921766683	-5.328754219	-3.764941457	-4.866744885	-4.796288889	-3.082108255	-3.58364237
-6.565462089	0.041413773	-3.326317261	-4.235411418	-1.910886743	-2.935797161	-5.402268878	-4.551073724	-5.167761246	-4.571573088	-4.241880978	-4.801870446	-3.349991684	-2.863154234
-5.780409282	-0.107179825	-3.701288682	-4.522884786	-2.063909038	-2.746416207	-5.807373438	-4.953009155	-5.440081993	-3.569012762	-4.878154932	-4.857749374	-3.498526525	-3.573909612
-5.512799242	-0.031744218	-3.58157911	-4.616658197	-1.927567394	-2.587861866	-5.595466146	-5.053051632	-5.489531325	-3.968303986	-4.739598297	-5.000318292	-3.245978978	-3.847751182
-5.856381738	0.411952705	-3.369190651	-4.368165277	-1.856145726	-2.550091969	-5.35190531	-5.246682509	-4.983759219	-2.985742978	-4.239429294	-5.032835595	-4.136865416	-4.76929452
-4.965400165	0.747400803	-2.959138985	-4.127099691	-1.702939815	-2.033797598	-4.619412369	-4.513017089	-4.5247011	-3.468498473	-3.575303764	-4.273882262	-4.132154603	-5.129461407
-4.87953888	0.371383336	-3.197750901	-4.636272324	-1.756556773	-1.98225616	-5.017028215	-4.50984564	-4.49733041	-2.458713206	-3.984695385	-4.884274466	-3.790867939	-4.991984566
-5.0789275	0.256495266	-3.087145998	-4.585429797	-2.097349672	-2.348298287	-4.843516006	-4.505574057	-4.783862871	-3.57634708	-4.108771425	-4.697149132	-3.497102981	-4.545282152
-4.942116582	0.115413298	-3.152967125	-4.39365607	-2.572696928	-2.307614219	-4.849087135	-4.93793954	-5.039612036	-3.774620926	-4.230855317	-4.729572318	-4.353975837	-5.374657857
-4.769840338	0.363737516	-2.814853914	-4.343282322	-1.863751197	-2.294806679	-4.963626699	-4.669970007	-4.899382988	-5.192645003	-4.108175567	-4.208763874	-3.707484929	-5.701107511
-5.117322221	0.388875761	-3.160314048	-4.521305097	-1.98841016	-2.474778639	-5.146090359	-5.001297362	-4.916221933	-2.692081131	-4.162267943	-4.596312533	-3.85259399	-6.051810409
-5.405906811	0.408862818	-3.47324634	-4.430674044	-1.827571012	-2.333362319	-5.238469109	-5.311564102	-5.20804911	-2.235867773	-4.332344601	-4.95837979	-4.038309697	-6.196866316
-5.678086927	0.624199878	-3.486238703	-4.806877387	-2.081316237	-2.577690109	-5.303389826	-5.531785656	-5.172640578	-2.550006814	-4.575356126	-5.161055422	-3.877280354	-3.903122346
-7.128387931	0.060299979	-3.40930085	-4.016496627	-2.457894555	-3.36982804	-5.577488739	-5.506716079	-5.289698511	-3.4268081	-4.519023915	-4.973294484	-4.488998435	-3.876966478
-6.677563857	0.341225807	-3.67058937	-4.735380553	-2.169575101	-3.218720294	-5.418131993	-5.58962877	-5.129676824	-3.723660693	-4.552398661	-5.356567117	-4.549874399	-4.399719729
-5.038281353	0.260798468	-3.440925285	-4.707374405	-2.018356255	-2.392084012	-5.366568392	-4.962635107	-5.28035557	-2.67877485	-4.720504562	-5.242584683	-3.95483803	-3.758102017
-5.336159701	0.351346659	-3.080561541	-4.699784224	-2.389812985	-2.345739184	-4.795039463	-4.532664155	-5.618101951	-3.627364032	-4.100025636	-4.94571379	-3.52076507	-6.283529158
-5.782639436	0.607526188	-3.152122747	-4.425204298	-2.048199861	-2.312090423	-5.089148141	-4.863946965	-4.803102274	-2.988270247	-4.030882791	-4.775599703	-3.904725162	-0.375766538
-5.276718809	0.213062349	-3.354360313	-4.566274909	-2.156141514	-2.282071984	-5.518544822	-4.934796138	-5.4781815	-3.298805252	-4.457320476	-4.949497615	-3.553191483	-4.425648911

-5.475409699	0.068403686	-3.394899042	-4.243370888	-1.940581807	-2.618065407	-5.761423254	-4.894318079	-5.353309408	-4.107661882	-4.722524443	-4.545571745	-3.048914247	-3.873188112
-5.451248456	-0.011345024	-3.561890542	-4.408409442	-1.947872582	-2.808254666	-5.263013022	-4.696799323	-4.731919164	-3.705142399	-4.629587901	-4.569988492	-3.022196301	-4.058741125
-5.328775557	0.295969746	-3.477470964	-4.347741098	-1.905145789	-2.330107362	-5.49617655	-4.812987767	-5.093638832	-3.066667437	-4.718322655	-4.713520489	-3.290453873	-3.863129642
-6.107440708	0.181064865	-3.458033938	-4.250598736	-1.896078682	-2.609940352	-5.320354126	-4.774402361	-4.849320468	-3.634301803	-4.556755931	-4.773879054	-3.274254242	-3.527201903
-5.894717053	0.026498094	-3.709683223	-4.578367877	-1.986189857	-2.851209116	-5.611668359	-5.300316199	-5.402571802	-3.686701902	-4.89628445	-4.752408361	-3.519743353	-5.03487638

hsa-miR-1247-3	hsa-miR-1247-5	hsa-miR-1249-3	hsa-miR-1249-5	hsa-miR-1254	hsa-miR-1260a	hsa-miR-1260b	hsa-miR-1268b	hsa-miR-1273g-	hsa-miR-1275	hsa-miR-1281	hsa-miR-128-1-	hsa-miR-128-2-	hsa-miR-1292-3
-4.383659513	-5.759893646	-2.814971891	-1.508469895	-5.607348031	-3.428357409	-2.561492429	0.905143576	-1.703226545	-1.332301257	-3.803834618	-1.752461833	1.156221098	-4.62928372
-3.898503221	-5.763907829	-3.12320678	-1.709048292	-5.390806386	-3.410018881	-1.878283102	1.188494975	-2.485751662	-1.763598983	-3.992373264	-1.428095587	0.625728844	-3.953279476
-4.250048349	-5.606044341	-2.844695649	-1.722405269	-5.206811505	-3.232454384	-1.328310348	1.214237585	-1.002351918	-1.172528748	-4.082611474	-1.812020342	0.48526139	-4.503163309
-3.927681587	-5.815922166	-2.972218089	-1.751181793	-4.830322321	-2.994671398	-1.049668731	1.307452798	-1.916795809	-1.460859773	-3.913411487	-1.689402591	0.747698231	-4.456507544
-3.858737415	-5.426859374	-2.451580639	-1.38946539	-5.360569935	-3.218647116	-1.976375965	1.116427585	-2.018862511	-0.96587627	-3.536888753	-1.590226557	1.239440438	-4.264341
-4.074410404	-5.633566163	-2.823076913	-1.764216638	-5.11450596	-2.887504992	-1.267998755	1.351258592	-2.104011338	-1.624508764	-3.784578601	-1.808557071	1.05138988	-3.982264598
-3.723334444	-5.46970265	-2.698992562	-1.623323924	-5.142799896	-2.768320959	-1.15705149	1.264449882	-1.339926394	-1.465879272	-3.557596132	-1.662412185	0.939356901	-4.144710674
-3.720240429	-5.818505981	-3.127705419	-1.827492269	-4.718745409	-2.941143827	-1.002423216	1.342486879	-2.439747146	-1.680488261	-3.865702665	-1.839919662	0.727250577	-4.044278604
-4.25984313	-5.657334469	-3.018323645	-1.823651992	-5.374581413	-3.23806462	-1.399833098	1.234097778	-2.29635503	-1.493478895	-4.003721843	-1.959609892	0.472810076	-4.345525003
-3.822945262	-5.795023421	-2.879519278	-1.724343619	-5.259858354	-3.053095561	-1.566034898	1.296575215	-3.271138693	-1.803296332	-3.825923028	-1.740653613	0.924265122	-4.013588909
-3.709857766	-5.699551055	-2.958499509	-2.090317269	-4.723097976	-3.081736967	-1.172549831	1.326378286	-1.811874963	-1.896026304	-4.303234283	-1.693382033	0.333945638	-4.409578675
-4.264540173	-6.322656413	-3.447908872	-1.69221637	-5.941409075	-3.798496846	-2.583163703	0.824143602	-2.527726507	-2.1661694	-3.8782724	-1.594247132	0.314013772	-4.486928756
-4.165229548	-6.370005157	-3.470057109	-1.860660455	-5.242164968	-3.65361319	-2.137895265	0.797055401	-2.394759659	-1.78280989	-4.095863684	-1.829256622	0.21457034	-4.510413127
-3.850771528	-5.774291868	-2.989452315	-1.84919822	-4.495433103	-2.990692635	-0.830363568	1.146407568	-2.389384949	-1.877361378	-4.319628835	-1.835768953	0.697211171	-4.46580749
-3.819533646	-5.951551288	-3.155139466	-1.953334502	-5.245433792	-3.180048554	-1.447920501	1.020419851	-2.593963712	-2.037343627	-4.37495575	-1.317020645	0.749319062	-4.271789854
-3.910813482	-5.742923895	-3.231818008	-1.894326466	-4.969252343	-3.53140667	-1.394053693	1.146697718	-2.315958622	-1.933359954	-4.396535697	-1.698515284	-0.02427513	-4.267902104
-3.940055269	-5.827401633	-3.237614907	-1.929836893	-4.96510427	-3.28919381	-1.520898159	0.96967997	-2.325552352	-2.015820208	-4.413510368	-1.804105595	0.57113896	-3.983756406
-3.960112954	-5.420381191	-3.163151715	-1.999030758	-5.064370207	-3.812712626	-1.870809978	1.004955138	-2.222288906	-2.112288595	-4.366561354	-2.30988013	0.15687479	-4.273418059
-3.748965337	-6.113328966	-2.964145948	-1.610808315	-4.88059564	-3.324156889	-1.321260819	0.779328972	-1.939616458	-1.672056287	-4.313357026	-1.849039892	0.797607035	-4.339798438
-3.868339212	-6.064118207	-3.061212931	-1.872014893	-4.769370641	-3.030578204	-0.975793783	1.072639427	-1.891476603	-1.760259918	-4.440967375	-1.865440535	0.510518822	-4.055078217
-3.978309937	-5.904626363	-3.187185787	-1.970165584	-5.36265501	-3.042857083	-1.37293232	1.072639781	-2.159371565	-1.987895169	-4.357582084	-1.458256769	0.635558468	-4.186436474
-3.783453127	-5.869839902	-3.163639856	-1.957754379	-5.409288903	-3.041830928	-1.334552963	0.997018274	-2.285797593	-2.028331504	-4.332181524	-1.572800274	0.756989419	-4.068152448
-3.883487214	-5.943006231	-2.968128871	-1.639920192	-4.995050383	-3.388367041	-1.011758958	0.961845619	-2.245950468	-1.525410179	-4.293820862	-1.845759743	0.721766509	-4.423097133
-3.860828578	-5.677953631	-2.797401009	-0.897583859	-5.254412583	-3.513297573	-1.654792867	0.768000724	-2.309151946	-1.144680528	-4.046283691	-1.591273456	1.465478664	-4.413245279
-3.717449852	-5.74370531	-3.07158824	-1.896027004	-4.884406271	-3.244437682	-0.907067078	1.152731392	-2.930624347	-1.703852174	-4.185930885	-1.599592834	0.544326539	-4.141453319
-4.319064061	-5.618618553	-2.551212331	-0.85402473	-5.604868297	-3.333959059	-1.077147033	0.825668891	-2.267867315	-0.753442882	-3.937078516	-1.792104371	1.352864224	-4.489137221
-4.222250023	-6.319626666	-3.41760958	-1.980379745	-5.087556699	-3.196203341	-1.128633174	0.93434039	-2.801326065	-2.030688483	-3.962516565	-1.937952664	0.211344257	-4.005096028
-4.101160208	-6.267793466	-3.056994033	-1.362677009	-5.753245723	-3.186267821	-1.150957734	0.524046348	-2.437825453	-1.596299451	-3.870097731	-1.922516515	1.084541999	-4.672479652
-4.243962103	-5.679611774	-3.019022952	-1.946954622	-6.17036157	-3.726942532	-2.4391441	0.966579868	-2.813526117	-2.055517926	-3.682019698	-1.726517257	0.872416564	-4.183393751
-3.929527761	-5.824644625	-3.040225939	-1.747732667	-4.756105704	-3.25773654	-1.183828925	1.170468391	-2.354860269	-1.771188368	-3.903142845	-1.759438365	0.251602736	-4.159819533
-3.793993735	-5.952428548	-3.252600833	-1.861662323	-4.436169403	-3.1724628	-1.094191479	1.134931668	-1.980426226	-1.835101474	-4.00631074	-1.685293748	0.393075726	-4.347938687
-4.711732938	-7.325769767	-3.760370526	-2.563276493	-5.935024889	-4.178956745	-2.889532122	-0.194993257	-2.659023146	-2.746776925	-4.497410738	-1.839505436	1.415305875	-5.814712935
-3.68638085	-5.559337467	-3.711785979	-2.569816161	-4.926459631	-3.669670372	-2.537253733	1.431704281	-2.652256605	-3.240707025	-4.290861198	-1.156924936	-0.053406756	-3.8545559
-3.967324392	-5.718429015	-3.591127378	-2.648405776	-3.859435553	-3.628577886	-1.528665881	1.74677201	-1.932835909	-3.395890104	-3.958190546	-1.109577582	-0.214289947	-3.628144332
-4.042010669	-5.798931449	-3.26385248	-2.763779131	-5.609630883	-4.435610616	-3.069238914	1.03845563	-3.243439206	-2.951781781	-4.530874397	-1.962266379	-0.189093401	-4.246181756
-4.020454623	-5.248708321	-3.159859651	-2.496245059	-5.877808928	-3.915816235	-2.553605405	1.631676058	-2.11840615	-2.622631571	-3.854925359	-1.058777446	0.553946013	-3.234663321
-3.999902433	-4.66766424	-2.854099163	-3.559234578	-4.297262343	-3.927104036	-1.38171974	1.159530836	-1.339095746	-3.084697458	-4.888791494	-1.76277947	0.518740002	-3.316603971
-3.908333409	-5.312417474	-3.079517395	-2.686406227	-4.762672526	-4.127363469	-2.376919514	1.728162777	-2.378065005	-3.516576687	-4.331092777	-1.816614512	0.141077406	-3.491035598
-3.796777739	-5.439537903	-2.931775834	-2.137354356	-4.809533252	-4.327480186	-2.85919123	1.260600152	-2.154886259	-2.133376276	-4.305496251	-1.867767076	-0.059368018	-4.090726649
-4.009594805	-4.995294307	-3.072964785	-3.05815492	-3.848407766	-4.440074325	-3.209135787	1.215115162	-1.922715877	-3.525523341	-4.350826697	-1.547840075	1.022988879	-3.739534824
-4.010086738	-5.303854729	-3.027193232	-2.338561953	-5.788858229	-4.05240676	-2.894950868	1.001567342	-2.31313787	-2.215824952	-4.209550034	-1.608817158	0.768615948	-3.822751997
-3.76802515	-5.568587196	-3.5560681	-2.065586527	-2.913446261	-3.43464245	-0.278563134	1.558336472	1.46664127	-2.80598255	-4.459999088	-2.065706039	-0.687125137	-4.121819292
-2.812556113	-5.453592091	-3.482066686	-2.700794576	-2.998064513	-3.716357461	-1.187714997	0.942248992	0.125165867	-2.765266958	-4.73683108	-2.917566406	-1.028388544	-3.971459524
-4.651040445	-5.042920323	-1.711538161	-1.697798831	-5.664098696	-4.155838617	-4.281116563	1.162553529	-0.870623309	-1.243616208	-3.790003551	-2.011782932	1.511612535	-4.588972631
-4.116895249	-5.344901279	-3.382150564	-2.197977103	-4.126599537	-4.652953765	-3.058434263	1.776316596	-1.338096599	-2.594036693	-4.574635738	-1.804374386	-0.046895856	-3.854161351
-4.183002311	-5.491184619	-3.308072964	-2.544943544	-4.79926818	-4.581673227	-3.237132378	1.556990285	-2.29416822	-2.887596506	-4.552789369	-1.843890562	0.209466779	-3.956373732
-4.148095974	-5.370590204	-2.834081012	-2.050230019	-6.286237776	-4.532339619	-3.649919846	1.334524669	-2.789203324	-1.947226721	-4.291456489	-1.876202993	0.442244193	-4.269288259

-3.587763768	-5.666163697	-3.340126016	-2.134543826	-4.240091331	-4.445489549	-2.931465462	1.494787977	-2.232844425	-2.08409724	-4.512898926	-2.03894193	-0.26224256	-4.054853298
-3.507343847	-4.819224562	-3.210864625	-2.818918206	-4.272616824	-3.734075552	-2.540206285	1.625137596	-1.677391161	-3.102426575	-4.937047154	-1.519123761	0.553753296	-3.423391636
-3.648878464	-5.255031609	-3.021806571	-2.409637653	-5.083505767	-3.899857964	-2.465486722	1.837346016	-3.654309351	-2.641771608	-4.365173012	-1.204704539	0.708030756	-3.357032312
-4.380792812	-5.50657503	-2.147855064	-1.111165517	-5.381982626	-4.174142284	-3.067524462	1.383887222	-0.9882742	-1.31790191	-3.877277655	-2.040701503	1.357222141	-4.597853312
-4.436572413	-5.56283783	-2.560430517	-1.81450795	-4.415467776	-3.991739888	-1.900322019	0.604102266	-0.736808285	-1.788130114	-4.33378712	-2.014796846	1.162260668	-4.498507422
-3.147169049	-5.829987185	-3.686739333	-2.48852291	-3.731348286	-3.644396337	-1.480756692	1.287238528	-1.737050613	-3.023644246	-4.217512535	-1.88459141	0.287160806	-3.400800583
-2.938017299	-5.397565306	-3.255358537	-2.983951259	-4.050193102	-3.859819011	-2.212149825	1.281031749	-3.056037993	-3.127581662	-4.146413082	-1.192052787	1.203665352	-2.841610408
-4.414682822	-5.550724371	-2.597532396	-2.666660975	-5.558100859	-4.156010223	-1.360907112	0.563997015	-2.230419653	-2.175580316	-4.282701079	-1.800830376	1.791509108	-4.05386404
-3.801432762	-5.164296958	-3.192463393	-2.368905186	-3.060809623	-4.173675018	-1.536729323	1.852683919	-1.747142697	-2.847048247	-4.208056235	-1.647711342	0.203209322	-3.096454215
-3.729351468	-5.39820123	-3.417488109	-2.011621663	-3.232663678	-4.063283084	-1.387093146	1.466564064	-0.449210007	-2.919782389	-4.302502178	-1.738817756	-0.281685082	-3.765483475
-3.592281394	-5.486534615	-3.534520524	-3.307504805	-2.210710922	-2.164203434	0.119677769	0.912776901	0.622173528	-2.517814271	-4.428941981	-2.196140709	0.279917469	-4.188446945
-3.813295964	-5.571065463	-3.424695024	-2.591809213	-2.719441704	-2.782719665	-0.006993215	1.368958707	0.022317191	-2.581367681	-4.20903469	-2.1004323	-0.377685027	-3.489501029
-4.64378628	-5.849556694	-3.212409258	-2.45251652	-5.307871374	-4.435498782	-2.883230587	0.602181783	-2.327400546	-3.036632634	-4.616792744	-2.003565518	0.471729413	-4.387024849
-4.20548201	-5.887501306	-3.542643405	-2.578921088	-2.723811253	-3.450679818	-0.65454657	1.408423518	-0.069891257	-3.779408547	-4.752046099	-2.322830595	-0.279654563	-3.918137258
-3.751116618	-5.163955644	-3.560143555	-3.186757409	-3.22671132	-4.055254459	-2.300405373	1.125387506	-2.740236893	-4.051246657	-5.020468036	-1.956668662	0.515504436	-3.890067934
-3.656998322	-5.267477097	-3.324701434	-2.918909163	-4.935672066	-4.544764026	-1.801415675	1.447206011	-3.706726241	-3.079764014	-4.794414869	-1.278187281	1.226436255	-3.796886702
-3.718094904	-5.394128108	-3.058446537	-2.628604237	-4.70257919	-4.646393089	-2.925179172	1.398385859	-2.430517025	-2.392891039	-4.222427761	-1.998576799	0.058131023	-3.603989964
-4.433018711	-5.325509034	-2.60960236	-2.043296907	-5.382037383	-4.066588911	-2.847289672	0.952254583	-1.451257227	-2.163201344	-3.616217123	-2.017116407	1.085275577	-4.310359258
-3.832009814	-5.427882921	-3.155272022	-2.177960074	-5.17840167	-4.508517925	-4.359165323	1.219345707	-2.603890855	-2.682522826	-4.207672028	-1.211852488	0.308447713	-4.007924655
-3.716875078	-5.698887491	-3.480274923	-2.462877045	-3.319505869	-3.142847112	-0.743996921	1.144875579	-0.407789795	-2.754623752	-4.563607901	-2.354380288	-0.687975341	-4.067314868
-4.181949054	-5.66631348	-3.105118387	-1.703971817	-4.465297331	-4.022507029	-2.459986574	1.143145902	-0.914304821	-1.915830596	-3.968968025	-1.536978645	0.292781955	-3.839499016
-3.6933763	-6.38196744	-3.977476155	-2.722333925	-3.879416898	-2.589058656	-1.139519502	0.866431626	-1.750247599	-2.369907441	-4.464257278	-1.656576747	-0.348368439	-4.395743075
-3.95655885	-6.715735246	-3.934025835	-2.246886993	-4.32374569	-4.57958062	-3.030041092	0.546636559	-0.753323306	-2.490472121	-4.316406038	-2.056819404	-0.332230109	-4.762643697
-4.196286464	-7.525673942	-4.414858478	-3.061566632	-4.900668506	-3.558677762	-1.724745686	1.225629609	-3.305971915	-3.108577294	-4.305701029	-1.168469724	0.147249347	-3.546981769
-3.850473948	-6.872286512	-3.614756439	-2.210648044	-5.426757109	-4.147776914	-3.116132279	0.852958933	-3.595288236	-2.172136306	-4.261641914	-1.507883038	0.115219328	-4.254589089
-3.470965492	-5.662506588	-3.055952886	-2.608144563	-3.438463096	-2.660072845	-0.816141372	1.393148458	-2.009351604	-2.150094614	-4.180249492	-2.19257181	-0.01072703	-3.623814329
-3.475477495	-6.680710241	-3.927420684	-2.549875156	-3.326914792	-2.516103103	-1.143684852	0.632259799	-2.533754513	-2.373274722	-4.139742189	-1.61994628	0.829780102	-4.378404492
-3.888432113	-6.263154101	-3.844814075	-1.930752914	-4.218849372	-3.328621547	-2.054059613	0.618416756	-2.421616376	-2.373976949	-4.255590948	-1.308229424	0.991132993	-4.135963224
-3.753840731	-6.475281104	-3.84146398	-2.51433163	-4.063936055	-2.530019387	-0.952478609	1.067278793	-1.921000527	-2.332412861	-4.235146643	-1.575205074	-0.061078521	-4.200289192
-3.612692139	-5.905102554	-3.348790124	-2.331164786	-3.771573312	-2.666956108	-0.262149736	1.450066867	-1.630951195	-2.03459744	-4.152979465	-2.313829978	-0.044291197	-3.90084037
-3.354012657	-5.781249426	-3.688435476	-2.729019905	-3.390568716	-2.33602889	-1.027817654	1.072326601	-2.214236399	-2.565536216	-4.467222903	-1.86325285	-0.148634537	-4.42281208
-3.489610534	-5.874729605	-3.565683838	-2.279731462	-3.939287106	-3.005608854	-1.757925788	0.957096666	-2.186433992	-2.513436193	-4.354440471	-1.508674216	0.832141515	-4.364020794
-3.433097296	-6.217069063	-3.926828425	-2.828206672	-3.453684682	-1.855287485	-0.501463059	0.866939836	-0.592859633	-2.365424598	-4.383618853	-1.73230217	-0.143950677	-4.474131931
-2.733723372	-6.412143074	-3.791763901	-2.458288063	-2.982330379	-2.489916412	-1.47348731	1.007295211	-1.933383593	-2.502375503	-4.48288522	-1.653449385	-0.034132878	-4.411981811
-4.114509117	-5.950715446	-3.417937233	-2.536403186	-4.055069093	-3.980970335	-1.093563316	1.238649296	-0.889080961	-2.681185516	-4.473781285	-1.989860683	-0.207078973	-4.539609623
-4.007021112	-5.24453236	-3.072388774	-2.036136554	-4.581571004	-4.349873493	-2.720697603	1.843814608	-2.281755567	-2.618351391	-3.977669961	-1.610645013	0.548166977	-3.83042073
-3.225603967	-5.199834241	-3.707156828	-2.406745711	-3.071766156	-3.548831706	-1.202244444	1.469691248	-0.458965464	-2.957186285	-4.398854844	-2.31749483	-0.120980851	-3.84253918
-3.607068244	-5.044988801	-3.065349665	-2.57708341	-3.645730283	-4.375391572	-2.396509896	1.636885602	-2.103453199	-2.624603177	-4.442687015	-2.037120857	-0.142623209	-3.423637665
-4.058840191	-5.145925922	-3.335399168	-2.504651863	-4.853572416	-4.55603734	-2.900034126	1.581162292	-2.751379479	-3.261500865	-4.625275459	-1.792063567	0.044912343	-3.526693244
-3.660928064	-5.298479314	-2.848273088	-2.873847466	-4.328611118	-4.299091332	-3.628723051	0.911895876	-3.915619872	-2.587900035	-4.319837126	-1.439807797	2.54804506	-3.435304814
-3.853637186	-5.346251889	-3.201174987	-2.571544854	-2.71571241	-3.597956724	-0.517059636	1.688980607	-0.204589188	-2.771956738	-4.585853669	-2.053947435	-0.268757854	-3.679140037
-3.752056968	-5.81434841	-3.708821334	-2.12860556	-2.726411912	-3.28138448	-0.500131389	1.364242978	0.158040555	-2.667990303	-4.493380168	-2.391027416	-0.276754293	-4.028898273
-3.957267738	-5.912124846	-3.64965633	-2.275556063	-2.433761067	-3.005036131	-0.688242194	0.781378035	0.229157311	-3.018247284	-4.827101701	-2.599786097	-0.932113291	-4.211588231
-4.66247862	-5.97395726	-3.493197305	-3.152537196	-3.979486119	-3.468743069	-1.329039305	0.368855359	-0.9967097	-3.059228589	-4.899157143	-1.790422217	1.434913673	-4.631863467
-4.677599945	-5.774267742	-3.856018552	-2.322410019	-4.267146623	-3.673886273	-1.609687457	0.370089511	-1.715543842	-3.344929357	-5.018414813	-1.701485394	0.960981676	-4.916004461
-3.601966117	-5.537910346	-3.421111007	-2.619213475	-3.320176878	-4.109269767	-1.530387273	1.206978503	-1.620462318	-2.670383013	-4.771467715	-2.632439884	0.207514358	-3.95230424
-3.366424102	-5.372031997	-2.894436668	-2.746319781	-4.747268527	-4.038896232	-1.95728978	1.526818129	-3.787111193	-3.172596691	-4.684108103	-1.349188042	1.095174693	-3.095331625
-3.834215762	-5.569676616	-3.52877431	-2.193951146	-3.526915732	-4.349829236	-2.04666061	1.132378671	0.361384158	-3.23823794	-4.278701336	-2.671327708	-0.231762435	-3.995267461
-3.655718095	-5.592300188	-3.505788659	-2.783324272	-3.031071658	-3.61298079	-1.806953245	1.740609006	-0.787531287	-2.910085888	-4.731656712	-1.96224998	-0.141261401	-3.736833851

-3.268383514	-6.153389395	-3.751282307	-2.411529999	-3.539232738	-2.160707131	-0.917664451	1.08985098	-2.196932212	-2.111432112	-4.183054991	-1.225738944	0.291286055	-4.101568032
-3.165413241	-6.051371505	-3.652312488	-2.788537461	-3.324068172	-2.516129187	-0.878854495	1.127563538	-2.31756496	-2.130088208	-4.399865195	-1.750125885	-0.23431439	-4.057749906
-3.27201699	-5.674175417	-3.796260114	-2.778806637	-3.421535607	-2.327989037	-0.832251104	1.154474792	-1.688860773	-2.131741676	-4.233041281	-1.802302019	-0.436531036	-3.87294829
-3.368806785	-6.364082887	-3.789999419	-2.534538652	-4.021263496	-2.703294021	-1.202984129	1.272116407	-2.012828815	-2.233566206	-4.198266295	-1.853949333	0.02949561	-4.140887031
-3.708602501	-6.685677911	-3.905871133	-2.546944131	-4.063272418	-2.532124748	-1.014692529	1.095023689	-2.09607817	-2.211057065	-4.460439273	-1.714629552	-0.125321264	-4.066933956

hsa-miR-1306-5	hsa-miR-1307-3	hsa-miR-1343-3	hsa-miR-135a-3	hsa-miR-139-3p	hsa-miR-1469	hsa-miR-1470	hsa-miR-150-3p	hsa-miR-1539	hsa-miR-1587	hsa-miR-1825	hsa-miR-187-5p	hsa-miR-1908-3	hsa-miR-1909-3
-4.056659414	-3.491372943	-3.959342705	-1.684367452	-4.189151092	1.198647158	-5.549154938	-4.710081375	-5.173256445	-3.363704587	-4.484796106	-0.552706412	-6.041413316	-0.52816985
-4.045404551	-3.054637027	-3.731082274	-1.284413477	-4.099098602	2.068262013	-5.129391856	-4.923472271	-5.403809749	-3.273801919	-4.676186923	-0.062249331	-4.91835846	-0.52865509
-4.082183534	-3.082659034	-3.85001287	-1.887417381	-3.743152676	2.080234714	-5.267881036	-4.737931578	-5.354250417	-3.274200899	-4.710255645	-0.332156281	-6.000812211	-0.830709226
-4.038051325	-3.09569073	-3.988191859	-1.716377592	-4.224374889	1.241079788	-5.303438663	-4.943238803	-5.325423059	-3.270879957	-4.690296594	-0.083352669	-5.84866368	-0.915736499
-3.753011551	-3.194355986	-3.73750679	-1.524803599	-4.03461601	1.780969581	-5.311373016	-4.472135222	-4.804986157	-3.233459789	-4.338644558	-0.153430563	-5.560872722	-0.833225278
-3.973922236	-2.978576741	-3.542895055	-1.24858235	-4.1269444	1.820381359	-5.13190759	-4.643864756	-5.181814985	-3.560432998	-4.5503298	0.033855817	-4.567033367	-0.376623269
-3.862410065	-3.048871432	-3.77892883	-1.383261531	-4.099963866	1.469622298	-5.369345417	-4.693846861	-5.184194097	-3.159570086	-4.388496716	-0.066143715	-5.266518711	-0.769239618
-4.150000957	-2.891526218	-3.68639598	-1.257445317	-3.99148486	1.909476355	-4.955778904	-4.577159566	-5.333380902	-3.653881142	-4.710106341	0.410026694	-4.845320758	-0.549719217
-4.173956158	-2.949236967	-3.8578099	-1.491595871	-4.336816366	1.571777659	-5.412517761	-4.657598773	-5.151409791	-3.63268595	-4.738646364	-0.076760726	-5.496030739	-0.67746546
-4.088346122	-2.996704962	-3.419847246	-1.136050747	-4.215766965	1.753408117	-4.880964604	-4.590147893	-5.190242098	-3.609184763	-4.49551351	0.128638104	-4.541594946	-0.335905449
-4.265412343	-3.109510987	-3.764904588	-1.2936199	-4.131408118	1.951838867	-5.206747449	-4.659436181	-5.338730103	-3.530353527	-4.963485077	0.564910108	-5.306551052	-0.722161301
-4.51388596	-3.454339179	-3.932952325	-1.238300657	-4.138082575	1.557479318	-5.667317515	-5.223794311	-5.474915194	-3.30987716	-4.714590067	-1.102836955	-5.340502367	-0.657670068
-4.915329977	-3.185451077	-4.378313959	-1.60783224	-4.015327118	1.769761543	-5.676960371	-5.006007932	-5.667782845	-3.66439519	-5.088201226	-0.53452324	-5.682849495	-0.837382249
-4.313001953	-3.44844409	-3.97995658	-1.317372799	-4.114121746	1.644336187	-5.133764111	-4.934633624	-5.506084925	-3.631732299	-5.023944108	0.134703386	-5.235092482	-0.6328545
-4.408211859	-3.538865922	-3.628743283	-0.923557952	-4.111450089	1.924202662	-5.194610121	-4.709437317	-5.382612735	-3.867429487	-5.194289842	0.125872397	-4.390724882	-0.259395775
-4.294853325	-3.165517738	-3.822687733	-1.342198074	-4.205803618	1.792426367	-5.169847056	-4.897849622	-5.29246628	-3.611063853	-5.093299375	-0.267254804	-5.303082521	-0.743518003
-4.309554315	-3.255776103	-3.68646862	-1.037566571	-4.14103248	1.723192962	-5.035118689	-4.471032897	-5.231309801	-3.940909064	-5.318001977	-0.549768899	-4.1043628	-0.328905981
-4.798845954	-3.425312719	-3.747900977	-1.798510935	-4.173649258	1.977622962	-4.869721604	-5.380274601	-5.918856825	-3.847337881	-5.080599741	-0.480193099	-5.169747504	-0.898055977
-4.498561656	-3.548907805	-3.936534143	-1.388337549	-4.129354225	1.639065955	-5.331329543	-4.849387487	-5.506266993	-3.808328551	-4.968205433	-0.169436308	-5.417233588	-0.736380888
-4.299202223	-3.288371276	-3.687444437	-1.210519258	-3.950296916	1.718420501	-5.120417897	-4.663242459	-5.390713929	-3.881143917	-5.259047574	-0.029260398	-5.00188504	-0.588848751
-4.432165147	-3.343790137	-3.610082315	-0.972183999	-3.856113203	2.099320696	-5.072572465	-4.70730274	-5.341045619	-3.637240601	-5.178345759	0.167376278	-4.491083689	-0.161178103
-4.360977683	-3.094971835	-3.548083498	-0.875125989	-4.056263082	1.89087	-4.858243955	-4.598125922	-5.232144106	-3.651884889	-5.32869723	0.601941741	-4.456738621	-0.28119847
-4.254983991	-2.973028281	-3.997639122	-1.363257578	-4.099451582	1.548390298	-5.403554004	-4.557844701	-5.288559802	-3.772981325	-5.118258292	0.203846367	-5.602973868	-0.898395161
-4.046254347	-3.454476596	-3.855121029	-1.293341541	-4.173084615	1.275241194	-5.35877946	-4.3041959	-5.259694122	-3.515065463	-4.692498079	-0.523240863	-5.720136808	-0.712049764
-4.395248261	-3.37051689	-3.622230695	-0.947211862	-3.966096352	1.944079394	-4.939146801	-4.422626427	-5.430716255	-3.838156534	-5.055451276	0.354583061	-4.704877363	-0.625626701
-3.992141051	-3.411247952	-3.842241261	-1.627238969	-4.380650608	1.218390984	-5.226337354	-3.914935191	-5.138225264	-3.793419682	-4.58243308	-0.519860876	-5.949654657	-0.673263655
-4.36199725	-3.370253555	-4.049436832	-1.107213715	-4.412862763	1.373070055	-5.263083925	-4.793982581	-5.546551133	-3.882113897	-4.988930708	0.243739511	-4.993509662	-0.708080109
-4.433336977	-3.846518479	-3.895056258	-1.426046451	-4.855052716	0.887088509	-5.591086494	-4.441989786	-5.615805865	-3.56185268	-4.684381448	-0.238300419	-5.74078277	-0.705085816
-4.523599228	-3.629107096	-3.816910431	-1.843738553	-5.035623719	1.308713298	-5.389574547	-5.442077198	-5.645860996	-3.3615655	-4.32636217	-0.439721439	-5.208293942	-0.277930421
-4.290357204	-3.047345376	-3.958778009	-1.566397824	-4.237722854	1.474243254	-5.282141141	-5.032608111	-5.36465386	-3.214226239	-4.66611994	-0.015591379	-5.444296675	-0.702120088
-4.349044601	-3.115850994	-3.80144228	-1.582185275	-3.506578332	2.064328107	-5.117199531	-4.993268718	-5.297030279	-3.330063522	-4.721678825	0.184517127	-5.562207673	-0.868672204
-5.240073855	-4.90042343	-4.477734493	-2.775668198	-5.036254448	0.512931558	-6.223451321	-5.240966253	-6.20496855	-3.860366586	-5.385946206	-1.917639861	-6.011685082	-0.286493868
-4.701792307	-3.487744641	-3.495443333	-1.48893788	-4.654637792	1.749840342	-4.24982915	-4.950666774	-5.39211248	-3.448635665	-4.967158498	-1.473841942	-3.794622979	-0.51406496
-4.635496226	-3.057251294	-3.508158172	-1.478195252	-3.482054494	2.124681144	-4.424426946	-5.19845817	-5.432256983	-3.424667944	-4.992630144	-0.873693143	-4.170261471	0.084912905
-4.642490013	-3.615065938	-3.953877165	-1.399144176	-4.720213497	2.106609171	-5.224401023	-5.20793956	-5.598408786	-3.741859844	-5.325138876	-1.530980777	-5.100832903	-0.786037421
-4.208944005	-3.064091341	-3.299277365	-1.434636482	-4.470703538	2.181230116	-4.625220316	-4.928182053	-4.993132062	-3.154987921	-4.763731246	-1.274593009	-3.896562078	-0.081075933
-5.331707953	-3.648477714	-2.957505413	-0.905422153	-4.33942577	2.286523488	-3.615646282	-4.36318601	-5.093175333	-4.356602985	-6.40812922	-0.693741351	-2.810289251	-0.264237819
-4.369920845	-3.026333024	-3.30842265	-1.491326129	-4.040578617	2.536612418	-4.210846427	-4.860445471	-5.533037187	-3.944002749	-4.94818683	-0.893353811	-4.174949363	-0.317531708
-4.247477342	-2.909848022	-3.696742542	-1.580714896	-3.93928229	2.406843944	-4.946646179	-5.197457263	-5.310819844	-3.628928309	-5.020880006	-1.624200624	-5.51816657	-0.778319196
-4.726216914	-3.726360915	-2.992667603	-1.528867256	-3.949860396	2.081020954	-4.283103029	-5.10450064	-5.230782662	-4.041988408	-5.288351775	-1.552918521	-3.181267891	0.355877731
-4.365258803	-3.661226622	-3.524176936	-1.59474632	-5.010811515	2.086193476	-4.859691793	-4.855726356	-5.170874537	-3.736869554	-5.009646535	-1.110738673	-4.145569114	-0.132699324
-4.618509361	-3.248337859	-3.306950685	-1.64206039	-2.82345432	2.132297871	-4.187498264	-5.271131848	-5.499021619	-3.944209427	-5.210702547	-0.687497851	-5.033520606	-0.520806102
-4.960467035	-3.132804556	-3.696701791	-1.69291511	-3.220722952	1.632422756	-4.330535142	-3.836172868	-5.895858917	-4.307525741	-5.595753225	-0.932298855	-5.167748062	-0.824026887
-3.779133966	-4.107272886	-3.998337941	-1.717677916	-4.426299552	1.733553422	-5.081988893	-3.790708875	-4.82138216	-3.182113625	-4.3397855	-1.241221775	-5.844711633	-0.490214269
-4.627053893	-3.373192839	-3.457471556	-1.276259645	-4.158205469	2.265161365	-4.380171546	-5.171037833	-5.585535235	-4.080920876	-5.253503325	-1.285818215	-4.811839865	-0.538826184
-4.733100153	-3.324002722	-3.557799028	-1.438368582	-4.600907517	2.11362505	-4.623637226	-4.857109442	-5.692554866	-3.934611005	-5.223260897	-0.680717379	-4.68887298	-0.399950064
-4.168352632	-3.146662008	-3.781013787	-1.938219472	-4.340602761	2.148531874	-5.290745569	-5.068557584	-5.234258031	-3.312917021	-4.859006699	-0.988925489	-5.786348815	-1.04239397

-4.531805625	-3.236587354	-3.739015335	-1.532977733	-4.088622896	2.167188809	-4.732617432	-5.348725737	-5.759460624	-3.859692574	-5.181417448	-1.053577032	-5.153368341	-0.73784685
-4.460636793	-3.220405156	-2.734748094	-1.341968899	-4.146695372	2.502543664	-3.604752969	-4.888363207	-5.429505216	-3.824197842	-5.987774	-1.25554302	-3.041443067	0.085469106
-4.446336705	-3.469569109	-3.032613255	-1.126168263	-4.061310721	2.531818567	-4.525108598	-4.94764752	-5.541300997	-3.571687654	-5.320787165	-1.247334049	-3.441475327	-0.016744081
-4.054711123	-3.740335975	-3.917750693	-2.100046222	-3.882071061	1.543153089	-5.126989143	-4.162095634	-5.101095707	-3.061739121	-4.475907208	-1.487519749	-6.65888147	-0.99799697
-4.366932594	-3.208227687	-3.890129569	-1.711797167	-4.289087488	1.519859159	-5.454228977	-4.745127944	-5.238291318	-3.721959631	-4.885407884	-1.184485464	-5.737807755	-0.403647563
-4.82146008	-3.572101913	-2.147865348	-1.626415728	-3.378648661	1.950303276	-3.318478818	-5.260298041	-5.900454467	-3.841394134	-5.285043594	-1.454357784	-2.938964283	-0.388198585
-4.361515588	-3.483079972	-1.633828305	-1.034022469	-4.236452266	2.2307636	-3.033644318	-5.122232013	-5.578774157	-3.603869298	-5.105876968	-1.842916307	-2.447974451	0.381188743
-4.463877329	-3.811221689	-3.63812019	-2.119158776	-4.697962376	1.606679959	-5.209511054	-4.344097248	-5.069819627	-3.889261248	-5.056110703	-1.357935496	-4.767952737	-0.03956124
-4.632905497	-3.213409858	-3.236083741	-1.267241835	-3.186818712	2.480160165	-3.887356651	-4.871518331	-5.142947628	-3.96908941	-5.210110892	-0.421687138	-3.828731086	-0.168114841
-4.658104063	-2.863866473	-3.538155328	-1.654799471	-3.110193986	2.06432875	-4.633273065	-5.173866789	-5.554913459	-3.878503318	-5.267010528	-0.644785809	-4.925675671	-0.418466008
-5.214455302	-3.038985874	-3.605052541	-1.326163054	-2.625337982	1.509114478	-4.309635163	-4.28827091	-5.340712125	-4.471697915	-5.73416367	0.236832201	-4.623208793	-0.191794332
-4.496655267	-2.971184489	-3.559489599	-1.43913166	-2.946549452	1.943786189	-4.802184235	-4.710456184	-5.424926501	-3.764205669	-5.123677836	0.316912755	-5.101330848	-0.583329854
-4.810889423	-3.912387046	-3.829897322	-1.862427229	-4.78824184	1.49354438	-5.200112942	-5.253741942	-5.748917581	-3.979348808	-5.3154173	-1.238781336	-5.258647571	-0.251023895
-5.000214553	-3.708225951	-3.144230042	-1.898590601	-2.897336657	1.907638496	-4.109057538	-5.073811921	-5.768044593	-4.200901568	-5.545635889	-0.440675821	-5.059034182	-0.596554711
-5.778751526	-4.094086438	-2.628611855	-1.670296806	-3.420255126	1.822445273	-3.779582389	-5.414323785	-6.307174415	-4.093975615	-6.234679407	-1.048702103	-3.994814038	-0.335911275
-5.150228293	-3.623961067	-2.58920263	-1.23913651	-4.813044175	2.107541552	-4.076605823	-5.401416727	-5.78069134	-3.791401403	-6.771293144	-0.305131676	-3.737843925	0.070281604
-4.660291965	-3.164618372	-3.375073242	-1.594794379	-4.60481303	2.472509133	-3.970613458	-4.713964079	-5.544982495	-3.967191392	-5.072551721	-0.724110715	-4.077625968	-0.610847098
-4.210579836	-3.634124287	-3.701440938	-2.216891649	-4.515714016	1.693461522	-5.095771599	-5.164531849	-5.065292039	-3.553768125	-4.264228026	-1.525798746	-5.120561597	-0.453535955
-4.196590117	-3.176141086	-3.671604289	-1.595263766	-4.091284613	2.177829073	-5.370453031	-5.315534072	-5.231003226	-3.423133636	-4.777269499	-1.34222364	-5.565258579	-0.625416948
-4.771048409	-3.021378552	-3.650524744	-1.431290639	-3.0059991	1.573275194	-4.358603918	-5.436271139	-5.848197407	-4.051463295	-5.609117078	-0.46808862	-4.807798415	-0.75429325
-4.344077591	-3.108304118	-3.554611689	-1.452399849	-3.763624905	1.933580681	-5.114934275	-4.720213669	-5.210074241	-3.470868208	-4.979503303	-0.619765603	-4.605107754	-0.305032512
-4.807313917	-3.377102922	-2.502124343	-1.47308439	-4.851929636	0.712482888	-4.839999958	-4.101885534	-5.418559847	-3.048840914	-5.227130498	0.024028402	-4.844446709	-0.743663921
-5.114270109	-3.673043893	-4.254032284	-2.001307452	-4.05621752	1.359066637	-6.222204462	-5.609853529	-5.991696579	-3.700835951	-5.147960201	-1.377694993	-6.263907862	-0.979026678
-5.616396488	-3.347687209	-3.440744377	-1.037531498	-4.586061465	1.624143955	-4.770505252	-5.700685817	-7.271817547	-3.562315589	-5.768318293	-0.474986926	-3.564833723	-0.337572937
-4.664920053	-3.48039066	-3.940805037	-1.464294469	-5.017739636	1.584858607	-5.52939027	-5.532376268	-5.768806107	-3.802630969	-5.158117687	-1.04067833	-4.548432169	-0.62255465
-4.726069661	-3.391630811	-2.05367731	-1.362014939	-5.18004524	1.304510553	-3.705033174	-3.696854488	-5.462257376	-3.892397885	-6.033039867	-0.447188728	-3.736613529	-0.474536476
-4.628275294	-3.4976462	-1.598315672	-1.667106523	-5.034808425	0.898671649	-4.849011917	-3.776098888	-5.254772053	-3.07110302	-4.963044687	-0.921551479	-3.848229366	-0.173237081
-4.524754047	-3.558729179	-2.119644256	-1.260992326	-5.159647468	0.749551848	-5.03671013	-3.653746872	-5.334984247	-3.111364768	-5.086285647	-1.055147374	-3.574917534	-0.00226291
-4.612983945	-3.506215593	-2.460892353	-1.441805937	-4.957312003	0.828544777	-4.919911817	-4.064623285	-5.468796866	-3.086996206	-5.066055818	-0.36275598	-4.421860507	-0.5271324
-4.453850885	-3.364990462	-2.416112803	-1.622669781	-4.090929721	1.391889972	-4.384593483	-3.942422024	-5.270341897	-3.640319609	-5.35437857	0.237993354	-4.41358583	-0.747966781
-4.73477777	-3.486198351	-1.798761371	-1.624788618	-4.72618831	0.859057205	-4.324725163	-3.962795627	-5.727455157	-2.935079729	-5.614265983	-0.714135893	-4.070084781	-0.777084572
-4.808502413	-3.752409582	-1.646938934	-1.656598422	-4.573450821	1.079595564	-4.227281725	-3.964349737	-5.649452844	-3.188595532	-5.458482469	-1.338956194	-3.338193854	-0.065058139
-4.460707594	-3.425715078	-1.90451643	-1.631572334	-4.51332298	0.679368358	-4.361020751	-4.136132487	-5.436974756	-2.981131986	-5.123676121	0.348975005	-4.454679107	-0.663989
-4.480474546	-3.189626319	-1.783128555	-1.545590038	-4.495430624	0.86089314	-4.635797374	-3.676985889	-5.480023553	-2.827915045	-5.166523969	-0.626536672	-4.392751816	-0.50189169
-4.622262166	-3.390559643	-3.659964996	-1.552758228	-3.558206785	2.019570461	-4.882076418	-5.14987999	-5.785145821	-3.905381998	-5.123788955	-1.2233434	-5.093866233	-0.602111676
-4.42721042	-3.110763471	-3.400150442	-1.413597361	-3.694479502	2.551050826	-4.265195344	-4.922062693	-5.186173697	-3.7287881	-4.913387143	-0.066655001	-4.345097791	-0.150733893
-4.723075191	-3.116938809	-2.960971669	-1.337582103	-2.699075738	2.048188235	-4.01699501	-5.156982598	-5.519265745	-4.085761307	-5.342104515	-0.23015518	-4.106385601	-0.406231416
-4.470231774	-3.247546319	-2.852681261	-1.437157635	-3.904200593	2.423148185	-3.653699394	-5.203344491	-5.498756994	-4.09868274	-5.456215937	-0.345248949	-3.880929656	-0.653961167
-4.480993188	-3.42637544	-3.298889242	-1.434471696	-4.362897729	2.36567679	-4.355935389	-5.093760947	-5.441435535	-4.225776834	-5.251877355	-1.44797981	-4.202372218	-0.504636071
-4.709021746	-3.446610743	-3.083234192	-1.85722918	-5.626597393	2.584754779	-4.271410243	-4.927959107	-5.484047508	-4.113352621	-4.749995957	-2.046856779	-3.390602532	0.105785994
-4.597801111	-2.806569318	-3.526855353	-1.249862682	-2.833157234	2.210985941	-4.054843943	-4.430220332	-5.28015079	-4.112336564	-5.35284211	0.305659061	-4.285551734	-0.425779904
-4.70917216	-3.160443182	-3.942445381	-1.589357081	-2.580810074	1.878339025	-5.087965703	-4.926174582	-5.633904016	-3.995243909	-5.187097005	0.013170515	-5.722419509	-0.782641637
-4.885931771	-2.936185049	-3.73482291	-1.57279839	-2.777623796	1.473955326	-4.679733424	-4.885152777	-5.589165117	-3.772704288	-5.792288958	-0.224595467	-5.604801281	-0.66857691
-4.966862642	-3.829075051	-3.648444424	-2.270344942	-4.278862031	0.976099168	-5.012737258	-5.233847245	-5.878371753	-4.302571292	-5.813599144	-1.691910623	-4.98142616	0.107446157
-5.409189256	-3.784506362	-3.827983921	-1.820856536	-3.914039182	1.456892587	-5.315307569	-5.33393987	-6.220604531	-3.909768507	-5.719923674	-1.029338776	-4.764482507	-0.211708467
-4.976955557	-3.290905942	-3.165889318	-1.418917771	-3.512295885	1.806575973	-4.151129845	-4.938261226	-5.923476453	-4.080919153	-5.828626212	0.169263282	-4.460690433	-0.550171421
-4.8626802	-3.46119832	-2.605134048	-1.402177556	-4.3164086	2.533920244	-3.564980103	-4.070749583	-5.33788207	-3.614863675	-5.235307336	-0.364453872	-3.4278695	0.191310875
-4.838442044	-3.368861478	-3.831449169	-2.303899157	-3.234592019	1.902734268	-4.624226069	-5.552527747	-5.78684554	-3.929094556	-4.998431238	-0.275051766	-5.286739026	-1.004221393
-4.684219804	-3.215624218	-2.84198661	-1.433345266	-3.064930613	2.150453356	-4.13963904	-4.725010736	-5.504939525	-3.990180386	-5.17579925	0.351488334	-3.407541275	-0.470580288

-4.511666055	-3.523437262	-1.535676951	-1.430457155	-4.818757685	0.949265493	-4.495494421	-3.811536462	-5.431856283	-2.774161119	-4.948356553	-0.448394054	-3.873429473	-0.271366803
-4.307705038	-3.381292971	-1.493212449	-1.706226484	-4.660575937	0.824359572	-4.227950058	-3.792645162	-5.432108326	-3.090127298	-4.926902727	-0.024036566	-3.93991457	-0.622902191
-4.370819001	-3.0827365	-1.778545821	-1.63620552	-4.307985132	0.944921256	-4.231613448	-3.754495035	-5.652181504	-3.053082646	-5.360415848	0.100676912	-4.011748306	-0.627961548
-4.450072228	-3.27201614	-2.122724929	-1.711331845	-4.781013041	0.921662922	-4.664278799	-3.976880134	-5.542320568	-3.077932432	-5.035594767	-0.269980415	-3.926011468	-0.583074814
-4.583363311	-3.478276722	-2.683338909	-1.69463413	-4.86226892	0.788256085	-5.185575828	-3.981348646	-5.609865829	-3.268914846	-5.070262794	-0.028732168	-4.980323181	-0.721296645

hsa-miR-1910-5	hsa-miR-1913	hsa-miR-1914-3	hsa-miR-1914-5	hsa-miR-1915-3	hsa-miR-1915-5	hsa-miR-191-5p	hsa-miR-193b-5	hsa-miR-197-3p	hsa-miR-197-5p	hsa-miR-1976	hsa-miR-204-3p	hsa-miR-208a-5	hsa-miR-210-5p
-4.849267566	-2.937796699	-1.656863644	-5.866581207	0.974517251	-2.037955697	-2.733139775	-5.993993175	-3.776806697	-1.767531141	-3.682394972	0.400673693	-5.458433852	-3.6704439
-5.039201424	-3.290402712	-1.950370511	-5.971820345	1.251441071	-4.113202197	-2.152987565	-5.466276709	-3.362686469	-1.432350979	-3.886082317	0.051722162	-4.613167311	-3.797528313
-4.971135711	-2.988534285	-2.008047528	-5.926649889	1.164613962	-3.084186793	-2.673460134	-5.203651732	-3.691991024	-1.609122577	-3.760492164	0.634272009	-4.816993228	-3.778831266
-5.200307708	-3.078443285	-2.005437366	-5.971586991	1.105404432	-3.566467371	-2.258864063	-4.683257697	-3.685177144	-1.443325052	-3.787803117	0.93113617	-4.310964629	-3.795851537
-4.804811277	-2.570161473	-1.712950605	-5.761310846	1.127647614	-2.33753602	-2.368605651	-5.291658847	-3.715268915	-1.549421437	-3.403172305	0.479302699	-4.940347761	-3.341819001
-4.786264589	-2.917876659	-2.010327343	-5.408925095	1.18743111	-4.191031951	-2.194236852	-5.364993882	-3.537371294	-1.318322158	-3.653079397	0.443081674	-4.34063529	-3.629765975
-4.842350081	-2.789037125	-1.898638094	-5.695117076	1.148525483	-3.43392234	-1.623589496	-4.805438041	-3.717654101	-1.49695809	-3.456873209	0.329825736	-4.568447125	-3.491078655
-4.896184251	-3.012796867	-2.160252772	-5.843538908	1.237724911	-4.822522196	-2.11995787	-5.023626835	-3.490924266	-1.435963817	-3.835317715	0.808760219	-4.000229397	-3.791772526
-4.905778056	-3.213165686	-1.993211741	-5.937025578	1.27777929	-3.978548737	-2.642862266	-5.483979719	-3.736963117	-1.494166839	-3.867204053	0.904383972	-4.505544244	-3.782097548
-4.754398497	-3.192104304	-1.994823061	-5.710989608	1.13350227	-4.669217638	-1.630473616	-5.429628437	-3.746191338	-1.471723327	-3.579992335	0.183156679	-4.392053373	-3.794724444
-5.118861919	-3.01762196	-2.100032985	-6.029633923	1.269681336	-4.833907989	-1.762255734	-4.850946396	-3.824380624	-1.538612176	-4.056238936	0.608245105	-3.849423517	-4.108898882
-5.371996532	-3.638264376	-1.966959087	-6.289490539	0.909652884	-3.070135227	-3.720092223	-5.636689555	-4.407468662	-1.543673203	-4.254142241	0.027509961	-5.58893124	-4.428997641
-5.374635392	-3.607190581	-2.033018102	-6.365794049	0.902923932	-4.027018547	-3.371619163	-5.201689151	-4.420311512	-1.556290293	-4.331336615	0.613051289	-5.350191394	-4.3972996
-5.108942328	-3.218160776	-1.821120749	-5.928394124	1.098304083	-4.057493907	-2.232399322	-4.915977217	-4.269463897	-1.947653122	-4.206747449	0.272420837	-4.744383647	-4.084459164
-5.343318216	-3.324492333	-2.071671425	-5.85803089	1.250572276	-4.164152477	-1.878291882	-5.01636119	-4.244219173	-1.764352904	-4.340046207	0.103116963	-4.637870865	-4.193570867
-5.044950033	-3.397835592	-2.104891392	-5.955673005	1.483684526	-4.451123405	-2.780512198	-4.836912022	-4.420296628	-1.455590124	-4.269746029	-0.165039719	-4.694783475	-4.201965622
-5.167842874	-3.333799395	-2.053437992	-5.814455268	1.299633686	-3.932576318	-2.953532984	-5.017634103	-4.616270149	-1.838423309	-4.365771586	0.46757884	-4.514628486	-4.128647572
-5.181546854	-3.362868934	-1.65249953	-5.750781504	1.034545313	-4.701254157	-1.741684766	-4.859987147	-4.033073066	-1.946678945	-4.307757015	-0.335408054	-4.9252553	-4.454308145
-5.327035797	-3.19599223	-1.644565839	-6.063993598	1.06200787	-3.778215033	-1.645966293	-4.86269501	-4.005873661	-1.702475296	-4.195054831	0.406863296	-5.005692755	-4.088889979
-5.023422647	-3.25025345	-1.913583553	-6.025909529	1.223464728	-4.252747519	-1.925997739	-4.816510207	-4.222628325	-1.659396243	-4.264379264	0.672668032	-4.545752432	-4.208688558
-5.32215467	-3.292296562	-1.997517091	-5.859284803	1.089400379	-3.769698694	-2.254858925	-5.32058361	-3.983431541	-1.773072819	-4.389829092	0.000705828	-4.405196314	-4.137603666
-5.274170265	-3.318149734	-2.03815794	-5.815097186	1.293633629	-3.41811343	-2.708746945	-5.041656703	-4.446401951	-1.884578183	-4.410353972	0.333037685	-4.628431718	-4.075431609
-5.158405235	-3.153757506	-1.838465934	-6.07321529	1.12996091	-3.961071052	-2.033348008	-5.562674057	-3.959747708	-1.574910176	-4.311367531	1.040961858	-4.496844471	-4.065748194
-5.067054824	-2.965163804	-1.502441814	-5.903763948	1.07984826	-2.412921349	-3.077899746	-5.051598597	-4.21819349	-1.568821652	-4.055239697	1.145387128	-4.532855108	-3.800192797
-5.385849015	-3.221441453	-2.115751082	-6.112357779	1.182586876	-4.27810545	-2.49663055	-4.420325179	-4.181096521	-1.985294922	-4.244690738	0.696857568	-4.522381182	-4.152077086
-4.872025969	-2.699885701	-1.615742994	-5.780367697	1.012815172	-1.700661336	-2.646393046	-5.074485658	-4.364566793	-1.533989379	-3.947995569	2.254310829	-4.788242115	-3.72287176
-5.327020918	-3.524096118	-2.246610702	-6.487679743	0.883066282	-4.759959294	-2.9780811	-4.879575047	-4.023737126	-1.464873833	-4.383992765	0.824336827	-4.57690694	-4.327886515
-5.317913275	-3.234129469	-1.756250919	-6.18091116	0.702358122	-2.720326578	-3.204194285	-5.415942816	-4.480268197	-1.706757358	-4.113243906	1.223164179	-5.006809812	-4.078016075
-5.06121181	-3.182615283	-2.00346512	-5.922123202	0.879771737	-3.16253437	-2.553463024	-6.06819712	-3.89732941	-1.515032241	-3.756506855	0.456813353	-5.043813963	-4.108778352
-5.064729441	-3.248950233	-1.984874965	-6.022077536	1.194574191	-4.136005199	-2.428585269	-4.758632609	-3.71669476	-1.451421886	-3.931913314	0.723374238	-4.36786596	-3.992173186
-5.191461574	-3.397973613	-1.995459087	-6.067480493	1.056251277	-4.666533552	-1.650598349	-4.594222399	-3.849266642	-1.344046444	-4.059558893	0.08893808	-4.241880056	-4.072463776
-6.22775797	-4.125680585	-2.186839567	-6.871048409	0.046851427	-3.538018271	-4.129768384	-4.853055437	-4.650309935	-2.361367692	-4.865289009	-0.843983683	-2.534966351	-4.921505019
-5.378293029	-3.856372464	-2.518950977	-5.993010895	1.113544943	-5.314832815	-2.484217673	-4.159996054	-4.551271601	-1.555602594	-4.40712515	-1.499570189	-4.011776794	-4.327510441
-4.816168243	-3.713396171	-2.379543328	-6.018836001	1.516248131	-5.463330348	-3.221419627	-4.591879988	-4.663614574	-1.15645567	-4.252163409	-2.015536678	-3.979885274	-4.176670999
-5.177898621	-3.617795825	-2.216818913	-5.778525297	1.350126046	-4.741338991	-3.801330534	-4.769859773	-5.31584665	-2.030821848	-4.475221814	-2.041579678	-4.830893111	-4.333893037
-4.814292037	-3.292404724	-2.371866963	-5.583048146	1.901627795	-3.379493627	-2.780186521	-5.036294489	-4.727537786	-1.789231871	-4.045990845	-1.17905853	-4.340962106	-3.716463521
-5.845164844	-3.764663713	-2.04536572	-4.781316884	1.877355747	-4.79924241	-2.380297311	-4.234345182	-5.433328529	-1.984284721	-5.238465293	-1.971650436	-3.869500318	-4.929877865
-4.466524192	-3.178598712	-2.061205475	-5.42744532	1.61806967	-4.564997875	-3.091130217	-5.217677539	-4.9249539	-2.069493831	-4.14451807	-1.929625509	-4.071639741	-4.036688134
-4.732303837	-3.049432643	-2.212945164	-5.347927498	1.559527934	-4.467679555	-2.609484369	-4.724026903	-4.889223742	-1.855743994	-4.113538085	-1.771498092	-4.724558187	-4.073843105
-4.909818684	-3.182231106	-2.339108198	-4.991123433	1.316373395	-4.353947457	-3.286872346	-5.953182162	-5.41770551	-2.221227217	-4.695458162	-2.350325146	-4.673961309	-4.17760051
-4.677616375	-3.240864493	-2.03893849	-5.214274916	1.470094155	-2.655230013	-3.456866588	-5.228554516	-4.944226638	-2.376678604	-4.033185784	-0.857518169	-4.881669758	-3.973109889
-5.034247241	-3.43406665	-2.459176939	-5.497925445	1.262592102	-5.873893649	-1.320038635	-3.382948563	-4.926831098	-2.070063733	-4.190441449	0.086260885	-4.099694914	-4.335667396
-5.229983817	-3.633330851	-2.331422738	-6.001851355	1.326344519	-6.127553221	-2.65184814	-3.773220216	-5.140499711	-2.324405295	-4.815262055	-0.854673438	-5.151256737	-4.604094465
-4.657462224	-2.002255447	-1.08929253	-4.890926788	1.228045686	-1.35301807	-4.386577185	-6.795482933	-4.530940903	-2.159910198	-3.762691715	-0.791799392	-4.769963475	-3.475279622
-4.952242295	-3.529968074	-2.585320205	-5.680721128	1.647166312	-6.18352322	-1.894006898	-4.470337409	-5.162813221	-1.741402859	-4.534783928	-1.993844383	-4.646576301	-4.124282658
-4.965808624	-3.460400254	-2.418457151	-5.757082661	1.714467423	-4.689051481	-3.215183509	-4.946625017	-5.257776915	-1.969335213	-4.469825033	-1.860702592	-4.191829784	-4.168619113
-4.76896581	-3.048878614	-1.887073888	-5.564728101	1.958809047	-2.877939216	-2.897974808	-4.984490321	-4.654071487	-1.689036329	-4.170645465	-2.060205265	-4.576254824	-3.960863453

-5.0170686	-3.574118157	-2.284504302	-5.925517954	1.681692518	-5.680189035	-2.499865707	-5.019039988	-4.659565536	-1.558068545	-4.582151249	-1.059260458	-4.034555352	-4.466787783
-4.631520253	-3.55686289	-2.434782852	-5.166605818	1.749454904	-4.90782244	-2.868630597	-4.523983273	-4.998009046	-1.885240904	-4.574031216	-1.466063233	-4.178996034	-4.137843727
-4.621461823	-3.313030578	-2.075319389	-5.479634269	1.724707891	-4.592261743	-2.99519945	-5.202141784	-4.768579958	-1.738847769	-4.310703082	-2.076207879	-3.474227451	-4.072863232
-5.004865923	-2.305549136	-1.106496086	-5.394740466	1.195894633	-1.707523847	-4.095196055	-6.093796023	-4.610701712	-1.735716601	-3.826281258	0.29216354	-4.676406061	-3.833971405
-4.881211094	-2.736725351	-1.676019202	-5.605818784	1.34500934	-2.035298925	-4.132443079	-4.493840961	-4.853412481	-2.234596158	-4.121375994	0.118927356	-4.407884729	-4.013996343
-5.246591847	-3.823176788	-2.125099066	-5.505929006	1.158341562	-5.369625961	-2.664538662	-4.423232744	-5.195714937	-1.66152078	-4.765126781	-2.05802167	-4.424113853	-4.005000492
-5.427681338	-3.601273344	-1.916737108	-4.33120962	1.478391404	-4.843522675	-3.869983016	-6.589967113	-5.227121785	-1.629061378	-4.250285179	-1.491163373	-4.976026175	-3.808260392
-4.845462188	-2.831730325	-2.049693018	-5.316775978	1.64992409	-3.365545157	-2.588393034	-4.991143163	-4.623206032	-2.197517427	-4.285075639	-2.549648593	-1.973374301	-4.009575658
-5.113768766	-3.53055937	-2.292763001	-5.478412277	1.811917678	-5.393642386	-2.539956608	-4.209930761	-4.7333558	-1.480561746	-4.699975691	-0.822074763	-4.115253459	-4.22911187
-5.012681294	-3.561377144	-2.099395086	-6.133747514	1.627340208	-5.633281299	-2.988159224	-4.053786954	-5.049488419	-1.644041743	-4.450890272	-0.937255802	-4.144720569	-4.324845102
-4.976457488	-3.599408055	-1.871420298	-5.488528755	0.910447814	-5.938151014	-2.327548894	-2.611982559	-4.963547551	-2.382835793	-4.623569016	1.329999264	-4.902198983	-4.270178362
-4.921746123	-3.46293495	-2.132866838	-5.593329845	1.480542543	-5.34296088	-2.462109898	-3.069982818	-4.50059082	-1.792516825	-4.3880733	0.366820685	-3.490844682	-4.414763406
-5.271544375	-3.331691642	-1.881908438	-5.75492311	1.083742405	-1.874496656	-5.222777878	-5.633153139	-5.294330785	-2.579449011	-4.520280925	-0.723267266	-5.180053114	-4.412273607
-5.198083297	-3.666621474	-2.291074052	-5.50515573	1.045068507	-5.230476347	-3.188169519	-3.631540039	-5.258921966	-2.187865517	-4.6857263	-1.030475229	-4.583543492	-4.574245423
-5.429866809	-4.227279336	-2.08153178	-4.854990263	0.767725251	-5.065505894	-3.452101065	-4.606079264	-6.05925406	-2.326693623	-5.542986215	-1.344215551	-5.6463493	-5.251806682
-5.397273549	-3.913935596	-2.131818579	-5.449116252	1.448936675	-4.611923944	-2.424781737	-5.5118395	-5.331696184	-2.027740372	-4.551418411	-1.852985283	-4.417345428	-4.362016443
-4.712821966	-3.358073238	-2.470732952	-5.16132705	1.466876452	-4.706581564	-1.339089508	-5.504713346	-4.626024025	-2.095462609	-4.208685886	-0.058146298	-3.690505272	-4.135228772
-4.582012405	-2.722539715	-1.835275818	-5.451670268	1.080696799	-1.869559631	-4.771035865	-6.093257183	-4.228361225	-1.96925194	-3.570998945	-2.299898161	-3.994898485	-3.693721257
-4.974704657	-3.239078321	-1.696593097	-5.942963316	1.931635242	-4.520785191	-4.192504769	-4.832522916	-4.728662509	-1.48315284	-4.237842477	-1.857118412	-4.048140461	-3.995698853
-5.392739376	-3.85750311	-2.484837115	-5.916388243	0.867982812	-6.361480913	-1.522602997	-3.463670868	-4.854657755	-2.029854329	-4.827320048	-0.886655073	-3.829593465	-4.430926095
-4.912105272	-3.230187536	-1.890169853	-5.744516416	1.405042289	-3.520935331	-2.928042372	-4.774293192	-4.803806483	-1.86222733	-4.122812124	0.616936949	-3.850621484	-4.009144803
-5.445703792	-4.059994046	-1.997645334	-6.739036029	0.853308801	-6.00081973	-3.19069842	-3.793589606	-4.184880956	-1.282321112	-4.476187954	1.901066577	-5.593832554	-4.567309946
-5.782334974	-4.089350823	-2.129423473	-6.694251064	0.837074099	-4.629310579	-2.641888911	-4.091579262	-5.101136119	-1.645828409	-4.653952145	-0.957890291	-5.249552393	-4.904272006
-6.509618687	-4.680103949	-2.388765401	-8.166994337	1.126268869	-6.312511464	-3.002061501	-4.575586187	-5.195887181	-1.492837296	-5.029682776	-1.484825156	-4.443652046	-5.190195449
-5.654662423	-3.786522177	-2.026558571	-7.201864003	0.734867844	-4.996103849	-2.430842575	-4.031941113	-4.711766813	-1.593644277	-4.508026522	-1.354042957	-4.725223218	-4.58888141
-5.085811252	-3.622373924	-2.121194063	-5.555628029	1.156361305	-5.183465479	-3.582437929	-4.431492632	-4.801363429	-1.604486149	-4.561011514	2.441076906	-6.295878672	-4.505090266
-5.581938637	-4.052765351	-1.959284666	-6.360830933	0.802371113	-4.624127556	-4.746370396	-4.514164381	-4.552808978	-1.481978401	-4.457159132	2.276856038	-6.317559481	-4.527251902
-5.295355453	-3.955051393	-1.921156504	-5.991930675	1.1936927	-3.56676898	-5.568950134	-5.171068927	-4.664424527	-1.727973465	-4.426555936	2.680982784	-5.934250765	-4.166867559
-5.50599315	-3.909706679	-2.121832688	-6.657178954	0.753151493	-5.31794523	-3.457642257	-4.161386912	-4.479493739	-1.423615218	-4.421612878	2.131080705	-5.710139131	-4.285083694
-5.287056534	-3.628767592	-2.332357015	-6.239864573	1.027190726	-5.568030875	-2.510832174	-4.395120935	-4.136939938	-1.43918372	-4.438852177	2.569985806	-5.084281782	-4.020536514
-5.319418705	-4.208602011	-2.041310826	-6.462910976	0.537092049	-5.74539364	-4.295215918	-3.827915257	-4.742723704	-1.356639871	-4.631547562	1.952486601	-6.48174938	-4.574742048
-5.421803635	-3.802378255	-1.88887463	-6.365447147	0.589832445	-4.348524962	-3.713554431	-5.056795175	-4.67293387	-1.682169946	-4.544268634	2.365356026	-6.186157283	-4.284770744
-5.735007546	-3.947823266	-1.99884743	-6.331444739	0.694905801	-5.982585699	-2.612001882	-3.779615782	-4.125352049	-1.294926193	-4.515237444	1.369201793	-6.341435594	-4.256082983
-5.541819829	-3.986495569	-1.637503107	-6.689496564	0.557477101	-5.891479595	-3.514541037	-4.164616461	-4.409253518	-1.322555415	-4.52657176	1.865589999	-6.273258406	-4.288388205
-5.087616354	-3.516370336	-2.372963237	-6.018941787	0.978705053	-5.006462808	-3.685628421	-4.713073299	-5.080606222	-2.191712271	-4.135473515	-2.2274782	-3.997540369	-4.372406147
-4.728680375	-3.126512346	-1.825730041	-5.251770512	1.676726476	-4.35589584	-3.028024138	-5.171777394	-5.035778889	-1.743745665	-4.055681137	-2.164285486	-3.251959744	-4.149314459
-5.110506271	-3.575822217	-2.123768904	-5.355461754	1.111442309	-5.304247997	-1.94064648	-3.465924554	-4.743753894	-2.02281439	-4.501273605	-0.450380604	-3.603185049	-4.096147569
-4.690667277	-3.224413831	-2.199359203	-4.946357577	1.635006603	-5.4054712	-1.861698696	-4.585014771	-4.822793835	-1.847998038	-4.327456784	-0.993778254	-3.699404298	-4.297946659
-4.887268455	-3.389260721	-1.895344875	-4.956734437	1.806019987	-4.810529169	-3.151863323	-4.938517482	-5.583752166	-1.879355716	-4.544598716	-2.115762075	-3.625541021	-4.11213084
-4.481489475	-3.083901143	-2.12864098	-4.981131336	1.980834939	-3.794693674	-4.105802494	-5.504875183	-4.940915101	-2.078926915	-4.377367319	-2.241756834	-3.176269284	-3.939030006
-4.661690848	-3.500495121	-2.368643315	-5.323301803	1.495184444	-5.589475394	-1.705027596	-3.624226407	-4.533927471	-1.840196471	-4.372196273	0.191007461	-2.910572397	-4.272038092
-5.213909754	-3.778640815	-2.124845668	-6.107969777	1.349652574	-5.760501731	-2.102266603	-3.201506553	-4.932764925	-1.893510909	-4.641990743	0.539195141	-3.245140708	-4.500971076
-5.138924881	-3.681467789	-1.621489397	-5.948170599	1.176620249	-5.837455683	-2.803431729	-3.102581298	-5.217314939	-1.946126278	-4.657833155	0.490059428	-5.49013222	-4.581324043
-5.588544427	-3.652211467	-2.253535355	-5.801296986	0.950692355	-4.25202022	-3.736489621	-4.283161035	-5.457956078	-2.568438783	-4.861358311	-1.136799002	-2.528235435	-4.626605786
-5.56997236	-4.009268499	-1.952888487	-6.047153487	0.955133894	-2.789765624	-5.18485347	-4.713978765	-5.537334564	-2.833488314	-4.950590725	-0.536193512	-4.797490739	-4.838872109
-5.170119103	-3.689214794	-2.058295033	-5.731041249	1.24672521	-5.258958638	-2.585626824	-4.106004482	-5.162770642	-2.223217851	-4.642099334	0.210439224	-3.943139162	-4.584756475
-4.704677249	-3.532874502	-2.291094483	-5.391035704	1.650558505	-4.621886294	-2.74024993	-5.160449067	-5.027840784	-1.915106636	-4.988612285	-2.184384686	-4.758230277	-4.477457405
-4.947282769	-3.412520424	-2.396269208	-5.684146865	1.214913949	-5.255348652	-2.788920945	-4.177716575	-4.899610875	-2.10452005	-4.199964664	-0.805650334	-2.972201632	-4.398242231
-5.279077538	-3.737034817	-2.466537602	-6.045436892	1.465967594	-5.869028822	-1.476281686	-3.354867659	-4.750146205	-1.977042206	-4.501252532	-0.452894845	-3.265395805	-4.133939368

-5.182263758	-3.812464454	-1.971692481	-5.9963141	0.972110421	-5.198003675	-3.321267942	-4.222805467	-4.271354883	-1.157387944	-4.278064009	2.410844618	-5.731588629	-4.161391995
-4.908592894	-3.757022261	-1.886283009	-6.745177118	0.686754361	-6.181111474	-3.726043267	-3.792913001	-4.155322219	-1.068630258	-4.201000617	2.125539989	-5.486463967	-3.949308806
-5.40626771	-3.968295641	-2.018300505	-6.098150537	0.954725667	-6.032164806	-3.161598121	-3.42500719	-4.195655242	-1.065252888	-4.356163166	2.355036734	-5.467000115	-4.13122047
-5.435855331	-3.856373906	-2.117380921	-6.405749078	0.878858149	-5.520691256	-3.434146048	-4.167409443	-4.272597406	-1.200275432	-4.345476353	2.317355547	-5.437703195	-4.380961235
-5.592446211	-4.034235272	-2.181483135	-6.475210567	0.662685815	-5.928435286	-2.921222275	-4.009947603	-4.191139355	-1.385601584	-4.365373853	2.483616281	-5.812212273	-4.52907725

hsa-miR-2110	hsa-miR-211-3p	hsa-miR-2116-3	hsa-miR-221-3p	hsa-miR-223-3p	hsa-miR-22-3p	hsa-miR-2392	hsa-miR-23a-3p	hsa-miR-23a-5p	hsa-miR-23b-3p	hsa-miR-24-3p	hsa-miR-2467-3	hsa-miR-2682-3	hsa-miR-296-3p
-4.337595182	-2.90562921	-4.119070547	-1.194757815	-0.620832577	-0.747537349	-2.596329419	-1.228991285	-5.916417192	-1.446763733	-2.228339688	-4.365472001	-5.429481168	-5.551625191
-4.03421068	-2.939501608	-4.540593485	-0.404035203	0.163659762	-0.168673376	-2.935102492	-0.277010622	-5.594912924	-0.524545482	-1.369067795	-3.648485201	-5.526075773	-5.378963891
-4.12434734	-3.534535355	-4.02346188	-0.900005003	0.135329168	-0.780585211	-2.644068306	-0.57862404	-5.449124177	-0.907039973	-1.467620717	-3.998471754	-5.328027892	-5.709657312
-4.281452699	-3.489701967	-4.272819015	-0.911158351	-0.808219304	-0.319397581	-2.638545119	-0.999690892	-5.159078259	-1.785523221	-1.774829785	-4.004684134	-5.289571656	-5.475227603
-4.081127083	-3.474141782	-3.707213262	-0.978016836	-0.82293445	-0.750863371	-2.598172457	-1.109986501	-5.471028421	-2.033478359	-2.099826507	-3.958016345	-5.225314417	-5.582540036
-4.084304384	-2.518498306	-4.083421668	-0.071960787	-0.497502081	-0.37630222	-2.843280633	-0.905818551	-5.535000351	-1.633447591	-2.041458567	-4.045467176	-5.340458927	-5.142551973
-3.759089885	-3.406400067	-3.930002925	-0.664532019	0.462185023	-0.24770483	-2.889356933	-0.146200553	-5.189445742	-0.444698703	-1.339345643	-4.008517284	-5.212630809	-5.485915624
-4.004197727	-3.067399315	-4.436064014	-0.330706731	-0.196983882	-0.629176145	-3.134440204	-0.610863146	-5.420225317	-1.128802529	-1.757085148	-3.826782798	-5.409083305	-5.383361013
-4.180960801	-3.145912171	-4.359419343	-0.933903749	-0.532538118	-0.818701072	-3.115229273	-0.968560697	-5.52418254	-1.542405803	-2.07313911	-3.983779077	-5.457331358	-5.626839875
-3.715442483	-2.484782216	-4.271970411	-1.006723072	-1.499626854	-0.444376894	-3.293685721	-1.352774116	-5.405878469	-2.447786008	-2.481431289	-4.090294145	-5.447043319	-5.043313028
-4.251023784	-3.328558414	-4.283874882	-0.185509441	0.467477947	-0.079765747	-3.277413601	-0.024505951	-5.283355663	-0.252072893	-1.161684529	-4.32390557	-5.535764876	-5.140088522
-4.316010736	-3.083818834	-4.745383879	-2.682171284	-2.230670552	-2.695974896	-3.040454149	-2.851434278	-6.100259042	-3.906480482	-3.789648325	-4.45318097	-5.720359191	-5.918639495
-4.543609373	-3.25533438	-4.61518503	-2.362274355	-2.922920349	-2.317762895	-3.098726522	-2.737611445	-5.724699904	-3.92565128	-3.195979262	-4.604724647	-6.094249547	-5.978466731
-4.217305062	-3.456092119	-4.322291309	-2.690811367	-2.570416389	-1.412879159	-3.151616277	-2.228740584	-5.57170012	-3.232939645	-3.131661656	-4.713990765	-5.39566591	-5.515655149
-3.980434252	-2.778949598	-4.576748294	-1.306899198	-0.722121873	-1.319795304	-3.426081316	-1.405538294	-5.615739085	-2.012138281	-2.329614936	-4.621735175	-5.722297499	-5.279751781
-4.464914749	-3.464594204	-4.490577568	-1.907289846	-1.315832132	-1.653049137	-3.041181828	-1.647550146	-5.230662747	-2.012900519	-2.430119956	-4.577883221	-5.586832405	-5.58731369
-3.89788003	-2.90863312	-4.582514889	-2.180967929	-1.365451227	-2.169659609	-3.357474931	-2.15788298	-4.880708116	-2.701393823	-2.876603819	-4.531173767	-5.728683685	-5.362682974
-4.104661918	-3.144012144	-4.421166095	-0.675189608	0.600680371	-1.012114267	-3.432628743	-0.29063304	-6.021115568	-0.506856061	-1.344135513	-4.750038077	-5.741887966	-5.640479695
-4.358908996	-3.313281431	-4.24336654	-1.161126696	-1.091193544	-1.024774727	-3.166294168	-1.091326686	-5.511039301	-2.063280613	-1.82590876	-4.796120596	-5.538450015	-4.945838145
-4.168187138	-3.023172451	-4.404644669	-0.85979958	0.483651156	-1.210993604	-3.336528567	-0.504361126	-5.106525041	-1.04946615	-1.469280053	-4.548355338	-5.472935208	-5.17542178
-3.794916405	-3.035763969	-4.300146573	-0.749588453	0.286787661	-0.977254871	-3.387643973	-0.432402328	-5.814889592	-0.468130122	-1.521433815	-4.136506915	-5.699163076	-5.258338854
-3.972301264	-3.245953769	-4.536849926	-1.935649569	-0.796140787	-1.997975239	-3.463692004	-1.608567606	-5.694936436	-1.665173621	-2.244886032	-4.370129533	-5.616901403	-5.420298818
-4.219084799	-3.347316477	-4.166691915	-0.972389848	-0.780670106	-0.530225702	-3.080453126	-0.844074135	-5.615493297	-1.653881839	-1.962567209	-4.433872012	-5.418790344	-5.864944268
-3.869784228	-3.220591296	-4.005450512	-2.092101197	-2.485490857	-1.657352508	-2.704337607	-2.222733116	-5.984255259	-3.933792743	-2.815727619	-4.338810225	-5.193358066	-5.997694676
-4.21050163	-3.502192428	-4.328012173	-1.500747627	-1.924746092	-1.233761935	-3.036318594	-1.911517256	-5.371236791	-2.790135931	-2.54362465	-4.024751352	-5.917896931	-5.272834598
-4.146644542	-3.405068112	-3.833642107	-2.558273746	-2.145447125	-1.957049486	-2.707485612	-2.343814354	-5.760495465	-3.281017692	-2.659023638	-4.403679718	-5.099152112	-6.187702804
-4.283607443	-3.357029789	-4.753614983	-1.699158309	-1.412858945	-1.934693609	-3.326810188	-1.706020063	-5.631047973	-2.444363104	-2.208842019	-4.425919051	-6.501662553	-5.452579833
-4.083310885	-3.430440819	-4.334481677	-2.65285323	-1.356426629	-2.852731295	-3.167734445	-2.137473607	-6.020162637	-3.252298512	-2.555679704	-4.976895346	-5.72877758	-6.081702083
-4.74216917	-2.670028485	-4.263694075	-1.912315025	-1.411792981	-1.069726185	-3.104275007	-1.874411087	-6.128814331	-2.531461811	-2.554511558	-4.569726097	-5.646494636	-5.922825148
-4.233772085	-3.287649387	-4.497295847	-1.605291163	-2.329895893	-1.548139043	-3.095404554	-1.824511177	-5.449346618	-3.092596237	-2.766566307	-3.538119171	-5.261083158	-5.493828378
-4.227632904	-2.924396248	-4.442264521	-1.066555686	-0.571564802	-1.218858544	-3.285662362	-0.753700459	-4.863871498	-1.332707331	-1.807782133	-3.533729754	-5.521959863	-5.211730739
-4.137172522	-3.534256634	-5.188899409	-3.500154208	-2.182091607	-3.184092239	-3.618618849	-2.869721424	-6.144759174	-4.190093281	-3.995500496	-4.977918257	-6.829874531	-6.563536181
-4.011450785	-2.023682018	-4.94913911	-3.921388628	-5.174112331	-3.868858586	-3.340647244	-3.84799369	-4.633196312	-4.739518344	-5.026487875	-2.72455758	-5.712229985	-5.008274908
-4.132318553	-2.292197327	-4.998104901	-4.448959424	-6.204500109	-4.293880188	-4.139130449	-4.687820206	-3.889657046	-5.629559402	-5.844731107	-3.964923303	-5.714592246	-4.946631932
-4.268009181	-2.571562398	-4.483421393	-5.076550559	-6.275625878	-4.561166438	-3.687980517	-4.630389614	-4.850742955	-5.153909256	-5.396036383	-4.946845238	-5.995736331	-5.433153162
-3.760795654	-2.242028525	-4.579433786	-4.233516506	-4.971213474	-4.279915262	-3.444326627	-3.155099965	-5.011766646	-3.240238991	-4.238951625	-4.291556799	-5.31740051	-4.889977947
-3.92123042	-1.927758361	-4.511745892	-4.396404095	-4.65328112	-5.071533514	-4.730167368	-2.640038703	-3.805792422	-2.698183285	-3.65452053	-5.208697616	-7.037025804	-3.769160472
-3.668131383	-2.075900942	-4.526330009	-4.857529588	-4.773819234	-4.623973627	-4.058636621	-2.780010487	-3.983369426	-2.661379292	-4.01007993	-3.674429293	-6.006696349	-4.725551894
-4.024092564	-2.745726811	-4.298957191	-4.749057917	-5.884566438	-3.978934326	-3.490839821	-3.609248603	-4.391637339	-3.337793284	-4.903234377	-5.046693775	-5.44795418	-5.122967096
-3.447016363	-2.037565856	-4.579816837	-5.003273569	-5.977777137	-3.59095674	-4.173570226	-2.512580993	-4.46985871	-2.617035533	-4.351997893	-3.508291805	-5.751323824	-4.447091304
-3.748579706	-1.88797494	-4.301421269	-6.403018628	-6.061112127	-7.559703051	-3.620897361	-3.512016358	-5.401079194	-3.119335759	-4.59101957	-2.985850183	-5.629362773	-4.934126632
-4.011099509	-2.777908721	-4.774561247	-3.576699749	-3.372484518	-3.728870701	-4.35827318	-1.829116927	-3.224133269	-2.099104053	-3.742882644	-2.424402919	-5.758567221	-5.160723126
-4.262579437	-3.004948492	-5.054857541	-4.675702973	-4.942911456	-5.370113073	-4.148488727	-3.141732338	-3.490024364	-3.181382014	-0.953229926	-3.950825756	-6.018865565	-4.5690197
-3.519620547	-2.241104431	-3.107391195	-7.399548222	-5.816986399	-7.906240559	-2.795045778	-5.764014426	-5.884357396	-5.75595524	-6.504778292	-4.203378636	-5.14514468	-4.872686428
-3.986166643	-2.404681148	-4.789845029	-4.743298621	-6.757874705	-5.743647851	-4.072945349	-3.386829352	-3.434713284	-3.324140001	-4.82113261	-3.453759213	-6.205677806	-4.566121523
-4.06207292	-2.306361097	-4.496519216	-5.570712289	-5.742366717	-5.174069172	-3.960983177	-3.438614174	-3.797626056	-3.404877293	-4.576103669	-4.458481287	-5.887372281	-5.027410736
-4.034908865	-2.984994823	-4.14790297	-4.880575237	-5.598950332	-4.350849218	-2.901381957	-3.686350933	-4.408332491	-3.59440191	-5.068151355	-5.140505373	-5.35966099	-5.42999014

-3.850763227	-2.456710339	-4.621684002	-2.76747375	-3.754296747	-2.541228492	-3.776339568	-2.78888404	-4.235094928	-3.035409256	-3.836881981	-4.777385041	-5.650927222	-5.024671974
-3.286435645	-1.632162338	-4.888039204	-4.95548058	-5.393351332	-5.189002137	-3.794863927	-3.483251429	-3.965483034	-3.446706691	-3.348456781	-2.574993459	-5.698436568	-2.967860891
-3.098393388	-1.606198336	-4.367697545	-4.267838016	-5.026577697	-4.321766527	-3.246027759	-3.360994665	-5.133650346	-3.217407069	-3.909499067	-4.024278618	-5.539995105	-3.627233134
-3.496788384	-3.17119374	-3.418417498	-7.087306564	-4.699920058	-6.660787176	-2.909781549	-4.344565517	-5.918438052	-4.543959963	-5.949558964	-4.614662921	-5.386540754	-5.075740596
-4.183594455	-2.633712763	-4.029660693	-6.262198648	-5.295030261	-6.064156808	-3.505729321	-5.105238495	-4.73321003	-5.269663924	-5.818988265	-5.064246217	-5.662040889	-5.519717842
-2.952718687	-1.934661861	-4.849507451	-4.210229285	-4.982966364	-3.848956024	-4.047821463	-2.812965733	-3.665474974	-3.053540621	-2.84689995	-1.939910988	-5.736069219	-3.203725257
-2.633459031	-1.586740106	-4.504552722	-4.241325667	-4.19833988	-4.630164409	-3.946257071	-3.846556728	-4.519629031	-4.177943697	-2.461685836	-2.752752152	-5.391651568	-2.726501129
-3.960343292	-2.64759298	-4.0223991	-4.81572689	-4.977710388	-4.738720503	-3.669631915	-2.912398147	-4.893318921	-2.82088681	-4.579013506	-4.618832815	-5.660954825	-5.103303686
-3.648473158	-2.006381296	-4.659002108	-4.640147206	-6.796525475	-4.762551257	-4.243983077	-4.718977131	-3.359552137	-5.52332613	-5.593568431	-3.487415481	-5.669973452	-4.408647777
-4.030699756	-2.468423901	-4.884940276	-4.658716462	-5.676053405	-4.868106918	-3.947328842	-3.929050165	-3.781343747	-4.089766696	-5.131390944	-5.007429451	-5.545784111	-5.064291613
-4.202097848	-2.300955202	-4.811756374	-3.382375875	-2.867378593	-4.139179919	-4.248085986	-2.102920909	-2.883949711	-2.333249858	-3.319076881	-3.359915849	-6.575301582	-4.455426318
-4.422344137	-2.457397112	-4.858204678	-3.959385174	-3.605554817	-3.839780925	-3.634724359	-2.774993857	-3.059591904	-3.053011117	-3.926364357	-4.559326506	-5.721070588	-4.777610569
-4.005258209	-2.372007799	-4.72209955	-6.353015758	-4.910995736	-6.098514634	-3.894753852	-4.937902734	-5.454383992	-4.988422929	-5.216786363	-4.636751438	-5.997053046	-5.150249148
-3.968168259	-2.469951578	-5.089913441	-4.886444304	-5.509457922	-4.500320844	-4.418892965	-4.402115431	-3.355912152	-4.685212738	-4.320698312	-1.144288669	-6.205328807	-4.502827372
-3.596383345	-1.79335236	-4.670727239	-5.211891785	-5.329144578	-4.791188664	-4.519273486	-3.683931121	-3.903678643	-4.146464504	-3.563240121	0.215106346	-6.760194637	-3.735632404
-3.601612449	-1.699505259	-4.693922597	-3.256017326	-4.321677649	-3.230736766	-3.785799413	-2.314316198	-4.52863637	-2.275095556	-2.950797303	-0.770356627	-6.369308114	-3.441789982
-3.806658068	-2.318723799	-4.600176929	-2.77729747	-2.279335367	-2.788421755	-4.00274792	-1.435305181	-4.117982303	-1.515198773	-2.975282087	-4.263310936	-5.74031795	-4.829689363
-4.082166305	-2.855167935	-3.902747764	-6.452409246	-4.782963358	-6.406080602	-3.566276245	-4.603219654	-5.962671544	-4.761102612	-5.70880681	-5.421917159	-5.295073764	-5.476313541
-4.191136176	-2.593058404	-4.452186237	-6.267728074	-7.005341806	-5.94349335	-3.117897257	-5.522504165	-4.552226629	-6.297566019	-6.544125825	-4.391777555	-5.35207919	-5.355246093
-4.402957211	-2.96107993	-5.266228954	-2.896157011	-2.914726996	-2.717823295	-4.267018088	-1.623131709	-3.545898999	-1.581516831	-3.001837608	-3.998380405	-5.869619632	-5.216203963
-3.962405408	-2.648430738	-4.596031539	-3.74484883	-3.547532737	-3.485136847	-3.50081991	-2.563794275	-5.031566171	-2.569262393	-3.901317484	-3.13305704	-5.458846453	-5.192385211
-4.301918039	-3.370930451	-5.341720476	-1.201568191	0.207073979	-1.559453444	-3.332272653	-1.030518259	-4.416984272	-1.123901045	-0.650043762	-3.16865973	-6.248250657	-3.503458223
-4.447001981	-3.354123837	-5.233193164	-3.107738353	-3.61158346	-2.715120561	-3.810289033	-2.42096762	-4.661629743	-2.651387	-4.035945083	-5.383040292	-6.379891169	-6.029876446
-3.967209489	-2.031379551	-6.972729235	-4.269820239	-4.518585594	-4.408502123	-3.824004869	-3.299785491	-4.224397339	-3.21121689	-3.852669938	-2.878635167	-7.77536339	-4.548832941
-4.204018732	-2.103855187	-5.141446787	-2.456228558	-3.584543551	-2.497776099	-3.410748931	-2.223261917	-5.279617555	-2.15830163	-3.431264068	-4.544055902	-6.637278851	-5.238751877
-3.777552752	-3.530610111	-4.501097978	-2.664559221	-1.12637562	-2.50793649	-3.794906549	-2.179370151	-4.932733141	-2.468190592	-1.301864731	-3.567397224	-6.203895357	-3.013279678
-3.830484517	-2.962219588	-5.426366184	-3.852099002	-2.354644235	-3.360616918	-3.163589085	-3.39130158	-4.199230921	-3.487647676	-1.412224546	-3.407342504	-6.476292988	-2.563972728
-3.976517796	-3.261470139	-5.238857611	-4.668720335	-1.840007552	-4.636871304	-3.276558331	-3.609287991	-4.870845028	-3.386687225	-2.267377913	-3.951574908	-5.949969407	-2.80767936
-4.200955367	-3.420932539	-5.110494528	-1.886098069	-0.151563923	-2.133877541	-3.174034369	-1.377746403	-4.835158911	-2.158947121	-1.244445458	-3.669500892	-5.885744633	-3.519933991
-4.064540382	-3.403560961	-4.80727939	-0.910996949	0.39994961	-1.016858339	-3.557779631	-0.934316788	-4.758436115	-1.321703416	-0.859344945	-4.104026515	-5.963074346	-3.374680851
-3.948276589	-3.073645902	-5.19386256	-3.420748305	-1.555288336	-3.248593312	-3.412942772	-2.708444401	-4.640669506	-3.522494184	-1.531843227	-3.405625817	-6.188439947	-2.794975741
-3.720907982	-3.09639088	-4.600455115	-3.313854019	-1.816029566	-2.652125388	-3.736112076	-3.18315493	-4.714615998	-3.124578895	-1.872972854	-2.95369827	-6.326421266	-3.232848901
-3.964994034	-3.032897937	-5.143729758	-1.340080605	0.820483247	-1.571238533	-3.308320145	-0.614130149	-4.177464319	-1.268128525	-0.424577602	-3.314854246	-6.492576519	-3.106528101
-3.800176565	-2.988176431	-5.22428877	-2.189711449	-0.758372229	-2.548479789	-3.151817421	-1.707702608	-3.778649407	-2.501702297	-1.025423714	-2.178103063	-6.170436308	-1.076358695
-4.223193728	-2.589338378	-4.876799653	-4.954471132	-5.844849609	-4.5092162	-3.762830562	-3.879648281	-3.976460634	-4.212395042	-5.043319254	-4.524090066	-6.159445746	-5.429411955
-3.612127157	-2.217753911	-4.314369126	-6.205676408	-6.962665843	-5.962125505	-3.763530097	-5.490357844	-3.806319047	-5.678316468	-5.405431094	-4.170691914	-5.39741887	-4.621277517
-3.502892616	-2.176126444	-4.95657537	-2.80588618	-3.27874213	-3.319909656	-3.998911406	-2.007392432	-2.91241642	-2.35268567	-3.259760878	-2.220465187	-5.259034709	-4.05553409
-3.906672723	-1.964441196	-4.695695883	-3.251433272	-4.302085586	-3.584845728	-3.794077688	-2.5828004	-3.662076236	-2.694341082	-3.400907081	-2.961189845	-5.622563861	-3.728996623
-3.810600286	-1.999676203	-4.765481078	-6.40191476	-7.027278647	-7.192379093	-4.724989069	-5.065188978	-3.846078047	-5.2000997	-4.815124639	-3.324202983	-5.616191852	-4.608283541
-3.528105625	-0.70433961	-4.042928155	-8.295368816	-6.636582967	-6.098626518	-3.841508081	-7.419903768	-4.656586126	-7.201986484	-5.246391453	-2.284910967	-5.756482281	-4.101512774
-3.974909615	-2.204467806	-4.63340385	-3.678721733	-4.797514759	-4.078382265	-4.260934308	-3.309508301	-3.170316304	-3.82607611	-4.624947338	-4.832387503	-5.84467212	-4.418180005
-4.383142834	-2.664883095	-5.20089919	-4.114500027	-5.861678028	-4.599460424	-4.305932977	-3.917746467	-3.084033946	-4.318694738	-4.661409824	-5.137121101	-5.847847467	-5.291902109
-4.489254599	-2.739112879	-5.169414858	-4.579809228	-4.495215365	-4.535456831	-4.270861919	-3.257715916	-3.470832765	-3.368146748	-4.196195308	-3.238592373	-6.105586214	-5.123979233
-4.603422899	-3.064946996	-4.756698719	-6.524226901	-6.519983934	-5.658146528	-4.003177062	-4.965613504	-4.140751683	-4.702348694	-4.958064423	-2.419060762	-6.064817442	-5.523465755
-4.1258117	-2.12788191	-5.185001948	-6.991858002	-6.441135008	-6.336717354	-4.288699082	-5.61560248	-4.820880978	-5.776136686	-5.436433719	-3.25984604	-5.989171519	-5.192699009
-4.11708039	-2.234401144	-4.949213799	-3.501713744	-3.314013083	-4.557685819	-4.182705076	-2.499348954	-3.706709405	-2.398487526	-3.197970933	-1.216103605	-6.106673575	-4.605473107
-3.828442612	-1.456310985	-4.438917291	-5.266432616	-5.910263159	-4.436986613	-4.069100622	-3.834653291	-4.19684795	-3.62285549	-3.348026668	-1.745034832	-5.637243651	-3.449903395
-4.224201383	-2.784382032	-4.711437051	-5.197997095	-4.219367168	-4.737690423	-4.514025264	-2.157269019	-4.058671925	-3.01167052	-4.83756825	-5.515382644	-5.646826273	-5.509322612
-4.067564647	-2.332837425	-5.078001519	-3.405805723	-4.066711611	-3.793444339	-4.361637592	-2.136411984	-3.135531696	-2.275842133	-3.329045562	-2.729787231	-5.266356366	-3.968357831

-3.656206407	-3.052394485	-5.073184849	-1.948170485	-0.040469901	-2.279417719	-2.8536384	-1.4336722	-4.372040474	-1.317531861	-0.681755177	-3.545135682	-5.683129943	-2.493469824
-3.612138175	-2.723197918	-5.163088998	-1.692000093	-0.433225373	-2.418363064	-2.915778577	-1.624821739	-4.086392713	-1.603048893	-0.610120967	-3.449019776	-5.998714399	-2.183029439
-3.82523986	-3.245222474	-5.051235961	-1.298649446	0.182085159	-2.1109058	-3.157294881	-0.961808276	-4.070846525	-1.046624373	-0.434584417	-3.650911132	-5.94866119	-2.770513856
-4.17751292	-3.264823888	-5.155531675	-1.830514218	-0.187739065	-2.159672846	-3.118471746	-1.594344918	-4.538542128	-1.630176748	-0.475511355	-3.496057803	-5.874806374	-3.012305122
-4.410839643	-3.701085591	-5.284504225	-1.208710378	0.383251173	-1.331866105	-3.381491922	-1.018677515	-4.78662262	-1.312963983	-0.67452183	-4.393505855	-6.121789398	-3.635160837

hsa-miR-296-5p	hsa-miR-30c-1-3	hsa-miR-30d-5p	hsa-miR-3122	hsa-miR-3131	hsa-miR-3135b	hsa-miR-3141	hsa-miR-3147	hsa-miR-3150b-	hsa-miR-3151-3	hsa-miR-3151-5	hsa-miR-3154	hsa-miR-3156-3	hsa-miR-3158-5
-1.526610898	-2.508292803	-2.816447832	-5.353651815	-2.014194304	-0.646207203	-0.780453576	-3.147258329	-6.108766218	-4.075844508	-4.567743026	-3.062975165	-4.232250633	-3.800078181
-2.126646922	-2.967963874	-1.935077003	-5.110774574	-2.93818705	-0.288145549	-0.749785184	-3.351633134	-6.053526171	-4.218111567	-4.248673035	-3.105101368	-4.595470932	-4.12503619
-1.746636282	-2.490837312	-1.976753676	-4.826894646	-2.353736088	-0.241978859	-0.434489005	-2.960338848	-5.943629637	-4.16864331	-4.550906319	-2.576518395	-4.286735707	-3.9419416
-1.682845884	-2.756033862	-2.168071781	-4.994674844	-1.882967688	-0.485828412	-0.53858166	-3.043139904	-5.783955943	-4.233493218	-4.367346093	-2.515660108	-4.416861503	-3.658431181
-1.175626353	-2.4445462	-2.024576321	-5.082172211	-1.60747103	-0.24469582	-0.478006355	-2.999548239	-5.61694007	-3.839969297	-4.481731726	-2.57880374	-4.147813308	-3.754857767
-1.582984284	-2.64659078	-1.82325943	-4.671461816	-2.155418255	0.048879301	-0.629148045	-3.379344607	-5.743285893	-4.050848089	-4.518902404	-2.778655561	-4.60660992	-3.914305879
-1.376476349	-2.472986362	-2.485838807	-4.737767297	-1.96035232	0.382023734	-0.338663029	-3.467838811	-5.72294012	-3.935829618	-4.480887394	-2.605583124	-4.294702919	-3.671097552
-1.992332212	-2.929685787	-1.70341564	-4.75406516	-2.569866853	-0.265707955	-0.646489297	-3.29762711	-5.884729968	-4.313134228	-4.486569708	-2.664876241	-4.749484994	-3.874732665
-1.833284479	-2.756488928	-1.964916623	-4.951362769	-2.326278936	-0.315655188	-0.672749906	-3.509935235	-6.002107564	-4.393907467	-4.719773078	-2.663927721	-4.630241143	-3.929630944
-1.757128267	-2.827849895	-2.457414365	-4.75014624	-2.202525866	-1.437237059	-0.784670074	-3.666358296	-6.119352992	-4.323667347	-4.479025974	-2.681896092	-4.655399527	-3.543227031
-1.744266001	-2.88409524	-1.545785503	-4.757984192	-1.695243189	0.344193824	-0.693569758	-3.588727883	-5.862472737	-4.585722264	-4.53545333	-2.500177772	-4.916090486	-3.34252468
-2.290611689	-2.779610397	-3.912548827	-5.101080166	-2.840103275	-1.188407628	-0.856354128	-3.55432993	-6.944142221	-4.892463418	-4.35255051	-2.963399664	-5.230435536	-3.93354734
-2.362811963	-3.078559413	-2.671603454	-5.151027944	-2.476234547	-0.718880426	-0.649252875	-3.589816583	-6.50202256	-4.876905977	-4.492678516	-2.774298423	-5.255543187	-3.896266338
-1.916783864	-2.811647549	-2.834615537	-4.879374163	-1.986667125	-0.799775964	-0.689536416	-3.447834333	-5.789452486	-4.641462705	-4.406721993	-2.731308	-4.747454993	-3.39990585
-2.058681513	-2.976708893	-2.419716794	-5.061704312	-2.742727084	-0.707040149	-0.952027597	-3.836773409	-5.853939231	-4.692505135	-4.601578972	-3.084157314	-5.118504595	-3.780246743
-2.189424297	-3.097448715	-2.597155621	-5.120745218	-2.415176032	-1.112730081	-0.658761109	-3.33036102	-5.899872131	-4.676347277	-4.495508298	-2.671037035	-4.915217985	-3.785384213
-2.161709148	-2.913564423	-2.782075668	-4.802789513	-3.053255493	-0.795960178	-0.963351434	-3.675993975	-5.623953702	-4.813721941	-4.559859271	-3.129812337	-5.032252147	-3.807712047
-2.292482142	-2.922411478	-1.948751591	-5.064863875	-2.932344095	-0.470814362	-1.00959885	-3.884758303	-6.365241158	-5.05866669	-4.583713408	-2.879436446	-5.116754318	-4.031770216
-1.870417898	-2.858483545	-2.024655757	-5.147676468	-2.00014636	-0.179473538	-0.873201873	-3.438353638	-5.894836254	-4.696528376	-4.212525127	-2.54150635	-4.830562063	-3.120964551
-2.034217575	-2.812618771	-1.970797108	-4.946129975	-2.523278695	-0.097498104	-0.862067781	-3.703275469	-5.424709558	-4.890133929	-4.595913854	-2.651790286	-5.138796651	-3.5899713
-2.133152809	-2.899293897	-2.134384467	-4.784495634	-2.860516229	-0.732769279	-0.840974596	-3.687818703	-5.86816649	-4.62314953	-4.598013277	-3.092915053	-4.972739671	-4.079212574
-2.138964925	-2.965145741	-2.862269857	-4.922258139	-2.911726546	-0.143868669	-0.955121359	-3.898294375	-5.859619245	-4.796581885	-4.563879589	-3.264570023	-5.088239285	-3.776601132
-1.890166442	-2.599285619	-1.857666723	-4.989792568	-2.070622657	-0.260449877	-0.687630933	-3.502011215	-5.805037658	-4.616785033	-4.534962105	-2.384656546	-4.68325284	-3.580491069
-1.473549072	-2.078693413	-3.001150499	-5.027265995	-1.593279015	-0.655263094	-0.555965402	-3.434539941	-5.674739778	-4.409382865	-3.992931659	-2.239934302	-4.207204349	-3.214025984
-2.023809271	-3.006663221	-2.630399011	-4.813099101	-2.551174062	-1.656845445	-0.792810734	-3.409169908	-5.832937961	-4.664803466	-4.36556083	-2.470771961	-4.822540468	-3.883520138
-1.206221843	-2.249204203	-3.120901426	-4.903969186	-1.167471548	-0.860016071	-0.474013261	-3.366320196	-5.621615536	-4.414891322	-4.325682039	-2.321686487	-4.011902896	-3.194357692
-2.369400391	-3.129231786	-2.253292806	-4.983035863	-2.713653864	-0.678698775	-0.889377828	-3.540902791	-6.42157876	-4.795013357	-4.486555999	-2.950311784	-5.315604651	-4.140195799
-1.84335502	-2.327689261	-3.300277613	-4.969397124	-2.144265872	-0.802770103	-0.815742129	-3.826474159	-6.299417986	-4.784009933	-4.357253301	-2.785691026	-4.905415855	-3.285552884
-1.681637539	-3.031747009	-2.908149411	-5.664557434	-2.806493975	-0.581998792	-0.94447646	-3.63448809	-6.633134229	-4.654504257	-4.526807695	-3.397101218	-4.813190585	-4.266261842
-1.970880853	-2.727837657	-2.518786727	-4.966087764	-2.330160121	-0.687393062	-0.608663114	-3.410195626	-5.888203491	-4.533570581	-4.597029565	-2.479014982	-4.680533434	-3.867493564
-2.060428837	-2.890783324	-2.266178946	-4.84925583	-2.019473453	-0.586575122	-0.675182259	-3.845280841	-5.670886566	-4.641509646	-4.372640141	-2.554215018	-4.831301863	-3.609999304
-2.424107954	-3.356838957	-4.092504163	-5.396558657	-2.915925707	-0.653226501	-1.637482005	-4.212562522	-7.493008257	-5.433911395	-4.525055479	-3.788075581	-5.435148238	-4.723990789
-2.720348269	-4.351724034	-5.183152255	-5.272118492	-3.382691829	-1.787531077	-1.298119105	-3.41385517	-5.598778532	-4.86950963	-4.453319651	-3.574079097	-5.569870743	-5.473637682
-2.540641042	-4.781660997	-4.842912041	-5.080861328	-3.84091995	-1.855683798	-1.159890445	-4.217730366	-4.428820773	-4.816970846	-4.435013547	-3.353749586	-5.328822319	-5.656552467
-2.350159531	-3.754591843	-5.293987556	-5.644375194	-3.364670358	-2.141692383	-1.283474192	-3.977780378	-5.687108357	-4.956719764	-4.772859146	-3.51876317	-5.339007313	-5.140231397
-1.885432332	-4.207636033	-4.213191675	-5.4105856	-3.860103121	-1.630261129	-1.060575372	-3.426254977	-5.052039685	-4.568839489	-4.628705867	-3.897482162	-5.252869284	-5.704997453
-2.562418631	-5.031057745	-4.281241462	-5.705705655	-3.503289786	-1.684765112	-1.562279538	-4.44912331	-4.450311517	-6.37364108	-5.321311716	-4.29814537	-6.158565939	-5.870906448
-2.349572413	-4.059062147	-4.390786336	-5.415042688	-3.575930115	-1.955431816	-1.126021218	-3.856428756	-5.240127091	-4.642871464	-4.260216551	-3.602386664	-5.378558784	-5.576769812
-2.146749352	-3.391297856	-4.622015485	-5.247034788	-3.155553055	-2.341453284	-0.840255308	-3.484932131	-5.189109597	-4.561175605	-4.196188574	-3.319296628	-5.139809081	-5.264657404
-2.083342713	-4.725480414	-5.12501347	-5.533631172	-3.194369616	-1.38098745	-1.687044731	-4.00555692	-5.506850821	-4.829219291	-4.593781067	-3.649879482	-5.591012429	-5.132074737
-2.053680342	-3.525148993	-5.476720165	-5.448409905	-3.129259574	-1.308858799	-1.164194617	-3.995877443	-6.121803122	-4.506180884	-4.264414885	-3.728377285	-4.989728078	-5.829136588
-2.764709533	-4.233061197	-3.586459716	-5.133017599	-3.484702551	-2.003990979	-1.028722345	-4.614835165	-4.622311794	-4.942533037	-4.199666344	-2.946333001	-5.989852798	-4.932274047
-2.752187167	-4.527938247	-4.567448288	-5.213122243	-3.10649296	-2.130649009	-1.501808844	-4.298064525	-4.40402199	-5.196018702	-4.623819081	-3.187564317	-5.795815773	-3.616762046
-0.698928353	-2.461059688	-5.241385335	-5.584215695	-0.287412054	-1.30473391	-0.708023975	-3.118106755	-5.474125679	-4.009715691	-4.030211968	-2.666602327	-4.131564613	-4.378088352
-2.558256327	-3.921092265	-4.362863035	-5.237378667	-3.46775325	-2.418472705	-0.991531017	-3.849435924	-4.63872536	-4.899113228	-4.740393269	-3.2492983	-5.423322149	-5.25149656
-2.487559076	-4.162202205	-4.62292707	-5.275730371	-3.808008106	-2.100634354	-1.116099928	-3.851713996	-5.356337614	-5.045410634	-4.700998546	-3.387134407	-5.528990925	-5.355114493
-1.815860665	-3.282454728	-4.861445992	-5.466672876	-2.983566181	-2.123924041	-0.61814483	-2.941019762	-5.91939494	-4.466852446	-4.327204831	-3.472671282	-4.763085941	-4.833433922

-2.462042283	-3.36267639	-3.387654279	-5.302355508	-3.307738743	-2.087615779	-0.979855342	-3.892786028	-5.593897485	-4.989723242	-4.469566919	-2.94865654	-5.118370442	-4.787261729
-2.668521467	-4.810172851	-4.137129685	-4.773668092	-3.003734559	-1.337992272	-1.375581888	-3.630961416	-4.560611844	-4.932850207	-4.111307251	-3.311486062	-5.87057156	-5.08936684
-2.160168244	-3.954914076	-4.471373772	-5.14662161	-3.070421739	-1.748045457	-1.010428182	-3.032876387	-5.838162889	-4.774670403	-3.884309561	-3.42665929	-5.621862443	-4.982857047
-0.896261418	-2.654939039	-5.53432141	-5.518817438	-0.806770568	-1.713988804	-0.812014339	-3.065197592	-5.560753875	-4.358334073	-3.641530698	-2.465711018	-4.437675213	-4.104729476
-1.263106337	-3.239532601	-5.31258268	-5.419064324	-1.588064184	-1.767021706	-1.143606749	-3.568935638	-5.399395929	-4.620160901	-4.507601373	-2.999763008	-4.779988477	-3.627517343
-2.821946545	-4.578697146	-4.354312039	-4.833761578	-2.706387967	-1.794965508	-1.783964878	-4.148996071	-5.471960248	-4.948482262	-3.43772808	-2.718583366	-5.459622349	-4.8423949
-2.226512931	-4.38778398	-4.657870312	-4.875636405	-2.501081956	-1.304990444	-1.805042346	-3.434723572	-4.943537493	-4.610762439	-3.603493014	-2.955504825	-5.215591877	-5.256183218
-0.881607577	-3.414127352	-4.670093565	-5.207946089	-1.881593582	-1.923036936	-1.131896965	-3.601598808	-5.568193339	-4.712524948	-4.498962856	-3.526053879	-4.932859819	-4.856936907
-2.20407649	-4.162878477	-3.977428517	-5.585679948	-3.245041558	-2.074086623	-1.117075788	-3.972653359	-4.383911326	-5.225143731	-4.53975558	-3.031945967	-5.474185706	-4.861923934
-2.478906925	-3.919580366	-4.662573252	-5.529744616	-3.572005715	-2.169173387	-0.957073238	-3.893434554	-4.505188004	-4.923149461	-4.354008452	-2.919388991	-5.466358955	-4.532004792
-2.975667178	-3.800241027	-3.431504323	-5.500654343	-3.52543333	-0.681222383	-1.561584871	-4.346441703	-4.889396264	-5.187791438	-4.293737918	-3.275683409	-5.7349985	-4.541254773
-2.619434548	-4.293972395	-3.668842451	-4.642939197	-3.474073707	-1.396202238	-1.047696868	-3.389283597	-5.066705463	-4.937274557	-4.403983884	-2.996128551	-5.231537119	-5.003485968
-2.090954248	-4.138742704	-5.536465654	-5.766133147	-3.303666895	-1.716899725	-1.497660038	-4.033954611	-6.027889243	-4.9346838	-4.355224682	-3.550871497	-5.494079472	-4.955059797
-2.852945751	-5.335150574	-4.512193068	-5.325426934	-3.558588414	-2.164777651	-1.523488476	-4.204440657	-5.218583143	-5.29286911	-3.994168973	-3.037582041	-5.699917949	-5.130008376
-2.973916418	-4.868577428	-4.925266744	-6.111389464	-3.097542537	-2.065339275	-1.891291599	-4.661850305	-5.765005601	-6.438708924	-3.98170973	-3.640281082	-6.498019917	-5.408149028
-2.34655961	-4.482040908	-3.951123408	-5.134902743	-3.253101343	-1.661061847	-1.479708079	-3.81584825	-5.971663792	-5.140018423	-4.260199771	-3.436556551	-5.883929432	-5.030095247
-2.276969576	-3.759747095	-2.77328347	-5.628189295	-3.879255519	-2.226677715	-1.136724917	-4.18395959	-5.444807054	-5.145103137	-4.454181954	-3.729668204	-5.619464879	-5.173354824
-1.492450869	-3.618707337	-5.969184919	-5.63639359	-2.793348965	-1.719658037	-1.206655575	-3.654351978	-5.772075556	-4.199805899	-4.4791739	-3.570868469	-4.775953878	-4.4447323
-1.961881009	-3.540341546	-5.046164068	-5.632805428	-3.180272273	-2.216399816	-1.042040074	-3.089891569	-5.504521079	-4.648077288	-4.580921167	-3.322376231	-4.986919242	-5.292698976
-3.036301257	-3.930949796	-3.42924017	-4.66330987	-3.514655578	-1.733013317	-1.284833292	-4.555201524	-4.860169907	-5.237197792	-4.987406149	-2.670717022	-5.764516788	-4.504054131
-1.847894664	-3.140379278	-4.014313487	-4.759879518	-2.762770475	-1.155635938	-0.907457239	-3.48859979	-5.378498968	-4.614714363	-4.643757276	-2.820213995	-4.74996388	-4.118919409
-3.018175113	-3.989153401	-2.295664639	-4.822929268	-2.422359868	0.099390372	-1.319637998	-3.771042235	-6.357144688	-5.040399163	-3.959791126	-3.080676163	-5.134453833	-4.039761096
-2.731530459	-3.209620425	-4.264274458	-5.121428386	-3.020703899	-1.993447094	-1.041286463	-4.405803913	-6.062136127	-5.447536811	-4.749600258	-3.261425233	-5.716726621	-4.501676832
-3.180371649	-4.406867132	-5.126624055	-5.119165119	-3.492556137	-1.774833925	-1.346038786	-4.058545941	-7.810847469	-6.350814488	-4.582461286	-3.664969393	-7.245451826	-5.44828549
-2.630510421	-3.023498792	-3.652911953	-5.170258872	-3.0825671	-1.656214038	-1.143047196	-3.939838539	-6.462852965	-5.055421875	-4.510505872	-3.455257268	-5.518087705	-4.806467946
-2.674976718	-4.034234275	-2.853844225	-4.464313047	-2.051934127	-0.326976028	-1.247252141	-3.819689943	-5.616840947	-5.344452821	-4.156012491	-3.234798332	-5.7956941	-4.332780847
-2.971820656	-4.099648628	-4.187625144	-4.370605885	-2.088782962	-0.528763213	-1.505370367	-3.286129455	-6.324381651	-5.087025696	-3.677735894	-3.337720875	-5.046540554	-4.024788853
-2.662366281	-3.931155923	-4.471306236	-4.577542653	-2.493932452	-0.531825064	-1.660734612	-3.634101837	-6.590140578	-4.791750088	-4.106717508	-3.383266036	-5.201610042	-3.617179579
-2.916008054	-3.731468644	-3.008555779	-4.857335279	-2.54499137	-0.438653369	-1.328440169	-3.490998838	-6.304586939	-5.040541215	-4.016010739	-3.078467546	-5.348984921	-4.10100168
-2.473134787	-3.995877878	-1.796662566	-4.487839753	-2.156299873	0.17459888	-1.144321023	-3.813027748	-5.656909902	-4.904081111	-3.964179291	-2.754165198	-5.30461049	-3.986271171
-3.179831079	-4.470567474	-4.131006912	-4.444774504	-2.127464112	-0.341226341	-1.46218265	-3.858320727	-6.107772887	-5.175516155	-3.704842343	-3.08747236	-6.186671946	-4.080359843
-2.343114396	-3.852401644	-3.63664764	-4.28897983	-2.066012674	-0.716656679	-1.624609932	-4.14299322	-5.884766279	-5.139156198	-3.692252933	-3.253920482	-5.072396468	-3.870341389
-2.95062993	-4.008653171	-2.282128172	-4.368347232	-2.283954387	0.124798059	-1.379888982	-3.510330758	-5.998352926	-4.909381719	-3.766137796	-2.940327791	-5.041002192	-3.820922774
-2.948133201	-4.052606138	-3.464324885	-3.954710713	-2.068646053	-0.452111665	-1.349281336	-3.559865716	-6.345565617	-4.980041593	-3.431604656	-2.945501577	-5.2395576	-3.463075608
-2.390438489	-3.840665978	-5.205588756	-5.217952513	-4.049800114	-2.338243393	-1.140778801	-3.953406674	-5.409146559	-4.927589468	-4.51063985	-3.403774294	-4.905181731	-4.646718439
-2.005547475	-3.68424769	-5.448129441	-5.165696699	-3.350422511	-1.937510498	-0.896349796	-3.966813405	-4.54549967	-4.6672459	-4.32105167	-3.301408927	-5.320374952	-4.939816387
-2.822203514	-4.137451622	-3.377573597	-5.260348236	-3.355828295	-1.87339445	-1.551672947	-4.033913706	-4.27613696	-4.712835919	-3.399505715	-2.97381567	-5.354518312	-4.430515405
-2.476011096	-4.253643648	-3.382524036	-4.710468025	-2.710280539	-1.848571714	-1.149443392	-3.497003375	-5.138503641	-4.694702164	-4.320707959	-3.013573782	-5.821749054	-5.260027137
-2.4788442	-4.971943992	-5.250286754	-5.27294253	-3.6291576	-2.256855971	-1.230562471	-4.43116347	-5.198581792	-4.944098629	-4.699615527	-3.715872066	-5.806645556	-5.826678403
-1.82479602	-3.572004882	-5.589139785	-5.481286479	-2.321967722	-1.589350618	-1.539241761	-3.7058736	-5.126845307	-4.553133997	-4.05496311	-3.409894658	-5.198145172	-4.81926843
-2.505562571	-4.228350852	-3.276627566	-4.830954895	-3.446097453	-1.90933623	-1.041840096	-3.866355277	-4.51509729	-4.969368176	-4.256486238	-3.047931671	-5.500269109	-4.832681375
-2.723858082	-4.080471258	-3.943341122	-5.054608715	-3.676887359	-2.10054396	-1.230683053	-4.335779013	-4.834882428	-5.070989677	-4.416609124	-2.971237288	-5.505394861	-4.661156122
-2.893567175	-4.477292931	-4.393349043	-5.432174901	-3.482411044	-1.170675553	-1.270651061	-3.821946749	-4.944317869	-5.296934867	-4.111624881	-2.67724038	-5.807751253	-4.92389895
-2.088459702	-4.468903039	-6.237508961	-5.69215754	-2.929188511	-2.001986107	-1.791892934	-3.811395923	-5.731543311	-5.213923138	-4.604865807	-3.85200978	-5.703300298	-5.595773258
-2.751215246	-4.22798921	-6.07394037	-5.679023042	-3.55269274	-1.719021731	-1.907782914	-4.671177305	-6.422065901	-5.286443305	-4.304416794	-3.630256977	-5.844875975	-4.550983401
-2.828440902	-3.993453181	-3.507568203	-5.433744075	-3.925902994	-2.37858091	-1.624817581	-4.583880995	-5.424449984	-5.353840289	-4.428858751	-3.212164081	-5.646227143	-4.702500931
-2.50755606	-4.860223385	-4.347247282	-4.932310108	-2.711281163	-2.039478737	-1.499205166	-3.666193477	-5.081236789	-4.742857732	-3.960011036	-3.694237437	-6.473178567	-5.457204714
-2.529842774	-4.334794362	-4.571271422	-5.442596722	-3.926092903	-2.664793135	-1.252826129	-4.215428567	-4.89275051	-5.098279986	-4.65623236	-2.858753034	-5.893918085	-4.92373166
-2.65248077	-4.485792859	-3.11486305	-4.279061175	-2.855445606	-1.580901323	-1.440115129	-3.84526648	-4.973020439	-4.933229482	-4.462152796	-2.944591823	-5.924287275	-5.165778926

-2.793980966	-3.815587986	-2.814888859	-4.438882777	-2.241458321	-0.386558167	-1.388311757	-3.212594719	-5.871204662	-4.81883709	-3.532415962	-2.999956742	-5.126921789	-4.000284393
-3.104342667	-4.238731967	-2.850167466	-4.297286246	-1.916969431	-0.061821136	-1.359199327	-3.199927506	-5.590036572	-4.746096497	-3.339769201	-2.930135549	-4.94712509	-4.110755136
-2.923642292	-4.055793966	-2.627012845	-4.624915782	-2.331744938	0.43353531	-1.361778359	-3.580961478	-5.514348637	-4.695245601	-3.572807847	-2.87724451	-4.980810425	-4.261614779
-2.979412484	-4.081066838	-2.865564431	-4.788171858	-2.418304346	-0.076825287	-1.233223813	-3.601764539	-6.181449906	-4.971518408	-3.81275187	-3.090455005	-5.244746904	-4.316775766
-2.964109706	-3.889946424	-2.059866363	-4.97301235	-2.48477506	-0.308959905	-1.287637653	-3.625155308	-6.095487123	-5.040634283	-3.998398898	-3.066124669	-5.276624178	-4.206915719

hsa-miR-3162-3	hsa-miR-3162-5	hsa-miR-3177-3	hsa-miR-3177-5	hsa-miR-3180	hsa-miR-3180-3	hsa-miR-3180-5	hsa-miR-3184-3	hsa-miR-3184-5	hsa-miR-3185	hsa-miR-3187-3	hsa-miR-3187-5	hsa-miR-3188	hsa-miR-3191-3
-5.128578798	-1.273938243	-6.543751712	-5.149416037	-0.39686358	-0.665494843	-5.362448446	-4.743919032	-0.693221134	-2.356514014	-4.379573223	-5.10860214	-3.259021961	-4.616256134
-5.470812362	-1.788525605	-6.233777714	-4.506759879	-0.577181793	-0.306567372	-5.232299432	-4.654010088	-0.569859089	-1.625326799	-3.380391054	-4.740264807	-3.17527511	-4.603966436
-5.192334643	-1.284448202	-6.181518205	-5.122599402	-0.71192676	-0.629396791	-5.548657066	-4.660479667	-0.759300047	-1.527543118	-3.898951758	-4.903603356	-3.109733075	-4.06320349
-5.210194535	-1.31937916	-6.548640081	-4.978299582	-0.710892914	-0.603383238	-5.478737503	-4.517704489	-0.876361581	-1.446017357	-3.552650256	-4.703067438	-3.022980604	-4.400081769
-4.951736928	-1.507276352	-6.622446495	-4.932835559	-0.496973627	-0.497564464	-5.097481578	-4.045913589	-0.816446925	-1.729185247	-3.854452085	-4.650484047	-3.081916714	-4.220359367
-5.171431905	-1.445861361	-6.443845315	-4.401502679	-0.524178495	-0.284731774	-5.114403967	-4.911543161	-0.762002789	-1.723962625	-3.353220893	-4.740563966	-3.234140688	-4.23465902
-4.955231034	-1.411304972	-6.30585339	-4.708247526	-0.52940256	-0.466060147	-5.195229833	-4.420487542	-0.836860481	-1.617384948	-4.081159639	-4.742721547	-3.05807586	-4.331676482
-5.224805872	-1.691784272	-6.360321637	-4.439796651	-0.647358116	-0.39507763	-5.349636331	-4.916970087	-0.956948742	-1.43537253	-3.561145531	-4.771955692	-3.349274413	-4.14457502
-5.410820738	-1.562912597	-6.52447675	-4.78838999	-0.655494029	-0.429798964	-5.363937553	-4.932851648	-0.925778824	-1.517319781	-3.586536794	-4.671864496	-3.253312425	-4.301148781
-5.206536307	-1.66724456	-6.16394216	-4.259343911	-0.562501085	-0.17389304	-5.049068023	-4.817276137	-0.652249589	-1.645776035	-3.409510471	-4.626779276	-3.295731253	-4.294069835
-5.116927838	-1.747611823	-6.280716473	-4.829959859	-0.650767872	-0.284498918	-5.430377818	-4.862718508	-0.743358474	-1.416283764	-3.202084101	-4.992667941	-3.350865954	-4.304253053
-5.645136857	-1.589641742	-6.933104522	-5.381373259	-0.839480367	-0.749995263	-5.867131057	-4.905156203	-0.460028828	-1.743375905	-4.254824477	-4.771256658	-3.324551597	-4.478637735
-5.569851763	-1.82374399	-6.594245405	-5.393102871	-0.852757419	-0.790464377	-5.845724177	-5.129127217	-0.853905925	-1.346792607	-3.885562172	-4.856753467	-3.429908335	-4.40717627
-5.087424953	-1.497737545	-6.24863116	-4.897922501	-0.606950504	-0.38304991	-5.462097827	-4.92810723	-0.759379814	-1.658202441	-3.671991104	-5.179322396	-3.40240136	-4.308601353
-5.467058759	-1.811198171	-6.314217308	-4.2889374	-0.460440184	-0.219437073	-5.385915278	-4.87267861	-0.575916329	-1.735781021	-4.015733491	-4.913377358	-3.477902004	-4.449414136
-5.281711704	-1.743438715	-6.226684955	-4.586801858	-0.820395188	-0.398414218	-5.471674646	-4.974862499	-0.627301127	-1.237434535	-4.028829253	-4.914186141	-3.43511963	-4.096130499
-5.368001217	-1.64548677	-6.010640134	-4.415920827	-0.525472171	-0.231750757	-5.09748239	-5.182078492	-0.845509689	-1.618680343	-4.173676391	-4.853526216	-3.452723672	-4.217596383
-5.384065338	-2.001038714	-6.260421172	-5.51913792	-0.832796133	-0.702640378	-5.748817506	-5.495666405	-0.755672088	-2.066009622	-3.740049716	-4.959343969	-3.503929787	-4.59589028
-5.236638674	-1.59761029	-6.213533338	-5.139701867	-0.711887487	-0.6127235	-5.506431144	-4.959472797	-0.77572839	-1.780215119	-3.799544641	-5.138107855	-3.392100221	-4.55061395
-5.140493608	-1.630187502	-6.164656697	-4.741204374	-0.657590579	-0.441279041	-5.310646053	-5.134404849	-0.815898647	-1.654890023	-3.868355467	-4.914309495	-3.289501141	-4.447321184
-5.388190514	-1.721044174	-6.375541574	-4.71527921	-0.519559057	-0.241948644	-5.441020821	-5.108776852	-0.461562811	-1.770990033	-3.976632231	-4.945346781	-3.450699523	-4.270009513
-5.442090562	-1.97514736	-6.282677371	-4.33413562	-0.459628016	-0.200749572	-5.477672138	-4.844172942	-0.497519724	-1.365493311	-3.940253219	-4.463952665	-3.572989123	-4.277516219
-5.254707839	-1.730009955	-6.191075982	-5.193059372	-0.728716257	-0.613980952	-5.550573548	-4.786013491	-0.738647167	-1.490171898	-3.378276557	-4.762643775	-3.193201795	-4.501972148
-4.964050435	-1.058774696	-6.224007619	-5.324816278	-0.483998009	-0.671271718	-5.391745424	-4.331262245	-0.802899886	-1.876294523	-3.883086889	-4.751722609	-2.870930125	-4.094459187
-5.404127456	-1.766507809	-6.258386623	-4.872843566	-0.686999372	-0.335754861	-5.365951583	-5.072179462	-0.625422152	-1.435229022	-3.792165297	-4.571307546	-3.516918763	-4.234340706
-4.899200587	-0.99563123	-6.47045735	-5.48585817	-0.597642432	-0.767600828	-5.276677212	-4.728956583	-0.87774019	-1.947347864	-4.188391013	-4.873794781	-2.912119479	-4.200152102
-5.647714564	-1.876948017	-7.183938473	-5.103794198	-0.97795954	-0.545023192	-5.868565487	-5.059039916	-0.852831795	-1.502131597	-3.661955877	-4.939757491	-3.556426193	-4.710109267
-5.223834621	-1.331271846	-6.846724132	-5.733358067	-0.743292602	-0.958848459	-5.747268099	-4.599998152	-0.933995216	-2.111787432	-4.507362055	-4.886425719	-3.280295881	-4.575574338
-5.624730178	-1.648229147	-6.58327306	-5.456182612	-0.693959251	-0.633473158	-5.727758815	-4.6343767	-0.252670684	-2.853778457	-4.68846626	-5.128430221	-3.393800366	-5.188868215
-5.426506901	-1.561537938	-6.533429738	-5.13779281	-0.783643309	-0.419925536	-5.595028474	-4.713788213	-0.632311807	-1.372359527	-3.102312303	-5.058150309	-3.059995038	-4.254711889
-5.345609383	-1.778211956	-6.051264772	-4.902279947	-0.8505272	-0.49635909	-5.586786963	-4.65316857	-0.992461539	-1.301657554	-3.29227799	-4.708628446	-2.975785841	-4.594724987
-5.954339914	-2.144265701	-6.864878888	-5.541018969	-0.841435048	-1.173611824	-6.768612742	-5.20611442	-1.22816802	-3.287537433	-4.831180371	-5.123894584	-3.832227557	-5.177834236
-5.386521132	-2.357743228	-6.408121392	-4.639068594	-0.760836717	-0.208221916	-5.270415796	-5.027062586	-0.371491254	-1.675697684	-3.465171155	-4.94898447	-3.578140474	-4.308218345
-5.509295767	-2.22967903	-5.585007106	-4.613320164	-0.727681474	0.090581888	-5.347014071	-4.840698774	-0.137213972	-1.207155259	-2.96306741	-4.174266469	-3.184144191	-4.316337162
-5.45787449	-1.938186942	-6.602792632	-5.560677277	-0.861911345	-0.377356571	-5.551453292	-5.526053208	-0.497954946	-2.088388501	-4.073122512	-5.632999933	-3.688719029	-4.653968009
-5.023029385	-2.122069904	-6.468162702	-4.725742759	-0.319905393	-0.087673433	-4.946348736	-4.561473325	-0.116869076	-0.882544109	-3.875359473	-4.653109643	-3.625458209	-4.182254663
-5.138652982	-2.988695763	-7.458377002	-4.251581604	-0.727586611	0.035372456	-5.289546466	-5.846372012	-0.994841672	-1.659131999	-4.227527355	-5.886141277	-4.402956108	-4.420482648
-5.15214947	-2.687912127	-5.831371523	-5.406285212	-0.629071456	-0.100203357	-5.233587446	-5.336649634	-0.724818535	-1.780637712	-3.72881141	-5.02606794	-3.849562982	-4.337224261
-5.289504354	-2.318099701	-6.274754418	-5.289639888	-0.686066674	-0.411396671	-5.295251241	-4.780883657	-0.840499008	-1.557659673	-3.871229781	-5.060334036	-3.350855633	-4.300438189
-4.86023658	-2.601781861	-6.499240136	-4.781529729	-0.331100542	-0.111039285	-5.167528484	-5.458937021	-0.974293623	-2.373367607	-4.42781171	-5.446034961	-4.226377369	-4.435764891
-5.266083828	-2.109469306	-6.633007404	-5.151365055	-0.446959971	-0.303302282	-5.213644769	-5.044651294	-0.640296201	-2.515495978	-3.785434104	-5.132653974	-4.107047362	-4.271368114
-5.112165076	-2.942609138	-5.826612853	-4.962606195	-0.867997777	-0.220926786	-5.646951691	-5.347602818	-1.295208258	-1.56092399	-2.707572073	-5.040656814	-3.511100365	-4.855474453
-5.437069141	-2.060285675	-5.642503774	-5.270112778	-1.079900951	-0.382723866	-5.965155253	-5.785399576	-2.537120283	-0.958344806	-3.478080177	-5.383474808	-3.636545483	-4.720755636
-4.415245749	-1.215920676	-6.213604429	-5.942726372	-0.211094033	-0.543635137	-4.767420124	-4.358381443	-0.589536517	-2.580523926	-4.91354356	-4.835439403	-3.226664348	-4.280165763
-5.594639509	-2.22013916	-6.212209977	-4.519529074	-0.757237893	0.019194609	-5.545316728	-5.235161583	-1.069546999	-1.59046884	-3.921557309	-4.995993859	-3.291746466	-4.269763967
-5.383010296	-2.413863309	-6.444490421	-5.184221014	-0.738496209	-0.113090095	-5.187711722	-5.33264373	-0.883216947	-1.995543539	-3.944151351	-5.030029934	-3.647668972	-4.432894367
-5.156094521	-1.88367312	-6.971974801	-5.388157342	-0.826272627	-0.631013742	-5.239349088	-4.691357386	-0.833099355	-1.717226223	-3.390228425	-4.892117709	-3.671344354	-4.117534844

-5.314759947	-2.437653525	-6.345483486	-4.955174945	-0.760873667	-0.179368776	-5.390786578	-5.434227289	-1.117210742	-1.877699179	-3.118465944	-5.13992193	-3.235090011	-4.410965991
-4.505577207	-2.857057793	-5.605359898	-4.085665165	-0.422345439	0.287199499	-4.843017532	-5.350863975	-1.037051192	-1.501548011	-3.874539629	-4.484796759	-3.969918269	-3.98854444
-4.983204765	-2.397664871	-6.083269896	-4.534215767	-0.176628596	0.224981172	-5.198611632	-5.326412941	-0.289808724	-1.795608132	-3.758530148	-5.139247355	-3.645173267	-4.067525483
-4.315479285	-1.678183217	-6.214853419	-6.141038044	-0.098669521	-1.064316238	-5.224179523	-4.376632119	-0.825180452	-1.932854687	-4.66757767	-4.838989814	-3.360430513	-4.460254464
-5.205076198	-1.745019418	-6.29086268	-5.730844646	-0.648780513	-0.626061908	-5.219654406	-4.564824655	-1.152900376	-1.882589938	-3.924406716	-4.979332042	-3.536817431	-4.600452846
-4.641505811	-2.70602359	-5.850497377	-4.430275383	-0.587167115	-0.26524371	-5.60829291	-5.503706772	-1.060694899	-2.022411445	-3.354831109	-3.519486245	-3.543764331	-3.816636751
-3.837390597	-2.556407813	-6.001039334	-3.954794203	-0.28510482	-0.008755679	-4.629082658	-4.857101294	-0.507137184	-2.233225744	-3.782738642	-4.439910788	-3.517164586	-3.878422203
-5.15460097	-1.901355354	-6.000957311	-5.027388662	-0.651200263	-0.4857611	-5.163203463	-4.603648007	-0.980696285	-2.116878988	-4.425092303	-4.930715861	-3.641553987	-4.233424138
-4.972616575	-2.402475663	-5.607051244	-4.406500346	-0.588812053	0.287163837	-5.228457786	-5.079411769	-0.689863808	-1.53020198	-2.769012958	-4.754856117	-3.082478969	-4.203976561
-5.226869014	-2.440703663	-5.700576834	-5.03804791	-0.729746738	-0.096631435	-5.602832605	-5.152929496	-1.018143377	-1.464354482	-2.693028431	-4.662760453	-3.30055698	-4.319434209
-5.250657306	-2.798491862	-5.608050329	-5.120733377	-1.038315211	-0.376455595	-6.037111039	-5.81813372	-1.919992582	-1.936242278	-1.807993488	-5.10474847	-4.025670654	-5.210570926
-5.500080537	-2.325361303	-5.896432601	-4.693245871	-0.934712231	-0.300263857	-5.424852411	-4.957488091	-1.155639473	-1.268809411	-3.144236449	-4.719814802	-3.603796902	-4.26246971
-5.589085486	-2.45296114	-6.627708106	-5.663094319	-0.617772483	-0.646396935	-5.69684188	-5.141111307	-1.032026423	-2.493784347	-4.516488222	-5.321445798	-4.412782815	-4.734026386
-4.962841022	-2.610835405	-5.681887857	-5.470268645	-0.940664676	-0.309678595	-5.844486758	-5.502392721	-1.527800458	-1.933279129	-3.08785227	-4.951185282	-4.211856576	-4.365678729
-4.186206996	-2.730324391	-6.766930143	-4.874675648	-0.678401224	-0.33888707	-5.534799182	-5.805667757	-1.242062239	-2.316496737	-3.648831627	-5.787613237	-4.734250095	-4.482553208
-4.974192622	-2.369833641	-6.245506907	-4.664148109	-0.546920877	-0.146952162	-5.538979556	-5.524928321	-0.511016598	-2.220963316	-3.777635504	-5.228012269	-3.642892899	-4.231196862
-4.710628451	-2.533895378	-6.048940735	-4.867676253	-0.741163425	-0.318778465	-5.077352829	-5.002267787	-1.413656808	-1.994426161	-3.926647132	-5.657787786	-3.763939674	-4.463980274
-4.909858195	-1.607594381	-6.459611028	-5.871209117	-0.406682098	-0.859464715	-5.025559196	-4.464337253	-1.339953503	-2.355277685	-4.899505871	-5.183383792	-4.054860202	-4.813263558
-5.29300274	-1.641522601	-6.300078751	-5.508871206	-0.868422509	-0.376562252	-5.302354017	-4.789226245	-0.313444051	-1.550602581	-3.514350524	-4.786751339	-3.056363942	-4.21823161
-5.404501448	-2.734865012	-5.98394327	-4.855369994	-1.277214418	-0.463140677	-5.654267738	-5.618010026	-1.77270538	-1.548200045	-3.217091549	-4.793642645	-3.301183176	-4.547992652
-5.684183171	-1.603489072	-6.276007575	-4.646034896	-0.503868841	-0.320482909	-5.237757935	-4.893703682	-0.700023019	-1.53005319	-3.628453826	-4.664698535	-3.500086519	-4.216813127
-5.810601183	-2.07954631	-5.568614604	-4.747844902	-1.223869211	-0.438249262	-6.011889581	-4.875660516	-0.931141514	-1.689895085	-4.234986803	-4.578871655	-3.329058703	-3.851068401
-6.098006588	-2.3796057	-6.770777409	-5.865793454	-1.23693405	-0.989617149	-6.238301091	-5.261913925	-0.945584612	-1.57105998	-3.648812621	-4.948392742	-3.276752037	-4.750211242
-6.933043219	-2.575836779	-8.221232707	-4.556951872	-0.901125657	-0.01601137	-6.265660227	-5.681946508	-0.172999907	-1.181399127	-3.487500155	-4.696082878	-3.520164893	-4.39323621
-5.980694816	-2.050981089	-7.063309154	-5.12523996	-0.986541972	-0.389582673	-5.945569594	-5.205047785	-0.437973456	-1.523527641	-2.930241744	-4.635711923	-3.270405939	-4.762837991
-4.889948218	-2.203648699	-5.14525294	-4.367084002	-0.910085425	-0.230731967	-4.839751523	-5.277761208	-1.016858756	-1.472634174	-4.165540451	-4.397418385	-3.634438248	-3.698346838
-6.079446495	-1.825261388	-5.336992903	-4.376052981	-1.142820735	-0.444250763	-6.031686716	-4.866367893	-0.900881769	-2.131069806	-4.536739939	-4.297451547	-3.469865292	-3.343757107
-5.949953481	-1.834261773	-6.007892935	-4.532640173	-0.754188161	-0.405781143	-5.776628636	-4.758853478	-0.836191094	-2.127287866	-4.95097435	-4.340854069	-3.471635797	-3.574092951
-5.802074035	-1.8263349	-5.687541525	-4.433041403	-0.997730451	-0.437905055	-5.921272839	-4.945562371	-0.737799809	-1.642842971	-4.086327108	-4.383268069	-3.29755749	-3.844054319
-5.497873991	-2.002560793	-5.596301531	-4.715430379	-0.982218575	-0.575561761	-5.285103114	-5.102045114	-1.34583568	-1.676282081	-3.97268876	-4.474785488	-3.468602979	-3.923569584
-5.21835185	-2.045499018	-5.370793378	-4.742165078	-1.186355971	-0.562025801	-5.510848108	-5.159631159	-1.161526967	-1.682580196	-4.157027933	-4.118965353	-3.284695357	-3.275473965
-5.204706475	-1.823911822	-6.027424623	-4.440686293	-0.578232439	-0.489322181	-5.354819936	-5.122737135	-0.914177273	-2.334963245	-4.358316468	-4.216293453	-3.620665848	-3.863868292
-5.585571372	-1.702220799	-5.304037667	-4.616894858	-1.186055406	-0.495449035	-6.028602102	-5.078569965	-1.241007105	-1.858716161	-4.373980495	-4.464809924	-3.316610495	-3.357702699
-5.75299401	-1.222649065	-4.953822013	-4.30664484	-1.117186367	-0.087267888	-5.917168147	-4.863554374	-0.944716881	-1.633192244	-4.139834623	-3.898397899	-3.289676508	-3.419747612
-5.559384263	-2.388487721	-6.285206831	-5.380447672	-0.914165447	-0.473110714	-5.640934989	-5.460320344	-0.947058743	-2.172239	-3.97102787	-5.068980998	-3.798457084	-4.810853669
-4.646009188	-1.906871566	-6.054157747	-5.100339289	-0.302391066	0.082488216	-4.93123276	-4.800485721	-0.476810981	-1.381759901	-3.398021263	-4.89423475	-3.402431775	-4.337933992
-4.648904547	-2.524709731	-5.638190597	-4.694264416	-0.762014998	-0.092129591	-5.368310127	-5.300552174	-1.768856666	-1.693210253	-3.118893431	-4.665995178	-3.594680939	-4.596222375
-4.83028203	-2.557983464	-5.592414673	-4.59444613	-0.903376683	-0.049642599	-5.145354767	-5.412064196	-1.185495808	-1.803813777	-3.221660392	-5.021961197	-3.378813284	-4.187911684
-5.311014084	-2.655075504	-6.309295408	-4.604614516	-0.760962109	0.0823809	-5.203613366	-5.352768536	-0.730658021	-1.96106852	-3.683783829	-5.423272906	-3.498199802	-4.420813678
-5.039366664	-2.459101605	-5.56211583	-4.444048182	-0.756164412	-0.098679251	-4.881000021	-5.051965004	-0.676104912	-2.076704391	-4.032406848	-5.041207603	-3.116515491	-4.432426801
-5.145503953	-2.522356981	-5.45577447	-4.724057274	-0.796462872	-0.023861848	-5.023704766	-4.978142404	-1.323991152	-1.405260147	-2.662266374	-4.897750285	-3.705160095	-4.338767079
-5.609861537	-2.727469338	-5.904952141	-5.157314043	-0.923148991	-0.318400453	-5.905108857	-5.349800543	-1.555741398	-1.456017852	-1.888441315	-4.930662841	-3.562771571	-4.839476177
-5.649539435	-2.558971375	-5.992733305	-5.504070429	-0.899240236	-0.511418973	-5.882562535	-5.312355043	-2.400864313	-1.187583341	-2.265718362	-4.830607922	-3.918908145	-4.792421232
-5.585893571	-2.677108435	-6.248426624	-5.658519373	-0.820173725	-0.625640102	-5.945933385	-5.297270061	-1.30736332	-2.254664954	-4.110925265	-5.299467281	-4.262065319	-5.066919889
-5.857020293	-2.245246964	-6.447394442	-5.536197864	-0.588950544	-0.65628331	-6.002292438	-5.535028932	-0.957923948	-2.531319635	-4.076243686	-5.406279568	-4.535614902	-5.017754746
-5.41453199	-3.108706467	-6.495153029	-5.378106736	-1.035404578	-0.478023207	-5.877589101	-5.895924572	-1.910769545	-2.091467081	-3.074777786	-5.175134216	-3.70739722	-4.634635218
-5.07007876	-2.486968494	-6.644832563	-4.634260661	-0.463234857	0.341946851	-4.797348223	-5.708410058	-0.257202118	-1.733170207	-3.545307591	-4.936937701	-3.841285418	-4.016856369
-5.203241878	-2.655215237	-5.879743237	-5.707423967	-0.800095834	-0.776671401	-5.483064086	-5.466568551	-2.026976118	-1.578359227	-3.717852996	-5.187464827	-4.020827695	-4.802266716
-5.220336618	-2.568156371	-5.791238838	-3.982267146	-0.801023019	0.07717996	-5.470826911	-5.301242691	-1.53312377	-1.372239618	-3.160181394	-4.455318544	-3.71009569	-4.144567039

-5.866235776	-1.657296764	-5.109305792	-4.590383273	-0.877273028	-0.326670157	-5.794500314	-4.930983394	-0.719478309	-1.701867706	-4.137989332	-4.203051723	-3.211985327	-3.188703664
-5.775400011	-1.92493791	-4.974395976	-4.712462769	-1.230893077	-0.298121624	-6.116819792	-4.864626088	-1.117559912	-1.612978072	-3.785161159	-4.040167565	-3.100888166	-3.039050189
-5.094385404	-1.847364281	-5.715600973	-4.61687673	-1.185156596	-0.443249495	-5.665902597	-4.861922562	-1.322324233	-1.289764214	-3.997316739	-4.064197884	-2.973639672	-3.310826219
-5.630764058	-1.771381945	-5.549377883	-4.466683804	-1.105245133	-0.363358704	-5.671752624	-5.005492126	-0.921942596	-1.494997655	-4.090401036	-4.134491723	-3.187247831	-3.629999809
-5.909697349	-2.105244435	-5.737204143	-5.175547815	-1.157623033	-0.525216016	-6.032963574	-4.850341756	-1.004379799	-1.597428937	-4.210564063	-4.400765952	-3.367385408	-4.285569828

hsa-miR-3194-5	hsa-miR-3195	hsa-miR-3197	hsa-miR-320a	hsa-miR-320b	hsa-miR-324-3p	hsa-miR-326	hsa-miR-328-3p	hsa-miR-339-5p	hsa-miR-342-5p	hsa-miR-345-3p	hsa-miR-346	hsa-miR-3610	hsa-miR-3614-5
-5.079627346	-1.203137576	1.965092488	-3.640014839	-3.67989534	-5.267596077	-4.346230719	-3.947514814	-4.142528607	-6.808608888	-4.292846392	-4.882239161	-5.128854782	-3.95145856
-5.063393847	-0.951107305	1.977301044	-2.206780602	-2.463697813	-4.694405236	-3.642259468	-3.747119076	-3.644745051	-5.891211041	-3.294345353	-4.876415059	-4.832453272	-4.479463566
-5.206428769	-1.015348713	1.808290686	-2.815082432	-3.221856988	-5.115725648	-4.163408113	-4.062890685	-3.70216473	-6.551499849	-3.331291743	-4.890667209	-4.850753109	-4.137495214
-5.089739642	-1.051547384	2.032184769	-2.440916672	-2.812423032	-4.830398616	-4.021937944	-3.883314367	-3.571684601	-6.293079116	-2.949940813	-5.019381549	-4.735004905	-4.354344528
-4.848503682	-0.911267641	1.962478758	-2.326735781	-2.801857786	-4.740223387	-3.996905789	-3.682742574	-3.859109313	-6.399463262	-3.696725715	-4.63446422	-4.736054513	-3.825386147
-4.940823432	-0.783953973	2.071756792	-1.810889384	-2.193642781	-4.646123306	-3.725186834	-3.716334237	-4.03217704	-6.08652127	-3.320877128	-4.912592576	-4.708246992	-4.168933334
-4.965162851	-0.66651319	2.066203857	-2.766228342	-3.197869615	-4.84117323	-3.947707774	-3.782626606	-3.257652457	-6.19993032	-3.659269479	-4.69689631	-4.790963512	-4.058995019
-5.081666316	-0.966677247	2.381549094	-2.099563426	-2.686260236	-4.764469926	-3.669670988	-3.847555082	-3.911354702	-6.070995486	-2.690849903	-4.739708014	-4.618622108	-4.515034743
-5.250773341	-1.042443239	2.207285843	-2.493936763	-2.858134209	-4.976127522	-4.108111788	-4.083186009	-3.817705989	-6.378194663	-3.296093543	-5.035090718	-4.797284801	-4.383735941
-4.874021495	-0.866601828	1.988228826	-2.697419877	-3.029947756	-4.86188161	-3.864332875	-3.919731928	-4.294359567	-5.598722849	-3.049587908	-4.710271123	-4.697524762	-4.370836295
-4.91893639	-1.062921117	2.410498584	-1.974855256	-2.575013833	-4.640479447	-3.702914217	-4.042017013	-3.688559129	-5.499569425	-2.557639667	-5.129620573	-4.528830278	-4.172406194
-5.630978509	-1.358420907	1.406226384	-3.709481849	-4.147219415	-6.067302853	-4.83335169	-4.405672455	-5.330603327	-6.503902592	-3.992851903	-5.471907491	-5.396690726	-4.874714741
-5.449232105	-1.479453055	1.4324726	-3.289752582	-3.957245483	-5.732318587	-4.699818846	-4.627140235	-4.754964549	-6.377441176	-3.47398003	-5.512848379	-5.201677151	-4.826386912
-5.052366081	-1.045909639	1.870883984	-2.935967703	-3.523550023	-5.638435493	-4.362642199	-4.335408882	-4.503369572	-6.149209408	-3.282233766	-5.172776215	-4.949554552	-4.283850728
-4.907816473	-0.916940637	1.597142998	-2.786547399	-3.342112363	-5.130622695	-4.229363276	-4.14862521	-4.403477746	-5.711966895	-3.337066705	-5.368557035	-5.017322035	-4.621195103
-5.19503315	-1.196226561	1.980642018	-3.177148732	-3.947694909	-5.423535357	-4.438800184	-4.394097713	-4.547076943	-5.945133003	-3.290032842	-5.238479218	-4.880626908	-4.510567852
-5.112797651	-0.881363093	1.470268261	-3.49953529	-4.023369986	-5.172025099	-4.54789122	-4.533095158	-4.947061796	-5.838103225	-3.463985627	-5.127353588	-4.996275483	-4.683128962
-5.585618116	-1.154283371	1.515276075	-2.757114456	-3.273723828	-5.405095714	-4.034834226	-4.154091943	-5.239848204	-5.842907357	-3.359024412	-5.215551463	-5.177537822	-4.557046326
-5.056506588	-1.058502673	1.640028268	-2.508800897	-2.960844724	-5.160399827	-4.114986762	-4.162457295	-5.031441845	-5.842559003	-3.361679753	-5.167779844	-4.84159069	-4.38280344
-5.041113196	-0.980904675	1.843264873	-2.862775733	-3.444588814	-5.06062741	-4.166010445	-4.220096566	-4.234944022	-5.894960843	-3.27151039	-5.175367609	-4.866430125	-4.485261247
-4.92938877	-0.923418133	1.374052639	-3.002500194	-3.525406164	-4.829615853	-3.971583605	-4.110516538	-4.128672585	-6.073518996	-3.525670066	-5.415884709	-4.894382746	-4.429005414
-4.951365954	-0.797991541	1.577967151	-3.130208358	-3.862261804	-5.502711142	-4.36845286	-4.293697196	-5.351947854	-6.219053217	-3.802416334	-5.36727194	-5.153102788	-4.722492255
-5.118660499	-0.997309351	2.003119082	-2.520722737	-3.125539638	-5.028074624	-3.99807893	-4.154469146	-3.826176432	-6.136243457	-3.06926081	-5.293658211	-4.731419328	-4.330367903
-4.876762289	-0.735967782	1.950926998	-3.324595083	-3.717867142	-5.411080339	-4.422304856	-4.17027933	-4.417071859	-6.205398854	-3.424329406	-5.123417241	-4.562763154	-4.096671408
-5.048990904	-0.673918054	1.888917053	-2.877387744	-3.443032243	-5.198180661	-4.27369865	-4.287716642	-4.778883958	-5.652214079	-3.198012403	-5.161412608	-4.640068686	-4.563319151
-4.936341219	-0.686413243	1.87253712	-3.594593896	-3.948531262	-5.328241881	-4.608394675	-4.050000655	-4.418925842	-6.59586542	-3.826977963	-5.1897437	-4.682686745	-3.862057367
-5.948050049	-1.23738299	1.774901249	-2.665282054	-3.104427881	-5.555206187	-4.375425007	-4.378337361	-4.998424662	-6.396213098	-3.225560123	-5.498000126	-5.028696596	-4.828522115
-5.283272176	-1.13433583	1.582955817	-3.643191977	-4.033057445	-5.925139034	-4.759917016	-4.259770275	-4.804294637	-6.899435333	-3.82814415	-5.370675277	-5.139241825	-4.484312599
-4.987470965	-0.584283762	1.296200389	-3.25156673	-3.668507785	-5.358090734	-4.338741076	-3.888918985	-4.420376384	-5.931960658	-3.731877033	-5.004780759	-5.57993828	-4.410160648
-5.078565401	-0.870894193	2.255113986	-2.513271786	-3.0524145	-5.329961983	-4.098361532	-4.042146938	-4.05439983	-6.003671338	-2.776179143	-5.100828134	-4.557205458	-4.531885622
-4.983194312	-1.239664402	1.98040375	-2.611127216	-3.106743379	-5.2059793	-4.09913719	-4.014116824	-4.613911503	-5.865799244	-2.673808762	-5.124170308	-4.596839558	-4.69169099
-5.206625365	-1.374625479	0.176352223	-4.480188543	-5.061577492	-6.635682778	-5.346395249	-4.932824542	-5.855026358	-7.191556937	-3.388126311	-6.479414543	-5.372406995	-5.327710851
-4.787393673	-1.302619669	1.420178372	-4.131821501	-4.778259851	-6.140990772	-5.02719338	-4.806004959	-5.621933244	-5.296883334	-3.058024501	-5.062885284	-4.910846753	-5.090307594
-5.273473509	-0.93115164	1.83492343	-4.04755926	-4.525791426	-5.791118539	-5.071992725	-4.593207571	-5.434397583	-5.377239996	-2.22822974	-5.069316332	-4.721214547	-5.001625672
-5.145805502	-0.898935724	1.266659274	-4.831698573	-5.637048508	-6.358585304	-5.239611957	-5.004256067	-6.17985409	-6.033885236	-3.678664539	-5.424834297	-5.347670139	-4.650583345
-4.849245902	-0.543770637	1.631949859	-4.737010333	-5.340269658	-5.650443336	-4.687864722	-4.200755241	-5.490397216	-6.757298696	-3.674902597	-4.893381907	-5.439017603	-4.48829828
-4.806889887	-0.618862136	1.136990125	-4.689011972	-6.184247086	-5.934519288	-4.727202051	-4.497153709	-5.671915112	-6.892950268	-3.574207264	-5.971006122	-6.379708059	-5.864269695
-5.166903578	-0.575491945	1.468501136	-5.045356255	-5.600003398	-5.89449283	-4.673802108	-4.701685153	-5.857527057	-5.969391144	-2.923433893	-4.811286053	-5.122709833	-4.664487117
-5.187474489	-0.974578767	1.341761193	-4.385356296	-5.004402036	-5.787448543	-4.738732015	-4.522141417	-5.464290119	-6.370580688	-3.643908081	-5.005591662	-5.424024705	-4.481292292
-5.024649577	-0.613940466	1.225876744	-4.048677218	-5.006558062	-6.028162197	-4.976344797	-4.448387255	-5.826973738	-5.080795214	-3.634493048	-4.721841977	-5.25460409	-4.64722643
-4.90271761	-0.672843205	1.750784706	-4.817296778	-5.257840349	-5.960558886	-4.703074863	-4.44320342	-5.214386891	-6.238027471	-3.923200379	-5.049643821	-5.057086656	-4.414185328
-5.398446389	-0.996799893	1.308723928	-3.559064238	-4.139600734	-5.957241559	-4.464390455	-4.483057795	-4.945940707	-5.758341629	-2.433531052	-5.071889629	-5.286629475	-5.05698208
-4.434876351	-1.067148491	0.718693514	-4.740742855	-5.453780028	-6.514760579	-5.088135208	-4.932790106	-6.668457631	-5.849744392	-3.309394931	-5.524310253	-5.574899708	-5.352557085
-4.531942912	-0.995789413	1.35995176	-5.494647244	-5.923460799	-5.462020497	-4.789206635	-4.13082605	-6.039616896	-6.864759729	-4.331587063	-4.886897897	-4.380282255	-3.101392905
-5.285861104	-0.874625043	1.416610135	-4.187848223	-4.924404778	-5.744800662	-4.855241132	-4.448531684	-5.786077857	-6.377426658	-3.590980935	-5.165294421	-5.575149711	-4.886453499
-5.111732299	-0.65121445	1.701330491	-5.136227151	-5.717637716	-5.838371323	-5.010050598	-4.614704864	-5.941231489	-6.428079525	-3.281485607	-5.17214111	-5.408871651	-4.764078527
-5.096831292	-1.075839177	1.738601947	-4.343860986	-5.018064693	-5.710376461	-4.788217172	-4.445248074	-5.506412027	-6.919757112	-3.620607592	-5.004047165	-5.256959217	-4.269644313

-5.158939828	-0.987075377	1.732924121	-3.480649494	-3.960974426	-5.645937834	-4.410879329	-4.633467592	-5.343108527	-5.833819957	-2.803060863	-5.22176968	-4.918751305	-4.792620175
-4.372659769	-0.974389037	1.603679624	-4.15102026	-4.895181597	-5.290626004	-4.770513823	-4.490287849	-5.911782173	-5.593086164	-3.406090327	-5.03851275	-4.899712879	-4.838142544
-4.452621751	-0.785289286	1.656793886	-3.945907707	-4.752184109	-5.657275389	-4.756354736	-4.43346151	-5.641833948	-5.620898737	-3.448281358	-4.872362162	-5.129556277	-4.654067132
-4.954182813	-1.365521065	1.328270891	-5.485161824	-6.278426901	-5.886848105	-4.884586259	-4.174049442	-6.185580455	-6.945131108	-4.184459832	-5.018302293	-4.834285382	-3.536482561
-4.730257543	-0.887647639	1.292303595	-5.495910534	-6.145626262	-5.739625884	-5.228848363	-4.368076953	-5.992565149	-6.808051202	-3.591397535	-5.099237548	-4.953360805	-3.980873254
-4.698321345	-1.32979242	1.169228672	-4.376967252	-4.964421076	-5.989822299	-5.166132063	-4.791762495	-6.95282432	-4.522904526	-3.167758899	-5.381707609	-5.020531904	-5.006337811
-4.382349814	-1.032721847	2.146767016	-4.593462277	-5.216939084	-6.056199921	-4.639156267	-4.507337186	-5.484932617	-4.061741176	-4.15960896	-5.422773965	-5.082549725	-5.708296879
-4.35585351	-0.266040458	1.202831778	-4.467571726	-5.179720689	-5.555579186	-4.847021276	-4.42277157	-5.460495292	-6.092546945	-2.963777186	-4.964475583	-4.731175873	-4.021928139
-4.922117413	-0.464812444	1.665043649	-4.050485997	-4.351945179	-5.599356876	-4.80060368	-4.513110469	-5.405101733	-5.744914101	-2.964567667	-5.16316653	-5.14988558	-4.819563059
-5.189007698	-0.831353295	1.562773601	-4.914050821	-5.32720717	-6.013123076	-4.874123566	-4.789481434	-5.817957684	-5.934178594	-2.580055606	-5.227066386	-5.012393491	-4.906701951
-4.987398851	-0.944683365	0.888347299	-3.644081115	-4.571192897	-5.899013835	-4.740189831	-4.54773152	-6.291108669	-5.018824091	-3.055156111	-5.279775619	-5.252410371	-4.966090449
-5.118250425	-0.579779274	1.490473296	-4.028512174	-4.826404754	-5.779178695	-4.48501897	-4.443107696	-5.634875142	-5.471425656	-2.020729897	-4.92184784	-4.410448243	-4.85712116
-5.135578774	-0.809085433	1.047129918	-5.757500693	-6.775752166	-6.254668395	-5.521416288	-5.062379016	-6.738410097	-6.865443447	-4.254983114	-5.157277319	-5.818980181	-4.610860226
-5.438035503	-0.888426074	1.006205737	-4.727439354	-5.947668571	-6.172398582	-5.208883824	-5.130328431	-6.679955449	-5.978108136	-3.384431659	-5.170483673	-5.545469168	-5.11496343
-5.162460285	-1.270854269	0.977823504	-5.287841808	-6.6211738	-6.062383402	-5.294978621	-5.669271012	-6.414908083	-5.329303752	-3.782482813	-6.140657794	-6.531627608	-5.428476299
-4.862566416	-0.836398394	1.936074165	-3.76995876	-4.633850055	-5.90596026	-5.16514026	-4.134768604	-5.54735914	-5.513653962	-3.51546363	-5.247515086	-5.124413667	-5.473232988
-5.202614085	-0.915657794	1.621514671	-3.459229913	-4.329899085	-5.440539817	-4.186850453	-4.266066791	-4.879790659	-5.663953131	-2.966410955	-4.858662461	-5.058271194	-4.889064355
-5.075973332	-0.811505781	0.866054263	-5.837210719	-6.492932496	-6.042994138	-4.967179414	-4.230672261	-6.447656254	-6.583913711	-4.142169468	-4.541233331	-5.465054423	-3.985607402
-4.765583762	-0.851403988	1.455661708	-4.892816442	-5.604238926	-5.962812468	-4.959886705	-4.445414815	-5.828749858	-6.357752316	-3.256622491	-5.065792177	-5.20192125	-4.544427545
-5.371089686	-1.064321222	1.171577689	-4.029142447	-4.49021596	-6.162786218	-4.663096569	-4.923675706	-6.065999318	-6.013687632	-2.318291596	-5.65903655	-4.596665334	-5.217591892
-5.051155727	-0.742343262	1.782792378	-4.438781227	-4.572331538	-5.868711591	-4.942334698	-4.372906718	-6.200927135	-6.299821863	-3.121641454	-5.022964426	-4.763141333	-4.536031782
-4.897233052	-1.638499271	0.994599939	-2.95816255	-3.620024525	-4.817592647	-4.706253379	-4.397640363	-4.86469116	-5.061961017	-3.673695202	-5.575849345	-5.08402179	-5.049852103
-5.438477707	-1.878314296	0.9694565	-4.401011523	-5.029731182	-6.366403777	-5.131701281	-4.907428411	-5.358497059	-6.782798969	-3.472440008	-5.859662892	-5.354683779	-5.477171695
-6.078774928	-1.592000722	1.306349665	-4.043824749	-4.831178438	-8.034668636	-5.637327051	-5.296641016	-7.190178601	-5.207549864	-2.547446001	-6.867404051	-5.51067704	-6.231429909
-5.377935964	-1.622770507	1.516431201	-2.914175588	-3.555290971	-6.426433625	-4.629702794	-4.769851222	-5.54617614	-6.397152404	-2.8587326	-5.834494101	-5.038469711	-5.225199721
-4.899640869	-0.951356807	1.517999986	-3.243621665	-4.090011951	-5.389769216	-4.751670183	-4.89567036	-5.303095278	-5.069972269	-3.863781461	-5.679829709	-4.72916268	-5.006087948
-4.890341773	-1.630840367	0.31988503	-3.759947242	-4.326238207	-5.109134043	-4.997954826	-4.800380751	-7.136327461	-4.500347238	-4.137168601	-5.517550358	-4.931648257	-5.105248037
-5.094723347	-1.211042324	0.607126783	-4.750559975	-4.963289232	-5.239683468	-5.45127731	-4.666604046	-6.696496023	-5.400673356	-4.324515137	-5.458623658	-4.74490252	-5.079263113
-5.062732106	-1.489426143	0.983170431	-3.400251425	-4.05666898	-5.061234144	-4.839032573	-4.447479518	-5.285618157	-5.220299393	-3.973707491	-5.331782801	-5.218726421	-5.081475071
-5.119459317	-0.913412881	1.539487097	-2.573818008	-3.214567437	-4.930024212	-4.34125826	-4.239732466	-4.561229392	-5.693864385	-3.530057827	-5.24537032	-4.765945209	-4.828119386
-4.96823875	-1.853340122	0.756856905	-3.634892415	-4.272686721	-5.851189499	-5.413877823	-4.801815416	-6.776386352	-4.372975576	-3.996935945	-5.604055078	-4.684487684	-5.288298131
-4.919125537	-1.26027461	1.028152754	-3.328640388	-3.691060023	-5.301982836	-4.939473625	-4.571967225	-5.871006289	-5.201062578	-4.123162453	-5.486591562	-4.709331598	-5.22783817
-4.860292246	-1.823286968	0.737952453	-3.292679082	-3.787220866	-4.769303138	-4.627970722	-4.319825134	-4.67743756	-4.58499491	-4.00727093	-5.305897112	-5.003210263	-5.484331063
-4.763880204	-1.972425257	0.64180138	-3.563590147	-4.067330272	-5.047188158	-5.122741897	-4.606283355	-5.81294485	-4.343704367	-3.959150506	-5.610208082	-5.160298057	-5.249401936
-5.378072766	-1.177071415	1.406595195	-4.529136957	-5.253222319	-6.325520502	-5.444004168	-4.801765392	-6.158615052	-5.912654283	-2.323508225	-5.177168009	-4.797624275	-4.81319343
-5.259450287	-0.476925031	1.713351032	-4.551548664	-5.028381492	-5.794946227	-4.99394128	-4.452848056	-5.677366142	-6.246375194	-3.437770958	-4.887479781	-5.280095513	-4.628730317
-5.336957864	-0.81285773	1.215694798	-4.012897895	-4.507640441	-5.674258717	-4.641531375	-4.592332245	-5.321073518	-4.80287479	-2.349342115	-4.848265508	-4.544351196	-5.426359871
-5.330257105	-0.77110258	1.718701834	-3.200095952	-3.811421667	-5.35205734	-4.401651164	-4.632598225	-5.269448005	-5.363264583	-2.52328149	-5.131726104	-4.571067409	-4.66478262
-5.042614813	-0.659956602	1.59787965	-4.752245709	-5.572563692	-6.221033941	-4.926272677	-4.627462478	-5.997320258	-5.878090468	-3.48791918	-4.90428862	-5.369259523	-4.904767662
-4.330686794	0.047624231	1.729599738	-4.15275468	-4.750173482	-5.25651693	-5.247203068	-4.598537249	-5.35663436	-4.921683258	-3.6391058	-5.032203562	-4.527144033	-4.17180147
-4.932378601	-0.655051463	1.68037587	-3.063880622	-3.826305516	-5.359679772	-4.19464485	-4.394979043	-5.166213425	-5.372324138	-2.348942144	-4.952794309	-4.399462489	-4.89168287
-5.461436892	-0.855495818	1.437460698	-3.996064536	-4.722809598	-5.815067792	-4.723149313	-4.699914342	-6.141257385	-5.96307643	-1.930173856	-5.385741357	-4.602226035	-5.148346
-5.149992064	-0.565553362	0.579461041	-4.484330323	-5.423560067	-6.027042995	-5.049777885	-5.049058586	-6.464658109	-6.761800286	-3.854742621	-5.503782987	-5.620683876	-5.234035713
-4.863544505	-0.668532789	0.579690279	-6.004998145	-6.694036351	-6.034905228	-5.72618595	-5.086923117	-6.883510314	-6.033037381	-3.786738551	-5.772440856	-5.581623528	-4.881310789
-5.009863728	-1.193516846	0.839380824	-5.980400347	-6.546909654	-6.764365279	-5.726263427	-5.227248056	-6.936406711	-6.615238066	-3.687156339	-5.810434296	-5.759970857	-5.176779453
-5.397742822	-0.646597614	1.340582905	-3.925778155	-4.623709526	-6.176659771	-4.444979461	-4.651468097	-5.696632868	-5.911635416	-2.519635176	-5.504576083	-5.070767171	-5.316199323
-5.08743515	-0.696151699	1.458108705	-3.654077414	-4.884629388	-5.194676845	-4.759668892	-4.841753223	-5.66955182	-5.155278563	-3.491709383	-5.277767401	-4.862526932	-5.064699361
-5.542851216	-0.729035455	0.862080961	-5.026341611	-5.87084506	-6.359534115	-4.854350808	-4.617318195	-6.170010541	-6.18442659	-2.409949661	-5.042496603	-5.081293354	-5.062089847
-4.920271233	-0.750702616	1.811564825	-3.101646863	-3.810500304	-5.265444995	-4.739692452	-4.573982592	-5.773071078	-5.315036508	-1.928318575	-5.224506316	-4.059507424	-5.355990731

-4.783031123	-1.535064033	0.96218977	-3.347484506	-3.81184225	-5.019650941	-4.59073235	-4.210658203	-5.381124184	-4.553867988	-3.946583771	-5.212130444	-4.697028094	-5.186164221
-4.886953733	-1.861197451	1.14649002	-2.672402926	-3.125930913	-4.825600587	-4.827082642	-4.34967391	-5.245022568	-4.076349167	-3.434404474	-5.072924164	-4.674840155	-5.26423401
-4.821843831	-1.59618156	1.000974025	-2.837958222	-3.343293038	-4.750191404	-4.527680841	-4.319637987	-5.368345932	-4.624161707	-3.661265339	-5.644353816	-4.960289612	-5.319068854
-5.247470098	-1.524797792	1.067950342	-3.013114479	-3.474931444	-5.344351981	-4.878363122	-4.470714986	-5.484050742	-4.705129869	-3.907084939	-5.27129174	-5.034926498	-5.331046353
-5.11541293	-1.363921832	1.154543654	-2.762393861	-3.471324154	-4.885695996	-4.621827963	-4.226154155	-4.872468294	-5.411331366	-3.812342879	-5.407442564	-5.046915931	-5.121133748

hsa-miR-3616-3	hsa-miR-3619-3	hsa-miR-3620-3	hsa-miR-3620-5	hsa-miR-3621	hsa-miR-3622a-	hsa-miR-3622b-	hsa-miR-3652	hsa-miR-3656	hsa-miR-365a-5	hsa-miR-3663-3	hsa-miR-3663-5	hsa-miR-3675-3	hsa-miR-3679-3
-3.854136128	-1.192556175	-4.546530143	-1.289140111	1.296151802	-4.660172651	-4.339293044	-3.835160961	1.724158228	-3.605866346	1.339656637	-4.062480481	-3.911736892	-2.943706579
-4.110075147	-2.409878146	-4.767438267	-1.335725671	1.517268213	-4.548760445	-4.549062653	-4.086052436	2.015625656	-2.957569822	1.974755777	-4.037031028	-4.269079522	-3.358820661
-3.755399423	-2.43342436	-4.561132899	-1.315760347	1.470270059	-4.232920161	-4.606846429	-3.212897979	1.947186433	-2.820343302	1.376598423	-4.207050342	-4.076380609	-3.095020589
-3.650971595	-2.989482149	-4.708918996	-1.385141615	1.315089732	-4.35435742	-4.333235031	-3.663416573	1.884139611	-2.284660114	1.357110223	-4.076988032	-3.961803807	-3.006287169
-4.006606822	-2.039685666	-4.284506304	-1.2472113	1.637282134	-4.18756033	-4.351167517	-3.871005873	1.919803824	-2.993142168	1.626057107	-4.062424113	-3.589972547	-2.605783592
-3.970375602	-2.424005033	-4.465001526	-1.420986697	1.694318135	-4.304486906	-4.422875071	-3.958470551	2.070919895	-2.922858444	2.118006965	-3.95538962	-3.90145905	-2.826694614
-3.707459552	-2.426018215	-4.463259862	-1.256207829	1.473419686	-4.18636792	-4.226542883	-4.275552011	1.888487842	-2.494625144	1.514041484	-4.020250963	-3.644595771	-2.628814646
-3.616339334	-3.091678145	-4.545232188	-1.430391824	1.45318595	-4.288005462	-4.569555582	-3.619821445	2.002572412	-2.400007471	1.961417936	-4.110811227	-4.314037035	-3.207545159
-4.125563431	-2.875614664	-4.748005682	-1.505139611	1.270751147	-4.480754779	-4.446359376	-3.913174172	1.911675251	-2.814817442	1.59345171	-4.097437236	-4.179085377	-3.141281825
-3.834048536	-2.857014376	-4.52147797	-1.541727781	1.571634399	-4.193843547	-4.329372482	-4.033423332	1.863289687	-2.841549507	2.292873637	-3.992257053	-4.072689619	-3.062815464
-3.556702523	-3.137715629	-4.920831294	-1.498212286	1.362235277	-4.286848323	-4.522550863	-3.903475964	1.952155002	-2.155744311	1.729621854	-4.469833542	-4.00426351	-3.126128743
-3.939530037	-2.373154063	-4.958710808	-1.675128955	1.344479315	-4.666700437	-4.835461533	-3.714072911	1.472805466	-3.454063318	1.480553315	-4.574501577	-4.393772281	-3.515087799
-3.662798864	-2.529016968	-5.143579239	-1.706002137	1.195269969	-4.466073717	-4.636384615	-3.508831867	1.723157071	-2.854768093	1.105883176	-4.687584811	-4.501212422	-3.523322871
-3.687352134	-3.183841359	-4.977570403	-1.558086835	1.4752408	-4.214823002	-4.910241948	-3.770685465	1.813332619	-2.573697637	1.747413423	-4.805874634	-4.099688236	-3.167485886
-3.682865335	-2.787585152	-5.098938798	-1.500734998	1.798943485	-4.399258294	-5.134076492	-4.294546887	1.950836644	-2.672086241	2.156058681	-4.773960311	-4.318985121	-3.399868683
-3.726853827	-2.847048517	-5.022554813	-1.429789475	1.47488126	-4.481003552	-4.94295883	-3.475962482	1.960368807	-2.636265067	1.556846832	-4.673374432	-4.429259158	-3.479592295
-3.761782806	-2.418518263	-5.042698948	-1.545519607	1.774516736	-4.372952938	-5.175825458	-4.059224512	1.872067504	-3.22436105	2.04494949	-4.539144574	-4.329335773	-3.389653216
-3.709632524	-2.902229641	-5.734751281	-1.771511077	1.19165185	-4.579235129	-5.054100816	-3.740083586	1.566739834	-2.802776831	1.706110777	-4.806165603	-4.662130643	-3.508442344
-3.770090091	-2.108905491	-5.182222422	-1.611732169	1.41034557	-4.453961324	-4.565118632	-3.911224915	1.627783344	-2.601699677	1.573202819	-4.738804036	-4.168519279	-3.246654124
-3.630062306	-2.827987583	-5.13696655	-1.650440064	1.502150247	-4.323228389	-4.808254507	-3.878537038	1.725838634	-2.505893577	1.754756164	-4.626417851	-4.253775735	-3.260244599
-3.734692887	-2.283676142	-5.144050502	-1.402454523	1.81056995	-4.216636328	-5.242577629	-3.981777913	1.923477396	-2.832552376	2.176249244	-4.747471655	-4.325833477	-3.375955662
-3.823290219	-2.774418187	-5.013540315	-1.390255871	1.758948804	-4.360901527	-5.344832602	-4.041906484	1.99304235	-2.806021369	2.121674515	-4.754103231	-4.397772257	-3.349123605
-3.676735672	-3.074756782	-4.99399242	-1.539006985	1.225673207	-4.277453333	-4.737975348	-3.648714755	1.809936701	-2.39307278	1.531356262	-4.817761324	-4.060857809	-3.113963576
-3.415089271	-1.5884466	-4.863593161	-1.422798025	1.616395652	-4.000115547	-4.539318708	-3.859408109	1.566346468	-3.074820838	1.423681621	-4.865784553	-3.647867466	-2.754908403
-4.003811583	-3.063069522	-5.094548683	-1.494055255	1.684577734	-4.272642952	-5.110157962	-3.904595191	1.981154885	-2.369090638	2.075396733	-4.904862286	-4.359781507	-3.254740958
-3.444989926	-1.815530288	-4.880625058	-1.714107276	1.616825151	-3.933462973	-4.636929184	-3.825406876	1.698527992	-3.387047381	1.438754412	-4.56689755	-3.59576628	-2.548403923
-3.774559932	-3.107428721	-4.988699654	-1.72774303	1.367684538	-4.429359456	-4.772545452	-3.850428529	1.673711189	-2.690366064	1.510735971	-4.73079544	-4.596626079	-3.493641477
-3.410765061	-2.702251765	-5.265909618	-1.776675583	1.445087523	-4.264950574	-4.853495393	-3.869485075	1.303312322	-3.285776193	1.334186394	-4.652245211	-3.992359496	-3.021254259
-4.208357402	-1.694110635	-5.213015471	-1.401434396	1.719016771	-5.113325894	-4.950516369	-3.934320311	1.387663145	-3.056937328	2.004630526	-4.226527588	-4.151336672	-3.240106793
-3.878922622	-2.861405031	-4.670201844	-1.535928312	1.580595532	-4.124123858	-4.480090204	-3.730146222	1.87107844	-2.641208935	1.71150167	-4.415674473	-4.201717052	-3.206489535
-3.481132248	-2.982608175	-4.843779415	-1.534824022	1.203280422	-4.169096273	-4.233917135	-4.093834123	1.796428731	-2.158184886	1.39604169	-4.360990687	-4.228727172	-3.25859295
-4.242385068	-3.445352438	-5.787096121	-2.105423988	1.49348665	-5.267289117	-5.301991844	-4.645851314	0.737610814	-2.852191704	1.650585992	-5.346672085	-4.782223682	-3.739122332
-3.951237228	-2.491299943	-5.161586691	-1.299621792	1.486486384	-4.423950032	-4.906231672	-3.818575356	1.834982332	-2.68502434	2.114547234	-4.4749569	-4.784226687	-3.783064951
-3.637739158	-2.881049915	-5.038593492	-0.875240037	1.80765148	-3.986314686	-5.081881069	-3.863828361	2.434520619	-3.282424691	2.374822899	-4.467646864	-4.756257751	-3.73248169
-4.100498341	-3.594620425	-5.587079023	-1.641830603	1.446816105	-4.616581216	-5.879705943	-3.835615182	1.698322944	-2.752505126	1.597702336	-5.372577442	-4.642085479	-3.552922222
-4.452572971	-2.776075065	-4.756643567	-0.959670368	2.189446492	-4.423326897	-5.79086764	-3.896418619	2.655162919	-3.513992748	2.415419921	-4.418722975	-4.165479519	-3.164196414
-4.378467429	-2.176203459	-6.262072685	-1.302059023	2.190104092	-4.71477382	-7.324864965	-3.854124179	2.282661377	-2.476223678	2.551139826	-6.251017629	-5.50012632	-3.912711251
-3.582150582	-2.865344793	-4.988173188	-1.561037314	1.556490137	-4.064965886	-5.71619978	-3.571640614	2.084328351	-2.793456516	2.429527681	-4.765322348	-4.932398039	-3.48809794
-3.739989695	-3.497044882	-4.910677225	-1.393419124	1.518948802	-4.331881496	-5.4672657	-3.346753145	2.263038088	-2.567168077	1.815682309	-4.888712374	-4.25725017	-3.231313469
-4.276193226	-2.30142476	-5.36739192	-1.555906036	2.010785524	-4.049127937	-5.791670063	-4.119846053	2.103934909	-3.952074457	2.865741832	-4.886450934	-4.362379144	-3.409072375
-4.402537041	-1.797565404	-5.023088562	-1.32187952	2.03106366	-4.426151763	-5.644564638	-4.281091029	2.103620873	-2.990180313	2.253024504	-4.821454884	-4.549018392	-3.268815104
-3.580225426	-3.204237533	-5.316074934	-1.389355992	1.67973098	-3.798351328	-5.198400879	-3.646506051	2.354623036	-1.919822287	2.144215501	-4.835274213	-4.860482805	-3.949287944
-3.511795627	-3.334731074	-5.456755471	-1.6790787	1.498689968	-3.766660025	-5.590287269	-3.076172714	2.097019197	-1.947407023	1.798828412	-5.023389598	-5.127488314	-3.731098896
-3.550218382	0.481928256	-4.716884727	-0.887171039	1.553276942	-4.601893908	-4.589582959	-3.857650411	1.890020892	-4.422136744	1.308170082	-4.723847621	-3.048356173	-1.941310124
-4.447333698	-3.132206527	-5.391596253	-1.430468827	1.767277658	-3.860024905	-6.002618172	-3.811153027	2.443152315	-2.69507088	2.388046188	-5.167320175	-4.803087596	-3.596995684
-3.881933046	-3.351756075	-5.257767413	-1.529576506	1.730397654	-4.252672024	-5.896039389	-3.535737696	2.005590931	-2.369316184	2.259724349	-4.978395228	-4.798406098	-3.662444255
-3.113074687	-2.522957994	-4.838988782	-1.228280505	1.483798723	-4.160362952	-5.219850241	-3.122699966	1.956829514	-2.727725311	1.61409982	-4.778266945	-4.115249177	-2.970402162

-3.670252303	-3.372005963	-5.322462673	-1.451648203	1.42598534	-4.175045744	-5.408275302	-3.71897014	2.093091279	-2.347378648	2.004451866	-4.803089227	-4.727400313	-3.5890989
-3.650727634	-2.037799614	-5.699899098	-1.042889794	2.155071523	-3.557876325	-5.437811298	-3.447486736	2.563531432	-2.663988091	2.779969862	-4.867162898	-4.877255445	-3.760443996
-3.759098045	-2.621718609	-5.111226741	-1.152291541	1.897250492	-4.136726015	-5.418042676	-3.709469769	2.328853683	-2.890290705	2.718304331	-4.51859221	-4.341395742	-3.228541377
-4.074541486	0.010759332	-4.810617466	-1.130042962	1.071619762	-4.495735739	-4.365568498	-3.580086104	1.821747493	-4.159066049	0.958994712	-4.748382069	-3.313334983	-2.180538837
-3.813346457	-1.49597653	-5.079649178	-1.552552532	1.837470356	-4.030858778	-5.123481349	-3.799359051	1.948326319	-3.019401731	1.539235348	-4.837151005	-3.811809432	-2.724346794
-4.061915907	-1.74608302	-5.712627702	-1.489952574	1.77158515	-3.563401464	-4.793738031	-3.395748175	1.90820684	-2.597994428	2.39760429	-4.775632164	-4.921386902	-3.686815435
-4.267146001	-1.250582024	-4.969729086	-1.144371937	2.041971393	-3.272355394	-4.859516216	-3.648577024	2.246365904	-2.64875736	3.016720661	-4.253553331	-4.463505746	-3.391319652
-3.782084181	-2.522812628	-5.055650279	-1.611565811	2.258889913	-4.568450709	-5.067470794	-4.201455885	1.91004947	-2.903058229	2.191731895	-4.786189274	-3.789178107	-2.847162325
-3.303211399	-2.65226057	-5.096818165	-1.316127053	1.892433291	-3.982206143	-5.584148483	-3.776461699	2.458947334	-2.937065982	2.623412011	-4.805011566	-4.501900012	-3.380651277
-3.425131484	-3.438603245	-5.170682828	-1.41680177	1.578342347	-3.618926958	-5.426803723	-3.246442261	2.321916899	-2.579002786	2.125644501	-4.74337147	-4.674074465	-3.514633106
-2.704699026	-2.427688955	-5.386332133	-1.869833281	1.655118944	-3.598868676	-5.679463999	-3.153049503	1.833290278	-1.538849854	2.082365617	-5.009169921	-5.325407896	-4.106096452
-3.111450104	-3.237652575	-4.957847196	-1.520658988	1.616527277	-3.839870354	-5.239116835	-3.219703448	2.152919262	-2.041046094	1.998716414	-4.697450963	-4.613746287	-3.738326675
-4.354121486	-2.774394143	-5.511176806	-1.759649661	1.720993406	-4.47575793	-5.814312529	-3.860919487	1.768111151	-3.861927482	1.762263907	-5.074975185	-4.575064409	-3.580000224
-3.007197442	-2.870504298	-5.571760484	-1.815647539	1.54490716	-3.767161677	-5.848669358	-3.118535452	1.960984233	-2.713044301	2.015216418	-5.133428744	-5.102548489	-3.818903986
-4.099367098	-2.154205257	-6.429045462	-2.00627638	1.499744147	-4.098304894	-6.907748873	-3.561625168	1.690482144	-2.572948469	2.35457733	-5.991651569	-5.213735643	-4.021758954
-4.010347467	-2.295931745	-5.892685529	-1.563504975	1.838927388	-3.874882446	-5.819519221	-3.760098024	1.990276191	-2.788262072	2.729338022	-5.284005109	-4.794327834	-3.672677117
-3.408860564	-3.175097923	-5.347751254	-1.446454068	1.530976235	-4.167744785	-6.005938134	-3.740032318	2.083094155	-2.902973922	2.259481125	-4.876620985	-4.744288592	-3.682268165
-4.669093459	-1.245712309	-4.877013459	-1.400711958	1.621594196	-4.827744985	-4.963158573	-3.77682125	1.867669705	-3.903339146	1.756090825	-4.159030082	-3.961340222	-2.778126045
-4.211514103	-2.639707036	-4.909312561	-1.356336282	1.806948975	-3.863924084	-5.324546741	-2.897641928	2.013942362	-3.211497424	1.692709831	-4.820402275	-4.211658407	-3.229951107
-3.179031308	-3.209650107	-5.424362064	-1.603959089	1.482348804	-3.785163151	-5.386719653	-3.717605674	2.036407047	-1.702035753	1.913844562	-5.269888927	-5.159972357	-4.047899478
-4.050132376	-2.413577185	-4.913774062	-1.375510153	1.711925529	-4.046964312	-4.974284492	-3.901312048	2.223524974	-3.15636597	2.023495738	-4.484741365	-4.222724175	-3.153498067
-4.008840242	-2.194131057	-5.055047624	-1.546107786	1.364924368	-3.955904599	-4.150719565	-3.422811567	1.67865488	-2.028903993	1.349575924	-4.320456411	-5.112027028	-4.243862955
-3.614606123	-3.593823344	-5.669208428	-1.796430282	1.072036586	-4.517699431	-5.202385337	-3.678005631	1.37661185	-2.191547038	0.772211164	-4.986966467	-4.916621405	-3.934180599
-3.882717209	-2.180923583	-5.993331637	-1.49182493	1.57891866	-4.373031154	-5.594264989	-3.862113754	2.088869715	-2.800765842	2.235552894	-5.151564026	-5.765968862	-4.467164995
-3.870976665	-3.046284328	-5.213515212	-1.571931797	1.553468839	-4.394593055	-5.038920695	-3.92369579	1.754988211	-2.351446894	1.698995346	-4.832134141	-4.841716789	-3.803708885
-4.345971016	-2.342759383	-5.344867516	-1.652172736	1.435322442	-3.88236145	-4.898971799	-3.679347505	1.839456105	-2.221174927	1.900751137	-4.590421751	-4.711336869	-3.780421283
-4.619210221	-1.170363807	-4.839795368	-1.534652967	1.981909739	-3.813017819	-3.89344513	-3.168269951	1.812157646	-2.665347246	1.950487015	-4.371577336	-4.706436797	-4.282391621
-5.005527278	-1.663291259	-4.870774445	-1.54747823	2.173088386	-4.195623232	-4.181583763	-3.817145047	2.024540816	-3.408370103	2.00355974	-4.220403357	-4.544832078	-3.93218412
-4.38526387	-2.172546185	-5.184003606	-1.531665412	1.568533796	-3.993931969	-4.309364947	-3.384301538	1.725067393	-2.31030495	1.588490219	-4.534806012	-4.827208681	-3.916020075
-4.330193702	-2.558622806	-5.247629467	-1.758786519	1.374072343	-3.932642377	-4.655875629	-3.771789684	1.767322261	-1.79759917	1.764056785	-4.555377214	-4.637672022	-3.53490704
-4.438760419	-1.604974149	-5.222158317	-1.536546862	1.310600515	-3.762785837	-4.047689002	-3.195002194	1.721595702	-2.354207519	1.512301953	-4.400130186	-4.861351818	-3.972843205
-4.75989097	-1.221392206	-5.029433838	-1.628117286	1.841784741	-4.079467632	-4.434689376	-3.894780005	1.657851248	-2.634754018	2.285559229	-4.742818771	-4.570832082	-3.490230004
-3.785489734	-1.674934834	-5.042623186	-1.690372762	1.424349307	-3.749253519	-3.952218574	-3.200778003	1.603616753	-1.940958099	1.426744067	-4.349054227	-5.129128014	-4.139839106
-4.332385888	-0.463332493	-4.976881465	-1.353994002	1.507650726	-3.8965108	-3.671060081	-3.211917685	1.689322551	-2.37992239	1.618955093	-4.38957252	-4.785449164	-3.972087146
-3.37455969	-3.167352584	-5.377382729	-1.693171273	1.425073485	-4.189469242	-5.424036574	-3.599902937	1.407320648	-2.706622445	1.70755906	-5.038359425	-4.954898091	-3.684021248
-4.122920642	-2.67998126	-4.879546673	-1.234676518	1.910264709	-4.270160742	-5.750719663	-3.598500855	2.527495254	-3.066295259	2.441834567	-4.807573738	-4.264009206	-3.255794767
-3.495016157	-2.402388505	-4.979656242	-1.68730954	1.574931809	-3.597184356	-5.369510166	-3.419495665	2.063380072	-1.827647603	2.451034444	-4.830102469	-4.626748236	-3.838422271
-3.941193937	-2.513953621	-5.270988843	-1.622633486	1.596028784	-3.841442936	-5.061798046	-3.239749461	2.21535644	-2.216115314	2.496459481	-4.990023068	-4.799803281	-3.57962935
-4.458990468	-3.197845419	-4.934723362	-1.46338532	1.693830987	-3.911238189	-5.628350135	-3.996238182	2.197902597	-2.923029987	2.425302317	-5.083599187	-4.964338655	-3.594021487
-4.517886377	-1.910578544	-5.413302363	-1.519406552	2.436355971	-3.915089862	-5.375805164	-3.961924567	2.207471812	-3.246538606	2.967359927	-5.072393741	-4.295323129	-3.102962444
-3.182936992	-2.998069839	-5.02009042	-1.550264749	1.517448877	-3.816181703	-5.376329057	-3.510474522	2.214147963	-2.244139832	2.232018936	-4.754924252	-4.690747004	-3.599791973
-3.137073605	-3.473378455	-5.396423212	-1.600121811	1.437760555	-3.92601364	-5.442748069	-3.718337251	2.126915682	-1.640460667	1.819257717	-4.796024295	-4.90427657	-3.776953426
-3.789200471	-3.562480305	-5.510885842	-1.567844563	1.568739838	-3.892467771	-5.526012135	-2.826337627	2.075772727	-1.898147255	1.535175406	-4.956175446	-5.113803413	-4.075327524
-3.981050074	-3.055728486	-5.797148045	-1.952852315	1.868319448	-4.629275387	-6.019660497	-3.95079614	1.679700751	-2.776950737	2.086625444	-5.226097978	-4.679280927	-3.707541563
-4.012652132	-2.760816483	-5.951853871	-1.916995445	1.662653175	-4.59807674	-6.112851195	-4.416975998	1.607358582	-3.234171521	1.915289376	-5.290873803	-5.265675123	-4.020081966
-3.452222685	-2.80316222	-5.77061369	-1.739912056	1.620636965	-3.88486887	-5.910594303	-3.669154403	1.866631224	-1.849815084	2.497010689	-5.268635809	-5.143664175	-3.928617259
-4.417776112	-1.758557351	-6.014213282	-1.211481061	1.981442333	-4.194711082	-5.989327231	-3.993454235	2.181533268	-2.815630075	3.037243831	-4.944843682	-5.012495452	-3.609137443
-3.70932221	-3.27856137	-5.462366109	-1.815209405	1.400168554	-4.096397868	-6.087481926	-3.193744822	1.908650972	-2.090652323	1.771890207	-5.020759458	-4.865007544	-3.632810106
-3.812529992	-2.427286606	-5.165927135	-1.431168594	1.708295311	-3.646650128	-4.890464434	-3.868986636	2.325060918	-2.003388217	2.546897767	-4.830322602	-4.654355691	-3.747317446

-4.081814924	-1.490547789	-4.961077225	-1.374785197	1.752236989	-3.474009564	-3.644486534	-3.185475738	1.981771624	-2.190912879	1.858153108	-4.262897137	-4.75283195	-3.775149523
-4.294855833	-1.313909454	-4.963527427	-1.476244235	1.561560518	-2.773995166	-3.612803028	-2.902739728	1.843686002	-2.082976242	1.639952794	-4.255228355	-4.663708383	-3.917216599
-3.998825456	-1.894120318	-4.872771466	-1.459804175	1.609159579	-3.518689349	-3.839767809	-3.340305672	1.959896367	-1.838917117	1.560161206	-4.16675979	-4.521748794	-3.899079036
-4.33125652	-1.915022376	-4.936275597	-1.419855958	1.779664672	-3.717427667	-4.122386924	-3.398149118	1.975770025	-1.97550789	1.810362255	-4.110694173	-4.746532619	-3.808569523
-4.092671911	-2.489724385	-5.196407657	-1.536927746	1.328469918	-3.794425906	-4.220785727	-3.515335561	1.694088273	-2.090643212	1.314953542	-4.458382345	-4.877099555	-4.12167225

hsa-miR-3679-5	hsa-miR-3689d	hsa-miR-370-3p	hsa-miR-3714	hsa-miR-371a-5	hsa-miR-371b-5	hsa-miR-373-5p	hsa-miR-3917	hsa-miR-3928-3	hsa-miR-3934-5	hsa-miR-3937	hsa-miR-3944-3	hsa-miR-423-3p	hsa-miR-423-5p
-2.469176789	-6.817732987	-4.606024694	-6.14718326	-1.842492388	-4.460077483	-4.288959428	-3.982005078	-3.711382092	-5.255511101	-0.975209394	-4.600925804	-3.607766007	-2.765091156
-1.718353463	-6.114223649	-4.499093913	-6.091141968	-2.578093346	-3.362961049	-4.593130808	-4.121741121	-2.545757075	-5.228255739	-0.723499737	-4.728218247	-2.985990194	-2.566510867
-1.546669181	-5.732780811	-4.609899033	-6.157339752	-2.009425136	-3.501380546	-4.616923393	-4.125478968	-2.482887863	-5.493620176	-0.61575144	-4.768771082	-3.548957367	-2.865242702
-1.613653289	-5.792430162	-4.392178267	-6.065589583	-2.040278318	-3.322940922	-4.526979178	-4.270702324	-2.579526326	-5.453261754	-0.679200894	-4.656592689	-3.349891539	-2.556742285
-1.882406993	-6.120005603	-4.321849699	-6.012612149	-1.917153575	-3.796321005	-4.141790642	-4.00673782	-2.934512246	-5.202678719	-0.922185589	-4.437647192	-3.482645114	-2.452505687
-1.919972402	-5.990391091	-4.52801236	-6.201922143	-2.297608035	-3.844826433	-4.462845354	-3.881569536	-2.851720219	-4.993850191	-0.896967439	-4.656494141	-3.109896493	-2.367434683
-1.93920444	-5.827316002	-4.350931218	-5.472594405	-2.05798013	-3.784834336	-4.344846585	-4.143385871	-2.818286261	-5.015869228	-0.634150172	-4.533276556	-3.343062417	-2.492930209
-1.592029603	-5.971752329	-4.432255749	-5.955578668	-2.685483187	-3.078786214	-4.919205227	-4.228136974	-2.239679764	-5.261786664	-0.502766704	-4.650651027	-3.026106639	-2.357526867
-1.507875301	-6.27144567	-4.546803986	-6.361480655	-2.202210662	-3.060960439	-4.670046576	-4.134578499	-1.341711656	-5.533237227	-0.682843495	-4.776531214	-3.569064665	-2.607625271
-1.78822072	-6.159228391	-4.303139095	-6.018898737	-2.331625096	-3.6272447	-4.405995679	-3.818055754	-2.646755724	-5.084498089	-0.736296483	-4.66706044	-3.806814227	-2.524186339
-1.417451996	-5.825123235	-4.44955071	-5.567785298	-2.346052442	-3.412832883	-4.432648506	-4.173715205	-2.083027089	-5.392177603	-0.660592726	-4.563674315	-2.972267451	-2.578606744
-1.784715841	-6.568200036	-5.159777381	-6.976298703	-2.467098526	-3.26172879	-4.528900655	-4.111899705	-2.543094281	-5.895277109	-0.663585663	-4.908895132	-4.900353864	-2.940791791
-1.566920223	-6.098204112	-5.061100322	-6.597995304	-2.73087061	-2.802457997	-4.782094504	-4.401926701	-2.251674802	-5.935950932	-0.587285907	-5.280018365	-4.447973018	-2.884114042
-1.804577439	-5.749990558	-4.735665803	-6.026753813	-2.322775535	-3.721884894	-4.647390797	-4.189108195	-2.65162181	-5.551552691	-0.86915702	-4.981432334	-4.126326384	-2.97111942
-1.899293142	-6.712202978	-4.717104773	-5.694408898	-2.491384453	-3.609526481	-4.475020771	-4.075743246	-2.576858568	-5.556074239	-0.999259484	-4.949711156	-3.72061503	-2.748496614
-1.622243425	-6.20749753	-4.623197225	-5.981133342	-2.248072143	-2.904356169	-4.354343413	-4.175964505	-2.115166194	-5.629639186	-0.550499859	-4.94864634	-4.233110674	-2.792827383
-1.532217284	-6.267275895	-4.7967738	-5.748373446	-2.413740755	-3.380188908	-4.298179119	-4.101560591	-2.615222479	-5.488556136	-0.705896173	-5.253196236	-4.437591417	-2.780862602
-1.809884791	-5.708452741	-4.958940624	-5.803498994	-2.892594344	-4.319893256	-4.438092521	-4.17132583	-2.166809868	-5.637888078	-0.668769294	-5.326258809	-3.171848229	-2.819614254
-1.829645684	-5.706931321	-4.387880601	-5.384992979	-2.303523519	-3.647796991	-4.207162874	-3.93301897	-2.423418617	-5.34105959	-1.050757817	-5.078385381	-3.554804272	-2.704016204
-1.4988517	-5.674589915	-4.664399233	-5.444431351	-2.485427165	-3.318185388	-4.430626733	-3.992183198	-2.275308141	-5.33502929	-0.754039711	-5.074240262	-3.482732979	-2.834460006
-2.02960874	-6.427839607	-4.633357804	-5.574664987	-2.412710244	-3.427397439	-4.517725807	-4.017617957	-2.592081414	-5.513948903	-0.72228834	-5.058728727	-3.274721329	-2.612370949
-2.003454185	-6.829741958	-4.722483316	-5.966950813	-2.451344332	-2.614142516	-4.550140756	-4.092662951	-2.72043767	-5.655228833	-0.84010609	-4.965861289	-3.894761724	-2.705726654
-1.44225267	-5.65996662	-4.728728054	-5.997683235	-2.450741442	-3.150506071	-4.548230498	-4.382758174	-2.080916727	-5.671760005	-0.736937223	-4.929807486	-3.342872502	-2.588758985
-1.832516109	-5.288745446	-4.664976597	-5.440985208	-1.599316679	-3.248627675	-4.014001377	-3.961247552	-2.556405167	-5.342031557	-0.76668217	-4.822795701	-4.569491146	-2.546303386
-1.398569547	-5.743367937	-4.602714585	-6.205374553	-2.771387875	-3.408475467	-4.429068874	-4.04419799	-2.128359425	-5.345198228	-0.713823032	-5.026453209	-3.947846753	-2.667237879
-1.868253287	-5.55685912	-4.992938098	-5.488159575	-1.413118363	-3.587670957	-4.115631115	-4.073884178	-3.052933191	-5.697361502	-1.044842247	-4.996998603	-4.594972389	-2.221745314
-1.741552614	-6.210288522	-4.953990264	-7.006078478	-2.9568195	-3.125981213	-4.942905584	-4.374782146	-2.459210962	-6.086074222	-0.799705339	-4.979556771	-3.997012757	-2.841700079
-1.764375434	-6.153997133	-4.720284196	-5.25245582	-2.202564596	-3.432648424	-4.200724118	-3.932729005	-2.921269604	-5.465227437	-1.13459931	-4.885607055	-4.703444254	-2.834089501
-2.181274633	-6.299882057	-4.574040117	-7.14221328	-2.362003505	-4.393786497	-4.499997595	-4.100600681	-3.345831739	-5.804236034	-1.507991262	-4.640986932	-3.727785229	-2.974141288
-1.41375949	-5.85388951	-4.568284523	-6.058353773	-2.366607305	-3.361102228	-4.759365655	-3.970586604	-2.462982159	-5.552677565	-0.858608676	-4.760777856	-3.814532639	-2.438510182
-1.475600133	-5.414235987	-4.474363777	-5.38386423	-2.395625062	-2.820789232	-4.680869126	-4.087610329	-2.135669352	-5.442016653	-0.779335274	-4.648249901	-3.602299289	-2.449609862
-2.080048431	-9.411187214	-4.764636783	-5.967592141	-2.624884751	-4.945578412	-4.674880793	-4.516099602	-3.869687305	-5.852329603	-1.868102614	-5.822338224	-5.161881147	-3.827906306
-2.114584	-6.224977673	-4.375554183	-6.399051579	-3.300240663	-3.148507373	-4.816686224	-4.046548158	-2.006314447	-5.4423049	-0.457728732	-5.162825462	-6.257402608	-2.060898543
-2.524286062	-6.564509694	-4.610103876	-6.785151685	-3.206901764	-2.756934967	-4.684341707	-3.890186229	-2.697906221	-5.211385096	0.022561272	-4.87273936	-6.329848615	-1.606149417
-2.000361115	-6.285207321	-4.959834257	-6.4935654	-2.690931741	-3.819881353	-4.668710126	-4.285151551	-3.074726235	-6.394716066	-0.762335164	-5.265375774	-7.447332606	-2.121501945
-2.455330463	-7.351621272	-4.932116715	-6.428884684	-2.677510226	-3.266664548	-4.363927526	-4.034621699	-3.343115822	-5.283629718	-0.221627177	-4.722729289	-6.072711808	-1.626605285
-2.979063427	-6.220228692	-4.910158735	-7.199570992	-3.125161267	-3.332783309	-3.819465548	-4.147888835	-2.503323556	-6.228453983	-0.629331927	-6.604983751	-5.290882439	-2.507969791
-2.687944165	-5.983762772	-5.256693326	-5.467116411	-3.41091984	-3.493499195	-4.739565049	-4.160181574	-2.387053687	-5.078582157	-0.110514129	-5.033816151	-5.885624139	-1.668534433
-2.363546533	-5.993862115	-5.056593583	-6.417378152	-1.53566901	-3.453357445	-4.555349987	-4.56027398	-2.768900546	-5.893251884	-0.240095037	-4.87954449	-5.920107589	-1.663495352
-3.630040555	-5.550386681	-4.642453027	-5.913834785	-2.841965096	-3.865157018	-4.188379747	-3.821720526	-2.968207335	-5.11624483	-0.622199209	-5.048021804	-5.345915533	-2.752797514
-2.540839903	-5.992139935	-5.176512772	-6.236243891	-2.858843325	-4.112976779	-4.60373941	-4.165050681	-3.42627219	-5.453745767	-0.759038236	-4.946488445	-6.284898312	-2.010204845
-2.201144726	-5.45428947	-5.040794007	-5.371946573	-3.680161671	-3.247066792	-5.575091807	-3.887830177	-2.242330855	-4.455029495	0.031479621	-5.238293635	-5.696095986	-2.080412205
-2.304617134	-5.426606341	-5.195763222	-5.119001941	-3.529552368	-3.019296241	-5.022435779	-3.68575508	-1.256679851	-4.700343643	-0.219786721	-5.497784236	-6.880317845	-2.213942906
-2.57609438	-5.463931792	-5.047662632	-6.388822809	-1.106458748	-4.446166869	-3.520466098	-3.917732155	-4.117118488	-5.545618594	-0.409453692	-4.96858715	-6.587780786	-2.83543683
-2.580957182	-4.836975452	-4.901734513	-6.463174069	-3.290047035	-3.668401292	-4.845151399	-4.18732431	-2.570316434	-5.677457365	-0.044029196	-5.307723235	-6.55815458	-1.939811792
-2.300683919	-5.922302079	-4.971150512	-5.952141697	-3.135952192	-3.665001846	-4.689165146	-4.266438958	-2.640569203	-5.960590428	-0.10587931	-5.401040315	-6.625777712	-1.780004132
-2.336583264	-6.991776877	-4.942952624	-6.10873715	-2.399716146	-3.311305421	-4.742342171	-4.341951008	-3.13733253	-5.874065237	-0.130912244	-5.196870891	-5.862672438	-1.561998303

-1.842836993	-5.68420003	-4.981604843	-6.298815839	-3.217812926	-3.2393039	-5.135602436	-4.321202104	-2.51860513	-5.650705862	-0.164031671	-5.329607476	-5.29072036	-1.90667562
-2.815756496	-5.251633016	-4.31104869	-5.248930032	-3.372430584	-3.141169911	-4.106729331	-3.577752265	-2.26570575	-4.355938845	-0.020794215	-5.417003547	-6.472885	-2.05474605
-2.5961392	-6.26236695	-4.634479529	-5.464750366	-3.050348466	-3.577403586	-4.212779902	-3.741641099	-2.778838986	-4.847937521	-0.15563018	-5.300859305	-5.781310687	-1.70777035
-1.88656596	-5.532031099	-5.11652924	-6.09009785	-1.391146994	-3.668023342	-3.987514532	-4.083411799	-3.159772181	-5.434429646	0.465944362	-5.149868445	-6.5081632	-2.692281521
-2.357036899	-5.66010709	-4.933149513	-5.778121168	-1.885202369	-3.687838599	-4.249814284	-4.236320568	-3.49121799	-5.602178547	-1.085909287	-4.900388122	-6.577777548	-2.883097467
-2.771480686	-5.041742073	-3.861757819	-4.671156116	-3.357562123	-3.320531429	-4.142183172	-3.475066724	-2.261799242	-3.570684622	-0.025942236	-5.791203148	-4.955853566	-2.438188126
-2.315859557	-4.892708917	-3.598897303	-4.969824892	-2.236246612	-3.004664075	-3.548548924	-3.025758954	-2.753369631	-3.758930159	-0.338365164	-5.176373694	-5.583790815	-2.529739326
-2.462668774	-6.282440603	-4.797236011	-6.118102142	-1.707276957	-3.687762322	-3.887108624	-4.401052973	-3.174421305	-5.900394418	-0.969503357	-5.221970437	-5.055939642	-2.356059051
-2.200005149	-5.812903598	-4.607907455	-7.118488387	-3.199178118	-2.895583003	-4.430015649	-3.680840309	-2.124900669	-5.281776731	0.11499171	-5.314818131	-6.031821425	-2.145269497
-2.018842305	-5.758139087	-4.866829975	-6.100990687	-3.022124916	-2.918352123	-4.728948752	-4.154079652	-2.124232142	-4.831352038	0.112763716	-5.28782128	-6.47922443	-1.910479098
-2.183722818	-5.383515851	-5.126680944	-5.892981692	-3.736909274	-2.819990113	-4.672764946	-4.23146043	-2.014268051	-4.871416711	-0.605902003	-5.659755688	-4.316182858	-2.917128803
-1.939023678	-5.308826163	-4.466718231	-5.926675701	-3.301596876	-2.985257771	-4.73967692	-4.298961942	-2.164814434	-3.914775484	-0.151756544	-4.88557155	-5.293630804	-1.949551076
-2.822812892	-5.782719504	-5.367831213	-6.833461401	-2.488849818	-3.95941603	-4.37902226	-4.28952831	-3.523951588	-5.562508498	-1.047931291	-5.098933484	-6.411043027	-3.015074951
-2.635059982	-4.774223688	-4.428652245	-5.882368714	-3.297871277	-3.502483725	-4.566114581	-3.820275362	-2.311231425	-4.059441235	-0.13957186	-5.683284859	-6.085355484	-2.658796043
-2.828126074	-4.481014786	-4.338524078	-5.671792025	-3.189450088	-3.386760565	-4.156877418	-3.608296877	-2.489161718	-4.356739598	-0.255196299	-6.586763679	-5.780613791	-3.219994558
-2.713748882	-4.758278805	-4.468850518	-5.336961913	-3.012320985	-3.452554469	-4.113760304	-3.6700094	-2.624794021	-4.318870352	-0.235485283	-4.976277761	-5.304011772	-2.271520837
-2.539702986	-5.53309162	-5.366542573	-6.122922867	-2.374912491	-3.326688769	-4.215449195	-4.649949025	-2.788139752	-5.928475212	0.040615175	-5.386581211	-4.372467783	-2.575994529
-3.284254139	-6.646341165	-5.254777579	-6.29389668	-2.119161555	-3.877192173	-4.069428678	-3.883786168	-4.095587629	-5.331409832	-0.818887376	-4.520072988	-6.411138989	-2.939166315
-2.269592372	-5.625641889	-4.736221417	-6.946888226	-2.198748123	-2.819187403	-4.550932939	-4.249690595	-2.693674476	-6.390838258	0.034948769	-4.994890971	-6.800973501	-1.705149536
-1.734277758	-5.613770913	-4.958517388	-5.623233086	-3.925975878	-3.066320319	-5.3819381	-4.27667904	-2.155698937	-5.015874289	-0.495283214	-5.353743594	-5.493891826	-1.719259193
-1.542956125	-5.858072613	-5.114099138	-5.976858571	-2.563201688	-3.07473979	-4.665130058	-4.0290366	-2.62969273	-3.524113066	-0.625369761	-4.813123464	-5.793806451	-2.295656621
-1.521211047	-5.753321369	-3.915701847	-4.800565882	-2.467060955	-2.835970841	-4.655084998	-3.34875925	-2.30074002	-4.158436846	-0.883200682	-5.434150924	-3.749451974	-2.947367176
-1.624907521	-6.700569883	-5.19403567	-6.139619188	-2.910920352	-2.833711016	-4.907558815	-4.581210927	-2.732145932	-5.995523132	-1.062576194	-5.550014946	-5.631322065	-2.038756226
-2.030350467	-7.372290488	-5.119012314	-7.214024513	-3.241442382	-2.358998721	-5.118279272	-3.964280789	-2.542814266	-5.489091459	-0.263427838	-6.118848428	-8.165082483	-2.434311807
-1.605178434	-7.108763001	-5.2274606	-6.860253145	-3.247032688	-3.14272955	-4.875420339	-4.257006345	-2.687788228	-5.870899828	-0.841013403	-5.607683112	-5.086739904	-2.044585785
-1.572362916	-5.759291094	-4.432026694	-5.255667322	-2.56347163	-3.079311338	-4.260355785	-3.335044981	-2.10442884	-4.112729338	-0.44411378	-5.603434703	-4.494521547	-2.76754966
-2.00147025	-6.101802718	-3.732316087	-4.513400103	-2.574548683	-3.339600078	-4.239816043	-2.700361794	-2.581871948	-3.25761757	-1.406374111	-4.544322804	-5.522541662	-3.014220769
-2.278976827	-6.778807065	-4.268557895	-4.598950046	-2.461312674	-3.578620138	-4.5888758	-3.111031825	-2.763669075	-3.76085494	-1.236719705	-4.385157757	-5.791113431	-2.547856113
-1.351869705	-6.341116464	-4.318564019	-4.845842992	-2.817018178	-3.56904391	-4.492463309	-3.173282755	-2.39027763	-3.950067828	-0.928988908	-4.873233221	-4.34329621	-2.801203975
-1.420306366	-6.233869639	-4.311290988	-5.015109011	-2.81460389	-3.429662617	-4.579349318	-3.24395063	-2.123549107	-4.087334399	-0.569477847	-4.865344795	-3.533624788	-2.649419402
-1.310664917	-6.089815811	-3.901707785	-4.16426556	-2.764916279	-3.262152347	-4.489296856	-2.865254743	-2.197587384	-3.285437732	-0.70439058	-4.821135555	-5.223512373	-2.889427532
-1.840257705	-7.121938778	-4.065317747	-4.539855911	-2.559818825	-4.181116113	-2.691042214	-2.676440157	-2.784771812	-3.706490961	-1.051627108	-5.074146161	-5.091231008	-2.930834738
-1.595950774	-5.845817622	-3.641237833	-3.912247814	-2.479720101	-2.921607335	-4.644953175	-2.904713033	-2.448631459	-3.329190909	-0.889303398	-5.136490497	-3.615184472	-3.106727923
-1.604376089	-5.021562052	-3.759506723	-2.950641718	-2.311976992	-3.045839591	-4.469998144	-2.44703029	-2.232537467	-3.205078031	-0.950051335	-4.902338012	-4.518741792	-2.9686252
-2.624482577	-5.961837106	-5.03069881	-5.844054206	-3.805379029	-3.624695858	-4.892209244	-4.168649183	-2.851511927	-5.186501571	-0.439738965	-5.101426765	-6.45198722	-2.46125507
-2.722162598	-6.274325962	-4.91204843	-6.105680225	-2.78406293	-3.473420588	-4.628698244	-3.948146717	-3.010906111	-5.72699331	0.103471755	-4.980566206	-5.945351552	-1.987832581
-1.91034407	-4.815038912	-4.51215691	-5.224973186	-3.484808839	-3.30772864	-4.825118168	-3.847160932	-2.168570704	-4.392195428	0.122170128	-5.201094893	-5.329636014	-2.101842184
-2.210721816	-5.417158574	-4.322433287	-6.021435058	-3.226383862	-3.416565817	-4.71339563	-4.00144879	-2.207221881	-4.583756739	-0.31669708	-5.405224454	-5.174717197	-1.760656093
-3.159754272	-6.315747136	-5.020425946	-6.313543952	-3.222840204	-3.59569713	-4.826349809	-4.246533047	-2.96415406	-5.812872264	-0.155904248	-5.164658253	-6.799373251	-1.618740701
-2.746407589	-5.755528983	-4.260878508	-6.893806462	-2.023493432	-4.033574937	-3.683131412	-3.650738926	-2.79182506	-5.445421796	-0.654021957	-5.132484464	-6.275541171	-2.079448388
-2.055016448	-5.488120422	-4.56675205	-6.035448099	-3.594148555	-2.795555043	-4.621518386	-4.037264161	-1.965095758	-4.591502249	0.047121731	-4.834449177	-5.278369179	-2.275684666
-1.640354077	-5.590710469	-4.814439591	-5.980587137	-3.552130994	-3.015674289	-5.222025644	-4.277757748	-2.353133467	-4.011011472	-0.14708136	-4.955254011	-6.045177074	-2.294074683
-1.860925598	-4.411985273	-4.655007304	-6.309878462	-2.996597443	-3.246622124	-4.935243859	-4.206576349	-1.612540812	-4.600595762	-0.361287448	-5.347984355	-6.355664179	-2.302739372
-2.836830102	-5.104573887	-5.14306365	-6.596838917	-2.299524309	-4.002993832	-4.677616426	-4.38499189	-3.438473394	-5.723466966	-1.320309916	-5.615484477	-6.635425177	-3.699749819
-2.699320213	-5.172350922	-5.209571948	-6.079459884	-2.795179385	-3.613410223	-4.860834499	-4.220213467	-3.400822787	-5.21802746	-1.088879371	-5.569229017	-7.304395848	-3.383900147
-2.156522059	-4.485031704	-4.724284491	-6.192975707	-3.818539783	-3.587463116	-5.025180425	-4.381444396	-2.626161371	-5.50711273	-0.046839058	-5.619561172	-5.53743796	-2.504054246
-3.027343462	-5.35316675	-4.241570995	-5.978915813	-2.734183279	-3.35885806	-3.781552296	-3.555121565	-2.331852323	-5.529791556	-0.451842362	-5.973595749	-6.28679706	-2.322471942
-2.271789144	-4.943989525	-5.0472038	-5.870878683	-3.580549322	-3.243976453	-5.286976509	-4.338385661	-1.867003629	-5.658002822	-0.011795303	-5.160704772	-6.133615295	-2.392663493
-2.054903392	-5.476507526	-4.052143746	-5.3674288	-3.406838807	-3.374150901	-4.469471089	-3.859669287	-1.920925909	-4.308577839	-0.268344076	-5.00982702	-5.529066019	-2.07913718

-1.385134288	-5.670894586	-3.789818963	-4.122425992	-2.564466369	-3.184377676	-4.575933437	-2.796154106	-2.406315916	-3.24020487	-0.756382834	-4.823249351	-4.252368632	-2.554758456
-1.492279641	-5.817911131	-3.535594199	-4.161683197	-2.683251952	-3.157974389	-4.242340079	-2.638817418	-2.291922114	-2.750463483	-0.895938659	-5.07056817	-4.206797483	-2.703087108
-1.180846833	-6.034317973	-3.708942301	-4.196728374	-2.799991358	-2.878727476	-4.503860865	-2.910817043	-2.182261674	-3.355890493	-0.518888778	-5.036388693	-3.784413394	-2.717201741
-1.320057114	-6.369508638	-4.031184643	-4.794633125	-2.948068302	-3.25111885	-4.411502177	-3.052981936	-2.435333131	-3.595911053	-0.768555722	-4.706880827	-4.236164185	-2.709979574
-1.339255029	-6.085491521	-4.194255569	-5.033227364	-2.909063986	-3.153438997	-4.897016829	-3.459822822	-2.314662119	-4.20350312	-0.827241696	-5.206751257	-3.656207858	-2.744964125

hsa-miR-4253	hsa-miR-4257	hsa-miR-4258	hsa-miR-4259	hsa-miR-4265	hsa-miR-4269	hsa-miR-4270	hsa-miR-4271	hsa-miR-4274	hsa-miR-4276	hsa-miR-4279	hsa-miR-4284	hsa-miR-4286	hsa-miR-4298
-4.218341835	-4.255427299	-2.469430125	-4.91730967	-4.49862821	-4.176367438	-0.896192104	-0.348827249	-3.755471158	-3.106432401	-3.851356291	-2.564085817	-3.048719839	-3.06354626
-4.472657211	-4.192419632	-1.505973273	-4.698836103	-4.143843312	-3.513545971	-1.435189002	-0.535007271	-4.176282936	-3.151705339	-4.038344794	-0.966716387	-2.78951838	-2.965019511
-4.619995547	-4.193398371	-2.229456022	-5.178192635	-4.58757149	-3.378988779	-1.577416939	-0.515845787	-3.60560474	-3.182613769	-3.738901953	-1.879364817	-2.581004615	-2.983008897
-4.560571778	-4.100889665	-2.260627401	-5.028755436	-4.533670642	-3.46553532	-1.290686578	-0.26925285	-3.792761186	-3.428888678	-4.012197593	-2.193727264	-2.761792497	-3.079742695
-4.342849625	-4.080557345	-1.970749459	-4.607886815	-4.609535367	-3.687507048	-1.131301663	-0.278158089	-3.336910761	-3.331565291	-3.81895871	-2.390473973	-2.736347098	-3.079377561
-4.592776168	-3.759106368	-1.288265746	-4.74658221	-4.629884621	-3.611320101	-1.352966171	-0.554041527	-3.709858047	-3.217568417	-4.115664009	-2.132139671	-2.616479497	-3.183564421
-4.593711434	-3.911329312	-1.87845579	-4.851701693	-4.282268001	-3.554287915	-1.204586881	-0.269569915	-3.535062539	-3.19936178	-4.011723686	-0.73934025	-2.396757202	-3.00642938
-4.624371222	-4.133093336	-1.785710524	-5.011179795	-4.449810732	-3.286322531	-1.573188357	-0.837900678	-4.066894047	-3.283485239	-4.08761833	-2.437616564	-2.571061611	-3.296323113
-4.628381219	-4.314861957	-1.969755907	-5.02147676	-4.641277766	-3.686044892	-1.555384861	-0.636982132	-3.980429058	-3.132487745	-4.28178574	-2.330284399	-2.868852145	-3.285352828
-4.670263841	-3.947760293	-0.951796393	-4.513076706	-4.284960342	-3.282235422	-1.422653746	-0.657921166	-3.949890773	-3.174916733	-4.087181918	-2.162444361	-3.00653947	-2.899112442
-4.572264303	-4.169384775	-1.847453061	-4.946081445	-4.641523792	-3.295885782	-1.502418181	-0.710372502	-3.743894145	-3.043205904	-4.497957923	-1.453424144	-2.570318233	-3.346515904
-4.76254348	-4.576014755	-1.945927279	-4.955200153	-4.456646879	-3.71965172	-1.553675735	-0.955177121	-4.355279914	-3.108884257	-4.345255083	-4.942974695	-3.711650268	-3.004298872
-4.69741312	-4.657300024	-2.163474439	-5.522998911	-4.769901669	-3.743275536	-1.629967845	-0.776335041	-4.358643422	-3.733621904	-4.347025903	-4.08921558	-3.619043917	-2.994775708
-4.732669538	-4.23403932	-1.670578084	-5.297814499	-4.681642856	-3.447902011	-1.599787296	-0.544320606	-3.765128038	-3.2970759	-4.380034766	-4.232438889	-3.32828915	-2.995781377
-4.618082403	-4.511137418	-0.917461519	-5.065022473	-4.574528563	-3.594818234	-1.522794276	-0.989992089	-4.133767768	-3.037755798	-4.473144643	-3.149394586	-3.151360092	-3.380573214
-4.409746132	-4.350568835	-2.041916343	-5.082738549	-4.504901351	-3.177449362	-1.597975143	-0.721476257	-4.116128168	-2.920989634	-4.420613587	-3.328890734	-3.350049784	-3.246471218
-4.685408365	-4.215462277	-1.10202588	-4.762270959	-4.41218641	-3.683852501	-1.657876789	-0.974899248	-4.105908029	-3.138051277	-4.45196453	-4.670172688	-3.390296954	-3.403547839
-5.093280857	-4.436387875	-1.881377183	-5.316630454	-4.497348012	-3.362551976	-1.708760679	-1.054631883	-3.97823198	-3.266963359	-4.44945687	-1.428534456	-2.710752177	-3.111922619
-4.786189824	-4.022627632	-1.810210939	-4.959518719	-4.606045756	-3.866460516	-1.309974617	-0.667873589	-3.774832164	-3.229162389	-4.323775461	-2.792243162	-3.186324345	-2.98162235
-4.752154314	-4.304883901	-1.607806539	-4.90881879	-4.589498882	-3.550477697	-1.605726616	-0.850653422	-3.919556431	-3.248807829	-4.645403195	-1.723356806	-3.041682253	-3.079535365
-4.585520713	-4.272581103	-1.106139135	-4.938574338	-4.376888932	-3.441788383	-1.732909894	-0.907349588	-3.979680724	-3.258073809	-4.540363255	-1.658014951	-2.660867671	-3.203048599
-4.299187313	-4.289284095	-0.932013453	-4.976931169	-4.516995074	-3.520306306	-1.607813799	-0.943886665	-4.206055708	-3.010175956	-4.506946932	-2.686899634	-2.944348413	-3.529332368
-4.70520545	-4.394817342	-2.002717719	-5.285242356	-4.711609911	-3.516514036	-1.403756727	-0.675503027	-3.862454289	-3.451773893	-4.321408141	-1.891669005	-2.991940286	-3.103607974
-4.729033148	-4.152702842	-1.985567535	-4.881079885	-4.280274862	-4.014364157	-0.805582998	-0.335984463	-3.643611332	-3.510707686	-4.056832499	-3.562929836	-3.454158461	-2.736190643
-4.702401572	-3.988764046	-1.309905384	-5.052636834	-4.699557514	-3.372919324	-1.724370703	-0.89271108	-4.020950106	-3.052138234	-4.526183206	-4.023376576	-3.195175997	-3.35483671
-4.637917326	-4.131533298	-2.175251687	-5.091923071	-4.639613701	-3.908038553	-0.703931084	-0.359857669	-3.515592394	-3.2040579	-4.060741871	-3.740633869	-3.442095139	-3.010541258
-4.859587681	-4.677356153	-1.651695194	-4.901336702	-4.954581211	-3.682302265	-1.815504637	-0.934001146	-4.403483976	-3.444139526	-4.482154099	-3.423150063	-3.218407295	-3.219638978
-4.89498289	-4.345024615	-1.78762281	-5.025800328	-4.714344256	-4.101066609	-1.185152041	-0.649666473	-3.989261223	-3.42610759	-4.1984918	-3.046457796	-3.543215012	-3.010564376
-4.921119627	-4.298694985	-1.699698039	-5.312230796	-4.545861057	-4.177553572	-1.442904556	-0.94475214	-4.117664925	-3.345641773	-4.316060391	-2.597347475	-3.247877887	-3.71567921
-4.869679062	-4.037505963	-1.899896502	-4.836046942	-4.641506813	-3.311714336	-1.720153836	-0.766522638	-4.055013101	-3.021141094	-4.151328473	-3.866983689	-3.393381708	-3.299129201
-4.803133971	-4.105626284	-2.101344598	-5.031640571	-4.36600006	-3.210020564	-1.390198784	-0.766420093	-4.208770544	-3.606059272	-4.278940376	-1.890417212	-3.119736473	-3.187560206
-5.682043699	-4.286648184	-2.899062385	-5.136338982	-5.563591754	-5.095246983	-0.906494869	-1.170565656	-4.833963619	-4.241803161	-5.138427941	-5.500127909	-3.836102794	-3.200506743
-4.562157251	-4.127216717	-1.050663737	-4.819396552	-4.567079147	-3.027198955	-1.539078222	-1.313055654	-4.714391677	-3.629821818	-4.594567331	-4.894799583	-3.971350956	-3.610240977
-4.728825311	-4.28823229	-1.184334438	-4.873009595	-4.107945792	-2.660519548	-1.408544457	-0.861630944	-4.620969751	-3.896423887	-4.512539803	-5.382892725	-3.979107748	-3.668606649
-4.69005273	-4.919575045	-1.6392844	-5.246220472	-4.654606115	-3.355964125	-1.909379638	-0.720144519	-4.245785233	-3.610559906	-4.743846586	-5.612912248	-4.439342866	-3.633006886
-4.027110861	-4.357066463	-1.074183574	-4.68958611	-4.06914602	-3.32752334	-1.946892437	-0.941805823	-4.014592173	-3.28029316	-4.351156489	-4.84597835	-3.477312742	-3.761261995
-4.687501179	-4.700224821	-0.248449994	-4.409298701	-5.708291507	-3.814181576	-1.858017646	-1.977361961	-4.092762484	-4.10716251	-5.854824857	-4.197075108	-4.436726	-3.601721884
-4.870227362	-4.442203685	-1.106845056	-4.793989216	-4.544708649	-2.974437917	-1.734653012	-0.81277685	-3.973098716	-3.992537727	-4.54839119	-4.37901605	-3.887486069	-3.371209397
-4.371061958	-4.792880147	-1.989534786	-5.111135334	-4.497973847	-3.226568783	-1.714870265	-0.755824622	-3.985070596	-4.031193932	-4.314039217	-4.339467731	-4.312659435	-3.42810525
-4.919358587	-4.147313113	-0.628246783	-4.50190102	-4.648431216	-4.16490327	-1.641687439	-1.258276706	-4.131751998	-3.950556451	-4.725617368	-6.305837162	-4.293000744	-3.490890672
-4.263932413	-4.45405084	-0.960629083	-4.934810347	-4.286160589	-3.836339839	-1.889845066	-0.86586293	-4.095996132	-3.669156159	-4.420789472	-4.201382417	-3.865043967	-3.553519633
-5.321033941	-4.960665461	-1.75356496	-4.987182734	-4.464731664	-3.629419009	-1.94124574	-1.516308022	-4.303392676	-4.445038251	-4.479706157	-5.105759458	-4.43899554	-3.143864905
-5.496603098	-2.135920527	-2.112876577	-5.067759761	-4.686286897	-4.224237275	-2.348600629	-1.957041139	-4.644805466	-4.712104827	-4.739552842	-4.642558553	-4.860812015	-3.351079906
-3.633296941	-4.175670629	-2.782495151	-4.863809945	-4.613706078	-4.457844534	-0.313082549	0.261056976	-2.855831387	-3.628033701	-3.868099266	-4.557216557	-3.975438974	-2.536975412
-4.676368806	-4.956490408	-1.5713974	-4.627241017	-4.853981171	-3.455328099	-1.743620294	-1.329588928	-4.499322029	-4.059187293	-4.782689534	-4.737097719	-4.525481757	-3.64271402
-4.616010526	-4.60231946	-1.630301073	-4.934439025	-4.793122165	-2.968022404	-1.819841805	-1.026439735	-4.409565246	-3.653074497	-4.802776689	-4.710808761	-4.348936029	-3.62624411
-4.250983514	-4.860103603	-2.26537753	-4.953104174	-4.566729483	-3.07323612	-1.675440637	-0.199778377	-3.81787587	-3.468248759	-4.401773596	-4.62667461	-4.342358289	-3.329379061

-4.65353502	-4.538642339	-2.041758792	-5.069366137	-4.359663628	-3.216007211	-1.615213873	-0.927068046	-4.391351759	-3.808240875	-4.545721661	-3.792176237	-4.197937053	-3.185064409
-4.180858374	-3.810982292	-0.501431753	-3.732769025	-4.786075202	-3.291062012	-1.769620105	-1.643524231	-4.427094906	-3.828426706	-4.885752602	-4.933425847	-3.433589599	-3.175041797
-3.970042569	-3.912768202	-0.611735461	-4.32679524	-4.263737105	-3.175509987	-1.5004001	-0.719767642	-4.140450963	-3.138144487	-4.460804986	-3.722840173	-3.746081805	-3.298258004
-3.942054647	-4.262256834	-3.289833954	-5.247247006	-4.265324232	-4.04208789	-0.343118243	0.179104696	-3.023453855	-3.712322445	-3.835707451	-4.753885728	-4.205045691	-2.330406529
-4.654356013	-4.879260486	-2.231853282	-5.003814489	-4.728710813	-4.621836596	-1.234763114	-0.840663684	-3.738375538	-4.146981948	-4.391539135	-5.269197314	-4.56433392	-3.241003796
-4.427876898	-3.317172063	-1.094699087	-3.630231022	-4.020881071	-3.929343528	-1.279534339	-1.673041626	-4.227457473	-3.946533882	-4.501209192	-4.543559426	-3.841455331	-2.676423826
-4.35869677	-2.794287807	-0.120541526	-3.369677556	-4.60757874	-3.979496968	-1.131693042	-2.049366405	-3.8363893	-2.75390142	-4.787896159	-5.601706047	-2.965443335	-3.040455837
-4.635875097	-4.66635076	-1.990383636	-4.586581321	-4.99061821	-4.191572197	-0.656211947	-0.776988653	-3.846598409	-4.005734003	-4.560411371	-5.060783891	-4.316945785	-3.302161364
-5.109834989	-4.223432182	-0.812966454	-4.556895499	-4.619967829	-3.164097384	-1.545777677	-1.217535575	-4.307610356	-3.756342204	-4.775626266	-5.729071599	-4.528181343	-3.391811602
-4.949254991	-4.669595285	-1.927359851	-4.943092466	-4.430503242	-3.011833843	-1.739639945	-1.287108562	-4.458092471	-4.000267941	-4.564655253	-5.06352913	-4.609179256	-3.290521471
-5.347556339	-4.897261739	-1.222120354	-4.271384941	-4.757605829	-4.492993339	-2.174305018	-1.521095744	-4.538022835	-4.515219809	-4.677354233	-6.200724702	-4.519499032	-3.302648957
-4.652218922	-4.555057821	-1.766941077	-4.351594022	-4.749785457	-3.387716123	-2.120087565	-1.133077865	-4.465240406	-4.197804782	-4.571973894	-5.79829066	-4.222020549	-3.631841806
-4.77144405	-4.794864709	-1.83905876	-4.928952872	-4.608507393	-4.442250991	-1.918406897	-1.395376904	-4.178748146	-3.823472201	-4.549309005	-5.922690544	-4.915776301	-3.273810401
-5.027422111	-4.198893968	-1.387781758	-4.498817393	-4.75852805	-3.608218234	-2.017540975	-1.498883659	-4.36443723	-4.066314775	-5.047794048	-6.541669039	-4.683202353	-3.291554392
-4.984543831	-3.598816837	-0.858050853	-4.166512136	-5.777662618	-4.084849947	-1.53553856	-1.880238174	-4.657367285	-4.220231477	-6.204304561	-6.204491135	-3.768059957	-3.179145719
-4.134211766	-3.734827655	-0.490697681	-4.368984976	-4.750605867	-3.33077734	-1.660698735	-1.358922848	-4.22430112	-3.503856185	-4.923284777	-5.262415614	-3.891020419	-3.219476473
-4.761136548	-5.121932643	-1.11236822	-5.115510448	-4.413781566	-3.519217234	-1.827010894	-0.919725041	-3.927504341	-3.627343923	-4.393749939	-5.64305064	-4.070054712	-3.386955222
-4.805527047	-4.389326847	-2.141180004	-4.981996475	-4.458811066	-4.585202773	-1.410133463	-0.494458563	-3.628738621	-3.831467524	-3.83568312	-5.290530252	-4.11679788	-3.372847359
-4.411950859	-4.399716307	-2.353806275	-5.25859972	-4.429848943	-2.818461806	-1.456532944	-0.7406632	-4.1358954	-3.538610268	-4.470802356	-5.580179512	-4.446220878	-3.27662394
-5.39183506	-4.630647701	-1.901807566	-4.705453739	-4.651316901	-3.32949077	-1.991257244	-1.938891469	-4.685700414	-4.612050591	-5.036743648	-2.767826344	-4.054557833	-3.583306921
-4.564560465	-4.268102722	-1.941982723	-4.830020551	-4.108805853	-3.668359616	-1.606269525	-0.839139154	-4.165758114	-3.276983704	-4.24927649	-4.247443152	-3.89749878	-3.265334919
-4.041702948	-2.988531421	-2.096570971	-3.61218868	-4.926064508	-3.519429623	-1.812744732	-1.495549417	-5.061309708	-3.836291948	-4.741636751	-2.562697751	-2.881717218	-3.118688506
-5.212747159	-4.800255288	-2.630965617	-5.497846945	-4.769792127	-3.368339566	-1.703715465	-0.946601804	-4.826586504	-3.861256047	-4.872345867	-2.976145726	-4.344881803	-3.140456347
-4.559703567	-4.036367475	-0.761377528	-4.656531829	-4.95289901	-3.252412957	-1.551272018	-1.599399632	-5.918951412	-3.19710171	-5.3965869	-3.203080878	-3.979554783	-3.391503566
-4.666336353	-4.323963051	-1.088800709	-5.161540489	-5.020697496	-3.390901692	-1.612908201	-0.861480816	-4.750487085	-3.557212661	-4.849040582	-2.265502532	-3.847439571	-3.345551447
-4.130383808	-3.021721257	-1.276971312	-3.901430193	-5.310226247	-3.386204615	-1.670167118	-1.293509262	-4.076088818	-3.346626894	-4.844271385	-3.030116612	-2.794219862	-3.233777721
-3.89420069	-2.426642389	-1.242371426	-3.027633535	-4.998292535	-4.282229133	-1.883086063	-1.902321612	-4.978669368	-4.194668485	-4.542838846	-3.768210345	-2.507261983	-2.953553635
-4.162289386	-2.975610387	-1.189825616	-3.279460527	-4.360415252	-4.470914353	-1.832111281	-1.733221934	-4.870538182	-3.617318093	-4.546141132	-4.318659688	-3.683955812	-3.270608064
-4.159158989	-3.005620794	-1.627742764	-3.656389096	-4.685867461	-3.762439686	-1.741407678	-1.493553192	-4.878163938	-3.679496774	-4.503012381	-2.233771498	-2.704004721	-3.187011841
-4.428644964	-3.032834884	-1.651629849	-3.846927357	-4.610012808	-3.538882655	-1.816348442	-1.307703426	-4.403204024	-3.304793081	-4.597572896	-2.983042705	-2.59665638	-3.303926303
-4.069732419	-2.387543348	-1.779111197	-3.221206473	-4.697802148	-3.844058311	-1.70038845	-1.979815461	-4.870738111	-4.358863822	-4.69933239	-3.299337634	-2.700091162	-3.006757892
-4.594644525	-2.831134214	-0.882717304	-3.695431284	-4.610632235	-4.287718739	-1.608356824	-1.73994591	-4.567175057	-3.155287555	-4.815119373	-3.597298497	-2.93058199	-2.995624504
-4.001089733	-2.505380883	-1.734702594	-3.030657063	-4.789719421	-3.779028913	-1.93546572	-1.605473394	-4.84039295	-4.041616645	-5.032580484	-1.525460895	-2.041359528	-2.915399518
-3.666976828	-2.012789482	-1.718754902	-2.96573475	-4.572312759	-3.756534215	-1.609003566	-0.813480141	-4.910289375	-4.105472378	-4.781891366	-1.569055146	-2.764576311	-2.51504168
-4.815987035	-4.856977612	-2.067243941	-5.125619489	-4.732204679	-3.4426044	-1.661162136	-1.286193735	-4.400314065	-4.206586633	-4.538464637	-5.73388326	-4.56508478	-3.449939035
-4.507423866	-4.266055629	-0.89877837	-5.184489214	-4.13548093	-2.938862198	-1.402175892	-0.562812365	-3.845867211	-3.323238883	-4.344049084	-5.664033352	-4.304540172	-3.30390297
-4.773701734	-3.82625128	-1.220891146	-4.464605305	-4.341516768	-3.420186981	-1.609168098	-1.735251922	-4.170830394	-4.327396608	-4.333072062	-4.904706863	-4.031535695	-3.103902059
-4.620268785	-4.070540852	-1.121571719	-4.150377079	-5.023030222	-3.30513605	-1.644134085	-1.20889474	-4.033426833	-4.289535224	-4.565152569	-3.158453979	-4.006717664	-2.965832813
-4.593259992	-4.59608515	-0.967453634	-5.097115533	-4.965038566	-2.945024175	-1.761063906	-1.034157134	-4.21523911	-3.975925782	-4.850932221	-4.836066713	-4.909387464	-3.596939055
-4.855868499	-3.7693768	0.814478306	-4.287142493	-5.03870587	-3.843700468	-0.899632576	-0.950547915	-3.799327285	-3.713814581	-4.701850431	-5.911611601	-4.314162209	-2.94016543
-4.687082066	-4.550351412	-1.430382768	-4.636664673	-4.883217109	-3.278076092	-1.954174106	-1.191323327	-4.513634783	-4.173069761	-4.741960079	-5.493256501	-4.137072589	-3.36882367
-4.746870896	-4.785129707	-2.337539273	-4.832728733	-4.594055876	-3.438701913	-1.94456287	-1.361586862	-4.756551032	-4.295006958	-4.736461502	-5.352596611	-4.735357551	-3.64942254
-5.298824884	-4.694354096	-2.327941013	-5.082505359	-4.854132879	-4.248166044	-2.127062284	-1.499912106	-4.54711742	-4.719596417	-4.78038066	-6.045270471	-5.104343518	-3.036464372
-5.620872713	-4.828581213	-2.427386996	-4.816602775	-5.008864598	-4.907423207	-1.274141863	-1.309831369	-4.398466504	-4.441753095	-5.005513026	-6.387573347	-5.016716932	-3.629532141
-4.931659282	-4.831662323	-1.42637556	-5.119898834	-4.613931701	-4.744746875	-1.768803496	-1.523416474	-4.708352582	-4.060522208	-4.90901339	-6.539614833	-5.274331004	-3.40533887
-4.969317584	-4.733856492	-1.171781201	-4.67809676	-4.832284956	-3.252297072	-1.850984303	-1.521385417	-4.57177979	-3.944116598	-4.923537332	-6.719264729	-4.779862351	-3.546802143
-4.065540692	-3.420367412	0.458818234	-4.181078611	-4.791587199	-3.144411334	-1.310926954	-1.202194822	-4.309142983	-3.341823219	-5.256156724	-5.096278911	-3.610704427	-3.491577372
-5.321682247	-4.722049986	-2.473337008	-5.406897848	-4.645758664	-3.563683437	-1.892918549	-1.236939591	-4.308373044	-4.340437129	-4.50305282	-3.258220322	-4.252855844	-3.117330942
-4.668170798	-3.875790149	-1.097454776	-4.090078369	-5.174760486	-3.527704692	-1.811782194	-1.708935262	-4.608254667	-3.56914291	-4.742140568	-4.652458092	-3.891168503	-3.439069966

-3.784605008	-2.420159107	-1.522328479	-2.79885353	-4.505967422	-3.787315664	-1.718243478	-1.697362294	-4.838925668	-3.843071173	-4.571737955	-3.724447583	-2.107856006	-2.910776896
-3.477779821	-2.231756816	-1.547369821	-2.732953311	-4.688861961	-3.662362775	-1.699134147	-1.595043282	-4.795272083	-3.794264646	-4.485132209	-0.932237805	-2.583694312	-2.691712365
-3.878419826	-2.556436799	-1.653082535	-2.948302187	-4.608028317	-3.682983564	-1.718175907	-1.660963509	-4.593166229	-3.848459122	-4.537184531	-1.558809682	-2.572913633	-2.981601689
-3.879158595	-2.743823397	-1.494758458	-3.319831876	-4.479151034	-3.588287029	-1.611375254	-1.482895238	-4.670720802	-3.584206977	-4.489513375	-1.82224891	-2.883654183	-3.119505789
-4.197641082	-3.031324459	-2.060241959	-3.822311456	-4.956093623	-3.591079196	-1.968228165	-1.534770375	-4.89522021	-4.09426801	-4.726938674	-2.680549874	-2.648886858	-3.260340857

hsa-miR-4313	hsa-miR-4322	hsa-miR-4323	hsa-miR-4327	hsa-miR-4417	hsa-miR-4419b	hsa-miR-4429	hsa-miR-4430	hsa-miR-4433a-	hsa-miR-4433a-	hsa-miR-4433b-	hsa-miR-4433b-	hsa-miR-4436b-	hsa-miR-4442
-6.003332181	-4.542743924	-3.993929468	-2.005096855	-0.505525295	-3.754007687	-4.095298926	-3.594402016	-1.116439723	-3.766792671	-0.683056246	-4.299423641	-3.810023337	0.547777309
-5.902726323	-4.154453495	-3.934175722	-1.631783276	-1.033794459	-3.95774281	-2.713490239	-3.662599591	-1.702942679	-3.422793146	-1.216918856	-3.646738605	-3.993030579	-0.572008923
-5.973485562	-4.154864377	-3.796944009	-1.798710819	-0.6104071	-3.833213055	-3.349466347	-3.252443511	-1.384869685	-3.685150812	-0.848854996	-4.009450137	-3.837293	-0.381863051
-5.853065915	-4.079930006	-3.946784016	-1.704243683	-0.843313322	-3.805597555	-2.88697063	-3.435707179	-1.554995079	-3.788770648	-0.862856166	-4.138260864	-3.940455977	-0.021094397
-5.396549468	-4.184351898	-3.801805105	-1.740119527	-0.44073403	-3.836543645	-3.084209298	-3.268895473	-1.184260356	-3.450185479	-0.714354533	-3.811131417	-3.61183277	0.341114716
-5.429768788	-4.017549931	-3.999366452	-1.378192444	-0.92405996	-3.927466905	-2.353652833	-3.448907838	-1.698204837	-3.511828243	-1.25136683	-3.935052688	-3.865560218	-0.08105284
-5.672566808	-4.295564683	-3.819420261	-1.796885104	-0.746411524	-3.624171568	-3.379279183	-3.529848409	-1.501919007	-3.439083885	-1.036730708	-4.100437618	-3.76876223	0.05796881
-5.872077866	-4.137933031	-3.9716216	-1.564812478	-0.973070567	-4.020193198	-2.66996942	-3.297529305	-1.808069588	-3.53142679	-1.352963828	-3.459926529	-3.857729993	-0.492703451
-5.833912858	-4.341945082	-4.148019435	-1.926208444	-0.874845915	-4.02592307	-3.091358114	-3.369386233	-1.693158288	-3.751562558	-1.32779845	-4.070209469	-3.916552814	-0.349828021
-5.416445403	-4.206139371	-3.976462209	-1.602844558	-1.131301238	-3.912119775	-3.162135527	-3.518494562	-1.977240485	-3.783806667	-1.423370629	-4.121475389	-3.756443901	-0.330934894
-5.595304244	-4.351128601	-4.066937424	-1.77914625	-1.110164453	-3.824507156	-2.675930786	-3.256492101	-1.944840778	-3.760042506	-1.374252411	-3.993715916	-4.250682169	-0.37962917
-6.404799599	-4.295558571	-4.360427074	-1.696205258	-1.265575265	-3.882565959	-4.22310059	-3.555038135	-1.931140173	-4.439226873	-1.312446763	-4.924562321	-4.549688993	-0.564276839
-6.402453532	-4.569100809	-4.350582711	-1.763525947	-1.318955207	-3.995578291	-3.889039977	-3.236452422	-1.889337339	-4.122547152	-1.549702029	-4.556041152	-4.532061525	-0.655656198
-5.834845945	-4.219611977	-4.12040521	-1.598222941	-0.983536557	-4.110520012	-3.402392224	-3.643183345	-1.720835895	-3.778154301	-1.193606181	-4.270324851	-4.331710448	-0.306382802
-5.753476586	-4.374913867	-4.453012123	-1.604097409	-1.269021791	-4.27561946	-3.379389708	-3.80680857	-2.159692794	-3.904447823	-1.610260169	-4.104732412	-4.480105665	-0.254375463
-6.012448177	-4.128999478	-4.430535756	-1.531089163	-0.823362808	-3.95536198	-3.735953743	-3.197804743	-1.748265082	-4.112391665	-1.301826516	-4.481577603	-4.437170296	-0.60092633
-5.871663323	-4.056981856	-4.393169245	-1.342909453	-1.224608658	-4.055428197	-3.648454318	-3.655785594	-2.186142421	-4.345006838	-1.71748576	-4.884911048	-4.43637534	-0.435626038
-5.72610003	-4.530146716	-4.28547206	-1.987707687	-1.270468104	-3.987489047	-3.29750782	-3.774294822	-2.375249377	-3.476522186	-1.658850347	-3.822903817	-4.462257987	-0.919981028
-5.693256337	-4.265265511	-4.183457329	-1.622668454	-1.147177436	-3.66862245	-3.185515767	-3.534466291	-1.937693091	-3.91900873	-1.481975509	-4.412753226	-4.426333639	-0.125390371
-5.632287129	-4.137873065	-4.299954151	-1.594256678	-1.232391439	-3.988795987	-3.372843089	-3.368810069	-2.094892481	-3.71932589	-1.588601864	-3.904174105	-4.393711551	-0.391164421
-5.825559191	-4.254369685	-4.15441807	-1.517914072	-1.082906544	-3.911769906	-3.520790063	-3.847728284	-1.916764097	-3.564483752	-1.388920845	-3.78338584	-4.517411287	-0.378524274
-5.838844751	-4.252480786	-4.285801037	-1.618326032	-1.150646426	-4.273038591	-3.816210983	-3.765337428	-2.028093485	-3.994287439	-1.516107506	-4.266321401	-4.519531857	-0.311762183
-5.833741924	-4.446760108	-4.156844495	-1.798027984	-0.896213376	-4.110255321	-3.20254737	-3.45228506	-1.795713268	-3.681854106	-1.154302314	-3.834951459	-4.417331727	-0.302351001
-5.682632706	-3.95354224	-4.035698823	-1.528372624	-0.861607673	-3.693481422	-3.964393528	-2.808934082	-1.514389203	-3.822601021	-1.087725384	-4.364151638	-4.295624764	0.789898977
-5.910499234	-4.114372883	-4.3273342	-1.542272222	-1.184722314	-3.971272314	-3.206572516	-3.384012254	-1.936577806	-3.653391131	-1.31183371	-3.901781235	-4.396374599	-0.469058128
-5.788066405	-4.023414694	-4.192704828	-1.789718068	-0.724148153	-3.952037458	-4.124643876	-2.97143326	-1.60798739	-3.624104184	-0.960041328	-4.201629656	-4.182383468	0.987893075
-6.650925201	-4.122678678	-4.329663218	-1.882133947	-1.508951252	-4.429692551	-3.325155038	-3.548003266	-2.151196115	-4.095399342	-1.670995162	-4.299253575	-4.557977042	-0.638319898
-6.26529218	-4.357917357	-4.214162169	-1.9607383	-1.241436338	-4.186407011	-4.184748719	-3.314598819	-1.88495407	-4.012943917	-1.404342723	-4.688990519	-4.277496713	0.172008198
-5.992155999	-4.288692033	-4.437302849	-1.394483553	-1.310966118	-4.117486723	-3.813254055	-4.404843836	-2.023102845	-3.879504727	-1.266200149	-4.200123616	-3.923843673	0.034155791
-5.737063445	-3.979206907	-4.161520887	-1.505558063	-1.057156696	-3.916186169	-2.95792798	-3.0065818	-1.934137848	-3.908847806	-1.184552589	-4.370515475	-4.253622638	-0.386893336
-5.596165531	-4.088755162	-4.244586322	-1.362180636	-1.255553338	-3.823689796	-3.014475477	-2.974019757	-2.052461753	-3.831593086	-1.431618949	-3.922557905	-4.066863822	-0.212648364
-7.017964724	-4.810558721	-4.876501013	-1.604937864	-2.275855214	-3.434188289	-4.412967605	-4.289794129	-2.823648588	-4.78383016	-2.229965155	-5.489014754	-5.150208488	0.320124581
-5.62755114	-3.713554861	-4.465751729	-1.02853568	-1.850959989	-3.7005105	-3.357653989	-3.391917041	-2.342720793	-4.357469911	-1.494360174	-4.732108128	-4.488307009	-0.811214058
-5.700962953	-3.037647698	-4.421325978	0.102707018	-1.40239211	-4.144022343	-1.946881558	-3.33141873	-2.279546484	-4.227008421	-1.582523643	-4.563453932	-4.235793956	-0.795265554
-6.276595526	-3.997250237	-4.667737755	-1.318626426	-1.175212766	-4.828627785	-4.192847286	-3.588857355	-2.010298837	-4.41961387	-1.340309225	-4.961322546	-4.563394383	-0.607734729
-5.443936018	-3.345818254	-4.312176008	-0.823632011	-0.666945737	-4.141667617	-3.681842159	-3.628035077	-1.59900637	-3.907587997	-0.908129108	-4.380901192	-4.123161765	-0.611188975
-4.498586399	-3.364698165	-4.89891267	-0.614326014	-1.660609874	-4.518613876	-3.943603471	-3.815257177	-2.937027289	-4.422499681	-2.270740283	-4.917876005	-5.870840027	-0.563865773
-5.560861888	-3.510221187	-4.253057238	-0.944713675	-1.129201424	-4.421263275	-3.725602062	-3.75061771	-2.170887587	-3.842231888	-1.495445534	-4.319689411	-4.10132729	-0.878003686
-6.163399968	-4.009284238	-4.132390762	-0.864950817	-0.708719862	-4.890153586	-3.776547106	-3.341229542	-1.679563018	-4.026297601	-1.153314505	-4.651526435	-4.195458065	-0.7552056
-4.975879854	-3.661954068	-4.37243961	-0.834143899	-1.515399128	-3.687852794	-3.39731415	-4.320031588	-2.550988741	-4.258587748	-1.844862217	-4.775262477	-4.121828002	-0.503064701
-5.661286862	-3.913437828	-4.122709876	-1.666203509	-0.803504384	-4.148720368	-4.821191795	-3.785111788	-1.505062217	-3.966817423	-1.133085969	-4.415410035	-4.193848554	-0.37557708
-6.132199461	-3.470628198	-4.58606518	-0.000867341	-1.321062294	-3.912439306	-2.576122671	-2.91622611	-2.830498808	-3.717478655	-1.800959005	-4.186423983	-4.405796717	-0.977039811
-5.884822648	-3.359763489	-4.949151765	0.283695493	-1.838390435	-4.206121812	-2.749675782	-3.298147648	-3.243716985	-4.369625124	-2.522644269	-4.718111346	-4.626300281	-1.338043866
-5.05120577	-3.09843584	-3.659445854	-1.728825561	0.190769881	-3.177963794	-5.704027419	-3.452331065	-0.435366621	-3.01839245	-0.099229888	-3.80305706	-3.877710842	1.464184336
-5.699296489	-3.621188318	-4.656687901	-0.055084633	-1.189216159	-4.281791676	-2.95949777	-3.142535018	-2.324461631	-4.25747213	-1.740034288	-5.11018145	-4.306028142	-0.933336793
-6.01012517	-3.837156075	-4.691812309	-0.978893765	-0.889310408	-4.779395878	-4.004410302	-3.510994595	-1.83411568	-4.275646743	-1.423800996	-4.693687532	-4.406097318	-0.728275783
-6.156847298	-4.107523585	-4.318341673	-2.145914892	-0.406749118	-4.367314231	-4.948019875	-2.483892359	-1.237814388	-3.833457214	-0.734407556	-4.565718202	-4.483192129	-0.314148196

-5.963652919	-3.703625819	-4.525083006	-0.916283435	-1.121328667	-4.350795122	-2.994088377	-3.539125039	-2.080430188	-4.079999431	-1.590741452	-4.678699396	-4.356067295	-0.832446675
-4.879328064	-3.447482478	-4.589962845	-0.135566102	-1.121144044	-3.284607303	-2.599757566	-3.67184628	-1.994125892	-3.965808452	-1.542752208	-4.319726588	-4.296517189	-0.642633411
-5.394333592	-3.912939289	-4.359525041	-1.337432642	-0.97338704	-3.81756079	-3.740727471	-3.797184544	-1.836886645	-3.930339345	-1.094157019	-4.267008651	-4.343699223	-0.489791079
-5.352944089	-3.398866853	-3.70382581	-1.553505391	-0.073758439	-3.225549347	-5.325054646	-3.300582273	-0.815958198	-3.180675328	-0.058599806	-4.046238543	-4.268894729	0.713989877
-5.85145605	-3.5761642	-4.306447102	-1.009327326	-0.976861954	-3.937646315	-4.323880262	-3.03166267	-1.78748111	-3.906871406	-1.366334809	-4.538803102	-4.363777384	0.74651879
-4.724904534	-3.413399233	-4.464925553	-0.507865987	-1.737434949	-2.731124755	-2.124861241	-3.224119025	-2.847389183	-3.986051889	-1.896660259	-4.456615529	-4.519473036	-0.797883573
-3.968971247	-3.244927704	-4.567350347	-0.858759863	-1.358660991	-2.339552282	-2.737365169	-3.725068739	-2.324532061	-3.9956504	-1.462686769	-4.022820807	-4.356148024	-0.311733461
-5.569827672	-4.135022417	-4.295441886	-1.329062155	-1.315651767	-3.851118669	-4.686412337	-3.31923883	-2.117405487	-3.962847692	-1.809364327	-4.376348759	-4.207606902	1.44865916
-5.137587608	-2.89958323	-4.670384951	0.126582458	-1.254045508	-4.155993138	-2.214124255	-3.207554691	-2.446390275	-3.949638312	-1.611645627	-4.601591649	-4.314387442	-0.702041809
-5.944278706	-2.878096449	-4.660309038	0.050667387	-1.210045304	-4.175647255	-2.369989756	-2.818108311	-2.408696203	-4.319120748	-1.641496231	-4.854193235	-4.362605763	-0.787161184
-5.761752455	-3.464859615	-4.526960038	-0.416896638	-1.9403628	-4.134490795	-2.967141687	-3.514830464	-2.996106476	-4.077694307	-2.495497766	-4.488981309	-4.718354636	-0.729854001
-6.39152997	-3.440575706	-4.625934756	-0.658566986	-1.019043288	-4.108840774	-3.211842632	-2.784442039	-2.220052946	-4.094109475	-1.55085812	-4.409233832	-4.350372996	-0.929379014
-6.295140932	-3.267194956	-4.564112179	-1.565932504	-1.402290235	-4.349719214	-4.884182349	-4.122789623	-2.188702744	-4.391255722	-1.614539635	-5.137803097	-4.507796533	-0.417601745
-5.584180943	-3.233442082	-4.636600292	-0.29879084	-1.537176556	-4.010846809	-2.83684698	-3.26632984	-2.816345121	-4.172897475	-1.930298446	-4.713102666	-4.653700905	-1.242836158
-4.671520281	-3.531887149	-4.813126076	-0.947822687	-1.920151524	-3.338123427	-2.823461786	-3.874184688	-2.915337765	-3.792668407	-1.911838182	-4.569466011	-4.893071553	-0.693406938
-5.128754063	-3.851062302	-4.403288434	-1.147040478	-1.340700793	-3.463275708	-3.414540242	-3.441261622	-2.189610014	-4.137323939	-1.420912625	-4.457580409	-4.517615443	-0.537706421
-5.331538076	-4.02211952	-4.22138547	-1.035958219	-1.245722915	-4.432833425	-3.563209996	-3.552319395	-2.212300388	-3.393444502	-1.555489018	-3.776710239	-4.072664619	-0.936274279
-5.940950942	-3.494594101	-3.913956182	-1.722888606	-0.935323294	-3.995462539	-5.248246168	-3.812235213	-1.822772828	-3.735370182	-1.245274484	-4.576057191	-3.751267211	-0.030548187
-6.172584794	-3.179290832	-4.511184358	-0.838884318	-0.562723194	-4.274463817	-3.602261485	-2.804583965	-1.613240245	-4.077663844	-0.917007445	-4.773662322	-4.521413647	-0.441976583
-5.846570658	-3.781326906	-4.801878164	-0.650120543	-1.70882503	-4.175963988	-3.143934142	-2.981706369	-2.815191628	-4.302823724	-2.103934723	-4.754399257	-4.635914827	-0.834236777
-6.226273702	-3.397851594	-4.482133978	-1.110461464	-0.802967498	-3.62885103	-3.818925093	-3.253958003	-1.871513857	-4.264118002	-1.245162437	-5.081265421	-4.104503506	-0.131751198
-6.347765242	-3.676269357	-4.702169312	-1.142511006	-1.586493004	-3.081366843	-2.8233999	-3.054714437	-2.418920356	-4.770529005	-2.012254195	-4.711203774	-4.592391518	-0.749696459
-6.941544	-4.343293467	-4.935174242	-1.530947531	-1.658205859	-3.716994152	-3.905147839	-2.546850858	-2.637868191	-4.826270647	-1.926352226	-5.200502896	-5.00834306	-0.635612147
-6.869548386	-3.758891546	-5.402263459	-1.249852065	-1.676737098	-3.826388117	-3.421495696	-3.445523022	-2.692114172	-5.034579681	-1.89361818	-5.424645461	-4.912524804	-0.591042845
-6.455006008	-4.292967138	-4.642499798	-1.638176644	-1.543775516	-4.370377947	-3.251972764	-3.361425075	-2.369446429	-4.713251373	-1.789708704	-5.002187294	-4.59138693	-0.558861925
-5.384365991	-3.678587794	-4.648226093	-1.312582088	-1.470157934	-3.486199857	-2.721231091	-3.535140747	-2.396190941	-3.965823979	-1.918688939	-4.332536059	-4.373481938	-0.758903847
-5.710306099	-3.469737364	-4.645138714	-1.031334986	-1.708320573	-2.550967043	-2.698810401	-3.103670445	-2.411935507	-4.467538715	-2.072072922	-5.011871519	-4.388396643	-0.428234949
-6.074540233	-3.829028522	-4.737529163	-1.170334225	-1.739077352	-2.960032129	-3.671920414	-3.337419229	-2.447303324	-4.86755326	-2.061447559	-5.594794818	-4.475924061	-0.290604659
-6.035290333	-3.558066073	-4.554166762	-1.294077268	-1.663578627	-3.221169775	-3.039527026	-3.266967836	-2.235079337	-4.192515753	-1.732149792	-4.328882188	-4.484148256	-0.598259565
-5.926668985	-3.786040439	-4.426358673	-1.448691485	-1.6188846	-3.184494636	-2.559297327	-3.373201739	-2.368227674	-3.898749173	-1.686794895	-4.060476757	-4.352260416	-0.724984176
-5.876985819	-3.521632386	-4.722551075	-1.126712404	-1.845174628	-2.591205798	-2.579608134	-2.971501009	-2.386157413	-4.690365255	-1.88670321	-4.856583328	-4.673227437	-0.820162454
-5.444179058	-3.854891695	-4.626621086	-1.08711908	-1.817209261	-2.874553231	-3.250616872	-3.582474117	-2.432733414	-4.400888317	-1.908560022	-5.158882201	-4.606459328	-0.448510269
-5.763874006	-3.565886005	-4.724969664	-1.025163599	-1.936953303	-2.577414158	-2.656416184	-3.048267611	-2.425352633	-4.385248585	-1.984273285	-4.334660414	-4.451938136	-0.663753521
-5.994348591	-3.452855789	-4.471432121	-1.08964813	-1.696939926	-2.655028502	-2.835446586	-3.038778702	-2.254258589	-4.446606759	-1.875668854	-4.494937808	-4.404442872	-0.508207091
-6.251613398	-3.890407968	-4.557034375	-0.772130229	-1.566132677	-4.530340065	-3.420430178	-3.394688384	-2.287639722	-4.375130252	-1.68265489	-4.836851419	-4.205657825	-1.025711343
-5.850171826	-3.500185435	-4.164186368	-0.254476679	-0.865114811	-4.453639532	-2.887321	-3.608598912	-1.946059366	-3.689762756	-1.219142994	-4.281287229	-4.228985533	-0.453703058
-5.27613552	-3.385776617	-4.235335145	-0.160927621	-1.628066257	-3.637798543	-2.253884773	-3.099823063	-2.784504659	-3.854510492	-1.823746965	-4.047884197	-4.630772445	-0.756695872
-5.106659213	-3.688621523	-4.346942996	-0.696431306	-1.319680875	-3.488614628	-2.752462734	-2.999799421	-2.323764836	-3.652261425	-1.66644109	-3.945701197	-4.290103698	-0.838682634
-5.561418532	-3.883401527	-4.625498316	-0.639761673	-1.296588058	-4.253878903	-3.428281613	-3.367545102	-2.225668011	-4.098776091	-1.678940128	-4.584349798	-4.259495345	-0.993190504
-5.335730255	-3.40660358	-4.215013492	-0.553237488	-1.355889362	-3.775051818	-3.134703587	-3.87568668	-2.140336512	-4.387600148	-1.775460977	-4.906050424	-4.164918984	0.839102699
-5.694084572	-3.28023825	-4.605849593	-0.32846718	-1.356077505	-4.257597832	-2.544369402	-3.254470805	-2.426078518	-4.085931485	-1.802357141	-4.296667459	-4.378984638	-0.950814719
-6.398950645	-3.355743317	-4.807432981	-0.378854901	-1.347152291	-4.216916394	-2.543958519	-2.869756287	-2.471126227	-4.40435604	-1.703708719	-4.670141095	-4.574365704	-0.875035748
-6.767963409	-3.159598266	-4.667123368	-0.12734515	-1.241926835	-3.863714491	-2.970282835	-2.598033752	-2.810281118	-4.422826271	-1.980539331	-4.99917999	-4.607821561	-1.031679383
-6.066093229	-3.884431177	-4.856783038	-0.793136213	-2.047255763	-4.330158838	-4.210796707	-4.16646323	-3.021317384	-4.60875577	-2.319999536	-5.228772825	-4.766067567	0.52246169
-6.310101785	-3.668846168	-4.94214373	-0.963157184	-1.825982687	-4.266136874	-4.077720821	-4.039012334	-2.537777275	-4.918960116	-1.896803293	-5.667483897	-5.163922705	-0.247963337
-5.323196146	-3.821974797	-4.651496487	-0.929209746	-1.659320064	-4.274229764	-3.27962506	-3.291915237	-2.751284037	-4.492856603	-1.868479803	-5.130091764	-4.551412722	-0.7050669
-4.936014205	-3.606183544	-4.515093591	-0.609187358	-1.243466511	-3.253232973	-2.854984821	-3.382359813	-2.012263272	-4.00757615	-1.39812691	-3.992516289	-4.479341325	-0.497829071
-5.977716243	-3.736719702	-4.598244499	-0.516558584	-1.245428288	-4.479693218	-3.113215663	-2.820246793	-2.669819096	-4.084527838	-1.770576989	-4.899506981	-4.557355306	-1.10198572
-5.791845091	-3.5188778	-4.587408015	-0.424676825	-1.439481311	-3.529564752	-2.593526661	-2.834228028	-2.569554557	-4.108226669	-1.790844536	-4.575063455	-4.186923	-0.645700883

-5.898975403	-3.463235887	-4.371873279	-1.046107126	-1.542987483	-2.543102595	-2.604321325	-3.045194032	-2.113178185	-4.336077824	-1.569921541	-4.406787335	-4.459417382	-0.51777657
-6.042998659	-3.324173863	-4.418651717	-1.100868185	-1.641875071	-2.349832739	-2.407126597	-2.799320691	-2.156561511	-3.920594275	-1.837407785	-3.897352829	-4.435118703	-0.672942014
-5.547060437	-3.269447404	-4.568589177	-0.962691076	-1.696640629	-2.519582092	-2.366732937	-2.930822777	-2.387368925	-4.383927403	-1.823191073	-4.486310967	-4.490460196	-0.595250514
-5.664982119	-3.500629612	-4.678285752	-1.1672004	-1.697728659	-2.851464772	-2.69453458	-3.111618977	-2.310155291	-3.921764844	-1.749774776	-3.939152024	-4.491883034	-0.486438879
-6.765870237	-3.672004691	-4.662265526	-1.365219951	-1.572822094	-3.31932099	-2.664472759	-3.252642974	-2.229794425	-4.166642415	-1.860841276	-4.324519064	-4.482495178	-0.71899559

hsa-miR-4443	hsa-miR-4446-3	hsa-miR-4447	hsa-miR-4448	hsa-miR-4449	hsa-miR-4454	hsa-miR-4459	hsa-miR-4462	hsa-miR-4463	hsa-miR-4467	hsa-miR-4472	hsa-miR-4476	hsa-miR-4478	hsa-miR-4479
-3.465627149	-1.984601773	-2.868802012	-5.397123664	-2.880910729	-0.415528881	-1.211514432	-4.452469367	1.861806596	-0.305768946	-3.436784822	-3.843670517	-3.307592721	-5.808526932
-2.402205971	-1.893175262	-2.990217121	-4.841787192	-2.826595414	-0.869948659	-0.795539074	-4.328081757	1.799292195	1.344288817	-3.369916025	-3.632332351	-3.365227754	-5.145485851
-1.496544353	-2.283707403	-2.862494194	-4.419241124	-2.71754173	0.405089715	-0.521787878	-4.383201862	1.762597388	1.543611183	-3.271887492	-3.191916889	-3.227846767	-5.197750511
-1.589308001	-2.332086386	-2.790434303	-5.261454319	-2.879997845	0.225993017	-0.216694389	-4.32389311	1.721508188	1.433136317	-3.122174043	-3.274767302	-3.259144217	-5.30792438
-2.067612977	-2.368446809	-2.681621151	-5.343468629	-2.886629374	0.092814498	-0.39702224	-4.615857019	1.972304283	0.979150138	-3.104265725	-3.440166335	-3.258565939	-5.521495391
-1.237940425	-2.031992309	-3.145201183	-4.993254013	-2.896255077	0.464712003	-0.636011301	-4.495662578	1.957591542	1.197282996	-3.394623776	-3.331760482	-3.492516038	-5.301011372
-2.146534878	-2.252771995	-2.80722578	-4.774370443	-2.722317999	1.135226055	-0.458951855	-4.360821282	1.925610229	1.299420255	-3.394713874	-3.130078478	-3.27030545	-5.126759443
-0.899490829	-2.116775677	-3.190654776	-5.045547809	-2.778530638	0.485665273	-0.111806801	-4.387123684	1.672529194	1.808800905	-3.525124906	-3.239696894	-3.39091321	-5.244085343
-1.983635609	-2.392552666	-3.06008184	-5.262150522	-2.93735092	0.119125602	-0.426981266	-4.60972959	1.732202279	1.639215963	-3.485425314	-3.057429383	-3.509919268	-5.25855566
-1.694006075	-1.788671164	-3.199827859	-5.033377601	-3.059365658	-0.369285524	-0.786462112	-4.277232725	1.758554164	1.089412144	-3.552802766	-3.637254195	-3.512592455	-5.103121726
-1.664631846	-2.484263451	-3.302873434	-5.062826808	-2.709100585	1.233075133	-0.418491961	-4.583032202	1.549755955	1.266589994	-3.486038922	-2.970639022	-3.414372494	-5.498676401
-2.918803527	-2.122569094	-2.794403028	-5.310397905	-2.790199536	-1.34112926	-0.936907794	-4.685144734	1.523998864	1.197387475	-3.34618876	-3.401550396	-3.493831932	-5.414754384
-2.537297584	-2.305237588	-2.997875817	-4.835201153	-2.883264199	-0.260950425	-0.589308361	-4.568856443	1.430824724	1.479460156	-3.365517605	-3.434315607	-3.538530316	-5.700807525
-1.889053453	-2.424938032	-3.197426022	-4.65934721	-2.889130392	0.288794089	-0.582237778	-4.457307644	1.691363488	1.051305218	-3.654342312	-3.502534315	-3.395285143	-5.436874798
-2.583083533	-1.95928472	-3.514299545	-5.260222753	-2.895189871	0.005863455	-0.94090198	-4.829558923	1.734496966	0.839264165	-3.852389519	-3.525129218	-3.850925573	-5.44269724
-1.964382546	-2.389379084	-2.981490178	-5.496411741	-2.788191755	-0.383952637	-0.557760721	-4.664424449	1.514739566	1.612460397	-3.392601213	-3.313012217	-3.535950276	-5.290669881
-2.334018633	-1.859831866	-3.330536061	-5.398552273	-2.980220266	0.005210877	-0.848864295	-4.432429375	1.691053595	1.078114149	-3.81452525	-3.185476918	-3.743452605	-5.180097806
-2.081752796	-2.446803561	-3.688111676	-4.681624839	-2.889438113	-0.322137817	-0.726040874	-4.636678181	1.327359801	1.603004202	-4.042905082	-4.005127554	-3.748964855	-5.060543291
-2.067355419	-2.425817793	-3.147166917	-5.570754259	-2.972512445	0.323987763	-0.594805127	-4.442072515	1.716974192	0.865075255	-3.597702001	-3.607221991	-3.484707886	-5.398536214
-1.764717193	-2.267672453	-3.336695883	-5.57073482	-2.722232971	0.510624242	-0.455711485	-4.541680273	1.668125217	1.230035274	-3.768590423	-3.227431131	-3.678287072	-5.359626089
-2.546235537	-1.851777217	-3.359377374	-4.23117261	-3.006302215	0.239667072	-0.879958961	-4.7847157	1.820618125	0.959636625	-3.799034021	-3.459212927	-3.641420553	-5.310014881
-2.745362525	-1.895502837	-3.278443631	-4.837531914	-2.936390455	0.534446947	-1.146896289	-4.898338945	1.767516939	1.654902725	-3.956686703	-3.299713436	-3.883848438	-5.341098851
-1.906468704	-2.50683668	-3.256977725	-5.387074028	-2.954261221	0.375297359	-0.214498721	-4.76663197	1.645902512	1.753231402	-3.497876203	-3.092549844	-3.599655655	-5.367525969
-2.238127433	-2.190917819	-2.374802044	-5.495640301	-2.750041961	-0.151010533	-0.091414089	-4.156240947	2.065421808	1.124236429	-3.059295597	-3.166068	-3.323847286	-5.493504636
-1.531283349	-2.061549943	-3.032950754	-4.761737603	-3.077971143	-0.451363525	-0.390246815	-4.637379244	1.650354567	1.537923518	-3.572940167	-3.287862973	-3.654179232	-5.142866377
-2.247107432	-1.994963852	-2.727329052	-5.182679189	-2.992334887	-0.114944067	-0.293215042	-4.387634162	1.957332522	0.871271378	-3.430868711	-3.093182227	-3.501133046	-5.322281736
-1.860532543	-2.245079525	-3.096600303	-5.20916389	-3.226648643	0.16126181	-0.562599453	-4.898197771	1.467122466	1.269764802	-3.572817944	-3.11312216	-3.726295517	-6.050569811
-2.688567479	-2.200508993	-2.969729978	-5.38044868	-3.155063991	0.326156359	-0.610994805	-4.582322337	1.79626033	0.709581368	-3.591063893	-3.034227838	-3.766139969	-5.993670702
-3.274365064	-1.745481895	-2.988925996	-4.342923151	-3.319791773	-1.089650463	-1.122674923	-4.44198637	1.615361006	0.336972359	-3.64646929	-4.838874384	-3.663975324	-5.187113944
-2.144462592	-2.434671571	-2.955703591	-3.477348369	-2.958348537	0.017166054	-0.24780113	-4.698990808	1.58890966	1.538032444	-3.215873593	-3.416670534	-3.26621115	-5.259830775
-1.854746909	-2.601694893	-2.692588689	-4.846627013	-2.90295057	0.042581679	-0.243378432	-4.561578582	1.500378087	1.636945492	-3.351220252	-3.298427847	-3.346298185	-5.303274237
-3.27984082	-1.579951197	-3.6394147	-6.125899889	-3.628181272	-0.466432975	-1.71389652	-4.733538226	1.099061173	-0.83949818	-3.847468794	-4.532793787	-3.982454635	-6.68295796
-2.551787742	-2.138950235	-2.803572511	-2.723526682	-2.925399256	-1.224341583	-0.96584048	-4.418596025	1.144355222	1.432908964	-3.044443783	-3.729744695	-3.94885209	-5.20546183
-1.982123239	-2.207727024	-2.741059255	-4.458721523	-2.391351512	-0.634928387	-0.429179918	-4.164830291	1.706898484	2.060382898	-3.742746919	-4.296601117	-3.864508633	-4.608578408
-2.336137006	-2.525214961	-3.383495899	-4.691468835	-3.206324351	-1.953398886	-1.2203938	-4.825205102	1.404232056	1.232076937	-4.106799259	-4.291355201	-4.233311254	-5.429097531
-2.756319708	-2.022449566	-3.007075681	-5.730418337	-2.530758145	-1.529653477	-0.967532945	-5.006387943	1.913487535	1.539374	-3.191881716	-3.96363944	-3.974777627	-4.734794831
-2.894415728	-1.89685868	-3.786616268	-5.772485762	-3.005590611	-2.113017234	-0.753238201	-5.28541552	1.331873511	1.390688089	-4.609098719	-5.111065924	-5.971533431	-6.003596244
-2.005603647	-2.025245801	-3.595568004	-2.685161734	-2.818396334	-1.420952568	-0.715504846	-4.410487043	1.243306773	1.990628841	-4.28240678	-4.617970739	-4.404340163	-4.896509776
-1.390754005	-2.662989234	-2.559483467	-5.329565619	-2.740107913	-2.25367199	-0.398623039	-4.71656018	1.459330897	1.645151346	-3.505477758	-4.060669863	-4.238889781	-4.988272907
-3.453752635	-1.808448527	-3.197368004	-2.62733527	-2.834286038	-1.912936528	-1.04096701	-4.576814812	1.458681514	0.833154	-4.169457105	-4.898260886	-4.596405309	-5.55324738
-2.749870958	-1.862466113	-3.254231244	-2.639977242	-2.976566871	-1.616927795	-1.07674501	-4.882569374	1.675561417	0.478170308	-4.098243035	-4.429616421	-4.200311372	-5.20678048
-1.358018468	-2.945133845	-3.490712856	-3.695074399	-2.174425758	-0.298377544	0.459157157	-4.272506925	1.619577583	2.027319632	-3.978733737	-4.162452042	-4.21990203	-5.512086632
-2.32069855	-2.958060793	-3.525277305	-3.260446564	-2.686199307	-1.334280021	0.345688475	-4.411660618	1.127919056	1.787749475	-4.446682353	-4.747649211	-4.357799237	-5.79068731
-4.234910003	-1.962658574	-2.632872248	-4.484591734	-2.74261875	-4.216702595	-0.858331953	-4.273341018	1.979916607	-0.591753959	-3.540371994	-4.744428959	-2.90193598	-5.407950093
-1.495186552	-2.401630228	-2.910870715	-4.197713304	-2.625606757	-2.121494446	-0.039012048	-4.661961669	1.633064574	1.847728955	-4.052843934	-4.089621391	-4.186545289	-5.413348633
-2.281212071	-2.190884004	-3.371701586	-3.830004567	-2.836053971	-1.676534605	-0.358674702	-4.557348533	1.305154	1.659743585	-3.951965781	-4.866771377	-4.31888708	-5.179366677
-1.651596867	-2.626961276	-2.550155093	-5.943799862	-2.359070429	-3.31822821	-0.81946675	-5.177280013	1.385819961	1.825410937	-3.300083682	-4.100829125	-3.985304492	-4.995115591

-1.890824542	-2.562477304	-3.286521062	-4.396667408	-2.459310843	-2.002698663	-0.292498193	-4.535891965	1.290705972	1.709106726	-4.013862224	-4.009251397	-4.103431746	-5.098267801
-2.91289034	-1.107011647	-2.86906545	-3.122797941	-2.763321088	-0.612007357	-0.420584546	-4.179194226	1.418549077	1.830893647	-4.22961815	-4.405910905	-4.232078829	-5.38292265
-2.208900401	-1.549234574	-3.001474784	-3.588016785	-2.702530869	-1.363418005	-1.267101899	-4.720729122	1.506369562	1.143182046	-3.452047834	-4.177749763	-4.13114512	-4.843338066
-2.849288159	-2.326268094	-2.790255627	-5.950513612	-2.513180926	-2.78397797	-0.496979263	-4.571727553	1.509255378	1.042952521	-3.558769034	-4.104770076	-3.063550363	-5.365252669
-2.381975628	-1.873505221	-3.086121224	-5.690579017	-2.754452739	-1.880852069	-0.262896408	-4.702231531	1.835628345	0.75356381	-3.545087348	-4.852413625	-3.927232084	-5.803717758
-2.538710465	-1.829056259	-3.129306782	-3.263478552	-2.397703499	-0.881826831	-0.567684521	-3.798965171	1.291287816	1.447332564	-4.4907596	-4.050371398	-4.380547608	-4.9690736
-3.021336586	-0.963084915	-2.788594235	-2.343933541	-3.021544989	-1.059544178	-1.28879474	-4.436577246	1.401875662	1.455034238	-4.192741241	-4.854633382	-4.26023908	-4.503561142
-2.063007794	-1.520908932	-3.010038916	-5.420799426	-3.146607127	-2.915850079	-0.891531942	-4.68905942	1.825282248	1.245411351	-3.773825964	-4.541619569	-4.185099837	-5.417096769
-2.016081247	-2.154858003	-3.059754627	-3.395392701	-2.29467833	-1.670596837	-0.042373633	-4.290335432	1.543347526	2.018070319	-3.88129214	-3.789768272	-4.165140991	-4.985152371
-1.682937796	-2.579597707	-3.157029443	-5.444065801	-1.983820279	-1.028317015	0.271854412	-4.293544472	1.300393887	1.963041095	-3.559375092	-4.132437012	-3.97200888	-5.265599044
-2.420879488	-2.333375061	-3.906640204	-3.108799384	-2.219387112	-0.169532691	0.190480537	-4.271917598	0.989030417	1.550375791	-4.215835785	-3.655794079	-4.576142892	-5.617721696
-1.362831957	-2.323953452	-3.354954703	-5.037325654	-2.498552557	-0.260160783	0.335667719	-4.257371069	1.297178424	1.731832177	-3.656250617	-3.757954642	-4.040517398	-5.279069509
-3.951431166	-2.067402312	-3.243404277	-5.05465165	-2.958056156	-2.038524424	-1.166888418	-4.878103608	1.455294045	0.338840441	-4.562345964	-5.15656024	-4.267981403	-5.683283169
-2.131323964	-2.621256942	-2.774787849	-2.918682446	-2.320774373	-0.407634241	-0.071960875	-4.095287406	1.136939209	1.352277996	-3.803249629	-4.631161012	-4.250049707	-5.775200124
-3.501420805	-2.041465721	-2.364918556	-1.63057213	-2.758215151	-1.178530108	-0.628302861	-4.22135455	0.847123555	0.988513868	-5.089593976	-5.242960739	-5.210916219	-5.401317167
-2.655006849	-1.745182836	-1.795076988	-1.309499821	-2.916427111	-1.712925504	-1.19968335	-4.518216839	1.179937157	1.38007617	-3.856729869	-5.148010272	-4.427345485	-5.390164703
-2.321093494	-2.583852589	-2.959409904	-4.074295198	-3.030874338	-2.128819076	-0.519171416	-4.754004426	1.393215172	1.584923273	-3.699360989	-4.252309021	-4.605618267	-5.47494552
-2.977038141	-1.970663191	-2.808717393	-5.512893947	-2.918840381	-1.577270634	-1.109377052	-4.270983525	1.984146844	0.655632511	-3.569063698	-4.788077758	-3.597947014	-5.403973508
-2.115737237	-2.39190229	-2.347331429	-4.495651934	-2.418470406	-3.216516742	-0.544237006	-4.426878305	1.497107239	2.090459736	-2.73516266	-3.768148154	-3.741923372	-4.889724167
-1.585997752	-2.806574376	-3.629834217	-3.207609383	-2.915000244	-0.973573406	0.246225357	-4.393349609	1.254805465	1.875177346	-4.141290501	-4.23224814	-4.051311407	-5.461763249
-2.772290678	-2.240063247	-3.028613731	-4.592735113	-2.703843642	-0.822616659	-0.179819351	-4.675459261	1.863610029	1.345950801	-3.734860762	-3.601657595	-3.49953002	-5.313722608
-2.483755294	-2.081836927	-2.673074586	-3.219906192	-3.430670848	1.443122982	-0.434960301	-3.75087007	1.07273431	0.82766509	-3.584629113	-2.781477017	-3.741427039	-5.874137949
-2.629738137	-2.858004695	-3.224746084	-6.415809225	-3.114177201	-1.918827095	-0.722216328	-5.045123297	0.861660887	1.222237893	-4.011768094	-3.795709905	-3.746467219	-5.756343265
-2.832196758	-1.794371488	-2.591269187	-2.782296041	-2.961239238	-0.877393721	-1.03102518	-4.907796653	0.926734434	1.350540725	-3.63264035	-3.892576263	-4.407513113	-6.09672277
-2.602881667	-2.076042016	-3.400421323	-4.278870243	-3.273729954	-2.215863454	-0.977265322	-5.041789874	1.212949707	1.212552505	-3.549130612	-3.966117925	-4.244142181	-5.598953215
-2.368525374	-2.017142019	-3.037130168	-4.980580176	-3.349340412	1.570483199	-0.337618509	-3.931629703	1.410214939	1.547667027	-4.250302389	-2.663871338	-4.259810157	-5.647735784
-3.315333208	-1.373032972	-2.739033404	-4.408031311	-3.720191933	0.742397044	-0.531875678	-3.228367059	1.374815542	0.08784934	-3.591094296	-2.716632653	-3.801117071	-6.432985434
-3.628505613	-1.365571455	-2.846416973	-4.907340999	-3.292038169	-0.115388225	-0.711043801	-3.86032186	1.602556153	-0.035535305	-3.672756031	-2.316210495	-3.723401221	-5.413714559
-2.627881768	-1.861805004	-2.865044043	-5.045564962	-3.410733606	1.007095543	-0.462085327	-3.704206368	1.313244378	0.626148988	-3.403755275	-2.51025741	-3.73807526	-5.601293872
-1.443015606	-2.244629636	-3.024261797	-5.176019074	-2.929924548	1.546025558	-0.248028538	-3.973530679	1.360532891	1.387597328	-3.800222646	-2.650287841	-3.720395125	-5.459112395
-2.541355946	-1.774664851	-2.767299132	-4.785455394	-3.539136111	1.202731195	-0.434425083	-3.20884475	1.031510874	1.041967921	-3.953677902	-2.889276997	-3.781842111	-6.427590163
-3.282591785	-1.40542384	-2.990752349	-4.014779064	-2.993157688	0.829777488	-0.780910932	-3.728220238	1.494469474	0.304153013	-4.141081133	-3.206585677	-3.968744875	-5.469021781
-2.331241898	-2.012408244	-2.644651659	-1.760083269	-3.421111875	2.527696183	-0.503140941	-3.505919364	1.209412999	0.721419968	-3.570814659	-2.982691563	-3.438523406	-6.263729999
-2.354508139	-1.310245826	-1.674306588	-3.599147152	-3.680135911	1.319960469	-0.484471522	-2.991504476	1.203545863	0.683174431	-3.092018454	-2.966847185	-3.620029928	-5.94686011
-1.787072748	-2.518544537	-3.693282468	-3.495553749	-2.7234751	-1.101142982	-0.656759248	-4.678329115	1.22268924	1.40554595	-4.329379993	-4.634216211	-4.364157505	-5.403502838
-1.908697089	-2.274646239	-3.089169973	-4.067685101	-2.432587744	-1.833745394	-0.526841325	-4.504182518	1.760348607	1.969124564	-3.738920987	-4.15432162	-3.995690422	-4.639207342
-2.256472587	-2.408495173	-3.381257689	-3.097939155	-2.37053965	-0.613130338	0.331458814	-4.098185343	1.221188662	1.826730716	-3.834542098	-4.148103612	-4.487192445	-5.309104162
-1.610839032	-2.622123567	-2.990755127	-2.895309652	-2.785241503	-1.239652548	0.144134835	-4.237075637	1.199477994	1.711741756	-3.886105095	-4.321839708	-4.239395433	-5.344284371
-1.483502131	-2.284323101	-3.034412742	-2.250815403	-2.873211406	-3.029519927	-0.561923548	-4.983043823	1.202416553	1.639065741	-4.267997805	-5.067356007	-4.455551813	-5.081334653
-3.252057634	-1.426520605	-2.7953662	-1.408646424	-3.30352462	-2.239151189	-1.380746331	-4.318046575	1.311217201	1.000187395	-3.895829109	-5.28045996	-4.961114541	-5.167903432
-1.263199762	-2.554755216	-3.3846803	-5.230518383	-2.544685456	-0.447951842	0.166364087	-4.419910606	1.278861972	1.895038035	-3.855975318	-3.893550223	-4.387612737	-5.361461444
-1.657201121	-2.547285979	-3.599585246	-4.991672475	-2.182931215	-0.482354955	0.680172623	-4.525381041	1.075073252	1.587416274	-3.792287088	-3.837828881	-4.014496464	-5.60654218
-2.30315897	-2.790786931	-3.053806991	-2.932172033	-1.84283561	0.455490135	0.972745042	-4.102178429	1.228366965	1.790502436	-3.638045496	-3.819599863	-3.735522579	-5.669988428
-2.982611797	-2.057292237	-2.731700224	-2.801446963	-3.332313177	-2.219629382	-0.695754732	-4.680401339	1.403533956	0.427020829	-4.035726904	-5.007341111	-4.591747175	-5.276880321
-3.357258266	-1.789929427	-3.491735289	-4.614825651	-2.702866023	-1.974060909	-0.924985508	-4.899328002	1.381681286	0.389337218	-4.403605298	-4.897997308	-4.675976401	-5.568263314
-2.851813889	-2.286421045	-2.910168445	-1.599109715	-2.446910188	-2.750690707	-0.115278328	-4.144531177	1.064717772	1.697518063	-3.849264287	-4.696146393	-4.78560101	-5.620529996
-2.119261897	-1.861692003	-2.351992629	-1.88153902	-2.800081264	-1.498138881	-1.16392136	-4.672570047	1.459426803	1.178661918	-4.074044521	-4.677842947	-4.947449314	-5.318433783
-1.544445901	-2.666993805	-3.661227171	-5.441417203	-2.458051028	-0.606820258	0.623271082	-4.208916311	1.2460059	1.823206781	-4.102681509	-4.342467423	-4.256678899	-5.299016732
-2.164705802	-2.042957434	-3.056180397	-2.542947789	-2.442674701	-0.22110378	0.336273847	-4.307009839	1.244981805	1.751869729	-3.89706465	-3.601489845	-4.023315946	-5.828206024

-2.50145033	-1.594688583	-2.619661371	-4.233114446	-3.381720111	1.355969393	-0.322692863	-3.241842031	1.390704439	0.825345011	-3.306834365	-2.561427417	-3.570760394	-5.951834823
-2.34726249	-1.697560539	-2.634054113	-4.310004512	-3.514546983	1.300801965	-0.180834832	-3.096636777	1.123043344	0.90950345	-3.328731316	-2.576630936	-3.650047499	-5.944693185
-2.037234703	-1.850132647	-2.725075981	-4.415720462	-3.387226683	1.527993777	-0.12204742	-3.46476836	1.185313204	1.436089593	-3.492581528	-2.468604357	-3.560013422	-5.859169099
-2.522352423	-1.580178158	-3.019522893	-4.608756109	-3.438513322	1.156075826	-0.389536273	-3.620088893	1.258801405	1.059405578	-3.585087794	-2.568188343	-3.683186342	-5.682040657
-2.385946706	-2.13236081	-3.099584851	-5.217701118	-3.413975978	1.131122992	-0.212483175	-3.859980068	1.188198904	1.041073489	-3.545437246	-2.449819808	-3.693507548	-6.016895502

hsa-miR-4481	hsa-miR-4483	hsa-miR-4484	hsa-miR-4485-5	hsa-miR-4486	hsa-miR-4487	hsa-miR-4489	hsa-miR-4492	hsa-miR-4497	hsa-miR-4498	hsa-miR-449b-3	hsa-miR-4505	hsa-miR-4507	hsa-miR-4513
-4.21085149	-3.915225616	1.676465511	-1.405926503	-2.161982515	-4.957529369	-4.541233558	-0.274551823	0.126074737	-4.105410003	-4.084821513	-0.326712809	-2.627994234	-4.209916877
-4.138697194	-4.030666095	2.054397126	-0.135269085	-1.711142228	-4.771149675	-3.727288193	0.173450995	0.973033458	-4.334830415	-4.254315112	0.126266662	-2.783385155	-3.58519657
-3.767697009	-3.888769445	2.105530412	-1.089361993	-1.689145699	-4.864911025	-4.404511441	-0.169235415	1.212445451	-4.062048605	-4.112127741	0.059258298	-2.73903077	-3.308901032
-3.288941539	-3.92818999	2.209167361	-0.48722519	-2.000035288	-5.107594091	-3.644838841	-0.139955755	1.36362758	-4.325323442	-4.233106952	-0.019873926	-2.755099261	-3.203584457
-3.887078078	-3.790068834	2.412137768	0.438412753	-2.070560932	-4.693387507	-3.239237913	0.148325855	0.912527099	-4.074754616	-3.734158859	-0.12002323	-2.677967498	-3.629152974
-4.038229862	-4.331433423	2.224844275	0.829764493	-1.930593172	-4.632176866	-3.086443946	0.589353512	1.07736136	-4.4739825	-4.14934301	0.107809146	-2.783545237	-3.40931605
-3.242246783	-3.831845753	1.921060803	-0.577164099	-2.066296608	-4.96358849	-4.347816453	0.078298803	0.656239681	-3.902557437	-3.910243767	0.010355379	-2.693690381	-3.520307887
-3.431194165	-4.28290035	2.463118512	0.300850262	-1.823744484	-4.664957589	-3.661236256	0.341698434	1.385237946	-3.961688641	-4.184921599	0.065249311	-2.804236615	-2.965832151
-3.747874254	-4.316931841	2.131760157	0.127419234	-1.767688533	-4.802963716	-3.549698327	0.029764997	1.218003418	-4.156026618	-4.245558547	-0.067646561	-2.808542267	-3.349464246
-3.59342866	-4.066854468	1.994493103	0.553228924	-2.095702638	-4.693002824	-3.486509474	0.603858241	1.3423069	-4.055914392	-4.08659952	-0.095479814	-2.735918285	-3.366339222
-3.16880654	-3.700676174	2.051391945	-0.164746477	-2.108438986	-5.134224814	-3.449746365	0.068978802	1.387087701	-4.595111176	-4.442809857	0.061796762	-3.067819392	-2.610000811
-4.017550644	-3.626338205	1.662503447	-0.645758924	-1.850168469	-5.078065064	-4.112032651	-0.376293	0.902066303	-4.56574904	-4.717793702	-0.114111724	-2.913342207	-3.896436169
-3.452603421	-3.596301718	1.876176397	-0.143155095	-1.776448755	-5.001333873	-3.367087866	-0.410055543	0.957759157	-4.493605673	-4.784744499	0.046834574	-3.081677827	-3.479806984
-3.38958839	-3.898506484	2.184887683	-0.508564146	-2.357230351	-5.064666272	-5.982645975	0.255549568	0.969507489	-4.773941675	-4.600583278	-0.000999296	-2.921165056	-3.490709864
-4.100527104	-4.410427367	1.882436775	-0.64213568	-2.237998003	-5.032562234	-4.090212923	0.532833929	1.032996892	-4.798559324	-4.692310398	-0.071350795	-3.071378889	-3.770219309
-3.116307399	-4.050699913	1.93293576	-0.974758391	-1.770117987	-5.076018222	-4.188479511	-0.143895916	0.919321887	-4.587572337	-4.693647687	0.152215381	-3.028186371	-3.269268358
-4.0641304	-4.378872969	1.772750636	-1.311737372	-1.977284971	-4.956547416	-4.824223379	0.428801769	1.235570831	-4.767476165	-4.744069989	0.140445833	-3.007430709	-3.652527457
-3.417227917	-4.091439518	1.652818748	-0.655873023	-1.819331558	-4.922982094	-4.94250957	-0.123651673	0.987216461	-4.805511362	-4.768679944	-0.062548623	-3.355352668	-3.697231346
-3.378187002	-3.666314808	1.952631197	0.220349679	-2.018042725	-4.884507568	-3.543835736	0.089213815	0.942910799	-4.674500577	-4.635298182	-0.063016147	-3.104378281	-3.538615207
-3.362248216	-4.15358744	1.953363096	-0.576009864	-2.002086245	-4.926747735	-4.27074898	0.221844085	1.445709316	-4.570241424	-4.826078638	0.041699165	-2.989682854	-3.404968189
-4.032599687	-4.42917951	2.03503303	-1.05835831	-2.200877785	-4.930901724	-4.216396134	0.456137998	1.024777966	-4.891748574	-4.733090141	0.058119239	-2.777433828	-3.871232333
-4.36225749	-4.395989677	2.10400666	-1.310703034	-1.789305164	-5.137111592	-4.301354307	0.473430684	0.875396879	-5.004414637	-4.84328628	0.105855795	-2.980577947	-3.76445431
-3.770313475	-4.043766628	2.180657033	-0.136342249	-1.650249043	-4.944095393	-3.830554139	0.028695948	1.367727489	-4.661779333	-4.721941735	0.122676494	-3.039213929	-3.130400711
-3.03124352	-3.47983439	2.201905053	-0.439358975	-1.85544498	-5.161121317	-4.051724914	0.214963283	0.947439272	-4.320057946	-4.516033553	-0.110310637	-2.812302939	-3.43096012
-3.799815173	-4.461956403	2.140221446	-0.478113425	-1.949738536	-4.978609839	-4.38055156	0.378871153	1.602492438	-4.816420627	-4.845010547	0.123688969	-3.207096494	-3.357843981
-3.739131125	-3.917917168	2.25433661	-1.941320361	-2.073131462	-5.157840676	-4.7415525	-0.012765123	1.329969253	-4.489936475	-4.667879757	-0.156357568	-2.874919766	-3.363934897
-4.030755251	-3.984590265	1.918075356	-0.101765764	-2.041638257	-4.937386272	-3.255963135	-0.212668417	1.268687127	-4.853469834	-4.814650765	-0.144532505	-3.250175995	-3.52677956
-3.917404751	-3.554869043	2.006873234	-1.042400574	-2.395862891	-5.220088174	-4.325098591	-0.167724772	0.87898906	-4.672577025	-4.567177809	-0.383889657	-3.060501562	-3.834826825
-4.768628023	-4.552564584	1.642850543	-1.226770275	-2.60718053	-5.40784269	-5.265457036	0.461555413	0.563558151	-5.037738184	-4.465324144	-0.341815202	-2.761524613	-4.339204192
-3.127800585	-4.033040337	2.37195943	0.453711936	-2.084389842	-4.894381501	-3.09952192	0.170586073	1.374043845	-4.353240508	-4.411451171	0.142177691	-2.769192843	-3.082212195
-2.680002897	-3.661653611	2.086236723	0.055809463	-1.898347664	-4.889742332	-5.295916307	-0.122764538	1.05413486	-4.129142758	-4.476942303	0.238629851	-2.890912484	-2.869419954
-4.901042264	-4.300438102	0.187847717	-2.217949583	-2.883862369	-5.767748723	-5.125514836	0.068187169	-0.838487381	-4.89143401	-5.373697457	-1.250151614	-3.644039339	-4.505800683
-3.389884789	-4.273021293	1.548327139	-2.217473119	-1.893375845	-5.106417499	-4.887987239	0.71406825	0.396360832	-4.467019139	-4.747870138	-0.190057179	-3.055303725	-3.845340101
-3.335320391	-4.546513767	1.178674772	-1.821959337	-1.522376218	-5.31375637	-4.341116854	0.870478659	1.933186037	-3.92765933	-4.624747235	1.145191183	-2.986960107	-3.213734189
-3.459699883	-4.041686258	1.689080845	-2.664030828	-2.489003427	-5.442722593	-5.854049401	0.394245415	0.306218045	-5.175404397	-5.164736783	-0.0886561	-2.998276705	-4.10597155
-4.269964578	-4.908588458	2.232176606	-2.876212894	-1.990588338	-5.351131306	-4.99167216	0.865196628	1.504416553	-4.606742994	-4.667659502	0.575454062	-2.327360049	-4.19645157
-5.231750698	-6.062173267	1.415426639	-3.11072165	-2.001436887	-6.164517229	-5.875427344	0.825661888	2.24881791	-6.024588977	-6.337556934	0.547243904	-3.668398297	-4.138409634
-3.698672491	-4.524811219	1.640931262	-2.932925288	-1.813427152	-5.123511629	-5.83853576	0.838596885	1.514964554	-4.529589066	-4.614402407	0.149631925	-3.266135145	-3.740856504
-2.931762991	-4.065224217	1.84388796	-2.757576427	-1.972557829	-5.223387172	-5.33884838	0.546497044	1.263006716	-4.365902779	-4.741503407	0.334526151	-3.035447477	-3.77285876
-4.714995589	-5.200274213	1.049467723	-3.945488784	-2.379684396	-5.219685704	-5.961979848	1.31923809	2.52373654	-4.601724316	-4.984541549	0.078072305	-3.356504138	-4.039247438
-3.854529879	-4.229274143	2.419186262	-1.977454534	-2.587068276	-5.057129662	-4.978409776	1.001120054	-0.291814969	-4.947727695	-4.795844861	-0.207333016	-2.868200631	-4.495776445
-3.16289339	-4.899860986	1.876045821	-3.251424462	-1.706153742	-4.816648499	-5.450621316	0.638761358	2.738347376	-3.86061417	-4.883824806	1.03712915	-3.313968806	-3.25470949
-2.662786282	-5.178388397	1.342566276	-2.918008441	-1.785783474	-4.455089703	-5.186672654	0.42270422	2.414846232	-3.295932727	-5.566361383	0.960868198	-3.530200862	-3.517570453
-4.510975595	-3.281392935	1.75623857	-4.100452862	-2.41563119	-4.977819186	-5.692837935	-0.215719258	-0.114893679	-5.049616833	-4.325893761	-0.734769048	-2.403351641	-4.419028937
-3.285933244	-5.165542403	1.730198471	-2.724239595	-1.95231291	-5.450880689	-5.064846813	0.813006214	2.750482437	-4.592957679	-4.945663073	0.902663748	-3.427910952	-3.265504179
-3.586074594	-4.743148918	1.413013253	-3.475490988	-1.989882632	-5.420677689	-5.738344471	0.672202166	1.679429833	-4.708513119	-5.22246819	0.240276982	-3.174870275	-3.699792691
-3.10189964	-3.913256474	1.833519116	-3.174342751	-1.607557353	-5.451220326	-5.427061103	-0.00632736	-0.365973623	-3.836680207	-4.868577685	-0.347171059	-2.649736707	-3.954981436

-2.274418512	-4.252960867	1.695516102	-1.50387542	-1.699052754	-5.225536771	-4.91759845	0.605009524	1.640778911	-4.42499918	-5.034069346	0.332744134	-3.127723172	-3.44013804
-4.327524893	-4.690992161	1.140926861	-3.238395806	-1.602237316	-4.88707923	-4.764584654	1.318675133	2.41968361	-3.91696287	-5.084650361	0.719305759	-2.926022672	-3.551588625
-3.467069269	-4.120505633	1.434791493	-2.169951515	-2.002195187	-4.932797724	-5.042648404	1.105149502	0.290455444	-4.508500407	-4.590940274	-0.133821942	-2.726161098	-3.928373864
-4.05049961	-3.103792418	1.576627842	-3.847896988	-0.732824806	-5.214388776	-5.811161033	-0.554025997	1.000036645	-4.274562701	-4.40521049	-0.694323567	-2.594940482	-3.997859383
-3.839280985	-4.382819965	1.99439655	-4.006539726	-2.349898784	-5.74378315	-5.399640398	0.369836101	1.560483274	-4.55002149	-4.729995598	0.117922439	-3.171387242	-4.043872743
-3.45255568	-4.059307235	0.811904849	-2.76536235	-1.669474576	-4.534473183	-4.484320037	0.770551294	2.448596892	-3.609857949	-5.098067503	0.488933557	-3.187440591	-3.499360207
-4.828129945	-6.604342075	1.633837433	-2.950566825	-1.757550153	-4.91486777	-5.092108257	1.448775724	1.720093967	-4.244228482	-5.185449994	-0.026471693	-2.687775689	-3.834661607
-4.63185054	-4.469481166	1.086273942	-4.018318909	-2.312766697	-5.413123223	-5.540458712	0.896722087	-0.059237586	-4.999990836	-4.696011336	-0.393495555	-3.351899096	-4.099865646
-3.44125435	-4.868551625	1.782061527	-3.032825166	-1.535576819	-5.503392959	-5.570564476	1.040387995	2.270365735	-4.2249672	-5.28619472	1.0730326	-3.209425325	-3.208081639
-2.644300151	-4.712458926	1.540618037	-2.798180565	-1.514055753	-5.27557172	-5.020870105	0.683249001	2.167245273	-3.995992709	-5.039572509	0.815904111	-3.2513529	-3.169480661
-3.836634822	-5.185339176	1.457696803	-3.288374578	-2.039093458	-5.124979878	-4.861647736	0.66657958	2.206256371	-4.398331889	-5.337837577	0.388097119	-3.853944131	-3.560769895
-3.322407835	-4.648828343	1.615826127	-3.761648664	-1.829173101	-5.11571313	-4.949386802	0.496700208	2.071352319	-4.043182226	-4.938658647	0.541357007	-3.249443719	-3.344805393
-5.104015093	-4.657476582	1.822811108	-4.395959686	-2.620676853	-5.639797806	-6.04214098	0.373796084	1.136087133	-4.745536663	-5.078575043	-0.212180476	-3.268286995	-5.022153248
-3.37849708	-4.50002108	0.804980283	-4.072042093	-2.147062994	-5.306692144	-5.632340366	0.705811773	2.154890567	-4.129679311	-5.446335346	0.404103049	-3.466357465	-3.650397082
-4.041974774	-4.525306336	0.701163964	-3.465571486	-2.046098769	-5.270071215	-6.252457885	0.786772704	1.389786504	-4.68576993	-6.223576579	-0.204558096	-3.464966285	-4.150432834
-4.351925079	-4.222928622	1.193762581	-3.827478406	-2.189128182	-5.294216494	-5.481205629	1.075305352	0.934670151	-4.400856964	-5.723643006	-0.116365582	-3.256441361	-4.008784347
-4.206048774	-4.429259312	1.964636259	-3.544555847	-2.137430672	-5.417184981	-5.790913172	0.642909969	1.781067376	-4.793624628	-4.855892115	0.161192899	-3.144915422	-3.881143014
-4.61879606	-4.104768815	2.060535251	-3.765131752	-2.223829992	-5.075565195	-5.58303227	0.222223094	0.656722842	-4.35114103	-4.067501479	-0.408301665	-2.916877036	-4.680324952
-3.016075244	-4.002476292	1.976176334	-2.359693059	-1.544353849	-5.471472352	-4.94816608	0.537976779	1.404315274	-4.205080894	-4.893329969	0.241973358	-2.91365538	-3.687165089
-2.83700591	-4.801765544	1.928259792	-2.255198806	-1.904989071	-4.878002922	-5.098177912	0.234147273	2.343852649	-4.294820067	-5.181592084	0.558383586	-3.313343096	-3.09179748
-3.891741759	-4.571013307	2.48580309	-2.690296002	-1.716371221	-4.865812076	-5.141940345	0.401316783	2.322204276	-4.431677064	-4.757888892	0.493185834	-2.57451783	-3.653032297
-3.740489	-4.158802813	1.157437602	-1.332192922	-2.319548402	-4.541865568	-3.842798851	-0.283059392	1.430819254	-4.293489967	-4.807363808	-0.016232389	-2.456197881	-3.425177989
-2.459737159	-3.893603651	1.407529839	-2.122665184	-2.169749144	-5.202036606	-4.588224051	-0.472681603	0.606590771	-4.805967423	-5.188230237	-0.121441403	-3.312623411	-3.3779874
-4.132852726	-4.307810061	1.568621567	-1.595755795	-1.865673802	-5.277491638	-3.91608461	0.18716262	1.550602842	-4.808152599	-5.413823649	-0.105268755	-2.799424163	-3.659058064
-3.233461929	-3.756768657	2.02705197	-0.551685591	-2.017989638	-4.727461873	-3.369493797	0.042829487	0.365725684	-4.814694586	-4.909866753	-0.259482671	-3.172201835	-3.876184715
-4.415447027	-4.361636429	1.828315774	-1.378230218	-1.916066033	-4.682225087	-4.141259152	0.261158355	2.198350237	-4.316482085	-5.117044232	0.089407932	-3.014158161	-3.494326212
-4.382473395	-4.029811524	1.124818274	-2.297973817	-2.539400168	-4.259515375	-3.569718455	0.110804517	1.668987898	-3.870759276	-4.407036466	0.077265676	-2.464663181	-3.55371104
-4.876648222	-4.301133358	1.602997002	-3.445045987	-2.36430026	-4.763117233	-3.978861247	0.139355108	2.197081541	-4.201278641	-4.782261394	0.261459163	-2.421332108	-3.82930788
-4.018049307	-4.176919862	1.382261418	-1.639981002	-2.325420702	-4.564504256	-3.608660397	-0.146574105	1.626433021	-4.161419379	-4.604513006	0.077701289	-2.382336457	-3.754537531
-3.791526597	-4.552964432	1.906129754	-0.075440627	-1.859842794	-4.665655423	-4.000165869	-0.039478056	2.16139978	-4.245839734	-4.740281502	-0.052105091	-2.894735252	-3.205645696
-4.02223671	-4.248725435	1.048416289	-2.393230809	-1.971393667	-4.537319402	-3.716392775	0.05915056	1.704432018	-3.786956196	-4.846342501	-0.065293129	-2.281551468	-3.503359238
-4.487954707	-4.378969141	1.479281935	-1.568453527	-2.223593566	-4.705911546	-4.317000532	0.557110509	1.720834553	-4.141754517	-5.053234491	-0.207051988	-2.895724651	-3.384367926
-3.708039523	-3.995088373	0.807007773	-1.864596615	-2.295864818	-4.379889367	-3.784257998	-0.106677847	1.293801742	-3.986799549	-4.731316738	0.01493806	-2.288621225	-3.37467837
-4.161740035	-4.113280338	1.045158854	-1.127160064	-2.273840349	-4.096768587	-3.256979972	-0.167842407	1.414952128	-2.91721255	-4.813432874	0.081264268	-2.142504953	-3.425466435
-3.343697879	-4.455786517	1.14732625	-3.70992802	-2.14343711	-5.140965799	-5.711511751	0.576267549	1.294359732	-4.264256867	-4.61532258	0.249043644	-3.554302912	-3.441332653
-3.607552764	-4.575873472	2.237258794	-2.971616512	-1.705828472	-5.216383748	-5.734380914	0.968090871	1.984978594	-4.156279712	-4.619096933	0.620862117	-2.980670664	-3.613083754
-3.046750186	-4.539735125	1.705186118	-2.745173589	-1.667552609	-5.01515935	-5.147209624	0.770912408	2.455909219	-3.822946553	-4.828761819	0.578970091	-3.42064462	-3.226899618
-3.331625268	-4.432335928	1.84221334	-1.482762878	-1.722250093	-4.778219804	-4.393782443	0.714339791	1.942150131	-4.202266584	-4.904984849	0.232027991	-3.470573622	-3.358672803
-3.547304772	-4.937258869	1.399039018	-2.347306837	-2.083912461	-5.135072355	-5.168540944	0.729681937	1.329487249	-4.686265982	-5.138077851	0.212944103	-3.355466825	-3.70273409
-5.029146217	-4.175129484	0.962645377	-3.800893549	-2.349307291	-4.770228545	-4.809359574	1.122734203	1.124997505	-4.873458905	-4.7493332	0.193208061	-3.141621279	-4.050543908
-3.306308941	-4.845717957	1.981322542	-2.920311358	-1.739792597	-4.949576605	-5.222824017	0.731256082	2.331843918	-4.11557064	-4.961927523	0.651525228	-3.419732701	-3.324992151
-2.557655173	-4.779877643	1.905644623	-2.810834548	-1.774409846	-5.381109091	-5.267622152	0.496847119	1.920345158	-3.843858283	-5.191186924	0.633564328	-3.290075915	-3.203364418
-3.120273451	-4.614297401	1.163488311	-3.425295365	-1.746126309	-5.086556136	-5.092096472	0.653620037	2.044328371	-3.917144288	-5.219896875	0.717279712	-3.273565181	-3.327050877
-4.659481748	-4.806573114	0.481528864	-4.098391136	-2.600552305	-6.120620696	-5.622348478	0.821179624	0.932227719	-4.636762639	-5.646512884	-0.125239951	-3.764988308	-4.247641895
-4.403039988	-4.503427267	1.39767804	-4.462471591	-2.503258325	-5.865261226	-5.864482379	0.861194295	1.577177667	-4.804763285	-5.611943886	-0.197870124	-3.329889318	-4.600536165
-4.062873476	-4.795670638	1.985253238	-4.007328081	-1.817533272	-5.359424376	-6.087200649	0.617306584	2.172745397	-4.153751374	-5.301813806	0.120251705	-3.654109542	-3.926924316
-3.857724327	-3.903323299	1.334331826	-2.836190909	-2.064726425	-6.523876353	-5.136012716	1.168007621	1.250317263	-4.956572679	-5.542434843	0.12217323	-2.978307823	-3.796136425
-2.741961704	-4.74075908	1.142582993	-2.613069549	-1.600918355	-5.58826786	-5.406240697	0.455216748	1.867493785	-3.795677081	-4.772573296	0.634978054	-3.410158774	-3.571818755
-3.721018228	-5.080211955	1.632144572	-2.689576553	-1.751425846	-4.724284821	-4.64225707	0.859743928	2.152496734	-4.289865851	-4.870846986	0.664057645	-3.252389871	-2.845033476

-3.892900974	-3.86322929	1.251014079	-1.601201853	-2.158475538	-4.100476966	-3.472629969	0.269941089	1.854903278	-3.809948183	-4.563332092	0.132265021	-2.175900642	-3.418916839
-3.90960084	-3.96248109	1.374943978	-0.403793744	-2.027975953	-3.866447365	-2.927040684	-0.236113235	1.784715773	-3.809266535	-4.508397158	-0.103750803	-2.390602511	-3.245433264
-3.697964917	-4.228612922	1.498653344	-1.056418301	-1.8886628	-4.158902938	-3.342748759	-0.181592799	1.895315682	-3.758611963	-4.699553334	0.126492321	-2.359745907	-3.261667698
-3.798450143	-4.179209607	1.467754969	-1.096607708	-1.967315348	-4.558994722	-3.605391296	-0.054177439	1.872810679	-3.939335045	-4.657213697	0.105539866	-2.458663713	-3.64750709
-3.654248076	-4.164764287	1.714569042	-1.048799741	-2.290200915	-4.673882437	-3.425939413	-0.200519642	2.017669702	-4.158622854	-4.927670611	-0.032254925	-2.548616052	-3.584203915

hsa-miR-4515	hsa-miR-4525	hsa-miR-4526	hsa-miR-4530	hsa-miR-4534	hsa-miR-4535	hsa-miR-4539	hsa-miR-4632-3	hsa-miR-4632-5	hsa-miR-4634	hsa-miR-4635	hsa-miR-4638-5	hsa-miR-4640-3	hsa-miR-4640-5
-7.738150331	-2.748143763	-4.301955372	-0.10252491	-0.395848619	-4.44915639	-4.416216399	-5.549578814	-0.953478461	-0.728425902	-6.015588839	-4.149672947	-4.409904867	-1.057865039
-4.650025065	-1.932421687	-4.470625375	-0.067820562	-0.710004743	-4.292791695	-4.136312772	-5.362440769	-0.30846067	-0.423666063	-5.73087661	-3.506659011	-4.891653142	-1.408012613
-6.857537361	-2.611697862	-4.249700894	0.730548316	-0.329962658	-4.541676962	-4.320438379	-5.208868015	-0.108457914	-0.610946512	-6.196981356	-3.213826919	-4.4640174	-1.287312112
-7.229108119	-2.009900348	-4.125109495	0.853237674	0.342231069	-4.484492952	-4.244883589	-5.430930389	-0.067274678	-0.70286316	-6.025199174	-3.019890908	-4.652430505	-1.461702035
-7.480375073	-2.697712265	-3.488225726	0.74241205	0.210548359	-4.270079605	-3.888706253	-5.22865179	-0.566029272	-0.559157483	-6.094994808	-3.42617527	-4.077335185	-1.190818795
-7.491406266	-2.429686828	-4.031244712	0.403972394	0.190058154	-4.200292161	-4.039081587	-5.267878096	-0.374920262	-0.425191312	-5.554185821	-3.354169661	-4.405394413	-1.385501116
-7.359658459	-2.130374709	-4.0816927	0.880296741	0.237053792	-4.367035313	-4.331079543	-5.255611622	-0.261763274	-0.432403007	-6.074689276	-3.245182453	-4.364374552	-1.276102665
-6.600878389	-2.112193314	-4.077819279	0.25748494	-0.222692604	-4.484090601	-4.115111352	-5.308544926	-0.147374851	-0.505904699	-5.600314051	-3.122565752	-4.752076529	-1.563267477
-7.589292422	-2.391912007	-4.22491054	0.63457994	-0.05252763	-4.428310423	-4.506362574	-5.40115171	-0.111596602	-0.675838261	-6.05153863	-2.89929454	-4.756557027	-1.460538391
-6.930438021	-1.997273035	-4.1486091	0.831651647	0.498382573	-4.382114027	-4.11944941	-5.348401167	-0.516458564	-0.364503761	-5.712106307	-3.60624685	-4.663000091	-1.376874351
-7.356393832	-1.877068969	-3.579123227	0.437787919	0.718650024	-4.744938708	-4.196549207	-5.646894573	-0.018168584	-0.413675314	-6.335157861	-3.397952845	-4.632274852	-1.636737696
-7.599736864	-2.29984259	-4.608435005	-0.050189723	-0.917138428	-5.016182365	-4.516696524	-5.640144204	-0.563491135	-0.712857243	-7.104994206	-3.698134221	-5.320769054	-1.329016057
-7.082496495	-2.204505408	-4.783482187	0.129346787	-0.102857292	-5.028159268	-4.635716178	-5.665757193	-0.224987012	-0.923038281	-6.447673041	-3.052816065	-5.110089191	-1.698211084
-6.932502469	-2.387001849	-4.099791733	0.572646826	0.602887779	-4.932825387	-4.461319658	-5.385766631	-0.404037646	-0.536910274	-6.257008612	-3.55008017	-4.552152778	-1.561948621
-7.0954575	-2.210359287	-4.081978134	0.11617568	-0.670295838	-4.7382422	-4.539387959	-5.735707025	-0.8097307	-0.495507285	-6.247447202	-3.763451383	-5.006464022	-1.580116876
-7.367738442	-2.333591264	-4.190743963	0.233411454	-0.129301797	-4.823418911	-4.680342668	-5.417035821	-0.151012531	-0.624568373	-6.459437371	-3.327943406	-4.854843313	-1.510221282
-7.118132797	-2.409315218	-4.372629014	-0.206779775	-1.041215115	-4.537704384	-4.617821971	-5.550875585	-0.69498763	-0.449189802	-6.077037914	-3.438787787	-5.056339509	-1.443392723
-6.383502409	-2.75336164	-4.677825356	-0.066883105	-0.702970155	-5.254789377	-4.535890821	-5.226796889	-0.263369121	-0.512920897	-6.201451575	-3.658913581	-4.804980721	-1.637662172
-7.2182973	-2.599010407	-4.060289174	0.413308087	0.242438271	-4.839058708	-4.355630076	-5.581520391	-0.256666944	-0.774900192	-6.533620073	-3.759618929	-4.604580348	-1.67352983
-7.181016043	-2.392040603	-4.032950816	0.270846393	-0.269225	-4.7470555	-4.511102102	-5.574501759	-0.246001353	-0.481389342	-5.995575278	-3.496589465	-4.722852079	-1.598035863
-6.336131011	-2.355356666	-4.186626916	-0.084692559	-0.829574877	-4.774270894	-4.535322311	-5.423481133	-0.696106117	-0.376854942	-5.601034708	-3.632468791	-4.761857986	-1.379210303
-6.587027747	-2.030918037	-4.225143007	-0.265479725	-0.888271575	-4.714878789	-4.677299738	-5.439856746	-0.76511314	-0.366712627	-6.053932569	-3.120742219	-4.678915864	-1.331017706
-6.995986267	-2.479043501	-3.904228023	0.924204523	0.39795318	-4.872604473	-4.578216735	-5.296628895	-0.078861751	-0.600269936	-6.28880461	-3.026702068	-4.497934808	-1.557619834
-7.128262167	-2.381552507	-4.014072617	0.969450056	0.02614263	-4.715429155	-4.628305671	-4.467858053	-0.056510128	-0.543056128	-6.451909756	-3.416465427	-4.359403114	-1.255719797
-6.367110204	-2.02475419	-4.212255305	0.169263077	-0.506299006	-4.79404375	-4.406927464	-5.495732513	-0.461000838	-0.333789529	-6.201758688	-3.289455904	-4.837243699	-1.409216541
-7.304863228	-2.129163813	-4.523673262	1.042834071	-0.10776604	-4.754723083	-4.890196969	-5.18501959	0.061676807	-0.64846676	-6.162460164	-3.514786762	-4.243122374	-1.077633586
-8.799716101	-1.970363898	-4.576707111	0.110729614	-0.306409344	-4.862835169	-4.406408582	-5.787456907	-0.37029148	-0.739140264	-6.56139153	-3.50697293	-5.316760861	-1.771917981
-7.001482544	-2.386815759	-4.54205565	0.516533344	-0.285897087	-4.919555893	-4.451796466	-5.415803151	-0.606649252	-0.822396001	-6.699899709	-3.878620752	-4.729354927	-1.316294476
-6.458628028	-2.55737011	-4.497471734	-0.325717367	-1.037418252	-4.907625746	-4.603848346	-5.647454473	-0.491185494	-0.26140025	-6.447754901	-4.392952272	-4.682073889	-1.491376119
-6.515879202	-2.099838808	-4.440659745	0.855327368	0.176539058	-4.657650239	-4.33748756	-5.308806208	-0.030749304	-0.403374899	-6.714820938	-3.189377908	-4.936530231	-1.529927817
-6.190184921	-1.524803327	-4.001994541	0.683467554	0.754052033	-4.507900187	-4.151153055	-5.323954336	0.021715233	-0.597757148	-6.512410641	-2.988955352	-5.019920949	-1.630242531
-7.986279209	-3.456725243	-5.241813756	-1.984301276	-1.575298807	-5.366822782	-5.175135084	-6.333671527	-1.518467673	-1.573192912	-7.138845091	-4.727367264	-5.612669526	-2.474322865
-3.920144302	-0.250742068	-5.135076936	-0.902525112	-1.134148285	-4.273883969	-4.261922119	-5.478325692	-0.578431454	-0.174737065	-6.336622614	-2.940761605	-5.148816669	-1.545020441
-5.562542406	-1.260282027	-4.430018233	-0.279222822	-2.062871015	-4.193086572	-3.401589604	-5.10603295	-0.338825989	-0.034215824	-6.31568471	-3.038914471	-5.095455865	-1.232773458
-6.625626938	-2.392305737	-4.811656662	-0.418413246	-1.463780159	-5.1029439	-4.874794585	-5.482321602	-0.684141292	-0.583314587	-6.921358822	-4.230322791	-4.854131235	-1.624253513
-6.928493935	-2.181874259	-4.477376885	-0.470564265	-2.527116619	-4.369389736	-4.446950243	-4.817160368	-0.914470065	0.231702262	-6.043230961	-3.552877201	-4.592214235	-1.01534543
-6.238042186	-2.366889706	-5.289526697	-0.834321076	-2.480140149	-5.708258524	-4.889096524	-5.959052005	-0.715080245	0.105942574	-6.350566542	-3.550273438	-4.398559689	-1.894295406
-4.758476215	-1.372229343	-4.490325009	-0.822524594	-1.953026083	-4.878163507	-4.56240966	-5.086342604	-0.527461772	-0.182853755	-5.674776748	-3.589850003	-4.641670906	-1.51849501
-6.180240721	-1.823282769	-4.88462205	0.18394646	-1.513370934	-4.981025654	-4.606229416	-5.120514787	-0.415011642	-0.513311808	-6.634121409	-3.699336827	-4.595358117	-1.45763499
-4.500650252	-0.689231103	-4.736780845	-0.854552049	-2.586701379	-4.893375325	-4.887567893	-4.780444851	-1.283576085	-0.107045113	-5.232162637	-4.345599997	-4.547938162	-1.762902712
-5.417155847	-1.793529624	-4.658653626	-0.349561623	-1.61197591	-4.839020724	-4.895853802	-5.105857675	-1.131999262	-0.196898122	-6.031551833	-4.711163869	-4.473468137	-1.269044893
-4.70562669	-0.24052449	-4.79731634	0.270502215	-1.601148942	-4.796922143	-4.058750837	-4.948998013	0.003792538	-0.48893769	-6.015606884	-3.107296093	-5.087301063	-1.814252793
-5.29734191	-1.001182986	-4.382002035	-0.396582905	-2.710548037	-4.79730046	-3.955627875	-5.497830011	-0.140464901	-0.713903321	-6.085811248	-2.783633809	-5.444056471	-2.306503001
-6.463053411	-2.489985856	-3.970037714	0.168788725	0.366969692	-4.796731434	-4.945305674	-5.205264747	-0.978036369	-0.25368882	-6.433789335	-4.745183697	-3.498258658	-0.643748751
-5.845242977	-0.706449341	-4.749290141	-0.265770504	-1.738706999	-4.906968761	-4.236021038	-5.43138028	-0.207811247	-0.436609146	-6.602236985	-3.672634528	-5.092706895	-1.566419992
-5.717629552	-1.55421271	-5.04748482	-0.306069236	-1.901649425	-4.877931259	-4.913400852	-5.188874361	-0.518720411	-0.268621136	-6.207777685	-3.940338782	-4.922698176	-1.510417524
-7.378325945	-2.063701728	-4.726976331	0.821800628	-1.071756195	-4.826273679	-5.068256027	-4.959468888	-0.281391161	-0.287958007	-6.593636319	-3.882978742	-4.421316084	-0.972384611

-6.334843325	-1.736460277	-4.911578024	-0.038332916	-1.288225134	-4.727485695	-4.426499957	-5.050137892	-0.093372861	-0.412405939	-6.271538587	-3.714999101	-4.985738827	-1.594001584
-4.863661697	0.066550556	-4.504642739	-0.726924344	-2.287206361	-4.327925128	-4.019055604	-4.97901425	-0.588848829	-0.054381216	-5.077881968	-3.273028393	-4.79171606	-1.427309634
-6.101595172	-1.40893245	-4.66027835	-0.909354566	-1.989895633	-4.630130989	-4.595005406	-4.812760598	-0.8464021	0.18912799	-5.923258782	-3.980473668	-4.390010949	-1.184666338
-7.391453612	-2.462724368	-4.246507032	0.113802957	0.112087193	-5.029784138	-4.855753986	-4.937419221	-0.496894405	-0.453227269	-6.673504517	-3.56789562	-3.969258125	-0.778261558
-7.005210654	-2.41463095	-4.410209742	0.185846627	-1.005433335	-4.037118387	-4.737167317	-5.379836145	-0.662704268	-0.73082733	-6.580244266	-3.761835319	-4.276608691	-1.599041551
-4.636620134	0.305229885	-5.232742893	-0.889504486	-1.850278832	-4.24443317	-3.885992214	-5.033683179	-0.577136389	-0.306206559	-4.52839681	-3.725566914	-5.06542276	-1.543531202
-4.187429051	-0.80193782	-5.307529135	-0.74549177	-2.314015888	-4.339052208	-4.553822363	-4.076500014	-0.529286259	-0.022802447	-4.473996406	-4.282054701	-3.923317899	-1.164760396
-6.224349084	-2.391303768	-4.620313523	-0.780519939	-1.059382792	-4.968567185	-5.053620302	-5.345901128	-0.906082431	-0.507139472	-6.17798088	-4.332137496	-4.277769094	-1.940121003
-5.55087823	-1.328130426	-4.713993578	-0.031873468	-2.191150255	-4.864309105	-4.01307455	-4.840944226	-0.031828853	0.121463783	-6.722546999	-3.367806699	-4.564189017	-1.442893371
-7.098087386	-1.748415671	-4.611079819	-0.023287383	-1.960805155	-4.77687994	-3.914932735	-4.945643969	0.236277564	-0.323131377	-6.495506639	-3.189153073	-5.042411438	-1.532796934
-4.71207902	-1.026264077	-4.80888432	-0.603533272	-2.028491753	-4.818789918	-4.078649777	-5.57100526	-0.410068756	-0.143385688	-5.58980248	-2.359608867	-4.947946358	-2.370087117
-6.149908093	-1.427642567	-4.897790419	-0.041082222	-2.160014769	-4.645880775	-4.440523184	-5.44371417	-0.218123585	-0.060935525	-5.877279782	-2.638155271	-5.155243334	-1.795603748
-6.080297441	-2.649384253	-4.933022092	-0.563797087	-2.424955446	-5.243214398	-5.038146329	-5.412574367	-1.221473387	-0.416928125	-6.308096236	-4.649707768	-4.884268304	-1.639263735
-3.652312686	0.045097206	-5.209191815	-0.628672953	-2.235877784	-5.075568759	-4.158057367	-5.063564583	-0.232972263	-0.476505108	-5.645780304	-3.551817986	-5.023200483	-2.010293001
-1.639991187	1.462054901	-5.034321852	-1.078031621	-2.196688756	-5.593103363	-5.204843475	-4.98085321	-0.640932918	-0.451381326	-5.991023266	-3.918307774	-4.388337507	-1.86345332
-3.625258807	1.197048765	-4.802730263	-1.12125991	-2.181272657	-5.274216149	-4.854215487	-5.09380282	-0.739129128	-0.069584146	-4.909827238	-4.485082758	-4.563991598	-1.504903024
-5.627821023	-1.21444353	-5.094530591	-0.317374199	-2.17791383	-4.949950934	-4.789281341	-4.810120491	-0.593417671	-0.241824984	-6.242549832	-4.024690484	-4.620271373	-1.774176932
-7.138826479	-2.675270041	-4.437271969	-0.396208748	-1.509735796	-4.77284217	-4.752165656	-4.895909235	-1.241047925	-0.236956758	-5.941999054	-4.20806972	-4.32170321	-1.375583763
-6.788637189	-1.552948471	-4.839657984	0.221485379	-1.715587966	-4.907838186	-4.479846964	-5.143334403	-0.090992156	-0.393705535	-6.970784369	-3.313434473	-4.65511467	-1.129825088
-5.931603037	-1.608343237	-4.870999204	0.186364494	-1.829024032	-4.595469104	-4.178473044	-5.547629641	-0.127917351	-0.57863197	-5.918036977	-2.994439746	-5.235273196	-1.958879712
-6.307037089	-1.391030663	-4.487575067	0.505763866	-1.367391589	-4.440311398	-4.527331312	-5.23328291	-0.582591467	-0.315256279	-5.749956692	-3.192156148	-4.986773278	-1.413032513
-6.786684091	-2.024512517	-5.464722825	-0.261353253	-0.872560537	-3.882983464	-3.967832147	-6.032377484	-0.473645927	-0.792323993	-5.337923973	-3.076993833	-5.888805461	-1.999759415
-8.132065123	-2.164598879	-5.028446152	0.101930383	0.236854185	-5.149830144	-4.574647703	-5.706357106	-0.340466476	-1.1008045	-7.395364032	-3.5784617	-5.821514796	-1.881134693
-6.916670371	-0.966151042	-5.795845345	-0.664358597	-2.19011308	-4.777114878	-4.333958209	-6.557742584	-0.735076701	-0.163305676	-6.921953011	-3.539218175	-7.432023076	-1.633564431
-9.411088704	-1.715637308	-4.985760148	-0.739175027	-0.79602837	-4.913072309	-4.201474507	-6.204042682	-0.460695092	-0.666865757	-6.793398793	-3.67854884	-5.705838846	-1.752010012
-6.541866136	-1.615299204	-5.077005413	0.101780708	-1.135934534	-4.452464734	-4.417021176	-5.220642537	-0.486006942	-0.540847766	-5.498470226	-3.309144269	-4.774817119	-1.913169201
-6.27783969	-1.612823483	-5.367832069	-0.523222162	-1.878739916	-3.554607228	-3.869493138	-5.722659266	-0.875844241	-0.9169618	-4.803913962	-3.617605866	-5.407061642	-1.81787033
-7.49166182	-2.45789571	-5.253792424	-0.635938885	-1.749459692	-3.772171526	-4.353665222	-5.71822058	-1.246685247	-0.535711879	-5.15517662	-3.960527244	-5.477922164	-1.701621173
-7.112748669	-2.231587268	-5.165779347	0.039672765	-1.089380322	-4.010846388	-3.838640951	-5.909575029	-0.666831285	-0.644414152	-5.68615314	-3.582267702	-5.61097924	-1.799915653
-7.372912491	-2.100188524	-4.612133229	0.397051223	-0.518127826	-4.558097904	-4.155300092	-5.55499847	-0.269419805	-0.303913108	-5.22963515	-3.310112106	-5.396620808	-1.925525596
-6.936281513	-2.205076171	-5.417724516	-0.157548	-1.845381616	-4.007523389	-3.633142135	-5.973919809	-0.657032584	-0.612881585	-4.778365836	-3.28977008	-5.545183554	-1.789747595
-6.578657608	-2.309256428	-5.044984489	-0.363520882	-1.643221202	-4.676895092	-4.127860567	-5.326935445	-0.99611756	-0.441453038	-4.819276532	-3.782715627	-5.016601284	-1.47745279
-6.06039792	-1.169192217	-5.440205516	-0.244541096	-1.191630577	-3.79277592	-3.869396756	-5.790610084	-0.50971478	-0.772762097	-4.779622977	-3.225678678	-5.291102268	-1.878247482
-5.583175879	-0.81540022	-5.2938459	-0.346877907	-1.343272696	-3.593424529	-3.591150984	-5.86010266	-0.485877189	-1.003453126	-4.748355414	-3.208587401	-5.83068977	-1.673863699
-5.75955808	-1.855046361	-4.447400579	-0.638749239	-2.082337662	-4.89134421	-4.277755916	-5.452053842	-0.683370118	-0.616830251	-6.186568857	-3.620881939	-5.092273731	-1.610897932
-5.093422838	-1.156485988	-4.546366726	0.16589978	-1.681763231	-4.789783531	-4.267579732	-4.919305318	-0.369169816	-0.025201695	-6.719275181	-3.684048616	-4.648356864	-1.321437863
-4.41126335	-0.309936284	-4.555696674	-0.386292783	-1.783842518	-4.732293544	-3.981266633	-4.875705198	-0.292271517	-0.181134283	-4.207672463	-3.348085702	-4.957077626	-1.856129659
-3.860309259	0.106418597	-4.796954893	-0.19491833	-1.745528518	-4.866374031	-4.462062595	-5.039651272	-0.34769066	-0.12162689	-4.92451845	-4.002417014	-4.711988535	-1.972202438
-4.568527381	-0.635512322	-5.164194431	-0.677562475	-2.323098667	-4.848227582	-4.833655643	-4.892414631	-0.503655729	-0.187692212	-5.925819933	-4.239535946	-4.920781254	-1.515567538
-3.61064073	-0.535739068	-4.910617851	-1.44123346	-1.20828904	-4.198573057	-4.204503053	-5.034183731	-0.73762063	-0.4641785	-5.571354844	-4.858072422	-4.465521908	-1.635283834
-5.821878789	-1.382845573	-4.298008832	-0.19585352	-1.933559686	-4.813404929	-3.949397553	-5.178960584	-0.284778003	0.114011493	-6.226737161	-3.351717827	-4.73340448	-1.814568822
-6.515206482	-1.423786984	-4.678008273	0.105814656	-1.947965413	-4.745762946	-4.247108785	-5.216968694	-0.146685999	-0.14781712	-6.742143986	-3.128033299	-5.357159453	-1.9301123
-4.462430619	-1.207490437	-5.016627288	-0.456828732	-2.257050944	-4.813319016	-3.936954327	-5.675274749	0.341721025	-0.707865394	-6.384146181	-2.500563194	-5.424658335	-2.032744862
-5.833302192	-1.305247443	-5.278111985	-1.406757825	-2.270302741	-5.349578474	-5.19962604	-5.673578125	-0.953800442	-1.108474544	-6.522790639	-3.226065677	-5.02699948	-2.550775023
-4.831338133	-1.419763694	-4.961640628	-0.885252502	-2.719651529	-5.240065167	-4.911171846	-5.681936478	-1.256821272	-0.60526375	-6.636459461	-3.795758065	-5.169337986	-1.711106132
-3.249792348	0.192229797	-5.850475011	-0.05685711	-1.327966434	-5.538654013	-4.982841675	-5.127921243	-0.479135463	0.012090894	-6.081764671	-3.72391266	-5.070193511	-1.922730725
-3.68970731	1.975635066	-4.935493769	-1.128098904	-1.024938753	-4.7816277	-4.520616966	-5.170460517	-0.781824937	0.03574266	-4.824743968	-4.418994512	-4.452220719	-1.52872175
-6.298783986	-1.905302838	-4.936898659	-0.154147738	-2.58348901	-5.233168081	-4.294446305	-4.992740152	0.017986676	-0.368247944	-6.877108786	-3.404976117	-4.950454416	-1.872657551
-4.563848645	-0.110074296	-4.683844304	-0.462856673	-1.940329037	-4.614128859	-3.709300258	-5.715823368	-0.467188156	0.037713533	-5.059091287	-3.276939851	-5.449556322	-1.868765558

-6.767137213	-2.014005139	-5.725423734	-0.186716239	-1.569322172	-3.579373537	-3.654864458	-5.244969832	-0.594006577	-0.454347436	-4.377973531	-3.177856123	-5.291696819	-1.515516667
-6.670273885	-1.615916637	-4.817745933	-0.198760885	-1.521678954	-3.456054653	-3.385897325	-5.718608874	-0.477324128	-0.684694238	-4.535874274	-3.287561326	-5.519510615	-1.864344442
-7.717693536	-1.918895618	-5.375743634	0.206791171	-1.490641604	-3.711670003	-3.56243658	-5.31946943	-0.398306373	-0.532534475	-4.809665422	-2.803775609	-5.391795439	-1.827144547
-7.52658326	-2.034198122	-5.486716157	0.036542124	-1.465582157	-3.812255365	-3.779928834	-5.840959603	-0.599574624	-0.45849725	-4.914119239	-3.141693108	-5.694074283	-1.790305029
-7.439576672	-2.241804436	-5.545428887	0.086028783	-0.813885373	-3.922077672	-3.946861899	-6.050804699	-0.399904886	-0.595452765	-5.305214615	-3.166167829	-5.828174361	-2.015780681

hsa-miR-4646-3	hsa-miR-4648	hsa-miR-4649-5	hsa-miR-4651	hsa-miR-4655-5	hsa-miR-4656	hsa-miR-4664-3	hsa-miR-4665-3	hsa-miR-4665-5	hsa-miR-4667-3	hsa-miR-4667-5	hsa-miR-4673	hsa-miR-4674	hsa-miR-4675
-3.984714351	-1.600445733	0.004221176	1.628006305	-1.76923481	-1.981265312	-4.855615318	-4.322255494	-1.900658295	-4.150213533	-3.147754149	-3.206587575	0.346620082	-1.288659119
-4.413376219	-1.899935494	-0.329123347	1.816933052	-1.470840442	-1.91375084	-5.029476403	-4.017738175	-1.334749413	-4.568443333	-3.365597329	-2.895430703	0.577340493	-1.810798438
-4.387722834	-1.618565117	-0.426754788	1.523418706	-1.391211379	-1.720640442	-5.000705933	-4.092507619	-1.603492268	-4.502343595	-3.040709232	-3.228859925	0.097413304	-1.790285374
-4.21469507	-3.022557345	-0.572948725	1.618726104	-1.469289448	-1.666564125	-5.141112309	-4.218892077	-1.354567362	-4.3130654	-3.063745003	-3.251640613	-0.005339159	-1.568777433
-4.030702394	-2.340844008	-0.054796411	1.690595843	-1.563538099	-1.673697638	-4.818672845	-3.95112568	-1.663766014	-3.848034691	-2.984062637	-3.237996578	0.34237672	-1.331850202
-4.234245288	-2.740334355	-0.079793882	1.598947233	-1.526941316	-2.031874159	-4.811745201	-3.932307026	-1.212820523	-4.233820396	-3.079292053	-2.806734396	0.20000137	-1.613937002
-4.094865467	-2.317760217	-0.386120618	1.793242636	-1.401586201	-1.719716883	-4.831894277	-4.026208718	-1.266769293	-4.158406552	-3.052875838	-3.115577513	0.270015159	-1.697177897
-4.451648154	-3.54642379	-0.64345922	1.645646615	-1.459229247	-1.59972425	-5.219380593	-3.980643685	-1.127662502	-4.459651746	-3.018103824	-2.907921545	0.111424675	-1.851923339
-4.376935246	-2.512628876	-0.553231579	1.542255968	-1.494450742	-1.798133297	-5.099261144	-4.239332598	-1.345373075	-4.387483841	-3.198124904	-3.043603515	-0.305169258	-1.849205071
-4.306144709	-4.290515263	-0.252327454	1.575131369	-1.530332585	-2.001318947	-4.958606334	-3.489838973	-0.962697354	-4.445339692	-2.930883448	-2.662954118	0.267267827	-1.658176807
-4.756562697	-4.000093788	-0.884837454	1.545063936	-1.492556329	-0.911610017	-5.275238421	-4.005533594	-1.191656016	-4.644309839	-2.90249741	-3.01635793	-0.002725199	-1.752682144
-4.591625704	-1.821782963	-0.346800874	1.368543745	-1.605128833	-2.094971011	-5.642855808	-4.405042604	-1.497382854	-4.859142778	-3.306334659	-2.914448626	0.177401897	-1.901423282
-4.832887426	-2.541791294	-0.721948429	1.30352684	-1.677127044	-1.799333448	-5.540634167	-4.400877589	-1.510222897	-4.79422515	-3.133958079	-3.233113722	-0.184079645	-1.957616253
-4.751352958	-3.95330018	-0.398787417	1.379921757	-1.557775189	-1.881461973	-5.293796714	-3.956626303	-1.23427289	-4.641934321	-3.013024846	-2.887991227	0.128091625	-1.676848017
-4.869481871	-3.436733119	-0.04148708	1.531940019	-1.596747304	-2.204956122	-5.314385383	-3.870934694	-1.19357528	-4.785887716	-3.256292155	-2.782424391	0.670983105	-1.86731629
-4.724716047	-2.548915092	-0.776808911	1.429130055	-1.342482305	-1.666576575	-5.334738131	-4.154289534	-1.488105221	-4.760850547	-3.104391251	-3.003544102	0.132570011	-2.117226942
-4.893914438	-2.784583449	-0.219552228	1.521437413	-1.515264542	-2.092954046	-5.046145636	-3.959270678	-1.114630878	-4.758784289	-3.228061742	-2.592683274	0.388796465	-2.116815062
-4.829410341	-1.623072285	-0.825650287	1.391095285	-1.497549667	-1.865380356	-5.387691138	-3.93380424	-1.711923841	-5.238895333	-3.289882535	-2.942122075	-0.312269096	-2.053640068
-4.641680796	-2.637160986	-0.273091529	1.311887944	-1.517425767	-1.7193871	-5.253661852	-4.062281563	-1.446265963	-4.66249132	-2.87458266	-3.073846983	0.121371593	-1.651065812
-4.698789402	-2.655484183	-0.552860748	1.399931847	-1.378102083	-1.925137276	-5.160407061	-4.01615682	-1.137320426	-4.733241967	-2.946773104	-2.80714789	0.11430675	-2.012451635
-4.973021563	-2.543764877	0.029890313	1.605332629	-1.510494044	-2.220925162	-5.153056942	-3.947681954	-1.209811748	-4.78793639	-3.191943634	-2.673296238	0.635277856	-1.917396676
-4.872354918	-3.416688906	0.020263357	1.692838672	-1.523043905	-2.227475969	-5.358890724	-3.744248075	-1.126429702	-4.797258445	-3.194410032	-2.653307866	0.553172932	-2.008572316
-4.608370038	-2.941381172	-0.632844832	1.489403951	-1.515638398	-1.663234891	-5.162378478	-4.034115554	-1.403881701	-4.511930965	-2.990745427	-3.829306904	-0.184878558	-1.555302534
-4.1464263	-2.32284041	0.197316704	1.518562969	-1.213389783	-1.720883844	-4.935611179	-4.134910889	-1.14791026	-4.26679913	-2.845721102	-2.980517734	0.216048184	-1.04517025
-4.797162718	-3.677988739	-0.212469004	1.547709176	-1.262719382	-2.044819559	-5.226705901	-3.890464161	-1.306894672	-4.81344926	-3.121849447	-2.690988512	0.510643733	-1.989055538
-4.046618253	-2.876425323	0.233974453	1.427366108	-1.213534335	-2.171470203	-4.686866766	-4.392161103	-1.210062489	-4.240770914	-2.805324147	-2.900179054	0.446775348	-1.187285714
-4.833701756	-3.481653838	-0.598948234	1.269187963	-1.669305234	-1.927418637	-5.71432492	-4.289242813	-1.023928268	-4.751500736	-3.18054246	-3.09832976	-0.001383453	-2.064095953
-4.611706205	-2.977722027	0.108953181	1.317682943	-1.469717709	-2.213556312	-5.292551777	-4.308546587	-1.364983047	-4.531384813	-3.061258135	-3.025924471	-0.003807128	-1.435638017
-4.195443232	-3.107747819	0.35175496	1.6714308	-1.769426522	-2.500289945	-5.117344661	-3.989725706	-1.207579186	-4.630991589	-3.70035893	-2.988150085	0.566772695	-1.620001423
-4.441942557	-3.42685335	-0.569285892	1.403373721	-1.313440371	-1.672539366	-5.27446331	-4.156631832	-1.131824164	-4.450027841	-3.027764845	-2.974461817	-0.110190623	-1.870731851
-4.503094178	-3.970356098	-0.866925271	1.403120361	-1.191052566	-1.45980547	-5.318867789	-4.25373732	-1.167533763	-4.630051554	-2.812910085	-3.24076404	-0.408939042	-1.899970179
-5.126570877	-4.326430553	0.605885349	0.630040692	-1.908206063	-2.216848921	-6.105795219	-4.550610037	-2.160095394	-5.238372233	-3.474254228	-3.982116509	0.755388762	-1.311165193
-4.69670981	-3.339390298	-0.435267335	1.345039205	-1.278868528	-2.245281102	-5.653599681	-3.744630783	-0.79473719	-4.993508936	-3.273626794	-3.277270721	0.32123825	-2.476235831
-4.742575954	-5.068791335	-0.701711856	1.659107868	-1.235552718	-1.422844745	-5.418816515	-3.52586952	-0.202831073	-4.829984708	-3.648847064	-3.394612913	0.054701183	-2.525959285
-5.057789736	-4.127565867	-0.720040503	1.157163976	-1.605318766	-2.21810254	-5.517552552	-3.895260967	-1.374216128	-4.847300614	-3.589573075	-3.362744282	-0.024395562	-2.565988686
-4.6884682	-2.795862275	0.310324707	1.995913546	-1.575963911	-2.169780951	-4.974232214	-3.718745927	-0.869354564	-4.376977874	-3.726294828	-3.294233537	0.985253865	-2.245291739
-6.896380239	-3.916706389	0.243973557	1.507286864	-1.416837987	-2.414783756	-5.259363356	-2.894530878	-0.744511238	-5.541537051	-4.081925413	-3.160875481	1.02397119	-2.227962967
-5.212331163	-4.325054163	-0.356971164	1.541630815	-1.414922767	-2.133005927	-5.134259327	-3.475213543	-1.050792285	-4.659227526	-3.709844655	-3.292589582	0.002586336	-2.436954161
-4.67466363	-4.09622736	-0.792789376	1.488697061	-1.587422825	-2.047144767	-5.076020404	-3.914897872	-1.353826586	-4.455842378	-3.702643945	-3.478136759	0.015754336	-2.467929849
-5.280530705	-3.744658079	0.566046574	1.792554898	-1.851917934	-2.190108137	-5.231801263	-3.455462055	-1.05990153	-4.732651524	-4.092028959	-3.455495271	0.823523734	-2.170658921
-4.747508159	-3.525625393	0.531961625	1.754903899	-1.568773801	-2.321096832	-4.973533583	-3.818019873	-1.657774002	-4.437405511	-3.504937366	-3.27942466	1.022339135	-2.000526379
-5.082623062	-4.999973976	-1.348281301	1.516756977	-1.097128275	-1.87179903	-5.319492975	-3.466502605	-0.399422839	-5.016677709	-3.808888865	-3.15330951	-0.225918574	-3.240687933
-5.530311653	-4.609970139	-1.65311562	1.21531667	-1.190513499	-1.83352373	-5.388114728	-3.580660994	-0.571637978	-5.30449593	-3.784600561	-3.510453282	-0.4605979	-3.422461786
-4.020508247	-1.787697992	0.322177436	1.464143509	-2.029515498	-1.39996465	-4.497616188	-3.543422414	-1.772853521	-3.697906981	-3.036311928	-3.389946678	0.751343402	-0.956052374
-5.097356693	-4.799718973	-0.683690947	1.397791848	-1.305855576	-1.906912142	-5.393311251	-3.873196416	-0.680994672	-4.981586107	-3.626517814	-3.061228173	0.239271129	-3.066637117
-5.131945362	-4.203688909	-0.136223015	1.393196915	-1.327842596	-2.37120744	-5.48737829	-3.726701451	-0.952260522	-4.833811102	-3.656858543	-3.264799821	0.313686637	-2.598596618
-4.525766997	-1.662431328	-0.425371017	1.43618519	-1.700868988	-1.834927363	-4.983665877	-4.029766278	-1.659886349	-4.470476168	-3.52453261	-3.701592253	0.230905083	-2.094441302

-4.828320919	-3.797609502	-1.036923161	1.325222055	-1.36963284	-1.861902125	-5.408426501	-3.999721098	-0.940436896	-4.881245818	-3.383087577	-3.384785911	0.034602837	-2.475656328
-5.136690208	-3.910405798	0.130336315	1.592489206	-1.391706346	-1.804242995	-5.1248186	-3.199821502	-0.479202176	-4.791775362	-3.517430461	-3.386472357	1.461746155	-2.606932896
-4.89943139	-3.202362768	0.038525664	1.752815532	-1.311861774	-2.428192426	-5.017889753	-3.349913258	-0.825951581	-4.748601148	-3.630227433	-2.8796929	0.820700762	-2.283190112
-4.152193506	-0.111983644	0.081421596	1.569451129	-1.726877539	-1.214904958	-4.638717459	-3.903830763	-1.703179014	-4.106916016	-3.259301256	-3.579885968	-0.084766309	-1.224016122
-4.553607041	-3.07785081	0.243433337	1.25500576	-1.610927024	-1.875543902	-4.904160856	-4.005223379	-1.573425057	-4.427542154	-3.5782432	-3.601877406	0.868735136	-1.817898876
-5.262729625	-3.863678633	-0.381435288	1.426804715	-1.126471941	-1.788896769	-5.460374214	-3.243902585	-0.60770448	-5.366384192	-3.255803214	-3.096326306	0.616657523	-2.734908991
-5.277613509	-3.770564825	0.946921312	1.550999837	-1.264236503	-2.20243937	-4.557820921	-2.863192171	-0.817085013	-6.040283407	-3.334028358	-2.612095497	1.42201871	-1.868018887
-4.635693477	-3.055408237	1.212095618	1.250047414	-1.813421093	-2.111493824	-4.931215748	-3.755376516	-1.773306728	-4.244889979	-3.40143721	-3.949196612	1.493197577	-1.20779346
-5.178189036	-4.898385485	-0.067579918	1.453253737	-1.174720415	-1.734137666	-5.010791203	-3.272487646	-0.155549965	-5.006515505	-3.664725675	-3.056702835	0.249474179	-2.444569005
-5.145870938	-3.863552211	-0.995382025	1.310973039	-1.233406603	-1.573189866	-5.239804805	-3.740048225	-0.376958668	-5.13266357	-3.461195432	-3.475058127	-0.095423137	-2.707301994
-5.493976282	-5.207211527	-0.138879535	1.108464414	-1.285653077	-2.097369842	-5.484054749	-3.016020086	-0.63506094	-5.192619518	-3.465281319	-3.372019314	0.312013637	-2.253632213
-4.897213978	-4.36370594	-0.94777061	1.213182965	-1.414900574	-2.033995031	-5.018292831	-3.66691934	-0.783340887	-4.967705646	-3.638405083	-3.63450209	0.383633283	-2.89648496
-5.125174142	-2.882047894	0.255582709	1.576702463	-2.258997704	-2.247559872	-5.36326689	-4.115102781	-1.800823897	-5.046607328	-4.17696752	-3.584858664	0.729710226	-2.052251287
-5.400059368	-4.274026928	-0.581682613	1.153032909	-1.629457794	-1.948639981	-5.763175143	-3.661734584	-0.463819546	-5.262222592	-4.149878976	-3.847896139	-0.226999682	-2.649296559
-6.464192239	-4.574807205	0.227368642	1.084341478	-1.456089465	-2.344153819	-6.952698327	-3.083293809	-0.705745991	-6.912487308	-3.717191808	-3.376491426	0.404497652	-2.072479325
-5.135104947	-3.825006149	0.872645051	1.457354805	-1.384147324	-2.371433354	-5.630259671	-3.290351627	-1.082920154	-5.322973416	-3.422318984	-3.128989282	0.870279181	-1.859678843
-5.234176515	-4.820282989	-0.619190443	1.541111106	-1.706574466	-2.439859036	-5.236864396	-3.605630338	-0.913592498	-4.784828947	-3.845443145	-3.358673685	0.287135682	-2.700993349
-4.048026375	-2.015302721	0.327337724	1.860401171	-2.069892199	-1.974381905	-4.952493286	-4.007026287	-1.782770174	-4.259215271	-3.971873454	-3.574434064	0.512843253	-1.719970517
-4.682855504	-3.178696921	-0.416334541	1.090008136	-1.766267459	-1.856612698	-5.006999067	-3.951934183	-1.030581916	-4.569915433	-3.082071572	-3.613708434	0.237858868	-2.069633697
-5.559388452	-4.829982035	-1.463143303	1.315645483	-1.036078408	-2.169275757	-5.828075328	-3.6159193	-0.876987759	-5.518647336	-3.019388616	-3.013649422	-0.40482835	-3.161361718
-4.526518456	-1.935986813	-0.376367517	1.780357357	-1.518459622	-1.755170486	-5.09104608	-4.139136252	-1.08519327	-4.544056086	-3.221182182	-2.971104755	0.535135437	-2.323075397
-4.961409747	-3.34234732	-0.893903059	1.138141334	-1.437647242	-2.125955529	-5.685400743	-4.250575061	-0.478667208	-5.16916547	-2.929038618	-3.647067748	0.006170734	-2.646911495
-5.121980278	-4.424956086	-0.998140617	0.968406958	-1.270624023	-2.121252874	-6.014171363	-4.826497807	-1.602701899	-5.289493525	-3.100574106	-3.63418395	-0.773549639	-2.326788557
-5.581734721	-4.307033622	-0.1713817	1.247179378	-1.139195133	-2.49175182	-7.11551407	-3.998278937	-0.536435082	-6.226606451	-3.185522682	-3.043183493	0.519335322	-2.461770819
-5.153256689	-3.835795307	-0.631806947	1.208847673	-1.217615909	-2.309203605	-6.037438723	-4.205659217	-1.334210793	-5.043993768	-3.066428276	-3.319354535	-0.042589057	-2.027045857
-5.786394293	-4.381144429	-0.718626373	1.487361348	-1.502780633	-2.293023595	-5.073828255	-3.536663483	-0.272095658	-5.30357506	-3.466609467	-3.066111683	0.133756374	-2.803873754
-4.921468395	-3.861313065	0.548807232	1.748723695	-1.480937676	-2.218324735	-5.155190812	-4.066674764	-0.221932116	-5.247081032	-2.880226749	-3.465558044	0.906944555	-2.264283194
-4.809211152	-2.51451718	0.805337698	2.048023934	-1.663141738	-2.290957878	-5.411849182	-4.222668091	-0.133559561	-4.790168006	-3.319008966	-3.105151176	1.017115808	-2.306964593
-4.912369592	-3.613726051	-0.432529242	1.394699588	-1.381232537	-2.277480573	-5.531855765	-4.104725608	-0.341011639	-4.957967916	-2.931740557	-3.384123733	0.376151622	-2.50521719
-5.151417307	-3.867594803	-0.859767833	1.311556281	-1.329737322	-2.148237632	-5.599572822	-3.912349359	-0.383675724	-4.820214013	-3.149963438	-3.279560024	-0.085835378	-2.751757735
-5.2135581	-2.692053399	-0.687135399	1.334903969	-1.364294081	-2.137437037	-5.803401166	-4.136872879	-0.356993715	-5.313426901	-2.835649453	-3.663380742	0.316623588	-2.916328258
-5.05793239	-2.513968983	0.573082894	1.644376809	-1.304512335	-2.191544916	-5.268099491	-3.670721559	-0.681875051	-5.191092975	-3.007797949	-2.906170881	0.988797527	-2.013550052
-4.819122121	-3.539920303	-0.584162193	1.174995595	-1.209903564	-2.173700194	-5.583895924	-4.166128052	-0.67223738	-5.081898321	-2.678406145	-3.55363328	0.120894974	-2.579141866
-5.027207304	-2.959427831	-0.315704293	1.310032636	-1.260608967	-2.342229794	-5.403130395	-4.145823859	-0.419116185	-4.986377064	-2.823946182	-3.623507093	0.366002966	-2.360579974
-5.067236393	-3.7046981	-0.653051055	1.241465517	-1.46212717	-1.928268113	-5.492935057	-3.861293718	-1.29263365	-4.953411599	-3.689371911	-3.519583187	-0.057574	-2.481652139
-4.761273977	-4.162138153	0.059979811	1.735927888	-1.018184462	-1.839931705	-5.060135523	-3.507785257	-0.500822732	-4.691662665	-3.663136341	-2.969163525	0.447585603	-2.321710325
-5.09703161	-4.384502868	-0.757239543	1.34906538	-0.887971862	-1.861781247	-4.970277813	-3.240365079	-0.383218788	-4.981097257	-3.26150578	-2.992504624	0.208385657	-2.755386809
-5.273094027	-3.642524879	-0.68915459	1.331841035	-1.254403133	-2.094747231	-5.235633732	-3.477231189	-0.945558007	-4.880194461	-3.67040188	-3.388185874	0.250065138	-2.712839028
-5.274808622	-4.79137162	-0.334758282	1.210181991	-1.457907004	-2.30356104	-4.975146522	-3.780782319	-0.93191533	-4.800968231	-4.001046114	-3.380712542	0.281201105	-2.692169518
-5.136822706	-4.786244392	2.649687142	1.878245211	-1.439166185	-2.246313616	-4.807393602	-3.187147619	-1.107399048	-4.596840237	-3.029574445	-3.820149616	1.12621358	-0.585712726
-5.060083139	-4.206682644	-0.890441696	1.279935817	-1.402409467	-1.898393002	-5.172819705	-3.532443564	-0.527229942	-4.844692108	-3.734283102	-3.34728517	0.067088754	-2.989748244
-5.121680895	-4.839835934	-1.171013136	1.06165388	-0.988595397	-1.891307454	-5.730850382	-4.073052697	-0.545062954	-5.209196616	-3.516983856	-3.548279328	0.02333414	-2.841851923
-5.490656652	-3.725389789	-1.562069664	1.009625347	-1.061091585	-1.597209725	-5.750300929	-3.662067363	-0.868023431	-5.315283176	-3.786716479	-3.652756331	-0.470961337	-2.581677838
-5.376532984	-3.748659997	1.234638074	1.068492534	-1.808562598	-2.435195994	-5.596280744	-4.032860882	-1.56618983	-5.154853032	-3.691242038	-3.809469691	0.43854767	-1.375857944
-5.384422305	-4.318235242	0.510984707	1.271414812	-1.795729071	-2.362016369	-5.71363897	-4.139616898	-1.373023508	-5.598834524	-3.964728864	-3.390534836	0.722358519	-1.904764691
-5.505426894	-4.528856387	-0.204799145	1.194243816	-1.321684726	-2.229096269	-5.599159684	-3.535779718	-0.914522523	-5.311997307	-3.503690888	-3.226625302	0.493171624	-2.642251841
-5.791588853	-4.565352132	0.847543233	1.743265579	-1.374159094	-2.159622011	-4.975076808	-3.184944518	-0.692082654	-5.406897467	-3.929242292	-3.424580353	1.005961595	-2.296282007
-4.89646425	-4.426323812	-0.996023935	1.174683374	-1.307486717	-2.014234242	-5.520965154	-3.776442751	-1.042873989	-5.037335197	-3.856731958	-3.962166431	-0.149545174	-2.713321278
-4.981195238	-5.130856807	-0.557875969	1.462482773	-1.136759523	-2.038962271	-5.461052458	-3.576728052	-0.494482309	-5.211866924	-3.354395177	-3.362035873	0.515124376	-2.878301874

-4.615304887	-3.170259468	-0.082975332	1.545560211	-1.206032848	-2.177371942	-5.482969391	-3.944011481	-0.10876961	-5.048671284	-2.800368906	-3.330254689	0.774854783	-2.545493265
-5.116075023	-3.092584524	-0.525755059	1.285359261	-1.276098037	-2.116593939	-5.17560192	-4.029728894	-0.118901868	-4.977934865	-2.69407906	-3.606557549	0.437479213	-2.552961119
-4.912972605	-3.358160432	-0.766603136	1.291481863	-1.179002878	-2.133373733	-5.722943969	-4.070471885	-0.013914691	-4.901810614	-2.721797657	-3.670478647	0.060600417	-2.778172844
-4.864968885	-3.052667215	-0.261479112	1.543537963	-1.350624555	-2.277076289	-5.581323129	-4.12729877	-0.080639666	-5.084613374	-2.868675213	-3.644529628	0.686255414	-2.770339263
-5.132704109	-4.188503901	-0.854908591	1.173930779	-1.41250045	-2.315774877	-5.774361689	-4.306159822	-0.378368656	-4.972257971	-3.04794778	-3.613110943	0.027756947	-2.631446191

hsa-miR-4685-5	hsa-miR-4687-3	hsa-miR-4687-5	hsa-miR-4688	hsa-miR-4689	hsa-miR-4690-5	hsa-miR-4695-5	hsa-miR-4697-3	hsa-miR-4697-5	hsa-miR-4700-3	hsa-miR-4701-5	hsa-miR-4706	hsa-miR-4707-3	hsa-miR-4707-5
-4.18623347	1.313199514	-3.879653893	-2.466268292	0.602888811	-3.641383665	-1.128958702	-4.798484517	-1.246074338	-4.360535756	-4.68164887	-2.48955926	-2.289304221	-1.857755377
-4.422322087	0.62443024	-3.860039942	-2.058727706	-0.081889587	-3.152071005	-0.774181679	-4.97227341	-1.536181496	-4.532619585	-4.947367517	-2.454677452	-1.98500689	-1.104921772
-4.440159736	1.026830194	-3.811720208	-2.245344626	0.286240926	-3.109922996	-0.424481803	-4.932816931	-1.567424478	-4.520579786	-4.738720437	-2.414751064	-2.167605655	-1.325960615
-4.611454084	1.081855332	-3.834447116	-2.012763783	0.363747619	-2.98704417	-0.599625036	-4.968326386	-1.57246883	-4.411489857	-4.962023091	-2.374865443	-2.394527327	-1.207005875
-4.475766283	1.288626119	-3.562469838	-2.530180763	0.642937546	-3.363823699	-0.739051848	-4.750172727	-1.28602457	-4.422133822	-4.65874171	-2.509245033	-2.103628546	-1.671889695
-4.188455111	0.880680122	-3.564214451	-2.216467119	0.158220145	-2.854409052	-0.693447013	-4.906351355	-1.264123221	-4.548052747	-4.710318115	-2.31465419	-2.094002041	-1.314713817
-4.374628876	1.170376227	-3.613265424	-1.952535656	0.521188566	-2.89731753	-0.874155186	-4.771194346	-1.411487331	-4.328255875	-4.768774668	-2.384943351	-1.858953318	-1.166992508
-4.520051664	0.698136673	-3.745084588	-2.30718207	-0.180581192	-2.677885303	-0.311058032	-5.087764257	-1.500293023	-4.55847121	-4.858981015	-2.362483519	-2.162276394	-1.045880759
-4.524025581	0.830791579	-3.893066624	-2.488360773	0.18504536	-2.964314293	-0.480512804	-5.070628689	-1.580614756	-4.792876035	-4.95166032	-2.420158815	-2.190947311	-1.188995526
-4.330566827	0.752542189	-3.846169518	-2.13501374	0.136063395	-2.961467463	-0.820731059	-4.944497382	-1.400957266	-4.601466917	-4.829021755	-2.281371634	-1.973473014	-0.837613123
-4.752153931	0.532970121	-3.883771209	-2.321030917	0.049424187	-2.964982901	-0.465539752	-5.108524474	-1.659433753	-4.984209718	-5.034707802	-2.442268227	-2.291261878	-1.09958779
-4.398993467	0.653619119	-4.257909583	-2.549950114	-0.033803828	-3.034895777	-0.786499507	-5.705305768	-1.651688254	-4.953628531	-5.611935145	-2.476120273	-2.021543552	-1.325715267
-4.673666984	0.495764575	-4.041113073	-2.502529697	-0.129674256	-2.877639694	-0.487490038	-5.703240939	-1.694058776	-5.040679007	-5.446083279	-2.492565773	-2.373974381	-1.298588031
-4.534650179	0.834344916	-3.961990053	-2.48569086	0.096536422	-2.993676465	-0.675604378	-5.242795604	-1.590295504	-4.844544296	-4.92095386	-2.601362342	-2.179080433	-1.148713265
-4.603807113	0.517383765	-3.925668754	-2.222822392	-0.125288819	-3.172898704	-0.875365491	-5.24315987	-1.541519856	-5.09184489	-5.215532746	-2.506158329	-1.903647034	-1.146109965
-4.719555476	0.431426467	-4.02531397	-2.431139031	-0.114639243	-2.999337569	-0.092401359	-5.296500499	-1.686424848	-5.112553379	-5.128999444	-2.506120655	-2.134213008	-1.122952961
-4.372936439	0.52199765	-3.918582714	-2.263342834	-0.107997609	-3.236814645	-0.846194046	-5.289468419	-1.506604328	-5.129928066	-5.076383623	-2.43429536	-1.803088596	-1.253475859
-4.705791212	0.244497467	-4.319643798	-2.365276389	-0.298268707	-3.555511808	-0.717162751	-5.372076588	-1.741304914	-5.178388185	-5.233882097	-2.689911873	-2.090068538	-1.176844753
-4.481703639	0.65830752	-3.967364953	-2.531074444	0.15494731	-3.305518981	-0.806289579	-5.23342042	-1.49583816	-5.018176229	-5.039730255	-2.540513683	-2.270122796	-1.445032894
-4.532768914	0.647810671	-3.814289593	-2.296097781	-0.027853887	-3.060307097	-0.468288748	-5.296139391	-1.577887138	-4.943553447	-5.186214402	-2.404330865	-2.080701689	-1.078430791
-4.409557799	0.638496382	-3.954994975	-2.222979939	-0.094261903	-3.212790493	-0.808217605	-5.284447609	-1.459922563	-4.975907526	-5.190343806	-2.41882788	-1.728582556	-1.220689697
-4.558045887	0.534416719	-3.84918522	-2.229585864	-0.087016023	-3.245802828	-0.550753023	-5.368735493	-1.614587627	-5.011427885	-5.210042565	-2.529618883	-1.564653652	-1.206627281
-4.622156572	0.779796044	-3.978324576	-2.464577361	0.169720119	-3.065962462	-0.44372389	-5.151312368	-1.565545894	-4.855907134	-5.058218784	-2.497001968	-2.188127964	-1.239834324
-3.962099418	1.450509385	-3.865860767	-2.436731388	0.886728428	-2.951545854	-0.661282718	-4.883705719	-1.128985791	-4.468569781	-4.849068677	-2.243808269	-2.233193321	-1.322390636
-4.796477842	0.508258359	-4.118178338	-2.536028649	-0.190508573	-3.029985821	-0.484104275	-5.188474568	-1.747353947	-4.975499348	-5.052502744	-2.485713115	-1.861299237	-1.374684034
-3.864192552	1.71787679	-3.773843241	-2.698837716	0.801822552	-3.120032936	-0.752014518	-4.703615032	-1.030451752	-4.351744951	-4.749380134	-2.223273728	-2.239098049	-1.546207597
-4.811870579	0.359186819	-3.859247131	-2.723438058	-0.381747562	-2.712313024	-0.59638919	-5.6220955	-1.779015303	-5.075684831	-5.436172811	-2.663101081	-2.349692365	-1.551413072
-4.249310341	0.956216874	-3.841704711	-2.726680779	0.373398037	-3.063055172	-1.199121141	-5.410383957	-1.436189175	-4.700668475	-5.250441674	-2.644412028	-2.369820624	-1.436865405
-4.063658712	0.908313003	-4.151294266	-2.231316107	0.020922643	-4.009504399	-1.397717808	-4.936817276	-1.253246549	-4.662671991	-5.327120667	-2.65774531	-2.069932314	-1.514487463
-4.717964944	0.648421506	-3.872202687	-2.3456658	-0.007501464	-2.947484024	-0.499610067	-5.186566979	-1.719784427	-4.701705057	-5.100569886	-2.388922375	-2.155246617	-1.196247455
-4.760629356	0.615851328	-4.039783148	-1.917502397	0.023132299	-2.55555817	-0.344220864	-5.28393128	-1.599006872	-4.731303572	-5.189682813	-2.345600713	-2.540733168	-0.840690141
-4.424233011	0.494950282	-4.506676439	-2.5145665	0.071141148	-3.434399109	-2.05849285	-6.059237348	-1.650388636	-5.504699315	-5.934439315	-3.06039178	-3.602000249	-1.760906106
-5.040690262	0.273297243	-4.199815908	-1.596779522	-0.83195071	-2.83994709	-0.769225184	-5.562392499	-1.706277997	-4.734486156	-5.373926362	-2.294593665	-2.382563087	-0.791291575
-5.00409236	0.639733531	-4.15073453	-1.897629635	-0.784348104	-2.150317436	0.005898522	-5.477727556	-1.275288966	-4.832360079	-5.51516417	-2.193469257	-2.533644853	-0.3685554
-4.915022585	0.220767417	-4.513062305	-2.327269641	-0.446657487	-3.585276833	-0.666942926	-5.500656593	-1.874091064	-5.181522645	-5.314243234	-2.720048818	-1.977301935	-0.937180968
-4.586647716	0.759809975	-3.800645862	-2.203455769	-0.338056585	-3.105684217	-0.367827555	-5.032783455	-1.391695556	-4.765632893	-5.164656003	-2.490231842	-1.632109228	-0.786598986
-5.341948788	-0.00454646	-4.034434241	-1.562582432	-0.951893304	-3.708291598	-0.573529333	-5.973303772	-1.500178215	-5.902899576	-5.142303627	-2.089389958	-1.732698218	-1.127795285
-5.172880014	0.380331482	-3.995439563	-2.051066592	-0.85319872	-3.371786282	-0.512227404	-5.209592096	-1.624085311	-5.032527495	-5.091810279	-2.48442878	-1.894672097	-0.630261806
-5.08485336	0.612433625	-3.842847517	-2.57196479	0.148940972	-3.376680784	-0.230375717	-5.012938012	-1.69283207	-4.83979771	-5.098018158	-2.553475029	-2.281026097	-0.856184901
-4.709862391	0.308774289	-4.192038439	-2.043623017	-0.460032835	-3.395818442	-1.252677245	-5.16081034	-1.473283696	-5.070968996	-5.272679331	-2.230500409	-1.679675495	-1.067184397
-4.698665025	0.646494652	-3.893152797	-2.113647865	-0.31350092	-3.703509368	-1.038048273	-5.080331694	-1.532157529	-4.80336099	-4.987756949	-2.653349972	-1.755359361	-1.118141132
-5.466858705	0.40166919	-4.081281149	-2.092104226	-1.020963073	-2.755193076	-0.32130728	-5.758696313	-1.855347557	-4.992992426	-5.579431292	-2.213055011	-2.725771294	-0.639638286
-3.876645915	-0.239371956	-4.147719866	-2.445297317	-1.364368934	-3.050080187	-0.432099293	-5.473911734	-1.825978744	-5.51301005	-5.500373225	-2.223985264	-3.185778556	-1.078409928
-3.781116438	2.013280756	-3.605723364	-2.455314796	1.149566374	-3.909179099	-0.723354269	-4.315200478	-0.62479082	-4.058654492	-4.354960368	-2.361891151	-2.148505211	-1.698709145
-5.305277516	0.511874798	-4.026685962	-2.018809741	-0.929714856	-3.023537519	-0.028129073	-5.647898217	-1.740142613	-4.985555278	-5.46172369	-2.233090726	-2.54496534	-0.82425352
-5.137418907	0.516158793	-4.241870473	-2.231423857	-0.826515402	-3.384927617	-0.438072687	-5.434996978	-1.75993993	-5.191973614	-5.320007062	-2.612397972	-2.233841158	-0.749141571
-4.985849999	0.908073612	-3.743923674	-2.80936451	-0.029426849	-2.976999012	-0.001422323	-5.01310984	-1.789002779	-4.630129015	-4.852767438	-2.620829248	-2.094997101	-0.593127447

-5.071170956	0.408109717	-4.118367498	-2.269267589	-0.693995551	-3.114364269	-0.204203406	-5.317784889	-1.772696317	-5.004714955	-5.324444144	-2.534923436	-2.749739534	-0.649105375
-5.221712802	0.257097126	-3.796235489	-1.676357937	-0.958477334	-2.827838781	-0.161257582	-5.364264893	-1.581477394	-5.159218817	-5.296581741	-1.847836502	-2.529904878	-0.475949195
-4.639135862	0.442712108	-3.853920745	-1.797328503	-0.4015964	-3.217880556	-0.508012047	-5.251089287	-1.487852773	-5.004542579	-5.274382931	-2.154132168	-1.656329616	-0.435712647
-4.094884614	1.690061617	-3.831619951	-2.86028468	1.047494142	-3.573704837	-0.502504381	-4.587291767	-1.103045653	-4.259313097	-4.661772367	-2.177086698	-2.486907699	-1.108658456
-4.480819684	1.094871074	-3.824397143	-2.439224745	0.347738425	-3.669978987	-0.731052396	-4.977168316	-1.381707169	-4.805502402	-5.020353978	-2.368838582	-2.563965233	-1.448617567
-4.667236856	-0.127344696	-4.072950972	-1.711212342	-1.041983164	-2.778571343	-0.594454488	-5.939489492	-1.854352525	-4.975672533	-5.439898853	-1.679881331	-2.576664855	-0.613103331
-4.234052962	-0.067024747	-3.303143069	-1.420911247	-0.50463571	-2.874059358	-0.460695815	-4.775281991	-1.448094267	-5.110050548	-5.191577705	-1.385145569	-2.230433865	-0.723285408
-4.318005424	0.980252342	-3.749409668	-2.369596728	0.408294267	-3.481079073	-0.60419894	-5.063372942	-1.040977851	-4.855298146	-5.180083876	-2.503856644	-2.656760706	-1.313404209
-4.773551762	0.627068801	-3.737177045	-1.952863874	-0.76166071	-2.425539978	0.047417835	-5.129838885	-1.367686494	-4.970017992	-4.058887219	-1.987770617	-2.461934732	-0.406443726
-4.87956891	0.670042311	-4.046743217	-2.241130114	-0.705732413	-2.658564646	0.031397065	-5.512232609	-1.632122027	-4.994108956	-5.421972027	-2.251630848	-2.804090699	-0.603894176
-4.938896817	-0.153995153	-3.652487772	-2.060676492	-1.13838825	-3.058036086	-0.872308377	-6.138515267	-1.721562742	-5.40437546	-5.336472978	-1.952293623	-2.773802946	-1.265420205
-4.886868363	0.392860668	-3.617133532	-2.310628891	-1.16430435	-2.829677223	0.004097355	-5.398531943	-1.702816644	-4.950592356	-5.227631086	-2.189635051	-2.617146636	-0.981774268
-4.690804681	0.588137012	-4.254022549	-2.10759985	-0.199638221	-4.041047878	-1.156984055	-5.281652169	-1.61962052	-5.154412646	-5.564524937	-2.819860155	-2.192105729	-1.372045003
-5.256801168	0.205783328	-4.064177446	-2.257218178	-1.075361055	-2.913724896	-0.371630832	-5.684199625	-1.798333358	-5.546671994	-5.200869241	-2.116632928	-2.878556985	-0.999037781
-5.003048288	-0.070095421	-4.404476214	-1.622612191	-0.84140497	-3.130255328	-0.842435852	-6.822716278	-1.877182722	-6.58214246	-5.116007769	-1.905542623	-2.797930302	-0.957408516
-4.949599665	0.256915069	-4.140245148	-1.578889206	-0.516824678	-3.436338419	-0.875228997	-5.187877964	-1.433056822	-5.078122803	-5.231008622	-1.838083698	-1.645155574	-0.83637294
-5.193459169	0.2950524	-4.027908552	-2.393410694	-0.474813871	-3.455122912	-0.572892455	-5.391634989	-1.788834171	-5.091317027	-5.177347079	-2.492724647	-2.325210802	-0.81473896
-4.342160001	1.143203275	-3.813245088	-2.689877024	0.311860419	-3.88365824	-1.032032641	-4.663660443	-1.246552537	-4.343508449	-4.873680482	-2.770589068	-2.347303722	-1.368950874
-4.318243518	0.980757891	-4.101569245	-2.28028407	-0.175143352	-3.07284496	0.158847787	-5.030448761	-1.508227216	-4.474704419	-5.042639314	-2.193761918	-2.354151745	-0.529454722
-5.380379254	0.128566211	-4.183242177	-2.01718932	-1.11252503	-2.943577641	-0.21847071	-5.882093389	-2.07638553	-5.619497141	-5.482996168	-2.432983264	-2.643413765	-1.263221304
-4.567953062	0.69508409	-3.878892741	-2.286514334	-0.165741927	-3.115350608	-0.735295086	-5.16764089	-1.478494757	-4.91987799	-4.959206537	-2.283898255	-1.772478432	-1.062471662
-4.526468087	-0.141789249	-4.075697768	-1.338348288	-0.726228614	-2.974817369	-0.685295905	-5.596915568	-1.79163288	-5.014100572	-5.594238849	-1.782897897	-3.147845742	-1.860485704
-5.086072453	0.161804731	-4.666335934	-2.377436893	-0.387052744	-2.975899976	-0.616634623	-6.06543366	-1.935381563	-5.452316902	-5.982033114	-2.626385645	-3.072338606	-0.653384333
-5.061287315	0.192997888	-4.678308209	-1.511565412	-0.752638335	-2.833984505	-0.381089214	-7.846491133	-1.540977416	-6.350547858	-6.601541101	-2.17136041	-2.727276626	-0.564276964
-4.855334982	0.048236263	-4.390305355	-1.930765089	-0.582330561	-3.000209091	-0.911255645	-5.979813804	-1.680128515	-5.237694207	-5.551592655	-2.571333123	-2.409691172	-0.896243065
-4.813524293	0.055131173	-3.749368098	-1.936022041	-0.704355926	-2.945091798	-0.512066726	-5.68635694	-1.743231978	-5.191977695	-5.379762113	-1.772682721	-2.262051017	-1.356748683
-4.106649026	-0.40011522	-3.790493982	-1.134965632	-0.625251087	-2.76067571	-1.139314358	-5.292055789	-1.812242253	-4.846793005	-5.611202745	-1.407467504	-2.659875361	-2.153784732
-4.108046802	0.047293493	-4.205953807	-1.628902656	-0.340645506	-3.222165577	-1.310919259	-5.780753523	-1.922259581	-4.950280465	-5.433980648	-1.709213154	-2.046645602	-2.012464328
-4.586553036	-0.009697464	-4.146426957	-1.426037304	-0.603729945	-2.843872845	-0.86996723	-5.723972239	-1.867795346	-5.14468842	-5.698898335	-1.895010077	-2.879399196	-1.725531611
-4.754605882	0.197486463	-3.794321609	-1.786765311	-0.505095738	-2.813909579	-0.442272861	-5.522923929	-1.924512294	-4.945932657	-5.434454476	-1.920200274	-2.729276907	-1.402564051
-4.480807214	-0.304932603	-4.379418802	-1.086430051	-0.780062897	-2.662477274	-0.894824365	-6.05338231	-2.064708928	-5.339808896	-5.954218037	-1.546245379	-3.375174371	-1.884518006
-4.100388298	0.072125794	-3.728574914	-1.490569059	-0.201270189	-3.114178676	-1.388384386	-5.701380047	-1.672236291	-5.265683377	-4.984128909	-1.492289432	-1.960050346	-1.646428888
-4.139481881	-0.071196711	-4.109856936	-0.989107848	-0.630847967	-2.696512284	-0.677484143	-5.643904636	-2.031679151	-4.885665459	-5.580549258	-1.438722108	-3.115286611	-1.806280654
-4.229992266	-0.203913856	-4.168138712	-0.919930763	-0.290709846	-2.635202732	-0.82572617	-5.5312234	-1.881536652	-4.822797582	-5.512203934	-1.488146207	-3.089162885	-1.794258744
-5.31558377	0.113808786	-4.367873059	-2.361407854	-0.861107552	-3.090603996	-0.664881161	-5.546068869	-1.679663832	-5.04264596	-5.291443977	-2.756827528	-2.507756732	-0.858520161
-4.649888047	0.768182185	-3.99208497	-1.986911055	-0.317050494	-2.733647406	-0.419760543	-4.947613387	-1.561744035	-4.808295702	-4.776663372	-2.186838583	-2.076464022	-0.285484079
-5.024325894	0.17313727	-3.992767101	-2.006978511	-0.969217016	-2.901386225	-0.501293766	-5.789522831	-1.746624694	-5.178718621	-4.99760847	-1.921836834	-2.500453675	-0.631118836
-5.171229739	0.152762759	-3.776702772	-1.864107812	-1.061741252	-3.146167521	-0.28635465	-5.311572205	-1.767673028	-5.172141023	-5.049829261	-1.964260278	-2.336258701	-0.826282233
-5.189066714	0.280895388	-4.015215031	-2.087486269	-1.048533824	-3.058331432	-0.416542748	-5.006561528	-1.793906159	-5.208124853	-5.300807501	-2.481148253	-1.983927461	-0.597628172
-4.568546977	0.459034476	-3.927900194	-1.013220343	0.145337453	-2.75775899	-0.940654496	-5.116167423	-1.01262557	-5.156413944	-4.99395318	-2.010219115	-2.308080815	-0.488510907
-5.328195854	0.289903398	-3.980155422	-2.317161204	-1.168583431	-2.817827377	-0.109301417	-5.387549881	-1.715976739	-4.988519696	-5.278710209	-2.351683117	-2.498677507	-0.697437179
-5.175538484	0.264911431	-4.152117509	-2.451781287	-0.986780921	-2.784088945	-0.213838077	-5.655019617	-1.807990377	-5.136510556	-5.575464859	-2.30315643	-3.07804563	-0.719850198
-5.198438819	0.196048617	-3.895946636	-1.943369835	-0.840006897	-2.841615815	-0.125177359	-5.683004641	-1.779555235	-5.436679311	-5.481264079	-2.066582576	-3.069069353	-1.13867454
-4.899713617	0.404146507	-4.275736469	-2.566996135	-0.053156711	-3.339226862	-1.311863618	-5.525055697	-1.571432387	-5.061781186	-5.473816656	-2.505179783	-2.83218572	-1.528647013
-4.734600135	0.191244808	-4.516663175	-1.789165551	-0.381056665	-3.84656197	-1.322993495	-5.626697141	-1.950664375	-5.544668091	-5.621391484	-2.695545457	-2.482686712	-1.31775105
-5.548331239	0.004610849	-4.365151721	-2.276114377	-1.14242068	-3.306545255	-0.514998397	-5.58419291	-2.000005527	-4.989854414	-5.576488593	-2.246881265	-2.488170658	-0.879337297
-4.627284628	0.205518662	-4.066486605	-1.516511935	-0.514632445	-3.137957705	-0.48785882	-5.236932878	-1.487667329	-5.273203765	-5.178458136	-1.590387837	-1.446701417	-0.578671865
-5.451593736	0.343894785	-4.104299588	-2.364020946	-0.886962661	-3.098700326	-0.146169892	-5.588433943	-1.724352325	-5.302341344	-5.480562472	-2.540401864	-3.144147812	-0.76642432
-5.52691327	0.144436425	-3.938853707	-1.92662317	-0.969954515	-2.608725575	-0.461805639	-5.716914084	-1.7890707	-5.295045056	-5.359454952	-1.743163172	-2.557710637	-1.058357932

-4.264728389	-0.039837414	-3.763159749	-0.917398266	-0.563226322	-2.68468981	-0.799125479	-5.673082417	-1.951241039	-4.776150733	-5.12107135	-1.367061249	-2.668508786	-1.686045896
-4.387028111	-0.258568178	-3.729021908	-0.897193238	-0.796564763	-2.509657869	-0.619053749	-5.365963077	-1.854881894	-4.842963826	-5.344841193	-1.182728006	-3.23252125	-1.870464131
-4.495626984	-0.134788736	-3.741903092	-0.974180814	-0.864175686	-2.564872424	-0.351085306	-5.517592086	-2.010886744	-4.73714021	-5.436782122	-1.511962723	-3.491997702	-1.808842009
-4.649930622	-0.129774816	-3.819279874	-1.29483245	-0.709218968	-2.614010183	-0.720410377	-5.526590982	-1.860267353	-4.91240711	-5.26034737	-1.695531896	-2.884562532	-1.582620469
-4.823512582	0.039243394	-4.075383631	-1.617084516	-0.757654762	-2.933632439	-0.695442399	-5.508070943	-1.944225729	-5.073244158	-5.60113648	-2.055196758	-3.177506307	-1.789134571

hsa-miR-4708-3	hsa-miR-4710	hsa-miR-4713-5	hsa-miR-4716-5	hsa-miR-4718	hsa-miR-4721	hsa-miR-4722-5	hsa-miR-4723-3	hsa-miR-4723-5	hsa-miR-4725-3	hsa-miR-4726-3	hsa-miR-4726-5	hsa-miR-4728-3	hsa-miR-4728-5
-5.261045927	-4.721821856	-3.8423025	-3.952064739	-6.674088274	-4.196037642	-3.666594912	-3.934173746	0.88957741	0.319165343	-5.779870293	-1.968183699	-3.71053568	-1.760045133
-4.579820562	-4.555441109	-4.115654757	-4.340635806	-5.811158903	-4.317891716	-3.417495751	-4.217683135	0.401835293	0.414198253	-5.929474427	-2.427265596	-4.02825018	-1.974833346
-5.154531154	-4.861132439	-4.143639699	-3.992694339	-6.137787226	-4.231498182	-3.520098649	-4.135912395	1.197163573	0.829701495	-6.033064694	-2.225172025	-3.805720185	-1.908328813
-4.673320681	-4.84493885	-3.961357801	-4.094537614	-6.509942429	-4.237408692	-3.51974711	-3.971029893	1.35764149	0.778130705	-6.051914955	-2.22495527	-3.836928964	-1.870646968
-4.800736696	-4.792988586	-3.677945882	-3.690542313	-6.588399419	-4.523255228	-3.5517742	-3.796744285	1.482757691	0.609378128	-5.743618405	-2.014227375	-3.495708791	-1.858949794
-4.698086665	-4.749270031	-3.823171649	-4.075019741	-5.963870716	-3.939474274	-3.621177166	-4.128257539	0.870434874	0.437464108	-5.711463885	-2.183689285	-3.81346086	-1.913306131
-4.800510546	-4.628587845	-3.802240332	-3.787952673	-6.240387368	-4.310065398	-3.284025496	-3.876031183	1.275855887	0.60093068	-5.837058336	-2.124477301	-3.593663027	-1.842149874
-4.354832343	-4.676000486	-3.999544223	-4.225162413	-5.691023018	-3.867967532	-3.589561901	-4.303024767	0.967489764	0.607236293	-5.921435111	-2.396317793	-3.979473029	-2.16648221
-4.603088956	-4.956775683	-4.115387396	-4.358409027	-5.927602374	-4.01717473	-3.579886708	-4.229231487	1.016870492	0.638676353	-6.249582219	-2.326253814	-4.001580606	-2.011657665
-4.515006491	-4.561803864	-3.914085919	-4.2384719	-5.596496646	-3.922484217	-3.562997677	-4.149931416	0.558611417	0.506859666	-5.738524201	-2.191763547	-3.951891365	-1.99468044
-4.771433527	-4.779844479	-4.484557537	-4.177613614	-5.720884662	-4.496922173	-3.527331952	-4.526173452	1.245368727	0.5496866	-6.071723937	-2.523479967	-3.935992422	-2.286507364
-4.931325976	-4.308855421	-4.66762059	-4.54986746	-7.091406872	-4.485736012	-3.927477943	-4.47599829	0.741706996	0.291970174	-6.903545239	-2.255329228	-4.193495103	-2.131997157
-4.500280629	-4.400896367	-4.741797047	-4.461117233	-7.255986216	-4.486926691	-3.816239571	-4.685016076	1.101928173	0.525269929	-6.475553013	-2.478524195	-4.164986783	-2.125282897
-5.350020205	-5.223843331	-4.609217233	-4.195368037	-6.830889667	-4.604394243	-3.607180938	-4.437841059	1.105658002	0.530814663	-5.975825501	-2.515713477	-4.04284257	-2.097478029
-4.921141479	-5.190901994	-4.472342929	-4.399595508	-6.458967799	-4.753879939	-3.56593172	-4.598101273	0.271625226	0.327198078	-5.796765594	-2.352578005	-4.11920433	-2.415799045
-4.748344285	-5.078982754	-4.536349689	-4.437217192	-6.692921938	-4.435788406	-3.572851167	-4.500799642	1.134564245	0.467731344	-6.183421326	-2.44749638	-4.217486066	-2.356861956
-4.565811274	-5.192971114	-4.461757117	-4.400950098	-5.610399637	-4.240047005	-3.659517311	-4.647258678	0.269154491	0.430834406	-5.889517291	-2.338444242	-4.22894815	-2.367606862
-5.761563909	-5.046148263	-5.022948486	-4.383214147	-6.080741458	-4.493909286	-3.943515822	-4.852353155	0.563309908	0.212697778	-6.405979743	-2.882171421	-4.260248341	-2.218324064
-5.367534289	-4.987411783	-4.518934411	-4.151553826	-6.888195756	-4.716961077	-3.787779955	-4.438714066	1.298616517	0.173464007	-6.043770486	-2.475683601	-4.05702026	-2.075697746
-4.743788846	-5.09263402	-4.540117393	-4.292782903	-6.277455989	-4.225083674	-3.762189665	-4.510005224	0.878937598	0.501001598	-5.949357833	-2.47502108	-4.190815088	-2.272089225
-5.054139502	-4.97739519	-4.554394241	-4.487542031	-5.887298486	-4.879381422	-3.724192859	-4.600319541	0.150496984	0.36927328	-5.991564698	-2.348670408	-4.321748325	-2.188860837
-4.297871437	-4.921105723	-4.526121462	-4.435939744	-4.641149527	-4.899767993	-3.746289335	-4.672372283	0.103989561	0.23767796	-6.210171717	-2.333778194	-4.157225639	-2.520936561
-4.481332149	-4.772257652	-4.435626275	-4.152980219	-5.800148376	-4.624999754	-3.72800408	-4.355466768	1.203418413	0.344924896	-6.23673748	-2.431604189	-3.988118793	-2.061260902
-4.649767836	-4.70874056	-4.07096259	-3.755652328	-6.848821207	-4.787139004	-3.662635844	-3.943019813	1.801728291	0.3662327	-6.003777271	-1.828911673	-3.774966793	-1.506714546
-4.475718261	-4.758710244	-4.581716553	-4.482312458	-4.666362808	-4.712662501	-3.628029658	-4.517677879	0.462073925	0.603866184	-6.234982781	-2.540519205	-4.033993743	-2.381476027
-4.947426738	-4.731692477	-4.064498258	-3.723450735	-6.119118594	-4.553194246	-3.586242896	-3.868747178	1.531152669	0.364216332	-5.803920442	-1.611595142	-3.715905691	-1.56902107
-4.916027307	-4.672340799	-4.748390882	-4.689857582	-7.057641691	-4.287778111	-3.834394968	-4.730891278	0.73672214	0.300817988	-6.745966992	-2.510904725	-4.361115397	-2.365772309
-5.201922375	-4.636793894	-4.526036705	-4.216781402	-6.277827559	-4.499083101	-3.65190295	-4.13775087	0.995167013	0.241948401	-6.389676518	-2.17471022	-3.959531953	-1.910409518
-4.936656569	-4.667173866	-4.621336894	-4.169852963	-5.1832398	-4.423272987	-3.756134174	-4.165354461	0.492993429	0.176625177	-5.978018008	-2.383163397	-3.890801664	-2.046260226
-4.415416714	-4.380097345	-4.336954057	-4.233082202	-5.116084625	-4.323054939	-3.487042005	-4.286226684	0.852583663	0.483937527	-6.231057986	-2.281286681	-4.038242252	-2.080609719
-4.029336213	-4.56850799	-4.274425047	-4.350648495	-6.03392459	-4.15514351	-3.441563187	-4.354009472	1.184982751	0.47582729	-6.475041505	-2.358858612	-4.168726318	-2.109052799
-6.032534065	-4.918542515	-5.184846935	-4.989007645	-8.212006037	-5.098695111	-4.113648157	-4.998254289	0.120829544	-0.647408667	-5.195172067	-2.790967599	-4.884471522	-2.411179834
-3.274109885	-2.982131089	-4.383339039	-4.899598643	-3.510822974	-4.179868745	-3.236903899	-4.697749512	-0.988283572	0.216736089	-6.45369691	-2.596679782	-4.166124677	-2.661406034
-4.104528196	-3.686067611	-4.449711009	-4.895765949	-4.900642619	-4.314138558	-3.284326398	-4.62722579	-0.207109139	0.45308795	-5.712857331	-2.569842026	-4.159283933	-2.564924758
-5.534965012	-5.009938389	-5.021845474	-4.671763828	-5.910478688	-5.025979156	-3.412921143	-4.854042638	-0.041846834	0.537413494	-6.139636763	-2.824001263	-4.356949352	-2.728258361
-4.9382986	-4.949992041	-4.388312889	-4.470690851	-5.210591766	-4.899395138	-3.618930291	-4.259674916	-0.369956681	0.603894793	-5.797258989	-2.472728114	-4.053547886	-2.692239607
-5.061111893	-4.077454078	-6.98997747	-4.658422452	-7.428904475	-5.43694741	-3.35933032	-5.862179476	-0.529790778	-0.307045505	-5.629224252	-3.100840926	-4.640023452	-3.173342632
-4.652027174	-4.626212505	-4.80075543	-4.37096247	-5.021626747	-4.848030618	-3.389249647	-4.666038615	-0.333832471	0.572173589	-5.743232138	-3.146276205	-4.032332289	-2.790557823
-5.119481816	-4.583680739	-4.506163481	-4.270163931	-6.786976409	-4.603459394	-3.578350268	-4.448327806	0.573369183	0.899047393	-5.944175518	-2.780561695	-3.81928258	-2.68757626
-3.591221394	-3.701066236	-4.745863135	-4.582061949	-3.427783777	-4.638117309	-3.658879718	-4.755897115	-0.38476461	-0.308101512	-6.127980575	-2.78231452	-4.399477248	-3.030870563
-4.949148642	-4.801236449	-4.507409818	-4.398840186	-4.905522514	-5.084176183	-3.695965254	-4.47164601	-0.387163036	0.357064038	-6.116161476	-2.707148599	-4.032407289	-2.579872533
-3.94739498	-3.946681087	-4.809802365	-4.714703514	-4.078658146	-3.916114753	-3.511480927	-4.936392162	0.125659828	0.455905352	-5.960988674	-3.007673808	-4.155682893	-2.859275999
-4.159749604	-4.378898452	-5.192497581	-4.897632292	-3.608459786	-4.265152545	-3.403141567	-5.133089079	-0.031054458	0.204160942	-6.007615481	-2.567547142	-4.575880114	-2.98189936
-4.928024871	-4.489409169	-3.937095907	-3.089871919	-6.310673814	-5.124621921	-3.294046015	-3.797614109	2.072915365	0.449904277	-5.584416169	-1.635872974	-3.383663343	-1.634186443
-3.90451822	-4.523661294	-4.779635201	-4.979727015	-5.415298056	-4.560197336	-3.334727834	-5.128307625	0.202372016	0.537966185	-6.103030812	-3.025960656	-4.419161063	-2.815548934
-4.911803941	-5.245388698	-4.872506638	-4.769686923	-5.325230579	-4.637897332	-3.521706966	-4.911563386	0.037566818	0.509316372	-5.691664396	-3.024544431	-4.409279059	-2.890573419
-5.315019497	-4.980352296	-4.33132865	-4.199495042	-7.989626681	-4.629687523	-3.258300475	-4.390859214	0.895241472	1.220199267	-6.094421086	-2.287338831	-3.938014803	-2.561436682

-4.88431557	-4.747771275	-4.795600526	-4.655357249	-5.407455449	-4.347424358	-3.450738272	-4.754886943	0.480600376	0.723477144	-5.931597102	-2.891007425	-4.273762944	-2.463234142
-3.179141187	-3.080469311	-4.667651122	-4.545354708	-4.745298442	-4.70335953	-3.029134353	-4.847641055	-0.194419993	0.106308279	-5.729371263	-2.696155756	-4.412077128	-2.8139785
-4.354112025	-4.340166259	-4.579140863	-4.475123464	-5.003381998	-4.472230104	-3.280127356	-4.459415922	-0.220127319	0.634211669	-6.051973756	-2.585242551	-4.26116811	-2.454519345
-5.279280967	-4.001542255	-4.199399093	-3.35653252	-7.564893613	-5.07065414	-3.384889049	-3.806031625	1.822922795	1.160018579	-5.929428442	-1.968969137	-3.450421936	-1.715266424
-5.00462505	-4.955246854	-4.557467083	-3.903341812	-7.123498263	-4.732264034	-3.689043903	-4.342956845	1.420017866	0.332215301	-5.742258392	-2.224608929	-4.038480356	-2.371210076
-3.399178022	-3.045966189	-5.077637654	-5.022644306	-3.806059277	-3.924637548	-3.163909338	-4.905589044	-0.412172121	-0.208028105	-6.584686253	-2.662395385	-4.53160652	-2.386746199
-2.985580376	-2.929031154	-4.695861061	-4.827156721	-3.102863616	-4.452695977	-2.717715241	-4.71293778	-0.649363899	0.131113131	-5.645826415	-2.370705185	-4.520329172	-2.13080446
-4.753369653	-4.802693866	-4.477369497	-3.98121385	-6.646959136	-4.737662436	-3.311740778	-4.440658149	1.647334919	0.014304458	-5.066753419	-2.351829515	-4.043769752	-2.445868164
-3.968196944	-4.688196101	-4.678934301	-4.871876841	-3.57195679	-4.181782933	-3.405201015	-4.681701554	0.302756422	0.359441085	-5.759897797	-2.625393934	-4.26509607	-2.60829932
-4.905410375	-4.898551867	-4.81720789	-4.805899188	-6.239340424	-4.196566624	-3.533906713	-4.763783578	0.951891234	0.320071476	-5.57292358	-2.809739296	-4.400066361	-2.690571602
-4.247520733	-4.943816554	-5.259299894	-4.755228105	-4.783787838	-3.386144457	-3.433331404	-5.385261473	-0.624795861	-0.065890026	-5.591562338	-3.160654032	-4.618461311	-2.89133176
-4.44441689	-4.8349365	-4.687044599	-4.888195244	-6.439326139	-3.90813647	-3.49662833	-4.77483714	0.444987858	0.544792877	-5.365159294	-2.861434421	-4.498651193	-3.051087134
-5.498488985	-4.734430367	-4.943226508	-4.61908294	-5.07406478	-5.167144462	-4.379352189	-4.791587766	0.478685217	-0.103798017	-6.26345569	-2.972614622	-4.362033574	-2.969042029
-3.870389788	-2.820084952	-5.31162369	-5.053865688	-1.977323635	-4.170827097	-3.652651342	-5.220237341	0.396426652	0.019723675	-5.911626627	-3.409068504	-4.559245156	-3.061884348
-0.068922211	-2.252439263	-6.555338171	-4.951655617	-1.496826324	-4.714203518	-3.265862893	-5.321690258	-0.710222804	-0.364237983	-7.032286726	-3.335807399	-4.975198553	-2.901332275
-1.859319646	-1.196861583	-5.379507672	-4.738852849	-0.563435287	-4.781616661	-3.258350009	-4.980588632	-0.603686469	0.043551153	-6.930109334	-2.863789312	-4.269935096	-2.50696695
-3.593590136	-4.942291791	-4.963495179	-4.582236944	-4.145078834	-4.606485287	-3.728153605	-4.647697809	-0.392545103	0.153276742	-6.445542676	-3.032397414	-4.059361276	-2.814243134
-5.374172352	-4.607174805	-4.238143379	-3.960940383	-6.924905487	-4.381521475	-3.899622005	-3.636331071	0.628442586	0.368366526	-5.576073898	-2.41841997	-3.440563915	-2.380483834
-4.44682154	-4.371396787	-4.522108777	-4.327760866	-5.432116862	-4.282100166	-3.25183024	-4.463543927	1.209379488	0.856830055	-6.04459585	-2.352591866	-3.976290372	-2.421231407
-3.990982313	-4.531641423	-4.989053613	-5.125008454	-5.958473126	-4.271608028	-3.570226593	-5.29942475	0.079482617	0.26281964	-5.816706258	-3.042577612	-4.697693943	-2.647749847
-3.433711301	-4.678393881	-4.307893673	-4.34518781	-4.233239093	-4.300793977	-3.548905138	-4.404661294	0.662336431	0.793193874	-5.993284741	-2.53480539	-4.226309105	-2.274074262
-3.475662393	-3.581293835	-4.684118883	-5.068171118	-4.786562076	-3.860560402	-2.869145135	-4.980652411	0.504287638	0.214936611	-6.342554839	-2.462487394	-4.577760372	-2.052849812
-5.079756506	-5.193037303	-5.326052751	-4.961648357	-8.076628314	-4.51039761	-3.721864455	-5.02847203	0.168476201	0.51115958	-7.005087516	-2.788205887	-4.708756261	-2.472685902
-3.561693873	-3.456569487	-5.714487986	-6.101983061	-3.690097639	-4.533661053	-3.576286756	-5.761521675	-0.767739226	0.051687639	-8.145245788	-2.527178387	-5.166513046	-2.494596034
-4.358424961	-4.532740646	-4.872490473	-4.915535774	-7.10280871	-4.535088482	-3.41928846	-4.881680008	-0.687313099	0.425464599	-7.103986894	-2.776813943	-4.489129728	-2.203217488
-4.516436687	-4.403731818	-5.172928638	-4.684119147	-4.559806868	-4.048875309	-2.893514324	-5.137811489	0.275051766	0.409270288	-6.372721963	-2.71165729	-4.561011514	-2.202675862
-4.31810387	-3.685932972	-4.603921872	-4.920929002	-4.843845993	-3.573133965	-2.484459597	-4.988942132	-0.292463126	-0.28637582	-5.792089044	-1.98378641	-4.523821024	-1.662987277
-4.441976076	-4.365931624	-4.457851931	-4.910228153	-5.963736438	-3.861066049	-2.818870168	-4.819173858	-0.222895008	-0.263125096	-6.580102346	-2.036395621	-4.59134363	-2.043437081
-4.621615231	-4.356756331	-4.515501609	-4.917766097	-5.91768693	-3.808807452	-2.853090743	-4.807919746	0.336721055	0.206441815	-6.599858958	-2.402757003	-4.47300538	-2.077288976
-4.458851908	-4.922592827	-4.72932512	-4.622742475	-5.505503244	-3.966160189	-3.083562356	-4.876233958	0.872219904	0.460846573	-6.26194142	-2.647238552	-4.345424731	-2.230383856
-4.386719181	-3.857727198	-4.888899593	-5.21221335	-5.728795574	-3.508796806	-2.747129249	-5.011248936	-0.304287211	0.16476019	-7.275834802	-2.169582869	-4.742004497	-1.882472896
-4.389392394	-4.1097349	-4.807712608	-4.936141734	-4.99467252	-4.469655338	-2.718298249	-4.84444194	-0.198195947	0.016994365	-7.431379876	-2.166753612	-4.536448039	-1.98234972
-3.742970836	-3.590309624	-4.623616892	-5.188001105	-3.059690109	-3.767452564	-2.690876269	-4.901523299	0.165507843	0.067235479	-6.419931416	-2.196859214	-4.769187884	-1.725284025
-3.556936001	-3.083248097	-4.269234957	-4.92265904	-3.925133669	-3.744335755	-2.658167143	-4.849340759	0.056017631	0.119151079	-6.119669879	-2.035497226	-4.556800335	-1.42066008
-4.999184576	-5.285722432	-4.838559468	-4.80750451	-5.702522381	-4.435108546	-3.786735112	-4.830432907	-0.605928966	0.649927268	-6.077000956	-3.310683969	-4.321198731	-2.703328538
-4.41573902	-4.459546759	-4.58658675	-4.358265498	-4.919313857	-4.729041725	-3.662127251	-4.40420955	-0.089710473	0.981743681	-5.800191745	-2.707392277	-3.940331849	-2.544175959
-3.479064804	-2.753913583	-4.862496576	-4.768169566	-4.004795828	-4.081238341	-3.149255636	-4.901651343	-0.382506666	0.461361685	-5.514035944	-2.828763493	-4.148369783	-2.778283123
-3.541833502	-3.314601641	-4.815750573	-4.641034668	-4.196520357	-4.383641059	-3.244330443	-4.801666966	0.020779394	0.565408332	-5.673793906	-2.897733498	-4.342611916	-2.782592414
-3.731993236	-4.463829475	-4.844758484	-4.805700139	-2.663925721	-4.609868135	-3.536229683	-4.773966418	-0.49326678	0.456263214	-5.558320287	-2.824993035	-4.282176338	-2.870183867
-3.408603805	-4.690629314	-4.666668226	-4.511276608	-3.151275117	-5.033355947	-3.064141701	-4.500867055	0.299411204	-0.218226212	-4.994677556	-2.542963699	-4.098092877	-2.276386728
-4.392207193	-4.696645028	-4.617546983	-4.592219801	-6.420946495	-4.152616377	-3.484889703	-4.998816423	0.046581993	0.539030908	-5.448635759	-2.876909874	-4.405956959	-3.045285047
-4.755690446	-4.883970444	-4.897431528	-4.949190993	-6.019185988	-3.866426877	-3.722044858	-4.733621849	0.428565984	0.609378044	-5.674663627	-3.002913929	-4.62663201	-2.909739757
-3.840821709	-3.92536659	-5.146570625	-5.200385525	-4.646974743	-3.848868129	-3.277123531	-5.283352672	1.172494963	0.384459403	-6.220471699	-3.082054547	-4.657012054	-3.035531095
-4.214977354	-2.994154834	-5.069258603	-4.864288472	-2.838632652	-4.774357608	-3.95291334	-4.968677588	0.700738221	-0.585682881	-5.623657326	-3.134811312	-4.548089477	-2.995604846
-3.421510105	-3.447094632	-5.441710257	-5.000577956	-4.126539639	-4.812908804	-4.33617345	-5.029009103	-0.203504645	-0.307296117	-6.319194872	-2.925340942	-4.812468922	-3.197079308
-2.556417939	-2.995100467	-5.163702496	-5.086626325	-2.30121161	-4.374115375	-3.63325731	-4.991163101	-0.566121814	-0.036082482	-6.330015621	-3.375117942	-4.460541139	-2.761960366
-2.859247217	-0.89727444	-5.66701941	-4.922902472	-3.143122142	-4.739809461	-2.991822479	-5.042727013	-0.771127857	0.18275427	-8.162214668	-2.724609913	-4.272351902	-2.709424321
-5.094977068	-4.822745055	-4.93848497	-4.810308115	-6.867847852	-4.431495992	-3.890948685	-4.700716042	0.566996862	0.442937095	-5.714767267	-3.425633921	-4.143650156	-3.152865597
-3.157408912	-3.35313893	-4.798216203	-4.940081354	-3.645872746	-4.184641582	-3.032226794	-4.979997561	-0.223421271	0.16140753	-4.949293545	-2.862662782	-4.503228108	-2.943461949

-4.014497487	-3.761190711	-4.458545764	-4.838334308	-5.668313461	-3.489083133	-2.314830296	-4.693302406	0.049487232	0.278744168	-6.782613548	-2.006607199	-4.472754128	-1.731727332
-4.310605953	-3.623471701	-4.388952089	-5.02454162	-4.973753122	-3.305073414	-2.286264624	-4.969495309	0.069698458	0.208113316	-6.351008007	-2.095352362	-4.430185176	-1.524833909
-3.746057944	-3.945021261	-4.491486794	-5.203360173	-5.866686564	-3.322020615	-2.480667053	-4.824585301	0.133076303	0.338400685	-6.842338888	-2.361009855	-4.629101654	-1.748911891
-4.411502177	-4.110464941	-4.428954649	-4.908657236	-5.934889257	-3.628856664	-2.776684558	-4.468626187	-0.067730359	0.253219595	-6.766199808	-2.44353815	-4.40803756	-1.854509201
-4.8165342	-4.548538384	-4.584460402	-5.105583798	-6.70789553	-3.65435966	-3.014162568	-5.01636946	0.590873412	0.340409997	-6.857962828	-2.579152762	-4.641184274	-2.197604927

hsa-miR-4731-3	hsa-miR-4731-5	hsa-miR-4732-5	hsa-miR-4734	hsa-miR-4736	hsa-miR-4738-3	hsa-miR-4740-3	hsa-miR-4740-5	hsa-miR-4743-5	hsa-miR-4745-5	hsa-miR-4746-3	hsa-miR-4747-3	hsa-miR-4749-3	hsa-miR-4749-5
-3.536774083	-2.709972051	-2.8295963	1.553269421	-3.801096043	-4.849431497	-4.088102004	-6.438422034	-4.621607047	-0.076781929	-2.707466082	-5.385689126	-4.001978797	-1.384289879
-3.995501382	-3.184622973	-3.751974585	2.211249248	-4.290443023	-4.755197703	-3.351806924	-6.641374144	-4.35202842	1.095904454	-2.498122248	-4.193568544	-4.348704018	-1.091006704
-3.756499358	-2.898716803	-3.688087142	1.932537265	-4.431209389	-4.600670031	-2.83545555	-6.591935395	-4.262014656	0.89855726	-2.291765091	-4.266250773	-4.020387444	-1.01476958
-3.765437314	-2.97099935	-4.322968251	1.907680089	-4.745882469	-4.731637647	-2.902140162	-6.811621221	-4.565842079	0.855716292	-2.335766566	-3.9636154	-4.11482921	-0.947559279
-3.232820056	-2.790853043	-3.402208171	1.89643485	-3.830361909	-4.817660157	-3.057896235	-6.415503284	-4.562513152	0.61225359	-2.720564989	-4.411438723	-3.724893411	-1.341762381
-3.623833016	-2.925223106	-3.880669736	2.102302527	-3.933918109	-4.379445252	-3.688291385	-6.590336273	-4.470833291	1.094682982	-2.417984623	-3.974904038	-3.848543684	-1.129331803
-3.401088055	-2.641294903	-3.565186299	2.055006752	-4.332147655	-4.680845179	-3.111776281	-6.644497183	-4.365748028	0.588435675	-2.459052482	-4.240555458	-3.80106109	-1.003482081
-4.019406638	-3.27198945	-3.991364441	2.154575114	-4.317772874	-4.474121071	-3.073024762	-6.764170013	-4.468842286	1.249092133	-2.364596773	-3.591660993	-4.185353994	-0.915783305
-3.950411999	-3.083839598	-3.794635866	1.947062736	-4.274481744	-4.546721075	-3.19152429	-6.567117736	-4.48373604	0.963038606	-2.470205499	-4.286720578	-4.208229557	-1.122007019
-3.881274293	-3.0657572	-3.492999638	2.104018964	-4.293218401	-4.358995952	-3.163736577	-6.316952021	-4.258172917	1.177807734	-2.527472679	-4.061785763	-4.036352154	-1.116408572
-3.961374178	-3.139444506	-4.335508855	1.826823721	-4.80378513	-4.572611516	-3.190960446	-6.360971429	-4.42050129	1.147248216	-2.740440079	-3.658606322	-4.114140041	-1.078037823
-4.270137327	-3.132533541	-3.308960179	1.760319405	-4.18580749	-4.696278581	-3.357830796	-7.198438968	-4.809623362	0.574698445	-2.436377499	-5.020589421	-4.632901486	-1.110493271
-4.274098255	-3.203798066	-3.50781983	1.587628738	-4.417827624	-4.805749218	-3.471424559	-7.216370743	-4.710069298	0.797032152	-2.621517584	-4.78710691	-4.610333065	-1.333791333
-3.979167396	-3.05343438	-4.605184581	1.835863787	-4.872891245	-4.691570582	-3.325898185	-7.209438474	-4.671260638	0.90026683	-2.531309777	-4.451331047	-4.172161307	-1.120845732
-4.156793752	-3.302782179	-3.796583008	1.968543041	-4.37032239	-4.746926578	-3.429287471	-7.080752521	-4.534663995	0.94626023	-2.588004859	-4.401927386	-4.248205955	-1.168867898
-4.086137127	-3.341057361	-3.659325287	2.035108293	-4.552365646	-4.399708651	-2.93994512	-6.96777942	-4.601349172	0.65082584	-2.155321596	-4.436879839	-4.304880591	-1.275381187
-4.201236415	-3.209030932	-3.555889717	2.062467861	-4.038636548	-4.35401802	-3.210781073	-6.540036438	-4.807564288	1.233686157	-2.371944486	-4.476813351	-4.16516248	-0.946035696
-4.414760682	-3.283836427	-3.54954069	2.040511189	-5.183764664	-4.772254273	-3.1606293	-6.567661607	-4.788283205	1.036963325	-2.601478157	-4.298032086	-4.151477409	-1.402146572
-3.975643793	-2.999388751	-3.081091277	1.693381652	-4.338025234	-4.850736876	-3.397133848	-6.805312551	-5.006201702	0.798417359	-2.809910765	-4.606473404	-4.105122779	-1.420167899
-4.186424369	-3.209512338	-3.36354974	1.952517697	-4.394765842	-4.64968568	-3.105914653	-6.766689218	-4.801388987	1.266335683	-2.426979749	-3.999242335	-4.099802442	-0.925207836
-4.211667207	-3.057132962	-3.049530199	1.99818054	-4.215887286	-4.561652422	-3.537311745	-6.369340207	-4.648687996	0.937837876	-2.525359067	-4.16104645	-4.247146769	-1.052636269
-4.064729331	-3.215485558	-3.772572058	2.043668185	-4.390728403	-4.742123084	-3.809475042	-6.556494388	-4.798626462	0.784134215	-2.454883548	-4.495155784	-4.360778125	-1.220369627
-3.861689736	-3.126936607	-3.964255875	1.783206777	-4.810572864	-4.867592297	-2.941542508	-6.947881427	-4.697605636	0.997493953	-2.609391268	-4.094309047	-4.135118555	-1.071880436
-3.502404799	-2.383466632	-3.270033709	1.695904864	-4.018387791	-4.646853471	-2.705747282	-6.524924824	-4.777080618	0.554946973	-2.438005625	-4.483727666	-3.780965688	-0.979856267
-4.109066	-3.643486488	-4.495587118	2.13838841	-4.852938063	-4.792966504	-3.000696129	-6.217511267	-4.626145626	1.233879321	-2.649507214	-4.049214586	-4.183080237	-1.160369769
-3.412321209	-2.593338311	-3.532559808	1.695758845	-4.225815649	-4.627040434	-3.036778932	-6.609139551	-5.07285443	0.50610776	-2.300496603	-4.69194398	-3.68278465	-0.980530279
-4.244405223	-3.422367876	-3.867168454	1.759670498	-4.259405008	-4.928630088	-3.579726787	-7.700615929	-4.762851357	1.052730601	-2.62307726	-4.353319646	-4.595866147	-1.197346849
-3.845732677	-2.820719685	-3.598183252	1.424740469	-4.367188431	-4.852810033	-3.422548776	-6.798868418	-4.76771573	0.29575254	-2.751973848	-4.960485257	-4.14009906	-1.311131628
-4.068189817	-2.863370376	-4.086743266	1.930530817	-4.61455177	-4.866871274	-4.003562331	-6.283016128	-5.098531033	0.731429139	-2.948559178	-4.91568959	-4.318068322	-0.99056071
-4.103090513	-3.1547492	-3.858010546	1.932315395	-4.74149797	-4.722070558	-3.050145016	-6.519718463	-4.514892668	0.622666122	-2.584414543	-3.742971682	-4.28900329	-0.955768154
-4.252891976	-3.151624805	-3.820763552	1.820459291	-4.389286717	-4.723969692	-3.000444884	-6.731334458	-4.33344644	0.706452196	-2.322236184	-3.843674656	-4.375807335	-0.737992342
-4.529135399	-3.047327484	-4.814257619	1.192203489	-4.181046146	-5.304827642	-5.317649677	-7.633569632	-5.437011334	-1.005074247	-3.827989072	-4.570480373	-5.048446088	-1.57648209
-4.65387346	-3.531036685	-4.099355252	2.214836784	-4.253349693	-4.773002574	-4.063768231	-6.623195677	-4.139548957	0.157278798	-2.511248457	-3.952011572	-4.34766221	-0.995528666
-4.560295221	-3.232668126	-4.200790755	2.377054259	-4.731776814	-5.172650393	-2.893476225	-7.33275063	-4.214771415	1.842424741	-1.398657314	-3.25810474	-4.370680139	-0.203185309
-4.324419631	-3.570527812	-4.774231826	1.740709266	-5.382328419	-5.067996584	-3.310319976	-7.658763328	-4.876370816	0.301398684	-2.240286054	-4.705502029	-4.436244029	-1.054280107
-4.026335616	-3.321780577	-3.98292907	2.763638226	-3.902707956	-4.853300073	-3.147566916	-6.711728928	-4.747323356	1.592115721	-2.1371418	-4.15947144	-3.775917923	-0.735263827
-4.38432561	-3.792852985	-4.321044388	2.271771672	-3.8560719	-6.294283963	-5.106258122	-7.119052021	-5.172202508	2.1073471	-2.048557095	-3.921015879	-4.323654671	-0.867070706
-4.209068149	-3.593562507	-4.665765882	2.206286314	-4.896253686	-4.950870915	-3.093808549	-6.312185823	-4.520642975	1.563255854	-2.082204057	-3.705535321	-3.983865878	-1.127354031
-4.063041014	-2.422010267	-4.576435908	2.123589361	-4.654838345	-4.884295015	-2.885430885	-7.388971919	-4.865944212	1.247312774	-1.968352303	-4.244514803	-4.193997208	-1.046856685
-4.116764791	-3.470758161	-4.036546927	2.296398137	-3.743281989	-5.203029677	-3.911779563	-4.908401114	-5.024827183	2.231848504	-2.361200452	-3.916756168	-3.94318776	-1.1779003
-4.083693719	-3.189765976	-3.995434767	2.202325484	-4.046248493	-4.774394397	-4.256016952	-6.552860085	-4.870120764	0.048309632	-2.642115377	-4.3931905	-3.997922397	-1.317918615
-4.703556905	-3.830450851	-4.380686889	2.211430006	-5.051181104	-5.803028847	-3.18344436	-6.307077168	-4.740750067	2.62622967	-1.491638636	-3.267380563	-4.230147495	-0.330090524
-4.838660689	-4.309075878	-4.688649153	2.032246153	-4.67102218	-5.060509826	-3.948643343	-3.729864637	-4.717199932	2.4158068	-1.695100057	-4.135357763	-4.576437114	-0.548188931
-2.807046423	-1.934274364	-2.22833831	1.81382723	-3.120814246	-4.87619059	-3.134307558	-6.007730949	-4.748496271	-0.13929487	-2.416203309	-5.033521578	-3.088906353	-1.149096606
-4.452957759	-3.888526032	-4.159408923	2.258036796	-4.295636969	-5.703240216	-3.596634346	-5.237057425	-4.650879499	2.412526926	-1.614676636	-4.087123578	-4.300638072	-0.618887596
-4.474740953	-3.726172988	-4.423891385	2.267124675	-4.855287019	-4.96839937	-3.62501568	-6.333065978	-4.697226309	1.550441275	-2.026779494	-3.95145031	-4.430674395	-0.970134716
-3.795631376	-3.233590087	-4.087051678	2.194361395	-4.285806028	-4.779699772	-2.726150763	-6.959627288	-4.91134487	-0.346527769	-2.513639343	-4.202470199	-4.057369481	-1.313752744

-4.620562154	-3.781028289	-4.119459911	2.255177804	-4.488150517	-5.026731423	-3.117292226	-6.311667241	-4.646977831	1.49359621	-1.975514844	-3.493662003	-4.257874066	-0.741177329
-4.31227104	-3.606665462	-3.499369222	2.783271245	-3.592784536	-4.810419794	-4.350892256	-4.453724091	-4.049708777	2.228117025	-2.070782398	-3.947472257	-3.811782499	-0.78980613
-4.202142693	-3.45165195	-3.737172954	2.629555718	-4.025869014	-4.491770892	-3.484082584	-6.000160256	-4.480889821	0.404905493	-2.346628884	-3.635184229	-3.943651027	-1.149354154
-3.190813685	-2.081522487	-2.225047614	1.766885134	-3.491270708	-4.86990611	-2.084243026	-6.235561756	-4.866918882	0.481984644	-2.273908874	-4.899306881	-3.253832652	-1.135287253
-3.508692559	-2.743748978	-3.920340679	1.659870188	-3.738253159	-5.283631768	-3.550313855	-6.499772432	-5.107440148	1.342517813	-2.279262199	-4.391744384	-3.864616641	-0.991491298
-5.212337306	-4.053127568	-3.571703501	2.271636875	-4.062667611	-4.927520806	-3.453361332	-5.240830162	-4.690896464	2.366788666	-2.152180251	-3.477658489	-3.891606716	-0.747511858
-4.326446482	-3.130658444	-3.652101824	2.813305949	-3.59751418	-4.453422767	-4.022931562	-4.140524488	-4.670378225	1.672371087	-2.218419837	-3.869200038	-3.147508552	-0.782145975
-3.529806371	-2.438608236	-4.130261805	1.999452876	-3.596899088	-4.927753124	-3.673300862	-6.171186466	-5.329301999	0.058972006	-2.505407732	-3.707087623	-3.888778818	-1.475856657
-4.495190853	-3.469035796	-4.389467305	2.423523437	-4.527024535	-5.096816959	-2.971039305	-4.829238516	-4.363611944	2.234411471	-1.647751551	-3.66008398	-3.813822998	-0.34106451
-4.539686809	-3.388812041	-4.126912024	2.192387467	-4.514911976	-5.049601043	-2.662575525	-6.859432281	-4.770451512	2.0428044	-1.586580791	-3.460865718	-4.163910168	-0.3855259
-4.679056635	-3.697814116	-4.950121503	1.790730916	-4.734314206	-5.252855499	-4.290959072	-6.49360697	-5.099050512	2.058132763	-1.90267504	-4.087333061	-4.758679616	-0.721471934
-4.366300519	-3.787444843	-4.754007992	1.994340124	-4.364519707	-5.07065273	-3.233214636	-6.735408583	-4.617394976	1.87874075	-2.060784143	-2.991957079	-4.725272363	-0.741978542
-4.18641818	-3.358631469	-4.299182328	1.890199588	-4.483972381	-5.30105124	-3.971835977	-7.422614446	-5.518220708	1.128805265	-2.677993077	-4.529556685	-4.383572854	-1.159659732
-4.703019868	-3.857539088	-5.13016738	1.830931292	-5.563084806	-5.534112694	-3.910365298	-5.539965939	-5.142160482	1.938352876	-2.134822827	-4.06676971	-4.186279176	-0.81736214
-5.190332781	-3.738389053	-4.733342821	1.982963489	-5.211379092	-6.25178544	-4.714079396	-5.929007111	-5.381834119	1.250019196	-2.974383665	-3.914419587	-4.226624757	-1.014562781
-4.353148538	-3.588329282	-4.528506456	2.343776507	-4.006923827	-4.761264203	-4.25387553	-5.084105606	-4.78496931	1.032897313	-2.300041133	-4.136235705	-4.276087166	-1.027422746
-4.525127394	-3.214739989	-4.965956251	2.236543165	-4.502090618	-5.145797491	-3.943355996	-5.996268184	-5.104530537	1.774841165	-2.366636591	-3.349563042	-4.116856364	-1.074250984
-3.524217299	-2.906711255	-3.534436611	1.978043964	-4.187814844	-4.780600893	-3.261837036	-6.604103913	-4.978540807	0.907413605	-2.503107459	-4.62483961	-3.654984073	-1.411704071
-4.156132726	-2.933435303	-4.337098844	2.177520637	-4.33309847	-4.687530559	-2.976705388	-7.106517429	-4.79356719	1.077479009	-1.704467848	-3.988325172	-4.131995572	-0.474067261
-4.870310114	-4.111948319	-3.928221271	1.977197249	-4.992449279	-5.285293915	-3.670572605	-6.907490148	-4.55828211	2.14856255	-1.644721098	-3.419216816	-4.355270686	-0.40456005
-4.021475316	-3.189007916	-2.962489185	2.297324748	-3.386633975	-4.66356886	-2.823602292	-5.92510609	-4.854550313	2.115021187	-2.04448683	-3.754646294	-4.244008463	-0.671894799
-4.940051973	-3.579801428	-3.851077325	1.599122864	-4.269480469	-4.721572783	-4.732041879	-6.138999368	-4.100516008	0.815275968	-2.847950627	-5.051615063	-5.06146664	-0.937164514
-4.832529924	-3.598538767	-4.850457459	1.454542766	-5.277506879	-5.097730149	-3.119920074	-7.646422335	-5.04382068	0.293528112	-2.194081499	-4.909552845	-5.105603215	-0.931038994
-5.731480092	-3.656421982	-4.299890949	2.129269452	-4.500663551	-5.355346719	-4.837710505	-6.836365008	-4.679200177	1.570987896	-2.463626914	-4.047309945	-5.277581479	-0.694190009
-4.649459292	-3.591696698	-4.263470875	1.734213432	-4.529419282	-5.020526664	-4.429741745	-8.493380371	-4.565584463	0.328751261	-2.780665596	-4.256039768	-4.842547801	-1.074899596
-4.583633514	-3.640707277	-4.270603448	1.982646379	-4.472749601	-4.603857492	-4.38473253	-6.694342825	-4.239815447	1.68538936	-2.495047173	-4.594576156	-4.099252967	-1.125759098
-4.489818965	-3.226187827	-3.889181777	2.056665718	-3.849250344	-4.258725713	-5.186235946	-5.868195136	-3.982339298	0.758343142	-3.067633476	-5.112604152	-4.650382692	-1.218306978
-4.374085969	-3.275964552	-3.42926536	2.125105589	-3.606892964	-4.352355868	-4.691801218	-6.462524097	-4.189499667	1.214839624	-3.136009215	-5.21698704	-4.778092066	-1.150651987
-4.728045394	-3.317352533	-3.869380563	1.919054348	-4.425195293	-4.702869228	-4.500247089	-6.437036343	-4.146631581	0.951142736	-2.851611602	-5.035422288	-4.549551726	-0.936762328
-4.440359286	-3.521659971	-3.962382144	2.008161973	-4.23315793	-4.51341326	-3.967565492	-6.242893014	-4.452027485	1.345736609	-2.472427571	-4.49372764	-4.225031727	-0.817845104
-4.876725466	-3.45782982	-3.729115264	2.064537196	-4.749453694	-4.684796275	-4.99722735	-6.487881163	-3.882532782	1.243213073	-2.921606287	-5.081102717	-4.667637978	-0.813863257
-4.495912716	-3.138937526	-3.631259517	2.313989818	-4.190465914	-4.545580427	-4.722987709	-6.062932439	-4.395158502	0.996177759	-2.783243929	-4.69777291	-4.303146919	-0.920166047
-4.744695889	-3.399047785	-3.655266929	1.717192787	-4.174866894	-4.53901407	-5.023940538	-5.883753329	-3.943804089	0.691128529	-2.79041912	-5.165151847	-4.733438659	-0.716932512
-4.776626964	-3.301523484	-3.763814846	1.779185434	-4.253522133	-4.362429291	-4.973263931	-5.711488022	-3.911589592	0.804106229	-3.077877534	-5.263698333	-4.743049595	-0.860221308
-4.512258553	-3.70175336	-4.58185626	1.834015709	-4.912402704	-5.23588026	-3.095256861	-7.139527001	-4.950234944	1.185261662	-2.223017193	-3.127049029	-4.625887115	-0.855659054
-4.008407238	-3.325797596	-4.429119255	2.535390417	-4.805468443	-5.072958935	-2.803126767	-6.875376317	-4.511777648	2.13105303	-1.512948905	-3.76749238	-4.00440277	-0.704135873
-4.556218399	-3.901783735	-4.722724323	2.175483734	-4.81068379	-5.239950718	-3.858269775	-6.246509771	-4.768666236	2.45262371	-1.771193065	-2.795231587	-3.986114387	-0.412532175
-4.403179175	-3.821608261	-4.542429285	2.181556852	-4.201119353	-5.021221681	-3.758574648	-4.983850469	-4.476196634	1.871589626	-2.214701076	-3.057634692	-4.124397963	-1.065081247
-4.42322881	-3.775412259	-4.528571903	2.377931002	-4.409786106	-5.14044208	-3.696524623	-4.39791164	-4.651324412	1.365772797	-1.761965563	-3.77211097	-4.049011083	-1.068956603
-3.959780226	-2.764579736	-4.746751466	2.18449798	-4.021746011	-4.948096887	-4.771701564	-4.911282477	-4.786494349	1.08544675	-2.269517865	-4.501972101	-3.738695014	-1.148916382
-4.466962932	-3.816325224	-4.663832388	2.00920059	-4.560911788	-4.929385321	-3.232046346	-6.106861951	-4.538068715	2.191790321	-2.17437785	-2.907054002	-4.221889184	-0.714479144
-4.799814348	-3.981950022	-4.817236596	1.98321526	-4.779803789	-5.254631052	-3.20704101	-7.004318871	-4.870867975	1.812011684	-2.060283627	-2.851515751	-4.549424446	-0.445776452
-4.799864311	-3.463705142	-4.41295018	1.783424772	-4.385433497	-5.72600705	-3.532627273	-6.311220939	-5.087956524	1.816265121	-1.883494707	-4.525788955	-4.667284303	-0.623739169
-4.380720188	-3.123217236	-4.958525873	1.42009262	-4.567126491	-5.668158626	-4.744543991	-6.574761439	-5.221904313	0.861303662	-2.485099423	-4.710577448	-4.492542317	-1.093273403
-4.800717804	-3.335102783	-4.746789901	1.798491404	-4.620426129	-5.164240993	-4.365424714	-6.992880883	-5.279235279	1.634007704	-2.885049039	-4.011666158	-4.804350127	-1.117250717
-4.403474359	-3.832707933	-4.675869076	2.249888687	-5.09781455	-5.935964173	-4.00948278	-6.389069699	-5.102842765	2.019147076	-2.272520916	-3.135716216	-4.248885191	-0.80955444
-4.427341842	-3.470600523	-4.905640083	2.203960285	-3.951606645	-5.129142142	-4.131975947	-4.280360269	-4.471225221	1.449645859	-2.247778611	-3.773638556	-3.629081091	-1.077302885
-4.756519578	-3.543221556	-1.273296264	2.122332361	-5.251966903	-5.350712294	-3.218166784	-7.398240862	-5.367823446	1.938649192	-2.182761657	-2.940481804	-4.245561308	-0.868295943
-4.641268869	-3.895619064	-4.125257116	2.393754842	-4.197811447	-4.823090522	-4.43124306	-6.158738203	-4.46263857	1.984341952	-2.179803238	-2.544115548	-4.318524995	-0.772579442

-4.613004186	-3.266848654	-3.624414643	2.186708658	-3.929730815	-4.291918791	-4.670588529	-6.229726993	-3.791380774	1.067364812	-2.821353072	-4.865091554	-4.409084173	-0.674205233
-4.368599908	-3.280402443	-3.855518745	1.897376106	-4.020786609	-4.102702965	-4.83218699	-6.010241033	-3.617444306	1.254846577	-2.831487604	-4.801374939	-4.534154961	-0.978552907
-4.724551473	-3.620277586	-3.641883323	2.003836169	-4.135652773	-4.499749183	-5.049529021	-6.610306718	-3.844238889	1.27902543	-2.450942909	-4.532929496	-4.273127702	-0.504977302
-4.646510984	-3.525226352	-3.670298693	2.201247236	-4.347162257	-4.613597641	-4.675956877	-6.442353903	-4.012798325	1.20493034	-2.815253563	-4.775009959	-4.52975463	-0.883705105
-4.781415335	-3.694273777	-4.252530862	1.646384471	-4.549997877	-4.834329627	-4.382436476	-7.096090622	-4.2243092	1.001541952	-2.876642487	-4.90321558	-4.919591166	-0.866849768

hsa-miR-4750-3	hsa-miR-4750-5	hsa-miR-4751	hsa-miR-4756-5	hsa-miR-4758-3	hsa-miR-4758-5	hsa-miR-4763-3	hsa-miR-4763-5	hsa-miR-4767	hsa-miR-4769-3	hsa-miR-4776-5	hsa-miR-4783-3	hsa-miR-4783-5	hsa-miR-4787-3
-5.132762415	-3.133291204	-4.549290385	-5.952605425	-3.553062572	-0.636198485	-0.737025807	-4.005840831	-5.422136146	-3.988754056	-4.166852132	-4.759364415	-6.756181228	-5.34047284
-5.240512741	-2.701413316	-4.581692192	-5.907896655	-3.794185459	-0.294212829	-0.103053745	-4.138893413	-5.098956275	-4.318502374	-5.115670405	-4.030434337	-5.858125579	-4.928069669
-5.217448223	-2.501343192	-4.291992274	-5.753067358	-3.437915139	-0.751906306	0.037412136	-4.152069052	-5.241710061	-4.184582533	-5.321471853	-3.627321368	-6.665859873	-5.188134347
-5.096198664	-2.522564261	-4.563473007	-5.706440515	-3.567541009	-0.722018446	0.001913017	-4.094073902	-5.46304698	-4.062289208	-5.427470523	-4.435224342	-5.969260799	-5.123203182
-4.702297675	-2.816713262	-4.340139256	-5.529740924	-3.402246718	-0.604081686	-0.295059223	-3.745939895	-5.251040443	-3.709009041	-4.922947406	-4.02414629	-6.089728117	-5.107292279
-4.815829972	-2.695745172	-4.36772571	-5.581062262	-3.542836826	-0.392987558	-0.267562587	-3.851123324	-4.874526679	-4.16768043	-4.062733955	-4.137492875	-5.759774749	-4.46019333
-4.77434721	-2.630336031	-4.458441285	-5.426590503	-3.318888178	-0.596044687	-0.3767485	-3.768960279	-5.119054373	-3.55453651	-5.250216322	-4.318601886	-5.924905302	-4.579217987
-5.048597753	-2.447781271	-4.386976346	-5.71876135	-3.591957325	-0.373839546	0.25802463	-4.032770351	-5.299183017	-4.049422147	-4.330717076	-4.350169774	-5.825356866	-4.728170505
-5.191810434	-2.428324715	-4.505416076	-6.045402996	-3.719222204	-0.492030155	0.002517493	-4.195261289	-5.304859766	-4.366594806	-4.213348525	-4.385529884	-6.265464981	-5.12236462
-5.136561918	-2.636602348	-4.222347106	-5.50458045	-3.606172639	-0.255717809	0.028755627	-3.894379467	-4.885025805	-4.392860786	-4.08993189	-4.154847068	-5.916235507	-4.626612975
-5.040336706	-2.615110008	-4.406181787	-5.553226388	-3.691938145	-0.343738156	0.527807248	-4.440115427	-5.431332013	-4.248837189	-5.764060091	-4.13570901	-5.951311543	-4.850031297
-5.607172736	-2.760480482	-4.675287948	-6.663337162	-4.133977661	-0.596306387	-0.219134131	-4.287498438	-5.426646019	-4.622439118	-5.775648269	-4.049655489	-7.251044393	-5.565667067
-5.559644598	-2.621864001	-4.826747224	-6.191757302	-4.154386938	-0.704819824	0.139909698	-4.621724819	-5.647880754	-4.487284303	-5.951682224	-4.049174499	-7.065643275	-5.535880108
-5.134443176	-2.864500945	-4.357411535	-5.584071953	-3.705451529	-0.5812703	-0.081291808	-4.44238224	-5.344209908	-4.249375679	-5.899527168	-4.240538117	-6.052720836	-4.798930895
-4.998961902	-3.186470235	-4.450214219	-5.898086475	-3.92467651	-0.316625611	-0.273197232	-4.539466204	-5.117210635	-4.511863361	-5.510639769	-4.234129783	-6.003821477	-4.754644065
-5.399800192	-2.580324625	-4.1814398	-5.973981173	-3.86338127	-0.415021737	0.097253077	-4.511447995	-5.229074874	-4.451706742	-5.563616692	-4.125480652	-6.431430383	-5.169596446
-5.187207539	-2.992141418	-4.337539947	-6.025736383	-3.969799595	-0.428142574	-0.381096013	-4.43921066	-5.040325207	-4.443891722	-4.581841524	-3.858506226	-6.005383509	-4.647627564
-5.212188645	-2.812289556	-4.345896162	-6.249708089	-4.265974779	-0.682168919	0.162099399	-4.769162841	-5.237774732	-4.393603822	-6.125264693	-4.187814299	-6.381304141	-4.743645008
-4.970764253	-3.073318526	-4.478777982	-5.579047937	-3.882153251	-0.514166522	-0.122294349	-4.385141753	-5.495991277	-4.273432867	-5.556557508	-4.159696851	-6.264463614	-4.863106054
-5.174841388	-2.664382737	-4.463646422	-6.038872131	-3.91845332	-0.453273423	-0.001501572	-4.375502022	-5.273547256	-4.494920719	-4.633596733	-4.006410976	-6.071391792	-4.623663827
-5.354405475	-3.23199101	-4.272112834	-5.76132584	-3.897473405	-0.351226117	-0.291972253	-4.445768779	-5.137163211	-4.624126078	-5.618165759	-3.851180124	-5.74691025	-4.764768732
-5.271088048	-3.261654161	-4.38611241	-5.885424964	-3.87995954	-0.251448068	-0.298071764	-4.419439238	-4.959128243	-4.613568658	-5.617446239	-4.165787754	-5.66399861	-4.53606636
-5.039399048	-2.645641633	-4.563164797	-5.841328899	-3.831652387	-0.605642328	0.184642552	-4.349724192	-5.516287819	-4.243845024	-5.844817588	-4.203255153	-6.314726599	-5.134936181
-4.897803491	-2.794800476	-4.569753321	-5.693739099	-3.627482271	-0.678250867	-0.285832433	-4.168646505	-5.420191164	-4.018627692	-5.638781564	-4.342218132	-6.30714447	-5.039932793
-5.328997425	-2.909903232	-4.516582219	-5.847728536	-3.876116529	-0.351223665	-0.133692871	-4.339849906	-5.293546916	-4.545124338	-5.660537984	-3.956029612	-5.233185186	-4.706006339
-4.590094979	-2.83631826	-4.535810613	-5.757819935	-3.578088534	-0.657275466	-0.44424249	-4.037105266	-5.260796413	-3.939216591	-4.906343929	-4.264047421	-5.995870809	-5.27560154
-5.857126131	-2.796633208	-4.793661525	-6.598012316	-3.848916905	-0.544540568	0.116037399	-4.45851555	-5.783208108	-4.763918728	-5.723721821	-4.702995643	-6.293151604	-5.242604231
-5.0913781	-3.181010567	-4.54574626	-6.16306767	-3.629156588	-0.790830312	-0.464731401	-4.292820799	-5.656374431	-4.203105209	-5.942725666	-4.699795554	-6.622655136	-5.075075109
-5.252661092	-3.285130124	-5.113926015	-5.97545151	-3.879249372	-0.338935913	-0.732426487	-4.192118841	-5.100117594	-4.304195283	-5.760181581	-4.389338462	-5.597501027	-5.041413018
-5.208463318	-2.618358111	-4.5068213	-5.669258639	-3.733199657	-0.659451946	0.189304546	-4.199415702	-5.421257312	-4.340762429	-5.652381035	-4.285934683	-5.731922738	-5.010823147
-5.164367874	-2.283752343	-4.480657491	-5.70801913	-3.734143752	-0.572225339	0.41306107	-4.316531449	-5.62623836	-4.3880433	-5.329866812	-3.75332177	-5.631818095	-5.134839093
-6.045606535	-3.686894425	-5.369120732	-6.315214396	-4.682673845	-0.939188606	-0.77319383	-5.155465387	-5.997865138	-4.914148212	-6.196960097	-5.266657512	-8.748746756	-5.404093775
-5.560629925	-2.634363864	-4.813537523	-5.74123367	-4.210393607	-0.382428194	0.106409776	-4.564369128	-5.004739305	-4.853972542	-5.169300709	-4.060283808	-5.089158478	-4.70490626
-5.597220993	-2.095169381	-4.520065271	-5.848767843	-4.103843357	0.093433493	0.969624168	-4.495357235	-5.027314436	-4.764018406	-5.180404955	-3.206130887	-6.365086426	-4.891205085
-5.451499471	-3.137457595	-4.774427321	-6.668089016	-4.35209578	-0.504063411	0.006732852	-4.872526843	-5.484068518	-4.662264477	-6.223921389	-4.35187049	-6.770742167	-5.563710753
-5.089055998	-2.915871267	-5.066447411	-5.851904628	-3.895422409	-0.011719802	-0.039741894	-4.066410741	-4.851779085	-4.241388483	-5.579585167	-3.708215982	-5.856119967	-4.448070865
-4.90707276	-3.063822752	-5.360142722	-5.751452215	-4.660403468	-0.258670269	0.097753971	-5.029068261	-5.048053627	-5.451630203	-6.256667936	-3.602319886	-6.972581245	-4.498413509
-5.066638609	-2.64510835	-4.690430489	-6.363049724	-4.198183573	-0.503547183	0.262423811	-4.497959366	-4.97241244	-4.273485987	-5.970894237	-3.966334982	-5.936797955	-4.745776072
-5.305779785	-2.762008787	-4.669340772	-6.417316341	-4.041210748	-0.592865723	0.2181129	-4.215409609	-5.367596204	-4.176085392	-6.184655147	-3.90581949	-7.002357969	-5.230250833
-4.976408105	-3.132182756	-4.960191768	-5.590211535	-4.469022933	-0.323663439	-0.350309711	-4.340548549	-4.833454849	-4.418324018	-5.615377569	-4.08050843	-4.918200712	-4.6792477
-5.257386519	-3.463350492	-5.033250873	-6.442861684	-4.135483914	-0.491342346	-0.494869062	-4.217266534	-5.109519004	-4.22737603	-5.696951097	-4.677994384	-5.726284505	-4.93512337
-5.577224106	-2.002829406	-4.474903531	-6.132097726	-4.148096968	-0.341980075	0.517132204	-4.260052127	-5.253178009	-4.650613391	-5.571199243	-3.953510518	-6.23354005	-4.50736584
-5.441176947	-1.255557048	-4.494635577	-6.44949083	-4.695174942	-0.656941104	0.818128485	-4.450957093	-5.412813123	-5.07644154	-5.890649141	-3.923185431	-7.058063995	-4.383493223
-3.988630496	-3.288664541	-4.591592367	-5.47492264	-3.465837333	-0.854712893	-0.710333672	-4.080481846	-5.338377924	-3.638877249	-5.347179901	-4.241895017	-6.444873761	-5.272953671
-5.797444527	-2.432418231	-4.7002163	-6.514576371	-4.459894173	-0.423078513	0.331304858	-4.587385393	-5.170604322	-4.794229025	-5.888299814	-3.99640651	-6.747154405	-5.038970388
-5.490420597	-2.562325761	-4.707504109	-6.963245465	-4.485410339	-0.505499672	0.004959037	-4.59368668	-5.172683881	-4.867840671	-5.883809288	-4.393785733	-6.625655004	-5.072513385
-5.097596417	-2.555326153	-4.549831476	-6.389956423	-3.91598029	-0.702636978	0.033052587	-4.431130943	-5.417785226	-4.200147064	-5.924666512	-4.352925494	-6.710574989	-5.144566924

-5.314045205	-2.209360274	-4.593529103	-6.213835987	-4.392796338	-0.535738256	0.425414823	-4.51420712	-5.317257984	-4.596170378	-5.632442048	-3.953798347	-6.858178489	-5.132466272
-5.664566452	-2.524766616	-4.5487843	-5.060404166	-4.367209709	-0.241676469	0.386382794	-4.463595667	-4.948178401	-4.581086209	-5.101133368	-3.733470058	-5.019591823	-4.301706163
-4.920643535	-3.010727812	-4.442280091	-5.877110967	-4.039132339	-0.158787446	0.013475285	-4.444545335	-4.687908908	-4.318853856	-5.314219023	-3.883749457	-5.80647996	-4.635773998
-4.25170932	-2.648968409	-4.554289254	-5.78600111	-3.775036984	-0.905160333	-0.061077139	-4.276572248	-5.586277899	-3.453365089	-5.466666075	-4.042880364	-7.262347835	-5.251298261
-4.704428174	-2.983876313	-4.780357131	-5.986303743	-3.936476686	-0.909041189	-0.207218087	-4.326403921	-5.460607528	-4.298111377	-5.691044957	-4.499325258	-6.595184073	-5.316027549
-5.351275381	-2.177951815	-4.415481131	-5.30168467	-4.386910281	-0.402357031	0.456604873	-4.732786408	-5.520659893	-4.706264713	-4.782223519	-3.839103182	-5.795301235	-3.897375243
-4.215812459	-2.624053921	-3.955478807	-4.853172659	-4.436862817	0.063628573	0.12865689	-4.083766867	-4.147974105	-4.480434589	-5.284180832	-3.936615788	-4.46711064	-3.403073026
-4.88689099	-3.378986029	-5.102586168	-5.742729063	-4.061747969	-0.710358925	-0.578234255	-4.456027639	-5.091034066	-4.43601635	-6.1131514	-4.480528012	-6.479027701	-5.218415162
-4.981975733	-2.207659912	-4.635902887	-5.718719966	-4.145447731	-0.248769254	0.598134478	-4.320056062	-5.041479871	-4.739085691	-5.8561497	-3.283954282	-5.8096971	-4.437785277
-5.294872656	-2.053705448	-4.461606426	-6.246992804	-4.237542896	-0.336943478	0.734908753	-4.47227543	-5.27014944	-4.588318381	-5.888103654	-3.485457481	-6.752754328	-4.730788588
-5.442927612	-2.58070285	-4.803145356	-5.887036626	-4.074689876	-0.684669349	0.268777607	-4.791428342	-5.192828615	-5.003280629	-5.926116302	-4.333360266	-6.060038725	-2.78046197
-5.671067571	-2.451505239	-4.676345665	-6.129530602	-3.91113945	-0.662330766	0.252627966	-4.396519403	-5.32352072	-4.827842595	-5.473496735	-4.133480021	-5.846110394	-4.188737953
-5.35089878	-3.158327499	-5.335797382	-6.856278149	-4.458315531	-0.527765264	-0.59279945	-4.720525174	-5.644968449	-4.526187513	-6.415979835	-4.739566144	-5.702610212	-5.290031558
-5.422931745	-2.289316554	-4.708796795	-6.296963435	-4.563800275	-0.649383466	0.306531521	-4.938657059	-5.634608657	-4.984097562	-5.757924475	-4.022828109	-4.769285007	-3.600741634
-4.614981863	-2.612615553	-4.740065518	-5.466744086	-4.609834905	-0.640133122	0.195678403	-5.177315133	-5.271245644	-5.125167842	-5.768565299	-3.997135869	-1.809213266	-3.681455716
-4.809620834	-2.800963628	-4.68368457	-5.65878777	-4.335857341	-0.192682245	0.03354543	-4.835723786	-5.346951347	-4.841227856	-5.688148296	-4.651806643	-3.612320223	-4.875510089
-5.105723543	-2.792092346	-5.3968928	-6.565270535	-4.346402409	-0.553632753	-0.012111459	-4.623404001	-4.945877403	-4.621330541	-6.000678709	-4.026716517	-5.830863068	-5.02076701
-4.842709444	-3.064734644	-5.064285729	-6.492316448	-3.605421775	-0.554182001	-0.624495577	-4.084269613	-5.31961714	-3.775187479	-5.663753581	-4.268551005	-6.339107351	-5.008349932
-5.246351837	-2.394096536	-4.668611145	-6.366254145	-4.171445878	-0.403618429	0.449847964	-4.228884286	-5.388885688	-4.332005934	-5.706993663	-3.820269344	-6.121851271	-5.074554164
-5.757412838	-2.299509982	-4.322153283	-6.616212485	-4.287641176	-0.557863983	0.296801794	-4.724768589	-5.385302669	-4.939301729	-5.738031878	-4.489121843	-5.919578718	-4.462490143
-5.365453163	-2.835630994	-4.389559138	-6.602824132	-4.183073903	-0.234278088	-0.170227619	-4.120659402	-4.929325928	-4.530813617	-4.275506762	-3.972045928	-6.109491321	-5.001121165
-6.307208055	-2.653975445	-3.988379148	-5.364556591	-4.146819684	-0.582447076	0.050472984	-4.619677123	-5.772131382	-5.166408999	-4.464403625	-4.530247933	-4.923112044	-4.328453733
-6.110791416	-2.404426801	-4.618392828	-6.834641597	-4.654244898	-0.701872479	0.101637252	-5.077891437	-6.155202194	-5.025845634	-6.248756242	-4.639437262	-8.200028961	-5.8669425
-8.233019745	-2.434627434	-4.800323266	-7.144088026	-4.81449251	-0.088569356	0.340706673	-5.364933041	-6.101126983	-6.268956317	-5.807414773	-4.293168882	-6.043845379	-5.068857251
-6.019204229	-2.998043398	-4.838022986	-6.64100928	-4.432359121	-0.53310817	0.093147772	-4.871673964	-5.919822434	-4.997713353	-5.698732777	-4.910498513	-7.819894108	-5.389196837
-5.102683756	-2.840796546	-4.134481027	-5.017593571	-4.014925483	-0.55242347	-0.231513387	-4.511854797	-5.136150806	-4.892564894	-5.205977556	-4.507138058	-5.80128662	-3.925013033
-5.580094443	-3.035000294	-3.521139449	-4.987491948	-4.030513665	-0.398740257	-0.579803611	-4.55712322	-5.547321873	-5.099592705	-4.244770361	-4.308498492	-6.136825372	-4.167654378
-5.781592585	-3.270232154	-3.871420254	-5.705042561	-4.106879883	-0.152836852	-0.75522223	-4.505776551	-5.277464461	-4.843151742	-4.531605113	-4.543568434	-6.192970976	-4.772737277
-5.65634189	-2.736068193	-4.021612036	-5.61489827	-3.991745482	-0.326430383	-0.18917869	-4.608107421	-5.675507184	-5.001912048	-4.607826928	-4.509897568	-6.412123976	-4.106178145
-5.310854117	-2.553798355	-4.12308848	-5.548127557	-3.985453504	-0.597388867	0.02217735	-4.507763614	-5.255657169	-4.865088753	-5.079765967	-4.177808007	-5.595728126	-4.312892977
-5.056912116	-2.474692317	-3.423706578	-5.279761855	-4.091762	-0.32917362	-0.144295599	-4.57711509	-5.666126698	-5.205619862	-4.303136543	-4.454949199	-6.116884331	-3.771715812
-5.086843216	-2.943727181	-3.735515675	-5.252391162	-4.153882956	-0.393200429	-0.467439017	-4.629052346	-4.909983166	-4.766658217	-5.16411719	-4.173123625	-6.536181873	-3.734135066
-5.994940937	-2.619640254	-3.534195792	-5.357321694	-3.69296539	-0.470693389	-0.166060432	-4.486324395	-5.927368647	-5.342773847	-4.288667254	-4.440357069	-4.677491434	-3.808452034
-5.482128931	-2.630925881	-3.492832559	-4.9624823	-4.061979448	-0.35026826	-0.148563558	-4.567486788	-5.554991147	-5.018113532	-4.193172426	-4.271189679	-5.172890994	-3.9713497
-5.795210204	-2.625382277	-4.341523987	-6.932113281	-4.232679917	-0.601342174	0.649501885	-4.600588519	-5.383356226	-4.542333752	-5.922904927	-4.224401371	-7.132094662	-5.39591519
-5.15482618	-2.416935015	-4.551141306	-6.040879887	-4.012739435	-0.327472213	0.443959624	-4.348692361	-4.876700959	-4.091953933	-5.727296816	-3.572729967	-5.599296592	-4.821537579
-5.149284777	-2.038740855	-4.619467973	-5.395876799	-4.323229743	-0.558463952	0.480135455	-4.10131217	-5.176904032	-4.596748854	-5.402913761	-3.835659828	-5.109488307	-4.100377905
-5.206360246	-2.733919504	-4.503523613	-5.365036278	-4.334529165	-0.737288834	0.318833412	-4.349013477	-5.140229491	-4.614320537	-5.966217806	-4.437572398	-4.890982836	-4.713876734
-5.415987054	-2.791191839	-5.044173936	-5.829512545	-4.345944464	-0.642998532	0.168500267	-4.425612345	-5.008236945	-4.734198091	-5.386020995	-4.123569979	-5.862522211	-4.943680374
-5.107202398	-3.071573314	-4.60976021	-4.790497838	-4.473655802	-0.58570116	-0.176922883	-4.593038043	-4.402955722	-4.565237844	-4.871136149	-4.085482434	-5.367461894	-4.705100961
-5.492644749	-2.491016229	-4.581754491	-5.821213355	-4.146285725	-0.626904636	0.506713904	-4.467481189	-5.29565478	-4.806337715	-5.759806968	-3.997740651	-5.938168628	-4.514941422
-5.460534391	-2.152394005	-4.574739553	-6.275849073	-4.282521929	-0.552115812	0.550893509	-4.684973791	-5.478019378	-4.841153581	-5.857908697	-3.890022506	-6.377491537	-4.599117853
-5.855226665	-1.922699118	-4.994055031	-6.947369674	-4.392831372	-0.70418267	0.719005007	-4.654507984	-5.518440826	-4.856356912	-5.799656488	-4.184857354	-5.391403908	-3.443391368
-5.367813462	-3.039127846	-5.415149619	-6.456757292	-4.481457929	-0.523088265	-0.35759958	-4.892669353	-5.423380024	-5.021112195	-6.240341724	-4.82429543	-4.774200274	-4.45954866
-5.872517649	-3.180736082	-5.160516708	-7.032086486	-4.613932531	-0.575366343	-0.163717322	-5.09732522	-5.554565958	-5.021876535	-6.140974975	-4.412739885	-5.023159659	-4.58558198
-5.553138393	-2.419500147	-5.126357082	-6.738309311	-4.733143135	-0.532349893	0.116997074	-4.68371224	-5.461354536	-4.954110676	-6.362970381	-4.557640156	-4.21158988	-4.448510888
-4.994156635	-3.141686818	-4.714412461	-5.25936314	-4.463011483	-0.129354335	0.128667095	-4.351307257	-4.716236494	-5.344414299	-6.276403394	-3.998501118	-3.479669274	-4.225540884
-5.503743914	-2.017706758	-4.800814112	-7.193708187	-4.472008562	-0.708342255	0.603160801	-4.472980365	-5.2144042	-4.412815147	-6.497199719	-3.771736486	-7.110408369	-4.296044444
-5.392053824	-2.411549844	-4.351952459	-6.184951251	-4.466066679	-0.37558658	0.301827188	-4.3393858	-5.192319547	-4.9346699	-5.14322802	-4.08477662	-4.820558037	-3.946500755

-5.453333256	-2.705182233	-3.421781432	-5.02334375	-3.711979242	-0.24860306	-0.307239753	-4.287090502	-5.280353418	-4.882208568	-3.987599084	-4.285454593	-5.756051945	-3.774370867
-5.772305254	-2.622201838	-3.271448396	-5.044768239	-3.908263355	-0.505697658	-0.109039987	-4.423420662	-5.963402575	-4.797413307	-3.963892085	-4.533632018	-5.70232606	-3.913664985
-5.397011187	-2.301631234	-3.544248173	-5.065434546	-3.99223127	-0.398449552	0.046893154	-4.277805172	-5.563925692	-5.056523861	-4.216341491	-4.311625084	-5.793680661	-3.633338198
-5.339570227	-2.567778393	-3.916687669	-5.743628797	-4.054192237	-0.348306757	-0.176402892	-4.440235107	-5.70067183	-4.712846785	-4.40392494	-4.561071165	-6.196676084	-3.880375149
-5.963745855	-2.723902396	-3.966887827	-5.479480286	-4.013545439	-0.601815256	-0.104764616	-4.54979012	-5.939045084	-5.093115122	-4.718083331	-4.768084867	-6.300719896	-4.545396605

hsa-miR-4792	hsa-miR-4800-3	hsa-miR-4800-5	hsa-miR-484	hsa-miR-486-3p	hsa-miR-486-5p	hsa-miR-498	hsa-miR-5001-5	hsa-miR-5008-5	hsa-miR-5010-5	hsa-miR-504-3p	hsa-miR-5088-5	hsa-miR-5090	hsa-miR-5100
-3.349254303	-4.281122903	-3.953635837	-3.002336251	-2.110098479	-2.752266096	-3.620308403	-2.60973972	-3.70201708	-4.507205389	-3.769698015	-3.960648086	-1.888328471	-1.263412536
-2.97713075	-2.694924529	-3.989050456	-2.1419911	-1.86951878	-1.220244692	-3.021419017	-1.151622951	-2.976766528	-4.210607231	-3.931809607	-4.242314059	-1.537927735	-1.27409292
-2.583301195	-2.433161965	-3.827500648	-2.661750124	-2.324722705	-2.753232961	-3.745120475	-0.931442642	-3.736623472	-3.815313331	-3.604056262	-4.338791678	-2.225770947	-0.474602445
-2.656476185	-3.386655597	-4.614085828	-2.689458264	-2.427929224	-2.761628587	-3.460659825	-1.125392563	-3.459247296	-4.173701005	-3.903055652	-4.341967893	-1.961444176	-0.606608946
-2.628024052	-3.473458412	-3.877047198	-2.749894798	-2.346350386	-2.495703874	-3.619569194	-1.985109275	-3.79492377	-4.283730908	-3.945507031	-4.201864207	-2.045393201	-1.096897153
-3.136767464	-3.326420025	-4.184295217	-2.087609257	-2.386043177	-1.498377598	-3.386557282	-1.309850781	-3.628741378	-4.251788079	-3.766713935	-4.252382436	-1.807889813	-0.396308314
-2.677615837	-2.723196846	-4.077355781	-2.061739326	-2.35469609	-2.242623263	-3.568252721	-1.11685566	-3.476303443	-3.936851235	-3.855049144	-4.327326168	-1.967481948	0.010825026
-2.825244012	-3.071607141	-4.417618202	-2.106784263	-2.379486397	-2.24461855	-3.103038459	-0.833328827	-3.770974032	-3.970378834	-3.729571031	-4.189030414	-1.850964694	-0.37126007
-2.818228336	-2.12573305	-4.191306117	-2.570253004	-2.725640106	-2.340177983	-3.584690517	-0.918081875	-3.925743153	-4.070220279	-3.834901888	-4.14697686	-2.131999443	-0.616320807
-3.166114208	-3.583615969	-4.334367589	-2.514336397	-2.028807777	-1.788093832	-2.733294741	-1.516688271	-3.796532778	-4.025090573	-3.86984812	-4.005620831	-1.700467662	-0.57076034
-2.988997661	-2.835946849	-4.600799941	-1.957559782	-2.45667469	-2.241035606	-2.713252052	-1.477355024	-3.868122916	-4.010756888	-4.067949102	-4.220897681	-1.931919363	0.345467759
-3.119131897	-2.635793103	-4.05233868	-3.424378363	-2.09954959	-2.407533853	-3.638032772	-1.498739982	-3.943707093	-4.134843563	-4.069319416	-4.359094796	-1.75377426	-1.699974041
-3.01383114	-3.081354287	-4.191979421	-3.351613202	-2.332079379	-2.886008386	-3.647653307	-1.166366134	-4.166145212	-4.034290133	-3.95393001	-4.563399389	-2.049401853	-0.876399591
-3.197511323	-3.733008916	-4.745069188	-3.054323108	-2.223152399	-2.932127144	-3.031920008	-1.68231961	-4.04985451	-4.093357941	-4.200772685	-4.355043168	-1.971980368	-0.199749976
-3.166801336	-3.544066639	-4.410107463	-2.468432593	-1.785536542	-0.748636311	-2.577275537	-1.892773691	-3.865328255	-4.4328862	-4.361489576	-4.342631364	-1.617306821	-0.493069873
-2.849227975	-2.339148814	-4.193178604	-3.233520733	-2.258804644	-3.057847498	-3.035413926	-1.116265003	-3.861163949	-3.934514402	-3.951722396	-4.257418346	-1.946948532	-0.586690157
-3.120783276	-3.00766774	-4.323241287	-2.747530395	-1.776476163	-0.274400553	-3.129205585	-1.454877751	-3.693708891	-4.134065256	-3.987433308	-4.319767471	-1.819435188	-0.707486777
-3.167482665	-1.475420966	-4.133325476	-2.412332258	-2.41474635	-2.603166543	-3.250013059	-0.694199161	-4.244801121	-4.178620387	-4.084101771	-4.45850225	-1.759101672	-0.902763026
-3.079859148	-3.804614296	-4.03704422	-2.877631576	-2.249778364	-2.103871523	-3.290440164	-2.015613698	-3.980183958	-4.431237978	-4.168108092	-4.288013328	-1.871160503	-0.604260633
-2.772352992	-2.769215415	-3.994044681	-2.402255302	-2.171195155	-2.029599396	-3.013461988	-1.249156879	-3.813693944	-4.119685857	-4.098254632	-4.31972888	-1.817618758	-0.340678337
-3.133914718	-3.131350077	-4.561900748	-2.402525159	-1.69465308	-2.084419509	-2.878686369	-1.543581841	-3.87603295	-4.138594613	-4.117760985	-4.40888101	-1.845756653	-0.444726833
-3.046580934	-2.283815981	-4.205344568	-2.840282374	-1.811496026	-2.284730426	-3.016660037	-1.195724056	-3.782778821	-4.39838273	-4.346943515	-4.402129288	-1.656305993	-0.187380895
-2.868630514	-2.982587019	-4.174716144	-2.732553321	-2.617730019	-2.796692132	-3.62104447	-0.727728852	-3.961942513	-4.022597021	-4.115232636	-4.436786734	-1.995130642	-0.382120836
-2.900024905	-2.530308823	-3.715799761	-3.378885574	-2.005060722	-3.442732341	-3.83307512	-1.16103121	-3.602141921	-4.04873988	-4.023377675	-4.255230002	-1.810667001	-1.065291644
-3.095082702	-2.770125507	-4.441998442	-2.940981426	-2.024082631	-1.916554555	-3.210183567	-1.311818131	-3.94640612	-4.144409789	-4.25065475	-4.492466199	-1.658547535	-1.07464904
-2.77021934	-3.007194094	-3.876452253	-3.352657308	-1.90277177	-3.336360326	-3.831074983	-1.40056083	-3.566039279	-4.165362552	-3.933711891	-4.341857111	-2.074704601	-1.106774058
-3.384207187	-3.146453845	-4.702303424	-2.867710706	-2.385679206	-2.963994994	-3.130082218	-1.644327112	-4.139496211	-4.192220059	-3.984885486	-4.668940772	-1.716187362	-0.736041046
-2.9597552	-3.253202906	-4.121798177	-3.431521088	-2.124513576	-2.994220983	-3.748265681	-2.004987214	-4.036285832	-4.269489768	-4.135134742	-4.546305597	-1.841545611	-0.825328766
-3.495522571	-3.45438629	-4.734057751	-2.71674615	-1.521533876	-1.339185445	-3.804752984	-2.070832224	-3.875729133	-4.547243347	-4.078613461	-4.399934393	-1.85137798	-1.501046964
-2.794613327	-3.348311686	-4.460314381	-2.917761112	-2.547049267	-2.893691846	-3.53757298	-1.032499992	-4.008088638	-4.004696861	-3.860870998	-4.270617701	-2.052324902	-0.657328228
-2.666912976	-3.09010842	-4.529521436	-2.553523311	-2.62098233	-2.611958468	-3.2750169	-0.798821139	-3.905421754	-3.903282634	-4.065927901	-4.217145932	-1.934857683	-0.561901484
-4.689967044	-6.239063778	-5.268924489	-4.017554418	-1.306461311	-2.861031053	-4.294323609	-3.090493274	-4.254003238	-5.02713205	-4.48095279	-4.951030953	-1.045752239	-1.782530821
-3.565323451	-2.055005252	-4.986673203	-4.069966963	-1.898987487	-3.327171842	-2.561105672	-1.239336011	-4.144075997	-3.882155528	-3.91980736	-4.03261831	-1.245509817	-1.467598743
-3.502748434	-4.10017145	-5.036512385	-4.109139756	-2.279914077	-3.109431057	-2.570938986	-0.872771415	-3.878687835	-3.702825478	-3.791580543	-4.115393286	-1.369372075	-0.749064409
-3.525496938	-3.431639976	-5.337465825	-4.547212207	-2.324378558	-4.299430413	-3.338395293	-1.773017712	-4.205995947	-3.883506137	-4.241253828	-4.44750576	-1.821829004	-0.626728823
-3.156384127	-3.776575878	-4.447344072	-3.593769608	-2.066996537	-2.088404127	-3.331386206	-1.334690915	-3.826108579	-4.082782585	-4.226334762	-4.38778438	-1.582728843	-2.01972937
-4.163966338	-3.084864312	-6.151358516	-3.144880711	-1.223456505	-2.412387471	-2.312177349	-1.443727803	-4.849686562	-5.307010064	-4.487005692	-5.13482323	-1.613301465	-2.041863315
-3.344517741	-2.744731343	-4.932867691	-3.735412814	-2.234429598	-3.240993184	-2.628864404	-1.044660098	-4.342279193	-3.881197317	-4.142979107	-4.290524336	-1.527887506	-1.294098843
-2.682947916	-2.976022448	-4.914720242	-3.948360318	-2.356108273	-3.925580131	-3.353706819	-1.395358339	-4.30049986	-3.871612371	-4.127003862	-4.444432049	-1.803552397	-1.164186984
-4.125971999	-3.913605236	-5.154006531	-3.71443218	-1.446119204	-2.357225741	-2.664929826	-1.998182796	-4.275254292	-4.260153611	-4.273754644	-4.603230488	-1.3413413	-2.155246971
-3.435668087	-2.859770947	-4.799216677	-4.143147134	-1.627798991	-3.719299775	-3.308274645	-2.115930368	-4.420179478	-4.176853881	-4.296504215	-4.473015875	-1.510795125	-1.74972978
-3.203790247	-2.810378272	-5.34007043	-3.40911757	-2.265828727	-3.023454042	-3.236595376	-0.460273504	-4.595680111	-3.891078138	-3.821413679	-4.468170961	-1.427968866	-1.052721363
-3.182275552	-2.575731293	-4.748606519	-3.930912136	-1.849666964	-3.684541764	-3.648798255	-0.445956957	-5.316704099	-4.207851771	-3.876605666	-4.843151396	-1.958265478	-2.194960388
-3.168879213	-5.071613121	-3.847160102	-3.708748054	-1.657041399	-3.970210086	-3.604825575	-3.145585386	-4.545142599	-3.424293965	-3.662779545	-3.995833192	-2.058336556	-1.605932738
-3.215888322	-2.442934329	-5.281680661	-3.963114165	-2.338015614	-3.538136643	-3.13789996	-1.135054388	-4.298587869	-4.086695635	-3.954800558	-4.495555406	-2.089606035	-1.317053426
-3.121965731	-2.121104479	-5.126509855	-3.910981345	-2.051788447	-3.323053448	-2.917701904	-1.090596165	-2.787515247	-3.703744527	-4.024963209	-4.475851944	-1.717260489	-1.461876511
-2.107910856	-2.281424595	-3.836319011	-3.713685512	-2.43176779	-3.908822245	-3.686034994	-0.762663524	-4.021538175	-3.576985139	-3.733985796	-4.454095214	-1.93483806	-0.538408218

-2.819263263	-2.248675779	-4.880846368	-3.494528786	-2.406235658	-3.246230353	-2.820575963	-1.036518817	-4.430356789	-4.032155067	-4.03125134	-4.351639127	-1.556715374	-1.207475098
-3.876026275	-1.070872658	-5.041471594	-3.359845768	-0.947222001	-1.687536361	-2.697635851	-0.602665565	-3.965841905	-3.920832486	-3.925751117	-4.449414383	-1.307312622	-1.773884794
-3.623066065	-3.447489833	-4.618699649	-3.915349286	-1.682811038	-3.102573255	-2.358083964	-1.782887857	-3.685108413	-3.931209468	-4.018828621	-3.910733711	-1.075220881	-1.202781275
-2.454175633	-4.509756278	-3.671373971	-4.181417772	-2.35919901	-4.342463666	-3.855597481	-1.703581952	-4.265092889	-3.690595424	-3.643995025	-3.997966922	-1.645580556	-0.823240478
-3.065810764	-3.720749703	-4.771833229	-4.219745151	-1.711960209	-4.016464819	-3.961921678	-1.723494576	-4.495414041	-4.384240697	-4.104612277	-4.710517067	-1.859568983	-2.024696368
-3.938670894	-3.236742154	-4.808157996	-4.285591187	-1.787588795	-2.16795299	-2.635147192	-1.200753359	-4.66969242	-4.220497438	-3.842081639	-4.542770814	-1.109051007	-1.89554534
-3.136317535	-2.970249846	-4.198347962	-3.618384847	-1.055930095	-2.546948599	-2.673537952	-1.475585497	-3.949334524	-4.799226775	-3.839401464	-4.468704831	-1.112293367	-2.465742444
-3.677133479	-2.845140275	-5.178347746	-3.396133465	-0.964938617	-3.207616551	-3.629501336	-1.578147168	-4.316209919	-4.413632952	-4.068262496	-4.84744522	-1.690211238	-2.087211172
-3.46003901	-1.78074281	-4.938123341	-3.699506836	-2.090782409	-2.991496128	-2.711399383	-0.698306079	-4.425384581	-3.776976205	-3.638058828	-4.191689573	-1.536401541	-1.350330525
-2.951318615	-1.627215824	-4.359172895	-4.004756534	-2.252723877	-4.144791154	-3.347669375	-0.406775015	-4.304775353	-3.578974467	-3.753709167	-4.186558694	-1.694011354	-1.14818761
-3.425023082	-3.406227951	-5.201360982	-2.553682056	-1.436492084	-2.228580573	-3.19606966	-0.629253898	-4.740743541	-0.738846235	-3.860485542	-4.682907281	-1.223921086	-0.029871071
-2.86556582	-2.407809886	-5.202589777	-3.52933683	-1.680410268	-3.30243426	-2.957871987	-0.813417572	-4.266540324	-3.631939446	-3.705290615	-4.46273518	-1.544555791	-0.088475321
-3.896187807	-3.820944309	-5.057982484	-4.421761037	-1.739604234	-3.816290506	-3.772435494	-2.279862932	-4.70741623	-4.472535284	-4.215585328	-4.641807238	-1.643703943	-2.712031647
-4.526710885	-2.519846986	-5.294302851	-4.260484758	-1.710833851	-3.78542122	-2.884108979	-1.044860297	-4.859827927	-3.767077747	-3.761801673	-4.571024338	-1.548581153	-0.533844593
-4.056035473	-1.948663878	-6.169241239	-4.52134783	-1.627212109	-3.161004453	-2.701060065	-1.224929026	-4.992694382	-4.626086052	-4.238563574	-4.865680015	-1.411943632	-2.086308956
-3.963002578	-1.9769401	-5.330620466	-3.178910202	-1.60704789	-2.636388911	-2.482357763	-1.38458147	-4.189423037	-4.144833371	-4.077663773	-4.57067628	-1.249637769	-2.041509511
-2.983877253	-2.077530512	-5.074955568	-2.790542223	-2.053695245	-2.168500736	-2.8261203	-1.218081136	-4.444232567	-3.68281271	-4.315131479	-5.047711438	-1.829878928	-2.08752365
-3.799960084	-4.005970648	-4.732398656	-4.269393255	-1.749826748	-4.111035547	-3.597395103	-2.171672625	-4.741798124	-4.263039501	-3.833142043	-4.260888078	-1.681294763	-2.020249322
-2.950946063	-1.932021646	-3.872688143	-4.306503255	-2.01599202	-4.199595081	-3.524604639	-0.441186938	-4.259126242	-3.489149983	-3.639424081	-4.286635825	-1.889509404	-1.304249502
-3.385931218	-2.465980122	-5.076803171	-3.371802056	-2.493612381	-3.110050662	-3.362705976	-0.482460242	-4.758914101	-3.883811989	-4.062665626	-4.539450704	-1.957944357	-1.085072555
-3.263331535	-2.719371317	-4.032968544	-3.601363842	-2.107103819	-2.932813286	-3.569327168	-1.013532025	-3.923738352	-3.797211302	-3.975906806	-4.264829603	-1.879591385	-1.419079642
-3.245739706	-3.195216582	-4.304535036	-2.734893926	-1.782074464	-1.861016858	-2.819270056	-1.518733861	-4.41202114	-3.938550489	-3.920247825	-4.252285284	-1.851698828	-0.014461256
-2.69194255	-4.215148265	-4.721104725	-4.175962488	-2.737634464	-3.974016263	-3.314388485	-1.721247382	-4.468454836	-3.941448822	-4.413960826	-4.651164329	-1.760502531	-2.100787212
-3.497125995	-2.020604977	-5.049886127	-3.9730294	-1.534722344	-3.181809896	-2.25998276	-1.215091941	-4.284566961	-3.896313594	-4.341924329	-4.545278624	-1.4748755	-1.701414774
-3.188290575	-3.530835135	-4.829503163	-3.446207373	-2.040175586	-2.575147739	-2.704494353	-1.782727632	-4.32530161	-3.704555948	-4.050971229	-4.528921782	-1.448497586	-1.742404097
-3.086643242	-2.878265442	-4.453770912	-2.908667725	-2.125249092	-2.187374147	-2.418842235	-1.265995341	-4.541125131	-3.837916024	-3.988960537	-5.000051547	-1.870421429	0.147456012
-3.985967967	-4.684608042	-3.69506017	-3.630364889	-1.195237903	-1.568617772	-3.062804241	-1.902740736	-4.074220683	-3.809320859	-3.803474794	-4.456705538	-1.628355922	-0.81760503
-4.206273057	-4.564361112	-3.981783535	-3.938845227	-1.392630408	-3.308958481	-3.300112176	-2.091568586	-4.183778836	-4.028058863	-4.212056213	-4.500136766	-1.511996058	-1.637179123
-3.344931735	-3.877044067	-4.248416241	-2.743953667	-1.724914901	-2.118637964	-3.018876515	-1.70092655	-3.707769473	-3.853987817	-3.988738169	-4.28959341	-1.682231427	-0.388644835
-3.01691991	-2.95210577	-4.250868624	-2.164663348	-2.639081291	-1.512180697	-2.797577861	-0.90940579	-4.397292765	-3.886399692	-4.13290513	-4.567956963	-1.990290205	0.201667563
-3.287547469	-3.303335081	-3.672396161	-3.109066586	-1.676451902	-1.150773834	-2.916933071	-1.094770951	-4.247637699	-3.638034045	-3.819929188	-4.360708679	-1.898235601	-0.360485403
-3.483111318	-3.980292332	-3.948358917	-2.8667559	-1.656822647	0.169089822	-3.26589381	-1.586528792	-3.971761182	-4.342995158	-4.13901314	-4.388216266	-1.549687274	-0.480079349
-3.628000926	-2.629030818	-4.001967966	-2.412156263	-1.749448816	-1.34253078	-2.794067453	-1.325075426	-4.712060696	-3.712839099	-3.653198901	-4.106304867	-1.854242449	0.859067054
-3.584967393	-4.084695538	-3.687272386	-2.932450035	-1.583718472	-1.724652363	-2.558549066	-1.457872493	-4.177112087	-3.479938151	-3.878171856	-3.980621882	-1.809515764	-0.384432818
-3.378344435	-3.44558613	-5.082903787	-3.959559448	-2.318095605	-2.163918245	-3.173462277	-1.18753116	-4.090927865	-3.791710072	-3.852601053	-4.387635159	-1.837336653	-0.732883367
-3.345891798	-2.670676024	-5.179756744	-4.277540415	-2.18263571	-3.384211826	-2.825627061	-0.848354714	-3.910344981	-3.690549061	-4.037252584	-4.245505924	-1.538607866	-1.061755
-3.137985019	-2.577887748	-5.15358814	-3.193761656	-1.919778593	-2.919541205	-3.062085904	-0.615437506	-4.596857787	-3.776951421	-3.988444267	-4.389163388	-1.200458886	-1.631105795
-3.292659258	-1.71863249	-5.096102151	-3.252177841	-2.320541394	-2.879129804	-2.708540577	-1.022656285	-4.255541208	-3.906063551	-4.011959654	-4.846677953	-1.91029298	-1.202888057
-4.012569643	-2.365048314	-5.11499668	-4.408832567	-2.302793706	-4.202363857	-2.602481096	-1.185737452	-4.238206803	-3.523524368	-4.282050556	-4.464165885	-1.894284911	-1.189925029
-4.371086986	-3.664728454	-5.17822155	-3.630051645	-0.815452771	-0.883909869	-2.770872553	-1.747621342	-3.798450217	-4.849240467	-4.139823399	-4.678365169	-1.516226751	-2.80054885
-3.395924624	-2.373662202	-5.362066737	-3.124168264	-2.16121167	-2.264617695	-2.596227343	-0.611931396	-4.398002132	-3.659952963	-3.874614052	-4.532648521	-1.516774362	-0.755197325
-2.939492091	-1.237989953	-5.195597722	-3.716867476	-2.096481488	-3.476050435	-3.27378333	-0.705974825	-3.595677041	-3.882165851	-3.854527685	-4.334975689	-1.353860612	-0.941332776
-3.573449001	-3.406746705	-4.450585273	-4.152024122	-1.621167808	-4.276596068	-3.979666043	-0.608494739	-5.257518881	-3.584705197	-3.10831032	-4.232353189	-1.760205583	-0.632925517
-5.040312611	-4.151986661	-6.191493197	-4.435739658	-1.53739352	-2.741900565	-3.569427986	-1.755441418	-4.814282744	-5.201486416	-4.315552113	-4.804109811	-1.219100668	-2.07643354
-4.28690378	-2.943683139	-5.15815117	-4.933840999	-1.404911037	-3.454558213	-3.586111004	-1.826389048	-4.584155211	-4.448190976	-4.579791722	-4.679762175	-1.162165898	-1.879730862
-2.670542441	-1.790846261	-5.810666192	-3.301784295	-2.005997134	-3.195140481	-3.173324798	-0.704687523	-5.193546889	-4.034930215	-3.981684809	-5.05418115	-1.620551341	-2.860815579
-4.221940223	-3.318334279	-4.975076533	-3.892601179	-1.638140191	-2.733472958	-2.144330523	-1.512284772	-4.120386624	-4.60153255	-4.45898756	-4.982718032	-1.407140509	-1.749699021
-3.289279579	-2.205687651	-5.431300296	-3.966212422	-1.682328363	-3.267169104	-4.109807704	-0.389274597	-4.736943271	-3.451223043	-3.919202598	-4.416786922	-1.820958143	-1.615260058
-4.036557236	-2.62623939	-4.916619083	-3.231316852	-1.721738771	-1.975078628	-2.820464346	-0.608563675	-3.929673236	-3.73740717	-3.953731917	-4.584937342	-1.71376917	-1.309029098

-3.341946168	-3.007761715	-3.770756364	-2.623816448	-1.511509443	-1.6979526	-2.698355822	-1.281227298	-4.021080634	-3.616855937	-3.732252224	-4.137964967	-1.579104737	0.018823085
-3.375618918	-3.333065183	-3.661887624	-2.431697274	-1.455013188	-2.029019345	-2.571432991	-1.36618413	-4.369127916	-3.297389363	-3.51665533	-4.077328466	-1.765986353	-0.150847626
-3.064275881	-2.785353214	-3.908544678	-2.429860697	-1.877323408	-1.510037107	-2.917612348	-0.841390284	-4.532055896	-3.576156247	-3.85905333	-4.301128845	-1.653182839	0.076625445
-3.091049356	-3.304433713	-4.006313009	-2.518580853	-1.505631515	-1.305946475	-2.773475478	-1.189898544	-4.466198388	-3.783286533	-3.989897924	-4.205964444	-1.785296005	-0.373382507
-3.096712625	-3.675496845	-4.365143953	-2.561900279	-2.0888053	-2.129894467	-2.997448969	-1.523289309	-4.571729436	-3.949938753	-4.048439445	-4.460225267	-1.864802758	-0.178146097

hsa-miR-5189-5	hsa-miR-5195-3	hsa-miR-5196-3	hsa-miR-5196-5	hsa-miR-532-3p	hsa-miR-548q	hsa-miR-550a-5	hsa-miR-551b-5	hsa-miR-557	hsa-miR-5572	hsa-miR-5587-3	hsa-miR-564	hsa-miR-572	hsa-miR-5739
-4.826013298	-2.068779812	-4.329569807	-2.11172946	-4.251301889	-3.726471461	-1.357902037	-7.641504061	-1.414132202	-2.388548711	-5.841397706	-4.377417854	-5.006819948	-2.957775138
-4.795549333	-2.640449196	-4.634091863	-2.597732273	-4.265019596	-4.057023346	-2.708187465	-6.373662434	-1.70763537	-2.27815882	-4.763694555	-4.198760251	-3.959297617	-3.020477114
-4.687381269	-2.826059213	-4.508402689	-2.412698093	-4.585620368	-3.806598352	-2.003963385	-7.264728836	-1.490730422	-1.953147148	-5.447968307	-3.780201147	-4.234769574	-3.069393802
-4.992210878	-2.448409406	-4.449884109	-2.406711568	-4.317412772	-4.215610627	-2.214979512	-7.362004774	-1.619392549	-2.017689621	-5.386272035	-3.972021217	-3.974417437	-2.868032765
-4.996919214	-2.278317842	-3.967903106	-2.068259325	-4.276360991	-3.726305254	-1.656992259	-6.587152611	-1.259094441	-2.148694629	-5.421153783	-4.263255646	-4.232496794	-2.966766234
-4.838421745	-2.549860236	-4.401584022	-2.423583678	-4.527135973	-3.682978505	-2.353354358	-5.901551257	-1.57767578	-2.148906429	-4.783782218	-3.828301892	-3.895209119	-3.123100803
-4.934965648	-2.596793436	-4.239146453	-2.238327214	-4.272349278	-3.937774357	-2.265044567	-6.885777825	-1.463494299	-2.607480498	-4.983056393	-3.957632455	-4.443693995	-2.932908007
-4.804920788	-2.884165098	-4.744030309	-2.719506645	-4.472993276	-3.789485218	-3.056874198	-6.252499402	-1.639311847	-2.4030707	-4.776060653	-3.691549504	-4.353416888	-3.041663668
-4.546412267	-2.760845159	-4.581813814	-2.435807573	-4.676949188	-3.846249812	-2.513284792	-6.894320879	-1.425098941	-2.64534616	-5.052436054	-3.830426349	-3.269873053	-3.133513146
-4.500169639	-2.326418223	-4.378915022	-2.634476121	-4.365078319	-3.56834626	-2.669869198	-6.001008477	-1.670294035	-2.63162944	-4.727592803	-3.901277933	-4.653148101	-2.94732434
-5.492742234	-2.760049497	-4.788194421	-2.702925617	-4.571711058	-3.878071936	-3.139325267	-6.697855804	-1.620790714	-2.71140714	-5.110379201	-4.178823035	-3.796479078	-2.946803321
-5.259335378	-2.649939818	-4.953137796	-2.476970205	-4.61729951	-3.588540047	-1.939007688	-8.209272199	-1.771917989	-2.747438787	-5.331875538	-4.555283142	-4.754658204	-3.002899994
-4.825992349	-3.096304044	-5.021647529	-2.615080913	-4.792019505	-3.851880813	-2.222639796	-7.381875267	-1.897008445	-2.816274556	-5.733689005	-4.053687706	-4.422351851	-2.969262171
-5.213224726	-2.782318223	-4.955216683	-2.744491327	-4.933522679	-3.605188482	-2.618404603	-7.372660092	-1.668511727	-2.45704199	-5.145977217	-4.404108535	-4.550507735	-3.017052952
-5.530541441	-2.818052312	-4.922479505	-2.688721001	-4.597366836	-3.567294186	-2.81879793	-6.362469499	-1.331412851	-2.885057227	-4.728310832	-4.708924503	-4.024839271	-3.097550384
-4.989709917	-2.897090936	-4.873146936	-2.653716061	-4.66712325	-3.875347476	-2.657316967	-6.945707395	-1.438126129	-2.213703429	-5.10296369	-3.980163093	-4.03825987	-3.063627948
-5.004884713	-2.959532936	-5.039233258	-2.460172195	-4.689821486	-3.64391463	-2.537159831	-6.261084869	-1.655122759	-2.631433931	-4.244229736	-4.145666552	-3.983068384	-2.991416734
-5.232147493	-3.242847119	-5.328129194	-3.0113886	-4.724705637	-3.740774706	-2.864167413	-7.292673063	-1.879745984	-2.752636157	-4.97144359	-4.262593482	-4.712276969	-3.116868054
-5.314667147	-2.37244065	-4.752647504	-2.538442892	-4.588571565	-3.852084956	-2.300060757	-6.786298796	-1.648256405	-2.598006754	-5.242414006	-4.40687399	-4.222848607	-2.85330437
-5.161976388	-2.673607838	-4.8834429	-2.638453066	-4.64206004	-3.882061463	-2.706665344	-6.745116963	-1.70199472	-2.845741833	-4.729270367	-3.896760753	-3.899630818	-3.008431661
-5.46132336	-2.95366714	-5.011013276	-2.769687826	-4.582406274	-3.456549648	-2.534491459	-6.519598562	-1.580973726	-2.608593214	-4.877782166	-4.491413894	-4.404402523	-3.157129478
-5.122834574	-2.914921762	-4.994755407	-2.739764248	-4.568187046	-3.425249902	-2.399369246	-6.528566466	-1.767033789	-2.934362488	-4.753072241	-4.266357407	-3.266308736	-3.013199137
-5.08877157	-2.639854886	-4.643834098	-2.617684853	-4.555629295	-3.787557458	-2.047297392	-7.346422699	-1.674135834	-2.429108286	-5.184145455	-4.171794851	-3.71439864	-3.062849381
-5.345605331	-1.62563992	-4.457070983	-2.014515157	-4.466931328	-3.832667147	-1.260158252	-6.92873988	-1.442030549	-2.491638763	-5.352380375	-4.276439614	-4.286346885	-2.70849836
-5.078035294	-3.166493502	-5.025859389	-2.775808881	-4.574063557	-3.742354339	-2.453939398	-5.989062762	-1.787064695	-2.319764268	-4.448250866	-4.45473011	-3.698087425	-3.003662588
-5.319458207	-1.961719578	-4.481425516	-2.127192909	-4.608325473	-3.708431264	-0.524796198	-7.43951824	-1.548590772	-2.443474623	-5.447201915	-3.846865937	-4.390261396	-3.125273516
-5.133929606	-3.02815863	-4.888601144	-2.491455168	-4.776380292	-3.763057653	-2.428774707	-7.553547186	-1.841613811	-2.828554444	-5.295313144	-4.397135414	-4.207487691	-3.098882125
-5.490325448	-2.463210549	-4.680959846	-2.273806189	-4.438005787	-3.628227328	-1.475560545	-8.79698194	-1.715625057	-2.86880011	-5.601388814	-4.306854962	-4.987095176	-2.94395537
-5.075661588	-2.779688139	-4.806097654	-2.842106789	-4.867116442	-3.769822775	-2.452727973	-7.55105991	-1.459797921	-2.49445442	-4.918056615	-4.456985203	-4.787738818	-2.953188316
-5.132807245	-3.371371693	-4.616323966	-2.61595505	-4.799612888	-3.832927373	-2.542623764	-6.779534134	-1.479335542	-2.635625955	-5.12993932	-4.115328846	-4.180152831	-2.911662401
-4.863100457	-2.647467046	-4.706521689	-2.627885234	-4.667709808	-3.966896761	-2.871623979	-6.581202508	-1.605796734	-3.019748479	-5.144900465	-3.918166125	-3.917785029	-2.867136097
-6.073534095	-2.256536033	-5.405183387	-2.745115932	-5.354521135	-4.564989373	-4.441214277	-7.598546235	-2.465704285	-2.909263542	-7.720460789	-5.205892857	-7.014678264	-3.217700639
-4.616583741	-3.572875139	-4.943009524	-3.402410198	-4.439410951	-3.616567081	-4.485333329	-5.473733212	-2.023099386	-2.410456144	-4.944106519	-4.519830186	-4.423785281	-2.646610887
-4.493702658	-3.459725936	-4.629216126	-3.430301878	-4.710457895	-3.341052905	-4.635686973	-6.022330131	-1.568846153	-2.837865716	-5.69041852	-3.582700201	-2.734018546	-2.848524138
-5.101670802	-3.307722681	-5.239702045	-3.29981936	-4.858339679	-4.08344605	-4.187715503	-6.652273453	-1.530134403	-2.649671401	-4.80015295	-4.632793683	-4.227295571	-2.935542927
-4.918990006	-3.508093722	-4.382182918	-3.0112706	-4.452193106	-3.579762735	-4.424563222	-6.964663645	-1.431574959	-2.07441872	-4.686516826	-4.515267775	-3.496973886	-3.05884312
-5.798450215	-3.772756958	-5.844412568	-3.721665272	-5.261724032	-3.935536827	-6.017593212	-6.73777681	-2.040739975	-2.882675132	-4.904722655	-4.75496502	-3.378825932	-3.243621981
-5.021727949	-3.28977504	-4.933538403	-3.505258286	-4.28696	-3.730712447	-4.326929412	-6.533598253	-1.593575563	-2.436981994	-4.931801981	-3.776142149	-3.263899267	-3.027989237
-5.266448218	-3.250931726	-4.711153958	-3.162332163	-4.405879778	-3.918362961	-4.274904213	-7.621992023	-1.260738516	-2.229929861	-4.794802521	-4.404247287	-3.24201639	-3.132778647
-5.486743829	-3.345695783	-5.084109934	-3.530232433	-4.122934771	-3.579348311	-4.884543154	-5.838542919	-2.18636145	-2.487597818	-3.889183922	-4.622323244	-4.109000977	-3.189402704
-5.462612376	-3.030739806	-4.672154365	-2.936457325	-4.487087109	-3.526139586	-4.0899214	-7.030326143	-1.490069426	-2.787284437	-4.712097071	-5.213986749	-5.176945736	-3.044896907
-4.490177847	-3.790614266	-4.949349242	-3.706886456	-4.538047445	-4.246910356	-5.202127964	-6.930480311	-1.989995716	-2.540076078	-4.435583348	-3.725676634	-3.846044208	-2.938224197
-4.692946062	-4.321698184	-5.527558558	-2.860574025	-4.890089677	-4.543585288	-5.376458611	-7.063599277	-2.625744127	-2.856638197	-4.555851981	-3.637532609	-3.918502951	-3.183301385
-5.646661536	-1.467799462	-4.127382775	-1.952600431	-4.162998167	-3.711760741	-3.050567106	-8.190905273	-1.172635575	-2.159807349	-6.080656027	-5.38552553	-5.01816363	-2.493527372
-5.46744955	-3.970948421	-5.051910509	-3.337919011	-4.761457741	-4.098885453	-5.026378692	-6.696088721	-1.392399554	-2.56357049	-4.523640285	-3.772871852	-3.291398813	-3.13918104
-5.044764623	-3.390608463	-5.047583359	-3.408941287	-4.690488734	-4.133470909	-4.400574504	-9.063582752	-1.549580672	-2.511399454	-4.688504041	-3.782065004	-3.695871979	-3.140403498
-5.277104538	-2.920019374	-4.591431107	-2.878592501	-4.457113578	-4.026415126	-3.362202236	-8.166038982	-1.029878524	-1.698462123	-5.309691937	-4.323412363	-4.080765819	-3.262191248

-5.082765243	-3.267874593	-4.880297233	-3.065303651	-4.771546029	-4.158207194	-4.458593614	-7.369900135	-1.451736271	-2.83696906	-4.95671082	-4.035280247	-3.632519289	-2.794611556
-4.561365392	-3.737517664	-4.890823758	-3.389669213	-4.350284645	-3.536158274	-4.228619979	-5.524409102	-1.962956263	-2.378423567	-4.884948287	-4.196867523	-3.639928022	-2.605323777
-4.947023626	-2.972231371	-4.85229295	-3.1618689	-4.133878907	-3.575364223	-4.05118306	-6.151306392	-1.55929316	-1.812275535	-4.464206044	-4.711937089	-4.021304427	-2.834892888
-5.40893665	-1.671988861	-4.338528653	-2.122206185	-4.247297376	-3.963591427	-3.091786721	-8.025247025	-1.573824101	-1.896847219	-6.02054392	-4.876676047	-3.57217073	-2.68876699
-5.205018025	-2.488472699	-4.686040166	-2.699111384	-4.766643381	-4.016462558	-3.741300309	-7.449410486	-1.371236206	-2.441551053	-5.453114149	-4.326230246	-4.233069792	-3.071436576
-4.357001717	-3.304863567	-5.294241968	-3.326601332	-3.736553077	-3.849978629	-5.544712643	-4.679708252	-2.090305951	-2.733475374	-5.347633054	-3.959908591	-3.549429056	-2.620853791
-4.624762354	-3.185931189	-5.150039182	-3.39927892	-3.015695966	-4.205624484	-4.237247412	-4.489311814	-2.409918225	-2.626230247	-6.78530723	-4.746618377	-3.62353274	-2.529459019
-5.553791574	-2.628618407	-4.549678	-2.748830337	-4.631863037	-4.03246262	-4.636946546	-7.379310743	-1.503076104	-2.40225226	-5.325802786	-4.409591444	-4.027911906	-3.162707252
-5.234634198	-3.644439561	-4.940826659	-3.410629933	-4.632915541	-3.904189758	-4.727313417	-5.059046657	-1.624695556	-2.49884404	-4.899684667	-4.253848966	-3.161895678	-2.668882131
-4.622153764	-3.583888603	-5.016558767	-3.245124976	-4.679418146	-3.86076523	-4.728175873	-6.706037332	-1.678640081	-2.355448891	-4.78354302	-3.842114643	-3.450031482	-2.884063163
-4.640453227	-3.690154748	-5.582111389	-3.570278982	-4.611759311	-4.298013442	-5.133150313	-5.940531457	-2.765556249	-2.555724609	-4.751565264	-3.765364307	-4.264672398	-2.96227399
-4.532151964	-3.919243927	-4.767985019	-3.129522828	-4.487405421	-4.026650244	-4.573953591	-5.913217846	-1.889122694	-2.06655884	-4.588098303	-3.684326319	-3.925067691	-3.059681264
-5.433136471	-2.805349548	-5.377519388	-3.473430055	-4.780651608	-3.837033016	-4.286013376	-6.769830037	-2.134291085	-2.614804256	-5.254957048	-4.785026544	-4.792264891	-3.170958585
-4.564790594	-3.161586447	-5.591453379	-3.928327971	-4.938793075	-4.146367613	-5.303269341	-5.266181883	-2.215012437	-2.350368828	-5.511945756	-4.342845327	-4.340270939	-3.052423613
-5.067907904	-3.305856671	-6.644960139	-4.077070896	-5.022881253	-4.347642752	-5.684514483	-4.868773444	-2.502356255	-2.920668527	-5.881860904	-4.631822002	-4.91398735	-2.792928824
-5.01130846	-3.292237921	-6.117646593	-3.406540375	-4.274921512	-3.843928165	-4.811937155	-5.839786209	-2.288652091	-2.519116634	-4.927355073	-4.666496262	-4.201432443	-2.883610458
-5.228949521	-3.636291239	-5.05183702	-3.651115976	-4.089417713	-4.094093756	-5.003267144	-7.464295029	-1.886107255	-2.390573883	-4.585098066	-4.317723302	-3.84029328	-3.345204545
-4.886360921	-2.497073652	-4.389415741	-2.988358982	-4.278042793	-3.842326663	-4.294163636	-7.784980899	-1.626574977	-2.261378835	-5.430308746	-4.377957838	-4.34248429	-2.932813591
-4.956485	-2.991069114	-4.515769153	-2.755965766	-4.61070748	-3.702480779	-3.907036786	-6.588862464	-1.030088085	-1.827244837	-4.877896486	-4.064189186	-3.572669436	-2.992849708
-4.681848483	-4.43177908	-5.532103791	-3.542008974	-5.257889158	-4.013137831	-4.56383252	-5.99279416	-1.897266456	-3.051199596	-4.630560695	-3.783578695	-4.78546803	-3.181065643
-4.986224737	-3.057937195	-4.732341947	-2.828511644	-4.598754178	-3.470784116	-3.784116541	-6.647300061	-1.355241873	-2.425835605	-4.595526733	-4.255341423	-3.890575878	-3.273027828
-4.012783563	-3.335628328	-5.123781702	-2.844441156	-4.588990238	-4.088036755	-3.707173794	-4.358933271	-2.372622252	-3.174535101	-5.469008139	-3.49763691	-4.93727192	-2.781950902
-5.167385008	-3.127204933	-5.332441415	-2.882525173	-5.233603846	-3.983653016	-3.194870933	-7.777812673	-1.855840955	-3.464386549	-5.641567612	-4.400924507	-3.518842674	-2.946699107
-4.947132514	-3.502159245	-6.092854966	-3.134549479	-5.167490925	-3.5388774	-4.278526504	-6.242499564	-2.162145267	-2.930095207	-5.868023562	-4.03928564	-4.491544809	-3.004198885
-4.95962843	-3.302429228	-5.14368871	-2.706780387	-4.947134648	-3.664289571	-3.284076028	-6.708330616	-1.710459605	-3.006920005	-5.492455747	-4.665791481	-4.031036005	-2.488347889
-4.615665697	-3.239399298	-5.241105772	-3.183342944	-4.580435609	-3.783523034	-3.862168637	-4.052949887	-2.211208344	-2.938042884	-5.091741897	-3.956282717	-3.842961761	-2.648914347
-3.76742692	-3.43594779	-5.351841615	-2.804538381	-4.39009159	-3.277761501	-3.176882231	-3.587402001	-2.355043388	-2.640132058	-5.11356941	-3.904405959	-5.537391084	-2.621880497
-4.222190143	-3.358904931	-5.054320755	-2.657603937	-4.659746337	-3.144955635	-2.786316778	-4.27991503	-2.178549456	-2.971875263	-4.573927605	-4.199710263	-5.447435713	-3.172298775
-4.093014012	-3.544200759	-5.170652349	-2.772553717	-4.57483548	-3.615420436	-3.730172162	-4.490389952	-2.228484614	-2.783503397	-5.146508064	-3.919696373	-5.025318026	-2.608087461
-4.540476134	-3.247810931	-5.184037817	-3.264433344	-4.781652817	-4.007929644	-3.667936949	-4.365756133	-2.285843708	-2.780136497	-5.21280687	-3.999714199	-4.175991674	-3.021931555
-3.729208498	-3.634610761	-5.490535569	-3.332510405	-4.552987653	-3.757384192	-4.028280004	-3.705335858	-2.635339983	-2.904520289	-5.582878721	-3.743799456	-4.832423918	-2.507105098
-4.684623872	-3.077214584	-5.697672926	-3.321752388	-4.613456881	-3.316164946	-3.291987144	-4.00446303	-2.369859632	-2.877792692	-5.366692849	-4.718488097	-4.965304029	-2.773238114
-3.809670514	-3.295044782	-5.105797978	-2.751684337	-4.471537078	-3.903022889	-4.002727607	-3.934261906	-2.408836743	-2.794435726	-5.359896323	-3.465118597	-4.949040268	-2.682078576
-3.639963625	-3.443911551	-5.137856181	-2.825497054	-4.250287605	-3.596487058	-3.673224785	-3.757128039	-2.440639147	-2.891379542	-6.031160772	-3.492185293	-4.879297377	-2.695177228
-4.89913265	-3.600123663	-5.006477616	-3.349550113	-4.705151096	-4.026866126	-4.423633988	-6.866815626	-1.950694824	-2.33779091	-4.946742168	-4.00914846	-4.141768665	-3.242191097
-5.144134507	-2.946504175	-4.67469861	-3.240173589	-4.338455676	-3.823349355	-4.676388933	-5.984174368	-1.490162155	-2.299313815	-4.655105038	-4.423019425	-3.530702951	-3.011939428
-4.599936392	-3.210965464	-4.950572212	-3.781457301	-4.322802535	-4.039689473	-4.903544945	-5.265788191	-2.267012116	-2.444470573	-4.028073889	-3.742310845	-3.997782736	-2.892013971
-5.398013751	-3.613828908	-4.940211346	-3.494310459	-4.38213627	-4.236062553	-4.643990635	-4.985643037	-1.77945495	-2.298276249	-4.5772886	-4.01328722	-3.946531661	-2.889161267
-5.468099934	-3.622547775	-4.800708338	-3.658656477	-4.519558956	-3.964075037	-4.550326106	-6.677549512	-1.793684178	-3.164658307	-4.633089946	-4.35098707	-4.166077025	-3.194580425
-5.524423206	-2.451685091	-4.66645237	-2.96620954	-4.307651516	-3.387553149	-3.671858844	-4.69693877	-1.826839534	-2.720924869	-5.983594763	-4.69424341	-4.137063578	-2.431265703
-4.659984855	-3.821983791	-4.852554389	-3.3742291	-4.551448077	-4.038697752	-4.936993716	-5.803740933	-1.83888171	-2.479113753	-4.655659397	-3.758463432	-3.780089263	-3.030148283
-4.584057736	-3.795614528	-4.977217859	-3.49758476	-5.009425218	-4.09078826	-5.217032589	-6.507838538	-2.13555485	-2.71340195	-4.772100308	-3.980406309	-3.994311568	-3.160865046
-4.054605617	-3.638204733	-5.522674617	-3.604651009	-5.031281877	-4.218086975	-5.500351729	-7.11168731	-2.012002328	-2.273816218	-5.435710575	-3.8197382	-4.192572807	-2.581105821
-5.12177974	-2.667990569	-5.497515021	-3.479755658	-5.173434959	-4.215164956	-5.795399499	-7.078859904	-2.400204997	-2.58933737	-5.471876427	-4.786502101	-5.351408923	-3.231433423
-5.342086783	-2.967284703	-5.789374378	-3.797522915	-5.254536008	-4.063870771	-4.538158593	-6.857713257	-2.337698307	-3.016028942	-5.47964921	-4.958866719	-5.118515031	-3.177573017
-4.880762792	-3.643298837	-5.609804511	-3.687535042	-4.742199173	-4.323499188	-4.992586779	-7.587645222	-2.42722491	-2.863523017	-4.568607203	-4.022135885	-4.008993225	-3.103957229
-5.268701776	-3.387457207	-5.312178907	-3.382701794	-4.914639254	-3.662314433	-5.23762745	-4.768588528	-2.017091179	-2.716562646	-4.987077124	-4.624553047	-3.618061599	-2.882413013
-4.47447219	-3.623312026	-5.09823847	-3.953711048	-4.777812428	-4.012962651	-5.623134208	-8.477391002	-2.046140839	-2.220785328	-5.033036993	-4.025377651	-3.783341898	-2.901016402
-4.884226254	-4.018440816	-5.140536631	-3.61634478	-4.506413956	-3.710525987	-5.017618358	-4.93825254	-1.827763451	-2.755462593	-4.990195471	-4.145208915	-4.418309567	-2.970515073

-3.821486664	-3.425459154	-4.914499871	-2.702147839	-4.285106452	-3.602366834	-3.5649009	-3.843783171	-2.248346377	-2.542106612	-5.069540601	-3.743437397	-4.729296173	-2.472933095
-3.181354098	-3.464336805	-5.145493206	-2.708565591	-4.159130658	-3.651885211	-3.662908758	-3.347827351	-2.350196953	-2.459601547	-5.623520973	-3.442526694	-4.583872009	-2.636058903
-3.736946209	-3.567689302	-4.888035735	-2.812304841	-4.286113972	-3.787687611	-3.727092484	-3.845957257	-2.221808012	-2.816607137	-5.294334667	-3.314380462	-4.192577211	-2.646010348
-3.978589413	-3.717974444	-5.003174567	-2.831543745	-4.281816139	-3.595356118	-3.54473718	-4.493124568	-2.3200392	-3.01530286	-5.752845661	-3.657569085	-4.619101662	-2.647892438
-4.197751257	-3.456124545	-5.030636227	-2.939027907	-4.990295354	-3.915875453	-3.575055535	-4.550828696	-2.256902414	-3.049762859	-5.260421611	-3.691156544	-4.210855205	-2.79706711

hsa-miR-574-3p	hsa-miR-575	hsa-miR-583	hsa-miR-584-5p	hsa-miR-602	hsa-miR-6068	hsa-miR-6069	hsa-miR-6073	hsa-miR-6075	hsa-miR-6076	hsa-miR-6081	hsa-miR-6086	hsa-miR-6124	hsa-miR-6126
-3.699551842	-0.973785007	-6.256714992	-4.308043824	-4.630381051	-4.691974401	-4.871805033	-3.559421347	-1.380130993	-3.002637998	-4.621555134	-3.132654231	-0.323181642	0.49443518
-3.795333423	-1.127741491	-5.299691855	-3.371669322	-4.40303975	-3.465949405	-5.116926761	-1.496760148	-0.393803031	-2.592180109	-4.520086921	-3.589040097	-0.791303416	1.103062786
-3.670038322	-0.231276365	-5.487369504	-3.600906321	-4.270302746	-3.512992677	-4.969311435	-2.297278025	-0.22672793	-1.874264046	-4.687559345	-3.560419936	-0.544981316	1.297552767
-3.763210944	-0.2459564	-5.474668231	-2.710383829	-4.427987938	-3.49508833	-5.00121603	-2.910183021	-0.146872439	-1.766582585	-4.622782076	-3.418245652	-0.416724528	1.409088362
-3.293391804	-0.019346527	-5.583391827	-3.938873321	-4.160702626	-3.807328269	-4.537976169	-2.37376821	-0.740341273	-1.976809129	-4.273774764	-3.220038347	-0.160257193	1.275663027
-3.616630034	-0.682843193	-5.735308926	-3.176431524	-4.235762357	-3.537533845	-4.830520899	-2.034027462	-0.301960976	-2.453419016	-4.374015981	-3.480458941	-0.438431926	1.227480814
-3.496049176	-0.080347057	-5.702191209	-3.714838937	-4.141003227	-4.029774706	-4.768077136	-2.537737416	-0.315707646	-2.100887582	-4.450824946	-3.302403372	-0.221788225	1.291743901
-3.746521403	-1.131243556	-5.172902614	-3.240633493	-4.340170585	-3.279655045	-5.033868576	-1.803783607	0.218446097	-2.008019108	-4.60135927	-3.565851853	-0.7802635	1.416363883
-3.90000901	-0.384013436	-5.80953793	-3.529124132	-4.510470842	-2.789560926	-5.085460555	-2.180635795	-0.060371644	-2.142230864	-4.697672947	-3.506369693	-0.735417368	1.057179357
-3.833011002	-0.104572278	-4.988575972	-3.680142245	-4.308503114	-3.788727423	-4.604573074	-2.813337713	-0.355943133	-2.40356908	-4.166743217	-3.520169739	-0.788328045	1.257256479
-4.000846965	-0.653468336	-5.080628838	-3.568614083	-4.270083933	-3.419210278	-4.864845021	-1.597764561	-0.285110211	-2.148944147	-4.641037242	-3.801788417	-0.809240705	1.241554378
-4.488479767	-1.372394253	-5.472647295	-5.331417566	-4.598698832	-4.162400706	-5.548560346	-2.980542184	-0.532178078	-2.65442635	-4.765020663	-3.525701118	-0.82643356	0.785774879
-4.549703738	-1.103838927	-5.380433716	-4.421015594	-4.801883122	-3.397593192	-5.822119435	-3.391679987	-0.335855102	-2.359581581	-5.108611872	-3.757993203	-1.072607217	0.905318061
-4.114522247	-0.540911371	-5.373384318	-4.127873776	-4.631916059	-3.835821706	-4.943261291	-3.392946262	-0.587017544	-2.443199821	-4.70899823	-3.912723529	-0.959206072	1.134544656
-4.227551727	-0.678819267	-5.028993141	-4.18096725	-4.26248286	-3.823227666	-4.981920569	-2.439585659	-0.653231618	-2.84232458	-4.483175322	-3.892107887	-1.226999766	1.127092325
-4.305559817	-1.04448295	-5.304588488	-4.57065205	-4.065484556	-3.534667079	-5.288207495	-4.125133546	-0.0829949	-2.424757905	-4.735046203	-3.74381514	-0.968853738	1.073364356
-4.193464144	-1.605942627	-4.985601294	-5.036275688	-4.216128188	-3.545833497	-4.825610257	-2.809900445	-0.463605434	-2.631093097	-4.507390829	-3.732366499	-1.204896973	1.180015276
-4.470240597	-1.307183077	-5.359567865	-4.005294938	-4.582775328	-3.242535904	-5.048955445	-2.399518796	-0.335919963	-2.547339205	-4.772092501	-4.351096213	-1.091031826	0.852016462
-4.110152005	-0.938858264	-4.689357821	-3.651108492	-4.694753473	-3.781498283	-4.969797707	-3.180641773	-0.793266768	-2.254996794	-4.735445397	-3.867423758	-0.626361546	1.053472125
-4.159485411	-0.97174656	-4.974077286	-4.241569606	-4.301547962	-3.252897118	-4.944474219	-2.146527113	-0.218943979	-2.15326656	-4.569219878	-3.738551311	-0.903619473	1.316193336
-4.19853923	-0.975391706	-5.427653032	-4.022836277	-4.201470504	-3.931286921	-4.920974083	-1.485968256	-0.603726953	-2.798871319	-4.618664113	-3.755882506	-1.166001796	1.132768838
-4.212976191	-1.438946215	-5.191998517	-4.906522382	-4.190834481	-2.65865495	-5.021202109	-1.680135778	0.151100458	-3.062817608	-4.572615517	-3.71388791	-1.355741174	1.143243235
-4.133724482	-0.162715217	-5.164413988	-3.752787874	-4.48584803	-2.848243353	-5.183601781	-2.144745157	-0.049092197	-1.951080446	-4.626410721	-3.651453272	-0.499898902	1.262331736
-3.955515824	-0.064935079	-5.519691095	-3.982143127	-4.312835645	-3.499060608	-4.436184866	-3.343958271	-0.509210252	-1.601924152	-4.458268658	-3.069564569	-0.087229926	1.166821884
-4.157063807	-0.828199957	-4.803084234	-4.400572917	-4.307443287	-3.346133196	-5.199928522	-2.53793587	-0.281405179	-2.008118407	-4.516201773	-3.817647879	-1.070691133	1.368291984
-3.838738314	-0.45209542	-6.253034088	-4.593518674	-4.464889086	-3.897978925	-4.465983363	-3.484348107	-0.609805004	-1.672163637	-4.342620574	-3.019714859	-0.098554347	0.965857244
-4.267404022	-0.827155846	-5.349953985	-4.484817893	-4.749785026	-3.915935163	-6.056647049	-2.616144438	-0.449282922	-2.256108697	-5.406778439	-3.642827929	-1.17588638	1.000010051
-4.279493282	-0.331044455	-5.525727346	-4.574510496	-4.818690839	-4.513453207	-4.914909273	-2.593886143	-0.999067319	-2.194758987	-4.69961411	-3.391868827	-0.637029128	1.075302103
-3.98201071	-1.677517379	-5.896148868	-4.76750361	-4.884710346	-4.333176381	-5.071792781	-3.394355859	-1.028214701	-2.956002677	-4.810289037	-4.173947432	-0.802944416	0.883065318
-3.951425728	-0.143605731	-5.21284656	-3.354119449	-4.45325656	-3.299302852	-5.023195876	-2.830226582	-0.263383092	-1.842030307	-4.708925466	-3.778993619	-0.720952047	1.432009251
-4.024775922	-0.295635063	-5.32774649	-3.931110454	-4.501104639	-3.151718635	-5.234115649	-2.039917209	0.051484168	-1.888445279	-4.95794081	-3.489336008	-0.672395655	1.316128223
-4.93270584	-3.34924152	-6.106201806	-6.123012517	-6.190398807	-5.859259926	-5.134210529	-3.525507052	-2.338570744	-3.928659678	-5.346732858	-4.296105189	-1.836920772	0.383320362
-4.494273486	-2.138493573	-5.447819274	-5.004769886	-4.440991918	-3.42091215	-5.138663639	-2.701568511	-0.191508917	-3.27779897	-4.940090654	-3.801627569	-2.351579599	0.805242649
-4.402417785	-1.619989972	-6.422563332	-4.936555787	-4.23352533	-2.011535539	-5.063705135	-2.892605589	0.384166781	-2.837515005	-4.726756089	-4.165723234	-2.302977549	0.595929889
-4.78811528	-1.513650865	-5.500497823	-5.707596034	-4.331930457	-3.514918259	-5.353856889	-4.634780504	-0.641850175	-3.126597729	-4.817384041	-4.497866178	-2.044654381	0.979440587
-4.10936898	-2.152208615	-6.162549251	-5.432644459	-3.696197672	-3.068830881	-4.723391912	-2.959213597	-0.217937157	-3.32079309	-4.15722115	-3.964798169	-2.160770365	1.024567625
-5.27045038	-2.234389045	-5.293386804	-6.588833446	-3.859636665	-3.649055879	-6.305926257	-3.029204537	0.18291249	-3.321810458	-5.309833408	-5.373442089	-2.982515371	0.674448015
-4.297062478	-2.129530771	-5.472139947	-5.43037481	-4.345422261	-2.383858653	-5.212727288	-3.029500965	0.01157456	-2.892977023	-4.714901279	-4.657485171	-2.255083905	1.024262525
-4.02503437	-0.928818415	-5.588337583	-5.066295655	-3.747981843	-2.871672143	-5.271395182	-5.073319798	0.018016814	-2.47230524	-4.596895149	-4.5117336	-1.488239069	1.263741571
-4.401349424	-2.192426141	-5.627997917	-5.102552666	-4.255836425	-3.756021	-4.830434818	-2.906846868	-1.075821603	-3.152669649	-4.755711013	-4.845895794	-2.694441494	0.141595308
-4.063620697	-1.413407737	-5.621973891	-5.18038798	-3.839106046	-4.970919912	-5.126456109	-2.861549523	-1.170293295	-3.384967978	-4.472012486	-4.009785435	-1.59486741	0.87318892
-4.397692415	-1.00568357	-5.663992991	-4.014789667	-4.498917742	-2.620278708	-5.306100945	-2.503387433	0.631039778	-1.71695547	-4.502999712	-4.678712794	-2.037799559	1.334245626
-4.787998759	-2.111236842	-5.347839488	-5.30064155	-4.620465599	-2.768561237	-5.914373069	-2.976114767	0.357451419	-1.830921945	-4.826114911	-4.613909576	-2.492898268	1.315432962
-3.628125809	-0.485632067	-6.267084413	-6.51565759	-3.816641619	-5.769826735	-4.334437159	-5.815889734	-2.129344677	-2.619406334	-4.337714181	-2.93446177	-0.402978742	0.352688687
-4.356153529	-1.68069892	-5.626591169	-4.744019969	-3.848067747	-3.015524225	-5.391511187	-3.322839703	0.207128604	-2.188326697	-4.333644874	-4.814287662	-2.094905983	1.324418206
-4.239041969	-1.566561443	-5.342291058	-5.533135829	-3.885236207	-3.041397846	-5.538100704	-3.473950204	-0.008922909	-2.405233223	-4.500377536	-4.678571592	-2.337802926	1.228112271
-3.871488857	0.243076976	-5.654309946	-5.436944062	-3.385506528	-3.486656049	-5.106913685	-3.694417703	0.007273178	-3.045735587	-4.515191374	-3.812798457	-1.16724678	1.200313035

-4.526832082	-1.251792732	-5.287030234	-4.177607332	-4.140175693	-2.670231829	-5.280264523	-3.115257027	0.159366718	-2.086325035	-4.656779926	-4.479958959	-1.639496918	1.347688637
-4.457525696	-2.322048677	-5.193102656	-5.325249015	-3.707210354	-2.988207564	-5.27958792	-2.190554414	0.005826103	-2.414643995	-5.032314898	-4.094306536	-2.670521702	0.901431882
-4.145851636	-2.131537582	-5.462093463	-5.497554515	-3.950987394	-3.379714645	-4.879387922	-2.836231001	-0.559768003	-3.377708671	-4.405243344	-4.016396471	-2.191883612	0.704357365
-3.843892711	-0.902767809	-6.081755894	-6.415455106	-4.4260647	-2.656018845	-4.319814232	-5.411149905	-0.654133435	-1.939592074	-4.666522849	-3.268800775	-0.503488312	0.80266937
-3.996968768	-0.907810383	-6.126201651	-6.580882649	-4.535785299	-3.681533016	-4.735087806	-4.88373395	-0.837880569	-2.070827019	-4.486915692	-3.756261008	-1.296688682	0.990019776
-4.573362241	-2.570722644	-4.909443355	-5.316018474	-4.786504628	-2.902834066	-5.25037492	-2.374295429	-0.135128301	-2.251279315	-5.144188197	-3.943069251	-2.787024786	0.385693121
-5.105151011	-2.616529752	-4.490496901	-5.845454447	-4.120687095	-3.150869675	-4.018678015	-3.691200367	-0.498322189	-2.932845341	-4.852620266	-3.984356168	-2.894302351	1.094294045
-3.940812319	-1.77601888	-5.85910013	-5.272160326	-4.178937729	-4.004315805	-4.510647001	-3.698033151	-0.820134186	-3.182050029	-4.536228142	-4.152364445	-1.505319938	0.700980744
-4.416595254	-1.343726696	-5.870869645	-4.531394313	-4.082988676	-2.341782801	-4.943164586	-2.505393321	0.284896123	-2.126055391	-4.713482071	-4.627126234	-2.29595416	0.988493166
-4.54027928	-1.547116911	-5.931640049	-5.190508097	-4.210265568	-2.159145167	-5.009607789	-3.491927934	0.599561123	-1.783640451	-4.677409377	-4.518496696	-2.062300934	1.18275681
-4.254398425	-1.947544418	-4.906053064	-4.482230531	-5.091501441	-3.160322815	-6.308058172	-2.458065216	0.111134347	-2.195532802	-5.316274625	-4.5992787	-2.18245457	1.649922097
-4.30185796	-1.072500878	-4.845843454	-4.884614497	-4.149494895	-3.229192296	-5.779961964	-3.013277304	0.254333811	-1.820267595	-4.911568719	-4.340915688	-2.192961107	1.330191474
-4.467884697	-1.828887225	-6.351065063	-6.712947422	-4.326783389	-4.365315792	-5.305040616	-5.00456189	-1.323307668	-2.92300866	-4.875568502	-4.635452858	-2.002730843	0.184348346
-4.478384755	-2.128344318	-5.797919998	-5.144603295	-4.716731435	-3.105167692	-5.70702524	-3.397175947	-0.223631813	-2.049588787	-4.885035791	-5.052170778	-2.877024597	0.300092433
-5.209632702	-3.214544044	-5.126060278	-6.693187176	-5.440912078	-3.640024909	-5.759674148	-2.469990356	-0.60740398	-2.583109379	-5.093296841	-4.876250556	-2.813646848	0.562169108
-4.788324991	-2.810564834	-5.102362269	-4.271069371	-4.45988739	-3.587446897	-5.564121474	-2.667783402	-0.398758123	-3.223987727	-5.533835283	-4.145186752	-2.331383113	0.759801287
-4.491062544	-1.77401025	-6.041489266	-3.724192867	-4.150009144	-3.326304628	-5.278573424	-2.334575498	-0.088871492	-2.584316753	-4.853296105	-4.714379424	-2.019301744	1.210728045
-3.925299639	-1.600075715	-6.843644078	-6.208995003	-4.55281428	-3.509116665	-4.757551351	-5.092521696	-1.214343024	-3.140874874	-4.617185962	-4.035106005	-1.411229798	0.048160202
-4.30894655	-1.24995006	-5.412535916	-6.116464549	-3.636965748	-2.883784453	-4.928462946	-4.492857707	0.191299417	-2.4084169	-4.520662964	-3.803501134	-1.581135459	1.495342966
-4.657447358	-1.080173896	-4.975717444	-4.898094996	-4.63946209	-3.127268663	-5.775883384	-2.108302361	0.491926107	-1.700347927	-4.61662955	-4.577255532	-2.026497996	1.698877384
-3.98555877	-0.698386531	-4.941728037	-5.4251663	-4.010439567	-3.199742368	-5.029040069	-3.669446189	-0.117977562	-1.986659486	-4.401682778	-3.815731873	-1.241509893	1.531120887
-4.601248989	-1.860193474	-5.770250666	-4.563268506	-5.086257818	-3.81617517	-6.041571499	-2.563138435	-0.782849684	-2.238077118	-6.161782171	-3.833122858	-2.077878779	0.8418013
-5.046735465	-0.569534183	-4.905947246	-4.999206488	-5.043521237	-2.884245298	-5.884687547	-6.248278862	-0.168536015	-2.859346245	-5.458465438	-4.043090954	-1.426060817	1.047729874
-5.535550518	-2.350100063	-5.096982086	-5.924063722	-4.939405041	-3.334804821	-6.899534023	-1.577561722	-0.156687217	-3.283543823	-7.005114345	-4.054560235	-2.042097894	0.878770871
-4.59577555	-1.910857531	-4.970714161	-4.411539666	-4.786095837	-3.394649478	-6.013452021	-1.590333862	-0.512440893	-3.321816339	-5.544420506	-3.973094652	-1.591803917	0.890332436
-4.463768323	-1.620721039	-5.749112071	-4.994421296	-4.555847987	-3.591891502	-5.970041061	-3.365064979	-0.388989673	-2.09930094	-6.010018789	-4.503647111	-2.163384694	1.315114323
-4.544973692	-2.633490586	-6.195964966	-5.322521001	-4.528809945	-4.718842668	-5.834857034	-3.497331222	-1.386929141	-2.207172568	-6.107949066	-3.466019956	-2.54643299	0.580074537
-4.410636292	-2.560616288	-6.641951073	-6.012854689	-4.24084564	-5.114920844	-5.325180677	-4.36805885	-1.559328177	-2.247322051	-5.552568668	-3.433957851	-2.143874223	0.284089503
-4.428575986	-1.478048746	-5.994286591	-4.738040846	-4.850522849	-4.375658194	-5.62198111	-2.556484284	-0.948436455	-2.12759086	-6.030388214	-3.743756748	-1.78650702	1.002581198
-4.091195969	-1.475369198	-6.166027744	-3.832889141	-4.955794867	-3.627306267	-5.492689356	-2.686875635	-0.237940191	-1.864693282	-5.480780886	-4.236855153	-1.489013287	1.368839567
-4.435787449	-2.781078154	-5.432815638	-5.571354556	-4.877518377	-3.69672991	-5.775172949	-3.131575948	-0.630451521	-2.124814467	-6.153178353	-3.784388022	-2.377034495	1.069989063
-4.537501092	-2.000433907	-6.303559403	-5.621206035	-4.611907059	-4.365984615	-5.378922241	-3.543183497	-1.169787575	-2.702486028	-5.087258351	-4.032952646	-1.833016008	0.990859047
-4.503849962	-2.375161738	-5.876931666	-4.761010187	-5.007914955	-4.077491132	-6.194684931	-2.597861451	-0.719776204	-2.14717186	-6.290423315	-3.704388173	-2.069734898	0.828081635
-4.637854934	-2.775174923	-5.598380888	-5.085000738	-5.176451981	-3.788096342	-5.431588795	-2.718158579	-0.849159609	-2.154924089	-6.300971927	-3.58404736	-2.038412009	0.791102578
-4.406337881	-1.745846898	-5.794025797	-5.141365334	-4.856221896	-3.064079087	-5.497101864	-3.212683486	-0.226981703	-2.725538502	-4.644398434	-4.661221365	-2.169542259	0.82237382
-4.217638268	-0.969364708	-5.844061011	-5.426486202	-4.088785298	-2.95322961	-4.931751084	-3.350646125	0.108894742	-2.660008861	-4.560844205	-4.180372851	-1.769542632	1.071192729
-4.490418759	-2.177073099	-4.877603445	-4.417924647	-4.470874028	-2.861635528	-4.94418427	-2.099809681	0.292464489	-1.669137244	-4.610014355	-4.628135385	-2.383808348	1.584400778
-4.21114676	-1.827273368	-5.054488218	-3.697833355	-4.049927698	-3.369795281	-5.70507055	-2.137855847	-0.01906394	-1.89162651	-5.340237478	-4.390439028	-2.152870004	1.392125005
-4.362097398	-2.349178986	-5.95148297	-5.004400103	-4.02363863	-3.721840674	-5.581841702	-2.955877182	-0.125530505	-2.915797915	-4.845163369	-4.685638792	-2.511761008	0.563316902
-4.221337746	-3.506661381	-4.93866157	-5.315975905	-4.226401064	-4.335113118	-4.400099097	-4.64790799	-1.119005983	-3.761065692	-4.84212291	-4.461480979	-1.760736396	0.478005934
-4.320950006	-1.418040301	-5.119016422	-4.166050007	-4.324653013	-2.738596582	-5.398687143	-3.023341057	0.26022963	-1.908994883	-4.82683766	-4.704848121	-2.325118751	1.322630155
-4.456764883	-1.407817712	-5.314039789	-4.350531531	-4.231545765	-2.593200354	-5.322360802	-3.005192313	0.538641108	-1.476967299	-4.748001548	-4.595262558	-2.036948038	1.562127751
-4.648805646	-2.039255465	-5.599718684	-5.144813108	-4.645252778	-2.844355935	-6.015807744	-3.732308986	0.282058971	-1.092247441	-4.866709381	-4.768679253	-2.144942344	1.122352957
-4.593192439	-3.248770065	-6.545066739	-6.598640001	-5.348989978	-4.268578622	-5.055170434	-5.580932386	-1.02596374	-3.243657228	-5.083064079	-5.127163163	-2.231056888	0.156931594
-5.049430223	-1.921140297	-6.207265209	-7.418031985	-5.213755529	-4.046799791	-5.10562715	-5.08051608	-1.345517861	-2.770395643	-4.942230486	-4.39732064	-2.251054774	0.137165207
-4.795873365	-1.731870192	-5.690823006	-4.987778692	-4.56750828	-2.666419554	-6.075640409	-2.600806587	-0.025596419	-1.971960469	-5.03355988	-4.921329446	-2.229221349	1.651783681
-4.91010381	-2.697338294	-5.094510639	-4.189778216	-4.271870826	-3.716046782	-4.907859724	-3.480107389	-0.602553744	-3.054430067	-5.406845924	-4.866035521	-2.368708536	0.499897499
-6.895557473	-1.79002325	-6.026443627	-5.242530662	-4.479744496	-2.34075586	-5.157161227	-3.042068899	0.340839161	-1.219011443	-4.856821796	-4.979930855	-2.127970039	1.119553454
-4.428634551	-1.883370455	-4.795438931	-4.28113275	-4.230777177	-3.553552568	-5.588888047	-1.914124036	0.116792318	-1.753360985	-5.112471826	-4.43578982	-2.539359841	1.403797366

-4.20402253	-2.482795371	-5.733339984	-4.873858598	-4.619243701	-3.960955439	-5.613877799	-2.515491938	-0.714651336	-1.883915956	-5.70590184	-3.180363207	-2.121538503	1.175315306
-4.307747833	-2.23643815	-5.521102305	-4.032133004	-4.775898176	-3.936133921	-5.633867924	-2.082488971	-0.769430049	-1.832158102	-6.411321254	-3.276217389	-2.399235135	1.020206608
-4.41964065	-2.023714983	-5.346867956	-4.512662606	-4.631656011	-3.399759745	-6.027098965	-2.1234211	-0.322343635	-1.731400172	-6.181722406	-3.516855985	-1.990974443	1.343527366
-4.50079493	-2.252977564	-5.753957904	-4.763497243	-4.532714531	-3.844257221	-5.87015817	-2.583929553	-0.524461785	-2.056890982	-6.296211154	-3.671015083	-1.910140648	1.10859188
-4.433386086	-1.79262236	-6.214510861	-4.083795874	-5.01955629	-3.648613963	-6.00268981	-2.743808695	-0.669357454	-1.970793773	-6.377664588	-3.741100724	-2.136809473	1.116579813

hsa-miR-6127	hsa-miR-6132	hsa-miR-614	hsa-miR-615-5p	hsa-miR-6165	hsa-miR-625-3p	hsa-miR-634	hsa-miR-636	hsa-miR-637	hsa-miR-642a-3	hsa-miR-642b-3	hsa-miR-650	hsa-miR-6510-5	hsa-miR-6511a-
-3.545499167	-3.130269579	-4.38270017	-2.129114236	-3.390293692	-3.808561274	-5.035031379	-5.404163298	-4.567413024	-0.658732308	-0.250699997	-6.366377924	-1.372052053	-3.843833042
-3.774629734	-2.246974351	-2.858245057	-1.414335163	-3.393298103	-4.217726535	-5.193673725	-5.043444314	-4.584921384	-0.914093142	-0.66360497	-5.876177723	-1.706394042	-3.091325658
-3.658350493	-2.251082112	-4.164784219	-1.641494847	-3.447710037	-3.83397844	-5.139359481	-5.061171167	-4.737103163	-0.767692344	-0.526149317	-5.667152194	-1.403070258	-3.493892288
-3.748464793	-1.777467818	-4.272922839	-1.579137705	-3.372276755	-3.946521394	-5.032194536	-5.255496049	-4.914648556	0.123685977	-0.470421608	-5.603917487	-1.358390379	-2.944343798
-3.436242436	-2.556354039	-4.030151097	-1.792472205	-3.217420229	-3.5243327	-4.661746496	-5.132869668	-4.634252148	-0.080549992	-0.051481059	-5.609111689	-1.256017925	-3.655407652
-3.798912547	-1.867027573	-3.193965233	-1.802549999	-3.492971951	-3.744496387	-5.21231293	-5.079608299	-4.894204217	-0.342008571	-0.385660135	-5.634228392	-1.709536217	-2.975333363
-3.593717748	-2.160662583	-3.552749546	-1.753936448	-3.319794861	-3.681674068	-4.955345982	-5.095703823	-4.738930034	0.208211126	-0.267001135	-5.46740924	-1.464354733	-3.384756922
-3.828776577	-1.810386951	-2.99013578	-1.386992557	-3.50422031	-4.158337336	-5.407562121	-4.941385484	-5.117586393	-0.454611388	-0.713201262	-5.938283065	-1.783949206	-2.726079371
-3.89485678	-2.528504689	-3.785938628	-1.579333528	-3.671109194	-4.111916476	-5.265234188	-5.128565245	-4.954290291	-0.326866142	-0.636437989	-5.771411403	-1.639547655	-3.313450773
-3.802054216	-2.193018488	-2.400238066	-1.380162707	-3.551634443	-4.140316037	-5.123761269	-5.011070695	-4.706701571	-0.243582277	-0.252355475	-5.386389696	-1.770392248	-2.98492388
-3.887031761	-2.278287479	-2.790966667	-0.831692424	-3.769732159	-3.790785994	-5.574229307	-5.118949274	-5.061922478	0.321976518	-0.289121992	-5.912386913	-1.783967534	-3.047037304
-3.763232303	-2.735423004	-3.178341458	-2.580831407	-3.382203621	-4.554837963	-5.564610706	-5.605885932	-4.307107171	-1.452956105	-0.519537942	-6.548260448	-1.847013966	-3.684927613
-3.974370206	-2.581993941	-4.015507768	-2.127218104	-3.552574431	-4.531647802	-5.502756672	-5.535117895	-4.978745295	-0.726836038	-0.684469612	-6.58874223	-1.822235577	-3.197331513
-3.891997301	-1.996030969	-3.651453641	-1.630342873	-3.776517639	-4.092179911	-5.722403321	-5.176774869	-5.138577677	0.37298761	-0.26783187	-5.810036242	-1.717931219	-3.360535282
-4.088299857	-2.486138988	-2.069024421	-1.645281652	-3.871455683	-4.207857839	-5.920681765	-5.221726896	-4.570895503	-1.000750999	-0.293013155	-5.681403922	-2.090844754	-3.495250417
-3.783232448	-2.474152168	-3.119312999	-1.638772216	-3.464807289	-4.235087998	-5.544407757	-5.192702507	-4.803725789	-0.586878784	-0.724743866	-5.950265586	-1.889227024	-3.517156825
-4.249878947	-1.976992221	-2.477286953	-2.487743945	-3.642447354	-4.299508466	-5.602353077	-5.104201312	-4.730375553	-1.71826886	-0.288878353	-5.891818891	-1.959641309	-3.258505528
-4.012046291	-2.370617525	-2.625655654	-1.853235803	-4.006122739	-4.414125513	-6.039826744	-5.08907282	-4.79605843	-0.99949035	-0.812208818	-5.981392935	-2.052769533	-3.259281888
-3.960589861	-2.30099603	-3.117873285	-2.124024208	-3.733508716	-4.002112925	-5.723414003	-5.475017606	-4.618021294	0.471027273	0.048927845	-6.053177379	-1.720255893	-3.133927157
-4.112585744	-1.912524729	-2.717972343	-1.880717043	-3.743745978	-4.028298798	-5.613720362	-5.11092278	-4.928386804	-0.710554157	-0.409705956	-5.799124201	-1.902071705	-3.098069666
-4.100638766	-2.440673487	-2.463870505	-1.588707626	-3.683612647	-4.250719634	-5.555109332	-5.037232358	-4.807957839	-1.136000583	-0.427043871	-5.8006001	-2.107223221	-3.488673122
-4.043096438	-2.514533116	-2.363480738	-1.393706099	-3.933347605	-4.13575167	-5.736917196	-5.144901909	-4.571960592	-1.164015086	-0.1180827	-6.073006665	-2.159419706	-3.645204534
-3.902011281	-2.037352882	-3.406665716	-1.556128455	-3.758909497	-4.111767664	-5.573206846	-5.171840529	-5.093737785	-0.049116242	-0.281227476	-5.978955198	-1.553567996	-3.00114694
-3.768576585	-2.131036688	-3.758269954	-2.503136221	-3.443211261	-3.764970777	-5.298826671	-5.275232118	-4.655485154	-0.144905346	0.221710055	-5.337236581	-1.105964258	-3.390121109
-3.931499072	-2.126243984	-2.196408824	-1.601609088	-3.701871718	-4.271427048	-5.960445798	-5.01418064	-4.930416326	-0.644652379	-0.277348689	-5.748596193	-1.965692326	-3.283427259
-3.907420639	-2.403890329	-4.641370124	-2.871674607	-3.56282684	-3.605239012	-5.252786061	-5.162893074	-4.813325115	-0.104749211	0.216879313	-5.840066892	-1.644773794	-3.801262413
-4.055466819	-2.419568745	-2.896475871	-1.770156471	-3.544008037	-4.131648782	-5.800097883	-5.452312513	-4.931804876	-1.100045219	-0.467551307	-6.86852728	-1.991064753	-3.038358832
-3.932550581	-2.736559948	-3.850305103	-2.621263715	-3.500953182	-4.012102823	-5.414857076	-5.614507009	-4.480727506	-0.479328205	0.286446988	-5.990232726	-1.476589185	-3.899333766
-3.640210746	-3.041578468	-3.299041952	-1.898311545	-3.645859541	-4.659326212	-5.586353151	-5.286164766	-4.392857898	-0.886728219	-0.094582922	-6.576482815	-2.108983133	-4.281924239
-3.670005361	-1.916152483	-3.137752333	-1.574084898	-3.117955392	-4.258398444	-5.450241386	-5.177559155	-5.052269816	-0.323176261	-0.670729546	-5.395767838	-1.670278853	-2.911042341
-3.847794969	-1.401586177	-3.24669567	-1.893888401	-3.530832332	-4.281260042	-5.327296846	-5.255150278	-4.660703344	-0.157124819	-0.67642159	-5.408986552	-1.872255333	-2.803805999
-4.531239618	-2.2357038	-3.610617641	-4.395843692	-3.785193607	-5.007763912	-6.146965375	-6.571491649	-5.341788783	-1.689441049	-1.463833093	-7.007256046	-1.981214632	-3.897340135
-3.686047133	-1.62358346	-1.585767934	-2.075801976	-3.280185217	-4.895555597	-5.586563769	-5.389656692	-4.991521228	-2.140110466	-1.955063736	-5.774757412	-2.848627743	-2.643782314
-4.019439461	0.49972411	-2.129213989	-1.440627899	-3.177826951	-4.866917317	-5.396332895	-5.091509294	-5.023173746	-2.089177482	-2.106957148	-5.586021616	-3.120658424	-0.730729207
-4.204991794	-1.71959154	-3.031975475	-1.902406779	-3.777007857	-4.521789538	-6.004639012	-5.2228506	-5.175795308	-1.42831807	-1.625612025	-6.156824943	-2.52495041	-3.152267269
-3.990907786	-0.586534682	-2.633635356	-1.816623469	-3.364071304	-4.222692116	-5.57783911	-4.856745355	-4.7114365	-2.712308295	-1.876162361	-6.132957299	-2.638302905	-2.228260588
-5.374432758	-0.771253503	-2.021021411	-2.155182739	-3.851791938	-4.151413273	-6.47411448	-4.493850814	-4.890264356	-2.632716311	-1.241314871	-6.62864147	-3.921521676	-2.383090576
-4.284444608	-0.965286433	-2.177254131	-1.445145783	-3.716220505	-4.374476404	-5.849811752	-4.424890274	-4.84632422	-1.858515966	-1.877250916	-6.017900802	-2.922157295	-2.214317498
-4.046814754	-0.606987358	-3.530006538	-1.546226299	-3.986768592	-4.071582479	-5.625526469	-4.787896777	-5.17871953	-1.495833036	-1.578928668	-6.485461166	-2.275369536	-2.29522591
-4.968409946	-0.997050496	-1.188613513	-2.174292692	-3.853773622	-4.719685985	-5.699362899	-4.352100826	-4.909189261	-2.480921263	-1.425875279	-5.95653459	-3.452258786	-2.229474836
-3.961247691	-2.756295134	-2.721244132	-1.747242241	-3.951174726	-4.244635636	-5.645739295	-4.811765539	-4.842772949	-1.648315111	-1.466095413	-6.691568681	-2.450029839	-3.831375556
-4.344532242	0.91917038	-2.393765747	-1.331970556	-3.631179719	-5.005004457	-5.740078323	-4.693585683	-5.15605808	-1.464746246	-1.697123415	-5.762969704	-3.007537191	-1.065194196
-5.200701359	0.48203397	-1.988536203	-2.364753123	-3.870272148	-4.609519255	-6.212804491	-4.795054564	-5.527462386	-1.590409802	-1.183846262	-6.210841849	-3.136623839	-1.136293768
-3.295982783	-3.733926804	-4.95647273	-2.806875536	-3.354213228	-2.938220905	-4.660674784	-4.64300246	-4.77376613	-0.419321689	-0.53571186	-6.589413771	-1.364586468	-4.41652226
-4.41588756	0.243999973	-2.606495345	-1.520560193	-3.708572133	-4.140056674	-5.663590228	-4.733255958	-5.086964102	-1.501471665	-1.546684765	-5.998722387	-2.397014883	-1.528172817
-4.444148381	-0.83839784	-2.718307108	-1.37817693	-3.68633904	-4.41056349	-5.57950467	-4.671796971	-4.860062563	-1.982387682	-1.641646271	-6.677971896	-2.607460904	-2.510200987
-3.864998058	-2.844620279	-4.442608911	-0.896515817	-3.482947933	-3.775824609	-5.274635241	-4.737059808	-4.992916832	-1.166080086	-1.582919019	-5.616928471	-1.843178029	-3.871839172

-4.134456204	-0.675154499	-2.856879769	-1.64487804	-3.694382447	-4.48246832	-5.626435564	-4.885427079	-5.450579221	-1.302764808	-1.660545526	-6.277822768	-2.069168301	-1.817930947
-4.374815631	-0.007848559	-1.36607056	-2.50313726	-3.09422562	-4.43503504	-5.578867485	-4.105525114	-4.581748025	-2.178343654	-1.645071851	-5.275984014	-3.262358835	-1.303660145
-3.856830913	-1.908848565	-1.882862402	-1.383421745	-3.458094453	-4.333300847	-5.606552656	-4.780553664	-4.468459112	-1.74046746	-1.515982104	-6.17389582	-2.729104325	-3.005030181
-3.237067257	-2.630128377	-5.108841452	-2.541483112	-3.465234884	-3.317237501	-4.869929319	-4.893487003	-4.680889486	-0.842412943	-0.468157521	-6.521833174	-1.205779558	-3.557643987
-4.261689051	-0.978684411	-4.083003848	-2.600700381	-3.709206145	-3.545977704	-5.167443281	-5.01635554	-4.814099299	-1.019275949	0.199397684	-5.916131359	-1.922497544	-2.634899737
-4.510229898	0.047886196	-0.090290046	-2.16034382	-3.071778222	-4.987168738	-5.530949489	-5.40227053	-4.752668279	-2.3290145	-1.812809372	-4.658243748	-3.136480611	-1.161428865
-4.649974438	-1.152452512	0.55260323	-2.877792063	-2.921555997	-4.125072855	-6.308956468	-4.825349638	-3.710100288	-2.502070644	-0.963944615	-4.550065978	-3.200169575	-1.921155125
-4.290094201	-1.884525269	-3.141016489	-1.889827675	-3.652124758	-3.444923753	-5.234915628	-5.002123432	-4.985757587	-1.111567219	-0.986286538	-6.419150352	-2.182675189	-3.49660685
-4.507784029	0.40487868	-1.605905111	-1.522977289	-3.502648866	-4.244948295	-5.767463865	-4.682820388	-4.854593513	-1.762495786	-1.63737714	-5.428851936	-2.827720532	-0.721068069
-4.221580199	0.573772094	-2.727348111	-1.165795099	-3.184820375	-4.479312414	-5.65148856	-4.88226721	-5.25318504	-1.698486922	-1.742108721	-5.738041371	-2.41003831	-0.80180906
-4.82390035	-0.232412993	-1.398783807	-2.196644745	-3.772833354	-4.435020967	-5.885713608	-4.603591818	-5.140602231	-2.47220418	-1.088211571	-5.236788456	-2.962293787	-1.700901864
-4.22521652	-0.432972298	-2.434453159	-1.215254065	-3.703459146	-4.47902593	-5.656557619	-4.590183427	-4.949944081	-2.048410503	-1.563749021	-5.249543672	-2.861753797	-2.010332786
-4.435128405	-1.776064267	-3.387934311	-2.419984025	-3.797363596	-4.382846394	-5.821504758	-5.014868614	-4.715931473	-2.557840828	-0.850820426	-6.455715885	-2.913180735	-3.218341947
-4.501503698	-0.05320753	-2.36181368	-1.651603209	-3.48601437	-4.76290728	-5.908943334	-4.762913158	-4.977694914	-2.118567517	-2.063585757	-5.595260261	-3.30246427	-1.262727276
-4.695024194	-0.96975189	-0.687757745	-2.493132735	-3.534066092	-4.740881146	-6.604573553	-4.761890503	-4.892182158	-2.709147077	-1.794324122	-5.610675033	-3.780326374	-1.78883159
-4.399081033	-1.606101209	-0.953972884	-1.116683155	-3.513397286	-4.26895249	-5.863720017	-4.511107975	-4.483351043	-1.773620432	-1.273659402	-5.643152676	-3.002962751	-2.460359682
-4.479666422	-0.707583231	-2.403187497	-1.195035908	-4.132808533	-4.393480742	-6.112417432	-4.352079802	-5.198535109	-1.723773215	-1.552201803	-6.344800185	-2.661136597	-2.616804755
-3.754932256	-2.124456864	-3.84970351	-2.773159602	-3.737906482	-3.937687346	-5.324799101	-5.026318515	-4.740306692	-1.452055511	-0.489010538	-6.733214686	-2.406100517	-3.760178852
-4.05188015	-0.935383896	-3.602145559	-2.133961908	-3.03840539	-4.352341608	-5.576765676	-5.121551543	-5.016752345	-1.69007656	-2.117239065	-5.539952235	-1.915343524	-2.131070581
-4.588802953	-0.345119361	-2.565674875	-2.61000488	-3.789439962	-5.024777903	-6.347353569	-4.952789443	-5.692645117	-1.53954989	-1.359649398	-5.123265174	-2.720627985	-2.082473851
-4.350927532	-0.996018482	-2.874885109	-2.325262998	-3.61647152	-4.193248106	-5.583452653	-4.923211359	-4.863134556	-1.207890011	-0.440058708	-5.640984519	-2.009714116	-2.842555417
-4.467896351	-2.11624234	-1.722548405	-2.144248522	-2.921910889	-4.853553911	-5.240240361	-5.669253874	-4.26753612	-1.17835892	-0.964698212	-4.393600239	-2.907846605	-2.91082852
-3.827112867	-1.57679281	-3.780466225	-3.147594159	-2.938456279	-5.002722869	-6.243801656	-5.912069858	-5.071287176	-1.273378538	-1.514725586	-5.608533317	-2.015947489	-2.721120456
-4.478668512	-1.553854947	-1.269171309	-2.172653863	-3.211804272	-5.747579144	-7.881632052	-6.823640701	-4.57810674	-2.749540789	-1.461815619	-5.819319576	-3.00115146	-2.522325089
-4.191207059	-2.258151267	-2.547635848	-2.287270144	-3.389318712	-4.737115896	-6.03115085	-6.023399832	-5.150125075	-1.557334341	-1.378386138	-6.162129867	-1.973652344	-2.783014024
-4.831259512	-1.775191643	-1.258383557	-1.68106528	-3.336657789	-4.297466143	-5.734087393	-4.468297162	-4.709143164	-1.157778614	-0.919804191	-4.184153607	-2.596077437	-2.534007089
-4.543501874	-2.274919908	-0.547115577	-2.652818892	-2.596050558	-4.812479366	-5.189123056	-5.43954996	-4.021279234	-2.173857589	-0.45924866	-3.737947889	-2.898735569	-2.43297657
-4.367834599	-2.847588327	-1.517399268	-3.055333656	-2.848605384	-4.735621085	-5.098352765	-5.663267116	-3.805435016	-2.301137361	0.170999857	-4.394585869	-2.823073366	-3.288230364
-3.713617883	-2.144800659	-1.591850183	-2.089172936	-2.927510972	-4.92229542	-5.557953718	-5.756879911	-4.538950757	-1.350225378	-0.736411822	-4.345810206	-2.628803208	-2.703553793
-4.512132764	-1.522536932	-2.024658428	-1.59228871	-3.447675041	-4.353128191	-5.620625155	-5.06904739	-4.829621635	-0.728498965	-0.880052465	-4.569676826	-2.571530564	-2.372505807
-3.794775619	-1.756259741	-0.654195293	-2.637016691	-2.811294323	-5.087327458	-5.784875556	-5.61622183	-4.518243717	-1.900276408	-1.014973798	-3.962120799	-2.957651028	-2.320947962
-4.553300042	-2.097910157	-1.004807789	-2.893882861	-2.88803264	-4.741321348	-6.254264808	-5.343140669	-4.184985263	-1.592192591	-0.37166304	-4.383053469	-2.712237856	-2.510503428
-4.182754062	-1.878632626	-1.036453043	-2.703858165	-2.511178862	-4.776024491	-5.399115846	-5.767851482	-4.209982137	-1.299926475	-0.843807864	-3.943398051	-2.735287687	-2.475431411
-3.876086721	-2.118157373	-0.71425353	-2.527598441	-2.596667183	-4.73232838	-5.165774349	-5.713953118	-4.116518603	-1.052247229	-0.791444324	-3.966435235	-2.96380196	-2.45031846
-4.010069912	-0.480544213	-2.722403044	-1.875276057	-3.584815325	-4.777193979	-5.599394382	-5.155040321	-5.386225671	-1.855010395	-1.090195269	-6.27955114	-2.777782678	-1.964650366
-4.002519689	0.117690622	-2.260343408	-1.312672994	-3.667478042	-4.380551561	-5.588452162	-4.608708703	-4.873826766	-1.156448895	-1.47742071	-6.044107339	-2.433288717	-1.255876767
-4.53488844	0.4172828	-1.207410866	-1.810514814	-3.702647558	-4.949922405	-5.488570206	-4.491644811	-4.945178649	-2.033263855	-1.44047764	-5.479267216	-2.774668966	-0.822302969
-4.289717042	-0.737085616	-1.596273639	-1.376385788	-3.790640969	-4.403678491	-5.56131846	-4.355862411	-5.028980015	-1.639710121	-1.874736555	-5.471059587	-2.889346016	-1.980314874
-4.643301885	-0.661862737	-2.139048993	-1.298204035	-4.033643154	-4.74408557	-6.096098306	-4.780814019	-5.297122335	-2.068635394	-2.327484942	-6.166402001	-3.257119111	-1.979233837
-4.588061959	-0.800752849	-1.22084649	-2.138372473	-2.921776707	-3.91911731	-5.263984787	-4.545739398	-4.965817698	-1.72704139	-0.860378218	-5.224844108	-2.449851729	-2.076830129
-4.345886005	0.059633961	-2.229413302	-1.608628426	-3.643369504	-4.375899896	-5.337400009	-4.298115873	-4.958886111	-1.921268704	-1.45427647	-5.699305789	-2.786789901	-1.145979461
-4.326372079	0.274022047	-3.084425249	-1.871240968	-3.773417187	-4.6724007	-5.955265044	-5.002886343	-5.265345213	-1.835423935	-1.801797513	-5.606196033	-2.836511241	-1.186492203
-4.681880284	0.140234138	-3.111296857	-1.576166355	-3.385116737	-4.95022838	-6.290561129	-4.819540291	-5.264147944	-1.899305136	-1.898471988	-5.812929891	-2.984315103	-1.3185704
-5.384718165	-0.896901289	-2.604897851	-3.459090705	-3.782059735	-4.457653354	-6.046618844	-5.361771523	-5.502332272	-1.915479683	-1.720493303	-6.715032173	-3.098455915	-2.43774101
-4.461001544	-1.249343826	-2.889865727	-2.54575166	-4.10781621	-5.03745753	-6.191076766	-5.426785327	-4.674581388	-2.594685478	-0.667643645	-6.139492592	-3.068449107	-2.614806959
-4.572125533	-0.568847051	-1.955007157	-1.307006009	-3.782786287	-4.809094019	-6.309673797	-4.672987131	-5.180499043	-2.288923739	-1.145299223	-6.006728797	-2.701475821	-1.870884015
-4.263228831	-0.728881199	-1.053656113	-1.357376664	-4.007892069	-4.483277293	-6.863986853	-4.477238672	-5.042553662	-2.005906215	-1.653007917	-5.623840511	-3.801972093	-2.006216837
-4.367741079	0.31687808	-3.555341827	-1.998711642	-3.946677689	-4.826166942	-6.282003811	-4.846012671	-5.442726484	-2.114618979	-1.799882925	-6.370851744	-2.951387032	-1.361123289
-4.418148435	-0.375295415	-1.260420008	-2.203539112	-3.640423436	-4.465894548	-5.994039495	-4.626999956	-5.010525613	-1.836247639	-1.638377977	-4.837856889	-3.286670534	-1.875382758

-4.041545126	-1.967728717	-0.759809096	-2.1799223	-2.475030826	-4.889766785	-5.060193788	-5.335608814	-4.376468067	-1.849772014	-0.686543245	-3.773773023	-2.6458586	-2.287763388
-4.133857356	-1.90894436	-0.358156321	-1.787912178	-2.308220353	-4.705142695	-5.018897191	-5.444430838	-4.191955861	-1.679317027	-1.161409796	-3.61572995	-2.839149933	-2.080590943
-4.062292609	-1.399556732	-0.631480678	-2.093615221	-2.495376704	-4.742138338	-5.156505606	-5.413620997	-4.396218179	-1.868143824	-1.182009507	-3.956670022	-2.714947624	-2.007272318
-3.631443558	-1.893751736	-0.836137769	-2.001680811	-2.87866339	-4.752220101	-5.507432279	-5.613167454	-4.41330763	-1.623880137	-0.848561464	-4.275751714	-2.596579689	-2.573745153
-4.422240445	-1.969967908	-2.073432081	-1.748109525	-3.162000339	-4.686513707	-5.309574061	-5.590874653	-4.738670242	-1.152168964	-1.01250511	-4.623187487	-2.519774719	-2.818476287

hsa-miR-6511b-	hsa-miR-6511b-	hsa-miR-6515-3	hsa-miR-6515-5	hsa-miR-652-5p	hsa-miR-658	hsa-miR-663a	hsa-miR-663b	hsa-miR-665	hsa-miR-671-5p	hsa-miR-6716-5	hsa-miR-6717-5	hsa-miR-6721-5	hsa-miR-6722-3
-3.872997567	-4.967693188	-2.219496495	-5.597564215	-2.934968825	-2.78550671	1.304424599	-2.186223809	-2.392915972	-1.408193458	-2.195294746	-4.758760694	-1.553999323	-0.088951524
-3.915167688	-3.961133128	-2.592549459	-5.230599317	-3.400810227	-2.957422264	1.505760735	-2.208251222	-3.488498852	-2.088267184	-2.653816004	-4.080842928	-1.855148191	0.04343079
-4.143085916	-4.844908766	-2.421327775	-5.537962387	-2.979396223	-2.860522811	1.554693113	-2.489923745	-2.978607947	-1.363812507	-2.717804949	-4.33267481	-1.836595193	-0.046855877
-3.95351294	-4.086588407	-2.257305591	-5.422187534	-2.339122118	-2.935121935	1.275056945	-2.778863762	-3.841619134	-1.000905505	-2.722628803	-4.515826699	-1.816423256	-0.073410632
-3.670438557	-4.348603067	-1.924603906	-5.500572684	-2.370277737	-2.755878578	1.472670317	-2.092169618	-2.691043357	-1.142059343	-2.346044841	-4.404117856	-1.465864303	-0.159715258
-3.94748329	-3.692822064	-2.235928175	-5.444401673	-2.61496426	-2.930437946	1.425155956	-1.968850197	-3.638732146	-1.522084432	-2.63291334	-3.906118773	-1.597583083	-0.026813763
-3.885763538	-4.661215144	-1.986756898	-5.703881803	-2.131822748	-2.98316484	1.337732115	-2.230676846	-3.109792204	-0.955025443	-2.611765329	-4.801604583	-1.669316844	0.134725519
-4.013456878	-3.909707658	-2.501565202	-5.48399007	-2.721869114	-2.843577355	1.391286327	-2.562719951	-4.082869586	-1.588563045	-2.913747307	-4.344474701	-1.688400751	-0.112210585
-4.278273823	-4.289396317	-2.510660022	-5.960705661	-2.676920592	-1.967532146	1.400102479	-2.463115776	-3.559581557	-1.517892507	-2.734427043	-4.783722074	-1.795912923	-0.177882306
-4.004277928	-3.846094457	-2.388022308	-5.390466312	-2.418125633	-2.925950113	1.482874906	-2.096671431	-4.166717786	-1.253120602	-2.753807424	-4.269928863	-1.833221422	-0.225214897
-4.172858902	-3.836166827	-2.397969739	-5.679139399	-2.074179101	-2.738075414	1.317965941	-2.656882165	-3.791770569	-0.701568557	-2.913058212	-4.60883369	-1.959216642	-0.216526435
-4.236154442	-4.850879959	-2.675841762	-5.625976808	-3.148919741	-3.363639398	1.17939195	-2.522682022	-3.25233899	-2.160653991	-2.793660159	-4.844420078	-1.932599096	-0.268693014
-4.232280768	-4.276174707	-2.948804904	-6.122094056	-2.811247245	-3.217368861	1.01766959	-2.904260317	-3.267329135	-1.566308977	-2.92640872	-4.957355512	-1.974589647	-0.322662687
-4.280331423	-5.400574448	-2.586783959	-5.923265254	-2.325546258	-3.087791888	1.245841553	-2.46872196	-4.049968853	-0.984938238	-2.874392079	-4.69107218	-1.885412968	-0.283510734
-4.313324601	-4.576885227	-2.664806736	-5.691175394	-2.941329591	-3.340625591	1.539534339	-2.037417506	-3.713766583	-1.784393317	-2.842198997	-4.527583201	-1.876786816	-0.320022729
-4.41792205	-4.572069235	-2.744282568	-5.488689327	-2.710448974	-2.891941848	1.499985494	-2.697059705	-3.518787917	-1.44773198	-2.736323926	-4.594182662	-1.915580732	-0.174318404
-4.338683215	-4.652862837	-2.746819571	-5.466707382	-3.202151687	-3.202060702	1.377852098	-2.101574377	-3.466167952	-2.363277622	-2.772506436	-4.624185673	-1.891395898	-0.182124925
-4.5971663	-4.4133776	-2.944766739	-5.740149484	-3.412192081	-3.125367354	1.562991638	-2.638943258	-3.486056777	-1.826469296	-3.074554836	-4.472623763	-2.422227163	-0.240731457
-4.293223695	-4.079705999	-2.607702363	-5.643908435	-1.965206155	-2.999894911	1.246899468	-2.407432413	-3.034301195	-0.411672171	-2.6450857	-4.600592124	-2.058072534	-0.346456709
-4.417621229	-4.393031712	-2.655709035	-5.883640613	-2.618358307	-2.966798537	1.4762637	-2.415578723	-3.743009869	-1.534448669	-2.80418374	-4.789780302	-1.956575549	-0.250785831
-4.413733996	-4.502845191	-2.753101211	-5.742252408	-3.222107416	-3.00283359	1.429069602	-1.90258514	-2.834033946	-2.164986421	-2.728770518	-4.504196642	-1.718914189	-0.157259144
-4.438392312	-4.786660395	-2.66659162	-5.73382841	-2.978836385	-3.437817243	1.572327872	-1.743588162	-3.895142383	-1.987736235	-2.747733828	-4.622292122	-1.841415953	-0.153363392
-4.118258033	-3.879204211	-2.507370471	-5.812332485	-2.392011608	-3.065605069	1.480115885	-2.597215786	-3.762290424	-1.090247264	-2.659273675	-4.771760273	-1.981716626	-0.288021679
-3.916203084	-4.494039894	-2.102258236	-5.631123119	-1.83335274	-2.834917882	1.256642949	-2.226284026	-2.581121809	-0.941999068	-2.392467418	-4.700247048	-1.589190821	-0.209576453
-4.397620388	-4.513987782	-2.769331929	-5.264007393	-3.009741169	-3.115283784	1.468287822	-2.017262784	-4.02283341	-1.757910062	-2.83337974	-4.183274089	-1.856749635	-0.127618518
-4.014207249	-5.141503437	-2.146363079	-5.963806973	-2.152647764	-2.857767997	1.013334411	-2.090019043	-2.723383768	-0.907999239	-2.284386161	-4.885040871	-1.686139309	-0.141279284
-4.698588952	-4.123935087	-2.834539421	-6.008061975	-3.11446668	-3.318543425	0.935444789	-2.81295829	-4.308207131	-1.994265086	-3.178261465	-4.895570099	-2.122643795	-0.462675962
-4.111966648	-4.765797279	-2.474595288	-6.162953379	-2.500919099	-3.530675827	0.948851899	-2.630631734	-3.677402078	-1.431068902	-2.815925466	-4.967760058	-1.878097866	-0.465372925
-4.46736437	-5.500782299	-2.47034416	-5.707394457	-3.441201549	-3.460706339	1.354879996	-1.479974538	-3.414298542	-1.994026056	-2.738877004	-4.641020309	-1.828337587	-0.107153399
-3.897047516	-3.839806411	-2.408529228	-5.644730986	-2.692343775	-2.985908882	1.254882433	-2.498190786	-3.816282931	-1.477515762	-2.85098361	-4.936667885	-1.870681152	-0.253548502
-3.894154226	-4.64807378	-2.443013178	-5.89036084	-2.338844684	-2.813317284	1.312175716	-2.934693344	-3.757361058	-1.164485019	-2.979684349	-5.005108247	-1.8889084	-0.225325725
-4.880829714	-5.16913066	-2.986374693	-5.93447121	-3.508728368	-3.233515437	0.645227765	-3.129572529	-1.686470943	-2.244934074	-3.108960784	-5.036781411	-2.172617484	-1.19320996
-4.232223205	-4.254528328	-3.045037245	-4.597860758	-4.192779067	-2.653185976	1.484197104	-2.117342271	-3.76270352	-2.975041589	-3.1394977	-3.742928674	-1.83936126	-0.616505517
-4.506686828	-2.971437081	-3.022259093	-5.465040944	-4.427704107	-3.413211038	1.872506793	-2.399431719	-3.738981046	-3.139646191	-2.980260151	-4.322729084	-1.617289558	0.244284104
-4.812195886	-4.819774968	-3.052050007	-5.743614107	-3.905846228	-3.64495157	1.652949041	-2.422538422	-4.553828673	-2.52636612	-2.917615832	-4.572012843	-2.152636107	-0.220373024
-4.134334417	-4.253578292	-2.602629575	-5.414311342	-4.802738072	-3.015376842	2.048741597	-1.400867549	-3.253572981	-3.735637785	-2.702660584	-4.082677345	-1.392999511	0.338251774
-5.798918001	-4.60621321	-3.116787577	-6.701589114	-5.329469114	-3.255003386	2.150271037	-1.86607292	-3.174359876	-3.265832517	-3.750090206	-3.796179506	-2.470178684	-0.136397577
-4.206956137	-4.177715727	-2.927921679	-5.708837659	-4.796079371	-3.149787517	2.042774461	-2.030122555	-4.205319587	-3.022134187	-3.17594623	-3.557392552	-2.213588873	-0.114117274
-4.253313278	-4.416982876	-2.746446611	-5.834418935	-3.858199011	-3.364042969	1.918538656	-2.496423933	-3.928343413	-2.654590272	-2.937953559	-4.604656016	-1.94544518	-0.052697719
-4.473709817	-4.35472193	-2.887341075	-4.754421379	-4.820753765	-3.474945288	1.802790024	-1.407045476	-3.192748788	-3.50469368	-3.303773093	-3.472616922	-1.952244044	-0.187998602
-4.187707728	-5.170328785	-2.774460362	-5.801843926	-4.097462843	-2.620158522	2.081898951	-1.090395076	-2.710641278	-2.763834794	-2.628562069	-4.075821646	-1.661655676	-0.196654541
-4.35891649	-3.578700862	-3.298980912	-5.804142492	-4.238757295	-2.649267894	2.042108269	-3.612807383	-1.887943436	-2.960262257	-3.480283249	-3.933581998	-2.396977387	0.471628014
-4.935517231	-3.451632662	-3.147819566	-5.699441328	-4.462798749	-2.459557068	1.908770481	-4.061367663	-2.681867741	-2.588074121	-3.484717585	-3.94630767	-2.550081976	0.056733067
-3.642368187	-5.813928552	-1.505765333	-5.952584772	-2.937935878	-1.193640868	1.999937426	-1.776954454	-0.855740519	-0.890463133	-1.883064354	-4.936270888	-1.218097964	0.034565887
-4.868330965	-3.992927125	-2.997412263	-5.614480121	-4.133270114	-3.125572	2.116566381	-2.999429555	-3.055593892	-3.027514899	-3.474630014	-4.276973235	-2.175399058	-0.049405447
-4.719603976	-4.80304437	-3.030045581	-6.144092566	-4.71476465	-3.233395504	2.05295744	-2.218542124	-3.428407202	-3.151931318	-3.083558663	-4.597967112	-2.100220535	-0.157236791
-4.223121043	-5.834358489	-2.406341351	-5.547494242	-3.777791963	-2.867917549	1.993564148	-2.100137596	-2.832671462	-2.336082981	-2.776010319	-4.590449429	-1.905049711	-0.412619247

-4.403185733	-3.881463045	-3.030809451	-5.867498008	-3.678584385	-3.268903184	1.81199085	-3.05461269	-3.50840062	-2.413165508	-3.022518197	-4.333995574	-2.165251336	-0.340970548
-4.629193413	-3.670163158	-3.038454818	-4.719017303	-4.505567481	-2.983827001	2.416505556	-1.735983363	-2.996066658	-3.302489778	-2.988704417	-3.10708344	-1.820806389	-0.106546357
-4.051028385	-4.346336214	-2.742438313	-4.773581112	-4.084268464	-3.577412525	1.965126761	-1.451142297	-4.034771492	-2.707917464	-2.54565271	-3.122424688	-1.647598951	-0.185564556
-3.4984977	-4.999830521	-1.713132129	-5.554640823	-3.267387247	-1.402483063	1.416097348	-2.353555919	-1.144326867	-1.115828706	-2.134738124	-4.404108316	-1.47099967	-0.174197199
-4.304781488	-4.918171165	-2.076705396	-5.98257301	-3.340103533	-2.376570357	2.132287857	-1.826028729	-1.808980297	-1.920609315	-2.937923373	-5.119472742	-1.912027162	-0.306450836
-3.957865627	-2.804981258	-3.156694814	-3.821258088	-4.167615745	-3.016546286	1.67207721	-2.531440352	-3.510534969	-2.86110265	-3.586407212	-2.133603207	-2.269001525	-0.077973082
-4.730650157	-4.1410358	-2.760383257	-3.742628941	-4.412226665	-3.345961509	1.988125626	-1.630752007	-3.455459416	-3.189121142	-2.915159667	-1.745503569	-1.705547767	-0.185114334
-4.43672771	-5.280831896	-2.229935517	-6.096776438	-3.617647856	-2.623917124	1.649555003	-2.032094707	-1.709833871	-2.052159622	-2.993825942	-4.580856121	-1.934756415	-0.466724142
-4.590449243	-3.234064923	-2.863084842	-5.172716413	-3.936601255	-2.982825954	2.163217427	-2.712973265	-3.484720534	-2.94399666	-3.347345208	-3.46477916	-1.674258624	-0.090032003
-4.508021316	-3.33664079	-3.039033492	-5.677187205	-3.822642763	-3.043482032	2.065036439	-3.172337455	-3.018946616	-2.744317646	-3.224048858	-4.350883185	-1.893085662	-0.00912654
-4.920699185	-3.914863105	-3.544015654	-5.845528113	-4.738691108	-3.30753548	1.971490797	-3.13813053	-2.425579326	-3.087486042	-3.73658507	-3.692970795	-2.597086479	0.040381554
-4.527426525	-4.432770098	-3.201748108	-5.375527846	-4.299070222	-2.784112361	1.879028284	-2.577339023	-2.678932524	-3.032020244	-3.287699729	-4.011012997	-2.068518198	-0.221898078
-4.51475912	-5.324739426	-2.923330916	-5.68202579	-4.615337132	-3.626255489	1.596581662	-1.500342284	-2.937917763	-3.240468132	-3.074424579	-4.419221514	-2.337418767	-0.287358513
-4.867202271	-3.944544873	-3.390615918	-5.057513347	-4.440823932	-3.183410307	1.747738721	-2.932155667	-2.760562627	-3.342671149	-3.583458877	-3.690252994	-2.573214663	-0.310373649
-5.023726158	-3.902739281	-3.462759884	-4.534295117	-4.680864459	-3.4410726	1.545678574	-2.523296379	-4.728344322	-3.207797594	-3.637175526	-3.025082727	-2.168705268	-0.660215511
-4.958368212	-4.370028412	-3.020435984	-4.479988783	-4.474579409	-3.65493778	1.8355613	-1.619650018	-4.005140167	-3.246554104	-3.009003337	-2.882720458	-1.916133987	-0.335475718
-4.495746383	-4.522007884	-3.010069134	-5.844428934	-4.295125273	-3.095750588	2.21855312	-2.179131085	-3.600998962	-2.809209687	-3.336189329	-3.911298467	-2.133175771	-0.178233436
-3.919242127	-5.169856196	-2.199157587	-5.575388611	-4.070905424	-2.822169194	1.518803246	-1.625143015	-2.168849735	-2.428107301	-2.716161805	-4.658461304	-1.853057574	-0.234359179
-4.27965824	-4.311952847	-2.47344446	-5.1875071	-3.876781314	-2.862536137	2.000830599	-2.676193978	-2.898102213	-2.491995715	-2.941467607	-4.262893336	-1.485471611	-0.533656695
-4.829897566	-3.952230467	-3.463080775	-5.940183203	-3.895239712	-2.931755075	1.720558178	-3.626574952	-3.464078981	-2.962267319	-3.615975605	-4.325323075	-2.477792859	0.034106578
-4.21013019	-4.598548804	-2.482003536	-5.680242768	-3.349167245	-2.486535945	1.821448569	-1.868633517	-2.143807959	-2.093762155	-2.700312691	-4.52399779	-1.701671666	0.149654344
-4.708443421	-3.916070021	-3.210229434	-4.063731788	-2.989936993	-3.440031058	0.53213387	-2.821056301	-3.864684721	-1.90516023	-3.326373226	-3.25176125	-2.127843783	-0.623245716
-4.915496667	-4.085387195	-3.157412764	-6.628887874	-3.076707961	-2.987947258	1.123467935	-3.285658136	-3.404484478	-1.967179747	-3.189195133	-5.538042914	-2.409299433	-0.548468212
-5.508908436	-3.834626787	-3.358711546	-5.506890693	-4.395109562	-3.665607248	1.395059036	-2.291445529	-4.318195471	-3.402970903	-3.308892499	-4.156916931	-2.101660266	-0.623648384
-4.525597843	-3.637169186	-3.045049107	-5.871574614	-3.326848828	-3.067808467	1.319825524	-2.651996557	-4.411419856	-2.371205429	-3.069939954	-4.599154277	-2.127164163	-0.652839619
-4.88388715	-4.032054597	-3.031272107	-4.018840046	-3.033116692	-3.651968584	1.02279829	-2.327163204	-4.708500185	-1.848319452	-3.494672322	-2.61139699	-2.136941115	-0.55643434
-4.575349203	-3.634496886	-3.036436916	-3.222636702	-3.250714406	-3.524757739	0.770993838	-1.9105262	-3.944249286	-2.505895675	-3.306370101	-2.108855735	-1.856000401	-0.586957383
-4.738477645	-4.181252713	-2.967369632	-3.828850497	-3.529130231	-3.79210868	0.724911834	-1.322120981	-3.439242079	-2.8711146	-3.257843481	-2.996981191	-1.839601835	-0.321179581
-4.34305256	-3.777531463	-3.207433776	-4.04084382	-3.059570761	-3.503025105	0.55114994	-2.489798454	-4.371874928	-2.13706437	-3.111896731	-3.030290065	-1.957431161	-0.40192431
-4.586826201	-3.772700491	-2.894793347	-3.945496962	-2.771910883	-3.361782357	0.753440384	-2.72997687	-4.262684313	-1.843498275	-3.20713194	-2.913450247	-2.203987163	-0.417697298
-4.700030667	-3.330887595	-3.26737189	-3.249870482	-3.26855407	-3.377239448	0.717518023	-2.358874046	-4.320747495	-2.440040238	-3.109807104	-2.343947016	-1.996061584	-0.350971734
-4.44062158	-3.757043565	-2.87896668	-3.46664153	-3.486814256	-3.871101642	0.798104443	-1.467678761	-3.646040651	-2.264854502	-2.993959854	-2.293111254	-1.763293892	-0.453197845
-4.762913988	-3.748554595	-3.209025406	-3.415687769	-2.976457228	-3.432789178	0.746840434	-2.737524857	-3.848703256	-2.05104054	-3.152920429	-2.335829109	-2.046617195	-0.434553358
-4.711600022	-3.501948588	-3.077341294	-3.404925575	-2.882203991	-3.321000663	0.541350748	-2.449872581	-4.319204688	-1.776130624	-2.98890242	-3.017907974	-1.934533863	-0.438706919
-4.331367964	-4.220227051	-2.987182027	-5.483069839	-4.316069258	-3.665475298	1.603298356	-2.66995897	-2.988357753	-3.1150556	-3.262727541	-4.556793151	-2.323252506	-0.124262938
-4.072096095	-3.705478317	-2.586674786	-5.338920658	-3.829323942	-3.167359447	2.147407829	-2.09304039	-3.70428865	-2.413583392	-3.071112032	-3.994202377	-1.805415559	0.244686012
-4.225569999	-3.183332385	-3.088789351	-4.79812035	-4.237641775	-2.862310869	2.03315265	-3.168483469	-2.667464397	-2.93618823	-3.538537948	-3.207135982	-2.149829251	-0.045503894
-4.609852541	-3.881517996	-2.944312307	-4.152192088	-4.149270362	-2.699428019	2.085856449	-2.627430685	-3.358849759	-2.725269073	-3.541901779	-2.529096989	-2.224925778	-0.438956096
-4.553148261	-4.332844148	-3.106765474	-5.281458548	-4.670745918	-3.021434062	1.917661937	-1.99150696	-3.966893975	-3.096720053	-3.323746541	-3.812544672	-2.082808829	-0.428477026
-4.609696256	-4.234991325	-2.590989644	-4.635063833	-3.970760955	-3.278189429	3.007546533	-1.848289453	-3.664374802	-2.492025721	-3.047095723	-3.449588939	-1.938970016	-0.329548826
-4.578335421	-3.766174482	-3.060257113	-5.492908499	-4.319528666	-2.698431889	2.032689524	-2.540899578	-2.694600556	-3.052107441	-3.59029914	-3.868990242	-2.189037106	-0.381867187
-4.550325149	-3.671719558	-3.273831798	-5.830909364	-4.052418402	-2.669006835	1.809348113	-3.361056888	-2.600580273	-2.821905962	-3.249315165	-4.744918573	-2.262237426	-0.272589241
-4.863648321	-3.965148149	-3.460853806	-5.841595043	-4.196381318	-2.646283211	1.752792453	-3.48713016	-1.997107505	-2.844256726	-3.334580174	-4.558020309	-2.435012717	-0.124984499
-5.074651147	-5.02529989	-2.886116053	-5.716416903	-4.461055259	-3.19783667	1.166430925	-2.697281949	-2.364595197	-2.777050191	-3.456366393	-4.338641867	-2.212873681	-0.447578511
-4.764194402	-4.727756228	-3.227539882	-5.754327518	-4.64488716	-3.84580831	1.534584076	-1.605206859	-3.112896275	-3.260960298	-3.223768181	-4.427593596	-1.994132196	-0.466432694
-4.691798329	-4.502984336	-3.354516869	-5.650423356	-4.679448003	-3.241324515	1.854784934	-2.229295383	-3.604623844	-3.260949186	-3.524417372	-3.623050936	-2.668637241	-0.295058003
-4.922960869	-4.183243081	-2.868081199	-3.860518044	-4.518947447	-3.498637236	2.135609497	-1.256529039	-4.229676499	-2.997084924	-3.241378158	-2.645744274	-1.888464621	-0.259481784
-4.322258875	-3.686133782	-3.010315255	-6.339718352	-4.640048485	-2.209956878	2.013878338	-3.254691019	-2.730324505	-2.700079234	-3.432343493	-4.656129012	-2.595351168	-0.288060753
-4.603933386	-3.513429442	-2.954235692	-4.566266435	-3.804010158	-2.699697338	2.096170864	-2.461049207	-2.930840664	-2.821239566	-3.484485363	-3.185910362	-1.988315969	-0.351816224

-4.556272703	-3.401727566	-2.945512235	-3.193318618	-3.208108782	-3.332703849	0.683167105	-1.641613647	-4.445724002	-2.521158538	-2.98653549	-2.164334514	-1.682571301	-0.240159499
-4.243867294	-3.097544837	-3.092331858	-2.020597051	-3.149945211	-3.304103512	0.51671664	-2.393649947	-4.412181238	-2.292986629	-3.127473847	-1.712710614	-1.894476366	-0.474278666
-4.71491495	-3.3150832	-3.136440594	-3.429995331	-3.238791435	-3.275209197	0.739365527	-2.652705999	-4.472935817	-2.430239914	-3.185214694	-2.254709791	-1.831767807	-0.286083416
-4.336192801	-3.729306768	-3.13173922	-3.469843846	-3.255854817	-3.581621087	0.661918843	-2.051910304	-4.41340708	-2.306774955	-3.197259534	-2.548551561	-2.103432249	-0.259683652
-4.829963805	-3.774923245	-3.182549448	-4.149759222	-2.959669548	-3.442876448	0.442795821	-2.706197293	-4.530338646	-1.971772573	-3.323331135	-3.169670659	-1.97699645	-0.563912037

hsa-miR-6722-5	hsa-miR-6724-5	hsa-miR-6726-3	hsa-miR-6726-5	hsa-miR-6727-3	hsa-miR-6728-3	hsa-miR-6729-3	hsa-miR-6730-3	hsa-miR-6731-5	hsa-miR-6732-3	hsa-miR-6732-5	hsa-miR-6734-3	hsa-miR-6735-5	hsa-miR-6737-5
-4.702021181	-0.389397868	-4.059584362	0.725227621	-3.724720663	-4.171830045	-3.375498932	-3.913511353	-3.134264548	-3.676603984	-0.730161653	-4.710598992	-3.665696308	-2.187785153
-4.316421536	0.265304174	-4.351299069	0.833605165	-3.949285566	-4.340419192	-3.806116636	-4.163889891	-4.173331118	-3.933033718	-0.303472808	-5.244290504	-3.763786192	-2.366109453
-4.541303906	0.202920733	-4.222529614	0.580199333	-4.000149405	-4.291045781	-3.495913069	-4.141930309	-3.041055427	-3.910341284	-0.557982808	-5.210153983	-3.903924652	-2.233328152
-4.604107652	0.118599816	-4.146168327	0.583737608	-3.924027215	-4.291764504	-3.533851729	-4.138245274	-3.125692339	-3.848319988	-0.42963642	-5.054882938	-3.810240105	-2.482580937
-4.686812683	-0.112389686	-3.956539761	0.796620634	-3.528540679	-3.844393189	-2.979345173	-3.788821702	-3.33265991	-3.612763603	-0.678627396	-4.636621464	-3.529687662	-2.103495046
-4.253556142	0.194164101	-4.218392276	0.763182065	-3.782522379	-4.229094609	-3.400813773	-4.025957505	-3.313304011	-3.736603781	-0.252344385	-5.295252374	-3.827302408	-2.350679759
-4.279105295	0.110644769	-3.861216057	0.828554227	-3.622796937	-4.062851231	-3.258660946	-3.796450671	-3.570255101	-3.604851015	-0.416246191	-4.965803894	-3.72068392	-2.166488956
-4.350400324	0.252733784	-4.051109548	0.664195353	-4.001227819	-4.468861717	-3.69900595	-4.194210732	-3.541129039	-3.861886809	-0.249565932	-5.063651884	-3.912618672	-2.322343442
-4.54247909	0.209434592	-4.295081385	0.607646692	-3.930581755	-4.438656632	-3.63429625	-4.274061284	-3.565923181	-3.979970719	-0.372804902	-5.293724221	-3.862606362	-2.371471923
-4.362980667	0.16848533	-4.218327749	0.453221154	-3.960052966	-4.303513824	-3.670028891	-4.087557	-3.57087533	-3.794541058	-0.172485138	-5.283807674	-3.815917117	-2.298915879
-4.525890706	0.330230682	-4.33263673	0.481605838	-4.240917357	-4.801570749	-3.469351874	-4.461236657	-3.724794028	-4.127538184	-0.153633614	-5.343916689	-3.814512424	-2.399756994
-4.647753176	-0.068244353	-4.753609008	0.199337619	-4.356329555	-4.700407878	-3.909506561	-4.665392782	-3.700057058	-4.355738076	-0.650674914	-5.55751244	-3.785213259	-2.573718774
-4.972482772	-0.323876709	-4.739277647	0.177102981	-4.363278761	-4.819379869	-3.873573497	-4.662288337	-4.00211674	-4.521803769	-0.692615643	-5.820926129	-4.068177593	-2.744825578
-4.655946726	0.071688183	-4.398596207	0.462209308	-4.239453913	-4.669744758	-3.547715194	-4.531109587	-3.194753307	-4.140229004	-0.44170339	-5.512623249	-4.243392236	-2.35286867
-4.497385144	0.040065147	-4.633246866	0.540179727	-4.351954287	-4.878542405	-3.721293709	-4.619706142	-4.339758551	-4.348473781	-0.428672953	-5.54021086	-3.818972266	-2.383676001
-4.630481428	0.417996362	-4.454920073	0.454525265	-4.36426103	-4.7828257	-3.791562478	-4.570708192	-3.906784634	-4.329799455	-0.373182651	-5.692317022	-4.007142296	-2.529388191
-4.325352868	0.272633814	-4.373019049	0.510367384	-4.289715755	-4.755272355	-3.867543974	-4.74232352	-4.050106673	-4.436344746	-0.474244994	-5.516285208	-3.906013199	-2.503325698
-4.514659956	0.408520106	-4.465856557	0.466206217	-4.819989621	-5.500704368	-3.994619238	-4.934553905	-4.283544104	-4.374540831	-0.48203266	-5.289901495	-4.813616606	-2.67580088
-4.75077591	-0.050668826	-4.487416875	0.382763916	-4.341229699	-4.921100269	-3.620977532	-4.584089223	-3.910905988	-4.209045246	-0.777080749	-5.197871743	-3.968439636	-2.701373731
-4.459592796	0.208398337	-4.362973974	0.438767188	-4.238351337	-4.793888459	-3.766991463	-4.677032867	-4.016760341	-4.294227091	-0.451565514	-5.305372266	-3.872797066	-2.533797639
-4.421112969	0.11982578	-4.530135436	0.658510756	-4.294448553	-4.986541003	-3.788171665	-4.892019673	-4.090750005	-4.301620793	-0.338675782	-5.588073061	-4.165595752	-2.202294527
-4.351699053	0.259189556	-4.615895409	0.734019375	-4.328377235	-4.913427228	-3.847940324	-4.681431917	-4.409185983	-4.395420741	-0.222182215	-5.892037833	-3.956365816	-2.276978156
-4.625173245	0.077065234	-4.453190941	0.51152684	-4.282858829	-4.74814139	-3.496816518	-4.554454626	-3.785604051	-4.263696362	-0.498787146	-5.433302528	-4.096858438	-2.631133479
-4.833873472	0.056785523	-4.215676459	0.591690862	-3.903825508	-4.416640808	-3.344793772	-4.221799376	-3.101015332	-3.704666729	-0.800874978	-4.822657323	-3.493240457	-2.474795425
-4.773542025	0.370344511	-4.666419582	0.527156149	-4.382577236	-4.845897157	-3.717717161	-4.896918185	-3.474852245	-4.341393263	-0.320731133	-5.741290822	-4.046890313	-2.506831809
-4.861248804	-0.001294174	-4.087314541	0.736532391	-3.954689821	-4.385691996	-3.174375188	-4.085276438	-2.415775699	-3.649998823	-0.873580561	-5.12423255	-3.480333923	-2.632129536
-4.661875634	-0.344907367	-4.823624183	0.156126432	-4.194931195	-4.634766062	-3.886219271	-4.672787353	-3.912143669	-4.515943014	-0.61203576	-6.03234443	-4.066578458	-2.562017899
-5.083746337	-0.679926024	-4.415561741	0.280984597	-4.143584633	-4.471550698	-3.514453921	-4.424053714	-3.313168552	-4.030535227	-1.052204702	-5.346579594	-3.820399621	-2.529245895
-4.699469712	0.369356538	-4.536257308	0.835127351	-4.329688147	-4.498344479	-3.755711167	-4.242871447	-4.105942093	-3.910556782	-0.652793545	-5.359535886	-4.622146223	-2.461311502
-4.474532984	0.250866919	-4.418571856	0.378756898	-3.994924675	-4.418909343	-3.629317074	-4.484620218	-3.64577524	-4.004581055	-0.403110859	-4.901361253	-4.135886817	-2.438132164
-4.517743274	0.186830326	-4.322602705	0.286399524	-4.12353043	-4.568471818	-3.83525162	-4.416844742	-3.954297353	-4.200522481	-0.422438953	-5.366098252	-3.768955208	-2.610195875
-5.565855129	-1.360182137	-5.101404499	-1.02316064	-4.966091189	-5.146901842	-4.296971647	-5.230398053	-4.018065366	-4.715875755	-1.681719998	-5.847077373	-4.458367279	-3.170123018
-4.722766441	0.615656242	-4.736797326	-0.264458774	-4.389834755	-4.870467283	-4.078679181	-4.623597346	-4.551490809	-4.3277077	0.010020227	-5.632105896	-3.7829029	-2.331156985
-4.514811912	1.043652093	-4.695280464	0.549605868	-4.19316605	-4.734625258	-3.955268781	-4.552646198	-4.7290166	-4.265209457	0.258689039	-5.780517478	-3.658331804	-2.345492789
-5.246686432	0.433879563	-4.769924695	0.277461091	-4.424137588	-5.264873142	-3.792757115	-5.01219023	-4.135313116	-4.623150031	-0.614200248	-5.76796456	-4.973010146	-2.79209259
-4.037370156	0.814383727	-4.237511687	1.27086803	-3.954601372	-4.404324672	-3.495373322	-4.183977727	-4.453200279	-3.963462938	-0.119233065	-5.446137059	-3.768484918	-1.825595304
-5.36205293	0.984088429	-6.006324537	0.491002844	-4.516190128	-5.807100102	-3.586347082	-5.025341093	-7.484485577	-5.601578907	-0.231632561	-7.442147078	-4.365584814	-2.566118614
-4.570085389	0.746107519	-4.371561708	0.470687133	-4.090536484	-4.78943746	-3.585459504	-4.787792801	-4.794508537	-4.265343644	-0.008501926	-5.252649338	-4.91205914	-2.695320414
-5.242198663	0.689602053	-4.27141159	0.633314966	-4.082856435	-4.741500779	-3.522088405	-4.341397894	-4.973714018	-4.103597347	-0.305448048	-5.181358523	-4.425435214	-1.984611426
-4.868463478	0.478673004	-4.413006536	0.86208532	-4.228081067	-4.936743795	-3.751448703	-4.717808088	-5.111332546	-4.400064756	-0.445249638	-5.815747885	-4.284354714	-2.393899091
-4.712812668	0.443165934	-4.389310814	1.029087396	-3.873307953	-4.724131808	-3.571805399	-4.506178739	-4.274221584	-4.070599394	-0.536458562	-5.073099794	-4.357394158	-2.261314414
-5.187468377	0.871608947	-4.302180887	0.82997254	-4.403101555	-4.716116161	-3.929086978	-4.628575253	-5.018582118	-4.411043269	-0.226617175	-5.222664236	-4.144213672	-3.058553659
-5.039620551	0.863952722	-4.798360912	0.372712718	-4.532810545	-5.571148105	-4.105996248	-5.043054328	-4.645538104	-4.93546802	-0.703559618	-5.97006838	-3.798357852	-3.163889374
-5.503605441	-0.159910847	-4.226605509	0.215882435	-3.676954356	-4.148071117	-2.484670394	-3.870856034	-3.026044273	-3.580100086	-0.558894209	-4.818489117	-3.815906154	-1.827954448
-5.473050805	1.040642582	-4.563540478	0.378700888	-4.404447424	-5.117794056	-3.97987754	-4.72571443	-4.397111104	-4.596334244	-0.114098747	-6.160164896	-4.241473506	-2.747592271
-4.792397474	0.781093805	-4.727552165	0.205224983	-4.320501118	-5.189232112	-3.781602947	-4.844429073	-4.40674652	-4.447471793	-0.158234294	-5.716508087	-4.856892701	-2.584000587
-5.151529834	0.779702846	-4.314739119	0.536814774	-4.122501266	-4.631390723	-3.427255873	-4.302652477	-4.103278539	-3.998249881	-0.618112808	-5.194416209	-4.305890659	-2.340770015

-5.128127808	0.753733272	-4.511936081	0.128929666	-4.377985153	-5.120608996	-3.952593453	-4.785643586	-4.33523348	-4.421363781	-0.247868657	-5.459581389	-4.329201746	-2.784489611
-4.693908562	0.977554266	-4.849898322	0.202192751	-4.252235867	-5.130384178	-3.616896998	-4.983829491	-5.213919101	-4.386865877	0.053216652	-5.390830196	-3.792345247	-2.043268739
-4.491466651	0.778072738	-4.296194012	0.397838553	-4.178183383	-5.000487818	-3.753801921	-4.613102034	-4.459936867	-4.222335361	0.174817103	-5.202354741	-4.060041478	-1.960283961
-5.28919713	-0.092698422	-3.955575246	0.631156855	-4.12291615	-4.37446329	-2.97181503	-4.044320574	-3.55192033	-3.732868815	-0.433939629	-4.486479227	-3.585148527	-2.204798374
-5.555969922	0.075665272	-4.507713099	0.503206998	-4.084464142	-4.55629741	-3.261375065	-4.380024361	-3.937059655	-4.072497915	-1.222381524	-5.38184103	-3.878989088	-2.687493255
-5.199692667	0.700440113	-4.558418715	0.455509502	-4.353506117	-5.544007608	-3.827968434	-4.546519065	-4.232350797	-4.601670958	-0.101564156	-5.672608681	-3.873995107	-2.541694574
-4.295919802	0.704683916	-4.842268586	0.161354274	-4.137842018	-5.14899472	-3.278427383	-4.373690467	-4.901230295	-4.179773436	0.144558628	-5.070697263	-3.969325398	-1.91571416
-5.243544558	0.25452748	-4.609411915	0.139112964	-4.110531655	-4.534168287	-3.243607562	-4.494444558	-3.863597932	-4.088721606	-1.173168214	-5.498296977	-4.047187792	-2.576504839
-5.327964437	1.213210618	-4.694068722	0.131171231	-4.229108052	-4.691285425	-3.661605261	-4.687533707	-5.06707692	-4.223790088	0.270652587	-6.076326842	-3.879896615	-2.615714758
-5.130698713	1.025505705	-4.534725668	0.347542443	-4.323855513	-4.893875281	-3.918800626	-4.684414563	-4.4439814	-4.436681087	-0.114970424	-5.501257313	-4.218887661	-2.28885416
-5.199452824	0.462480388	-5.149701282	-0.139006199	-4.329542128	-5.176356142	-3.805258521	-4.944048316	-3.812173792	-4.723134692	-0.647935804	-5.832374925	-4.216957272	-2.96397835
-4.648300023	0.742931143	-4.711392273	0.070803406	-4.258788753	-5.014181941	-3.922203002	-4.684959818	-4.077036487	-4.574293293	-0.519542248	-5.552298005	-3.960978664	-2.667148964
-5.18508713	0.267123618	-4.675484434	0.908876137	-4.592915	-5.231180434	-3.913336511	-4.882150529	-4.831095101	-4.54916252	-1.164219154	-5.545049284	-4.582554438	-2.748325496
-5.477267163	0.732430289	-4.752614595	-0.056891051	-4.321407447	-5.459494006	-4.060154623	-5.04938694	-4.651080092	-4.778762202	-0.391503276	-5.520668427	-4.514296312	-2.957065384
-6.698244923	0.50433395	-5.235894501	-0.418427785	-5.036049438	-6.476613891	-4.030084649	-5.584115894	-5.193101758	-4.795268657	-0.227996389	-5.32671995	-4.773350019	-2.819661202
-5.099487265	0.808144451	-4.541375787	0.555350555	-4.247494114	-5.53125426	-3.515240619	-5.048354975	-4.581580844	-4.402981225	-0.002963995	-5.963477642	-4.391661156	-2.325922106
-4.772921108	0.771021867	-4.478254785	0.53141961	-4.338255477	-4.92118919	-3.764711755	-4.836337535	-4.872530899	-4.447847778	-0.357984199	-5.259564732	-4.510012245	-2.327800894
-5.152446337	0.122486114	-4.091461656	1.227522813	-3.787849437	-4.273346293	-3.240042807	-3.992077976	-4.322477252	-3.687715237	-1.019746555	-4.795827061	-4.379238697	-2.413269468
-5.162487261	1.265790544	-4.313731285	-0.019494871	-4.12188217	-4.630499366	-3.526211309	-4.353008828	-4.157129261	-3.916045654	-0.286771176	-5.253825198	-4.248361908	-2.467505555
-3.752927537	0.382057927	-4.898851503	0.401556451	-4.619805392	-5.604535727	-4.176704176	-5.12717874	-4.68065073	-4.859575668	-0.309952424	-5.994777734	-4.290533407	-2.964635985
-4.599994574	0.367443688	-4.433787444	1.076514487	-4.034925562	-4.645976666	-3.77911531	-4.544479783	-3.841834453	-4.162012403	-0.475381829	-5.631689513	-3.845279556	-2.36247436
-4.383458338	0.208591996	-5.033179144	-0.405309517	-4.621823987	-4.988450527	-4.406655375	-4.910411958	-4.170187342	-4.73963655	-0.4295649	-5.955889263	-3.705996737	-2.268026904
-5.414095519	-0.060000769	-5.112106408	-0.075521537	-4.901661695	-5.298275742	-4.377044196	-5.189375008	-4.685716016	-4.826759135	-0.822776638	-5.860511234	-4.238706428	-2.904754501
-4.499007688	0.308301514	-5.757297411	-0.426092006	-5.18789736	-5.990434087	-4.966158069	-5.777478532	-5.026089688	-5.685927023	0.122651517	-8.197532955	-3.971775728	-2.350831962
-4.460032946	-0.241404088	-5.107299837	-0.346110777	-4.466947876	-5.045902908	-4.062400545	-5.041048984	-3.914801777	-4.737747277	-0.400598007	-6.264088954	-3.965744611	-2.541627972
-4.456645818	0.335770617	-5.107123882	0.113733893	-4.508213135	-5.629266061	-3.870300251	-5.030860704	-4.691509594	-4.751728596	0.051940965	-6.077452946	-3.937013306	-1.834187228
-4.374107522	-0.004877647	-5.12703655	0.889490143	-4.711252829	-4.877673202	-4.132990915	-4.782092277	-3.780728667	-4.855569328	-0.743344026	-5.850029077	-3.40145732	-1.643653754
-4.063832351	0.166382994	-4.939957377	1.377207908	-4.314607884	-4.720012612	-4.071203027	-4.795289623	-4.034669274	-4.701731736	-0.777688898	-5.933170717	-3.421477676	-1.892489849
-4.402439581	0.084259749	-5.049345559	0.194224197	-4.558150916	-4.88568567	-4.272826269	-4.961254664	-3.996980592	-4.745862107	-0.322580733	-6.078022698	-3.626280129	-2.023960814
-4.285753772	0.274007565	-4.739508552	0.19825669	-4.42845579	-4.843821623	-3.982265585	-4.923102934	-4.298852905	-4.633846869	-0.236686446	-5.689904576	-4.06133902	-2.082995867
-4.279741987	0.227179286	-5.120642494	0.011470873	-4.993313044	-5.205619862	-4.59187404	-5.132885952	-3.995736934	-4.956480788	-0.215128942	-5.862572791	-3.360597647	-1.581254888
-4.12018805	0.025868591	-4.798413236	0.648225477	-4.52353103	-4.955920746	-3.958773983	-4.783125223	-4.295364857	-4.570271511	-0.368131221	-5.856982316	-3.938214835	-1.676588374
-4.104244326	0.18374145	-5.161334633	-0.181138568	-4.505192468	-5.058280706	-4.259954757	-4.8251304	-3.712834565	-4.715163971	-0.428154297	-6.029461505	-3.367227756	-1.904469543
-4.228032394	0.118143137	-4.917170876	0.027755531	-4.501892705	-5.006891041	-4.2947393	-4.914275514	-3.813335931	-4.643002709	-0.277876259	-5.567593334	-3.376396035	-1.71682444
-5.140091672	0.175090211	-4.554415361	0.153933714	-4.461659309	-4.961686433	-4.084447949	-4.680073216	-4.524065468	-4.463151513	-0.630803822	-5.559467143	-4.547840301	-2.577849814
-4.300086813	1.11472831	-4.039181291	0.816975342	-4.236672762	-4.899694186	-3.398661542	-4.356092044	-4.633561788	-4.215885046	0.158159091	-4.897864797	-4.345834804	-2.271481049
-4.186999271	0.948616787	-4.611286555	0.231004033	-4.432501445	-4.788183179	-3.677496787	-4.620400709	-4.523603328	-4.566129105	-0.121735269	-5.171746022	-4.116872208	-2.687308667
-4.760649077	0.776867883	-4.731760692	0.014256023	-4.335724444	-5.263208966	-3.723908185	-4.707108328	-4.851095179	-4.520499042	-0.125202897	-5.498138226	-4.091943228	-2.556764036
-4.993028438	0.891509292	-4.311428178	-0.024059546	-4.543702608	-5.032741846	-3.790364736	-4.678440073	-5.35160731	-4.371888778	-0.147406493	-5.723144433	-4.453300675	-2.545346552
-4.801600101	0.692113774	-4.613270529	1.071620133	-4.127218006	-4.818059673	-3.154043813	-4.66138008	-4.192206733	-4.132097573	-0.553559705	-5.766565537	-3.966295062	-2.455376121
-4.855859074	0.773234166	-4.678368389	0.030670435	-4.21485606	-5.122651019	-3.805882657	-4.726911194	-4.682460426	-4.437881577	-0.163533159	-5.568636104	-4.278013027	-2.662581602
-5.227133996	0.635221742	-4.650852867	0.055475962	-4.427254191	-5.102505545	-4.105575677	-4.910800959	-4.574189282	-4.525777139	-0.453348586	-5.862913518	-4.046866906	-2.884946788
-5.396353766	0.944061737	-4.706463703	0.143557371	-4.514648397	-5.295052082	-4.10136018	-5.098280365	-3.965921144	-4.790397981	-0.795917274	-5.974644506	-4.071299576	-2.859516256
-5.491773815	0.181132163	-4.808824814	0.275963132	-4.687406762	-5.075187135	-3.887925026	-5.039423919	-4.485782952	-4.726869488	-1.211996621	-6.282377734	-4.299424855	-3.370158835
-5.330860129	0.171470387	-5.038652794	0.312640554	-4.814361034	-5.743354949	-4.327481045	-5.320274324	-4.852914014	-4.937332012	-0.93250173	-6.204168705	-4.502784276	-2.761668139
-5.6845408	0.839483286	-4.817852825	0.15807269	-4.488318191	-5.556279236	-4.073766455	-4.992109469	-4.751025777	-4.996695112	-0.418804113	-5.68716776	-4.666562066	-2.959450507
-4.86146156	0.874100971	-4.510294737	0.381814158	-4.593729604	-5.455995589	-3.190387449	-4.78398816	-5.444199279	-4.968704655	0.20054132	-5.585061325	-4.212804731	-2.165980853
-5.35058759	0.828017912	-4.594387428	0.336962808	-4.369373261	-5.34305778	-4.042508116	-4.937557326	-5.365051829	-4.251783099	-0.725152473	-5.387817693	-4.446127234	-3.063428266
-4.811967642	0.668567747	-4.856716938	0.063553203	-4.335653443	-5.157576565	-3.862745916	-4.698273075	-4.779359639	-4.604976695	0.019793522	-6.160313426	-3.930637744	-2.321865037

-4.037571405	0.289733592	-4.944684544	0.437626769	-4.296283508	-4.780524353	-4.162046	-4.821261343	-3.612516762	-4.525701158	-0.203624952	-5.94794153	-3.276192639	-1.47828049
-4.130560309	0.153062953	-4.980029636	-0.173763809	-4.508040936	-4.763882193	-3.94668993	-4.641379802	-3.723193494	-4.578151558	-0.144958419	-5.226827787	-3.137005451	-1.657922169
-4.250078118	0.345742154	-5.000893221	-0.098401926	-4.474955177	-4.763933622	-4.069872256	-4.724518601	-3.75192689	-4.478337087	-0.090551556	-6.208898966	-3.318628282	-1.829944099
-4.124598915	0.225609155	-4.862121232	0.253931991	-4.539098751	-4.883023766	-4.247499347	-4.972371691	-3.810674516	-4.591365434	-0.135343437	-5.727629978	-3.513661049	-1.964521696
-4.483703847	0.080694421	-5.173214508	0.056298682	-4.568760858	-4.913511306	-4.206482746	-4.899395795	-3.99164658	-4.749767131	-0.424136994	-5.971511978	-3.636095608	-2.140618607

hsa-miR-6738-5	hsa-miR-6741-3	hsa-miR-6741-5	hsa-miR-6742-5	hsa-miR-6743-3	hsa-miR-6743-5	hsa-miR-6746-5	hsa-miR-6747-5	hsa-miR-6748-5	hsa-miR-6749-3	hsa-miR-6749-5	hsa-miR-6752-5	hsa-miR-6753-5	hsa-miR-6754-5
-2.429413026	-3.83259973	-2.378772677	-4.287614916	-3.830954894	-0.745357379	-2.861146715	-5.168023172	-5.073381582	-4.057717846	0.072460514	1.698095712	-4.506968494	-4.328909627
-2.602231884	-4.09437546	-2.420346691	-4.585492615	-3.973396319	-0.336752135	-3.001506353	-4.996092604	-5.05888032	-4.27269869	0.003155018	1.963713197	-4.323546898	-4.586171331
-2.830820301	-4.162344619	-2.366775505	-4.690231282	-3.892594672	-0.463840887	-2.894429819	-5.157375779	-5.313699372	-4.133945094	0.117192352	1.751511948	-4.220408112	-4.436749928
-2.857612058	-4.133713593	-2.181206302	-4.464748878	-3.865157496	-0.314707536	-2.855694945	-5.033995766	-5.583380105	-4.279501642	0.127971558	1.916486015	-4.230674413	-4.147399921
-2.598660493	-3.783111964	-2.104763868	-4.309145347	-3.572099659	-0.510813682	-2.711933012	-5.353311883	-5.60813133	-3.898532752	0.452302586	1.948141649	-4.094959626	-4.390725322
-2.748835385	-4.357607226	-2.481376819	-4.460372553	-3.800588944	-0.252020448	-2.900391976	-5.078737191	-5.268846354	-4.106606273	0.243207596	2.092040454	-4.314990584	-4.491840324
-2.733207411	-4.039155148	-2.048177649	-4.246176153	-3.739738075	-0.083299505	-2.674959106	-5.319979991	-5.173550168	-3.853474568	0.299799076	1.861296577	-3.978199997	-4.111223225
-2.993332714	-4.11731053	-2.404817403	-4.506456448	-3.887382203	-0.262426978	-3.00177753	-5.204788986	-5.445135398	-4.250805958	-0.0420321	2.014950944	-4.224563395	-4.38025867
-2.885935786	-4.236754103	-2.477199347	-4.640254404	-3.982746256	-0.329620511	-3.05841495	-5.600672453	-5.488150995	-4.278701272	0.045475334	1.954925148	-4.588254593	-4.505644249
-2.729931359	-4.248310354	-2.399630571	-4.339970992	-3.89720923	-0.403252133	-2.915996834	-5.056300172	-5.276714666	-4.198411436	0.130073249	2.075496284	-4.222664807	-4.20589438
-3.276735458	-4.587687489	-2.399694438	-4.391393307	-4.332181469	-0.15279662	-2.995958345	-5.76306285	-5.794112655	-4.215070467	0.047311382	2.171280641	-4.640702628	-4.223523636
-2.53311124	-4.566969345	-2.368615674	-4.89666083	-4.573515868	-0.646740035	-2.996769005	-5.002477272	-5.044817335	-4.510340995	0.029726241	1.286127132	-4.667841758	-4.807114853
-3.115457364	-4.615059999	-2.336531473	-4.941821441	-5.015713801	-0.531271101	-3.010896552	-5.557074009	-5.457310567	-4.534550464	-0.290711126	1.448514594	-4.953881086	-4.692732358
-2.808805769	-4.486521189	-2.563536086	-4.578127251	-4.262992176	-0.384725152	-2.969309251	-5.565163682	-5.829716387	-4.075632059	0.044162701	1.866752668	-4.698708905	-4.622160258
-2.976260699	-4.671924958	-2.54167655	-4.647245068	-4.34835994	-0.404282268	-3.174555282	-5.972344128	-5.830696551	-4.308085592	-0.085798619	1.940726103	-4.804488725	-4.835296678
-3.031385798	-4.67285628	-2.367728051	-4.772631594	-4.391348438	-0.331292236	-3.057483536	-5.457941589	-5.618633127	-4.378973679	-0.065323542	1.729651487	-4.629100034	-4.639823555
-2.720750816	-4.712861559	-2.683810211	-4.895089644	-4.278261248	-0.393997151	-3.15973521	-5.366267004	-5.377285365	-4.380748126	0.028839093	1.792844086	-4.699355247	-4.912762621
-3.141791919	-4.836499423	-2.691829407	-4.711431256	-5.00968736	-0.760315034	-3.195568347	-5.797352404	-5.303998934	-4.477497234	-0.122444616	1.484148136	-5.206616709	-4.644456562
-2.920467365	-4.536228333	-2.265672108	-4.57058244	-4.358082427	-0.382967299	-2.858713858	-5.694367325	-5.499119484	-4.337093477	0.07942045	1.46575378	-4.623063684	-4.474947704
-2.813732893	-4.784842834	-2.533955117	-4.667218068	-4.349034923	-0.281985155	-2.866824193	-5.563579971	-5.555307122	-4.392630074	0.045733337	1.721587209	-4.578365457	-4.632442288
-2.688270242	-4.819222214	-2.738445388	-4.621126136	-4.441540274	-0.539141033	-3.079769766	-5.573876849	-5.405232759	-4.319506104	0.028966797	1.875223472	-4.657862371	-4.903387575
-2.740342778	-4.733015669	-2.587481812	-4.921767167	-4.376779304	-0.210813487	-3.216791153	-5.832304244	-5.927131348	-4.384257463	0.048016642	1.861382188	-4.530820678	-4.94088706
-2.838585819	-4.465430635	-2.450634155	-4.595653577	-4.299510189	-0.338815342	-2.899799392	-5.557156998	-5.855941935	-4.213435658	0.109576805	1.701071241	-4.568276616	-4.597869052
-2.382234084	-4.001311663	-1.880002109	-4.343714667	-4.084660068	-0.34588959	-2.497913332	-5.130791301	-5.190426328	-4.053901455	0.582851493	1.57986916	-4.188089027	-4.440186293
-2.767639316	-4.676743446	-2.509993117	-4.824712483	-4.437155267	-0.263215173	-3.032193005	-5.398419083	-5.731273356	-4.385600434	-0.104723529	1.940090947	-4.553528243	-4.696053433
-2.241557518	-4.20104181	-2.37340205	-4.583133909	-3.956515564	-0.482504125	-2.778416533	-5.390798453	-5.598998548	-3.91927686	0.366703611	1.680413604	-4.403985087	-4.694176111
-3.080647134	-5.031107149	-2.633720002	-5.072420772	-4.488769656	-0.386641832	-3.151049183	-5.797426486	-5.68361809	-4.60965163	-0.26700458	1.658217661	-4.89201008	-4.70881316
-2.764092902	-4.351684145	-2.323752026	-4.481654719	-4.432507049	-0.671984199	-2.947312223	-5.47270104	-5.720008095	-4.225437377	0.049433617	1.304751203	-4.46131794	-4.782855317
-2.193750495	-4.251338541	-2.524214357	-4.806031375	-4.372706846	-0.84083879	-3.048146675	-5.237920594	-5.155949229	-4.285201142	0.177059164	1.721497225	-4.738852205	-4.86172483
-2.582510399	-4.399454305	-2.350792131	-4.654625545	-4.039052805	-0.26143859	-2.840306526	-5.21361232	-5.520950741	-4.261530317	0.109977244	1.797685817	-4.345288577	-4.547725977
-2.763494173	-4.374858902	-2.226845476	-4.493782119	-4.161709316	-0.276367525	-2.785661426	-5.430912858	-5.462088648	-4.399494307	0.133270955	1.696951562	-4.199172972	-4.328123264
-3.109692581	-5.172581649	-2.927543378	-4.909123822	-5.147445832	-1.147318639	-3.418280079	-5.627834304	-6.098064232	-5.057068401	-0.464866157	0.989940388	-4.98826116	-4.920187168
-2.743088019	-4.685917009	-2.193382143	-4.504614749	-4.396566787	-0.372135955	-3.226306534	-4.58693257	-4.79447748	-4.574576292	-0.267706209	1.774924435	-4.389656027	-4.932047383
-2.743737152	-4.722752695	-2.410917097	-4.661076808	-4.750452561	-0.076889663	-3.164096629	-5.348423301	-5.354356141	-4.373590528	-0.008603401	1.917547948	-3.786512942	-5.01783834
-3.176469905	-4.943335083	-2.802506381	-4.774981898	-4.8399145	-0.814745783	-3.508621204	-5.702026131	-6.099039321	-4.548617269	-0.452454369	1.454336778	-5.258185236	-5.677824361
-2.254452998	-4.506651844	-2.804772318	-4.463872069	-4.223092576	-0.274439389	-3.442768891	-4.940589473	-5.64596324	-4.06827387	0.222082228	2.246330129	-4.449596198	-5.369247265
-3.016933131	-6.0218588	-2.898324581	-5.85732217	-5.610428748	-0.207253738	-3.061938317	-7.383270051	-7.689824985	-4.824727716	-0.410491083	2.016691389	-2.810805458	-6.921887141
-3.258155995	-4.672042073	-2.615254354	-4.838772744	-4.61837075	-0.474037295	-3.327918995	-5.861422494	-5.883479583	-4.049759496	-0.40527274	2.260595267	-4.676849203	-5.692820886
-3.393457499	-4.470199397	-2.410150865	-4.556975883	-4.252044455	-0.587565174	-3.287405784	-5.633647142	-6.264865579	-4.035189877	-0.390159392	1.81544868	-4.586806836	-5.557789153
-2.828418576	-4.905633105	-2.755608897	-4.785891425	-4.679144621	-0.582132707	-3.231237143	-5.368558484	-5.723346638	-4.389905979	-0.156868808	2.180271093	-4.837615036	-5.563528612
-2.576589531	-4.630749767	-2.627689519	-4.587505443	-4.249698304	-0.658583697	-3.377132663	-5.958944933	-5.857611381	-4.106739017	0.021505299	1.98060551	-4.897589242	-5.195310355
-3.242195984	-4.724844768	-2.413454399	-4.606190676	-4.590053333	-0.289040284	-2.623326918	-5.479048348	-5.879767966	-4.220846107	-0.397726361	1.9797109	-4.12374179	-5.053578912
-3.273847213	-5.246011363	-2.479649818	-5.242063001	-4.835896123	-0.605087231	-1.639507794	-4.943562053	-6.010955567	-4.653141807	-0.745422801	1.429498478	-4.358538933	-3.589233368
-2.525907591	-4.042987558	-2.122703613	-3.839339049	-3.93163302	-1.056970531	-2.45689152	-5.444481786	-4.862046311	-3.673419888	0.515729517	1.809270367	-4.790074947	-4.300019823
-3.555544429	-5.068834329	-2.704686482	-4.866394986	-4.640519219	-0.195446721	-3.111535932	-5.730973409	-6.282351742	-4.530957202	-0.441531731	2.160372875	-4.252711677	-5.589368627
-3.314295172	-5.002171605	-2.693099338	-4.870674215	-4.698609279	-0.598784987	-3.228631591	-5.704688524	-5.898032011	-4.513554237	-0.356015482	2.024977024	-4.744517987	-5.721054064
-3.165083348	-4.435952509	-2.344998454	-4.422970619	-4.220511009	-0.733107608	-3.214424666	-5.238246045	-5.933343614	-4.149242737	-0.118128198	1.617628254	-4.398165807	-4.971394121

-3.392721562	-4.651838398	-2.494011339	-4.420521447	-4.651255076	-0.45837378	-3.31756684	-5.795271437	-5.9319595	-4.471471885	-0.430280222	1.848712251	-4.745642156	-5.383750573
-2.894693004	-5.057688955	-2.462941659	-4.51355962	-4.374402929	-0.048114504	-2.854608498	-4.659445504	-5.729951831	-4.562944622	-0.252000786	2.412797826	-3.961467085	-5.542060494
-2.719292852	-4.722627606	-2.628665287	-4.261481113	-4.52415134	-0.173217183	-3.300773497	-4.559425966	-5.54866889	-4.220740567	0.142752174	2.319233897	-4.535468898	-5.82453844
-2.698465151	-4.063280549	-2.1979756	-3.627049913	-4.141371311	-1.029456321	-2.608558677	-5.248964653	-4.328370055	-3.709991797	0.43523231	1.564995691	-4.663963086	-4.397060234
-2.744626663	-4.578360123	-2.590087263	-4.587733409	-4.257023825	-0.853706037	-3.003649964	-5.684489717	-5.838460594	-4.294733118	0.075693482	1.5412962	-4.633341556	-5.01737597
-2.706958304	-5.324469315	-2.386004359	-4.758168436	-4.903739507	-0.028957661	-2.685231289	-4.055752439	-4.271954532	-4.536194457	-0.254644674	1.775030242	-3.801042133	-5.310090648
-2.469549082	-5.276608088	-2.004504434	-4.811196107	-4.881167151	0.16536802	-2.356379733	-3.950970934	-4.098050164	-4.161542128	-0.051627223	2.061106544	-4.034896493	-5.02556137
-3.056134233	-4.628868018	-2.71421737	-4.763665094	-4.284045847	-0.739926666	-3.102499396	-5.823889875	-5.887310626	-4.346223797	0.018718994	1.654768956	-4.757865471	-5.223509518
-2.884666918	-5.124757965	-2.628391894	-4.700464808	-4.367907177	-0.096222465	-2.897601365	-5.319621361	-5.921277826	-4.31652354	-0.219587536	2.313106869	-4.104342544	-5.752738264
-2.980325695	-4.787728145	-2.465511063	-4.48157213	-4.71064069	-0.262039575	-2.919840818	-5.281941247	-5.814190787	-4.329829766	-0.011107685	1.923784521	-4.061325911	-5.153812168
-3.288892551	-5.581490551	-2.703344512	-5.222413361	-5.013131253	-0.682990194	-2.786850371	-5.836491844	-5.564781235	-4.797826716	-0.738753748	1.530209733	-4.544530649	-5.372890228
-3.064838633	-4.988075153	-2.634031032	-4.821865092	-4.603601561	-0.413326572	-2.869000127	-5.129949663	-5.676636597	-4.463764119	-0.574647178	1.913831292	-4.18081243	-4.809497689
-2.476618484	-4.957826003	-3.112113754	-5.105165439	-4.635348879	-1.128217681	-3.537352304	-5.675962648	-6.097195922	-4.519474944	-0.157794607	1.523892299	-4.865108166	-5.666967333
-3.34515968	-5.23346623	-2.739434341	-4.693051913	-4.946313352	-0.592359534	-3.039709339	-5.306842002	-5.425565137	-4.668473855	-0.605288912	1.854145048	-4.150574189	-5.591521248
-3.028964364	-6.431048505	-2.378496945	-5.011525711	-5.664743176	-0.469591715	-2.945628766	-4.824219255	-5.299331981	-4.856875276	-0.485632947	1.73879836	-4.644723557	-5.970723437
-2.763070902	-5.598410177	-2.16173439	-4.729527135	-5.4612103	-0.368112795	-3.024439859	-4.824387487	-5.190296321	-4.694820189	-0.162168851	2.150912306	-4.872961139	-5.456529949
-3.191517503	-4.905091769	-2.809299457	-4.744317839	-4.550669482	-0.55808366	-3.401244873	-5.725136851	-6.100586975	-4.348848	-0.543607824	2.051692779	-4.823837049	-5.82341445
-2.416188488	-3.953373555	-2.508479978	-4.434188345	-4.118549939	-1.153045928	-3.273992141	-5.536108915	-5.055854986	-3.827645113	0.047839554	1.733115303	-4.934430982	-4.877099414
-2.409120574	-4.534452401	-2.286365712	-4.46081052	-4.356218392	-0.299551821	-2.886770293	-4.5208046	-5.685733254	-4.264729514	-0.036079986	1.82749567	-4.480557272	-4.913020651
-3.047359768	-5.400957189	-2.647103364	-5.000725511	-4.865616086	-0.45965498	-2.973460976	-5.43460112	-5.580680948	-4.717596627	-0.46825111	1.735406774	-4.079363843	-5.22325059
-2.711138798	-4.542088018	-2.690631354	-4.662338574	-4.00575359	-0.472413695	-3.10981498	-5.466254943	-5.410273867	-4.296016974	0.139548651	2.043604017	-4.192568116	-4.818321331
-2.648578165	-4.937044535	-2.221264158	-4.953282827	-4.572473622	0.313046102	-2.897366496	-4.354291528	-4.566990508	-5.442764016	-0.441503409	1.322704932	-4.168828671	-4.499568418
-3.237947562	-5.042657744	-2.606014011	-5.028017536	-5.176931877	-0.770396526	-3.1375853	-6.330516057	-5.56543293	-4.928821994	-0.3660431	1.002132218	-4.758751015	-4.470765259
-2.464728245	-5.593166022	-2.304579734	-5.630324196	-5.317834741	-0.155182084	-3.312835465	-5.106213661	-5.414629787	-5.649900819	-0.144975032	1.7005308	-4.593064463	-5.553478646
-2.879236264	-4.946918396	-2.574402421	-4.847358848	-4.866403509	-0.488999768	-3.275566775	-5.493271389	-5.594581111	-4.884791028	-0.357021218	1.721846824	-4.660884032	-5.015893274
-3.06143058	-5.38138013	-2.30881467	-5.483184193	-4.732753283	0.50680815	-2.831577502	-4.645559352	-5.318141765	-4.624683627	-0.494278848	2.238541032	-4.317382659	-5.075760602
-2.003580642	-4.859095395	-1.941425536	-5.244835226	-4.553217205	0.319158756	-2.6169981	-3.471312414	-3.719228862	-4.901373462	-0.197396004	1.371591701	-3.650807138	-4.733920929
-1.619702157	-5.024621862	-2.198447947	-5.252218977	-4.495095439	-0.12220087	-2.93597937	-3.92348519	-4.312921637	-4.993516221	0.151617484	1.715750402	-3.943238486	-4.558406179
-2.507498912	-4.78895051	-2.188954797	-4.971826514	-4.4403943	0.255089248	-2.986191187	-4.137817735	-4.520995448	-4.971492618	-0.204070935	1.561511204	-4.017463101	-4.608451017
-2.817515801	-4.986077915	-2.479386716	-4.721280248	-4.456987051	0.450883883	-2.9554931	-4.442682265	-4.709488275	-4.527375972	-0.249446417	2.017747301	-4.114086518	-4.921156014
-2.313343349	-5.06728965	-1.659034617	-4.964568861	-4.747574637	0.443898406	-2.510476365	-3.558878872	-3.898327684	-5.212755176	-0.315399948	1.428912958	-3.68275862	-4.567960666
-2.291225161	-5.131192832	-2.14137259	-4.751507192	-4.692213675	0.130106629	-2.797270363	-4.016032977	-4.412943833	-4.855148738	0.047395309	1.726355022	-3.943302725	-4.825813306
-2.298709481	-5.062895458	-1.891263303	-5.078198733	-4.436355687	0.333377278	-2.72205134	-3.711554604	-4.018921537	-5.222282051	-0.330584449	1.22467042	-3.711162612	-4.211150289
-1.965306738	-4.849594783	-1.632733345	-4.756533009	-4.436587399	0.419826132	-2.607760536	-3.317621768	-3.891925585	-5.153986245	-0.157978122	1.493233292	-3.538964271	-4.262745403
-3.442586951	-4.763908737	-2.71727674	-4.496559284	-4.698109916	-0.859231194	-3.658872892	-5.550356327	-6.072371735	-4.430356308	-0.536790002	1.611626589	-4.718658801	-5.38341678
-3.005635156	-4.584207142	-2.524412838	-4.395636785	-4.451791164	-0.355502506	-3.289500236	-5.33943664	-6.156674203	-4.033737197	-0.076634573	2.385093579	-4.402210723	-5.485417186
-3.122847313	-4.764600109	-2.392275384	-4.47551767	-4.716332858	-0.220809012	-2.708941161	-4.748248346	-5.359283229	-4.299813436	-0.443483154	2.051860848	-3.880459755	-4.956249075
-3.35421437	-5.054345279	-2.352624346	-4.810459507	-4.540949824	-0.270461641	-2.830123643	-4.877420566	-5.158581031	-4.313537019	-0.709927738	2.250229605	-4.616331212	-5.400911442
-3.00912113	-4.980789054	-2.851382335	-4.822003433	-4.491447091	-0.312578012	-3.373875991	-5.974293725	-6.000218705	-4.265566589	-0.512467295	2.192181906	-4.616530004	-5.330871191
-2.590319476	-4.683967263	-2.437620012	-4.841227547	-4.462926075	-0.453203742	-2.944047074	-5.415576299	-5.50402378	-4.291195824	-0.457395529	1.630982757	-4.676553307	-5.640011909
-3.237872353	-5.010838299	-2.778586443	-4.517721621	-4.714848504	-0.446003487	-3.029765937	-5.438758281	-5.903594281	-4.575432422	-0.538045297	2.140710375	-4.342864271	-5.322539257
-3.085079325	-5.015703728	-2.470351425	-4.233653261	-4.704375917	-0.439046928	-2.810011941	-5.516576343	-5.717567777	-4.489242735	-0.502274423	1.826652317	-4.318639663	-4.957045856
-3.244465509	-5.259315177	-2.102809697	-4.654382317	-4.700755317	-0.233823808	-2.436761398	-5.554950841	-5.961800475	-4.638634546	-0.4889194	1.530314943	-4.103154417	-4.924044887
-3.016875265	-5.26574214	-2.959720732	-5.482759944	-4.897922271	-0.977038654	-3.281703293	-5.527469426	-6.440023692	-4.857502594	-0.405599832	1.654674493	-4.713758836	-5.529345843
-2.423845541	-5.324926933	-2.815586075	-5.038741318	-5.14692141	-1.032640308	-3.493709127	-5.829269471	-5.975004223	-5.051347896	-0.047842087	1.372739538	-5.063288163	-5.604276464
-3.007183375	-5.020287921	-2.501823617	-4.559428128	-5.108291989	-0.667688055	-3.341757781	-5.703205886	-5.69286972	-4.620563901	-0.541410129	1.866778287	-4.700820924	-5.662533251
-2.636282704	-5.51404907	-2.283842472	-4.68511793	-5.315274723	-0.003027058	-2.852845187	-4.724853137	-4.872816526	-4.040892274	-0.036039163	2.235130624	-4.610576423	-5.86302337
-3.398680041	-4.74970942	-2.467705329	-4.616886689	-4.627980481	-0.641228478	-3.105613544	-5.650821338	-6.080780442	-4.246812462	-0.457798098	1.472691074	-4.494820324	-5.220021779
-3.131569517	-5.086975789	-2.433447483	-5.104566159	-4.465277648	-0.079252989	-2.637826542	-5.159596807	-5.453691698	-4.675709248	-0.438456233	2.375292014	-3.639960749	-5.001945338

-1.992879621	-4.772823249	-1.789988341	-4.986404773	-4.308131039	0.557848876	-2.549922642	-3.331059597	-3.844779294	-4.870862389	-0.030327031	1.691875546	-3.625735923	-4.26500077
-2.206263867	-4.802554748	-1.658844514	-5.089167427	-4.341751917	0.557294915	-2.652275657	-3.265127378	-3.637018476	-4.936133612	-0.435962395	1.543656065	-3.499343654	-4.26497751
-2.175838507	-4.861424431	-1.917303335	-4.825879528	-4.361386804	0.594582102	-2.61490779	-3.74531681	-3.953160123	-4.774256992	-0.279920666	1.596928962	-3.5244441	-4.197236694
-2.31847983	-4.843880042	-1.921118614	-4.965884503	-4.295325054	0.652422468	-2.705123769	-3.873190104	-4.055902282	-4.697853931	-0.065426548	1.709921269	-3.783875214	-4.508727175
-2.610473682	-5.020456592	-2.214648199	-4.988288306	-4.56758721	0.465650127	-2.921393358	-4.36467265	-4.706412695	-5.067393401	-0.44063484	1.545983834	-4.048917327	-4.662794345

hsa-miR-675-5p	hsa-miR-6756-3	hsa-miR-6756-5	hsa-miR-6757-5	hsa-miR-6759-3	hsa-miR-6760-5	hsa-miR-6762-5	hsa-miR-6763-3	hsa-miR-6763-5	hsa-miR-6765-3	hsa-miR-6765-5	hsa-miR-6766-3	hsa-miR-6766-5	hsa-miR-6768-5
-2.008644154	-4.17550131	-1.01765019	-1.939695037	-3.657707823	-4.719624842	-1.25199651	-4.433152998	-2.504919999	-2.692046617	1.492179573	-3.200725647	-3.674758821	0.584005161
-1.998395181	-4.32824399	-0.975377822	-1.862519282	-4.040503816	-4.492272683	-1.265769039	-4.564468213	-2.318189518	-1.538185666	1.389487665	-3.634855772	-3.375920055	0.122075648
-1.752645351	-4.223943595	-0.900565471	-1.616979521	-3.87836703	-4.861985603	-1.438133825	-4.29025662	-2.662666271	-1.22636692	1.188039163	-3.372884413	-3.632324676	0.883396133
-1.591310997	-4.178643962	-0.998945594	-1.173096183	-3.791581474	-4.932284301	-1.547320139	-4.51127601	-2.382760592	-1.075837353	1.158715164	-3.196685167	-3.597583411	0.903039986
-1.754149044	-3.953540967	-1.167982935	-1.452336264	-3.350336869	-4.274003175	-1.365706549	-4.157233013	-2.486908715	-1.33175864	1.529482801	-2.90184751	-3.782529367	0.647826452
-1.889171633	-4.215825651	-0.915421363	-1.415222647	-3.82090965	-4.545302913	-1.486218765	-4.255711107	-2.267544756	-1.198970492	1.581680255	-3.244343451	-3.887246163	0.534720256
-1.732448982	-4.018131148	-0.902524405	-1.439963372	-3.588554161	-4.386856593	-1.409228764	-4.11726293	-2.254617727	-0.889869953	1.34790461	-2.973661955	-3.511388195	0.879468154
-1.756896339	-4.373602456	-1.16802882	-1.412842713	-3.935556936	-4.744082699	-1.446056737	-4.473386469	-2.31925279	-0.726884165	1.143150169	-3.512384586	-3.398390348	0.449701061
-1.759910016	-4.391537961	-1.130283499	-1.532371369	-3.988156934	-4.760523388	-1.746259683	-4.573220025	-2.495560731	-1.033780842	1.298566041	-3.440767643	-3.707543045	0.710203071
-1.976508886	-4.250185238	-1.016620959	-1.531778753	-3.793388151	-4.627373983	-1.365061645	-4.379142742	-2.406431353	-1.288053284	1.409951332	-3.31276454	-3.458596527	0.193099041
-1.569717639	-4.404842281	-1.275189995	-0.833298183	-4.179615772	-5.150111896	-1.610949105	-4.264724654	-2.633570274	-0.060473176	1.05921656	-3.375833269	-3.190273028	0.879625471
-2.159578509	-4.608657179	-1.025396401	-2.195245835	-4.431597542	-4.784975235	-1.1454997	-4.894607134	-2.450632378	-2.345334861	1.019797249	-3.792040957	-3.222750134	-0.09583101
-1.988294344	-4.665009526	-1.255125377	-1.940205055	-4.423711777	-4.513634022	-1.350218156	-4.90345942	-2.610646445	-1.246047991	0.85835637	-3.904305831	-3.450380109	0.038520244
-1.948496849	-4.380070233	-1.131432631	-1.636634487	-4.20706225	-5.433624206	-1.45295329	-4.175164793	-2.872130945	-0.734457188	1.239348229	-3.400535727	-3.537327126	0.654533423
-2.029932158	-4.521291261	-1.24932396	-1.936791537	-4.358479696	-4.905714738	-1.226356002	-4.388862382	-2.737289803	-1.349795127	1.400823237	-3.77669098	-3.467803561	0.190057364
-1.70136395	-4.491555487	-1.263746598	-1.765999251	-4.257918651	-4.888916121	-1.475600296	-4.582310642	-2.626570051	-1.333696937	0.970088212	-3.7747948	-3.352654338	0.549916907
-2.121801637	-4.4577431	-1.030933444	-2.12193449	-4.195044817	-4.879364147	-1.386783128	-4.481576355	-2.592121615	-1.774996913	1.338120093	-3.76365303	-3.558322381	0.327642023
-1.98090073	-4.628265972	-1.115855224	-2.015440113	-4.832893722	-5.432712885	-1.684077099	-4.447279716	-2.736414485	-1.129980032	1.1296727	-3.813770418	-3.177528424	-0.115603788
-1.78134329	-4.499021326	-1.206086987	-1.75347976	-4.279164469	-4.855552469	-1.547519201	-4.596534939	-2.877307051	-0.935687752	1.119630782	-3.590036952	-3.503049264	0.396148952
-1.750390357	-4.403137529	-1.110128035	-1.656719824	-4.214405095	-4.73706417	-1.476753494	-4.561422651	-2.615168836	-0.72883794	1.193630382	-3.580638376	-3.37849819	0.432304346
-2.088922177	-4.494021381	-1.065906459	-2.081617095	-4.487630184	-4.994496957	-1.215941918	-4.457306769	-2.775649545	-1.335672345	1.355465857	-3.806806565	-3.733493127	0.069478919
-2.533944296	-4.609569545	-1.264633318	-2.267178463	-4.388005138	-4.859456357	-1.243385327	-4.398241792	-2.660036188	-1.482409381	1.265334336	-3.801416318	-3.622343952	0.069711789
-1.783566837	-4.318240945	-1.199073481	-1.432448027	-4.091875131	-4.980113167	-1.437537876	-4.493502177	-2.628741024	-0.89616981	1.077565658	-3.436760271	-3.555322004	0.629774762
-1.236423908	-4.042049217	-0.744658166	-1.385065453	-3.714747859	-4.55715934	-0.997205814	-4.173970531	-2.496373615	-1.353086577	1.336040372	-2.879096624	-3.375176631	0.786093048
-2.023601421	-4.553239101	-1.398910398	-1.782144869	-4.20103927	-5.024240836	-1.27163201	-4.542216409	-2.700878375	-1.568895376	1.211650773	-3.934789163	-3.509576077	0.218892723
-1.328812935	-3.989476654	-0.958736744	-1.529296862	-3.664712247	-4.813976474	-1.368480907	-4.177689731	-2.545072378	-2.20497368	1.206668613	-2.994162115	-4.113085206	0.762021121
-2.229970829	-4.783016943	-1.289690726	-1.804504856	-4.545980726	-4.670014638	-1.366264144	-4.854875201	-2.654667009	-1.137439151	0.90942072	-3.970651568	-3.340930857	-0.045924842
-1.844633661	-4.125767109	-1.008339138	-1.699919323	-4.157404057	-4.490096281	-1.180138466	-4.489785734	-2.749262754	-1.595560092	1.146137585	-3.377669086	-3.271779098	0.211973471
-1.794350704	-4.506939997	-0.819567142	-2.11481692	-4.203500548	-5.302901707	-1.153925455	-4.530406671	-2.438263288	-2.569902942	1.468578202	-3.965545373	-3.962008757	-0.165289596
-1.631199622	-4.360970646	-1.04352098	-1.154004911	-4.107952576	-4.676479219	-1.435331857	-4.515853251	-2.458995774	-0.794546762	1.187817654	-3.585244201	-3.511531826	0.455937499
-1.442751576	-4.436615715	-1.102455768	-1.116193531	-4.177621306	-4.340332275	-1.544857666	-4.534615	-2.367020275	-0.647915215	1.023486689	-3.382087532	-3.179724202	0.615288557
-2.387606872	-4.991082223	-1.499687235	-1.936002868	-4.892025517	-5.254931203	-1.387963096	-5.200640162	-3.099537183	-1.655670466	1.200594627	-3.835875	-4.075311655	-1.013444454
-2.553068542	-4.641037712	-1.259657052	-1.926623102	-4.637602682	-4.767719174	-1.181396812	-4.296487182	-2.166637486	-2.168743625	1.432848715	-4.175529491	-3.427498638	0.063184908
-2.071235046	-4.490499456	-0.670529111	-1.545892912	-4.995668163	-5.360886925	-0.567135021	-4.179839385	-1.823728835	-0.799086781	1.61307832	-4.040327898	-3.301938246	0.990601395
-1.921421079	-4.743240244	-1.29765114	-2.335236349	-4.701371456	-5.603465221	-1.295154005	-4.580707499	-2.818942287	-2.136336255	1.186248555	-4.253979438	-3.61828695	0.544011872
-2.397069892	-4.042872159	-1.080912673	-2.418449561	-4.337634816	-5.388543573	-0.96651992	-4.130838549	-1.863239824	-2.470513117	1.980473749	-3.670109505	-3.745373512	0.6068431
-2.682612144	-5.319306158	-1.246286486	-2.727614808	-5.397591571	-5.118380053	-0.935878809	-4.089653724	-2.605973017	-2.03315022	1.473719627	-4.291946145	-3.963394248	0.569782871
-2.1185424	-4.431443282	-1.336529239	-2.228934626	-4.513426225	-5.280234067	-1.291224204	-4.047126002	-2.217559251	-1.383238069	1.422313355	-3.896227266	-3.298804966	0.221971207
-1.788431654	-4.35605681	-1.345260191	-2.61253328	-4.257106098	-5.396596976	-1.345397456	-4.403386658	-2.276783941	-1.956737856	1.093238617	-3.766351138	-3.278773018	0.824257224
-3.017795593	-4.405535574	-1.326315341	-2.244562491	-4.712176408	-4.685819041	-1.105796224	-4.004013903	-2.248181566	-2.605194969	1.886334815	-3.676900843	-3.626032405	0.199546132
-2.433958719	-4.414529793	-1.354421542	-2.931801425	-4.152089415	-5.391124909	-1.160359498	-4.255155444	-2.376239534	-3.540575569	1.781247441	-3.895229981	-3.625554833	0.120704567
-1.790062555	-4.264728195	-0.952899449	-1.750931939	-4.97427854	-5.006568171	-1.115159156	-4.27524218	-2.166650105	-0.490913995	1.156636755	-3.889867197	-3.393260461	1.201414986
-0.822818547	-4.777282465	-1.1885044	-2.443731813	-4.990035487	-5.101428785	-1.455123401	-4.376178787	-2.610272368	-1.189096455	0.730734609	-4.077240004	-3.924478701	0.941276049
-1.509701617	-3.763282209	-0.903623092	-1.14387955	-3.505997167	-4.980433978	-1.202054302	-3.428233083	-2.316714704	-5.102643382	1.863861003	-2.257870886	-3.721646861	0.95707042
-2.005939901	-4.631025141	-1.0919387	-2.389568465	-4.63630657	-5.183304735	-1.070877614	-4.277965213	-2.100998432	-1.178123823	1.4520624	-4.203877935	-3.617895578	0.98870642
-2.052931364	-4.615645294	-1.367520041	-2.464292671	-4.581185455	-5.488002101	-1.304166545	-4.368561508	-2.345373543	-1.869698415	1.336498623	-4.292734402	-3.635687904	0.348794277
-1.744064815	-4.291921967	-1.404444583	-2.028519727	-4.185452131	-5.277889838	-1.408261496	-4.297444878	-2.387754469	-1.357341378	0.9400834	-3.390279994	-3.483815035	0.515971524

-1.590406767	-4.532021247	-1.29919614	-1.774578334	-4.705300401	-5.516342261	-1.602161832	-4.493534259	-2.201531173	-1.078761276	1.236612301	-3.890552561	-3.274569392	0.553295913
-2.412429236	-4.61348827	-1.110591357	-2.18528204	-5.038377049	-4.301511859	-1.073189798	-4.167577775	-1.995133321	-1.673353823	1.800751385	-3.865059253	-3.314957813	0.519794576
-2.376360833	-4.332350426	-1.225759248	-2.158290912	-4.668388272	-4.631955647	-1.238946008	-3.950032128	-2.037941961	-2.378580704	1.877730706	-3.55591632	-3.223987836	0.224668304
-1.632776656	-3.731600625	-0.959574174	-1.019538462	-3.840947614	-4.971479745	-1.764704194	-3.621955853	-2.094189921	-3.80167707	1.550711883	-2.124454591	-3.115270026	0.927417699
-1.763712884	-4.266636401	-1.168626504	-1.889555962	-4.090335398	-5.146202408	-1.069404733	-4.104088653	-2.843660656	-2.553055784	1.519517316	-3.000064978	-3.925463131	0.782221546
-2.41976379	-4.580393424	-0.935766262	-1.677491369	-4.940986936	-3.910161632	-1.052058981	-3.721700924	-2.057995711	-2.127176623	1.461051258	-3.905926311	-2.648623527	0.442633274
-2.834824443	-3.919577035	-0.908721929	-1.525965601	-5.152825913	-3.390529151	-1.008028849	-3.342826882	-1.717222058	-3.789747975	2.007542531	-3.437709781	-2.759520327	-0.354363252
-1.711917809	-4.376433383	-1.265457852	-1.687675733	-4.154748728	-5.142334095	-1.163429159	-3.985375355	-2.713682915	-2.494633104	1.601718679	-3.249930074	-3.994721355	0.389609168
-1.698537795	-4.316026327	-0.7917936	-1.847693313	-4.8538522	-5.079403963	-0.879260535	-3.66641585	-1.974230721	-1.028301222	1.796812925	-3.858474778	-3.261905883	1.102405915
-1.390748276	-4.435933195	-1.024388299	-1.612578686	-4.724090102	-5.111582136	-1.013766836	-4.392891525	-2.052416903	-1.020575947	1.372635667	-3.975693815	-3.472205189	1.206998142
-2.396281622	-4.945693222	-1.305692194	-2.480160124	-5.051812968	-5.05355283	-1.269306606	-4.381344424	-2.841696703	0.033093381	1.08823204	-4.422653467	-3.721663437	0.701500279
-1.994498184	-4.739930926	-1.329558117	-1.380934611	-4.726647364	-4.907063797	-1.310242612	-4.396132718	-2.505553083	-0.914105657	0.95376998	-4.32669818	-3.561426435	0.943906823
-2.565412099	-4.77290168	-1.393289972	-2.967822172	-4.700401108	-5.293105697	-1.226090917	-4.628849735	-2.650308692	-3.205379795	1.607481031	-3.914896712	-3.663688212	-0.025432345
-2.271309788	-4.518348391	-1.161782142	-2.261521549	-4.782692669	-5.061237139	-1.326931319	-4.264421637	-2.558756731	-0.951832624	1.194418259	-4.203758931	-3.311783	0.833425078
-2.721426646	-4.358217364	-1.143172627	-2.063371502	-6.092017447	-4.543152535	-1.576023042	-3.695139397	-2.422555368	-2.608348169	1.41194163	-3.593191795	-3.053794957	-0.219031164
-2.731479941	-4.516906127	-1.32717146	-2.115825941	-5.06909098	-4.169927038	-1.081797318	-3.871975357	-2.122324537	-2.455921757	1.79709011	-3.893954012	-3.216823877	-0.194988419
-2.218679454	-4.497697197	-1.563115953	-2.355235017	-4.72205619	-5.447139168	-1.594329873	-4.091291365	-2.38758811	-1.686838217	1.196949117	-4.147488536	-3.67703444	0.444417894
-2.479614296	-3.873016555	-0.985782361	-2.376862119	-4.119454301	-4.997991675	-1.414960328	-4.161411874	-2.375253885	-3.497698377	1.64721486	-3.260827609	-3.396512789	0.299594304
-1.28811191	-4.153998464	-0.880096902	-1.934430473	-4.224836436	-5.448759818	-0.871344401	-4.354032565	-2.177123084	-1.6475495	1.273417163	-3.543340862	-3.320430896	1.064866063
-1.954102831	-4.783746848	-1.092974803	-1.371372816	-4.939435443	-5.014399146	-1.601187551	-4.463124657	-2.458054185	-1.430147567	0.902927268	-4.631662211	-3.564419251	0.800399829
-1.841776758	-4.340612774	-1.047636214	-1.893002881	-4.146129649	-4.93245909	-1.455375014	-4.627470954	-2.175177711	-2.056731882	1.529087002	-3.741866913	-3.800494163	0.822395637
-2.633480589	-5.151538197	-0.895048357	-1.970738845	-4.710464324	-4.397332411	-1.502260642	-5.021812253	-2.358012519	-0.39337502	0.26434599	-4.325990973	-3.494980316	-1.120206612
-1.79025296	-4.999772517	-1.420409217	-2.193802252	-4.932801223	-5.058368589	-1.676853997	-5.361421732	-2.62934094	-1.388843947	0.70328608	-4.313505747	-3.365230233	0.130332424
-2.742149687	-5.746829609	-1.181159597	-1.719429274	-5.644867033	-4.926267649	-1.171115811	-5.20474195	-2.430514529	-2.662593537	1.19881313	-4.77814167	-3.532711154	-0.389778831
-2.079280234	-4.944602328	-1.282380539	-1.876321591	-4.730908106	-4.836596838	-1.350230508	-5.010353775	-2.545898617	-2.509494886	1.129993452	-4.134765506	-3.52321427	-0.250021025
-2.638199602	-4.920897611	-1.06421392	-2.229749347	-4.759584207	-4.847392046	-1.599436002	-4.007295821	-2.315284913	-1.041884113	0.797876249	-4.128984951	-3.437353101	-0.097262792
-2.6593885	-4.886082511	-0.565742182	-2.100988505	-4.770715158	-3.888289641	-1.183964308	-4.726295659	-2.088313541	-2.083633124	0.817459817	-4.461418451	-3.361148662	-1.271111801
-2.700096007	-4.942640981	-0.797721349	-2.427972668	-4.372435293	-4.098535475	-1.04782559	-4.945207477	-2.15320807	-2.643478882	1.202762602	-4.341316409	-3.632667737	-1.219101214
-2.512327182	-4.929144429	-0.857781549	-2.147718286	-4.580977771	-4.192762645	-1.358125378	-4.765317574	-2.195066588	-1.134052303	0.698534692	-4.228531392	-3.361440251	-0.840306213
-2.198120937	-4.73997207	-1.048643246	-1.784960276	-4.458106409	-4.641472923	-2.059963487	-4.582799463	-2.247891028	-0.428187368	0.727241925	-3.938813791	-3.527804858	0.134716029
-2.512706618	-4.948070716	-0.629095233	-1.977516432	-5.047556708	-4.133314393	-1.660890757	-4.648129019	-1.922430598	-1.397815225	0.421815138	-4.40056549	-3.18197285	-1.101437284
-2.617969695	-4.665633027	-0.815047746	-1.869487845	-4.643153848	-4.364678978	-1.289817533	-4.431339645	-1.966767842	-1.944395473	1.297741876	-3.804537744	-3.390050684	-0.69358514
-2.64283879	-4.988690222	-0.725088775	-2.013098523	-4.872390954	-3.916971208	-1.5088905	-4.688678684	-2.08078256	0.205254775	0.416022191	-4.312563888	-3.284468762	-1.044889613
-2.596747679	-5.026192997	-0.460431332	-1.969862105	-4.534088474	-3.726573116	-1.390474874	-4.917299959	-1.630390528	-0.922017043	0.479610437	-4.072176779	-3.229372647	-1.242073166
-2.427371124	-4.714056556	-1.177945545	-1.572674197	-4.824955441	-5.257267903	-1.227084573	-4.601013477	-2.515142898	-1.177385654	1.19337472	-4.001283394	-3.386774804	0.482103701
-2.047845745	-4.182778475	-0.866242807	-2.145076531	-4.450786913	-5.257590749	-0.976011091	-3.721473826	-2.049536397	-0.964009429	1.704549553	-3.455832593	-3.126969367	0.953665548
-2.132877852	-4.401898166	-1.027438694	-1.406376413	-4.746924574	-4.62928349	-1.36974135	-3.859493249	-2.30399021	-0.48419192	1.251707192	-3.71909858	-3.220820916	0.990503031
-2.151275893	-4.70559298	-1.334939953	-1.599908865	-4.789561652	-4.602612147	-1.693402924	-4.157733833	-2.271381012	-0.991604325	1.116551697	-4.182997782	-3.316613121	0.327529838
-2.436148724	-4.450261309	-1.594049461	-2.466856503	-4.362185108	-5.539257624	-1.371058337	-3.973212786	-2.125724603	-1.892690519	1.3827123	-3.961848994	-3.607857986	0.578737089
-2.244825843	-4.577066602	-0.998819202	-2.011265623	-4.332294667	-4.875916158	-0.883787824	-3.624174337	-2.472245425	-4.207047257	2.150184042	-3.697990689	-3.829578321	0.146256111
-2.109324574	-4.549584013	-1.287501635	-1.477998005	-4.711047864	-5.084967077	-1.3824921	-4.205835315	-2.4189604	-0.279606236	1.259796924	-3.990537854	-3.605846357	0.901099014
-1.930155899	-4.689379758	-1.285779253	-1.102771632	-4.783293761	-5.245566724	-1.479803047	-4.788804512	-2.565512839	-0.341774913	0.993349139	-4.158549439	-3.597420192	1.221393308
-1.628370237	-4.899497554	-1.098689416	-2.507611297	-4.693332748	-5.151043068	-1.3610684	-4.669534828	-2.726523086	-0.452669079	0.991784803	-4.444680679	-3.359112987	1.308460496
-2.451937912	-4.885603269	-1.371301048	-2.410222458	-4.700806322	-5.589988673	-1.165849177	-4.539660241	-2.818674178	-2.873900439	1.601268825	-3.936674843	-4.3269431	0.184456367
-2.884086475	-4.944361273	-1.200879036	-2.639730974	-5.156238727	-5.639563632	-1.18531937	-4.657841172	-3.046339515	-2.40107466	1.532233399	-4.216548059	-3.698656184	-0.19793962
-2.314587116	-4.761035396	-1.398486015	-1.68611418	-4.735446477	-5.145737428	-1.505878045	-4.145517537	-2.481199572	-2.101232149	1.103941055	-4.474725549	-3.571936543	0.247979725
-2.834330158	-4.289887426	-1.045094199	-1.94637351	-5.39328904	-4.314635134	-1.075709175	-3.173274938	-2.548400717	-2.461047507	1.810714439	-3.638022785	-3.236949297	0.262899271
-1.769582253	-4.387800677	-1.259619198	-1.678348165	-4.902719761	-5.52441025	-1.726388593	-4.395147171	-2.68764938	-1.444360454	1.063099487	-3.909500536	-3.421157626	1.093987189
-2.119126795	-4.861141048	-1.235434673	-0.927445061	-4.757954728	-4.348340996	-1.4748876	-4.294082625	-2.489595618	-0.337757486	1.350809163	-4.241169287	-3.448528837	0.888229683

-2.534954562	-4.619121176	-0.664326762	-1.929365331	-4.499291006	-3.731967117	-1.188517713	-4.763080494	-1.755715402	-1.211186576	0.841709571	-4.104595417	-3.144651924	-1.133518406
-2.577556028	-4.747125012	-0.604199233	-1.711427572	-4.543537402	-3.547077174	-1.517929393	-4.597271679	-1.890070676	-0.789350567	0.369036353	-4.149902414	-2.973129336	-1.107451389
-2.477074368	-4.750012103	-0.666746683	-1.7934039	-4.531486822	-3.608903914	-1.485697889	-4.358067444	-1.896374921	-0.723265423	0.450844527	-4.265392256	-3.243181216	-0.843349034
-2.768567826	-4.745754186	-0.77597894	-1.944026349	-4.562926329	-3.905496116	-1.403076394	-4.622745508	-2.108728173	-0.967456288	0.54289083	-4.352819986	-3.28531578	-1.171097263
-2.503011352	-5.097758555	-0.955702284	-2.101213441	-4.704868258	-4.530609824	-1.57520165	-5.08221517	-2.356152432	-0.950442674	0.420431544	-4.377756398	-3.418949471	-0.964640069

hsa-miR-6769a-	hsa-miR-6769a-	hsa-miR-6769b-	hsa-miR-6770-3	hsa-miR-6771-5	hsa-miR-6772-5	hsa-miR-6774-5	hsa-miR-6775-5	hsa-miR-6776-5	hsa-miR-6777-3	hsa-miR-6777-5	hsa-miR-6778-5	hsa-miR-6779-3	hsa-miR-6779-5
-3.786260984	-2.609901603	-2.098946628	-6.074566228	-1.020082416	-4.215600886	-2.456825269	-0.145357826	-3.393494188	-3.522675113	-3.14166747	-0.657520226	-4.210424705	-1.464603071
-4.375172436	-2.970853047	-2.341530572	-5.355924384	-0.580310239	-4.350287306	-1.823876239	-0.053933186	-3.519281944	-3.957424753	-3.012779131	-1.846429248	-4.531444155	-1.807176018
-4.270605129	-2.609358599	-2.199121436	-5.281612076	-0.728136261	-4.369389488	-2.07602028	-0.238991691	-3.480134041	-3.696728511	-3.097140527	-1.851237288	-4.362083095	-1.539104354
-4.2247843	-2.510625117	-2.372112838	-5.555668687	-0.775067154	-4.5023772	-1.897207466	0.036722381	-3.279545173	-3.695479205	-2.883810851	-2.905284983	-4.47757235	-1.436473628
-3.939488144	-2.463933849	-2.038494156	-5.441596702	-0.79125427	-4.070113446	-2.152662632	-0.200009796	-3.30381571	-3.427532717	-3.1920376	-1.649948183	-3.935383353	-1.230699592
-4.008993726	-2.6484109	-2.18934295	-5.390436024	-0.771243305	-4.344523499	-2.303201116	-0.423618272	-3.428194557	-3.620632359	-3.032320232	-1.944712816	-4.535857598	-1.751558245
-4.068955082	-2.46267219	-1.980821663	-5.672771413	-0.841614204	-4.281356317	-2.059924204	-0.003220528	-3.734202685	-3.510465965	-2.753492525	-1.843796225	-4.428668578	-1.383578069
-4.261373423	-2.908706245	-2.347909447	-4.970731233	-0.69283274	-4.543431267	-1.919651601	-0.208589212	-3.648701923	-3.77335564	-2.94149637	-2.965837715	-4.446776055	-1.906172059
-4.279343214	-2.716160109	-2.45319638	-5.161116499	-0.92061309	-4.52167314	-1.014780255	-0.415092659	-3.689710548	-3.852165645	-3.101769327	-2.202578702	-4.637584341	-1.78251178
-4.162978161	-2.664709845	-2.293987967	-5.156077213	-0.528899516	-4.323239567	-2.259489329	-0.068688552	-3.903564734	-3.834806236	-2.884127466	-3.256172106	-4.306853196	-1.811892462
-4.585508266	-2.823382082	-2.655871648	-5.426700451	-0.468734315	-4.893463714	-1.789115537	-0.251693761	-4.090644834	-3.775260478	-3.125163585	-3.503883614	-4.742345875	-1.661515318
-4.807099111	-2.675187332	-2.236031766	-5.460394257	-0.676017267	-4.663280253	-2.193906992	0.037485888	-3.722907348	-4.182863049	-3.163203393	-1.499034208	-4.855865827	-1.530375214
-4.798846129	-2.89198497	-2.224632584	-5.395677955	-1.032172398	-5.112007664	-2.181546823	-0.211381242	-3.906725256	-4.189970879	-3.332704431	-1.916020019	-4.978845615	-1.881437625
-4.537736347	-2.755280313	-2.245739	-5.492789018	-0.563205562	-5.057175215	-2.043222562	-0.224283878	-3.972003714	-3.78420921	-3.13197308	-3.762111818	-4.854161216	-1.717664697
-4.638573171	-3.286961993	-2.446911455	-5.220240135	-0.464840411	-5.062274923	-2.367135634	-0.441979077	-4.305649835	-3.903161912	-3.348954121	-2.765984492	-5.057466192	-2.08749247
-4.679787922	-2.893693933	-2.514917662	-5.043746469	-0.528023932	-4.825765156	-1.918915965	-0.328586669	-3.648162958	-4.00781567	-3.280222282	-2.665277245	-4.914570488	-1.840308937
-4.633468461	-2.89079198	-2.534873012	-5.072960271	-0.573068734	-4.925314593	-2.495730323	-0.416433789	-4.033497774	-4.00251115	-3.19206176	-1.95712217	-5.065793273	-2.16739866
-4.890131959	-3.238321579	-2.453708155	-5.210272462	-0.619438044	-5.005284386	-2.018220593	-0.287612118	-4.381528237	-4.102679384	-3.453443461	-2.376890656	-4.955400636	-2.002353383
-4.593506479	-2.712071232	-2.119293356	-5.643664877	-0.800969923	-4.93849544	-1.938609382	-0.187304997	-3.950917601	-3.952317554	-3.356387597	-1.96121826	-4.916851482	-1.6281432
-4.613354817	-2.718305511	-2.273803649	-5.139143292	-0.552627072	-5.045590857	-2.127378647	-0.191537355	-4.105923371	-3.902817409	-3.147502849	-2.438803415	-4.940379142	-1.843464876
-4.707752476	-2.977221642	-2.510557589	-5.335653259	-0.560255174	-5.008487884	-2.537357849	-0.322774837	-3.936565319	-4.048482282	-3.164931931	-1.901514384	-5.041591202	-2.078508717
-4.720291997	-3.211478479	-2.66748719	-4.70065652	-0.580265	-5.245384477	-2.544868847	-0.501274469	-4.355791629	-4.093266521	-3.291762739	-2.820005838	-5.086689051	-2.117882735
-4.388258487	-2.766998391	-2.349827788	-5.017709528	-0.779913101	-5.222123714	-1.699005169	-0.260155685	-3.95355137	-3.829218796	-3.234713785	-2.773409732	-4.781231581	-1.700952611
-4.047519138	-1.873325459	-1.730621103	-5.416311193	-0.594226742	-4.791900765	-1.779369352	0.360113555	-3.529947593	-3.50695594	-3.234235082	-1.599877754	-4.372524507	-0.935374678
-4.588717248	-3.272229673	-2.771518185	-5.001555417	-0.544956464	-5.079576567	-2.25164266	-0.587705501	-3.924423898	-3.873059467	-3.566995571	-3.581032833	-5.027960691	-2.000603556
-3.93974103	-2.207934705	-1.962669754	-5.853982821	-0.78041793	-5.12664804	-2.105373605	0.092957462	-3.705500014	-3.503389025	-3.374871366	-1.902740783	-4.560601039	-1.229364994
-4.725121178	-3.128438713	-2.575691292	-5.800462465	-0.800296512	-5.097731153	-2.157665923	-0.216502796	-4.10671618	-4.09942595	-3.37674333	-2.821338479	-5.223003869	-2.02941128
-4.589997412	-2.657543479	-1.899451368	-5.773230148	-0.863959237	-5.02843924	-2.207759472	0.124808997	-4.183844727	-3.95998299	-3.252913468	-2.561812444	-4.813269726	-1.436707753
-4.053730822	-3.133825872	-1.93371877	-5.595375388	-0.446044666	-4.286432323	-2.635983024	-0.788305243	-3.906135481	-3.831168024	-3.2052385	-1.881515611	-4.368008469	-1.742631098
-4.504970862	-2.629323166	-2.332841936	-4.822113148	-0.524893477	-4.816914467	-2.212129582	-0.007470687	-3.762547673	-3.936907544	-2.950161804	-2.911446338	-4.726908124	-1.506469682
-4.546050356	-2.654399563	-2.363934711	-5.004221049	-0.581842409	-4.81424097	-1.753732625	0.086903262	-4.064275834	-3.999583813	-2.888560134	-3.153388418	-4.671522383	-1.460679481
-5.238432369	-3.47614828	-2.729510286	-6.812506285	-1.009695174	-5.378021901	-2.66167673	-0.229871764	-4.433988985	-4.636918527	-3.612488676	-3.409584876	-5.604986562	-2.107853698
-4.660490932	-3.397045103	-3.073962308	-4.947233798	-0.217145971	-4.682619547	-1.873315042	-0.430894131	-3.432887256	-4.334816633	-2.818486386	-2.88571131	-4.898007504	-2.142734913
-4.729410995	-3.500070581	-3.106911356	-4.85202409	-0.260306801	-4.652854092	-2.441679986	-0.032061222	-4.023161099	-4.265584612	-2.657029755	-3.752389915	-4.951237255	-2.023044288
-5.128216593	-3.551396508	-2.816611391	-5.860902505	-0.646912011	-5.393324652	-2.771755605	-0.597911944	-4.177269499	-4.323212291	-3.583456222	-4.468091035	-5.226586684	-2.419081296
-4.418270207	-3.3576941	-3.014174634	-4.945476556	-0.449069353	-4.533037486	-2.65594673	-0.311269552	-3.481097139	-3.82696684	-2.820359479	-2.423833481	-4.775826092	-2.049019591
-5.383145095	-4.156834286	-3.850056901	-6.095124258	-0.339138242	-7.110508163	-2.666777447	-0.90787767	-4.501576481	-4.255495805	-3.498894776	-2.796715421	-7.560796569	-2.555212715
-4.969525067	-4.010917982	-3.208924517	-5.034969249	-0.646803855	-5.275215355	-2.4681285	-0.652806143	-4.391995716	-4.073798342	-3.455290476	-4.406671577	-4.955193471	-2.486505095
-4.691639685	-3.586485617	-2.984199849	-5.31045688	-0.709479408	-5.004312357	-2.414102369	-0.493954767	-3.643893626	-3.974741257	-3.509397568	-4.392414635	-4.772998569	-2.341609333
-5.09953456	-3.424881379	-3.405974765	-4.826888917	-0.560016385	-5.145665026	-2.847552572	-0.749385851	-4.582570857	-3.953611576	-3.202269666	-2.766111597	-5.314789142	-2.642632032
-4.722935234	-3.64721306	-2.895637631	-5.530111232	-0.893176362	-4.960403919	-2.745446753	-0.667649927	-4.185534756	-3.984986288	-3.414967493	-1.74002532	-4.819860616	-2.472518347
-4.906642331	-3.679510357	-3.367058264	-4.94388088	-0.550392658	-5.271097751	-2.207102628	-0.639363175	-4.094235968	-4.082905529	-3.047377054	-3.828601841	-5.254696162	-2.158119194
-5.342978891	-3.817010489	-3.288153597	-5.375298458	-1.151142362	-5.410982075	-2.46636669	-0.938481223	-4.389510303	-4.505360595	-2.284534469	-3.836023149	-5.664685573	-2.736855478
-4.030388087	-1.929158023	-1.965817896	-6.068906664	-0.639975783	-4.148248669	-2.160172938	-0.142431493	-3.239184636	-2.960801233	-3.297788592	0.380270814	-4.279533446	-0.808250275
-5.137548881	-3.743932515	-3.474581684	-5.483791168	-0.465538726	-5.458334043	-2.847695442	-0.628904958	-4.219530093	-4.113459238	-3.244871083	-3.772124172	-5.281312243	-2.500078239
-5.130895635	-3.832621431	-3.277097241	-5.426056604	-0.637370211	-5.165685439	-2.790405484	-0.540439823	-4.096304127	-4.195475463	-3.273720386	-4.287827924	-5.241904905	-2.521666585
-4.524435645	-3.345754706	-2.672877339	-5.203642844	-0.656551785	-4.687561283	-2.465978736	-0.095646176	-3.497433875	-3.769334858	-3.54632489	-1.881076756	-4.656959162	-2.225019516

-4.862090897	-3.607234154	-3.103723006	-5.053200909	-0.482381266	-5.065127772	-2.070837609	-0.226980833	-4.269772564	-4.162163911	-3.219054216	-3.402896988	-5.033059155	-2.451047687
-5.260272335	-3.405261389	-3.552421561	-4.470675035	0.042036803	-5.029091148	-2.526116744	-0.510176068	-3.865212997	-4.129092416	-2.928855465	-2.532503193	-5.445503606	-2.547698959
-4.830861335	-3.25621177	-3.038930541	-4.754222728	-0.051137161	-4.726652436	-2.364112846	-0.066321696	-3.518947486	-3.903768856	-2.842975376	-3.577650709	-4.970339489	-2.318672493
-4.028776202	-1.978388192	-2.107398663	-5.153317708	-0.561940461	-4.177830091	-1.426278227	0.199042907	-3.204709873	-3.166646451	-3.232385411	0.812253002	-4.344321283	-0.854545031
-4.323392452	-2.83450387	-2.690436052	-5.667263118	-0.896645894	-4.969880633	-2.442862998	-0.431103468	-3.764025442	-3.59279995	-3.484262645	-1.37807473	-4.791694831	-1.731182629
-5.537402838	-2.790645063	-3.046169672	-4.54744039	-0.12909359	-4.960406959	-2.036634597	-0.365223039	-3.907956101	-4.494841498	-2.378581201	-2.628977958	-5.30649878	-2.317331664
-6.068617138	-2.234995886	-3.084310568	-4.294389122	0.407252394	-5.425038154	-2.184852062	-0.386778539	-3.555892523	-3.93641395	-2.325956397	-2.53261903	-6.129318924	-2.143821981
-4.406280822	-2.982309166	-2.625275633	-5.638697878	-0.471080654	-4.992999125	-2.641465711	-0.507423625	-4.044651206	-3.627313349	-3.656720657	-2.632277532	-4.962139981	-1.894683091
-4.886756239	-3.349916597	-3.166422622	-4.82957794	-0.125495259	-5.656868994	-2.175824924	-0.278442642	-4.07007584	-3.950510401	-2.923780848	-3.753701534	-5.520702231	-2.203871038
-4.800939655	-3.370703133	-3.199615732	-4.63074404	-0.404924003	-5.155212792	-1.863296776	-0.209320079	-3.730060527	-4.09552609	-3.207317794	-3.428686386	-5.091428877	-2.063220568
-5.242616938	-4.071694079	-3.329840299	-5.271673017	-0.992130163	-5.653899686	-2.455018018	-0.860201935	-4.405332454	-4.608795864	-3.431090834	-3.144337602	-5.598136325	-2.691048149
-4.786817103	-3.731869498	-3.326369484	-5.104872155	-0.58869881	-5.186917244	-2.351562271	-0.709178207	-3.779006259	-4.166296783	-3.215857954	-3.669676593	-5.208435952	-2.544086776
-4.904532488	-3.641740791	-3.152606138	-5.437937273	-1.265860231	-5.183943852	-2.936634433	-0.832510081	-4.150275403	-4.206187135	-3.59755935	-2.636986471	-5.185602788	-2.263748579
-5.333442251	-3.777025232	-3.54364575	-5.27107683	-0.641093297	-5.229051831	-2.912051745	-0.921771273	-4.084015451	-4.529818229	-3.225804845	-4.272643291	-5.683699176	-2.614443746
-6.743687852	-3.205966855	-3.616583309	-5.811461393	-0.191162793	-6.356097493	-2.566390565	-0.570767223	-4.533673352	-5.187311782	-2.613466932	-3.907310132	-6.785972149	-2.249316387
-5.171220284	-3.558903574	-3.305834498	-5.039543818	-0.224485532	-5.639119789	-2.605730829	-0.500250437	-4.250030807	-4.176275829	-3.167568572	-4.062188891	-5.507568748	-2.504960523
-4.83444016	-3.746368954	-3.365981008	-5.065014946	-0.73873104	-5.176304412	-2.457608157	-0.790602973	-4.073373154	-4.170711526	-3.474134515	-4.322914245	-4.964843674	-2.788956485
-4.021287339	-3.240731806	-2.202773006	-5.343681449	-1.062656579	-4.241710357	-2.622494201	-0.597624726	-3.755202017	-3.500132881	-3.404117372	-0.846842471	-4.084902616	-1.997253181
-4.510143185	-2.917274832	-2.496883208	-4.570222878	-0.120337026	-4.885898348	-2.149589174	-0.033605877	-3.183250474	-3.862392857	-3.439860741	-2.472244812	-4.764573684	-1.677522569
-5.155436945	-3.603789653	-3.320032867	-4.834509863	-0.492254213	-5.725818827	-2.392596829	-0.639890131	-4.368455503	-4.36583361	-3.154873575	-3.686522614	-5.63442468	-2.259460597
-4.527918866	-2.865977007	-2.727480941	-4.953817726	-0.567766464	-4.656230817	-2.064941926	-0.570570191	-3.702694683	-3.936396325	-3.106303953	-1.258757658	-4.851333917	-1.808357906
-4.933124551	-2.578275141	-2.529459861	-5.306072188	-0.537507799	-4.444219263	-2.871310733	-0.878782019	-4.105457702	-4.59699416	-2.487302594	-2.410542621	-5.088440297	-2.028851894
-5.25355603	-3.348312352	-2.912695887	-5.692161142	-0.910963308	-5.340767699	-2.184597329	-0.04176613	-4.706071215	-4.669363926	-3.443886013	-3.367860434	-5.431620678	-1.897852503
-5.860485858	-2.86406639	-3.33348846	-4.711093979	-0.170803924	-5.654686507	-2.615962428	-0.057136102	-4.30523371	-4.904476313	-2.957329765	-3.291973067	-6.259109289	-2.029784899
-5.185825375	-3.656941634	-2.645378575	-5.671759566	-0.784691931	-5.149441257	-2.354616823	-0.235374479	-4.289805704	-4.481817075	-3.23183226	-3.368020989	-5.32623507	-2.354915207
-5.546409392	-2.681639642	-2.689335652	-5.247398157	-0.506124187	-5.143522876	-2.7028536	-1.072242117	-4.375351073	-4.055637489	-2.867430965	-3.262155296	-6.308319108	-2.43452033
-5.146190846	-2.2077578	-2.113350692	-5.417921165	-0.449643319	-4.003552367	-2.996823072	-0.906928413	-3.565114616	-4.458323658	-2.355080047	-1.733028083	-4.936766343	-2.221399433
-4.564210914	-2.478828257	-2.32206487	-5.076390715	-0.478273728	-4.193346979	-2.796062539	-0.683802534	-3.788447548	-4.258933807	-2.526193046	-1.483863801	-5.089474213	-1.955175964
-4.863106684	-2.509212689	-2.473765096	-5.525374121	-0.429163612	-4.314940845	-2.781171589	-0.703651991	-3.669745897	-4.407703098	-2.421264541	-2.605225135	-4.965661043	-2.023324686
-5.095074964	-2.680903346	-2.549048049	-4.989357473	-0.416529301	-4.602712435	-2.308316185	-0.70843342	-4.227087791	-4.036239123	-2.820875723	-3.029774949	-5.116594175	-1.950533112
-5.293546536	-2.068989331	-2.244754662	-5.149305596	-0.362145027	-3.93392589	-2.743666159	-0.771355514	-3.625399704	-4.75241715	-2.21048741	-2.112500337	-5.515445682	-2.1101596
-5.018582138	-2.616031476	-2.498583328	-5.307464615	-0.388195312	-4.514281782	-2.618150087	-0.716621496	-4.22470871	-4.423749469	-2.902406806	-1.714693436	-5.288549288	-2.139002814
-4.891851978	-2.28886505	-2.302499858	-5.060287766	-0.544444505	-4.147985395	-2.889684929	-0.774345861	-3.754924337	-4.600974489	-2.179255618	-2.109455394	-5.100312047	-1.951453467
-5.067614	-2.112265726	-2.050543351	-5.219060069	-0.347201342	-3.916145748	-2.75963209	-0.791525081	-3.549428735	-4.641378702	-2.005127251	-2.123057768	-5.179976743	-2.00991467
-4.974407182	-4.048265732	-2.955908073	-5.433865034	-0.800908745	-5.130605285	-2.835966706	-0.39750269	-4.127476111	-4.380015648	-3.477807575	-3.67586466	-5.002576416	-2.525929452
-4.640354397	-3.586608345	-2.968925197	-5.057976281	-0.262290496	-5.033168513	-2.712630332	-0.317356501	-4.031656155	-3.907205434	-3.096041005	-4.063010871	-4.896323038	-2.200134494
-4.877697878	-3.436637405	-3.362189614	-4.898780973	-0.341536974	-5.2714674	-2.228706436	-0.276405649	-4.106705148	-4.083077019	-2.916924109	-3.605290813	-5.189810709	-2.376659456
-4.985666472	-3.55887516	-3.321979974	-5.155256133	-0.289440889	-5.162973168	-2.460363176	-0.731530572	-3.931517191	-4.113770913	-3.38295274	-3.434923023	-5.718677212	-2.52503673
-4.97010257	-3.807191541	-3.531711336	-5.507571987	-0.570120034	-5.319540261	-2.906337615	-0.701224726	-4.656311156	-4.215966573	-3.317938382	-4.254006126	-5.234743054	-2.615510778
-4.777090574	-3.197452434	-3.208000994	-5.201205523	-0.284270983	-5.108098115	-2.846369339	-0.554643603	-4.528417973	-3.953867202	-3.400120285	-4.434531978	-4.952034284	-2.29492798
-4.906803344	-3.648661712	-3.452821671	-4.889685279	-0.467129353	-5.448381073	-2.26188567	-0.564549785	-4.14220168	-4.080396511	-3.337704553	-3.666290429	-5.007894791	-2.469897223
-4.86237715	-3.637418379	-3.269210213	-4.745678768	-0.571742404	-5.364331639	-2.175598388	-0.169720047	-4.034042677	-4.230336759	-3.360556571	-3.707438471	-5.212251142	-2.366388783
-5.378787428	-3.85973044	-3.389797142	-5.109100833	-0.760678558	-5.197962586	-1.926166137	-0.643607479	-3.986632044	-4.594314916	-3.578867037	-3.307560785	-5.400488961	-2.126911109
-5.085491339	-3.697394127	-3.407854184	-5.841714209	-0.962788286	-5.925952023	-2.857059267	-0.849352728	-4.159759239	-4.423098907	-3.540805645	-4.085727539	-5.619161811	-2.538025585
-5.246647683	-3.724365974	-3.087783828	-5.354212222	-0.933674496	-5.537903175	-2.796358389	-0.157227275	-4.507621762	-4.73953782	-3.623801336	-2.980688272	-5.653254291	-2.134327559
-5.209387574	-3.995233375	-3.6137316	-4.93543577	-0.51055926	-5.692058733	-2.65110872	-0.492968402	-4.095244446	-4.534627047	-3.542227731	-3.787574733	-5.563074501	-2.690816409
-5.321838525	-3.397893149	-3.243578543	-4.679450177	0.006183217	-5.115614056	-2.680747183	-0.466456568	-4.421701594	-4.237328697	-3.250288459	-4.068317719	-6.244665463	-2.313282029
-4.986650734	-3.988276777	-3.428515988	-4.90172967	-0.849826554	-5.105854098	-1.778765756	-0.298608038	-4.043386287	-4.266653693	-3.639469677	-3.369633607	-4.935112766	-2.117351422
-5.146717581	-3.534966771	-3.444355864	-4.654133375	-0.240773811	-5.077874262	-2.472057901	-0.875633722	-4.009519213	-4.191283611	-3.039499	-3.496991925	-5.594996841	-2.210976756

-4.548719446	-2.113722076	-2.030422187	-4.990057372	-0.185263965	-3.922802437	-2.748054162	-0.552770618	-3.140624752	-4.17614953	-1.99463663	-2.192339006	-5.22633307	-1.946022121
-4.931036451	-2.092878254	-1.847069338	-5.437396027	-0.361530613	-3.799736898	-2.813339199	-0.859581907	-3.341158632	-4.301323662	-1.96592796	-2.136939548	-4.933779033	-2.098303003
-4.835645159	-2.245447371	-2.189091496	-5.011378818	-0.265000779	-4.128290847	-2.73117361	-0.687865035	-3.612421875	-4.307869128	-1.959693429	-2.316554181	-4.944491552	-2.024500521
-4.824341162	-2.44284245	-2.314734012	-5.222178686	-0.387296338	-4.077680777	-2.893932173	-0.659859726	-3.701920421	-4.401621745	-2.307546886	-2.14767131	-5.116579614	-2.104333298
-4.973254988	-2.686580788	-2.531982493	-5.678428192	-0.641445359	-4.514155236	-2.657926885	-0.706696281	-3.885869932	-4.507824663	-2.542822477	-3.061316695	-5.092627329	-2.168594523

hsa-miR-6780b-	hsa-miR-6780b-	hsa-miR-6781-5	hsa-miR-6782-5	hsa-miR-6784-3	hsa-miR-6785-3	hsa-miR-6785-5	hsa-miR-6786-3	hsa-miR-6787-5	hsa-miR-6789-5	hsa-miR-6790-3	hsa-miR-6790-5	hsa-miR-6791-5	hsa-miR-6792-3
-4.263914689	-0.588875706	2.126678376	-3.031182737	-4.663760381	-4.623954129	-1.101474789	-4.275721671	-1.170045751	-0.987990221	-4.423601446	-4.476737281	-0.483724306	-3.722702747
-4.517206204	-0.327446611	2.033066845	-2.902320825	-4.928017408	-4.955903143	-1.049644557	-4.383943065	-0.690199388	-0.435461372	-4.052940902	-3.85501036	0.313078364	-3.838089272
-4.468635742	0.074988503	1.972994952	-2.832012048	-4.869531229	-4.785399136	-1.311899669	-4.47254223	-0.88797039	-0.813684977	-3.956608984	-4.235759144	0.099415081	-3.889088676
-4.507299356	0.181907576	1.974615272	-2.439853544	-4.651759897	-4.887971274	-1.171080519	-4.460095339	-0.92966387	-0.71461409	-3.884648205	-4.003596513	0.260994391	-3.768130825
-4.277506556	0.144386028	2.120796779	-3.027608489	-4.472166898	-4.465067604	-1.301595602	-4.096266292	-0.892021098	-0.76726638	-4.199263702	-4.237139005	-0.26113106	-3.477138367
-4.558601391	0.013735845	1.894783935	-3.136600575	-4.638147198	-4.6582801	-1.082751777	-4.418204941	-0.982545451	-0.537858302	-4.299569517	-4.340058036	-0.043837797	-3.697469457
-4.331900594	0.022075911	2.304632408	-2.521085396	-4.410160372	-4.579393915	-1.046037062	-4.241322571	-0.717007916	-0.609677293	-3.889410904	-4.257984876	0.134475808	-3.651776819
-4.597133643	0.140754575	1.871431803	-3.161222756	-4.91613899	-4.757522776	-1.021668314	-4.458179129	-0.907103353	-0.761008828	-3.767874852	-4.28613031	0.317484666	-3.925718
-4.592539128	-0.023888004	1.873765483	-3.461675556	-4.8090298	-4.865044665	-1.365874855	-4.597914416	-0.878534618	-0.667408836	-4.073255664	-4.335648405	0.129723833	-3.976503226
-4.311217836	-0.22135614	2.052244656	-3.439659142	-4.747172385	-4.945634438	-0.937624959	-4.501410843	-0.949290292	-0.375314632	-3.947807335	-4.078809512	0.190435301	-3.795142511
-5.002234231	-0.113774294	1.879392171	-3.14828344	-4.839909043	-4.878081543	-0.945783841	-4.607303576	-0.89868472	-0.884473514	-4.01253887	-4.392682399	0.129641725	-4.24212161
-5.182914217	-0.681318535	1.842241838	-2.99036486	-5.071544619	-5.292224061	-0.833148275	-4.885149546	-0.870527865	-0.842257987	-4.317605109	-3.974776576	0.04421812	-4.35929452
-5.503950331	-0.220337673	1.479460156	-3.194834957	-5.264849571	-5.367379413	-1.348288186	-5.089033501	-1.101613707	-1.182906583	-4.311721126	-4.228327712	-0.025615081	-4.565858158
-4.947104221	-0.279246015	1.769319985	-3.107775466	-4.914969642	-4.938469592	-0.988843505	-4.756956255	-1.068989298	-0.840020914	-4.092224156	-4.361769146	-0.01445942	-4.414981492
-5.063938099	-0.286183101	1.90112378	-3.353016571	-5.063250601	-5.160667125	-0.770418156	-4.805445792	-0.954500179	-0.519993807	-4.272911581	-4.250667483	-0.185048858	-4.382427191
-4.931574089	-0.53691068	1.860117701	-3.093959794	-5.059674834	-5.043125705	-1.198652222	-4.757729782	-0.9915509	-0.759973607	-3.859815882	-4.17581424	0.215527333	-4.42174655
-5.036830786	-0.097003818	1.901546005	-3.42476664	-4.913893127	-4.93448259	-0.738793101	-4.71641448	-1.038869418	-0.554050193	-4.236200409	-4.414286719	-0.099040752	-4.428304999
-5.131771655	-0.743213212	1.86719039	-3.205316795	-5.445517164	-5.08468743	-1.142934077	-5.093678587	-0.86402578	-0.658243229	-3.959331496	-4.327991652	0.199968434	-4.74885176
-4.841881718	-0.276846189	1.804809621	-2.938591772	-4.88934211	-5.025805429	-1.168447142	-4.818607959	-1.115225769	-0.961576505	-4.459661409	-4.386655789	-0.227345027	-4.504035096
-5.061807521	-0.045790925	1.837192601	-3.169589209	-4.835605017	-5.018596577	-0.833002633	-4.885361905	-0.975611843	-0.626058008	-3.911170555	-4.295693657	0.103072917	-4.289659637
-5.175090219	-0.40661096	1.994512969	-3.297197433	-4.986767086	-4.895696646	-0.726596439	-4.842540888	-0.939789495	-0.563603053	-4.28012949	-4.464443944	-0.097906916	-4.424043233
-5.121247329	-0.417430057	1.970277285	-3.211870023	-5.171257066	-5.034251864	-0.830861185	-4.771479823	-1.039516992	-0.525261178	-4.36147934	-4.444502051	0.079746537	-4.479537636
-4.902606138	-0.0063388	1.893407718	-3.12788821	-4.724680184	-4.782197411	-1.188135031	-4.647031951	-0.91441238	-0.741953373	-4.178583371	-4.247888651	0.168934096	-4.120600437
-4.447533146	-0.106521155	2.222242264	-2.787959985	-4.432283519	-4.622804947	-0.542481921	-4.408553534	-0.743337145	-0.75324868	-4.146505267	-4.052072739	0.020807731	-3.892278566
-5.003995628	0.217990111	1.838957112	-3.231498864	-5.274205798	-4.965127986	-0.935840434	-4.733299304	-0.922966235	-0.693224136	-4.086160547	-4.331712804	0.036536457	-4.390932819
-4.511849901	0.202181963	2.155704273	-3.229954019	-4.46219671	-4.404995111	-0.611606242	-4.391925289	-1.098855597	-0.926308337	-4.307990284	-4.632001329	-0.298854105	-3.960172369
-5.205265605	-0.282226469	1.602802579	-3.121612129	-5.230866435	-5.415018947	-1.006490451	-4.842378259	-0.986978148	-1.213232504	-4.497788439	-4.228221856	0.016054133	-4.620059269
-5.025348131	-0.167371541	1.842883929	-2.816694997	-4.635895924	-5.069800016	-0.605452104	-4.545276383	-1.006896987	-1.046968946	-4.640185836	-4.258279506	-0.454694693	-4.076707799
-4.744599149	0.1217025	1.788378579	-3.435506727	-5.035011835	-4.955949132	-0.703406384	-4.586506355	-1.106594546	-0.267190188	-4.685201871	-4.323853184	-0.63481196	-4.03549066
-4.731059608	-0.183872589	2.004684096	-3.278273761	-4.920512561	-4.962421601	-0.910022092	-4.52541218	-0.664811134	-0.735578142	-4.425425476	-4.111987346	0.174557124	-4.034290847
-4.799121604	-0.273218604	2.079219062	-3.241794835	-5.054524801	-4.979357263	-0.878332931	-4.678755897	-0.559444334	-0.771696454	-4.161224493	-4.006017007	0.476547951	-4.107412713
-5.550346073	-1.068598077	1.086067885	-2.88711259	-5.725612956	-5.86633444	-0.68585528	-5.402170949	-1.633501625	-1.517722897	-5.802295134	-4.537784128	-1.099209316	-4.802575374
-5.014533021	-0.998671774	1.541741287	-2.920112289	-5.214713308	-5.408369381	-0.434861638	-4.630457234	-0.795997957	-0.27795525	-3.875311025	-3.546786577	0.327993604	-4.359171671
-4.981550199	-1.193345619	1.842744163	-3.718359113	-5.140815556	-5.314561716	-1.060795225	-4.669027989	-0.491028614	0.01339847	-3.129245274	-3.3754819	0.791735994	-4.234787586
-5.393911123	-0.471728876	1.667976193	-3.634552509	-5.362831107	-5.367543281	-1.398148832	-5.164822314	-1.258703213	-0.5436344	-4.083131652	-4.414838563	-0.065014871	-4.922313948
-5.405084489	-0.333901932	2.108534558	-3.343923331	-4.779364509	-4.846419142	-1.036112197	-4.549172304	-0.54624103	0.106881114	-3.8282987	-3.704277442	0.40443259	-4.130846919
-6.232232023	-1.034913514	1.464228153	-4.334219611	-5.689621478	-4.783709126	-0.174167908	-6.328535464	-0.821691412	-0.339629066	-4.058072776	-4.111085687	0.051544201	-6.22850594
-5.239020193	-0.647199446	1.821706525	-3.6313262	-5.186309639	-5.158675491	-1.470943786	-4.729274973	-0.8000385	-0.20070222	-3.689881091	-3.657289948	0.523699165	-4.691299591
-5.083974506	-0.065802635	1.761775915	-3.509678046	-5.064557832	-5.162010118	-1.86710256	-4.710957974	-1.134143908	-0.414781643	-3.653823975	-3.914818342	0.305119517	-4.27827685
-5.379218851	-1.757810135	1.817445661	-4.17073799	-4.918195742	-4.669417042	-0.160108757	-4.497668924	-0.649242728	-0.151815348	-3.95268708	-3.770528487	-0.147348858	-4.575301803
-5.118685728	-0.368877036	1.909956364	-3.737047418	-5.019583935	-4.966494092	-1.468295181	-4.806237206	-1.004783467	-0.156974766	-4.340787205	-4.042566265	-0.202241512	-4.387916493
-5.350409036	-0.282135231	1.596754849	-3.699540756	-5.209269747	-5.147703794	-1.52083855	-4.990489355	-0.70946586	-0.434428006	-2.803875292	-3.558609721	0.615173776	-4.50047421
-5.810229801	-0.328716289	1.097022213	-2.072322271	-5.625088806	-5.484015235	-2.002955439	-5.236175215	-0.906187619	-0.997410871	-3.643047263	-3.897330772	0.173658138	-4.806367483
-4.36118924	-0.69616509	2.101859296	-3.167286032	-4.020983371	-3.924868766	-0.633597118	-4.024186667	-1.166781912	-0.915796274	-4.423577143	-4.309372799	-0.392541704	-3.783694647
-5.465547665	-0.467748744	1.63507176	-4.347692964	-5.55946704	-5.487005225	-1.504566864	-5.001787055	-0.836018052	-0.309368782	-3.151251715	-3.891534233	0.428282406	-4.723590669
-5.325246139	-0.426890232	1.691525765	-3.968457645	-5.403923474	-5.560499062	-1.548399139	-5.138772898	-0.99678389	-0.349645467	-3.430590207	-4.052645485	0.429059154	-4.626558009
-4.798538649	0.01208262	2.21873612	-3.104758538	-4.8698825	-4.933370936	-1.67617476	-4.784723288	-1.231113491	-0.425516751	-3.154941098	-4.237406121	0.459256214	-4.352452082

-5.089124743	-0.131755645	1.694341684	-3.601681559	-5.381446079	-5.432126245	-1.50503273	-4.933139184	-0.977033197	-0.329581345	-3.26090852	-3.754333448	0.666884368	-4.567206245
-5.917037699	-0.746757213	1.665895043	-3.881763874	-5.417203249	-5.334950116	-0.392198512	-4.994859761	-0.527458648	0.05738158	-3.415720397	-3.492448099	0.672383434	-4.510127527
-5.287352786	-0.579774762	2.181857612	-2.913679264	-5.024112477	-5.234574421	-0.291735684	-4.569054064	-0.706837998	0.140886915	-3.313022206	-3.465909314	0.588397353	-4.571323479
-4.523475821	-0.094506302	1.859676405	-2.48180635	-4.198870247	-4.09966136	-0.515498115	-4.183357232	-0.940718273	-0.92319609	-4.127785084	-3.924822658	0.270055776	-3.852831682
-4.834233809	-0.171263269	1.840742582	-3.578762441	-4.713075879	-4.722550398	-1.147506108	-4.656026427	-1.291269813	-0.594438714	-4.511747437	-4.078422272	-0.53517896	-4.234374336
-5.590727775	-1.230374776	1.460790806	-3.066367424	-5.171401243	-5.302630592	0.098691631	-3.84329266	-0.130485745	-0.22808844	-3.182318278	-3.187766749	0.509417082	-4.431019095
-5.832467367	-0.142805474	1.804854113	-3.486392163	-4.392541505	-4.889509094	0.98954779	-5.142059218	0.0652483	0.177327507	-3.773508784	-4.275850844	0.577836787	-4.201581496
-5.005725978	-0.429856801	1.762169274	-3.776673086	-4.890935393	-4.821901316	-0.420052567	-4.745935861	-1.142618615	-0.608996943	-4.109647076	-4.447369205	-0.250028173	-4.250023199
-5.254746727	-0.810072851	1.802884821	-3.764647726	-5.199614357	-5.209398302	-0.460825294	-4.743303697	-0.678050423	-0.060934549	-3.023345898	-3.399058576	0.66228716	-4.362306514
-5.318155281	-0.633267624	1.744786156	-3.615636681	-5.382517121	-5.328387868	-1.311414108	-4.817525572	-0.732770827	-0.259127036	-3.184785897	-3.537140501	0.559274498	-4.434597052
-5.686362843	-0.279795779	1.017222438	-3.207776017	-6.078182302	-5.443373379	-1.158314644	-5.718557477	-1.217416862	-0.947295136	-3.469329753	-3.863462052	-0.258943245	-5.037895184
-5.29915752	-0.023711053	1.413136968	-3.349173843	-5.381395529	-5.467271861	-1.23269377	-5.102428675	-1.013166633	-0.731226045	-3.019983936	-3.809956307	0.168275688	-4.55011276
-5.407758484	-1.023701632	1.60224448	-3.824056105	-5.370394933	-5.314258749	-1.379419777	-4.943389661	-1.116711451	-0.55131869	-4.409190674	-4.1810507	-0.48966776	-4.550432109
-5.60573756	-1.30671703	1.243490899	-3.876023532	-5.640100133	-5.247974146	-1.122306088	-5.247066065	-0.847496794	-0.718005741	-3.314180065	-3.65953853	0.100895519	-5.069672296
-6.962994376	-1.205067296	1.338105976	-3.23785578	-4.961334996	-5.016668783	0.105255149	-5.979144346	-0.379275099	-0.208593912	-3.458395156	-3.553161802	0.199961087	-5.15740633
-5.934675353	-0.643333292	1.789601085	-3.766083633	-5.732581107	-5.176646837	-0.173132968	-5.04635319	-0.265317246	-0.059002639	-3.45307284	-3.73990131	0.334028075	-4.698915011
-5.383823082	-0.301440465	1.562137434	-3.8610002	-5.04498174	-5.065666686	-1.359970112	-4.928919861	-0.908350912	-0.386451282	-3.036648045	-3.974578761	0.326704731	-4.648820935
-4.387449888	-0.999805455	2.004310799	-3.168930366	-4.545024859	-4.646577043	-1.197145064	-4.210973253	-1.241497244	-0.607031385	-4.210643753	-3.958960082	-0.237584713	-3.808953397
-5.018173608	-0.27984405	2.117509261	-2.919502029	-4.875340482	-4.887707941	-1.515769031	-4.622160794	-0.980498593	-0.045501512	-3.270959491	-3.609979986	0.514322845	-4.214660679
-5.727100075	-0.234075585	1.522259348	-3.909382431	-5.604665807	-5.574425081	-1.322926421	-5.345095799	-0.924247915	-0.682933909	-3.689131429	-4.121222641	0.108166812	-4.889782304
-4.893541032	0.388685866	1.873466399	-3.628726246	-5.034524705	-5.101509077	-0.861516889	-4.481105208	-1.006530946	-0.386830026	-4.286231831	-4.091949941	0.110089739	-4.161824918
-5.036079373	0.049502906	0.977704464	-3.1245121	-5.579864513	-5.655520574	-0.52365906	-5.155269244	-0.589402912	-1.136348026	-4.68725207	-4.554893214	-0.145476629	-4.570431566
-5.58503911	-0.186654589	1.614241997	-3.359190178	-5.645082353	-5.798077387	-1.461309768	-5.551679571	-1.222623853	-1.040161259	-4.596692048	-4.315606346	-0.069628859	-4.860651882
-6.513920183	-0.996143035	1.453715065	-3.578578011	-6.989843434	-7.392537602	-0.170198724	-6.140961875	-0.780146466	-0.384238892	-4.613176208	-3.653388158	0.410493568	-5.173974478
-5.472263304	-0.705397196	1.595823026	-2.999815896	-5.614557958	-5.760442664	-0.956832119	-5.328770926	-1.198132311	-0.637378241	-5.194463725	-3.675521592	-0.138682267	-4.613370527
-5.737686774	0.325303695	1.15860724	-3.603325783	-5.175744118	-5.095942256	-0.67653346	-5.523230337	-0.462385063	-0.793247747	-4.004168015	-4.520058218	0.02813354	-4.88916369
-4.924008197	-0.195759687	1.199891257	-3.348864082	-5.412733001	-5.5083535	-0.00527364	-5.002954712	-0.175216008	-0.714635605	-5.631221772	-4.374292114	-0.480125044	-4.506472393
-5.068330207	-0.589182947	1.548779531	-3.724566044	-5.196207656	-5.239525018	0.072848345	-4.823441742	-0.294670314	-0.458001054	-5.272087464	-4.638519925	-0.58656561	-4.314081916
-5.247880173	0.191793971	1.23760294	-3.091031137	-5.415324996	-5.391604696	-0.370479862	-5.010718059	-0.556529106	-0.855633419	-4.954536991	-4.221675077	-0.203243349	-4.568880558
-5.213167513	0.436457529	1.275041792	-3.346586648	-5.130290138	-5.096128212	-0.75877099	-5.113234019	-0.482383345	-0.928104042	-4.104906648	-4.699258839	0.22947195	-4.504164868
-5.534202711	0.376184118	1.047519749	-3.0931448	-5.562255612	-5.283408499	-0.108810874	-5.223152092	-0.118658085	-0.673435738	-4.92541974	-4.121389475	0.039307749	-5.032701121
-5.236566236	0.018046407	1.474784136	-3.276200689	-5.598168466	-4.930743605	0.090395394	-5.448919701	-0.184789966	-0.387352909	-4.805319271	-4.143413389	-0.266487292	-4.599345188
-5.193180131	0.039006881	1.088118371	-2.870750799	-5.571049771	-5.372476457	-0.323486818	-5.278074428	-0.166641234	-1.083060514	-4.550784303	-4.381656189	-0.167151238	-4.667705321
-4.990030491	0.098194452	1.07444462	-2.69130895	-5.267623137	-5.473069243	-0.120834197	-4.800113928	-0.236255816	-0.831506952	-4.996083296	-4.41656776	-0.099561634	-4.370061589
-5.377395482	-0.734574593	1.561748694	-3.816372518	-5.830041596	-5.402946081	-1.708376707	-4.959079015	-1.10816895	-0.55195898	-3.641140581	-4.075780812	0.079511812	-4.649489618
-4.979852552	-0.814279101	2.118102204	-3.26689881	-4.901746684	-4.777641034	-0.911701328	-4.593835836	-0.803583832	0.051522517	-3.168306079	-3.456402774	0.785105616	-4.182567176
-5.122941845	0.065711758	1.494436908	-3.568290593	-5.145608963	-4.873278456	-0.809216671	-4.92211133	-0.567784918	-0.366701427	-3.279973579	-3.245225869	0.67378177	-4.401726269
-5.393623864	0.110227727	1.438264916	-3.822975701	-5.553051667	-5.409893929	-0.958988896	-4.867122034	-0.646533224	-0.460746484	-3.378880123	-3.575083528	0.371796594	-4.696959741
-5.452157795	-0.873080638	1.619101845	-4.215039821	-5.193681492	-5.158207465	-1.168163819	-4.801559639	-0.856432677	-0.005790915	-3.383602331	-3.881328247	0.393292556	-4.375551649
-5.300860871	-0.718720433	1.376920053	-4.256230435	-4.971231688	-5.182252409	-0.248855631	-4.760580612	-0.871749274	-0.026144032	-4.147630263	-3.28704812	-0.028344805	-4.257235964
-5.660954481	-0.272458785	1.379703036	-3.833809267	-5.065579279	-5.324041967	-1.024287965	-4.931261513	-0.920757354	-0.424070365	-3.149484542	-3.743269716	0.419107168	-4.51419377
-5.330338941	0.357320803	1.503603831	-3.533464192	-5.406550343	-5.460402607	-1.407518555	-5.077277368	-1.173594759	-0.743772598	-3.156260429	-3.618631545	0.360742405	-4.670872501
-5.429976923	-0.305359055	1.210457864	-3.379892716	-5.798739192	-5.682115669	-1.575940753	-5.44448479	-0.993721143	-0.839214395	-3.323258987	-3.706396232	0.133854215	-4.909553774
-5.471967331	-1.175848109	1.323146136	-3.71833227	-5.265181487	-5.334349115	-0.581596499	-5.181959486	-1.370930534	-1.101160664	-4.404533206	-4.245606938	-0.712186468	-4.74755944
-5.707552483	-1.020058401	1.734477627	-3.402483081	-5.540174153	-5.685896162	-0.899776859	-5.375515201	-1.177384935	-0.365105711	-4.222074143	-3.682192307	-0.323358565	-5.036462447
-5.712014929	-0.674845028	1.50785888	-4.008913572	-6.068762798	-5.627737446	-1.047070694	-5.517573588	-0.808048619	-0.25257234	-3.252495401	-3.845005203	0.285213969	-5.098516492
-6.248193013	-1.062796851	1.866898453	-3.608427317	-5.568116912	-4.723954281	-0.080223427	-4.739917533	-0.501214678	0.097018057	-3.451120566	-3.588353663	0.394225098	-5.827441376
-5.451726421	-0.330312735	1.494882273	-3.179317251	-5.513001876	-5.305813222	-2.0242722	-5.09145157	-1.021287774	-0.515881738	-2.961992233	-3.374830849	0.544688493	-4.668236437
-5.30355826	-0.440686365	1.481857822	-3.803965549	-5.51938246	-5.500255209	-0.555066555	-5.165389901	-0.541806191	-0.39653758	-3.588895592	-3.6309763	0.170989028	-4.441461719

-4.890319544	0.411041287	1.333797649	-2.97876964	-5.378908586	-5.170321227	0.068210492	-4.847718098	0.027209735	-0.583481398	-4.733405715	-4.605888001	-0.009299343	-4.363507863
-5.182980451	0.300244397	0.95210997	-3.009680813	-5.237211413	-5.237374494	-0.122992194	-5.241165668	0.060336175	-0.950759102	-4.941292681	-4.232394262	-0.033552886	-4.479016801
-5.101550154	0.580131922	1.100195095	-3.029016176	-5.103762921	-5.044252221	-0.165891543	-4.891298173	-0.052886057	-0.707333693	-4.559972502	-4.297333817	0.152753482	-4.506109514
-5.022624966	0.361002682	1.236347156	-3.030572629	-5.395665989	-5.214327995	-0.222034125	-5.231089933	-0.295864441	-0.625261424	-4.883839645	-4.190824058	0.050452588	-4.564531801
-5.368824766	0.351551074	1.021272888	-3.143310374	-5.387196732	-5.622812811	-0.604686578	-5.126587822	-0.618052927	-1.148709693	-4.759803098	-4.807281655	-0.180536029	-4.498778089

hsa-miR-6792-5	hsa-miR-6794-3	hsa-miR-6794-5	hsa-miR-6795-3	hsa-miR-6795-5	hsa-miR-6796-3	hsa-miR-6796-5	hsa-miR-6797-3	hsa-miR-6797-5	hsa-miR-6798-3	hsa-miR-6798-5	hsa-miR-6799-5	hsa-miR-6800-3	hsa-miR-6800-5
-4.153793256	-4.756071075	-1.003054862	-4.791552892	-3.045562914	-3.642277155	-4.473949073	-4.36788424	-3.077728078	-3.71292389	0.555564491	0.79843655	-3.540092544	-0.284798949
-4.030093813	-5.083353478	-1.124083445	-5.240230503	-2.937287988	-3.885128174	-4.539154882	-4.608826327	-3.569529724	-4.09922015	1.217086018	0.633275529	-3.829737295	-0.351541422
-4.237781268	-5.09838997	-1.26525406	-4.83010381	-3.025166045	-3.740095818	-4.601969078	-4.397593925	-3.499551319	-3.919587231	0.745213258	0.686778215	-3.62204442	-0.801441621
-4.036196316	-4.812199908	-1.262859371	-4.834401274	-2.978094275	-3.591698537	-4.53579536	-4.36099133	-3.747945897	-3.689519978	0.988569981	1.230315713	-3.541012904	-0.704264198
-3.836898876	-4.53535939	-1.247657486	-4.345457017	-2.891664406	-3.375125669	-4.395364232	-4.108079555	-3.257720659	-3.483384183	0.822569537	0.897493477	-3.216123508	-0.26408674
-3.84411463	-4.847089931	-1.056180515	-4.619281019	-3.07667302	-3.602349231	-4.52331765	-4.427204781	-3.520787716	-3.791119253	1.211220925	0.599849334	-3.555971566	-0.190852316
-3.867652511	-4.539768421	-1.082474928	-4.518954751	-2.993233451	-3.315964663	-4.512936313	-4.153457788	-3.26935388	-3.451269255	1.104994514	1.224189291	-3.339062416	-0.399056068
-4.058995733	-5.097327983	-1.276143577	-5.029308201	-3.201590309	-3.857155655	-4.579008838	-4.762021291	-3.753760896	-4.113220567	1.275713914	0.566529697	-3.758113871	-0.56216902
-4.176000856	-5.07574144	-1.310925109	-4.964775091	-3.2175031	-3.814012929	-4.741963807	-4.759683671	-3.600290874	-4.0043186	0.919420558	0.527985062	-3.694994041	-0.425112229
-3.843438837	-5.01361054	-1.240916972	-4.711813817	-2.994220641	-3.644635233	-4.399554387	-4.616735544	-3.556167818	-3.781200173	1.282053231	0.793184603	-3.610128602	-0.453223687
-4.159889953	-5.458061942	-1.449826992	-4.748697932	-3.038208964	-3.82245883	-4.908086817	-4.745116133	-3.92869704	-3.794512014	1.204057381	0.908986876	-3.770727499	-0.65231977
-4.218840932	-5.084193171	-1.210011529	-5.569260537	-2.937867575	-4.11944299	-5.132632535	-4.759033572	-3.412163047	-4.239075124	0.468944784	0.847146937	-3.940878283	-0.732672273
-4.263194661	-5.508814348	-1.388875126	-5.397368513	-3.264934137	-4.277579723	-5.07220983	-4.968411018	-3.584091711	-4.347595866	0.557491372	0.886048706	-4.066242494	-0.957425866
-4.062358505	-5.167109643	-1.380479469	-4.860621824	-3.182109829	-3.823492999	-5.060270997	-4.475512067	-3.826270075	-3.89271884	1.093515616	0.81839857	-3.884887602	-0.646603968
-4.12443685	-5.442998019	-1.393932111	-5.290234886	-3.363519965	-4.050049575	-5.299602514	-4.8138978	-3.926296811	-4.153508008	1.04980742	0.269520244	-3.944948751	-0.357023364
-4.111582846	-5.478373724	-1.439430542	-5.222387985	-3.200450263	-4.00555212	-5.057295523	-4.872649103	-3.85509593	-4.23527632	0.842611064	0.406786488	-3.984719046	-0.694677163
-3.799663778	-5.249488901	-1.263614225	-5.266949673	-3.529369525	-3.912066412	-5.072265806	-4.92763634	-3.777490896	-4.145761856	0.777206206	0.064308924	-3.928395288	-0.299764272
-4.525189759	-5.791782966	-1.559379859	-5.144739851	-3.376140641	-4.094913448	-5.236104257	-4.840636385	-3.844177878	-4.308408487	0.888034816	0.545531652	-4.274065828	-1.012893702
-4.214556555	-5.265962809	-1.438911981	-4.94307909	-3.243076516	-3.849248367	-5.131744216	-4.501240123	-3.473478724	-4.067519163	0.600041839	0.84257825	-3.812906067	-0.693399086
-4.042909271	-5.430460523	-1.352175935	-5.049368764	-3.29778857	-3.803427432	-5.303672097	-4.774507865	-3.689139754	-4.033994761	0.913767996	0.659204538	-3.850482397	-0.644758724
-4.086488076	-5.647823698	-1.273787848	-5.16881784	-3.365871219	-4.058690755	-5.359047627	-4.694115375	-3.735175266	-4.190657329	1.056644667	0.341815546	-3.992481818	-0.398429754
-4.241963248	-5.581365706	-1.358844707	-5.171177449	-3.574820011	-4.181638723	-5.268166858	-4.822495933	-4.095288543	-4.272970393	1.018684822	0.043695832	-4.028383437	-0.424075504
-4.310497683	-5.149767692	-1.460084376	-4.944002211	-3.074967931	-3.836172743	-5.326627284	-4.508925696	-3.619940818	-3.97113632	0.801209282	0.792621852	-3.793472424	-0.77986568
-4.140165649	-4.705769488	-1.24501926	-4.454221617	-2.785505837	-3.463516141	-4.943128631	-4.178892779	-2.916797488	-3.403134442	0.378312804	1.372657348	-3.40552972	-0.497816007
-4.010494304	-5.377925079	-1.454917358	-5.214431712	-3.301806329	-4.051583249	-5.187362583	-4.723397432	-4.230533096	-4.157956276	1.071530857	0.137289911	-3.993489038	-0.617145529
-4.062361973	-4.547261709	-1.219865684	-4.453067855	-3.165862048	-3.392306202	-5.290462709	-4.173372944	-3.020979616	-3.407798334	0.430894386	0.744027708	-3.304070087	-0.646910118
-4.268763987	-5.534438781	-1.443469257	-5.615335307	-3.167113231	-4.341680849	-5.150904858	-5.045025463	-3.943492291	-4.385545477	0.908223885	0.763832102	-4.011652767	-0.840440839
-4.231666949	-4.683956439	-1.331600392	-4.963469782	-2.947304606	-3.708434267	-4.704574646	-4.72809347	-3.336522264	-3.956935451	0.281649458	0.892059412	-3.675529097	-0.699008783
-4.228378939	-4.614326098	-1.035193067	-4.928218501	-3.080934495	-4.197588477	-4.669413118	-4.748925621	-3.965068287	-4.363168578	0.726454414	0.078537324	-3.801490093	-0.728758277
-4.017105279	-4.914224559	-1.442997798	-5.010869114	-2.94721773	-3.894883355	-4.793885664	-4.598094246	-3.697380208	-4.08476645	0.948698753	0.917303734	-3.713980569	-0.840501743
-3.798635678	-4.953728567	-1.514462882	-4.927437675	-2.779265045	-3.792083652	-5.010542289	-4.873557377	-3.587312938	-3.896779727	0.895215315	1.236788502	-3.83557084	-0.863652696
-4.265401075	-5.40026219	-1.07600162	-5.746316832	-3.061853283	-4.430997043	-5.621383132	-5.252586561	-3.069273767	-4.418767417	0.015123967	0.260174734	-4.422389831	-0.890589058
-3.613452123	-5.201014245	-1.127918753	-5.472228057	-3.121586028	-4.317491382	-4.537585442	-5.323028667	-3.939497944	-4.536721264	1.528032009	-0.027765656	-4.17493003	-0.729948571
-2.432036967	-5.35895321	-1.154823828	-5.375387476	-3.078099374	-4.222313044	-4.606149976	-5.098249764	-4.084704505	-4.478677808	1.757756807	0.763778507	-4.157973496	-0.634471506
-3.951095896	-5.66666554	-1.429907784	-5.298807332	-3.73353494	-4.281746973	-5.369427699	-5.124579588	-4.401129135	-4.501889863	0.94171243	-0.023666358	-4.282809748	-0.997533277
-3.562995451	-4.798647851	-1.016701017	-4.996220186	-3.411010563	-3.799097726	-4.95005402	-4.6085208	-4.082404106	-3.930459945	1.521357588	0.086980486	-3.541572354	-0.082282191
-3.605511403	-7.397514919	-1.305689916	-5.225738599	-3.928098664	-4.7552771	-6.66017529	-5.788896686	-5.837487713	-4.30425014	1.504916216	-0.282774118	-4.567974976	-0.626531294
-3.871719194	-5.401470944	-1.352081253	-5.178549967	-3.779864533	-4.087263798	-5.000640984	-4.843922941	-4.508405811	-4.274989778	1.969736762	-0.015089547	-4.22741744	-1.028695573
-3.994194013	-5.268474135	-1.450852255	-5.29706442	-3.903490882	-4.039417769	-5.029320884	-4.670136122	-4.58353129	-4.252919879	1.437376582	0.215144043	-3.883036504	-1.255993138
-3.745573729	-4.914803512	-1.150241539	-5.126215111	-3.546115301	-4.128829988	-5.031887001	-5.16204802	-4.124054879	-4.220676929	1.582453222	-0.286553894	-4.0567326	-0.523808711
-4.36419224	-5.147120214	-1.169296654	-5.048265867	-3.741926435	-3.999295893	-5.049193435	-4.673521418	-3.800865567	-4.152115331	0.933126017	-0.17437828	-3.928467868	-0.605429984
-2.916132546	-5.372605683	-1.649211048	-5.23691459	-3.811642504	-4.140774343	-5.015870317	-4.924910509	-4.329482356	-4.196692982	1.91856018	0.52096956	-4.225233524	-1.45002814
-3.207424912	-5.791411296	-0.711745731	-5.608406028	-4.093730755	-4.282657386	-5.685048552	-5.350881147	-2.507030348	-4.524196923	1.392748501	0.093103863	-4.31337863	-1.421375719
-4.169697417	-4.41969495	-0.685373813	-3.559110788	-2.870654416	-2.975197252	-5.009677024	-3.685554769	-2.263771965	-2.642014993	0.844400482	0.871216415	-3.03822552	-0.499797406
-3.204600013	-5.816018108	-1.436903568	-5.552460793	-3.910889156	-4.458701263	-5.317269211	-5.110740471	-4.541710804	-4.59029936	1.751986341	0.173384361	-4.265266728	-1.074304508
-3.882256092	-5.663128048	-1.244716468	-5.583226821	-3.819856408	-4.399271766	-5.038546866	-5.144340478	-4.530675164	-4.624714793	1.730201799	0.08211283	-4.347005998	-1.146769386
-4.692082629	-5.139386526	-1.362945318	-4.845673722	-3.566844232	-3.720913179	-4.959839168	-4.432522837	-3.98632077	-3.726431952	0.968742067	0.840258705	-3.819676666	-1.675693546

-3.771855872	-5.350038156	-1.494386169	-5.427794366	-3.604146069	-4.149552829	-5.249272951	-4.668824695	-4.150681007	-4.191439192	1.876678818	0.731813276	-4.265252291	-1.179150361
-2.879932388	-5.722821783	-0.932464715	-5.197733421	-3.556439106	-4.180681794	-5.106209765	-4.850394694	-4.210110483	-4.411500321	2.055349188	0.223902324	-4.099247489	-0.555401675
-3.961245011	-5.18288974	-0.92751064	-5.076270571	-3.421211893	-3.9285229	-4.793358277	-4.489876445	-3.934452348	-3.779760163	2.037971211	0.443840795	-3.869651607	-0.387384829
-4.353781088	-4.421559531	-0.967809816	-3.773164465	-2.852180356	-2.899096554	-4.652120521	-3.649087798	-2.260167554	-2.572309093	0.992806002	1.242037895	-3.222307372	-0.795762976
-3.717850668	-5.054442921	-1.417970785	-4.359350846	-3.647137701	-3.405991414	-5.307728397	-4.526378032	-3.541794908	-3.337944369	0.540239745	0.395269393	-3.493127488	-0.851138725
-2.859094716	-4.68184795	-1.092004545	-5.169343333	-3.075599743	-3.91821289	-4.307080506	-4.423817724	-3.875850257	-3.978421521	1.734467957	0.436464924	-4.370831624	-0.807337305
-3.168642699	-4.280223001	-0.563825294	-4.043908018	-2.724381775	-3.654962542	-4.527621221	-5.15490547	-3.083565799	-3.391617002	1.629757463	-0.134767735	-3.603575833	-0.346253066
-3.996960761	-5.052235172	-1.334363818	-4.43881035	-3.464963123	-3.750506626	-5.212060544	-4.581270629	-3.47411161	-3.602776648	0.555524105	0.251755306	-3.599384995	-0.526945603
-2.566951966	-4.835525628	-1.106263613	-5.223959441	-3.136665325	-4.075938652	-5.24346401	-4.767013964	-3.913579476	-4.038608739	2.295654973	0.728004198	-3.70675372	-0.906624761
-2.679997608	-5.470127895	-1.44493119	-5.302049027	-3.452746507	-4.141737577	-5.125111809	-5.191281416	-3.927228895	-4.311050078	1.540854861	0.639400155	-4.159938365	-1.259221598
-3.430695086	-6.110852102	-1.511389461	-5.514703741	-3.89696663	-4.887439986	-5.4633513	-5.575724221	-4.465760717	-4.739636333	1.31206862	-0.125474947	-4.683025176	-1.189780732
-3.520403433	-5.427619603	-1.401883074	-5.776453253	-3.736828613	-4.541963391	-5.370412295	-5.225087656	-4.400439648	-4.872892662	1.460761438	0.124816625	-4.102647089	-1.268097233
-4.15148708	-5.644871607	-1.412629188	-5.230100167	-3.838267052	-4.242697502	-5.094832018	-4.987736917	-4.174436563	-4.176263834	0.307001926	-0.20606986	-4.209714914	-0.803806326
-3.05523762	-5.869898234	-1.474673086	-5.478315488	-3.484941032	-4.375851324	-5.251853272	-5.343110453	-4.192407187	-4.450570053	1.64173594	0.442782129	-4.652224618	-1.599726282
-3.372854303	-5.257143244	-1.098749268	-4.306486107	-3.246311057	-4.232503467	-5.064399862	-4.780312983	-4.147093808	-3.75739379	1.497061621	0.088392442	-5.272697431	-1.255151738
-3.467775135	-5.085219376	-0.640549569	-5.466935401	-2.92048451	-4.228977367	-5.217465867	-5.189074832	-3.920738811	-4.295634313	1.759955456	0.039805997	-4.585286697	-0.665899928
-3.847366552	-5.023152813	-1.540848288	-5.03719754	-3.787038941	-4.255052979	-5.407902985	-4.82448308	-4.739727221	-4.216060912	1.767229208	-0.063906593	-3.991331622	-1.129654504
-4.301875009	-4.411861567	-1.355570555	-4.548918047	-3.405785661	-3.535705629	-4.537082416	-4.168310028	-3.458646551	-3.524859463	0.699427574	0.103438153	-3.43050965	-0.499737789
-3.51289474	-4.839954663	-1.059169227	-4.926550613	-3.134351026	-3.799132181	-4.881042086	-4.629432765	-3.782334603	-3.980989827	0.959623672	0.805640168	-3.527492977	-1.071491114
-3.450049917	-6.165307781	-1.748382529	-6.029016024	-3.827941072	-4.604189431	-5.562639144	-5.495607736	-4.443413375	-4.912965662	1.305199855	0.034743712	-4.533402994	-1.335099026
-3.79839968	-5.071310822	-1.244055257	-5.2450148	-3.584235321	-3.96181687	-5.230737023	-4.799091341	-3.707874131	-4.037655407	1.041211692	0.074949786	-3.905836907	-0.390144637
-3.430171397	-5.761557397	-1.255522892	-5.815816517	-2.841293194	-4.660132174	-4.501018897	-5.860324907	-3.690054641	-4.930865512	0.654365093	0.014437026	-4.318343664	-1.003233405
-3.922235593	-5.498193599	-1.787651894	-5.989430105	-3.239203753	-4.475372684	-5.555058089	-5.334769104	-3.857640365	-4.856812007	0.357432233	0.737669226	-4.458781326	-1.336021679
-3.5124862	-6.698886122	-1.160899135	-7.718512816	-3.23241206	-5.256245034	-5.512832199	-7.59638843	-4.048422583	-5.453997846	1.145978447	0.136412627	-5.080072619	-0.769300711
-3.994490637	-5.778553055	-1.292032856	-5.897542346	-3.253024177	-4.522633853	-5.066821466	-5.313354334	-3.986348954	-4.581318039	0.953280559	0.570369001	-4.344236869	-0.869485098
-3.481341364	-7.222671308	-1.31105705	-5.00142163	-3.192400049	-4.327181275	-4.555944024	-5.179075609	-4.083396832	-4.456691353	1.670882043	-0.224604183	-4.165247501	-0.856006379
-3.192680413	-5.774363623	-0.953201636	-5.107941248	-2.551444334	-4.755580418	-3.912121666	-5.822745148	-3.31117357	-5.017519332	0.311350536	-0.726616811	-4.333632987	-0.613496953
-3.45457326	-5.484076653	-0.983589516	-5.636848304	-2.801560079	-4.487191622	-4.324718917	-5.366708385	-3.469926175	-4.788083488	0.366208092	-0.636941995	-4.144016043	-0.375034214
-3.37695161	-5.609941424	-1.088820776	-5.639113313	-2.752048787	-4.57492834	-4.336821579	-5.218149677	-3.392770555	-4.784915455	0.893035548	-0.033141436	-4.240616273	-0.662327142
-3.623058888	-5.542411681	-1.288976894	-5.356419118	-2.957178467	-4.204083837	-4.690851957	-5.154166009	-3.841570387	-4.408602409	1.467080676	0.225286819	-4.009952328	-1.067240623
-3.263730775	-5.486607217	-1.056648985	-5.369762605	-2.382511896	-4.670228994	-3.948409546	-5.457691523	-3.193434877	-4.612883896	1.018847543	-0.397363866	-4.464490609	-0.907256794
-3.210660874	-5.312974758	-0.81761928	-5.127919191	-2.537245213	-4.096886132	-4.332171694	-5.204168171	-3.290926774	-4.377240256	1.050665343	-0.278086557	-4.133545365	-0.709383775
-3.080013662	-5.776068161	-1.069198277	-5.584135088	-2.631598807	-4.533721548	-4.143657999	-5.740826112	-3.445446895	-4.875881705	0.590326333	-0.050250298	-4.33599082	-0.924175008
-3.081164599	-5.586936568	-0.116205203	-5.459080261	-2.539508095	-4.198249061	-3.814614829	-5.451053113	-3.196177526	-4.729157464	0.739983159	-0.270165102	-4.262616255	-0.208043193
-3.72301914	-5.461971365	-1.427135174	-5.488577648	-3.858586307	-4.172415952	-4.816629275	-5.150165068	-4.198710253	-4.693251986	0.853161883	0.069233883	-4.454335379	-1.278407793
-3.153811888	-4.834648802	-1.0236273	-4.637454225	-3.402960249	-3.670399087	-4.841978422	-4.367504573	-3.979738899	-3.737621833	2.049408804	0.678262481	-3.897245647	-0.823981879
-3.22097864	-5.033140319	-1.209526287	-5.373200579	-3.316166132	-4.140888001	-5.031555001	-4.530600295	-3.916718774	-3.921622208	2.077096451	0.640907829	-4.336191836	-1.290249234
-3.749299222	-5.257412303	-1.352719432	-5.47935665	-3.651578192	-4.290962465	-4.884708082	-5.01974799	-4.565987957	-4.377949498	2.032124429	-0.015729301	-4.267197255	-1.332884834
-3.626622504	-5.195510118	-1.388025744	-5.420160964	-3.782791841	-4.25907092	-5.382155561	-5.172177181	-4.653147546	-4.277294327	1.891113126	-0.234542307	-4.151500182	-1.071151813
-3.309993713	-5.370687067	-1.071281332	-4.662677735	-3.244511269	-4.065499315	-4.966935702	-4.703236736	-3.726978502	-4.018974792	1.054911309	-0.078633838	-3.910510697	-0.899133169
-3.195163124	-5.547680101	-1.367314691	-5.655015458	-3.664811019	-4.198198831	-5.227990484	-5.052140417	-4.485554463	-4.401906923	1.950454384	0.423881874	-4.206648619	-1.232014913
-3.15302528	-5.525442732	-1.675845502	-5.694148886	-3.651215113	-4.437813208	-5.345923669	-5.159447981	-4.323825235	-4.587830921	1.565821766	0.757386202	-4.300706365	-1.619630202
-3.039154465	-6.035629161	-1.681080153	-5.746522289	-3.51290764	-4.671402152	-5.23832449	-5.400851497	-4.102649251	-4.821374647	1.095767582	0.343409224	-4.552205542	-1.515575065
-3.56857778	-5.654765939	-1.206477177	-5.082242426	-3.449037603	-4.294580441	-5.609934083	-5.29696734	-3.984923771	-4.253940735	0.595736526	-0.092036898	-4.396648906	-0.690091359
-3.979555905	-5.885300306	-1.367378867	-5.555560006	-3.847214008	-4.517233284	-5.540501312	-5.159497865	-4.290600318	-4.457885253	0.6586105	-0.065882782	-4.805343407	-0.870941665
-3.82186985	-5.414912414	-1.563352892	-5.857517752	-3.595984363	-4.492245777	-5.500798016	-5.182737428	-4.871600971	-4.895476082	1.511847229	0.24292001	-4.652471606	-1.56519731
-3.235962896	-5.532604798	-0.900323611	-4.96460913	-3.454339905	-4.300869984	-5.245953591	-4.969544846	-4.571584381	-3.956895461	1.985404945	-0.050056989	-4.128303726	-0.507433937
-3.72890685	-5.420852122	-1.838017399	-5.143659238	-3.817350335	-4.129050488	-4.877996094	-4.795440919	-4.338507454	-4.053243456	1.27343211	1.111381316	-4.328722007	-1.576781436
-3.204377387	-6.094484321	-1.44850332	-6.325524908	-3.503746454	-4.620537261	-5.11545465	-5.609120199	-4.505696315	-4.760200637	1.957541486	-0.099023052	-4.38707968	-0.80860725

-2.95059875	-5.364437548	-0.763761983	-5.492886089	-2.4315598	-4.257465772	-3.781240127	-5.432507633	-3.281040053	-4.596897201	0.970926758	-0.190091905	-4.174278024	-0.567681282
-2.810326127	-5.514815146	-0.97210605	-5.631205629	-2.377799071	-4.504399898	-3.669487167	-5.448315085	-3.225872458	-4.828272487	1.049408863	-0.332346098	-4.019856869	-1.008760204
-2.92832014	-5.638602528	-1.045684408	-5.398854414	-2.496214077	-4.420724757	-4.123224441	-5.566659527	-3.431287406	-4.626062601	1.067554391	-0.016587394	-4.05354289	-0.796915049
-3.332394056	-5.809731485	-0.943657632	-5.481341014	-2.685252919	-4.499446597	-4.215769385	-4.932282409	-3.387022818	-4.785440658	1.13770866	-0.092381623	-4.231037269	-0.812315327
-3.608834491	-5.606128262	-1.265071913	-5.871378348	-2.936549883	-4.65905438	-4.459350583	-5.516593316	-3.799373937	-4.935393351	0.946597281	0.139382097	-4.175429954	-0.928929617

hsa-miR-6801-3	hsa-miR-6802-5	hsa-miR-6803-3	hsa-miR-6804-3	hsa-miR-6805-3	hsa-miR-6806-5	hsa-miR-6808-5	hsa-miR-6810-3	hsa-miR-6810-5	hsa-miR-6812-5	hsa-miR-6813-3	hsa-miR-6813-5	hsa-miR-6815-5	hsa-miR-6816-5
-4.1975105	-0.173107056	-3.915831143	-3.551363926	-3.133919453	-3.324254615	-2.288970118	-5.052960641	-4.76388089	-3.469851325	-4.09052956	-3.298430516	-5.158637656	0.737097796
-4.585623339	-0.871255073	-4.123208218	-3.73924162	-2.672955695	-3.52691917	-2.115898619	-5.130432471	-4.665786615	-3.887493309	-4.33598493	-3.254080258	-4.664309505	1.059510223
-4.42113211	-0.52596778	-4.045330214	-3.795168061	-2.720239273	-3.809411796	-1.859613684	-4.999093532	-4.656089758	-3.699675127	-4.028879354	-3.686089268	-4.817376235	0.760446533
-4.530617471	-0.444058846	-4.110415644	-3.746775511	-2.828507678	-4.031661313	-1.794770583	-4.984950699	-4.969628793	-3.744579803	-4.225979339	-3.533252692	-4.568610742	0.76820145
-4.181561114	-0.075566575	-3.830410624	-3.474021243	-2.700309912	-3.447075562	-1.762171281	-4.732714669	-4.92587992	-3.48128871	-3.733571573	-3.380280635	-4.658411325	1.015004252
-4.3239654	-0.493417882	-4.00908825	-3.456646027	-1.823106364	-3.175231385	-1.953411352	-4.992813251	-4.633400586	-3.686909478	-3.823992201	-3.192703202	-4.562402129	1.155161596
-4.230304444	-0.206074246	-3.984777614	-3.544664189	-2.546574335	-3.695810975	-1.918880037	-4.701691655	-4.719124044	-3.611690922	-3.980793162	-3.536199519	-4.549171024	0.868066804
-4.517532017	-0.928483746	-4.076979343	-3.677840524	-2.27530956	-3.75377129	-1.753805292	-5.194008968	-4.829184126	-3.825214639	-4.27465385	-3.189396021	-4.357977833	1.064000457
-4.603964622	-0.679099597	-4.211841563	-3.743936555	-2.986384305	-3.824604442	-1.881999091	-5.260602149	-4.940365968	-3.780356601	-4.389428014	-3.377131838	-4.590224255	1.045432989
-4.460823034	-0.720265904	-3.974968255	-3.626919785	-2.505860192	-3.570245851	-2.080213732	-5.118879419	-4.609141678	-3.576757024	-4.276472792	-3.06314372	-4.502512799	0.93810628
-4.825324058	-0.864242931	-4.575256309	-4.05098301	-2.61610957	-3.981140294	-1.964295176	-5.016772739	-5.047062126	-3.651578477	-4.325761493	-3.546466877	-4.654254703	0.843782485
-5.07641524	-0.833081735	-4.466315836	-4.342055009	-3.175436836	-3.897101967	-2.288022894	-5.457450522	-5.1009909	-3.98846693	-4.77144716	-3.33096778	-4.907737955	0.445534122
-5.061866308	-0.901328472	-4.64175607	-4.434935455	-3.239613657	-4.174871719	-2.169583311	-5.573608069	-5.084420691	-3.704905714	-4.645490426	-3.653467558	-4.691892848	0.483110206
-4.812700702	-0.807012773	-4.508624094	-4.099099814	-2.484089868	-4.017365774	-2.227626292	-5.144301922	-4.643647595	-3.894594997	-4.231612938	-3.463171171	-4.634719899	0.837195313
-4.884240825	-0.94493188	-4.79662326	-4.248904	-2.541485914	-3.488515414	-2.203308186	-5.120227413	-5.025449326	-4.11294586	-4.368202712	-3.396759646	-4.55983053	0.948639958
-4.861610838	-0.991183071	-4.657671644	-4.28246012	-2.708520691	-4.020370777	-2.087265686	-5.179065191	-4.980797981	-3.940291624	-4.432932937	-3.561489259	-4.484675114	0.728949254
-4.739944835	-0.912529946	-4.738534557	-4.249992481	-2.335193287	-3.522820538	-2.224345396	-5.185467033	-4.926511096	-4.219991249	-4.490977063	-3.291445413	-4.552192905	0.92870445
-5.036594554	-1.217496714	-4.687200206	-4.464999399	-2.378078219	-3.89732412	-2.43033827	-5.373151029	-5.345692566	-3.964066858	-4.521676648	-3.579311362	-4.434405944	0.634497049
-4.93004663	-0.572518264	-4.622412372	-4.250742716	-2.774585375	-3.69532249	-2.06230735	-5.113019266	-5.199591954	-3.612863811	-4.266847848	-3.458648048	-4.615538491	0.635426321
-4.86657917	-0.896517533	-4.701476923	-4.118682232	-2.530204843	-3.909548345	-1.912045998	-5.172752882	-5.055152432	-3.990152	-4.56999323	-3.316898079	-4.400162692	0.754473517
-4.934542835	-1.055180974	-4.702500296	-4.354976769	-2.377152597	-3.553749274	-2.236603599	-5.296186416	-5.162613709	-4.073689468	-4.332868225	-3.365891108	-4.669644791	0.937680773
-4.769079764	-0.867999917	-4.813157909	-4.299031571	-2.481086447	-3.439298165	-2.475840908	-5.304317761	-5.08983369	-4.468187152	-4.3914735	-3.442266099	-4.911283682	0.841576837
-4.795005086	-0.657942746	-4.516990188	-4.216259562	-3.053542136	-3.82050198	-1.767351982	-5.205911426	-5.225602251	-3.819388939	-4.238169426	-3.634380086	-4.344628274	0.820101439
-4.40498547	0.224239698	-4.22114758	-3.937244655	-3.069153536	-3.448078157	-1.54580448	-4.86649387	-4.933098278	-3.541609694	-3.921565649	-3.190338044	-4.366504591	0.710610827
-4.82271348	-0.992682316	-4.695050246	-4.348498702	-2.147121956	-3.949914229	-1.944154173	-5.131866624	-5.045325868	-4.200796557	-4.477289204	-3.494638724	-4.492006958	0.926144925
-4.329278519	0.138654626	-4.294748788	-3.961273554	-2.907693688	-3.688878513	-1.679291027	-4.691795032	-5.024140501	-3.701333683	-3.806118703	-3.49904082	-4.680816656	0.564493362
-4.827697605	-1.063009728	-4.635825933	-4.428905114	-2.812600455	-4.197071506	-2.147971503	-5.6477017	-4.921858401	-4.077501372	-4.691239498	-3.689483208	-4.879602697	0.565821485
-4.755873349	-0.362457588	-4.383967333	-4.155765961	-3.169210397	-3.85758021	-2.217797557	-5.230568003	-5.1163154	-3.809316394	-4.267902827	-3.510635444	-4.440158411	0.436130892
-4.71847616	-0.452978326	-3.818860508	-3.835736665	-2.614544532	-3.63199214	-2.637520189	-5.059386245	-4.913041698	-3.863166896	-4.382221024	-3.275016466	-4.934083269	0.88773284
-4.72577405	-0.843643568	-4.275600699	-3.902587574	-2.762872491	-4.139396359	-1.864759106	-4.97787458	-4.925790442	-3.788679536	-4.436980765	-3.371317263	-4.59734911	0.82218721
-4.796828008	-0.800970172	-4.354889574	-4.012424069	-3.130239669	-4.161011011	-1.86640445	-5.165960679	-4.659751872	-3.767341471	-4.574141357	-3.414852129	-4.22322504	0.74188352
-5.680897898	-0.887488076	-5.200774282	-4.896512392	-3.410117618	-3.59077795	-2.925442533	-6.00055434	-4.801642346	-4.319001114	-5.120729586	-3.354574279	-4.56170624	0.041092952
-4.780719204	-1.363801296	-4.5060056	-4.141692537	-2.020962706	-3.873617226	-2.403874978	-5.249237011	-4.700329553	-4.175226346	-4.83709097	-3.045779391	-4.528058105	1.045925819
-4.805496783	-1.137793707	-4.588074641	-4.016911442	-2.374907818	-4.065206259	-2.17398673	-5.315027418	-4.939780452	-4.389892529	-4.713938922	-2.911317512	-4.594190428	1.483235625
-5.201423208	-1.329591953	-4.938111436	-4.777362141	-2.60501612	-4.27884837	-2.577210837	-5.517165701	-5.335619856	-4.674528844	-4.843209106	-3.513732111	-4.798001412	0.993021064
-4.521189749	-0.895595911	-4.296890173	-3.970778541	-2.152882438	-3.546647661	-2.396417625	-4.783324583	-5.019178583	-4.654836722	-4.470948944	-3.219957008	-4.690413287	1.571481351
-5.767200885	-1.194310585	-5.745863642	-5.928642087	-1.111162045	-4.195670342	-2.335104851	-5.405887581	-6.940644447	-5.535424902	-5.189754399	-3.784685051	-5.213462965	1.342224711
-4.807624239	-1.46561487	-4.751386628	-4.395292974	-2.013879698	-3.969265348	-2.276886467	-4.991336815	-5.425363095	-4.557401029	-4.448013448	-3.524307667	-4.434003125	1.297463676
-4.719374976	-1.32290826	-4.382295358	-4.310818466	-2.503966248	-4.126756204	-2.315426118	-5.062124759	-5.326617143	-4.337639972	-4.371380328	-3.646756874	-4.304172147	0.928242588
-4.431911319	-1.251921971	-4.799533141	-4.316037213	-1.590912733	-3.211589525	-2.404023496	-4.622054519	-5.685351062	-4.613050439	-4.403382036	-3.341552945	-4.807959514	1.177108487
-4.740852722	-0.977744322	-4.468015977	-4.428239905	-2.545587396	-3.448286436	-2.36996681	-4.871993659	-5.215031012	-4.51852477	-4.407126517	-3.520947366	-4.740945294	1.32730893
-5.184253746	-1.809713058	-4.616292317	-4.627713223	-1.243216969	-4.400496812	-1.727458078	-4.970816697	-5.03082038	-4.507242237	-4.588916495	-3.556458457	-3.866417562	1.348497814
-5.270027755	-1.988409041	-4.851255527	-4.760555524	-1.363416998	-1.765224189	-1.968991718	-5.472554219	-5.583097593	-4.778902995	-5.018360332	-3.903602561	-4.125019623	0.890829881
-4.007885298	0.496899427	-4.142245553	-3.998452029	-2.549617194	-2.936302413	-1.769589183	-4.230010214	-4.903844601	-3.278845764	-3.451855434	-3.698073024	-5.11746579	1.134407796
-5.263325139	-1.657955567	-4.905734906	-4.599127404	-1.552898265	-4.318975223	-1.958514531	-5.658476009	-5.450291984	-5.009491885	-4.993799236	-3.759373974	-4.690914761	1.360271725
-5.082992317	-1.550115067	-4.786620165	-4.494081066	-2.020454069	-4.374726216	-2.1551985	-5.284111824	-5.061034781	-4.512198898	-4.774092691	-3.781771966	-4.613088956	1.121350411
-4.635820712	-0.928208477	-4.401311307	-4.125068526	-2.804492348	-4.022962712	-2.506753913	-4.973492096	-5.265403466	-4.398518524	-4.304226138	-3.685925031	-4.332883584	0.513976764

-4.997854741	-1.659350777	-4.646204906	-4.28385128	-2.260387161	-4.066225907	-1.910347135	-5.385910314	-5.217614538	-4.640065251	-4.464454917	-3.527244251	-4.393291044	0.999394952
-4.400629277	-1.232564623	-5.039237115	-4.313960575	-1.152736524	-3.622152753	-1.80535668	-5.3682895	-5.105924194	-4.464447228	-4.771213239	-3.461452975	-4.486830238	1.542140229
-4.758909225	-1.062588093	-4.585066571	-4.125064029	-1.223616725	-3.380231854	-2.338564484	-4.890534526	-5.044338996	-4.158402214	-4.350720953	-3.237494107	-4.270803784	1.422599942
-4.315979942	0.189913557	-4.299848554	-3.986238193	-2.701934341	-2.932515825	-1.818216756	-4.396929406	-4.952089446	-3.322322287	-3.539469527	-3.648458967	-4.252868768	0.736364293
-4.559754905	-0.517329024	-4.55859055	-4.290826153	-2.792668131	-3.575508548	-1.845515217	-4.996660346	-4.760458513	-4.344467249	-4.09973838	-3.672621652	-4.824874324	1.00684283
-4.998316281	-1.198901893	-4.484967111	-4.892389263	-1.039110486	-3.47399705	-1.926553462	-5.565435625	-4.688438405	-4.138002055	-4.466761824	-3.325935869	-4.06010135	1.23044926
-3.495127986	-0.82394869	-4.360274948	-4.533291545	-0.969464652	-3.157404797	-2.076052676	-5.407031592	-5.991658127	-4.077052519	-4.073438323	-3.288493993	-4.480292331	1.369612087
-4.644789073	-0.308810505	-4.492671942	-4.29908528	-2.37047681	-3.418132291	-2.304291248	-5.133425237	-5.268863788	-4.142921346	-4.375834043	-3.432968086	-4.454622509	1.254772044
-4.875204798	-1.33940214	-4.799007515	-4.399686862	-1.25093005	-4.069557057	-1.883558675	-5.095735796	-5.668382338	-4.6633331	-4.556744648	-3.33722128	-4.110992667	1.446336555
-4.969265724	-1.440817332	-4.654602536	-4.313530474	-1.72430538	-4.056491526	-1.747987522	-5.351054091	-5.150623126	-4.481298712	-4.753039535	-3.336879687	-3.987920051	1.021648188
-5.056978497	-1.456876591	-4.810715907	-4.599028414	-1.242971665	-4.315888923	-1.944409289	-5.632165035	-5.129260039	-4.639705825	-4.985887455	-3.96927436	-4.127693015	1.060389245
-4.999914891	-1.654688732	-4.600432798	-4.472022087	-1.753571865	-4.14191722	-1.822346694	-5.422214464	-5.23218677	-4.545230205	-4.674234663	-3.734894662	-4.617235981	0.918019723
-5.023901098	-0.926934745	-4.820613965	-4.773268822	-2.816476866	-3.663371147	-2.477482317	-5.244669079	-5.54295544	-4.845576652	-4.639826761	-3.62023223	-5.229776459	1.001532207
-5.360770963	-1.595490521	-5.148541878	-4.891044022	-1.15913391	-4.718139938	-1.994829361	-5.014972438	-5.184457437	-4.8357587	-4.957326453	-3.579133516	-4.499559742	0.937157981
-5.365108231	-1.43431599	-6.388810719	-5.584701713	-1.060771901	-4.050280166	-2.393203386	-5.440777319	-6.792368402	-4.795435432	-4.937444355	-3.795240351	-4.372833941	0.961671996
-4.884050299	-1.054428023	-4.989949508	-4.761344578	-1.170113213	-3.593336334	-2.32325017	-5.054439496	-5.12252818	-4.420457947	-4.817959208	-3.475102563	-4.549821626	1.208815983
-4.685216221	-1.529696731	-4.300503718	-4.510676069	-1.373646241	-3.985977988	-2.271687026	-5.252935562	-5.659954533	-4.65608031	-4.267852114	-3.975273014	-4.507534017	1.083893589
-4.248827715	-0.499238108	-3.789395732	-3.733755199	-2.81877943	-3.343258248	-2.519947768	-4.795697869	-5.153166875	-3.888636913	-4.005479416	-3.356082449	-5.03467807	1.043962748
-4.780866609	-1.114984199	-4.413325569	-4.364711572	-2.502759452	-3.981724847	-2.011189294	-4.850129407	-5.425178149	-4.397103496	-4.494002237	-3.252175509	-4.456492363	1.056829955
-5.390980418	-2.053400112	-5.169742041	-4.654822006	-1.665834328	-4.601876178	-1.668084284	-5.586851238	-5.290048936	-4.677412632	-4.978904231	-3.576634245	-4.319007661	0.834348914
-4.72000815	-0.962240351	-4.306238956	-3.959174163	-2.648806592	-3.360061022	-1.723028819	-5.222442892	-5.231532786	-4.230887003	-4.519307553	-3.255239298	-4.409009584	1.192866395
-5.102275608	-1.487488863	-4.811114493	-4.294561993	-3.29018426	-4.484828224	-2.351172233	-5.885354151	-4.507899181	-3.503750297	-5.429892777	-3.370392486	-5.082751703	0.191619615
-5.534409059	-1.393941361	-4.95276447	-4.829055837	-3.639483134	-4.54778754	-2.636328853	-5.859246538	-5.316662863	-4.045445564	-5.186722581	-3.677796914	-4.321753704	0.303815426
-5.988496663	-1.496350766	-5.261314657	-4.80741689	-2.375933486	-4.355274884	-2.29266887	-7.219727634	-5.283264092	-4.463593562	-6.301537717	-3.310452891	-5.537643878	0.763392672
-5.146514574	-1.292795433	-4.807082409	-4.516782659	-3.066981059	-4.073055003	-2.417075878	-5.905211556	-5.017951664	-4.062449137	-5.153218529	-3.524234998	-4.887042745	0.740951334
-5.319936684	-1.271595979	-5.162396288	-4.753599258	-1.878790425	-3.989155517	-2.268651383	-5.786381963	-5.461626334	-3.782788132	-4.757088267	-3.837007045	-4.841974997	0.676362825
-5.015353565	-1.226867797	-4.775275196	-4.162601084	-2.774889337	-3.628515173	-2.32450552	-5.662728202	-4.289401189	-3.130096208	-5.339251973	-3.129451563	-4.945438307	0.329232391
-4.715842496	-1.208396189	-4.678547887	-4.377448333	-3.300218024	-3.371147165	-2.226914263	-5.51260788	-4.292769449	-3.43061767	-5.02118515	-3.124617927	-5.082302818	0.571623485
-5.197449499	-1.294860317	-4.681232568	-4.145565009	-2.786236957	-4.170424652	-2.149151882	-5.665660314	-4.517982931	-3.473578179	-5.179475821	-3.162166445	-5.194621881	0.064245996
-5.057822547	-1.281649838	-4.914870181	-4.262002191	-2.381520201	-4.388471806	-2.06362753	-5.516723636	-4.998468892	-3.830236728	-4.877754763	-3.641550328	-4.442207549	0.605885983
-5.363585764	-1.358974246	-4.964724066	-4.496193516	-2.865731693	-4.179405388	-2.095662799	-5.461347209	-4.301255769	-3.056346422	-5.435143644	-3.1670862	-4.409921785	0.267619257
-5.12360739	-0.983771035	-4.812713728	-4.324572151	-2.291362237	-3.395198473	-2.655703255	-5.169731095	-4.910938569	-3.626384655	-4.819429509	-3.242293596	-4.547835705	0.69920517
-4.892677707	-1.320556166	-4.696020297	-4.279406077	-2.534504126	-4.120807426	-2.201843942	-5.559507374	-3.984400562	-3.176321985	-5.53913207	-3.233543696	-5.010128773	0.372303779
-5.07468415	-0.637786328	-4.761560207	-4.243186978	-3.089117468	-4.044271947	-2.098284585	-5.559228586	-3.977875567	-2.997426559	-5.503232651	-2.955887595	-4.775862221	0.359100018
-5.071859294	-1.621262715	-4.558172647	-4.425074216	-2.580295362	-4.346256237	-2.403126398	-5.467534245	-5.266326486	-4.471616194	-4.766956175	-3.689496278	-4.583619368	0.814913927
-4.745487956	-0.975794079	-4.494709422	-4.108361997	-1.585853194	-3.922424258	-2.180763729	-5.030275043	-5.083175092	-4.710535858	-4.265005354	-3.416810087	-4.444674253	1.443227791
-4.503597508	-1.508502785	-4.693365326	-4.495089008	-0.963562408	-4.216882362	-1.73014123	-5.178819207	-5.303211341	-4.470275267	-4.617275927	-3.203934162	-3.841589384	1.197689232
-4.566580847	-1.392237041	-4.617258525	-4.678962963	-1.224139514	-4.313854939	-1.631037122	-5.02190762	-5.860816304	-4.540680841	-4.447433047	-3.972555665	-4.325410069	1.143453562
-4.951326112	-1.65105653	-4.734820128	-4.520890129	-1.867335883	-3.929373067	-2.425973627	-5.046750449	-5.534548448	-5.092946229	-4.73419681	-3.752270004	-4.397242706	1.135928916
-4.495126542	-0.408626296	-4.73788012	-4.279713259	-1.699065186	-3.836116413	-1.705276523	-4.937066209	-5.670568109	-4.507960446	-4.559815104	-3.361538058	-4.795072709	1.529289657
-4.917166094	-1.669132046	-4.670912767	-4.510383292	-1.503868015	-4.294664394	-1.772426673	-5.216418883	-5.298765229	-4.89506383	-4.6916155	-3.792468183	-4.418252069	1.10716417
-5.095181578	-1.785131912	-4.79647735	-4.445333426	-2.020425524	-4.509673239	-1.746094949	-5.442728298	-5.435775441	-4.658088501	-4.737486454	-3.59524399	-4.232296015	0.880335254
-5.379558049	-1.59863586	-5.075906157	-4.866659966	-1.544230628	-4.423023555	-1.440253993	-5.476887411	-5.471845637	-4.717334601	-5.272501036	-3.426437334	-4.041381774	1.00226903
-5.289017274	-0.937037856	-5.313314816	-4.53218437	-1.777321727	-3.745663828	-2.653388744	-5.213932989	-5.015482361	-4.874357104	-4.837522835	-3.426397315	-4.511481528	0.94807616
-5.612772369	-1.379618614	-5.274536393	-4.837980685	-2.718684754	-3.442105977	-2.362292218	-5.715669051	-5.698144102	-4.897644749	-4.960389405	-3.294904454	-4.637197541	0.989399123
-5.310560326	-1.819474795	-4.906437114	-4.816804933	-1.197655608	-4.27133651	-1.984162876	-5.254937341	-5.433332933	-4.765753737	-4.722923674	-3.705756921	-4.554194962	1.081162349
-4.716840517	-0.847021555	-5.149640812	-4.788090231	-1.067562777	-3.462815544	-2.211021579	-4.814405992	-5.457782719	-4.678394959	-4.563833512	-3.400684713	-4.555555289	1.415384158
-5.188226626	-1.717263238	-4.489062581	-4.317426852	-1.759970914	-4.326928164	-1.570562118	-5.287163708	-5.724389839	-4.559409147	-4.53275267	-3.637549504	-3.619356352	0.89512893
-5.032811304	-1.525304507	-4.967329309	-4.456849068	-1.278897304	-4.063199499	-1.627424853	-5.525241341	-5.064111997	-4.39219437	-5.246012839	-3.555230268	-4.401531516	1.179921104

-4.851493702	-1.160857188	-4.710427639	-4.249662656	-2.48357425	-3.646609825	-1.962266518	-5.327329834	-4.008576814	-3.089393562	-5.222217606	-3.132076733	-4.749109612	0.615483665
-4.6258344	-0.970378229	-4.552963021	-4.135445667	-2.707798618	-3.951994726	-1.912289994	-5.2604365	-4.027120783	-2.752338457	-5.132814279	-3.341924863	-4.733199812	0.344957152
-4.947791671	-1.354787108	-4.722239977	-4.158828342	-2.623207866	-4.252883461	-1.890188646	-5.299782899	-4.00098571	-3.064950055	-5.362241474	-3.326748235	-4.951288517	0.572122422
-4.933365734	-1.200984414	-4.581984094	-4.166398947	-2.891629274	-4.132464308	-2.168398856	-5.652630858	-4.247333883	-3.300733513	-5.23924499	-3.235072467	-5.069179642	0.454212197
-5.247328715	-1.490827556	-4.805153536	-4.501780867	-3.132966331	-4.518428138	-2.085218975	-5.880614752	-4.771103446	-3.612068612	-5.252213788	-3.52730296	-4.97016831	0.361074993

hsa-miR-6819-3	hsa-miR-6819-5	hsa-miR-6820-5	hsa-miR-6821-5	hsa-miR-6822-5	hsa-miR-6824-5	hsa-miR-6825-3	hsa-miR-6825-5	hsa-miR-6826-3	hsa-miR-6826-5	hsa-miR-6827-5	hsa-miR-6829-5	hsa-miR-6831-5	hsa-miR-6836-3
-4.959809315	-1.190242615	-1.989786298	-0.291122535	-5.109285117	-2.121545946	-4.220087252	-1.842798356	-3.906306485	-4.094783938	-3.731362584	-3.075327741	-2.191686505	-4.012032367
-5.213659625	-1.985609204	-1.570173113	0.091523978	-4.657742818	-2.07930569	-4.139253467	-1.70857111	-4.425848341	-4.56548481	-3.865802893	-3.146615026	-2.669068711	-3.046739104
-5.042453506	-1.795126007	-1.50360515	-0.0496466	-4.967134216	-1.737381813	-3.995157338	-0.965129367	-4.233295626	-4.299879664	-4.024257008	-3.124541963	-2.475274074	-3.512149482
-5.17936134	-1.69333229	-1.63335471	0.039945204	-5.012389424	-1.662559245	-4.123412808	-0.879069375	-4.164033098	-4.248981404	-4.050889094	-3.203384174	-2.184033878	-3.833949142
-4.745153677	-1.376378077	-1.904055803	-0.059650656	-4.727370474	-1.58039571	-4.164273023	-1.071229698	-3.874491171	-3.880148441	-3.816148198	-3.178448608	-2.200309458	-3.48920312
-4.808598199	-1.714977974	-1.350482302	0.07263179	-4.476231772	-2.003320058	-4.004335968	-1.420688581	-4.182548462	-3.731664053	-3.991515001	-3.042045828	-2.369956975	-2.611073769
-4.839041145	-1.550314231	-1.656016553	-0.03716352	-4.489745722	-1.69099536	-3.835941976	-1.547322613	-3.971125625	-3.816456079	-3.902920779	-3.255806319	-2.153924641	-3.212434829
-5.192129232	-2.037161137	-1.451130455	0.165183903	-4.66177102	-1.892310687	-4.055473433	-1.373425212	-4.303063011	-4.19064061	-4.19414846	-2.845900598	-2.80371229	-2.638474245
-5.194017331	-1.908665346	-1.657535043	0.1494896	-5.085099488	-1.945791453	-4.211901217	-1.582147629	-4.344388518	-4.261152298	-4.092180784	-3.0996552	-2.601803339	-3.286446385
-5.016205107	-1.922742519	-1.473615193	-0.068276351	-4.419669495	-2.028059838	-4.082341214	-1.860288919	-4.351104157	-4.164988596	-3.92597843	-3.040751025	-2.41691537	-2.549383557
-5.138074799	-1.878577427	-1.529280741	0.049790251	-4.537949737	-1.796121011	-4.020452369	-1.599170443	-4.515337365	-4.117585966	-4.178040919	-3.076488489	-2.451615841	-3.124699465
-5.663276284	-1.881709166	-1.704083798	-0.468679686	-4.934954893	-2.141449875	-4.439098431	-1.822962195	-4.606128357	-4.846930539	-4.080828223	-3.169057599	-2.283228784	-3.228287792
-5.705014	-2.116633687	-1.580199313	-0.365142541	-5.132973052	-1.888197427	-4.301258869	-1.759082999	-4.759630268	-4.637682676	-4.409440007	-3.387219589	-2.27592342	-3.571239493
-5.122577196	-1.873534082	-1.763176045	-0.261685086	-4.984377833	-1.916164849	-4.143813842	-1.62474506	-4.586198505	-4.347050399	-4.258048282	-3.072792006	-2.512638953	-2.976853238
-5.423309851	-2.074948761	-1.793554957	-0.211209273	-4.743268444	-2.207611972	-4.240155093	-2.163293749	-4.740512576	-4.285446939	-4.059648204	-3.21657178	-3.221942239	-2.664051176
-5.335209865	-2.046391993	-1.579667202	-0.096586355	-5.142679269	-1.981731634	-4.201826143	-1.436840809	-4.567069028	-4.688793743	-4.098986627	-3.10414225	-2.85372067	-3.283802475
-5.311988365	-2.195979577	-1.567869442	-0.306535223	-4.775958978	-2.147498997	-4.180302594	-2.030984535	-4.729649772	-4.379350428	-3.981922547	-3.026402848	-3.425487643	-2.358409741
-5.36132562	-2.224378447	-1.550935288	-0.184526418	-4.972717425	-2.214562859	-4.287299684	-1.874546335	-4.803328507	-4.48774148	-4.313655989	-3.194683042	-2.706356774	-2.649626403
-5.196600759	-1.864759648	-1.647451862	-0.356866713	-4.792269578	-1.894169901	-4.399385345	-1.711697251	-4.621993961	-3.918208886	-4.23488206	-3.074779485	-2.210337243	-3.154788516
-5.258385644	-2.152212389	-1.422314465	-0.151642926	-4.846122933	-1.921393835	-4.21316332	-1.5937236	-4.667424345	-4.20696501	-4.285863125	-3.064175351	-2.664166073	-2.854305852
-5.368703995	-2.011468409	-1.598864945	-0.471357774	-4.674236829	-2.278615251	-4.298700838	-2.180094287	-4.834800009	-4.372138137	-4.083395439	-3.287103515	-3.072709387	-2.66849321
-5.318321674	-2.068087629	-1.636140895	-0.5212817	-4.630709785	-2.477800804	-4.188085195	-2.514904587	-4.691047626	-4.313801656	-4.048667779	-3.370750201	-3.512135291	-2.424933558
-5.143230746	-1.945847541	-1.453238144	0.05431381	-4.795979697	-1.66862382	-4.221248526	-1.305568495	-4.526293699	-4.086503681	-4.286325868	-3.297805338	-2.329905657	-3.40602974
-4.986397152	-1.224429785	-1.654214712	-0.210836367	-4.758284615	-1.414724475	-4.172079536	-1.147045011	-4.069374181	-2.758243817	-3.803034949	-3.042386265	-1.817372988	-3.679164289
-5.334911889	-2.070306733	-1.612319841	-0.121086393	-4.851315487	-1.901978244	-4.261680986	-1.293317222	-4.653147727	-4.117459528	-4.270148264	-3.084931166	-3.19489357	-2.513059854
-4.886991221	-1.04731309	-1.532509957	-0.179151713	-5.067850922	-1.573174743	-4.075124405	-1.183876106	-4.008754997	-2.701333779	-3.838576357	-3.004449945	-2.300249397	-3.631851543
-5.953145189	-2.343699485	-1.601091174	-0.355091812	-4.949909852	-1.995970099	-4.379492307	-1.753558651	-4.847873751	-4.375506454	-4.475840914	-3.283224458	-2.583665635	-2.969261439
-5.306621754	-1.676674394	-1.918450884	-0.626337137	-4.825565599	-1.792336403	-4.395613286	-1.942427096	-4.337987274	-3.210388058	-3.90619833	-3.158023327	-2.228938039	-3.493586304
-5.140426188	-1.686916889	-1.907831165	-0.316671047	-5.096965446	-2.515670428	-4.492568939	-1.977055959	-4.202067494	-4.200369833	-3.829638394	-3.289025831	-3.090593265	-2.877629385
-5.207002524	-1.890324255	-1.358458343	-0.05683864	-5.183868594	-1.745634852	-4.258071556	-1.507699136	-4.434918279	-4.408915434	-4.190241005	-3.056162997	-2.48824239	-3.093212761
-5.31058226	-1.977552061	-1.484129543	-0.023530052	-5.008125887	-1.822084488	-4.228424774	-1.678025074	-4.450456953	-4.330736735	-4.106778709	-3.009567363	-2.260911355	-3.52381526
-6.123524515	-1.85910474	-2.870240636	-0.798003075	-5.134088208	-2.748514991	-5.563360866	-3.053085693	-5.031040125	-2.707003321	-3.986190697	-3.5070756	-3.170318042	-4.000056938
-5.781224977	-2.374996574	-1.794339743	-0.189061124	-4.823516121	-2.6695368	-4.40489518	-2.062154888	-4.815894036	-4.773289365	-4.422927209	-3.036091832	-4.138597529	-2.119027115
-5.583461474	-2.366727677	-0.576149728	0.241656066	-4.407512871	-2.426356442	-4.051789998	-2.358585934	-4.806829227	-4.768832389	-4.548686559	-2.883788361	-3.845202975	-2.1357097
-5.364859035	-2.549124285	-1.540058212	-0.478998455	-5.270873773	-2.475839977	-4.39206445	-2.195345378	-5.084072075	-4.996292867	-4.944363978	-3.510962909	-3.859604327	-3.090520329
-4.92052395	-2.044870484	-1.271411491	0.015320964	-4.922305117	-2.66521486	-4.028853445	-1.73988938	-4.518272997	-4.736623147	-4.167239723	-3.287826519	-3.963091256	-1.909292428
-5.394923678	-2.810072332	-1.301489398	-0.343396614	-4.600542514	-2.737257667	-4.50892603	-2.659965125	-6.040559946	-4.714036865	-5.213441909	-3.385930173	-5.261748234	-1.262463109
-5.047844582	-2.635102535	-1.4639253	-0.115192472	-4.508420078	-2.563348656	-4.070181301	-2.015216213	-4.93081298	-4.843670815	-4.802692767	-3.297749432	-4.013475328	-2.070082326
-5.184902249	-2.235921513	-1.379104317	-0.249231528	-5.091560626	-2.452273623	-4.112701179	-1.469546133	-4.706325822	-5.043555168	-4.652404476	-3.373201793	-3.453086684	-3.092526061
-5.072372163	-2.409389267	-1.813684516	-0.402928175	-4.696358068	-2.785717184	-4.379740707	-2.290234196	-5.064383138	-4.113803659	-4.044814716	-3.510016538	-4.291028181	-1.584900537
-5.11302209	-1.985212332	-1.974225616	-0.473479296	-4.695818181	-2.634913765	-4.194589252	-2.573200919	-4.750830338	-3.455271514	-4.270733579	-3.374460109	-3.604057164	-2.530888344
-5.524726374	-2.584130526	-0.314802026	0.281078789	-4.543110207	-2.108754226	-4.092618129	-1.698908855	-3.870516989	-4.718904152	-4.70446222	-3.060778332	-4.039419553	-2.507245021
-5.724923575	-2.894666289	-0.616431296	-0.134596133	-4.758102518	-2.481638038	-4.370035878	-1.852690237	-5.377193728	-4.898918418	-4.705911009	-3.360738869	-4.558007755	-2.689032661
-4.441128773	-0.355769075	-2.513193217	-0.6373484	-4.974420485	-1.579015308	-3.994540393	-1.520783418	-3.82487837	-3.751272203	-3.76098992	-3.156014987	-2.011644136	-3.422758355
-5.821961806	-2.631477089	-1.085260439	-0.061235573	-4.865890569	-2.159792457	-4.214967111	-1.643046963	-4.995919139	-4.993614566	-5.077569656	-3.471885462	-4.17425223	-2.372992339
-5.388497341	-2.643918787	-1.496382406	-0.233625597	-5.241436399	-2.258584225	-4.193960992	-1.852437093	-5.181536952	-4.861680791	-4.906105529	-3.523338872	-4.083789286	-2.690052328
-5.031315072	-1.899209913	-2.198869094	-0.198754305	-5.275849927	-2.338958736	-3.972334725	-1.144622731	-4.435749268	-4.635457558	-4.476739393	-3.600492158	-2.949873669	-3.984134896

-5.48249114	-2.553821364	-1.436483789	0.028869853	-4.823686947	-2.118371078	-4.392746616	-1.670853248	-5.082374487	-4.488603079	-4.790855379	-3.315331658	-3.182349581	-2.784104275
-5.190278148	-2.358097842	-1.213214792	-0.126249772	-3.993227911	-2.142680814	-4.302285203	-2.004318585	-5.06798518	-4.496531831	-4.622800243	-3.296151761	-4.425768835	-1.632608715
-4.654721759	-2.018955395	-1.714264108	-0.224647571	-4.075411308	-2.579112396	-4.106511908	-1.592143637	-4.645661582	-4.499682344	-4.547636468	-3.207596565	-3.373300127	-1.576233908
-4.516091452	-0.615740104	-2.260556723	-0.317621638	-4.845995119	-1.824052444	-4.049259198	-1.053922978	-3.948644889	-4.137363771	-3.954879981	-3.003858011	-1.701863901	-3.452646506
-5.111011436	-1.542519854	-1.726480562	-0.555571708	-5.251122789	-1.923428056	-4.474175848	-1.320496673	-4.426583018	-3.93986665	-4.251101499	-3.485010676	-3.210451528	-3.59915183
-5.122195578	-2.421937613	-0.907308858	-0.083614894	-3.281275566	-2.287866087	-4.362511013	-2.181213324	-5.191460126	-3.641196397	-4.556755797	-2.799601907	-3.553094522	-1.312608105
-4.543161587	-1.816693015	-1.649285589	-0.327373407	-2.965340344	-2.634909927	-4.164256543	-2.447859399	-4.73472675	-3.694836691	-3.905163693	-2.745177337	-4.198347962	-0.72312916
-4.997218583	-1.421808779	-2.169224936	-0.414079133	-5.37317006	-2.226451794	-4.520513025	-2.201508384	-4.521647291	-2.100920645	-4.276704932	-3.504884415	-3.446192105	-2.655685047
-5.312820403	-2.491578742	-1.071341081	0.233367055	-5.13027579	-2.041393557	-3.960705301	-1.742442137	-4.789724371	-4.271666727	-4.587857138	-2.75858724	-4.200254216	-1.66870747
-5.457257904	-2.474932706	-0.809702345	0.266069279	-4.782393972	-1.989921568	-4.228969647	-1.239074208	-5.012606135	-4.326247871	-4.731424092	-2.880612558	-3.912906047	-2.807782402
-5.506226651	-3.071381049	-0.789037248	-0.257173076	-4.019782551	-2.226459983	-4.000143626	-2.022670871	-5.58685622	-3.906411049	-4.518371519	-2.91747933	-4.711832589	-2.404403975
-5.58090618	-2.752594848	-1.223290093	-0.121595342	-4.586405746	-1.991661435	-4.242892395	-1.239232462	-5.034013387	-4.57259578	-4.79739478	-3.125849825	-4.450366428	-3.020715571
-5.574352654	-2.431507129	-1.884074679	-0.620471355	-5.34013569	-2.465958527	-4.721506013	-2.061755575	-5.079963175	-5.037171509	-4.389181505	-3.562672122	-3.613128856	-3.110425806
-5.946047349	-2.955728451	-1.184683809	0.017555067	-4.824743319	-2.166378028	-4.430287213	-1.69685574	-5.040862779	-4.28277522	-5.004020555	-2.833062306	-4.287032066	-2.370754827
-5.140660351	-2.668606937	-1.902401495	-0.18141662	-4.408669142	-2.607751075	-4.587092982	-2.499269342	-6.10867269	-4.436422707	-5.079791579	-3.023425988	-4.541489193	-1.821333546
-5.351174994	-2.133438537	-1.836701796	-0.181729323	-4.469929678	-2.445617039	-4.319113861	-2.261461651	-4.950605025	-3.794657648	-4.150650424	-3.02096349	-4.069037161	-1.6972902
-5.069588816	-2.651930688	-1.561931006	-0.095034244	-4.721079527	-2.661778406	-4.342323426	-1.937301101	-5.163860748	-5.011125374	-4.857831713	-3.670859609	-4.104231923	-2.062727899
-4.786880096	-1.780276237	-1.968256668	-0.593492406	-5.162630956	-2.540135444	-4.132073772	-1.886386511	-4.010404726	-4.504099441	-3.928387644	-3.353378082	-3.243933989	-3.132898421
-5.405659229	-1.868793167	-1.630364697	0.062261229	-5.504051575	-1.969059334	-4.133504439	-0.981468819	-4.410156391	-4.948217037	-4.442452667	-3.100128396	-3.238631366	-3.855729818
-5.814826514	-2.779851639	-0.662171135	-0.003360353	-5.057677339	-1.929273662	-4.28231572	-1.620810321	-5.454177892	-4.712235875	-4.877385687	-3.143229443	-4.22077691	-2.495998763
-5.248591765	-1.904262761	-1.225017769	-0.31428432	-4.77099617	-1.899938288	-4.08391971	-1.348692479	-4.584934479	-4.447746797	-4.210628865	-3.235625363	-3.447393677	-2.587817773
-5.948764255	-2.2510384	-1.448046367	-0.536080244	-3.494675296	-2.388878159	-4.873693414	-2.526009648	-4.921012278	-4.307087094	-4.121779847	-2.816514713	-3.688868754	-3.143085802
-6.133133234	-2.429095822	-1.61320107	-0.445348512	-5.606936236	-2.552817211	-4.684307305	-2.449513201	-5.1343662	-4.970063842	-4.828198318	-3.31772701	-2.930885468	-4.193638818
-8.310961311	-2.534497287	-1.5141851	-0.324053107	-4.893189178	-2.705618765	-4.950605637	-2.670383941	-5.930030408	-4.917431127	-4.670200127	-3.133716629	-4.537603161	-2.250636149
-6.00699407	-2.394440216	-1.735798355	-0.411118382	-5.237999221	-2.370545786	-4.710586848	-2.563160668	-5.00554123	-4.753604188	-4.484687446	-3.200864379	-3.139154338	-2.697486568
-5.437456049	-2.194671855	-1.505924731	-0.461301552	-3.610214915	-2.484276023	-4.54729445	-2.20230715	-5.245380324	-4.183037558	-4.552235095	-2.942617452	-3.680527431	-1.685327466
-5.556905701	-2.008672536	-1.37132172	-0.945742577	-3.171755405	-2.642965497	-5.055363726	-2.886574876	-4.760441104	-3.720074628	-3.647476599	-2.517125052	-4.064341163	-2.721116986
-5.7750829	-2.131112552	-1.293939804	-0.814647169	-3.741832995	-2.618158827	-4.71530276	-2.965855157	-4.736520799	-4.206363223	-3.631052482	-2.810578564	-3.97882408	-2.842473582
-5.672528318	-2.183381619	-1.351094823	-0.551940591	-3.631783749	-2.421852067	-4.807418728	-2.282686118	-4.821769597	-4.474797864	-4.133745818	-2.725739237	-3.529928409	-2.967916125
-5.562856792	-2.124251494	-1.450970357	-0.301030959	-3.752519041	-2.247812375	-4.521310589	-1.799862285	-5.099147599	-4.034034419	-4.501363632	-2.714483777	-3.133444266	-2.514196467
-5.715645912	-1.980544088	-1.223711697	-0.469697292	-2.891220356	-2.705939842	-5.037670215	-2.72906335	-5.140819845	-4.264334593	-4.115285497	-2.339878801	-4.030539491	-2.737035216
-5.587319177	-1.795273732	-1.755694501	-0.516781315	-3.275981884	-2.611211678	-4.873629655	-2.946543315	-4.659312143	-3.691414064	-3.725721824	-2.426555956	-3.495899892	-1.98717908
-5.95032242	-2.275298115	-1.284422798	-0.613615026	-2.817199623	-2.397874984	-4.723171766	-2.525534793	-5.062846989	-3.848432176	-3.955985015	-2.666025476	-3.754370467	-3.048919076
-6.158610207	-1.790297808	-1.357655386	-0.573109807	-2.944551054	-2.316312943	-4.895343873	-2.701625823	-4.936258106	-3.914409746	-3.7430526	-2.448718156	-3.626523095	-3.038765516
-5.549335151	-2.884484181	-1.090435784	-0.230397136	-5.074740712	-2.545168649	-4.242760169	-1.899829146	-4.917513466	-4.567025068	-4.745745682	-2.686887625	-4.175888526	-2.99289818
-5.158309121	-2.105782538	-1.130669461	-0.070172987	-4.775746011	-2.427298056	-3.758400935	-1.664976511	-4.667025565	-3.852425204	-4.486445819	-2.819303085	-3.439365136	-2.159634439
-5.089164961	-2.487767738	-0.829581368	0.079844343	-3.948525962	-2.30368389	-3.952788567	-1.639567956	-4.817619816	-3.761882282	-4.455278316	-2.970844599	-3.479420853	-1.895679563
-5.612417072	-2.516771587	-1.60882361	-0.257649853	-4.185411527	-2.207960208	-4.151435205	-1.459733198	-5.026985461	-4.196099022	-5.033540696	-3.409946291	-4.029682289	-1.762241105
-5.42664608	-2.60961894	-1.59299557	-0.050418078	-4.664332861	-2.681455243	-4.188730103	-2.647393028	-5.15461509	-4.00894881	-4.978604396	-3.399486383	-4.312406017	-2.082488796
-5.16727516	-1.899245483	-1.993923762	-0.361250405	-4.77010684	-2.497916266	-4.316194961	-2.863823399	-4.676502539	-1.571657597	-4.299563762	-3.447284031	-3.783904846	-1.420579639
-5.451604496	-2.876076767	-1.141967593	-0.113246926	-4.881070082	-1.948454707	-4.047485286	-1.554309589	-5.150938957	-4.378933419	-4.79645768	-2.973729167	-4.220931889	-2.268601679
-5.719221424	-2.76705045	-1.053695087	0.026699379	-4.949479675	-1.935394823	-4.144179479	-1.590395748	-5.078153867	-4.426487076	-4.949914383	-3.107130492	-4.061767982	-3.525854099
-6.122230359	-2.758517319	-0.833534661	0.135634528	-5.093059706	-1.67028501	-4.440864159	-0.973267608	-5.365150472	-4.206108921	-4.934945295	-2.689657808	-4.143901514	-2.895968831
-5.747412606	-2.136200627	-1.717011727	-0.332683465	-5.392789629	-2.839286345	-4.919069568	-2.204269549	-5.108105013	-3.17072602	-4.137830849	-3.356670143	-4.04641661	-2.837074696
-6.000495094	-2.469509606	-1.785685283	-0.587080871	-4.666092606	-2.543464071	-4.846714973	-2.48530197	-5.419020002	-4.79824676	-4.413270364	-3.258557458	-4.093708831	-3.020012413
-6.163254103	-2.950160655	-1.287571326	-0.023633506	-4.858517816	-2.245867561	-4.613902416	-1.924099184	-5.403334972	-4.896082486	-5.162660698	-3.284703183	-4.392986982	-1.900324477
-6.05953415	-2.108095326	-1.816921656	-0.088834284	-4.650784207	-2.474151885	-4.38790379	-2.610963194	-5.367536866	-3.82359353	-4.276331877	-2.918098081	-4.525842049	-1.2135452
-5.292475931	-2.943374712	-0.752739472	0.020248379	-4.529814255	-1.991923905	-4.419064588	-1.256261163	-5.021641355	-5.039186427	-5.090682421	-3.087347178	-3.806239912	-3.049949562
-5.761789262	-2.480896773	-1.222256636	-0.004447138	-4.016059916	-2.01139938	-4.176069428	-1.828805584	-4.919336952	-4.151857699	-4.548699762	-3.149785663	-4.432097363	-1.912808961

-5.83135068	-1.934449939	-1.174983063	-0.509354614	-2.677346265	-2.272767782	-4.651185973	-2.31157837	-4.628101262	-3.842431021	-3.713473531	-2.40041647	-3.856891682	-2.852045742
-5.596008314	-1.93369173	-1.213589761	-0.646071145	-2.822014209	-2.241126515	-4.447169901	-2.320832079	-5.085085352	-3.797553676	-4.053024778	-2.389454656	-3.841771372	-2.572105564
-5.668760498	-2.087696371	-0.918398271	-0.247426889	-2.943992747	-2.185671628	-4.917354772	-2.364653897	-4.76525921	-3.977798246	-4.072426817	-2.47145359	-3.732452476	-2.574418628
-5.780714712	-2.109879204	-1.201464812	-0.473120556	-3.247309514	-2.382189854	-4.761105927	-2.543657489	-4.977147225	-4.311014584	-4.101770958	-2.655769352	-3.78263293	-2.61708572
-6.040077428	-2.273226862	-1.41032895	-0.571360228	-3.715955798	-2.266488868	-4.812711836	-2.150243348	-4.826600908	-4.499185508	-4.158837394	-2.860086196	-3.507408891	-3.316936758

hsa-miR-6836-5	hsa-miR-6837-5	hsa-miR-6840-3	hsa-miR-6842-5	hsa-miR-6845-3	hsa-miR-6845-5	hsa-miR-6846-5	hsa-miR-6848-5	hsa-miR-6849-5	hsa-miR-6850-5	hsa-miR-6851-5	hsa-miR-6857-5	hsa-miR-6858-3	hsa-miR-6858-5
-4.641056185	-3.641237138	-1.710468573	-2.936493398	-3.620686771	1.679911976	-3.638429757	-1.418356176	-4.691654954	1.291471644	-2.570060405	-2.892865545	-4.396518175	-0.934099957
-4.143881311	-3.882954256	-1.177696591	-3.021957336	-3.690192995	1.474597325	-3.480765895	-1.745236722	-4.70498119	1.869918858	-2.627416978	-3.623008255	-4.37718079	-0.863455651
-4.078262135	-4.064343305	-1.350180129	-2.624862807	-3.619731308	1.441899493	-3.313206138	-1.816271771	-4.560990233	1.380699968	-2.767543151	-3.533279907	-4.311615095	-0.720242655
-4.059610993	-3.895702261	-1.314323872	-2.40082602	-3.727221625	1.290996043	-3.389229799	-1.778871498	-4.73132696	1.457671031	-2.740286217	-3.646259651	-4.361858451	-0.639014588
-4.122586552	-3.986448953	-1.181453604	-2.669243161	-3.54422767	1.50302786	-3.759415995	-1.482694008	-4.237300259	1.579468261	-2.829559783	-3.087718239	-4.23433426	-0.622582802
-3.959572112	-4.222988907	-1.135809488	-2.932328966	-3.692669283	1.510767735	-3.822256605	-1.507315104	-4.550986845	1.863061013	-2.57925277	-3.39369333	-4.27304899	-0.78305503
-3.803609655	-3.771519427	-1.341527987	-2.2696055	-3.520898292	1.343884992	-3.548062923	-1.591833646	-4.605039512	1.69610455	-2.654326567	-3.404624387	-4.174452862	-0.74265856
-3.917483654	-3.971563404	-1.065118624	-2.597649912	-3.638024382	1.424651043	-3.540405894	-1.73750497	-4.603121159	1.798818342	-2.821243008	-3.657120405	-4.380487209	-0.869657716
-4.199511092	-4.188272767	-1.306382541	-2.858106223	-3.866807799	1.356504486	-3.770925929	-1.688053933	-4.644284554	1.5069602	-2.829784599	-3.550580888	-4.452238019	-0.82125046
-3.806739511	-3.9274411	-1.198869848	-2.814593662	-3.83646345	1.711416961	-3.673836891	-1.855856442	-4.537783555	1.886838654	-2.639273511	-3.691825904	-4.315868555	-0.709127492
-3.895994424	-4.371939799	-1.111588507	-2.392656482	-4.063321702	1.336314198	-3.961091058	-1.82690946	-4.832096004	1.736048019	-3.062971742	-3.805947113	-4.33318908	-0.894009308
-4.751526551	-4.045915998	-1.665310263	-2.840857776	-4.126787359	1.530572657	-3.636058959	-2.014470594	-4.965068709	1.342251333	-2.665734116	-3.969848518	-4.700813338	-0.853187613
-4.636894419	-4.340884838	-1.734558445	-2.721281071	-4.245915281	1.169940622	-3.707018083	-1.929392954	-5.131770718	1.280945595	-3.036548542	-4.165275211	-4.819103614	-0.699054604
-4.20268976	-4.269988958	-1.542038525	-2.591317691	-3.765334339	1.366992659	-3.621212919	-1.925823663	-5.230667089	1.589592353	-2.999831677	-3.6850003	-4.479185621	-0.774684416
-4.159905244	-4.148809508	-1.329739607	-3.154838661	-4.091263117	1.716359711	-4.133413829	-1.935880488	-5.188266646	1.900806403	-2.950759078	-3.79435758	-4.585228536	-0.913521148
-4.016093176	-4.167743654	-1.536070443	-2.430925347	-4.14550168	1.425298545	-3.735989573	-1.930788109	-4.954075419	1.570405318	-2.974627026	-3.901118704	-4.593894327	-0.929093193
-4.292918909	-4.176166958	-1.30905839	-3.115133516	-4.122978305	1.5993188	-4.008970124	-1.926895243	-5.022153579	1.705666199	-2.706302228	-4.03704116	-4.577396301	-1.106886633
-4.2182471	-4.479621909	-1.71346349	-2.781367664	-4.261335193	1.284349984	-3.745026141	-2.559280011	-5.484874606	1.513880631	-3.291619251	-4.452677918	-4.831960973	-1.049320941
-4.515452758	-4.144775656	-1.637747279	-2.600497289	-3.951523961	1.30991738	-3.725246257	-2.008392772	-5.08400747	1.348857074	-3.087316111	-3.957774742	-4.656451661	-0.908269314
-4.134720863	-4.24037979	-1.337312567	-2.687969217	-4.125374974	1.515831085	-4.062282446	-1.987394524	-4.918924445	1.656050183	-2.768737966	-3.99676799	-4.420167034	-0.841814803
-4.174653299	-4.276780437	-1.286130771	-3.001819494	-4.200453684	1.50930452	-3.890917369	-1.88167595	-5.166148907	1.812155274	-2.66551005	-3.719077797	-4.558390335	-1.069196398
-4.077574032	-3.989345986	-1.256478529	-3.24801376	-4.241606941	1.704052096	-4.208036083	-1.860504041	-5.253949581	1.818689708	-2.897439419	-3.628722215	-4.661901605	-1.174170931
-4.234378873	-4.159947661	-1.254441041	-2.702225567	-4.016362825	1.458392675	-3.87076395	-1.851808232	-5.035151105	1.418024358	-2.918033097	-3.800474126	-4.453821188	-0.583061634
-4.587493696	-3.640208352	-1.304532513	-2.285886137	-3.680399211	1.77729006	-3.564009807	-1.80769138	-4.757516852	1.192290354	-2.673339198	-3.853038125	-4.371597113	-0.534847906
-3.840980739	-4.30501533	-1.317064994	-3.004326263	-4.137663236	1.551868797	-4.068007704	-1.88302501	-4.868428935	1.864271848	-3.297119817	-3.763039557	-4.627840432	-0.946059729
-4.271233325	-3.59327353	-1.4787749	-2.575350798	-3.711954304	1.789969652	-3.924666141	-1.739668593	-4.801127742	1.205079861	-2.635220441	-3.626132566	-4.285241898	-0.625038722
-4.6224267	-4.447849174	-1.446333947	-3.018352541	-4.186507222	1.221191636	-4.021392902	-2.045896484	-4.924051442	1.492466586	-3.116301075	-4.147469529	-4.670581349	-1.14500585
-4.889836946	-3.967212899	-1.598922425	-2.917384668	-3.913247994	1.509300831	-3.817614818	-2.082334408	-4.963413626	0.988250015	-2.945541931	-3.961582559	-4.584113181	-0.910889963
-4.272589872	-4.023789548	-1.375247897	-3.095989285	-3.705734096	1.67603402	-3.437904004	-2.118306856	-5.208507245	1.719018552	-2.663872024	-3.764181335	-4.629676287	-1.103302125
-4.088484079	-4.229041042	-1.134602382	-2.283656074	-3.915762259	1.469858841	-3.663802198	-1.889652498	-4.554033004	1.558387466	-2.777239236	-3.755297706	-4.496180677	-0.774085198
-4.098549288	-4.110624751	-1.036120472	-2.001828978	-3.913435926	1.373016091	-3.779457196	-1.994385838	-4.685317111	1.477800484	-2.801472036	-4.367331731	-4.455996736	-0.586530665
-5.593098469	-5.071155428	-2.373208226	-3.70683535	-4.540195247	0.688338955	-3.917418071	-2.621331032	-5.709412362	0.590584574	-3.155833805	-4.676277506	-5.308458736	-1.618373621
-4.205838451	-4.667347456	-1.298661041	-2.526796937	-3.967549303	1.420366299	-3.191238182	-2.1009097	-4.902587666	2.040613866	-2.77088816	-4.396228633	-4.598432414	-0.899483812
-3.976141563	-4.542138004	-0.209532816	-2.602485482	-3.964460514	1.86865335	-3.509103483	-1.919208134	-5.316520036	2.297002436	-2.821172372	-4.739091424	-4.532261926	-0.590759627
-4.113623954	-4.727437861	-1.803462232	-2.817531836	-4.387716709	1.291286736	-3.927377873	-2.081053049	-5.869232662	1.570820439	-3.294241175	-4.343569934	-4.917636103	-0.989032149
-3.740594763	-4.635890821	-0.927678968	-3.1113272	-3.734138551	1.771998844	-3.631509439	-1.426720692	-4.92020917	2.47292713	-2.543641052	-3.209686293	-4.163678073	-0.735434738
-4.578044872	-5.482771318	-1.439270226	-3.735016618	-4.864877796	1.208992305	-4.454395676	-2.136024369	-5.057164008	2.336372674	-3.327779498	-5.393740423	-4.795601292	-0.614101237
-3.484685815	-4.707812191	-1.355096786	-3.192622205	-3.993284826	1.144963636	-3.78908702	-1.902568028	-5.166042471	2.220325843	-3.455976382	-4.342754891	-4.582693661	-0.780790074
-3.838317448	-4.675365321	-1.784824613	-2.426759004	-3.887032944	1.22536475	-3.646792774	-1.786240955	-4.756516784	1.769379636	-3.199819365	-3.85543863	-4.531875161	-1.001496921
-4.561714793	-4.895731088	-1.213310362	-3.741498391	-4.122617434	1.084193983	-4.020455599	-2.139448129	-5.066775186	2.147565363	-3.14814689	-4.312116747	-4.418195567	-1.123996391
-4.007454458	-4.459258916	-1.183865178	-3.112907764	-3.933103048	1.211035538	-3.871188603	-1.562270439	-5.333010796	2.015149817	-3.05992591	-3.176075947	-4.590484948	-1.025334732
-4.410718786	-4.915082924	-1.375712853	-2.804102434	-4.108717362	1.185253914	-3.92902641	-2.163985174	-4.855168267	2.070726616	-3.304002242	-5.347858074	-4.371523243	-0.42156544
-4.823128836	-5.30265034	-2.23787244	-2.614577223	-4.457604426	0.793880714	-4.391711233	-2.469989183	-5.228358329	1.742402261	-3.76699006	-6.356846283	-4.781123051	-0.987413257
-4.603941773	-3.887372197	-1.708608821	-3.087830519	-3.566160048	1.279548155	-3.574676742	-0.949723788	-5.368465297	1.547739348	-2.762106808	-2.418767452	-4.082726975	-0.271795162
-4.429173402	-5.380744704	-1.599443746	-2.753071779	-4.246366957	1.310258589	-4.447103704	-1.902238258	-5.25187783	2.157665414	-3.324936917	-4.937046336	-4.675760754	-0.659278841
-3.804346384	-4.899001081	-1.576629597	-2.885178061	-4.380938085	1.030546881	-4.08636876	-1.990187695	-5.285789972	2.063120302	-3.274746606	-4.28816924	-4.68386743	-0.912586732
-3.515954812	-4.078933847	-1.576681442	-2.386291542	-4.084519226	1.553296936	-3.655092947	-1.741518441	-5.37693134	1.568639504	-3.049372399	-3.640963652	-4.507274132	-1.194631783

-3.943687008	-4.623495702	-1.431709784	-2.112745075	-4.076430103	1.182103582	-3.923413089	-2.035166202	-5.412044109	1.93293562	-3.380028662	-4.44172111	-4.679680505	-0.970835093
-4.332028298	-5.106715437	-1.302263949	-3.290951087	-4.226761397	1.346597792	-3.999483319	-1.70152421	-4.845460087	2.583660414	-3.015224613	-3.921449407	-4.849582843	-0.597836586
-3.674708875	-4.400552244	-1.043065115	-2.669827355	-3.867299084	1.617077461	-3.204682984	-1.57644491	-4.699407785	2.419324887	-2.6456011	-3.42099629	-4.34639293	-0.873846353
-4.28918682	-4.155105602	-1.926863197	-2.868304377	-3.532467133	1.143659788	-3.374727228	-1.30340152	-5.149925691	1.025282516	-2.667037828	-2.863191206	-4.209948314	-0.461283895
-4.924677114	-4.478997047	-1.53977364	-3.098324405	-4.04075157	1.265254977	-4.105019928	-2.011580041	-5.216940367	1.331426229	-3.148443056	-3.967686484	-4.579917967	-0.837864286
-5.501949456	-5.036795676	-1.700184076	-2.973353022	-4.194196021	1.048932465	-3.591271986	-2.137069211	-3.552843964	1.943006481	-3.144079319	-5.033607696	-4.430997881	-0.756162484
-4.093854417	-5.449025913	-1.545786877	-3.168567708	-4.626009098	1.642914566	-3.277937347	-1.648967351	-3.636476883	2.309114126	-2.749334921	-3.48272605	-4.291788037	-0.538567387
-4.586510936	-4.838633603	-1.609512704	-3.450464443	-3.897029547	0.951389261	-3.875226076	-1.923088292	-5.176174383	1.503249117	-3.103254132	-4.43420934	-4.510313076	-0.961199287
-3.798702686	-5.035653361	-1.390560065	-2.783857484	-4.087704605	1.630197477	-3.865184909	-1.895306499	-5.30256903	2.413494041	-3.171989355	-5.161586655	-4.446027736	-0.491732838
-3.846543744	-4.78785298	-1.219381209	-2.240847178	-4.151232402	1.526693293	-3.66426396	-1.983757962	-4.909910169	1.998578455	-3.124543747	-5.061179931	-4.336551986	-0.553268753
-5.279652833	-5.163455842	-1.964730332	-3.531610383	-4.470165872	0.749366879	-3.988808927	-2.374931996	-5.419388082	1.706103649	-3.615679362	-5.406098782	-5.113394179	-0.674128571
-3.971871711	-4.903060853	-1.357976436	-2.713476022	-4.202541577	1.168526664	-3.868309288	-2.001219005	-4.78360097	1.882043593	-3.485831479	-3.967031316	-4.410714895	-1.098767013
-4.539907727	-4.84839184	-1.537473655	-3.862408366	-4.332120382	0.962938279	-3.905340942	-2.155965236	-5.24210748	1.481544724	-3.421859268	-4.284842575	-5.037996141	-1.093312245
-4.648621399	-5.662857804	-1.912195126	-2.877559837	-4.434618285	0.876493157	-3.808305413	-2.385373839	-5.010750461	1.74919242	-3.603668176	-5.2224869	-4.903689691	-1.056689367
-5.37635942	-6.273262723	-1.979751799	-3.591928604	-5.085170115	0.635532604	-3.864758136	-2.626860521	-4.576229681	1.63833276	-3.541501577	-5.436135355	-5.192779049	-0.815430903
-3.893665338	-5.228767616	-1.284404639	-3.048821305	-4.322133888	1.265104875	-3.776975145	-1.985972198	-4.610121367	2.185831384	-3.002071422	-4.028575348	-4.695294265	-0.745327249
-3.882514834	-4.79930213	-1.452000947	-3.507442555	-4.072307848	1.070477141	-3.970362867	-1.937254989	-4.675033263	1.970086483	-3.159684133	-4.081717507	-4.643633752	-1.076333389
-4.556085382	-4.208702034	-1.472870425	-3.437437511	-3.48442271	1.094361198	-3.428785018	-1.860386415	-5.210920864	1.392908116	-3.039892983	-3.547368165	-4.283081345	-1.329151082
-3.504600977	-4.51348411	-1.087674106	-1.991096858	-3.99744533	1.719716338	-3.352429631	-1.830243337	-5.252413646	1.844605847	-2.762408532	-4.30327074	-4.394908283	-0.527616878
-4.326143669	-5.241409454	-1.330417985	-2.111060729	-4.565890995	1.020234252	-4.183557955	-2.432874889	-4.762561047	1.869684773	-3.499377511	-5.252369477	-4.583758997	-0.631819778
-4.340715558	-4.54187339	-1.233834845	-2.886417296	-4.05703776	1.177272378	-3.967368486	-1.570105015	-4.757066629	1.927196094	-2.848783868	-3.386498864	-4.585465735	-0.961782736
-4.829838597	-5.145346766	-1.977596442	-2.826119289	-4.562200152	0.38266791	-3.593371264	-2.249057706	-3.363363943	1.289957676	-2.870950013	-4.339315926	-4.84814867	-1.462410871
-4.614305978	-4.839280532	-1.967950461	-2.103276164	-4.519350211	1.064852946	-4.249749043	-2.402753827	-5.452636198	0.935755058	-3.225005285	-4.934948784	-5.115173	-0.906966574
-5.111351418	-5.291434182	-0.984134864	-3.033793587	-4.95556521	1.466278662	-3.703596902	-2.146938374	-4.767092915	1.890323292	-2.662620454	-4.94198844	-5.470852645	-0.74399226
-4.716322666	-4.620665561	-1.378847936	-2.560658902	-4.380872682	1.231066359	-3.689845562	-2.047681647	-4.931938988	1.645672133	-3.130089622	-4.093969051	-4.988850856	-0.720273938
-4.228937082	-5.251376737	-1.712532439	-3.294456401	-4.297948422	0.591909195	-4.004531861	-2.097361492	-3.7352212	1.85969415	-3.196975473	-4.242564209	-4.697023488	-1.628426694
-5.204635277	-4.595952737	-1.37051064	-3.059930525	-4.403393691	0.968927327	-3.27714451	-2.184006051	-2.69115438	1.487069633	-2.663486689	-4.111442319	-4.901805904	-1.465377407
-5.183856132	-4.551707234	-0.986406457	-3.359665364	-4.70009667	1.177348607	-3.555066695	-2.041881608	-2.969731537	1.605764173	-2.520748715	-4.240822843	-4.820908329	-1.572094883
-4.986278963	-4.874757981	-1.673245663	-2.932800677	-4.438394388	0.831347414	-3.342237329	-2.082944059	-3.235912715	1.471358542	-2.740370351	-4.117529618	-4.751683222	-1.432148372
-4.005337949	-5.1839603	-1.700248259	-2.888542552	-4.370324744	0.526514461	-3.903707642	-2.18227279	-3.657290936	1.686484115	-3.121952429	-4.080416734	-4.59934669	-1.257204648
-5.035423109	-4.942846582	-1.623058251	-2.987809746	-4.649113208	0.59028883	-3.12181312	-2.168746474	-2.663793892	1.574685951	-2.678914684	-4.36140007	-4.865660608	-1.293223376
-4.577500736	-4.804889277	-1.360201001	-3.163024129	-4.354199001	1.13300785	-3.755334608	-2.143466113	-3.505701553	1.709781111	-2.716111413	-4.060915302	-4.669142091	-1.460798404
-5.013612553	-4.903333931	-1.688236498	-2.604540418	-4.533358846	0.483263535	-3.444915999	-2.217305768	-2.744223019	1.252091395	-2.632436427	-4.75746856	-4.909018385	-1.407118526
-4.759410305	-4.720974621	-1.570966123	-1.794355388	-4.425190608	0.623804262	-3.143368345	-2.058187484	-2.803971403	1.349855681	-2.646826979	-3.969179294	-4.855466428	-1.374967975
-4.424840959	-4.846738505	-0.83550745	-3.090099777	-4.148669168	1.103269458	-3.775730705	-2.301175325	-5.52226648	1.609435213	-3.636130993	-4.705851522	-5.000001692	-0.993194171
-3.514669031	-4.57985363	-1.382415146	-2.621550105	-3.874497319	1.571539481	-3.83131819	-1.969964683	-5.849681196	2.329649075	-3.14199153	-4.030201853	-4.36727844	-0.789334623
-4.111936933	-5.011211136	-1.468959485	-2.861585796	-4.158653948	1.074007462	-3.734751964	-2.25688381	-4.547093918	2.145517019	-3.763285416	-4.970654518	-4.448361054	-0.774731692
-3.903868937	-5.17323659	-1.303255714	-2.696067953	-3.974274098	0.904424	-3.750008163	-2.138058941	-4.927761367	2.29232615	-3.327528649	-4.886854449	-4.455441604	-0.630704892
-3.411676326	-4.893002946	-1.200384236	-2.84332345	-4.171581657	1.217728244	-4.402351533	-2.060322077	-5.371264935	2.24867212	-3.239886854	-4.444485727	-4.655372088	-0.852043392
-4.55484207	-5.273957745	-1.766649519	-3.241220101	-4.19200475	1.052850222	-4.334418783	-2.149575475	-5.436219579	2.038379058	-2.722965852	-2.434156114	-4.688701577	0.193557907
-4.230695885	-5.042838443	-1.207429877	-2.988058287	-4.195781501	1.104565772	-4.044067064	-2.141846289	-5.054879364	2.114588921	-3.523833012	-4.743268039	-4.455381417	-0.704024722
-3.920318867	-4.915128849	-0.9249869	-2.418481311	-4.281382715	1.218202113	-4.111274217	-2.084577969	-5.158544818	1.968836444	-3.565770386	-4.699376138	-4.406790483	-0.826502871
-5.195001656	-5.428760082	-2.296842929	-2.638394979	-4.460894234	0.850814051	-4.112279522	-2.143963634	-5.053294009	1.43492437	-3.590435689	-5.464543921	-4.942033976	-0.674776494
-5.198887167	-5.63444163	-2.267513186	-3.622349704	-4.679381296	0.655176865	-4.073829461	-2.402270107	-5.749612559	1.218454697	-3.230432184	-5.150325438	-5.09011375	-1.159382741
-5.187293619	-4.800174078	-1.380609855	-3.882631555	-4.634400322	1.057320618	-4.276618596	-2.458395919	-5.244380358	1.251462493	-3.271408537	-4.300366998	-5.166111813	-0.983820358
-3.803769915	-5.491861493	-1.501497862	-3.526312225	-4.435880602	0.788358876	-4.016391009	-2.470879522	-4.496612816	1.926193132	-3.933955888	-4.760833768	-4.787847551	-1.212624458
-3.350923647	-6.127426741	-0.936548876	-2.633678532	-4.016379562	1.554469613	-3.969892799	-1.95755941	-5.889845757	2.235683605	-2.860944186	-4.031777992	-4.6806592	-0.67437351
-4.31653684	-5.207101013	-1.587516819	-2.6313369	-4.035220085	0.968681096	-3.834499358	-2.417111917	-5.114639439	1.416897936	-3.484809957	-5.0804832	-4.773046321	-0.602971411
-4.445830442	-5.136221175	-0.717244939	-2.829368636	-4.435211291	0.974661563	-4.200367136	-2.033801991	-4.046977562	2.390259162	-3.40475969	-4.543794393	-4.624652732	-0.613263403

-4.657552298	-4.672399329	-1.386614794	-2.649069685	-4.466664057	0.906419036	-3.13685878	-1.87575725	-2.423238278	1.74031105	-2.325980247	-3.981685588	-4.623023678	-1.411166214
-4.636642192	-4.866808537	-1.505660178	-2.665510777	-4.229714423	0.491497231	-2.982460811	-2.121989427	-2.333295869	1.654307349	-2.738385864	-4.235161342	-4.656364805	-1.258901457
-4.589708054	-4.871319776	-1.506315594	-2.645158291	-4.578379427	0.451467942	-3.357773414	-2.02371826	-2.475194734	1.634272955	-2.722298674	-4.2309911	-4.605584933	-1.368651404
-4.59589851	-4.636771266	-1.366554537	-2.932251514	-4.461526817	0.770830243	-3.275240064	-2.051432383	-2.899222517	1.825981627	-2.688097743	-4.316804163	-4.788202277	-1.359240956
-4.972832663	-5.080801682	-1.774005166	-2.768964816	-4.527058484	0.495278736	-3.600108141	-2.084177084	-3.222573534	1.408992353	-2.923723951	-4.011611822	-4.788486307	-1.476692647

hsa-miR-6859-3	hsa-miR-6860	hsa-miR-6861-3	hsa-miR-6861-5	hsa-miR-6862-3	hsa-miR-6865-3	hsa-miR-6865-5	hsa-miR-6870-5	hsa-miR-6872-3	hsa-miR-6875-5	hsa-miR-6877-3	hsa-miR-6877-5	hsa-miR-6879-3	hsa-miR-6879-5
-3.863841887	-3.707893086	-3.775319564	-3.092857294	-3.52452093	-4.091402779	-4.209973792	-1.083365839	-4.678039938	0.653836178	-4.098852762	-2.31883399	-3.809871582	-1.060651855
-4.214737239	-3.730356747	-4.063307568	-2.78894642	-4.023926818	-4.247127064	-4.520283971	-1.257028417	-4.544158979	0.928787544	-4.211372066	-1.991269462	-3.65990297	-0.316865832
-3.971248393	-3.756366526	-3.832544483	-2.937765424	-3.856129567	-4.252876994	-4.739677272	-1.061102253	-4.584630542	0.593857148	-4.123366852	-2.059950185	-3.68030667	-0.294042602
-4.043687899	-3.695395678	-3.859803881	-2.953674839	-3.919302251	-4.079507474	-4.555036114	-1.00084785	-4.45491424	0.559569988	-4.221549432	-1.926747231	-3.697481653	-0.120766155
-3.640713154	-3.729839027	-3.773179383	-3.24045431	-3.533376092	-3.675333777	-4.115807274	-0.676858996	-4.355692436	0.485613192	-3.990966847	-1.479117548	-3.412758544	-0.579637351
-3.789490941	-3.568263549	-4.020759232	-3.013783895	-4.152706711	-4.293678878	-4.676971331	-1.215648761	-4.106356489	0.614242949	-4.273818571	-1.429256823	-3.491821115	-0.58862444
-3.766142241	-3.571299687	-3.659240852	-2.787477142	-3.471804478	-4.043689408	-4.428084663	-0.926903085	-3.891615013	0.656827537	-4.007249971	-1.932151699	-3.45780351	-0.409580451
-4.123988457	-3.763323801	-4.147057005	-2.910734422	-3.717484379	-4.302408825	-4.665096782	-1.461630503	-4.217000148	0.484672	-4.060289807	-1.828262087	-3.573854071	-0.009092433
-4.116214324	-3.75389542	-4.150189722	-3.236827012	-4.036967314	-4.334019991	-3.490894886	-1.382072427	-4.544336224	0.493661289	-4.242377257	-1.94489538	-3.708160067	-0.165777934
-4.117878387	-3.701221087	-4.149619666	-2.848566947	-3.83067863	-4.172457204	-4.37801203	-1.667547608	-4.217938007	0.657159628	-4.211153989	-1.504305258	-3.680706785	-0.403580169
-3.927826909	-3.992740067	-4.244518852	-3.161502252	-4.162208725	-4.549670643	-4.985375843	-1.669030263	-4.004441607	0.404887357	-4.482784923	-1.969435966	-3.785647985	-0.163568243
-4.346542919	-4.003601223	-4.324502369	-2.97982991	-4.49668032	-4.748001945	-4.513196051	-1.471273786	-5.072687056	0.831204916	-4.618751045	-1.9895059	-3.927919023	-0.472804878
-4.436799826	-4.301199633	-4.37911728	-3.083262917	-4.610382438	-4.690411176	-4.614209796	-1.480249714	-4.927231426	0.427165871	-4.673694361	-1.944705023	-4.006840238	-0.34061382
-3.995658172	-4.149516973	-3.949869527	-2.974397938	-4.306003806	-4.68707214	-5.064743867	-1.553114397	-4.379021047	0.640015091	-4.521805684	-2.095923042	-4.028374827	-0.464759522
-4.305661305	-4.212075313	-4.468598409	-3.011521527	-4.583419974	-4.584858933	-4.82832819	-1.73107518	-4.274133269	0.740110133	-4.467778547	-1.980753676	-3.877540835	-0.648855296
-4.251366821	-4.143731239	-4.314501411	-3.173476292	-4.497515505	-4.624993941	-4.788636348	-1.4912362	-4.764545381	0.549600594	-4.571612671	-2.20590758	-3.770560098	-0.454119622
-4.211815177	-4.035906393	-4.393903646	-2.860207071	-4.521960982	-4.743843833	-4.736167509	-1.751780553	-4.381289221	0.733565948	-4.550534461	-2.097540786	-3.846092861	-0.360160812
-4.309230531	-4.124646575	-4.272948704	-2.851277859	-4.414532156	-4.917158379	-5.172345311	-1.810730159	-4.853989197	0.380717681	-4.69841581	-2.215890387	-4.481777274	-0.634832141
-4.103486391	-4.063848436	-4.080630119	-2.856543507	-4.416206861	-4.625367491	-4.506485177	-1.374344648	-4.438376163	0.385120103	-4.58582956	-1.899329237	-4.061852923	-0.628672881
-4.215513669	-3.841462404	-4.446726866	-3.013877656	-4.579255363	-4.579796313	-4.765368116	-1.595305452	-4.051425561	0.622818896	-4.646040279	-1.875714638	-3.957628528	-0.113521095
-4.256985168	-4.155117682	-4.310658784	-2.939218748	-4.653469167	-4.671564959	-4.888389732	-1.71296856	-4.248440485	0.856988024	-4.588338776	-2.167053676	-4.048298132	-0.830324984
-4.306170338	-4.26530786	-4.569578362	-3.06975607	-4.615582631	-4.607395858	-4.891545979	-1.703218536	-4.452094063	0.794038084	-4.588900588	-1.999171268	-3.845392335	-0.809385284
-4.012217496	-4.086215458	-4.150232585	-3.12300882	-4.500643509	-4.539313941	-4.730201162	-1.128203065	-4.576450019	0.485061547	-4.46655507	-1.742547311	-3.87392354	-0.021424545
-3.776520632	-3.815816431	-3.802089438	-2.800411278	-4.012912214	-4.124717811	-4.033619976	-0.8297441	-4.488836301	0.71225918	-4.320964573	-1.868644195	-3.810587636	-0.333634558
-4.260253895	-4.020474471	-4.431073238	-3.193052426	-4.651324384	-4.602657134	-4.998118281	-1.423232716	-4.608546455	0.551424999	-4.643929126	-2.036164264	-3.900327133	-0.257405351
-3.573588295	-3.866004101	-3.742146794	-3.131572026	-4.200541893	-4.325357772	-4.428100425	-0.935169863	-4.653941691	1.054170065	-4.395801145	-2.123512127	-3.816021704	-0.445355501
-4.424527887	-4.178962364	-4.587071653	-3.206002927	-4.7240949	-4.533504041	-4.825836144	-1.693619526	-4.620400574	0.634522416	-4.858883284	-1.789352737	-3.893048048	-0.281738183
-3.969977004	-3.804980663	-3.966065947	-3.027447529	-4.422552518	-4.470154877	-4.452265239	-1.198650612	-4.615558302	0.686193239	-4.468450508	-1.973986704	-4.049507335	-0.355126756
-4.052486922	-3.675333872	-4.21850246	-2.869557737	-4.309341414	-4.412611035	-4.884687276	-1.310388092	-4.740966147	0.909484409	-4.331174861	-2.372977118	-4.188561767	-0.937618531
-4.121727719	-3.856724429	-4.148315334	-2.910810194	-4.103502435	-4.394332852	-4.75000796	-1.513876696	-4.397216504	0.751336248	-4.439268701	-1.49664458	-3.77257259	-0.107881351
-4.305504341	-3.807542181	-4.281410805	-2.965565047	-4.179249104	-4.385994306	-4.63785163	-1.572682786	-4.501929807	0.496410019	-4.286149343	-1.393494438	-3.815166143	-0.026755909
-4.923587816	-3.916780897	-5.052670736	-3.59844628	-4.933547388	-5.073397328	-4.514278161	-2.118113644	-4.945013265	0.266261482	-5.258838313	-2.801033007	-5.057952759	-0.517358237
-4.693804254	-3.760454125	-4.583234375	-2.816649589	-4.513398116	-4.402833142	-4.091521032	-2.121292417	-4.291597282	0.667966834	-4.558283668	-2.087991987	-3.824524008	-0.609512428
-4.636310872	-3.774473541	-4.605623246	-2.649784151	-4.389316123	-3.757414624	-5.260880463	-2.520672506	-4.477156371	0.910484724	-4.43404015	-2.244357971	-3.535681455	-0.769486191
-4.446631406	-4.479701679	-4.717730199	-3.179605399	-4.932230941	-4.959141131	-5.80789479	-2.143033601	-5.291567219	0.662189681	-4.830221068	-2.69741403	-4.442441909	-0.756117267
-4.106456971	-3.829731221	-4.425248284	-2.928350292	-4.273690538	-4.346621668	-5.084305381	-1.87217352	-4.67301815	1.080125907	-4.173134532	-2.15434576	-3.54927358	-0.58184
-5.311235889	-4.628317939	-5.06905927	-2.741679407	-6.255811863	-3.502395417	-6.388903024	-2.828183753	-6.139922561	0.188492375	-5.456332742	-2.496249559	-4.616241248	-1.22373967
-4.354414765	-4.399130946	-4.5005846	-2.903649108	-4.390273699	-4.673821185	-5.790081445	-2.088116366	-4.822918282	0.237708206	-4.541074638	-2.480119402	-4.002078515	-0.917469211
-4.246334779	-4.407322552	-4.17356421	-3.180310591	-4.394372473	-4.372921135	-5.558338031	-1.440746922	-4.944100879	0.391931668	-4.382630224	-2.543591849	-3.86302399	-0.769282049
-4.281043691	-4.334993535	-4.703534949	-2.844236771	-4.589126502	-4.117513673	-5.46584326	-2.755449045	-5.074275912	0.423939291	-4.464103733	-2.54066543	-4.267047346	-1.770898185
-4.168085545	-4.332046326	-4.413245247	-3.112688167	-4.386177912	-4.602099656	-5.074215779	-1.850821199	-4.862591968	0.442650581	-4.324345263	-2.387353195	-4.090681894	-0.945152366
-4.601173327	-3.971903037	-4.529959897	-2.791376811	-4.521099384	-4.016502034	-5.57534856	-2.154740393	-5.180465466	-0.117056971	-4.433891181	-1.572745908	-4.088260148	-0.402265886
-4.747818029	-4.181476667	-4.890297271	-2.824223617	-4.97205318	-3.751616842	-5.227300224	-2.74097542	-5.470377768	-0.971247291	-4.928089072	-2.456793324	-4.398467349	-0.647457103
-2.926728005	-4.033996474	-3.627494001	-2.929816776	-4.114161148	-4.106470332	-3.757582307	-1.005355202	-4.759004892	0.072170207	-4.227393015	-2.541858254	-4.093301116	-1.210495158
-4.65776768	-4.350892505	-4.832938011	-3.084003083	-4.861679593	-4.299436425	-5.478036748	-2.161764126	-5.251544963	0.431570187	-4.84720867	-2.322602219	-3.863431891	-0.867408214
-4.458737498	-4.379788613	-4.845888393	-3.23852459	-4.809902549	-4.623508314	-5.886544998	-2.282559176	-5.345726471	0.386124315	-4.804221782	-2.278059323	-4.281783796	-0.726479322
-3.942295218	-4.145278976	-4.198328906	-3.262392972	-4.140518997	-4.356473845	-5.06828959	-1.160800076	-4.844945063	0.56530953	-4.441906761	-2.336252184	-3.693308586	-0.492628252

-4.513045856	-4.119439254	-4.486261575	-3.05155757	-4.459734228	-4.584422223	-5.640001526	-1.80326185	-5.100446164	0.243629784	-4.512698106	-2.0945696	-4.014708466	-0.18233775
-4.583224675	-3.822644615	-4.728079534	-2.551776653	-4.888023076	-3.51359774	-4.87239937	-2.624806017	-4.845341425	0.503074253	-4.705248468	-2.269455595	-4.00393207	-0.914629401
-4.161181289	-3.868681646	-4.26635009	-2.614426725	-4.456073169	-4.489949588	-5.268927586	-1.723880852	-4.861476716	1.298597817	-4.451089668	-2.022729885	-3.690187911	-0.864051119
-3.166673862	-3.617439627	-3.657148433	-2.93420337	-3.816655142	-4.268799023	-3.52684284	-0.844239181	-4.848291746	0.302031668	-4.225821652	-2.524986946	-3.982875458	-0.149749222
-3.696921574	-4.4620289	-4.247316804	-3.262100379	-4.4523162	-4.537174949	-4.676820669	-1.500125195	-5.006427305	0.371109679	-4.605888293	-2.650268573	-4.288334019	-0.724079191
-4.603761768	-3.560910871	-5.020190812	-2.288905782	-4.732777611	-3.588657773	-4.638107743	-2.819280507	-4.627693444	0.175646102	-4.934192989	-2.058780957	-4.349215347	-0.60286486
-4.948941554	-3.834849756	-4.887823952	-2.207403673	-4.213320817	-4.613158069	-4.695263705	-2.632690446	-5.297802016	0.824281156	-5.431629157	-1.947038503	-4.057792885	-0.327168659
-3.795656269	-4.218630742	-4.350326975	-3.271878981	-4.567350399	-4.532021994	-4.338913373	-1.773652545	-4.846654842	0.151931629	-4.679694499	-2.940553864	-3.960859471	-0.754777801
-4.45820614	-4.019218314	-4.713163399	-2.765957179	-4.635112256	-3.576398643	-5.116618815	-2.228028873	-4.959379642	0.727637233	-4.645511992	-2.087097412	-3.951968064	-0.543882757
-4.461144423	-3.992511261	-4.523130295	-2.922096367	-4.653193744	-4.239385577	-5.174625434	-1.9804104	-5.023448944	0.404711716	-4.577675388	-2.08356244	-3.924957755	-0.279968843
-4.87389434	-4.251829237	-4.775475661	-2.749427757	-5.448646529	-3.560623313	-4.994243399	-2.496336018	-5.020999022	-0.600670092	-5.294695623	-1.865354393	-4.567841288	-0.724782402
-4.640308094	-4.058010033	-4.53795352	-3.039631513	-4.679331111	-4.022186018	-4.897477844	-2.200541645	-4.598699761	0.049439821	-4.837830769	-2.007215965	-3.894193267	-0.375243939
-4.387665529	-4.503983355	-4.681134206	-3.182806585	-4.719302288	-4.886530021	-5.294534702	-2.254222147	-5.270583182	0.12368789	-4.863371631	-2.856637513	-4.413251437	-1.407623886
-5.070971495	-4.1954864	-5.0057396	-2.673667779	-4.984152303	-3.654452804	-5.247035238	-3.019895159	-5.147795786	-0.281027851	-5.113403137	-2.537184019	-4.429021483	-0.956084963
-4.963693094	-3.972827221	-5.725632751	-2.853063339	-5.392582033	-3.679186581	-5.282384713	-3.094600919	-5.54251248	-0.10098114	-5.635862868	-2.401059096	-5.582257619	-0.750722023
-5.067323934	-4.227697012	-4.848994747	-2.822721819	-4.882993948	-4.580140417	-5.325021287	-2.415875361	-5.635875527	0.723118864	-4.661223264	-2.199670585	-4.287944246	-0.906190722
-4.541257474	-4.516640805	-4.57108181	-3.108176209	-4.700479022	-4.160252576	-5.984777732	-1.948151379	-4.981055961	0.252866474	-4.644254708	-2.019163783	-3.9832198	-0.805105799
-3.751689823	-3.851049266	-3.790030842	-2.853707726	-3.794528042	-4.10894444	-4.772486998	-1.378705573	-5.044876716	0.181444428	-3.901587515	-2.689597429	-4.044483139	-1.333188496
-4.057820146	-3.886636511	-4.212534441	-2.972542091	-4.426159157	-4.402702963	-4.913720024	-1.590016603	-4.741372039	0.845997248	-4.438020622	-2.248965645	-3.86846952	-0.514812719
-4.897986868	-4.153391998	-4.79127958	-2.897083177	-4.899490775	-4.271902255	-5.126497442	-2.537309068	-5.338667892	-0.038706833	-5.09266744	-2.097190614	-4.478035944	-0.411278165
-4.287775334	-4.032088824	-4.337651653	-2.81494124	-4.396430086	-4.343610161	-4.512651485	-1.704579569	-4.805559071	0.629362594	-4.450187937	-2.301424778	-3.723462299	-0.180058834
-5.051878435	-3.708003224	-5.027023767	-2.340582006	-4.886605361	-4.546028314	-4.473666364	-2.387125609	-3.05436859	-0.129837846	-4.955640423	-2.132885285	-4.108325434	-0.174780699
-4.991704531	-4.004220919	-4.877014944	-3.3580142	-5.103069064	-5.020639874	-5.073220495	-1.948512434	-5.460030931	0.093205018	-5.218735626	-2.514842538	-4.584238305	-0.347223095
-5.739494794	-4.159202765	-6.277616999	-2.717353014	-5.452835828	-4.59570636	-5.312923743	-2.692536902	-5.015794715	0.744109012	-5.669153982	-2.128092311	-4.301028319	-0.775844854
-4.643161932	-3.918432464	-4.809412635	-3.005639711	-5.075348619	-4.870289911	-5.109968234	-1.839759711	-5.068113137	0.619136132	-4.966803512	-1.944130467	-4.296272456	-0.457593962
-4.794526711	-4.166868131	-4.945622302	-2.225950213	-5.077697718	-4.446376966	-4.679492429	-2.205109248	-3.266109228	0.202419847	-5.171450053	-2.118257017	-4.068503577	-0.166042757
-4.923974258	-3.500502966	-4.804600044	-1.725514599	-5.036108554	-4.538437651	-3.548517982	-2.352776236	-3.80090678	0.207336655	-4.877936926	-2.200012898	-4.161203104	-0.401585512
-4.800829763	-3.487726988	-5.027587833	-2.171320578	-4.749166164	-4.691528795	-3.859721061	-2.448867769	-4.403284702	0.654287096	-4.759395803	-2.405941693	-4.040535819	-0.67395896
-4.75256926	-3.506452324	-4.643717559	-2.271613106	-4.859260158	-4.690820188	-4.309450312	-2.097508045	-3.495698789	0.313607555	-4.854580701	-2.157162491	-3.936428872	0.09872229
-4.725193026	-4.005581766	-4.693382814	-2.517557674	-4.730543904	-4.435614713	-4.473962689	-1.933285157	-3.293696878	0.179885243	-4.783917811	-1.793328746	-3.970762845	0.18441423
-5.093071629	-3.18739084	-4.956420258	-1.77691955	-4.877078087	-4.820129705	-3.87341379	-2.421824918	-3.315298379	-0.077221639	-4.768163045	-2.112548885	-4.34002604	0.233682174
-4.714453184	-3.357136586	-4.693489941	-2.087891062	-4.84375899	-4.576175089	-4.362634557	-2.33816691	-3.947955073	0.445785027	-4.900933987	-2.011447118	-4.467960647	-0.283099775
-4.950032157	-3.436777859	-4.91275957	-1.943464113	-5.037475534	-4.273950743	-3.843026834	-2.431071832	-1.86896216	-0.242274133	-5.093664971	-2.24533902	-4.207136628	-0.08988947
-4.826336596	-3.22055547	-4.715855039	-1.574475381	-4.570699789	-4.583259228	-3.757550631	-2.384237718	-3.051031514	0.032080569	-4.748476234	-1.611208528	-4.001282411	-0.050569472
-4.529523652	-4.321913002	-4.533574387	-3.144427227	-4.511129839	-4.626740978	-5.534118731	-1.938948986	-5.374705233	0.288403285	-4.703376568	-2.799346405	-4.273153969	-0.75704587
-4.206589	-3.99745704	-4.298123364	-2.833441976	-4.069772945	-3.933957226	-5.698698511	-1.805131763	-4.875098093	0.721563579	-4.322522501	-2.446084294	-3.916044677	-0.865294499
-4.628923284	-3.791363655	-4.527582247	-2.494147944	-4.632742833	-3.407325513	-5.463437245	-2.457010535	-5.183503874	-0.439444165	-4.568021692	-2.015050929	-4.346548229	-0.206068647
-4.479731609	-4.118113158	-4.505550301	-2.767884456	-4.868086015	-4.077479399	-5.206360246	-2.119914915	-4.988478769	0.017040513	-4.859863283	-2.232031658	-4.110534808	-0.655803946
-4.481931717	-4.488443525	-4.774325861	-3.202674527	-4.490234085	-4.250225647	-5.232077813	-2.772951692	-4.761675838	0.855953315	-4.610539128	-2.418892108	-3.787576571	-1.351415336
-4.126230968	-4.157873268	-4.631372209	-2.727934412	-4.68582324	-4.364781443	-3.770318665	-2.160405685	-4.999387297	0.46956689	-4.592961181	-2.873723372	-4.060461541	-1.112074857
-4.452023044	-4.288382173	-4.676480838	-3.009485466	-4.739101225	-3.954650504	-5.163603343	-2.35967043	-4.791959398	0.06134336	-4.789432693	-2.120867032	-3.947575575	-0.461207257
-4.741677851	-4.053349405	-4.823691564	-3.123725629	-4.66279536	-4.409408376	-5.18516177	-2.094911454	-4.971859598	-0.157140065	-4.748660864	-2.05151282	-4.181723203	0.010197091
-4.956913094	-3.825213494	-4.911927861	-3.05332827	-5.102511143	-4.316248222	-5.070633972	-2.296729518	-5.26139275	-0.981188877	-4.959303148	-2.358868557	-4.244077922	-0.265430658
-4.562245726	-4.179929244	-4.970372778	-3.330387892	-5.189827604	-4.54527709	-4.802571563	-2.502808963	-5.77162568	-0.071265474	-5.213832086	-3.127285748	-4.687001327	-1.096570297
-4.895273475	-4.251836015	-4.970315602	-3.113786832	-5.000693827	-5.004951127	-5.349721674	-2.61276421	-5.70994371	0.29154444	-5.125414936	-2.723234128	-5.007074099	-1.033971463
-4.783469411	-4.443113495	-4.893882604	-3.054997846	-4.817046818	-4.174779608	-5.653979588	-2.376286661	-5.388336748	-0.279134461	-5.10712365	-1.86438413	-4.627277463	-0.500959266
-4.712866563	-4.202095798	-4.755788817	-2.484770284	-5.254127328	-3.949914641	-5.557557263	-2.680058147	-5.327058331	0.767886686	-5.027239907	-2.083096331	-4.279852915	-0.903198301
-4.614841224	-3.790138138	-4.44674112	-2.839103545	-4.480042264	-4.534757997	-5.366984987	-2.060408977	-5.09478188	-0.789490987	-4.498859256	-2.399747011	-4.323652526	-0.438751045
-4.732520833	-3.984447448	-5.04385375	-2.890943658	-4.745265492	-3.581880976	-4.573157941	-2.724701933	-4.721621524	-0.253353074	-4.595007656	-1.848057149	-4.178269107	-0.538236608

-4.917748589	-3.271480777	-4.900681346	-1.827472698	-4.817941332	-4.386663698	-3.761621244	-2.100536552	-3.173961448	0.542945568	-4.829765303	-1.966042033	-3.881884352	0.233610858
-4.785788737	-3.288315657	-4.857248405	-1.660329413	-4.964632862	-4.302994052	-3.455890007	-2.304539147	-2.836658234	-0.05330356	-5.063485423	-1.769490244	-3.997332132	0.056198695
-4.898306977	-3.263759925	-4.977241375	-1.971963086	-4.837391087	-4.346101345	-3.758177952	-2.247071674	-2.665438519	-0.161483954	-5.010244453	-1.839045755	-3.981329399	0.379656827
-4.822164848	-3.29044865	-4.577909392	-2.093124154	-4.513745418	-4.43044274	-4.069674989	-2.238892142	-3.06616998	0.184304886	-4.601369905	-1.834474066	-3.978665423	0.011570755
-4.924567217	-3.72306742	-4.978555194	-2.385103991	-4.741191923	-4.712697472	-4.339405462	-2.184703444	-3.569817037	0.159295081	-4.992769523	-2.149207969	-4.103794009	0.198742564

hsa-miR-6880-3	hsa-miR-6880-5	hsa-miR-6884-3	hsa-miR-6885-3	hsa-miR-6887-3	hsa-miR-6887-5	hsa-miR-6889-3	hsa-miR-6889-5	hsa-miR-6890-3	hsa-miR-6890-5	hsa-miR-6891-3	hsa-miR-6891-5	hsa-miR-6892-3	hsa-miR-6892-5
-3.594343445	-1.080345532	-4.386748507	-4.564882825	-3.816408608	-2.571713705	-4.623657251	-1.232688175	-4.558596239	-4.411646049	-4.583134642	-1.024601231	-4.627171788	-4.635540459
-3.789957926	-1.355006975	-4.574203352	-4.855752132	-4.149917903	-2.593567089	-4.870110516	-1.397444847	-4.682370816	-4.717766411	-4.915597368	-1.335411235	-4.933299769	-4.725730291
-3.695433171	-0.816254109	-4.519067821	-4.582578521	-4.044356793	-2.852242131	-4.905613385	-1.218147656	-4.637396515	-4.545970929	-4.459219212	-1.04102425	-4.74922793	-5.279869931
-3.624176262	-1.180474738	-4.353097019	-4.704949467	-3.971884977	-2.551698823	-4.792070002	-1.121535429	-4.680080323	-4.660093238	-4.650706193	-0.919588346	-4.639063181	-5.185112962
-3.216313607	-0.771849115	-4.219085857	-4.378611453	-3.565655189	-2.425381345	-4.425266898	-0.956066919	-4.434088441	-4.312155871	-4.26603119	-0.904269782	-4.326913269	-4.940007786
-3.501424824	-1.184288108	-4.44996191	-4.696467379	-3.917683339	-2.865419119	-4.800656492	-1.206167436	-4.693783224	-4.646532102	-4.568240868	-1.191465037	-4.642564817	-4.72067154
-3.430716721	-1.040975777	-4.223697677	-4.475509546	-3.799521349	-2.487614856	-4.56939041	-0.953515626	-4.440912307	-4.520204907	-4.471792079	-1.073513306	-4.411195272	-4.952064132
-3.720014876	-1.362028736	-4.481399257	-4.684067554	-4.265557472	-2.773157675	-5.048968144	-1.346280971	-4.731384893	-4.808187496	-4.957090359	-1.454962981	-4.883151602	-5.151427161
-3.772160899	-0.956044472	-4.65776199	-4.833956367	-4.163548448	-2.993412597	-5.035460703	-1.415606005	-4.863357105	-4.852314444	-4.843413368	-1.311784844	-4.898261995	-4.997006037
-3.558163659	-1.405193065	-4.464999304	-4.723290051	-4.089238033	-2.554457991	-4.897462907	-1.360434974	-4.518479209	-4.673810625	-4.762688012	-1.376376876	-4.700397903	-4.78974993
-3.826424881	-1.11197895	-4.803737982	-4.717216886	-4.292038166	-2.650487414	-5.158198062	-1.377370589	-5.054949172	-5.020495375	-4.774050865	-1.679556206	-4.702461756	-5.253957711
-3.848305199	-1.061346819	-4.890803323	-5.236752878	-4.448138965	-2.521309183	-5.444377305	-1.385334418	-5.125196158	-4.791574443	-4.951655231	-1.282366533	-5.1816804	-4.955054354
-4.077404891	-1.372900249	-5.046950237	-5.034515167	-4.444496401	-2.922070865	-5.493069399	-1.658686485	-5.365703541	-4.929568354	-4.993868768	-1.631856877	-5.302650762	-5.358904689
-3.818261679	-1.180780027	-4.833348899	-4.754757808	-4.346848143	-2.883707451	-5.072382712	-1.520456527	-4.883391543	-5.256612123	-4.672842174	-1.47556531	-4.877862914	-5.095349247
-3.941438125	-1.222883198	-4.950447164	-5.099436512	-4.390671962	-3.038875827	-5.107465794	-1.562076531	-5.078887972	-5.137855068	-4.981026106	-1.789961836	-5.008469449	-4.967600944
-3.969358915	-1.192014138	-4.883538821	-4.941978615	-4.35243381	-3.028109713	-5.223506386	-1.415974919	-5.161719273	-5.077294454	-4.939774813	-1.620259028	-4.99103825	-5.418034324
-3.81829408	-1.122888138	-4.936298113	-4.923819521	-4.386812551	-3.100913979	-5.225901169	-1.293703216	-5.103675619	-5.262762195	-5.048946386	-1.697602463	-5.056416405	-4.788957874
-4.128346004	-1.926469053	-5.105447018	-4.973192484	-4.831758891	-2.969670092	-5.445420883	-1.889035644	-5.397704345	-5.308990212	-5.032969898	-1.825286223	-5.221347215	-5.21330871
-3.836966154	-1.62746782	-4.848499437	-4.856911725	-4.343825736	-2.911359661	-5.09616847	-1.441933712	-5.117484484	-5.240949928	-4.7415487	-1.46950011	-4.864121322	-5.077625403
-3.783522932	-1.371644433	-4.696272612	-4.892306386	-4.326372801	-2.820320835	-5.160951488	-1.280074219	-5.09427552	-5.249957618	-4.940701691	-1.486173849	-4.767133157	-5.139390019
-3.980515055	-1.237141436	-4.899514024	-4.98181879	-4.443474543	-3.072595516	-5.141807021	-1.581425172	-5.066996607	-5.09406572	-4.892829934	-1.694171755	-4.982617857	-4.783439353
-3.889378724	-1.20376805	-4.968180169	-5.069321756	-4.456776585	-3.267369646	-5.12675739	-1.615675188	-5.200578484	-5.142131385	-5.214802267	-1.9070724	-4.986949337	-5.072260857
-3.79012574	-1.167059814	-4.581129687	-4.659820004	-4.243803777	-2.674410619	-4.870560124	-1.310544878	-4.901434869	-4.906992482	-4.727311968	-1.325312526	-4.778694483	-5.462226913
-3.469381331	-1.138889705	-4.274069507	-4.494273352	-3.802529692	-2.239881905	-4.61885439	-0.91349496	-4.649770787	-4.737371934	-4.292559196	-0.769664142	-4.444132548	-4.787567532
-3.9344671	-1.655668854	-4.848942079	-4.889471319	-4.476128581	-3.023018946	-5.112869071	-1.395618398	-5.169925719	-5.023958999	-4.887643682	-1.621355328	-4.868972369	-5.320467412
-3.464801576	-1.412226283	-4.324402239	-4.590980651	-3.776080993	-2.511229991	-4.625806478	-1.23543642	-4.614659555	-5.007523799	-4.365452986	-0.755925216	-4.33385689	-4.96357959
-3.934196538	-1.311577633	-5.127025098	-5.285570078	-4.582154152	-2.983161935	-5.679393212	-1.641465939	-5.494743381	-4.989897772	-5.348410214	-1.80095652	-5.291669284	-5.300202462
-3.637385142	-1.165632206	-4.599111016	-4.756790272	-4.147724992	-2.701578152	-5.002169989	-1.457549226	-4.903497841	-4.762924841	-4.705181164	-1.277448919	-4.641453323	-4.628547476
-3.736274454	-2.180793123	-4.677504684	-4.822392752	-4.465824104	-3.214448392	-5.041794202	-1.502775942	-5.030381533	-4.860486864	-4.738261921	-1.268737492	-4.960779709	-4.691649058
-3.686190544	-1.157647027	-4.584640196	-4.835384692	-4.237283278	-2.761404597	-5.158001113	-1.210443731	-4.824079675	-4.807847297	-4.755260308	-1.20081998	-4.877758025	-5.193795715
-3.905613193	-1.308104846	-4.672785365	-4.812093213	-4.19450445	-2.481150302	-5.191778815	-1.18339728	-4.989371077	-4.772187949	-4.876090022	-1.431794504	-4.964333033	-5.173777773
-4.497052727	-2.915641137	-5.271349574	-5.573066926	-4.957973013	-2.508974028	-5.852161888	-2.240252157	-5.73833841	-5.224344333	-5.567402353	-1.806620973	-5.495508488	-4.367441492
-4.043394256	-1.320661022	-4.875470922	-5.262666629	-4.732131123	-2.875420043	-5.26826693	-1.443059248	-5.11039648	-4.701929733	-5.268782979	-2.020415818	-5.195940873	-4.983812631
-3.392154564	-1.692730965	-4.716633166	-5.081504906	-4.775514574	-2.937884432	-5.382935559	-1.108027986	-4.936011344	-4.835127139	-5.27075856	-2.197667908	-5.241859346	-5.24322881
-4.235648027	-1.340617747	-5.323855375	-5.091379317	-4.856522261	-3.623466932	-5.57164953	-1.647152198	-5.410496709	-5.527483995	-5.093078293	-2.162643229	-5.347124714	-5.533236262
-3.497017031	-0.590573893	-4.482205939	-4.697354212	-4.248868026	-2.942076926	-4.892296506	-1.147713516	-4.750379372	-5.038654182	-4.739353763	-1.774799086	-4.541955026	-4.864861342
-3.2934231	-1.841370985	-6.321010498	-6.431686623	-5.521950567	-3.362278987	-6.087736032	-1.212413014	-7.453128355	-6.089775231	-5.689928401	-3.130436559	-5.831279951	-6.167945467
-3.847554426	-1.594744858	-4.715854295	-4.713444983	-4.721903087	-3.363879106	-5.068328054	-1.54699578	-5.20102163	-5.51456094	-5.20480971	-2.34440688	-5.091956596	-5.396275475
-3.806022358	-0.809525094	-4.61151641	-4.657451872	-4.325248636	-3.517996338	-5.050535429	-1.429499293	-5.041377617	-5.557182609	-4.963794837	-1.83270346	-5.030875953	-5.831437334
-3.520839662	-1.695026289	-4.80444379	-5.472903838	-4.729463492	-3.168967963	-5.18560265	-1.365367284	-4.815129277	-5.250758804	-4.957515943	-2.574884544	-4.614690757	-4.604566677
-3.952976337	-1.02060653	-4.598569117	-4.772843964	-4.325322157	-3.41170188	-4.914953111	-1.778027924	-4.932082156	-5.272351575	-4.85564176	-2.029385989	-4.868372992	-4.946564479
-3.291223819	-1.579559443	-4.873324241	-5.050089034	-4.817945498	-3.478581521	-5.280511381	-0.896191368	-5.151293423	-5.358569497	-5.025470884	-2.356854357	-5.025951227	-5.432569594
-3.533303125	-2.138555986	-5.363025286	-5.402414532	-5.012281158	-3.653738329	-5.710564101	-1.264625664	-5.577892728	-5.66665254	-5.315956458	-2.768108721	-5.245161662	-5.206019186
-3.173577794	0.195028698	-4.166460624	-4.08898101	-3.459396592	-2.45370821	-4.10351341	-1.271668315	-4.220873777	-4.644680045	-3.819741306	-1.114213859	-4.143875305	-4.969566503
-3.553644479	-0.998209046	-5.089832641	-5.12214367	-4.73606915	-3.679272144	-5.43737801	-1.053162101	-5.24396921	-5.617737872	-5.314453293	-2.305328626	-5.389037763	-5.958259556
-4.062553775	-1.352440463	-5.001148063	-5.124795859	-4.896385393	-3.662874399	-5.510164742	-1.359072662	-5.125237176	-5.539803278	-5.254360834	-2.290523082	-5.159439886	-5.476313382
-3.803782815	-0.46530808	-4.447787968	-4.636926111	-4.210606911	-3.067272718	-4.881424652	-1.163503386	-4.89498525	-5.164317622	-4.695122947	-1.502570695	-4.781691242	-5.728177524

-4.026380525	-1.344885051	-4.789368952	-5.039381499	-4.681379866	-3.194820858	-5.526468774	-1.280566196	-5.214672597	-5.420495975	-5.079747349	-1.972722548	-5.006582107	-5.761444548
-3.19514801	-1.399050283	-5.05035137	-5.184875044	-4.592958027	-2.694239624	-5.353699773	-1.447996626	-5.204185038	-4.622593065	-4.947869544	-2.63636211	-5.356949615	-4.962104887
-3.876925749	-0.765894526	-4.504652659	-4.686024187	-4.567846412	-2.969985165	-5.037679345	-1.175179273	-4.935159984	-4.756805775	-4.794475596	-1.726759386	-4.655901072	-4.692310058
-3.26889809	0.426884252	-3.936045339	-4.134714959	-3.656293286	-1.965979671	-4.292667332	-1.231318261	-4.403612297	-4.689981643	-3.943905602	-0.937449329	-4.138260524	-5.054355227
-3.686617109	-1.660896087	-4.627796262	-4.7647431	-4.069813913	-3.216939828	-4.74611132	-1.353712841	-4.946663242	-5.19030393	-4.56035855	-1.738251783	-4.775605665	-5.168038906
-3.032342945	-1.468575391	-4.932025633	-5.130812107	-4.755389083	-1.893065582	-5.036687821	-0.875664946	-5.167972834	-4.069318351	-5.208761822	-2.452416347	-4.646934661	-4.276543058
-3.714067043	-2.011023527	-4.014852733	-5.128112012	-4.884926429	-2.094911605	-4.554054124	-1.594385339	-5.226940268	-3.974357654	-3.688665965	-2.437098193	-4.26415896	-3.984665779
-3.701476966	-1.768869719	-4.745712641	-4.885343326	-4.194646778	-3.005367318	-4.824674709	-1.784887644	-4.922859862	-5.23545291	-4.609755885	-1.977516265	-4.964393573	-4.950909715
-2.946093006	-1.350817377	-4.53139739	-5.075352184	-4.530526473	-2.9577268	-5.279063319	-0.633996263	-5.216181317	-5.706788926	-4.914483732	-2.090000041	-4.783279124	-5.448577257
-3.600552934	-1.227362429	-4.732083094	-4.986138922	-4.645213203	-3.149645405	-5.258091742	-0.904157202	-5.095984886	-5.285387052	-5.143080968	-2.005144706	-5.149238365	-5.387746662
-3.069816462	-2.474874242	-5.18800795	-5.534604841	-5.229867034	-3.561097973	-5.972063902	-1.004968893	-6.068940112	-5.104429849	-5.688446967	-2.758363599	-5.273099872	-5.267399814
-3.479168756	-1.58837487	-4.86034588	-5.155786443	-4.812186767	-3.56049206	-5.384062999	-0.975158782	-5.291227413	-5.109525287	-5.273586505	-2.361701172	-5.283845219	-5.59100097
-4.126331647	-1.618742687	-5.113327685	-5.06224355	-4.825976628	-3.564362736	-5.39884562	-1.694219309	-5.310787472	-5.466889378	-5.281731263	-2.323835253	-5.298471913	-5.077021242
-3.241053916	-2.130676133	-5.165467589	-5.554224582	-5.17284619	-2.978041973	-5.788664696	-1.139786869	-5.553973087	-5.524275348	-5.388942243	-2.765908147	-5.072582913	-5.556780702
-3.423437199	-2.454541665	-5.17177358	-6.537715492	-6.066570525	-2.375889476	-6.741457048	-1.165538866	-5.822371792	-5.369646897	-5.105757417	-2.629067821	-4.488852505	-4.745356782
-3.997878718	-2.015055118	-5.035815514	-5.954591585	-5.643988119	-2.800452945	-5.252080571	-1.487470516	-5.329664726	-4.802080515	-5.277170212	-2.280839566	-4.6278012	-4.415900338
-3.71137094	-1.344047297	-4.647000081	-4.974070516	-4.722219367	-3.298162667	-5.277596678	-1.360027346	-5.074155786	-5.637328821	-4.954776874	-2.127974843	-4.921664101	-5.727230853
-3.642474822	-0.955661137	-4.090981905	-4.213324395	-4.097201098	-2.755742136	-4.641693502	-1.511443903	-4.55373151	-4.565412904	-4.379790039	-1.727963938	-4.439906669	-4.847879688
-3.646061485	-0.541392253	-4.338926108	-4.732481468	-4.302965854	-2.758526561	-4.895683695	-1.057165009	-4.821644309	-4.943492772	-4.547605151	-1.41080779	-4.847524148	-5.36295646
-3.658136947	-1.759887034	-5.484274185	-5.480966851	-5.100582102	-3.64886875	-5.909151158	-1.072128532	-5.38579235	-5.249176068	-5.622649327	-2.375288276	-5.581991886	-5.561284952
-3.714911486	-0.575408478	-4.671508071	-4.854824982	-4.3130806	-3.291538808	-5.069475641	-1.209450529	-4.954535712	-5.217604287	-5.061754022	-1.614382271	-4.875794575	-5.012788555
-4.26428712	-1.810630456	-5.412477323	-5.42750661	-4.974746432	-2.643040136	-5.883014837	-1.74397549	-5.622495854	-4.194846332	-5.810743297	-2.085784374	-5.59358492	-3.787016388
-4.331258545	-1.259679441	-5.080645344	-5.461091853	-4.940224264	-2.954511454	-5.688882508	-1.569965583	-5.918368962	-5.425311354	-5.342873859	-1.627949662	-5.575163117	-5.596224915
-4.16126083	-1.231566462	-5.673091918	-6.998342143	-5.563634892	-2.737292762	-7.719917182	-1.158957068	-7.731443491	-5.019356701	-7.803050567	-2.222486012	-7.048159531	-5.477947461
-4.144454385	-0.859488332	-5.311176627	-5.671047027	-4.664155859	-2.927205479	-5.701768417	-1.716513083	-5.70681007	-4.699208378	-5.398141362	-1.816563694	-5.533624559	-5.012971442
-3.858417296	-1.453743085	-5.578065324	-5.675427551	-4.812487157	-2.89439846	-6.019025628	-1.847980289	-6.444619081	-5.061593499	-5.228303791	-2.323917267	-5.455771337	-3.68787511
-4.203367163	-1.341779252	-5.747048675	-5.192468233	-4.799929305	-2.268344375	-5.706313519	-2.00292068	-5.636432393	-3.656615643	-5.555642708	-2.145558562	-5.60820722	-2.840250187
-4.021689104	-0.987916471	-5.318076139	-5.425683732	-4.737558267	-2.524521032	-5.3787179	-1.747530179	-5.1644476	-3.852825841	-5.406072462	-2.000821187	-5.521188252	-3.100670278
-3.986780722	-1.403536827	-5.344903347	-5.290892723	-4.778893121	-2.593583652	-5.645692626	-1.617808018	-5.293393337	-4.064128444	-5.264975474	-1.871122323	-5.24188247	-3.506839521
-3.80986116	-1.672933548	-4.890992026	-4.989222039	-4.607496238	-2.675482184	-5.74322178	-1.604955129	-5.451374936	-4.909965252	-5.155992379	-1.889273552	-5.018202178	-3.512313004
-3.850184177	-1.502698915	-5.428787412	-5.380094433	-5.071182414	-2.061250708	-5.889864749	-1.782518311	-5.606499204	-3.674569318	-5.241101868	-2.258999175	-5.430203992	-2.906907731
-3.923437084	-1.799051637	-5.38337834	-5.572438059	-5.104774944	-2.183399261	-5.559411495	-2.023003434	-5.45306251	-4.426939435	-5.174774938	-2.168788357	-5.020207976	-3.049865346
-4.126110828	-1.821138516	-5.464103719	-5.572098941	-4.922647338	-2.295308375	-5.675424771	-1.50356945	-5.522888091	-3.499739591	-5.504281443	-2.026621924	-5.286435759	-3.381840571
-4.240482831	-1.272421284	-5.157189493	-5.407748655	-4.87159919	-2.148090722	-5.654790228	-1.934864648	-5.279755619	-3.569156283	-5.512600104	-1.972312788	-5.175655492	-2.902839219
-4.082548392	-1.683278375	-5.014516933	-4.963723176	-4.871154654	-3.362279537	-5.745908021	-1.448454495	-5.448906622	-5.330927913	-5.367191321	-2.28011423	-5.380125822	-5.373902814
-3.542476733	-1.341123888	-4.538037045	-4.683129859	-4.575784139	-3.101304321	-5.171880652	-1.091805333	-4.848278121	-5.349674547	-4.658721646	-1.720797219	-4.7859543	-5.319243044
-2.868673175	-1.941018659	-4.06429409	-5.157544759	-4.75249653	-2.935061764	-5.29888499	-0.717363507	-5.109936396	-4.430342037	-4.944033394	-2.436319383	-4.672393775	-4.797804776
-3.457673746	-1.929635393	-5.239618685	-5.400715081	-4.77471769	-3.010283577	-5.12803242	-1.21676516	-5.324428873	-5.683152746	-5.145553125	-2.211311214	-5.513659902	-5.320327936
-3.796209335	-1.323165636	-4.857539599	-4.958886273	-4.609973381	-3.471539605	-5.318691638	-1.406981117	-4.888153018	-5.453101709	-5.183730094	-2.548919926	-5.190627664	-5.565019546
-3.731149215	-2.084714404	-5.068290074	-5.221907922	-4.520462701	-2.900723453	-5.00642994	-2.056033759	-5.123013198	-5.150977231	-4.749158004	-2.211845923	-4.691115724	-4.932281004
-3.352889958	-1.537806487	-4.784083948	-5.156987208	-4.969840842	-3.401949903	-5.429830368	-1.03210336	-5.233986842	-5.281744069	-5.149779099	-2.454371083	-5.128565229	-5.666798084
-3.684332021	-1.693824023	-4.701559463	-5.134715143	-4.770356325	-3.248329945	-5.623839276	-1.062617409	-5.237427687	-5.393164073	-5.323558349	-2.208016931	-5.2344549	-5.746366356
-3.82404982	-1.694418406	-5.253849552	-5.491663008	-4.962499925	-3.558312978	-5.944216522	-1.078271209	-5.646165625	-5.205910934	-5.683862121	-2.541657254	-5.486464898	-5.744266383
-4.034422315	-3.093741736	-5.375833671	-5.387969409	-4.861610041	-3.306913806	-5.573739569	-1.549817235	-5.472096399	-5.341840363	-5.387011602	-2.392276188	-4.974955723	-4.620678137
-4.326862642	-2.472044929	-5.462851343	-5.468889987	-5.336859397	-3.136385437	-5.638975911	-1.54929211	-5.465499486	-5.24944703	-5.678303628	-2.449795047	-5.306007886	-4.895234267
-3.639587004	-2.32194967	-5.117714275	-5.607259489	-5.257955602	-3.183970316	-6.000956307	-1.3023368	-5.481960663	-5.549450704	-5.435120223	-2.475207926	-5.503578147	-5.685571555
-3.61691861	-1.929944962	-4.770515624	-4.727870111	-5.293381261	-2.538415177	-5.701815035	-1.61098991	-5.137882173	-5.523083013	-5.233135834	-2.663604517	-4.603108444	-4.719581845
-3.841470852	-1.591889143	-4.688687428	-4.952154053	-4.912202379	-3.344070933	-5.533141442	-1.453778887	-5.184839162	-5.510096873	-4.943001608	-2.527916521	-4.865015849	-5.544677483
-2.966221753	-1.53243478	-5.219730657	-5.408748829	-5.02816751	-3.063498755	-5.376053475	-0.719308743	-5.302590527	-4.902099154	-5.715142634	-2.869477134	-5.414068768	-5.024638644

-3.991223187	-1.244777702	-4.952525373	-5.175751332	-4.548373904	-2.064366178	-5.446902475	-1.573704158	-5.00564217	-3.288743256	-5.24258336	-1.724229396	-5.345058403	-2.967666884
-3.708631018	-1.521813791	-5.207323362	-5.285909738	-4.373545661	-2.044206773	-5.620592636	-1.732793258	-5.199794197	-3.304388439	-5.296426502	-1.954972207	-5.47236699	-2.843881745
-3.528081468	-1.582596579	-5.068522298	-5.356185191	-4.485391377	-2.197854013	-5.422565746	-1.410728188	-5.219775144	-3.566810068	-5.212076145	-1.964276947	-5.138990402	-3.002484961
-3.78321569	-1.662576562	-5.123942673	-5.198657456	-4.552655421	-2.353835086	-5.593854731	-1.742128792	-5.317373592	-3.85525622	-5.276939309	-1.984921409	-5.202914701	-3.218634942
-4.090259178	-1.456081803	-5.500840571	-5.53164648	-4.74975172	-2.626211031	-5.691343362	-1.673075332	-5.395730282	-4.162048507	-5.458456664	-1.908015269	-5.483159267	-3.922385482

hsa-miR-6893-5	hsa-miR-6894-3	hsa-miR-6894-5	hsa-miR-6895-5	hsa-miR-7106-3	hsa-miR-7106-5	hsa-miR-7107-5	hsa-miR-7108-3	hsa-miR-7108-5	hsa-miR-7109-3	hsa-miR-7109-5	hsa-miR-711	hsa-miR-7110-5	hsa-miR-7111-3
-2.779893477	-3.779781117	-3.61133808	-4.949434663	-4.33794403	-2.062116728	-0.970174421	-4.480979214	-0.795170779	-3.633153968	-0.687557448	-2.118746701	-1.620605481	-3.965068481
-2.427827572	-3.969040103	-3.813327246	-4.806182841	-4.450507334	-2.517814273	-1.131183018	-4.362754332	0.194929615	-3.856090214	-0.987150862	-1.182536889	-1.557583151	-4.158905181
-2.235629011	-3.884395531	-3.815395597	-4.419542878	-4.488085261	-2.451967068	-0.977658938	-4.394358788	0.151169583	-3.740024358	-0.647107074	-1.742084871	-0.546413347	-4.105635209
-1.910510915	-3.919989984	-3.686584614	-4.144580373	-4.325947507	-2.251472867	-1.049059938	-4.289690876	0.079305054	-3.832465445	-0.44287612	-1.573293011	-0.668844638	-4.100878787
-2.415651946	-3.533145868	-3.565949137	-4.742396955	-4.197579744	-1.8718312	-0.808225926	-4.16398564	-0.373767107	-3.480210039	-0.371155482	-1.668788499	-0.938352206	-3.873382961
-2.106248402	-4.306715427	-4.004546335	-4.52979299	-4.398881374	-2.275884519	-1.12617837	-4.14351006	0.12388729	-3.729036408	-0.775935721	-1.561323906	-1.371378892	-4.238855806
-1.831458542	-3.628719499	-3.556600599	-3.733556506	-4.200285759	-2.063274442	-0.794988038	-4.076143121	0.10682888	-3.749859826	-0.504415722	-1.542870551	-1.741606412	-3.952430827
-2.203771164	-3.985455714	-3.756634187	-4.183920227	-4.231598899	-2.524772091	-1.14205441	-4.288449176	0.342256337	-3.751377413	-0.852044478	-1.307430456	-1.32443168	-4.197389412
-2.460318081	-4.021096592	-3.934518616	-5.012288939	-4.458663867	-2.484516966	-1.077180181	-4.479991066	0.288609575	-3.960933219	-0.911793384	-1.723205825	-1.577422056	-4.349013176
-2.345540074	-3.725922484	-3.886349339	-4.743336294	-4.170156104	-2.242238576	-0.978958699	-4.222034138	0.106262748	-3.763743358	-0.658739603	-1.356249984	-2.156381555	-4.295016598
-2.165938605	-4.353660133	-3.754504978	-4.10524591	-4.649357581	-2.316993369	-1.345750431	-4.352618691	0.076505316	-4.097550595	-0.396379634	-1.663932961	-1.759986569	-4.347439278
-2.394069454	-4.401868334	-3.819191528	-5.261826536	-5.017073846	-2.529245844	-0.953280313	-4.606147675	-0.153523168	-4.366412295	-0.79101072	-1.629620845	-1.601385244	-4.421793209
-2.536219762	-4.72238772	-3.861277627	-5.201777325	-5.234985679	-2.322466684	-1.252685789	-4.637392338	-0.054657852	-4.594290011	-0.893510456	-1.557280618	-1.716758043	-4.447241524
-2.203741836	-4.460841465	-3.981518236	-4.502355416	-4.692176102	-2.382279872	-1.24035432	-4.424710888	-0.282629512	-4.120654478	-0.610561027	-1.834229735	-1.459517771	-4.416491219
-2.393483883	-4.651250062	-4.160050583	-4.744925215	-4.90317755	-2.357390876	-1.295899113	-4.334739202	-0.250199626	-4.347349202	-1.105698552	-1.510319129	-2.021852938	-4.594823414
-2.480417296	-4.585190912	-3.88158929	-4.752131012	-4.71825808	-2.467317299	-1.228109583	-4.451916884	0.365507942	-4.213914571	-0.987119325	-1.469883788	-1.163128024	-4.628981016
-2.177092365	-4.519697639	-4.12264712	-4.25045414	-4.80016242	-2.815276573	-1.048578443	-4.184487642	0.026521676	-4.406994497	-1.438968016	-1.562943487	-1.81885285	-4.653548434
-2.548256628	-4.504355568	-3.969741108	-4.669178997	-5.185572212	-2.960831724	-1.453089178	-4.357444833	0.380812845	-4.401633534	-0.999336446	-1.753438415	-1.800748525	-4.414500151
-2.27921501	-4.631906189	-3.820154088	-4.706924122	-4.891732264	-2.216721917	-1.306241	-4.442222569	-0.334038137	-4.227639487	-0.619551886	-1.719209979	-1.666935497	-4.367091693
-2.072640578	-4.481565045	-4.091929953	-4.003189102	-4.484803392	-2.406923604	-1.003230588	-4.283973437	0.062156814	-4.285658196	-0.757697629	-1.428664339	-1.958798136	-4.689444184
-2.406848305	-4.622604489	-4.116040409	-4.459087902	-4.723042965	-2.587823974	-1.135240545	-4.329062124	-0.162415751	-4.366686207	-1.121100471	-1.528619539	-1.867418745	-4.686333949
-2.176107474	-4.802444859	-4.199392462	-4.327803918	-4.773321491	-2.828269566	-1.221660995	-4.362191906	0.080544901	-4.387647058	-1.395465591	-1.421034745	-2.175966317	-4.677778058
-2.307391139	-4.477858452	-3.844798718	-4.584895049	-4.539625978	-2.246267757	-1.113915136	-4.495469712	0.248450043	-4.156732575	-0.571896473	-1.489442297	-1.367212465	-4.310434661
-2.180250456	-4.118894905	-3.511581468	-4.835217383	-4.463615653	-1.781402174	-0.551012054	-4.022255844	-0.151340286	-4.030679958	-0.385774229	-1.542665229	-1.160732168	-3.910536099
-2.20913428	-4.738808411	-3.95714654	-4.708060805	-5.044904716	-2.817719213	-1.63997912	-4.264425215	0.110416595	-4.142769069	-1.240996317	-1.554217017	-1.195321542	-4.626248585
-2.286116906	-4.208558248	-3.965681944	-4.916347323	-4.505063851	-2.072027907	-0.777762237	-3.952414215	-0.162859444	-4.054905521	-0.812314696	-1.97571454	-1.345815596	-4.187622774
-2.250163488	-4.83387684	-4.08094372	-5.142820585	-4.83645207	-2.434020992	-1.405737852	-4.564146207	-0.114807323	-4.482802828	-1.033800343	-1.650104349	-1.756161517	-4.55495469
-1.996353666	-4.43287573	-3.736347819	-4.604843516	-4.81701149	-2.199321962	-1.121729702	-4.150104456	-0.643660719	-4.330054523	-0.787183179	-1.660495513	-1.800976684	-4.061235592
-2.500679869	-4.306804607	-4.311824566	-5.585179106	-4.845903975	-2.554094502	-0.986546388	-4.454832844	-0.118658479	-3.908258267	-0.783820972	-2.068803562	-1.66279295	-4.47443093
-2.059965635	-3.991545794	-3.762022579	-4.81600982	-4.312338322	-2.367819563	-1.037072168	-4.416594768	0.113427415	-3.996343469	-0.52541523	-1.635429771	-1.624288904	-4.022941514
-2.082085291	-4.220989231	-3.750514993	-3.886698021	-4.485991068	-2.092278741	-1.092120686	-4.587324132	0.233946578	-4.22293209	-0.523155895	-1.420249315	-2.000150955	-4.26148061
-2.777566586	-4.996895815	-4.129724885	-5.113322777	-5.790666038	-2.489343399	-1.92442737	-5.06502015	-0.633930217	-5.115336528	-1.404387048	-2.164351703	-2.47218889	-4.582260056
-2.498051822	-4.436420362	-4.179828991	-5.057883635	-4.71231122	-3.259667399	-1.566193818	-4.126239384	0.33916358	-4.27958186	-1.759294817	-1.588118174	-1.492169274	-4.639946343
-2.389699661	-4.417450117	-4.122192143	-4.366509438	-4.459827989	-3.292831532	-1.300951839	-4.041823696	0.746385154	-4.288640284	-1.557060335	-1.592061081	-1.879567004	-4.992325007
-3.028292845	-5.047371227	-4.476004074	-6.096424626	-4.983349413	-3.47109201	-1.424312148	-4.470362477	-0.101959998	-4.567719822	-1.909938915	-1.694135664	-1.574327463	-4.925013658
-2.779383786	-4.434378161	-4.281465546	-4.89146481	-4.238256892	-3.381912483	-0.815039965	-3.846203868	0.408312547	-4.26523179	-1.34957002	-1.786608332	-0.939774806	-4.47041838
-3.022388912	-6.05807266	-5.757198927	-7.439784816	-5.965768932	-4.375149559	-1.598335358	-3.4648629	0.399769573	-5.392404574	-2.407242301	-2.303113482	-1.99821751	-5.918653343
-2.915709405	-4.47600732	-4.168238109	-5.366295497	-4.787859869	-3.93519776	-1.69722106	-3.66791844	0.418624322	-4.225942674	-1.988328634	-1.575137344	-1.336572828	-4.553950657
-3.223634643	-4.298575746	-4.194737571	-5.165567928	-4.515687833	-3.331905754	-1.38932995	-4.161033622	0.196870309	-4.043722387	-1.692613779	-1.413649993	-0.606915147	-4.442939686
-3.080397871	-4.588798259	-4.438157796	-5.365871671	-4.737155633	-4.063240612	-1.609165223	-3.628141411	-0.09862442	-4.420958497	-2.288223826	-2.201117538	-1.923664845	-4.705461248
-3.042462952	-4.487715711	-4.303680726	-5.696515012	-4.755861443	-3.44872202	-1.485040881	-4.129007501	-0.269928988	-4.379726377	-1.822411854	-1.670411027	-2.211776603	-4.424194322
-2.332154185	-4.547869827	-4.048914987	-4.362420357	-4.885811943	-3.548156164	-1.620207953	-3.671185729	0.439380842	-4.268079745	-2.037486314	-1.757566277	-1.216734048	-4.713292233
-2.491402533	-4.802797492	-4.060902322	-4.551256777	-5.530131339	-4.365147886	-1.811826002	-4.102616763	0.478344966	-4.816922471	-2.513230834	-1.632745065	-1.360324047	-5.124464852
-2.992554809	-4.126103987	-3.485439379	-5.615588402	-4.310784104	-1.945509838	-0.827770001	-3.908693118	-0.864743747	-3.88138592	-0.492171421	-2.321964636	-1.261695029	-3.830018733
-2.834893692	-4.99126621	-4.388308012	-5.055927687	-4.939859998	-3.725539042	-1.602209481	-3.976058928	0.436367337	-4.420619435	-1.920906723	-1.641126941	-1.003642236	-5.006094403
-2.936785021	-4.761572445	-4.462303699	-5.819344529	-4.703356246	-3.745117661	-1.469577187	-4.158005916	0.532378952	-4.464263945	-1.916813239	-1.507654971	-1.225063529	-4.748367331
-3.284249708	-4.271262643	-4.171884465	-5.887153821	-4.196509477	-2.799078625	-0.952092982	-4.161897058	0.469104354	-4.008935679	-1.230683435	-1.511994552	-0.2291173	-4.305694388

-2.763434936	-4.58044359	-4.170922247	-5.059222777	-4.565868908	-3.165817007	-1.48248779	-4.097690271	0.492276762	-4.346831395	-1.593460515	-1.269551012	-1.5181658	-4.358641258
-2.517035423	-4.717724853	-4.187124797	-4.413811387	-4.708975647	-3.849579288	-1.750148226	-3.425762006	0.708033517	-4.170180096	-2.27558593	-1.605558188	-1.33952802	-5.072230749
-2.469644747	-4.535808374	-4.035758828	-5.512561759	-4.355670522	-3.341849879	-1.205974867	-3.725478753	0.255893508	-4.122155787	-1.744880843	-1.441368622	-0.925846153	-4.731074381
-2.41892216	-3.863028095	-3.154084878	-4.74354916	-4.222645114	-2.162483858	-0.756314351	-3.94797771	-0.419145529	-3.932745984	-0.493233652	-1.788473693	-0.541724101	-3.660628588
-3.104294287	-4.539391659	-4.247296652	-5.31957877	-4.639627529	-2.76242463	-1.336416762	-4.302861041	-0.350280066	-4.199896692	-1.423269454	-2.007146673	-0.981935701	-4.476998435
-2.268933439	-5.026856914	-3.879026486	-4.23926298	-4.39403289	-4.351137944	-1.699551339	-3.518812233	0.09153798	-4.223954838	-2.047909034	-1.55932298	-1.724477322	-4.524468414
-2.027966297	-6.113368558	-4.284035147	-4.732833381	-4.015115281	-4.486758735	-1.58020808	-2.719607962	0.344319627	-4.384536715	-2.252637504	-1.551066591	-1.664642943	-4.187052936
-3.413234486	-4.688149709	-4.322126697	-5.581066988	-4.892004891	-2.585750383	-1.469939018	-4.092516782	0.287893958	-4.251489576	-1.155059806	-2.205238849	-1.474082419	-4.571591247
-2.481173149	-4.777020053	-4.106298816	-4.366733528	-4.32612861	-3.370746943	-1.407269127	-3.084779207	0.796094121	-4.366091434	-1.556718624	-1.703571685	-1.360128422	-4.592264007
-2.44677108	-4.585813909	-4.011145908	-4.230722585	-4.639425682	-3.200959006	-1.202677907	-3.935597926	0.766435954	-4.327606007	-1.484636176	-1.557659413	-0.561748706	-4.674251824
-2.692021263	-5.496513395	-4.357268411	-3.970590127	-5.610690093	-3.955834576	-1.962388247	-3.985242771	0.300105444	-4.992170579	-2.276181917	-1.294774823	-1.54069888	-4.840044423
-2.644558875	-4.80250671	-4.336149975	-4.42979919	-4.710936938	-3.577132427	-1.573701965	-4.078696156	0.477148118	-4.504676807	-1.919734926	-1.67407981	-0.810139703	-4.818100567
-3.26515794	-4.477474318	-4.428642533	-5.925592879	-5.063673173	-3.730648925	-1.628235339	-4.652281525	-0.55858283	-4.412187142	-1.906924752	-2.079273205	-1.417178653	-4.666070475
-2.481156041	-4.629968815	-4.18279906	-4.406567944	-5.148298213	-4.107293913	-1.86347374	-3.702606932	0.218978788	-4.58388062	-2.175414524	-2.420657945	-1.040683766	-4.586295029
-2.076054475	-5.975251601	-4.708851347	-4.35665444	-5.13872387	-4.459733851	-2.075887152	-3.197923509	0.105133217	-5.189915146	-2.464026667	-1.874824186	-1.888354042	-4.651305732
-2.594135427	-5.288951439	-4.299297254	-5.395089872	-5.197968219	-3.958337729	-1.840582772	-3.546052756	0.471580327	-4.684155038	-2.084524683	-1.73330604	-1.69265208	-4.599999583
-3.148963579	-4.944638728	-4.789180936	-5.703913552	-4.721960356	-3.578358704	-1.314481777	-3.646643874	0.458488981	-4.449721142	-1.87995677	-1.352192335	-1.37967011	-4.738332572
-3.069683074	-3.803231729	-3.760837581	-5.587492604	-4.379315888	-2.804890775	-1.267928609	-4.016677449	-0.36339675	-3.723963819	-1.124796195	-2.456268433	-1.178132688	-4.321090567
-2.962730306	-4.439815088	-4.101984243	-5.184685652	-4.501866522	-2.690724283	-0.69869339	-4.100431489	0.894530975	-4.234449758	-0.708487255	-1.32687413	0.316343869	-4.520624154
-2.405078165	-5.251463674	-4.241089655	-4.401643347	-4.936210932	-3.526850306	-1.478071575	-3.921393097	0.463852085	-4.829533753	-1.828409245	-1.011989543	-1.741479856	-5.15276147
-2.71328826	-4.384564245	-4.072660134	-4.934871299	-4.347438637	-3.131677419	-0.950014251	-4.452052431	0.067284827	-4.129122447	-1.410633699	-1.460786413	-1.150766169	-4.446361098
-1.554701173	-4.87720778	-4.060604017	-3.837875407	-5.116471921	-2.708357593	-1.199363972	-4.887185058	-0.222852894	-4.747000543	-1.396135238	-1.75025473	-2.652877027	-5.007530263
-2.494863301	-5.089014029	-4.186050599	-5.184776918	-5.300825966	-1.941220959	-1.138502633	-5.196668241	-0.137367374	-5.084279382	-1.033939492	-1.242812089	-2.142824122	-4.786525691
-2.498259512	-5.784069648	-4.764075932	-6.121609626	-5.646810102	-3.319974964	-1.025449099	-4.643265767	0.313616993	-5.462481434	-1.681620785	-1.265282499	-1.889715161	-5.543971485
-2.779449926	-5.077086743	-4.270832122	-5.447993401	-5.149932344	-2.708732206	-1.37587891	-4.98886007	-0.349003365	-4.600659795	-1.202915415	-1.272638029	-2.094286187	-4.855650007
-1.501166822	-5.461265205	-4.061680487	-4.023331338	-4.98090237	-3.335127027	-1.578558825	-3.962245801	0.029431571	-4.697292347	-1.696899053	-1.44732753	-2.25455202	-5.08275618
-1.401952035	-4.921535529	-3.528060991	-4.209638489	-5.003314036	-3.021886204	-1.087124027	-4.872387154	-0.357740179	-4.366778327	-1.962191996	-1.851980357	-2.349792609	-4.907388741
-1.630440996	-4.749384825	-3.918697906	-5.054584172	-4.993011913	-2.897270784	-0.70293138	-4.81454085	-0.583143453	-4.647024787	-1.623052633	-2.006051648	-2.718017825	-5.062163035
-1.203894098	-4.686451534	-3.826709112	-3.769483969	-4.965796878	-2.724725985	-1.122960841	-4.75332531	-0.312077033	-4.627771053	-1.355774614	-1.766047143	-2.052673745	-4.829467108
-1.415083755	-4.745596066	-3.872929618	-3.70673883	-4.592533588	-2.793677979	-1.470078092	-4.049835516	0.244743483	-4.583838809	-0.948148326	-1.832135376	-1.879254609	-4.813452107
-1.22333633	-5.142780862	-3.443267139	-3.777030184	-5.336379773	-3.156757523	-1.170247532	-4.332444324	-0.050762888	-4.764425513	-1.899037547	-1.632791408	-2.244076145	-5.022649845
-1.25150239	-4.778236687	-3.814295855	-4.13901314	-5.29865225	-3.2648084	-1.516887329	-4.256652269	-0.329914211	-4.592319458	-1.664828239	-1.800229397	-2.837290501	-4.707122151
-1.431110969	-4.877997192	-3.727052259	-3.645311965	-5.191057005	-2.642416023	-1.136579731	-4.620905381	-0.207126134	-4.647594023	-1.439213695	-1.832611223	-2.227619965	-5.008763226
-0.764159433	-4.711443697	-3.555273719	-3.71233495	-5.108386027	-2.765311233	-1.012588682	-4.616258517	-0.305922456	-4.477299526	-1.62483777	-1.866192777	-2.530712234	-4.941230001
-2.920100308	-4.344090819	-4.295266482	-4.947150225	-4.789079489	-3.610447262	-2.015909067	-4.449405943	0.045613851	-4.388597588	-2.037109505	-1.78279598	-1.180665099	-4.771289875
0.197887884	-4.313587891	-4.024030388	-4.632503856	-4.558615906	-3.251861083	-1.187396583	-3.794798495	0.698875431	-4.056258941	-1.58848391	-1.697609328	-0.832930821	-4.193232869
-2.133000131	-4.448026647	-3.988739004	-4.300338685	-4.844086367	-4.124243765	-1.63682632	-3.246086466	0.564653842	-4.27350294	-1.904117227	-1.171282293	-1.165985682	-4.432108421
-2.858581609	-4.762456948	-4.337793647	-5.236406567	-4.891258707	-3.692533195	-1.906050455	-3.451715952	0.398472673	-4.511206407	-1.933572486	-1.585892076	-1.176745096	-4.613013269
-3.122350035	-4.67427735	-4.588054389	-5.296483827	-4.580215676	-3.872909148	-1.775192987	-3.609010163	0.442762396	-4.352790056	-2.341175041	-2.001172339	-2.515257215	-4.908735149
-3.26766171	-4.856096275	-4.558406433	-5.876108997	-4.714828058	-3.15844105	-1.527371762	-3.744699913	0.719452637	-4.386893484	-2.030312875	-2.007352768	-2.082843813	-4.525318405
-2.701706039	-4.805369822	-4.390137746	-4.989900284	-4.560980306	-3.466236792	-1.761191249	-3.759225134	0.564180186	-4.455243795	-1.941364013	-1.566587004	-1.271627957	-4.868338323
-2.468829684	-4.701625505	-4.151911402	-4.548787239	-4.68738242	-3.26599602	-1.395989525	-3.987418122	0.509872584	-4.521973641	-1.388863105	-1.362160921	-1.193050017	-5.070316163
-2.094703372	-5.100195159	-4.008789713	-4.133508062	-5.586505915	-4.023056021	-1.598614746	-4.254556444	0.437022784	-4.552632887	-1.879636432	-2.286322829	-0.461191141	-4.666409599
-3.347778588	-5.192427262	-4.645557745	-5.42202015	-5.778258248	-3.549622077	-2.038149998	-4.28178417	-0.09805901	-4.7113015	-2.189897586	-2.630587925	-1.603039809	-4.917177683
-2.831035843	-5.05255327	-4.714166591	-5.65171688	-5.614351589	-3.856099935	-1.761184491	-4.665824116	-0.41796165	-5.059843412	-2.108328416	-1.974650854	-2.311733856	-4.909636993
-2.760946574	-5.079368923	-4.827224684	-4.766263931	-5.122178924	-4.140846292	-1.806210583	-3.606451324	0.605696585	-4.686721097	-2.042065807	-1.069270163	-1.54738076	-4.69919998
-2.92788558	-5.593130113	-4.50793919	-5.876506116	-5.197485693	-3.887677312	-1.615454536	-3.222198469	0.505651059	-4.61593247	-1.873307681	-1.986058019	-1.99972155	-5.489563164
-2.368670807	-4.560368875	-4.023137064	-4.347607104	-5.022042574	-3.718347939	-1.543156354	-4.069812331	0.611790462	-4.409218176	-1.938255698	-1.897853765	-0.468426322	-4.492063677
-2.674191169	-4.888219056	-4.198484048	-4.43424031	-4.574748701	-3.873828424	-1.68033311	-3.651504144	0.503231171	-4.522254061	-1.990297088	-1.465576299	-1.820162259	-5.182967586

-1.035900635	-4.747416866	-3.471351604	-3.517718405	-4.991555286	-2.813079302	-0.972793737	-4.460871686	-0.051139492	-4.334540184	-1.604962237	-1.609814867	-1.946451889	-4.902238312
-1.101183952	-4.656326697	-3.297167036	-3.66264917	-4.781116751	-2.726014796	-1.329531876	-4.25172214	-0.206494208	-4.456311191	-1.715259116	-1.580726875	-2.09127126	-4.842734292
-0.843839025	-4.691201203	-3.598886426	-3.257383514	-5.003691932	-2.807372187	-1.146730995	-4.169727159	0.161022448	-4.479070106	-1.627921732	-1.626721433	-2.43607079	-4.88171849
-1.153418543	-4.621515463	-3.662333097	-3.895951842	-4.920175745	-2.824129711	-1.04586967	-4.489946497	0.028283788	-4.569808299	-1.492488718	-1.612010185	-2.3597954	-4.708534386
-1.331211246	-4.826000503	-3.939854335	-3.768035022	-4.911341414	-2.726129595	-1.342073679	-4.977392557	-0.233722455	-4.62320146	-1.362955854	-1.743355991	-2.148933969	-5.098757943

hsa-miR-7111-5	hsa-miR-7112-5	hsa-miR-7113-3	hsa-miR-7114-3	hsa-miR-7114-5	hsa-miR-7150	hsa-miR-7155-5	hsa-miR-7160-5	hsa-miR-718	hsa-miR-744-5p	hsa-miR-760	hsa-miR-766-3p	hsa-miR-7845-5	hsa-miR-7846-3
-0.726501808	-4.742593638	-3.375649546	-4.593549195	-1.78401065	-0.593120783	-3.474605703	-4.341997779	-2.99595529	-3.318850124	-0.818463385	-3.73692089	-1.893130793	-3.774265518
-1.035734249	-4.073875985	-3.493008994	-4.924314969	-1.073419243	-0.824568022	-3.563217923	-4.156715682	-2.713380416	-2.576092898	-0.830488579	-3.89582232	-2.009461769	-3.302861059
-0.810183683	-4.333861964	-3.461009668	-4.834844352	-0.740510974	-0.527753146	-3.663569538	-4.08420708	-2.885067065	-2.626289584	-0.278079334	-3.707167203	-1.998327564	-3.560421729
-0.789927903	-4.362204208	-3.41553528	-4.697809337	-0.329943892	-0.481634462	-3.684503434	-4.176754606	-3.036464999	-2.408578711	-0.617436128	-3.919845467	-2.065642426	-3.208001121
-0.616238835	-4.650902049	-3.119947514	-4.398846044	-0.716976559	-0.290310547	-3.35901567	-3.894246966	-2.77588182	-2.820661682	-0.460733354	-3.649370132	-1.895770295	-3.49808549
-0.918807094	-4.245298553	-3.277950541	-4.648278079	-0.664527286	-0.669896658	-3.605310851	-4.16238443	-2.628593486	-2.222657738	-0.771241902	-3.748024319	-1.98979421	-3.700227871
-0.621023572	-4.347904802	-3.160598376	-4.47317435	-0.635200462	-0.361119094	-3.546355888	-4.168969853	-2.709049064	-2.644339492	-0.431178506	-3.659177104	-1.813891961	-3.371667536
-1.058496271	-3.96576194	-3.388108054	-4.934909684	-0.218748159	-0.762440387	-3.694619802	-3.95164307	-2.718431047	-2.138487742	-0.584385942	-3.698671096	-2.206426603	-3.384940905
-1.014654415	-4.334411624	-3.477112142	-4.889557272	-0.589817005	-0.700388096	-3.811577517	-4.245377895	-2.8472731	-2.788285266	-0.595314004	-3.951458883	-2.113388044	-3.700162149
-0.884009163	-4.026335661	-3.367617872	-4.762346642	-0.67144901	-0.666192112	-3.46488037	-4.143523881	-2.491807457	-2.567432362	-0.598850435	-3.904691773	-2.282671341	-3.317654379
-0.981944736	-3.921974751	-3.665682035	-5.024382509	0.583063902	-0.849656412	-3.740455521	-4.25989386	-2.885426386	-2.497572504	-0.5081365	-4.231310696	-2.362003893	-3.443432124
-0.927209205	-4.442577295	-3.876351939	-5.025849668	-1.290798162	-0.892878101	-3.702470522	-4.547958994	-2.902227954	-3.117161985	-0.674701255	-4.455051478	-2.256707504	-3.16701089
-1.020135864	-4.56122303	-3.894464721	-5.304426038	-0.483547908	-1.052911776	-3.953089825	-4.711992551	-3.094934278	-2.941298914	-0.785281738	-4.382779976	-2.247433335	-3.399413774
-1.04456703	-4.405508989	-3.759819583	-4.93275591	-0.439083656	-0.851936171	-3.852496519	-4.488337648	-2.789034994	-2.385927671	-0.602868598	-4.434906647	-2.485033913	-3.479546999
-1.282361109	-4.303711944	-3.739996333	-5.285562103	-0.924333629	-1.24038446	-3.693538431	-4.401591606	-2.552601211	-2.544385164	-0.790854638	-4.273803526	-2.536871988	-3.598068693
-1.082537801	-4.071937677	-3.830003482	-5.093836126	-0.512166875	-1.120332871	-3.798640584	-4.36071348	-2.780376232	-2.700674164	-0.374120966	-4.342562235	-2.261132096	-3.36294875
-1.241948579	-4.234933115	-3.82071797	-4.987974145	-1.581794433	-1.154351835	-4.015599443	-4.620666528	-2.406620946	-2.339261269	-0.519600631	-4.3763938	-2.361785391	-3.647751077
-1.053895937	-4.269569699	-4.024306994	-5.276783009	-0.921318399	-1.294034633	-3.888295852	-4.778524365	-2.930141687	-2.602021952	-0.711153891	-4.375985498	-2.304863493	-3.55363687
-0.851022589	-4.193802112	-3.69766682	-4.86410848	-0.597899057	-0.918562552	-3.573310194	-4.608510994	-2.940337765	-2.607226969	-0.910081066	-4.333609058	-2.376908885	-3.465425387
-0.972562656	-4.170678606	-3.811931604	-4.89235433	-0.695235233	-0.819962095	-3.783720474	-4.356878083	-2.687304912	-2.279284719	-0.609679811	-4.313876562	-2.417521552	-3.442811944
-1.160605349	-4.400960523	-3.782832793	-4.970660392	-0.978571448	-1.124161453	-3.660686143	-4.622392325	-2.571352948	-2.545516484	-0.613374346	-4.290195921	-2.330211347	-3.678558277
-1.28753361	-4.067647434	-3.723497645	-5.031766716	-1.306316587	-1.39571054	-3.604937095	-4.407339356	-2.362603343	-2.715241291	-0.685975377	-4.297328684	-2.330621138	-3.608561455
-0.756749481	-4.168108944	-3.730156792	-4.928639009	-0.273672126	-0.525064435	-3.691994848	-4.479415287	-3.040008191	-2.350811266	-0.617372275	-4.279060419	-2.12577406	-3.427646647
-0.42299752	-4.15678812	-3.474533428	-4.504877582	-0.562518527	-0.155691008	-3.219136477	-4.243630334	-2.873117943	-2.66593513	-0.173573129	-4.141404438	-2.113822878	-3.158093185
-1.330642538	-3.947259169	-3.718105019	-5.041155514	-0.963276642	-1.258813655	-3.765871499	-4.301966102	-2.61913444	-2.279519371	-0.617618975	-4.281319421	-2.404063465	-3.495357628
-0.636790035	-4.308393747	-3.410241887	-4.394109859	-1.222749389	-0.254891504	-3.477307618	-4.712847189	-2.900081177	-2.712658748	-0.195171294	-3.982702527	-2.212683865	-3.580201714
-1.293249015	-4.359947167	-3.848112732	-5.417933903	-0.564142148	-1.159872006	-3.840762432	-4.606222882	-2.827934432	-2.854909346	-0.74591246	-4.281526678	-2.429528149	-3.331769619
-0.809572364	-4.419211864	-3.769190567	-4.927973052	-1.179101055	-0.735881194	-3.342071846	-4.569280401	-2.838279989	-3.093246338	-0.480982282	-4.144042013	-2.343177044	-3.199508614
-1.210234065	-4.929885178	-3.630063931	-4.966012992	-1.704841536	-0.92049357	-3.582460211	-4.449463322	-3.015857424	-3.10664432	-1.252470211	-4.119089976	-2.491778011	-4.10689723
-0.887045998	-4.161875964	-3.571279184	-4.875121609	-0.409443026	-0.654582079	-3.809358266	-4.161574453	-2.961788265	-2.326344406	-0.576581871	-4.027742944	-2.116328747	-3.509136164
-0.869372417	-3.840055673	-3.597576037	-5.137720085	-0.080395382	-0.740348527	-3.786198005	-4.216712666	-3.104014869	-1.896594118	-0.693004652	-4.095090029	-2.176984967	-3.229297993
-1.565397962	-4.951903291	-4.468197433	-5.885374169	-2.446824087	-1.755665861	-3.526844638	-5.195926601	-3.774665515	-3.084065665	-1.447506513	-4.905460323	-2.568581222	-3.599881134
-1.77563631	-3.895011802	-3.79058897	-5.473949273	-1.406827009	-1.778850026	-4.127322611	-4.386665016	-2.810778396	-1.959165924	-0.824240943	-4.438086867	-2.430417795	-3.432824002
-1.629693301	-3.629376138	-3.571782067	-5.308162109	-0.729286292	-1.97965371	-3.971496576	-4.376386223	-2.66808823	0.139272715	-0.302730021	-4.380939442	-2.172142306	-3.643381516
-1.574592493	-4.615089143	-4.120142249	-5.366052812	-1.264409367	-1.88583626	-4.199224684	-5.257833681	-2.91508608	-1.970298419	-0.701568289	-4.901488152	-2.533823088	-4.243805193
-1.149348663	-4.3806269	-3.25923261	-4.742237926	-1.712675353	-1.529225863	-3.833143271	-4.590250618	-2.498809767	-1.093454729	-0.350114613	-4.152940527	-1.733490761	-3.883313779
-2.249702955	-4.357839297	-4.601093157	-6.084450175	-2.24694758	-2.409121851	-4.629386175	-6.022643841	-2.178763024	-1.144083324	-0.387784331	-4.659529165	-2.944278409	-4.102212525
-1.618402418	-4.102671586	-3.646523036	-5.328973676	-1.493705353	-2.161476741	-4.186327801	-4.818915064	-2.625351422	-1.325100841	-0.397499442	-4.276016072	-2.107990136	-4.061568502
-1.312362142	-4.344042685	-3.608627091	-4.933349439	-1.187012966	-1.546538272	-3.966668899	-4.532310189	-2.934717362	-1.092479145	-0.376082954	-4.289880636	-2.040895985	-3.749454824
-1.887245623	-4.516473028	-3.598832862	-4.996323953	-2.296777197	-2.437708225	-3.847823192	-4.444678079	-2.405701664	-1.377751497	-0.507492994	-4.335251073	-2.462567923	-4.112589885
-1.35183669	-4.592386866	-3.625148164	-5.006408838	-1.919123642	-1.637364612	-3.85079854	-4.639061458	-2.691127669	-2.916357906	-0.646562196	-4.337272489	-2.326390962	-4.035292088
-1.556792652	-3.791442261	-3.716519111	-5.485955843	-0.938418536	-1.717839924	-4.288476552	-4.213317702	-2.97175079	0.125508559	-0.536073989	-3.74866945	-2.551267023	-3.509294341
-1.840969348	-3.879047999	-4.075925887	-5.724405405	-1.675456596	-2.063831682	-4.751077074	-4.698799671	-3.161663209	-0.065541283	-0.840943193	-4.458743555	-3.033007796	-3.224769471
-0.458732365	-5.056577451	-3.396747488	-4.18052556	-2.185734953	-0.618491957	-3.431957749	-4.649230083	-2.851592381	-3.674700084	-0.361955993	-3.925191887	-1.272373748	-3.733839224
-1.647321301	-4.053450764	-3.746083493	-5.778251529	-0.950520937	-1.771771229	-4.189881018	-4.43115496	-2.629015967	-0.303657628	-0.222393232	-4.483203302	-2.22709213	-3.71619079
-1.483103224	-4.391904253	-3.907731029	-5.419619297	-1.374379049	-2.047646335	-4.26723531	-4.666137401	-2.634681775	-1.394285569	-0.233778108	-4.55642195	-2.302868878	-3.862571121
-0.932032816	-3.876404377	-3.532468323	-4.811552004	-0.734436513	-1.412199608	-3.852348665	-4.880386216	-2.908912576	-2.844402957	0.12660561	-4.251458383	-1.783748318	-3.42292184

-1.262693197	-3.757228854	-3.894687677	-5.310133578	-0.660803734	-1.400091956	-3.930992206	-4.611428432	-2.978453635	-1.178308399	-0.457293604	-4.442722964	-2.211611287	-3.106458448
-1.652173963	-3.724450188	-3.695420742	-5.3778304	-1.874405987	-2.415820253	-4.087218159	-4.649830303	-2.324187736	-0.465241788	-0.444057759	-4.354870175	-2.25461507	-3.263270698
-1.265546469	-3.802489088	-3.594155767	-4.937214727	-1.504718364	-1.846494877	-3.533155135	-4.732632493	-2.337047521	-2.357081216	-0.434461192	-4.369260908	-1.739020198	-3.549694191
-0.372122989	-4.281873168	-3.552389919	-4.260212496	-1.603344694	-0.508558224	-3.324370057	-4.399848257	-3.18463138	-3.144082047	-0.227758476	-4.290812052	-1.011134497	-3.096548898
-1.38551003	-4.733333015	-3.639918971	-4.872652776	-1.488625979	-1.10172357	-3.747275826	-4.615779843	-2.981507728	-1.508265791	-0.74311451	-4.202554757	-2.552369274	-3.712979736
-1.872729751	-3.434755899	-4.012115845	-4.856017947	-1.773112565	-2.05019251	-4.023415885	-4.516425476	-2.882089829	-0.508100932	-0.32229759	-4.478289769	-2.091895679	-2.718831998
-1.571450969	-3.716750364	-4.255234765	-4.661757227	-3.114365727	-2.152765541	-4.397282685	-4.856064121	-2.055811391	-1.413153604	-0.547071521	-5.310203008	-2.257354504	-3.288114418
-1.4735453	-4.697918255	-3.732337659	-5.02075382	-1.692356792	-1.374173935	-3.598186128	-4.668106473	-2.804791363	-2.386857162	-0.665428642	-4.181069629	-2.482129359	-4.051978642
-1.379356872	-3.582211632	-3.484085194	-5.146731407	-0.97792536	-1.774114788	-4.00413836	-4.396487488	-2.463570502	-0.032403174	-0.210219945	-4.650896973	-2.158012468	-3.665290415
-1.246947085	-3.566200389	-3.687248942	-5.299147902	-0.485428396	-1.741450203	-3.99684026	-4.343694425	-2.966723523	-0.030432891	-0.198962722	-4.401810558	-2.390836498	-3.230103394
-2.059169389	-3.737303058	-3.983818055	-6.429395399	-1.006455945	-1.977862673	-4.365474783	-4.816780138	-2.753650265	-0.637445845	-0.950860204	-3.945604857	-3.316822208	-3.283164541
-1.693084911	-3.768501761	-3.696501426	-5.482600173	-0.726411095	-1.927561231	-4.416547644	-4.452701291	-2.936380196	-0.934773966	-0.61274817	-4.017358195	-2.665160131	-3.574352028
-1.632976278	-5.010265084	-4.078467177	-5.460224248	-2.662533336	-2.093123886	-3.981563833	-4.72841747	-3.007679769	-2.137942999	-0.642370659	-4.620825911	-2.648674048	-4.515969851
-1.75607409	-4.109882754	-4.14006846	-5.620505386	-0.91421101	-2.565143389	-4.431663748	-4.596229778	-3.00636459	-0.45160637	-0.569374328	-4.571113071	-2.971851151	-3.894431103
-2.172859404	-3.988113479	-4.51230654	-6.342843155	-2.13291321	-2.610037008	-4.30878059	-5.465536241	-2.858613116	-1.330283696	-0.765094723	-5.382146056	-2.8648702	-3.596444004
-1.724909069	-3.940555173	-4.312326132	-5.653025989	-1.915981257	-2.240526094	-4.181441612	-4.711770367	-2.55508891	-1.734073147	-0.600494844	-4.802913727	-2.552275189	-3.638602283
-1.675248972	-3.953685907	-3.552512379	-4.870090006	-1.65237708	-1.590093169	-4.010355646	-4.580876522	-2.51615794	-1.227793325	-0.514130141	-4.226045123	-2.543172583	-3.58747848
-1.057985283	-4.707612672	-3.353282184	-4.548507702	-2.22619177	-1.332042889	-3.473509547	-4.390527647	-2.897071266	-2.603057581	-0.494183309	-4.013605169	-1.900302028	-4.071278366
-0.851638787	-4.015190947	-3.500031719	-4.779115543	-0.387091774	-1.377908737	-3.968814513	-4.562530318	-3.024707074	-1.220640771	-0.223042965	-4.523474924	-2.198954328	-3.109947401
-1.571998916	-3.726150736	-3.98136902	-5.796292454	-0.754371326	-1.75276862	-4.380220495	-4.196866761	-2.991637593	-0.773413409	-0.578075252	-4.310260484	-2.768451588	-3.123839744
-1.092262722	-4.128152867	-3.50157699	-4.996242226	-1.372545062	-1.120660334	-3.781293679	-4.039781859	-2.599426225	-1.548566402	-0.266427209	-4.029486148	-1.835417671	-3.406112352
-1.675257522	-4.355400946	-4.157692944	-5.469349359	-0.862393538	-1.517810007	-4.312723575	-4.367101775	-3.244180926	-2.344944645	-1.106442772	-4.408209862	-2.061283406	-3.388143698
-1.272508736	-4.107792168	-4.362058615	-5.763835506	-1.46989274	-1.209269377	-4.153180848	-5.218158797	-3.526152866	-1.977686278	-0.792416717	-4.544055535	-2.567746412	-3.151474829
-1.735974269	-4.103626662	-4.341121248	-7.596651096	-1.673924206	-1.906912582	-4.345947342	-5.3003344	-2.600959873	-1.817207369	-0.224108044	-4.829989517	-2.599149482	-3.285485645
-1.436477505	-4.20706299	-4.181037077	-5.838728905	-1.499336402	-1.186968266	-4.123798749	-5.180513968	-3.028801486	-2.482698829	-0.999778992	-4.520115818	-2.422961586	-3.077765705
-1.731191106	-4.087990996	-3.833966048	-5.200391566	-1.28681202	-1.664037905	-4.302467361	-4.870760974	-2.296584607	-2.073166312	-0.922523413	-4.437738903	-1.839170094	-3.359269247
-1.940611338	-4.526480632	-4.174107009	-5.405855475	-1.864584923	-1.902682013	-4.005018814	-4.219117037	-2.797142823	-2.697727045	-1.19913758	-4.385756852	-1.634329566	-3.207699709
-1.889627477	-4.672238986	-3.959576576	-5.354712317	-2.100107841	-1.615821226	-3.931408849	-3.998642843	-2.554597218	-3.177276611	-0.949763813	-4.365376006	-1.939677231	-3.503362149
-1.557810393	-4.31885144	-3.907431454	-5.148002725	-1.146083597	-1.4337203	-4.245182928	-4.218019651	-3.019555901	-2.414893267	-1.040252166	-4.432184603	-1.93616334	-3.337432182
-1.254954798	-4.041114193	-3.81915794	-5.062631816	-0.529490549	-1.202373405	-4.13087198	-4.076350072	-2.863043277	-1.921270496	-1.048971231	-4.47047853	-1.918107441	-3.456372137
-1.769067179	-4.112205545	-4.057118793	-5.219377263	-1.45590175	-1.712200899	-4.267227185	-4.087804558	-3.123725059	-2.150671244	-1.018104638	-4.760786423	-1.336697406	-2.971499883
-1.58974983	-4.208100753	-3.949568483	-5.053201131	-2.06839096	-1.816789019	-3.95335901	-4.555023604	-2.53582731	-2.459601962	-1.122265248	-4.739046461	-1.486701378	-3.257761091
-1.722062754	-4.363000281	-4.147018229	-5.222282051	-1.068166174	-1.398460554	-4.141738323	-4.28504322	-3.07163353	-2.184811733	-0.972845213	-4.258297451	-1.904282815	-3.171999048
-1.632572532	-4.215137776	-3.965879366	-5.391084732	-1.360448422	-1.654805638	-4.306264376	-4.12010248	-3.215851008	-2.306621716	-1.042081428	-4.449440749	-1.687138791	-3.032361271
-1.724274899	-4.428237205	-4.064498925	-5.577144063	-1.358347468	-1.959026638	-4.375035096	-4.715165074	-3.061233511	-0.958452518	-0.667574752	-4.479606807	-2.685396099	-3.941073266
-1.263570105	-3.809283276	-3.571513208	-4.9800584	-1.156655375	-1.879133146	-3.74178435	-4.596802272	-2.501726006	-0.480905052	-0.071302587	-4.267923896	-1.960517002	-3.560176513
-1.713945789	-3.431817523	-3.835568106	-5.25500638	-0.995615753	-1.996180391	-4.009552976	-4.189796474	-2.607994859	-0.164790978	-0.420157771	-4.182955185	-2.580831102	-3.117845597
-1.686807506	-3.829486702	-3.79766822	-5.532283013	-0.713120836	-1.949031617	-4.51329554	-4.590095762	-2.69281739	-1.068973498	-0.773275744	-4.269629624	-2.415632383	-3.602485965
-1.715516505	-4.279350169	-3.657267282	-5.250414576	-1.326815428	-2.333252395	-4.310012215	-4.742499645	-2.631935863	-1.082183448	-0.407698523	-4.436260502	-2.2859135	-3.795563084
-1.888080996	-4.391714419	-3.712910776	-5.138103754	-2.221994125	-1.750279127	-3.643322539	-4.610028184	-2.103620492	-1.164291434	-0.626460932	-4.494320334	-2.631237339	-3.974413683
-1.727812042	-3.698224651	-3.730695267	-5.229458021	-0.488865723	-1.897945209	-4.289499352	-4.642982263	-2.65629015	-0.407530264	-0.547314991	-4.152982417	-2.591753075	-3.672141258
-1.234437276	-3.542111882	-3.829191587	-5.469827336	-0.441598092	-1.70333548	-4.149366498	-4.384685527	-3.219084179	-0.445181094	-0.713089641	-4.327739365	-2.851193052	-3.286361106
-1.646663822	-3.86506366	-4.130627293	-5.925182383	-0.889714724	-2.146530368	-4.389473942	-4.471813378	-3.107324166	-0.372316906	-0.863248123	-4.441176772	-3.192838736	-3.537715261
-2.077350239	-4.582876444	-4.28886738	-5.893219456	-2.304571021	-2.232260245	-3.966462114	-5.359946536	-3.236114947	-1.378289956	-1.426338162	-4.822382145	-3.100609888	-4.228166976
-1.90207106	-4.489475846	-4.537758453	-5.907047923	-2.249049385	-2.327530552	-3.756622475	-4.981384513	-3.086866734	-1.719442391	-0.711052845	-5.042394317	-2.92583247	-3.878260282
-1.787721612	-3.801239174	-4.071994325	-5.721825494	-1.797024502	-1.862367989	-4.428816988	-4.666070786	-2.840291984	-0.908951495	-0.671877147	-4.705650053	-2.87000874	-3.296579777
-1.599296081	-4.17225406	-4.226397064	-5.38905799	-1.715341824	-2.561862029	-4.051886512	-5.204884701	-2.185280709	-1.187821223	-0.773902936	-4.433117839	-2.42212531	-4.143296085
-1.474480007	-3.678515368	-3.75405082	-5.536512236	-1.309234945	-2.162815091	-4.060886398	-4.495469775	-3.243942789	-0.380638118	-0.647835291	-4.518600021	-2.68234817	-3.421373664
-1.858251358	-3.589772075	-3.701958767	-5.395486456	-0.95714551	-2.122263276	-4.527325807	-4.262594772	-2.646459002	-0.655270736	-0.615516936	-4.1359804	-2.621087475	-3.345133276

-1.59049395	-4.138528869	-3.713652683	-5.096891521	-1.377589222	-1.424435444	-4.014695839	-3.788323283	-2.886436217	-2.354268569	-1.074856504	-4.193560688	-1.494433386	-3.084919536
-1.785936452	-4.083594451	-3.823124223	-4.899032696	-0.984337695	-1.537422884	-4.143381331	-4.223259366	-3.039398557	-2.229463635	-1.014869044	-4.120688466	-1.544371586	-3.055262995
-1.62596661	-4.021718485	-3.804711402	-4.813896799	-0.926547055	-1.328912298	-4.268999588	-3.992536573	-3.042489214	-1.755706391	-1.048612893	-4.297448978	-1.709796902	-3.020173377
-1.639959715	-4.146754875	-3.911911647	-5.134678859	-1.104710951	-1.41858719	-4.099341749	-4.029984795	-2.950057392	-2.30629191	-1.070729552	-4.173369597	-1.818094165	-3.131801525
-1.659713031	-4.405515275	-4.068497255	-5.37475647	-0.706321581	-1.342143985	-4.175311156	-4.377441508	-3.207037641	-2.215173845	-1.139211615	-4.44592036	-2.027488828	-3.281271629

hsa-miR-7847-3	hsa-miR-7851-3	hsa-miR-7975	hsa-miR-7977	hsa-miR-8052	hsa-miR-8059	hsa-miR-8060	hsa-miR-8063	hsa-miR-8064	hsa-miR-8071	hsa-miR-8073	hsa-miR-8089	hsa-miR-8485	hsa-miR-873-3p
-2.294907512	-4.177315377	-2.020643877	-1.422295202	-4.134931164	-2.465175751	-5.888468482	-1.902323424	-4.569697187	-2.847203213	-2.86781279	-3.598295145	-3.471184216	-6.912912804
-2.487278107	-4.774317307	-2.158370851	-1.36299332	-3.895469084	-1.412688717	-5.825928685	-2.126369712	-4.151791705	-2.647167183	-2.763018567	-3.294539593	-3.742682352	-5.954043881
-2.679057636	-4.37143857	-1.0087303	-0.461252841	-4.091026027	-2.299194137	-6.001562625	-2.294183665	-3.946078258	-2.247020751	-3.065184697	-3.287729249	-3.604183318	-5.585925653
-2.376105294	-4.586370628	-1.214929993	-0.554303583	-4.230494046	-2.163546366	-5.925051244	-2.302553943	-4.001063632	-2.226233328	-3.185783005	-3.49245126	-3.614168109	-5.135651056
-2.091505741	-4.14529451	-1.254272876	-0.727191891	-4.303553485	-2.323745447	-5.62363758	-1.595923378	-4.183819408	-2.730623347	-2.868014298	-3.338014058	-3.150046753	-6.330623656
-2.542273186	-4.799862492	-0.926268693	-0.373803304	-3.972340866	-1.559694267	-5.616717469	-1.611726469	-3.937102356	-2.559391377	-2.969158674	-3.419821432	-3.41681731	-5.705124607
-2.275241007	-4.245993581	0.066775608	0.157278942	-4.15616168	-1.969396395	-5.698154147	-1.936437749	-3.605099205	-2.391004211	-2.818210563	-3.194798308	-3.49603051	-5.575605453
-2.705492619	-4.889043817	-0.896033672	-0.14917124	-3.858823417	-1.330968653	-5.857524226	-2.104380381	-3.644345605	-2.224391751	-2.62257778	-3.482371905	-3.691912951	-5.382614747
-2.803113089	-4.710212748	-1.274193499	-0.562241606	-4.087823153	-1.726790239	-5.985418827	-2.683454402	-3.969435833	-2.070285954	-3.236076914	-3.752074278	-3.623207115	-5.910851939
-2.397297249	-5.038515003	-1.484864769	-1.036573502	-3.700410014	-1.214483192	-5.778502775	-1.693216807	-3.851842325	-2.530754645	-2.728172031	-3.579763749	-3.612497238	-5.367694967
-2.824205073	-4.316200578	-0.073059867	0.520710468	-3.949623681	-1.485953957	-5.792273402	-2.593821005	-3.817880112	-2.391827701	-2.988675947	-3.723640907	-4.044040796	-5.077020169
-2.524939468	-4.630572786	-2.116586632	-1.609770828	-4.060411106	-1.497844055	-6.789482554	-2.194634359	-3.984856997	-2.549847658	-3.212514964	-3.540365381	-4.377696755	-6.626819414
-2.660302238	-4.410241664	-1.436265383	-0.94534309	-4.147070102	-1.879246534	-6.49846593	-2.799651895	-3.748564296	-2.111414553	-3.342169394	-3.511473674	-4.372988874	-6.098522979
-2.743326804	-5.161756695	-1.211036286	-0.743192482	-4.141188659	-2.085764604	-6.272185717	-2.276150798	-3.844738295	-2.558593928	-3.235004409	-3.388529793	-4.009487022	-5.391786594
-2.839959404	-4.971687183	-1.16572026	-0.599281012	-3.912444731	-1.286734948	-5.898302703	-1.472448304	-4.11846597	-2.929313413	-2.838323158	-3.715244715	-4.143338748	-5.438655879
-3.040932804	-4.904966002	-1.543399136	-1.061591558	-4.036175633	-1.607109044	-5.564316163	-2.617765533	-3.75203326	-2.102057226	-3.104926647	-3.550585494	-4.164814875	-5.43195998
-2.877530871	-4.78760764	-1.048667566	-0.590016677	-3.881941935	-1.23662392	-5.833909215	-1.439760399	-4.033735081	-2.655867466	-3.14205943	-3.608677333	-4.104797585	-4.661330332
-2.881387542	-5.364254258	-1.603997099	-0.87025364	-3.848443122	-1.578172301	-6.254541765	-2.815563665	-4.17770106	-2.343306594	-2.869003096	-3.654172861	-4.395381989	-6.115187314
-2.601125155	-4.182641721	-0.934168899	-0.354756328	-4.003318271	-1.635697877	-5.875075115	-1.869316029	-4.308393564	-2.575013343	-3.038273603	-3.544088185	-4.059456934	-5.748955354
-2.643724955	-4.78376419	-0.671364602	-0.156609771	-3.876228562	-1.431799477	-5.837230175	-1.909588064	-3.968032763	-2.280766222	-3.04736918	-3.723920583	-4.025691758	-4.667702722
-2.785428134	-4.639475433	-1.116985361	-0.476362579	-3.846388764	-1.438927929	-5.940920477	-1.449319583	-3.997313694	-2.743601236	-2.882390185	-3.723647804	-4.066586534	-5.940801968
-3.041460486	-5.217343464	-0.721840466	-0.251966346	-3.985386786	-1.407842067	-6.087817445	-1.790585685	-3.959807092	-2.458870749	-3.117491851	-3.723557407	-4.140790656	-5.95750179
-2.63093263	-4.673882216	-0.88235053	-0.164402396	-4.063461748	-1.628722619	-6.081416596	-2.435544509	-3.864358785	-2.173530606	-2.964084136	-3.625283571	-3.9988356	-5.826732954
-1.704650363	-3.86291431	-1.182010831	-0.593026542	-4.078439034	-1.738816642	-5.782994657	-0.877392155	-3.820166171	-2.128525263	-2.816011632	-3.27513107	-3.713068063	-5.916176585
-3.030317827	-5.101466826	-1.658000488	-0.904591923	-4.059315679	-1.30002818	-6.006889951	-1.897928423	-3.923990149	-2.359394308	-3.077820082	-3.660585559	-4.111585559	-5.575388126
-2.202948538	-4.030658476	-1.526116983	-0.938396239	-4.147579257	-2.199226739	-5.975149126	-0.976606248	-3.859362959	-2.461805444	-2.980835408	-3.484442025	-3.58440235	-6.043359633
-2.793082934	-4.792645766	-0.938438572	-0.452404173	-3.805221407	-1.322160511	-6.275573152	-2.312917622	-3.754279405	-2.479921099	-3.252931169	-3.600860477	-4.179180049	-6.373289376
-2.445509578	-4.855298579	-0.764542018	-0.294303499	-4.20059411	-1.805284148	-6.379949743	-1.221696947	-3.948664051	-2.814162525	-3.085915686	-3.514254082	-4.093844091	-6.969396747
-3.085147693	-4.910003563	-2.457276077	-1.750198973	-3.928771799	-2.237679495	-6.647321966	-1.565267817	-4.589174777	-2.845054633	-3.461033245	-3.847088958	-4.005055928	-6.54393018
-2.595884216	-4.653620783	-1.413953952	-0.347599077	-3.977843862	-1.656878657	-6.098508102	-2.376510187	-3.72449647	-2.113590107	-3.002143456	-3.685358646	-3.889473081	-5.324627698
-2.451315646	-4.762133846	-1.140561205	-0.251515332	-4.063149889	-1.429839724	-5.715578898	-2.704611532	-3.427914267	-1.998863527	-2.92092938	-3.509285736	-4.04584848	-4.77226787
-1.895707946	-3.777859791	-1.864078031	-1.206879936	-4.395470599	-1.816157401	-6.863653543	-1.081106468	-5.005766936	-3.612653466	-4.391531476	-3.398384993	-4.75826369	-7.057418347
-2.945178727	-4.753034902	-2.379195715	-1.796253657	-3.669743815	-1.067449099	-5.822835779	-2.446517252	-4.009350855	-1.960048712	-3.365690536	-3.239881947	-4.362318898	-4.280970029
-2.953565263	-5.243246912	-1.712173926	-1.037854085	-3.790464286	-1.204129058	-5.64566887	-2.630239345	-3.814936007	-1.693804658	-3.046554776	-3.118524483	-4.297899627	-2.829265471
-3.467412928	-5.863315184	-3.000434338	-2.917683875	-4.22994721	-1.912680642	-6.529617274	-2.923832495	-4.250266363	-2.577930907	-3.339195337	-3.907223751	-4.573441081	-4.531647988
-2.913154864	-4.982509514	-2.831210851	-2.265290744	-4.08045285	-1.662991919	-5.713495316	-1.708517967	-4.411480323	-2.388644984	-3.029782896	-3.578615032	-3.922014784	-4.759915541
-3.352728124	-5.471453807	-3.632256075	-1.950577003	-3.98040037	-1.621259365	-6.835690919	-1.608937428	-5.404390105	-3.020810671	-3.338809679	-3.960460275	-5.735024048	-3.552663951
-3.235429935	-5.984275025	-2.827819177	-2.291104715	-4.016412426	-1.682609929	-6.179204254	-2.708121413	-4.270419468	-2.350272895	-3.04853645	-3.53742538	-4.083603293	-3.84764238
-3.221893461	-5.758222381	-3.719859875	-3.51034391	-4.275560965	-2.32358086	-6.494896187	-3.04583822	-4.219210617	-2.297261931	-3.075313584	-3.709669586	-4.012544525	-3.852363565
-2.925625709	-5.448794727	-3.258623294	-2.535973038	-3.855944658	-0.98416549	-5.829411303	-1.278983558	-4.820928206	-3.130741021	-3.014485617	-3.632043669	-4.385734927	-4.352165039
-3.080942733	-4.865793759	-3.007728903	-2.469415386	-4.231637286	-2.112190106	-6.341384089	-1.57628333	-4.50580776	-3.05833918	-3.082531297	-3.774040174	-3.912593538	-6.052330461
-2.983404311	-4.564275579	-1.988744614	-1.621750391	-3.900187363	-1.518185189	-5.919030957	-3.07857314	-4.232929382	-2.228483397	-3.256388705	-3.497059689	-4.474264754	-2.36798378
-2.442625838	-4.763390445	-2.742393012	-1.766837512	-3.825791209	-1.641580845	-5.415092464	-2.346570192	-4.738243779	-1.676165536	-3.492026938	-3.727460436	-4.710989699	-2.807189627
-1.697693114	-3.342638201	-5.067606781	-4.270376366	-4.315766412	-3.102598454	-5.761426027	-1.953313544	-4.829885775	-3.64126207	-2.61015953	-3.305627834	-3.470317778	-7.522185338
-3.247360596	-5.394067475	-3.525171068	-3.181109026	-4.147186746	-1.824963272	-6.096711351	-2.722551276	-4.414741453	-2.384947606	-3.239784483	-3.716301486	-4.280270727	-2.823042829
-3.210614424	-5.8022961	-3.307433174	-3.043609422	-4.090617902	-1.975258266	-6.318432997	-2.438696002	-4.43251201	-2.328341882	-3.296705812	-3.761349361	-4.3323567	-3.439622088
-2.776505653	-4.70895765	-4.666012142	-4.047457735	-4.53730257	-2.602545647	-6.306008352	-3.118147786	-3.84575954	-1.942795057	-2.775197227	-3.498386977	-3.927693121	-4.017574408

-2.941488149	-4.966749521	-3.361010639	-2.622265199	-4.073149173	-1.518064348	-6.181571815	-3.173740963	-4.203452197	-1.877928306	-3.043713828	-3.665502977	-4.301543359	-3.43449699
-2.627477726	-4.828629843	-2.230745623	-1.811136411	-3.727185493	-0.788161785	-5.129398499	-1.832818887	-4.384357651	-2.06045516	-2.818214234	-3.163295727	-4.608729795	-4.557927823
-2.571723994	-5.389242216	-2.481043072	-2.268735988	-3.709156418	-0.884724855	-5.990326542	-2.000809629	-3.891966681	-2.651432965	-2.67634275	-3.175435834	-4.019158156	-5.011420311
-1.637921122	-3.45295877	-4.063503491	-3.710621825	-4.49440589	-2.154586984	-5.928359511	-2.728430609	-4.430779749	-2.749475023	-2.773858952	-2.84707702	-3.776835074	-6.601514089
-2.525283392	-4.004473794	-3.192575941	-2.574595394	-4.427588467	-2.79973907	-6.316557158	-1.28297309	-4.938781194	-2.741670995	-3.297578462	-3.616753317	-3.867368552	-4.581922128
-2.150966114	-4.847255608	-2.105789043	-1.438342556	-3.664171138	0.227673168	-4.439417756	-2.155101587	-4.604832268	-2.699655381	-2.565897375	-3.01933974	-4.30127334	-3.331883112
-2.08853374	-4.859943413	-2.526131331	-2.317828552	-3.424614898	0.363152474	-4.838757493	-1.792044343	-4.693147948	-2.599980753	-2.543151523	-3.034527648	-4.603514295	-4.796050442
-2.459363822	-4.015332043	-4.264192279	-3.576355346	-4.368596222	-2.270038028	-5.860695002	-0.839014341	-4.619238658	-3.186789987	-3.395601385	-3.787261697	-3.818784155	-4.575787015
-2.770256885	-5.362902458	-3.293213853	-0.988802434	-3.761049894	-0.771730846	-5.928178413	-2.064883944	-4.105099246	-1.935995174	-3.119877496	-3.498796344	-4.422265916	-2.67899369
-2.81945664	-4.632034586	-2.573634041	-1.201570425	-3.970454592	-1.316363926	-5.903619232	-2.792695308	-4.183402563	-1.28987605	-3.082857312	-3.290282041	-4.282768255	-2.842379222
-3.225572714	-4.050476832	-1.527177379	0.451811809	-3.70994473	-1.364071107	-5.979377463	-2.54272738	-4.459468653	-2.058523486	-3.414934415	-3.590873132	-4.404542665	-2.314253411
-3.230152686	-4.437175873	-1.85298466	-0.274019262	-4.101024458	-1.888493159	-5.521298003	-2.654281376	-4.077990027	-1.915929361	-3.29367437	-3.64605626	-4.221760556	-2.434120187
-3.164839259	-4.835983503	-3.29037022	-2.428338448	-4.288881771	-2.813677451	-6.679429429	-1.823249235	-5.164051688	-3.335100378	-3.67695932	-3.826478745	-4.41684926	-5.762369975
-3.15712917	-4.843616108	-2.035256506	-0.993859049	-4.143961134	-1.786876407	-6.28842474	-2.51585549	-4.337689102	-2.148745265	-3.719219655	-3.62503244	-4.700679276	-2.923446167
-2.539512466	-5.991005066	-2.731452262	-0.745021568	-3.845801532	-0.434883337	-5.798057661	-2.182163148	-5.012938494	-2.53629508	-3.050963918	-3.25806455	-5.440050369	-3.446207591
-2.690707349	-5.782634014	-3.10166993	-2.559585296	-3.570869924	-0.650520576	-5.786363569	-1.936032571	-4.007823354	-2.478839286	-2.828468731	-3.549521964	-4.591625501	-4.547809527
-3.269662986	-5.825172033	-3.738337607	-3.316127502	-4.096520296	-1.565434421	-6.097137883	-2.213013434	-3.782626482	-2.569609848	-3.185322773	-3.705402955	-4.234623629	-4.047945237
-2.639024274	-4.111284945	-3.167164782	-2.524583767	-4.207666513	-2.697642581	-6.279042546	-1.351820297	-4.65095296	-2.933773563	-2.955707189	-3.361892576	-3.730205273	-6.204888182
-2.691303246	-4.673108427	-4.243720939	-3.537567463	-4.521276302	-2.563852476	-5.812540422	-2.860971626	-4.121072197	-1.64474792	-3.13824488	-3.182617561	-4.167928689	-3.849311451
-3.255505055	-5.189960322	-2.137806734	-0.79586333	-3.991007459	-1.425848987	-5.757361258	-2.618313668	-3.971266104	-1.687995304	-3.194596493	-3.808576969	-4.507022367	-3.151369862
-2.962028799	-3.754426649	-2.307973467	-1.633890698	-4.044462389	-1.654375765	-5.89422147	-2.213811532	-4.452182113	-2.461511253	-2.879999306	-3.566270215	-3.82734029	-4.652621741
-2.747721498	-3.665298049	0.254143349	0.967142849	-3.801838973	-1.418186495	-4.166756215	-2.325460388	-4.050160367	-2.158940681	-3.917933973	-3.34766913	-4.75562012	-5.239933066
-2.873366279	-4.531285194	-2.709339502	-2.925440658	-4.218186501	-1.963862374	-6.161238978	-3.177500551	-3.949923569	-1.915100559	-3.527660632	-3.760803964	-4.9512495	-4.158898841
-2.761852722	-6.527782176	-2.094231175	-1.000886056	-3.804582065	-0.580538764	-6.797937672	-2.2463409	-4.354869066	-2.034725373	-3.728303702	-3.587918128	-5.386398328	-3.989962632
-2.910437538	-5.380138424	-3.421281387	-2.455514242	-3.649657748	-1.275062105	-6.319993897	-2.736021577	-4.19654856	-2.280544429	-3.375387731	-3.390990414	-4.601578035	-5.321113682
-2.86580219	-4.882918663	0.191530935	0.315823956	-3.605043364	-0.646307304	-4.342432655	-2.41466486	-3.897854487	-2.327770452	-3.600787529	-3.42348118	-4.420968115	-5.853507637
-2.229445893	-4.652737374	-0.437492146	-0.031637959	-3.408088738	-0.465305731	-3.335561664	-1.290338579	-3.807150075	-2.642680132	-3.674419886	-3.150579041	-4.630056599	-6.243025071
-2.428631972	-4.521721544	-1.326132959	-0.632597942	-3.594229948	-1.009489955	-3.86496924	-0.991361633	-4.278331791	-2.969509374	-3.441356042	-3.373298824	-4.443167469	-6.858614896
-2.61466558	-4.4667551	0.032350359	0.465684966	-3.695756395	-1.158157222	-3.959250953	-2.157731729	-3.870739582	-2.272651964	-3.626729256	-3.228409984	-4.358190858	-5.612392404
-2.665864589	-3.974741491	0.271658383	0.80787994	-3.94722583	-1.064174689	-4.404563386	-2.611103893	-3.792595113	-2.023754514	-3.809436202	-3.587289265	-4.108575266	-5.245577395
-2.117524489	-4.559849035	0.100333063	0.61206499	-3.48182231	-0.577326495	-3.364130339	-2.29992285	-3.789024125	-1.89562261	-3.657801435	-2.913834744	-5.004957841	-5.463871224
-2.290914211	-4.808966907	-0.421594047	-0.041703969	-3.499314891	-0.484633757	-4.227199063	-1.652626582	-4.255121772	-2.718864897	-3.426938032	-3.232576521	-4.327096544	-6.068005466
-2.428602695	-3.355574541	1.612655612	1.821628304	-3.494633311	-1.024787375	-3.578065708	-2.089551083	-3.616180757	-1.866089942	-3.701423304	-3.015518787	-4.700729082	-5.036469277
-2.289326102	-4.168482042	0.207890112	0.66883226	-3.355708745	-0.829010582	-3.459551092	-2.045029111	-3.645612924	-2.042082796	-3.610453695	-2.740441834	-4.536998573	-5.462508726
-3.173472641	-5.435995934	-2.455971293	-2.214566144	-4.121790067	-1.821017853	-6.361033594	-2.814165826	-4.301823946	-2.425170973	-3.252301795	-3.793151328	-4.413590969	-3.313886988
-2.974969277	-5.732022379	-3.317130511	-2.855939767	-3.964134187	-1.411548418	-6.271340624	-2.373409134	-3.78462435	-2.479183975	-3.135704027	-3.379899719	-4.05725938	-3.37379427
-2.644039223	-4.969263705	-2.144090835	-0.828523764	-3.827597092	-0.715852925	-5.77851177	-2.559022627	-4.226067185	-2.093214727	-3.08055186	-3.356126753	-4.422218985	-2.325177157
-3.10712806	-5.212494581	-3.019405419	-1.625533523	-3.798822651	-1.167409149	-5.74397442	-2.872240657	-4.253853356	-2.546326528	-3.139147551	-3.535137271	-4.472166865	-3.427341351
-3.212467863	-5.916329341	-4.546288153	-3.729068234	-4.069136503	-1.50753574	-6.599063947	-2.598835843	-4.154102565	-2.586727893	-3.244220358	-3.622415623	-4.34330106	-3.449578659
-2.236392233	-5.732962026	-3.571696778	-3.440122337	-3.825389599	-1.102564265	-5.954933911	-0.210155356	-5.080528767	-3.204013322	-3.487896299	-3.487548658	-4.18807854	-5.141586095
-3.050319255	-5.042486408	-2.175004878	-0.426184161	-3.894064207	-1.421139032	-6.051843103	-2.782100316	-4.166067237	-2.006145601	-3.277953139	-3.677052845	-4.327836582	-2.68930646
-2.85961277	-4.125014457	-2.117470527	-0.401931902	-4.290808932	-1.570469605	-5.900375153	-2.858515656	-4.01396352	-1.431152655	-3.442431786	-3.575666016	-4.490300929	-2.138433512
-3.191907954	-3.622651622	-1.137162431	-0.249017006	-3.459955866	-1.876975941	-6.406352598	-2.732416315	-4.615074987	-1.617791406	-3.564315064	-3.412741844	-4.635711207	-3.082241843
-2.730976529	-4.371999975	-3.574335404	-2.570356116	-4.459040063	-1.830188222	-6.524592232	-1.169056847	-4.801199888	-2.791761129	-4.112122973	-3.60864662	-4.476586504	-4.023740532
-2.80846017	-5.006819841	-3.473394056	-1.623198725	-4.151144861	-1.922301398	-6.540204279	-1.580175089	-5.430224092	-2.918821885	-3.351701527	-3.534338028	-4.995320652	-4.306729064
-3.174423664	-5.486392526	-4.140362034	-2.136352526	-3.893715417	-1.513916384	-6.254053472	-2.707861787	-4.664595677	-1.807238676	-3.176356626	-3.575403131	-5.059846553	-3.179233707
-2.621402043	-6.747443247	-3.057143575	-2.254313985	-4.162766819	-0.458620675	-5.775853163	-1.254190513	-4.319923854	-2.484499507	-3.099665971	-3.473209425	-5.091470482	-4.2360063
-2.891069615	-5.217650247	-2.190892433	-1.440870314	-4.352064658	-1.954213935	-6.277790585	-2.800601941	-4.573983475	-1.689950698	-1.987322462	-3.316096928	-4.372012833	-3.227857769
-2.979241568	-4.988380694	-1.545182389	0.032309203	-3.978486563	-0.765082425	-5.310054617	-2.008098907	-4.246139834	-2.146822065	-3.267122325	-3.703275047	-4.396967294	-2.855431837

-2.145325907	-4.486069674	0.349315894	0.71516629	-3.33056051	-0.51315812	-3.196238373	-1.847799096	-3.631247288	-2.0654678	-3.421453131	-2.986093456	-4.402791744	-5.621335623
-2.233406684	-4.225953958	0.17002147	0.791624304	-3.189829998	-0.36081254	-3.10943883	-2.103186817	-3.530423827	-2.132479112	-3.185470746	-2.892268786	-4.461953487	-5.116934616
-2.373412855	-4.189802414	0.476439925	1.106285448	-3.429364504	-0.502050187	-3.341988254	-1.967742581	-3.572435865	-1.69565074	-3.513991475	-3.071688689	-4.364044216	-4.77951231
-2.413368826	-4.176848283	0.039024192	0.625379249	-3.47000933	-0.81838376	-3.767919062	-2.031217554	-3.754112599	-1.907285932	-3.50576869	-3.091522174	-4.492219094	-5.324508543
-2.707124955	-4.300412942	-0.048190877	0.481479396	-3.804498775	-1.411987493	-4.307053807	-2.35317319	-3.953628973	-2.069894485	-3.900664374	-3.376809138	-4.58164369	-5.66808318

hsa-miR-874-3p	hsa-miR-874-5p	hsa-miR-877-5p	hsa-miR-885-3p	hsa-miR-887-3p	hsa-miR-920	hsa-miR-92a-2-5	hsa-miR-92a-3p	hsa-miR-92b-3p	hsa-miR-92b-5p	hsa-miR-936	hsa-miR-937-5p	hsa-miR-939-3p	hsa-miR-939-5p
-3.469682198	-3.389934775	-4.835967194	-5.205682828	-2.109903752	-4.884997792	1.468838764	-1.876907295	-2.990470901	-0.482964328	-5.684950634	-0.289725361	-4.450113762	-2.110703988
-3.79946588	-3.718736389	-4.903177697	-4.415198991	-1.744758799	-4.312802769	1.468134261	-0.677408746	-2.175987194	-0.261271066	-5.132234316	-0.366831134	-4.615099031	-1.963694353
-3.653780382	-3.563462535	-4.893202485	-4.27092232	-1.981450835	-4.887904419	1.613755238	-1.533389639	-2.675802551	-0.395276097	-4.716038087	-0.134387698	-4.58863229	-0.98931002
-3.78092996	-3.726453926	-4.621726136	-4.105248587	-1.861651374	-4.829201746	1.533288068	-1.320884667	-2.948355563	-0.248778112	-4.322973295	-0.326275674	-4.679685043	-1.188626208
-3.565738452	-3.257924078	-4.644560099	-4.616329365	-2.036802279	-4.943168129	1.369573723	-1.665377582	-3.1857791	-0.094333894	-4.992857177	-0.56935052	-4.305963576	-1.458546297
-3.773439313	-3.523453536	-4.731569246	-4.25242982	-1.623100679	-4.745192065	1.435030002	-0.851261577	-2.755401003	0.055051098	-4.794974686	-0.416801747	-4.478792829	-1.778307001
-3.843608995	-3.433189929	-4.743375198	-4.413174308	-1.641132253	-4.514684755	1.751621682	-1.077283186	-2.783067952	-0.163765732	-4.548507335	-0.189313274	-4.389657157	-2.144800397
-3.754363648	-3.474266717	-5.053367167	-3.924929882	-1.526094535	-4.516308006	1.197211194	-1.24062476	-2.790518184	-0.370991413	-4.660728139	-0.602430316	-4.631023557	-1.822975744
-3.886104196	-3.675174649	-4.91415152	-4.258794556	-1.802183967	-4.80383758	1.638332697	-1.557439578	-3.034038627	-0.156824742	-4.666609618	-0.456209595	-4.592887222	-2.005800996
-3.60090734	-3.552852196	-4.807274664	-4.350202184	-1.47895962	-4.373325475	1.094696029	-0.982383968	-2.727097764	0.114416076	-4.83852918	-0.504638883	-4.535600239	-2.441197556
-3.894803995	-3.624159161	-5.134183035	-4.425109225	-1.437032841	-4.766164818	1.422192104	-0.95026764	-2.335476263	-0.293449585	-3.888590662	-0.624635613	-4.75896428	-2.195760547
-3.869345387	-4.113844342	-5.062029767	-4.374878572	-2.209891501	-4.530374588	0.889729518	-2.276620457	-4.566645409	-0.672633655	-5.148561244	-0.342755932	-5.146276257	-2.114515855
-4.078713683	-4.210411897	-5.217281822	-3.914392513	-2.135501763	-4.775272525	0.833071422	-2.339044783	-4.094529355	-0.597296318	-4.882797016	-0.486913937	-5.205372391	-2.134971259
-3.917393485	-3.835983979	-5.221496434	-4.770884768	-1.668618601	-4.998690716	1.140013269	-2.205855728	-3.964300513	-0.437747088	-4.681989136	-0.331447686	-4.732700589	-1.800926758
-3.874909836	-3.799675139	-5.099597351	-4.67012959	-1.687590553	-4.630967284	1.108419105	-0.938296491	-2.318146372	-0.068022605	-4.770713565	-0.54148129	-4.729390165	-2.487162039
-3.846183392	-3.930678442	-5.071413701	-4.068279458	-1.811455831	-4.813157134	1.147219303	-2.297208617	-3.727060148	-0.480372798	-4.194509443	-0.619979388	-4.819829991	-1.513921101
-3.801095762	-3.937877253	-5.088716987	-4.361921357	-1.901433579	-4.786201421	1.034221185	-0.770189278	-2.336698528	-0.457808053	-5.055863594	-0.411216305	-4.882795337	-1.891486803
-3.913386804	-4.110710485	-5.304993836	-4.56506092	-1.915808488	-4.324025209	1.208470889	-0.958254662	-1.916698221	-0.797823533	-4.759661505	-0.346295684	-4.801073162	-1.968996111
-3.938082039	-3.862097141	-4.780881583	-4.738596707	-2.290463207	-4.774610821	0.97363211	-1.531893792	-3.445754691	-0.560524527	-4.681502551	-0.459153291	-4.722474294	-2.17103725
-3.721340617	-3.926677177	-5.181155718	-4.356347445	-1.869833211	-4.551175065	1.213692088	-1.052625294	-2.274830925	-0.394524247	-4.557861225	-0.377137294	-4.700423083	-2.228015427
-3.722392977	-3.925537729	-5.063153094	-4.756279253	-1.562100119	-4.406306279	1.110108096	-0.778302033	-1.830248637	-0.28277279	-5.371319489	-0.390258111	-4.92225094	-2.172851646
-3.646292599	-3.866397151	-5.261981873	-4.51326393	-1.55861683	-4.684535974	1.022573458	-1.435890736	-2.612604831	-0.422441622	-5.249123536	-0.615626596	-4.743599196	-2.456102157
-3.673006279	-3.861500899	-5.033459795	-4.02411529	-1.941634667	-4.740508133	1.106490451	-1.445676419	-3.052054489	-0.17699861	-4.372969785	-0.634618597	-4.624406504	-1.743667521
-3.443524877	-3.57357603	-4.588145947	-4.441758047	-2.401735463	-4.252649515	1.122200296	-2.616700273	-4.562621173	-0.268335587	-5.038100944	-0.0565482	-4.492854419	-1.715939656
-3.625959689	-3.898083543	-5.216123081	-4.364465903	-1.674080476	-4.660091125	1.053725618	-1.81741304	-3.334565936	-0.375072932	-4.742950778	-0.866720937	-4.662811283	-1.737447251
-3.012192078	-3.550118095	-4.540725439	-4.526286873	-2.313337686	-4.895358836	1.255076531	-2.569408979	-4.047527208	-0.489136433	-5.193977417	-0.293050361	-4.378894351	-1.641308474
-3.910963903	-4.133366091	-5.410005976	-4.358519705	-1.857914507	-4.633670052	0.668752027	-1.734430849	-3.574621062	-0.452874348	-4.631024021	-0.5745509	-5.335247025	-2.4697526
-3.896827127	-3.884134793	-5.092526349	-4.879889383	-2.446451394	-4.912791762	0.881245073	-2.409080375	-4.036938334	-0.607697994	-5.134343203	-0.13310327	-4.707532632	-2.483504116
-4.18619059	-3.770357279	-5.497333858	-5.545884256	-2.104719515	-5.417215071	1.032437428	-1.109544905	-2.723401297	-0.811487351	-5.860348132	-0.220778674	-4.796545211	-2.212085765
-3.830890688	-3.810653296	-4.889645869	-4.32071997	-1.867238757	-4.711283182	1.307175232	-1.964462029	-3.872012584	-0.187861505	-4.419363879	-0.388816239	-4.685457741	-1.909716881
-3.885469492	-3.860338356	-4.848088062	-3.783129836	-1.830787683	-4.384100643	1.105169383	-1.246088406	-2.718204449	-0.098345946	-3.587677457	-0.483548272	-4.811497464	-2.421420231
-5.000086432	-4.398378754	-5.751912968	-4.944721339	-3.265542202	-5.069901995	-0.488348667	-2.731645998	-4.934549473	-1.698048049	-6.159841901	-1.00663545	-5.601650024	-2.991753239
-4.13266142	-4.041451396	-5.498823029	-4.238892142	-1.424799681	-4.265124991	0.115181314	-2.502773597	-4.348110519	-0.220067379	-5.532857875	-0.929484596	-4.862537933	-1.827760446
-3.765158532	-3.874329053	-5.559950499	-3.526415047	-1.286239616	-4.291716382	0.661353848	-2.52475254	-4.327836216	0.048891888	-4.011094683	-0.587190601	-4.728697885	-1.991447214
-4.052488306	-4.421204623	-5.990092626	-5.008459504	-1.988918433	-5.106731258	0.708500167	-3.690232279	-4.837150685	-0.190278399	-4.849536418	-0.294440359	-5.130286157	-1.710581681
-3.773566159	-3.7672849	-5.931329017	-4.527270383	-1.627134507	-4.563410173	1.274724627	-1.652979115	-2.536053671	0.365294648	-5.772091486	-0.654614363	-4.310568121	-1.200986743
-3.940953362	-4.542678792	-7.446739805	-4.614309055	-1.760484637	-4.44049892	0.220183783	-1.45838571	-2.286284013	0.368322636	-5.664068745	-0.978758995	-6.926040603	-2.019975694
-3.680747245	-3.847763841	-5.785650192	-4.195356639	-1.268072337	-4.030235794	0.96182392	-2.146504231	-2.992237501	0.182019878	-4.983425618	-0.881661212	-4.674220999	-1.401385674
-3.701935783	-3.919895006	-5.745031129	-4.471503478	-1.807401498	-4.863514709	1.535073516	-2.412702375	-3.339331545	0.163898305	-4.801192387	-0.620590994	-4.629495386	-0.996610001
-3.917360999	-3.833333161	-6.558118834	-4.870417717	-1.858451524	-3.844823441	0.493701875	-2.128236531	-3.392453687	0.181460621	-5.186615919	-0.908861485	-4.701898762	-1.890034304
-3.72676639	-3.872013341	-5.861555339	-5.513533161	-1.771591065	-4.413651757	1.165030925	-2.921230532	-3.623314087	0.429494147	-6.505777241	-0.627570876	-4.439947807	-2.049367849
-3.834994141	-4.064796654	-5.412143699	-3.366622208	-1.827136814	-4.007385513	0.984512084	-0.960938524	-1.990603373	0.146625214	-3.683407535	-0.834218624	-4.707755646	-1.401794745
-4.116013573	-4.464293052	-6.260207808	-3.601726542	-2.604815021	-4.515661688	0.395331251	-2.326310644	-3.386970497	-0.370991135	-5.248663205	-1.21494075	-5.105549257	-1.535063558
-3.283174489	-3.498327935	-5.275628049	-5.895548933	-1.817263981	-4.54858195	1.676339705	-4.606075681	-5.061574464	0.144934825	-5.644374075	0.007044201	-4.057588817	-1.556434131
-3.904448091	-4.28238825	-5.670629896	-3.776026386	-1.692947118	-4.801118884	1.469637504	-2.37624518	-3.180844454	0.415247307	-4.46470839	-0.630523151	-4.982159499	-1.245308141
-3.71373533	-4.206700719	-6.099462046	-4.06906906	-1.485560222	-4.486484367	1.141702105	-2.257918943	-2.816414784	0.080394504	-5.153728208	-0.747388427	-4.848055996	-1.310206382
-3.215233349	-3.860162824	-5.665498645	-4.126980857	-2.124824318	-5.094730411	1.531386806	-2.740060001	-3.53794115	0.271784846	-5.119937682	-0.484823303	-4.557542988	-0.455467154

-3.793855633	-4.068916585	-5.738508578	-4.093475094	-1.735127736	-4.486200853	1.098872569	-1.906153464	-3.071743417	0.115055892	-4.358450263	-0.701731267	-4.828768481	-1.75823029
-3.579155042	-4.055992916	-5.602341642	-3.488234517	-1.215966407	-3.26206263	0.309684446	-1.658729009	-2.48975263	0.637619991	-5.228036413	-1.138391964	-4.879638624	-1.460162644
-3.462315852	-3.674350655	-6.34330617	-4.834175498	-1.157649075	-3.639918448	1.006096767	-1.991905634	-2.872318302	0.437023309	-5.578593778	-0.711449635	-4.564375195	-1.19515375
-3.397218266	-3.364644435	-5.250554273	-5.273095506	-2.103881476	-3.795527342	1.47632987	-4.388421541	-5.006608072	-0.431508852	-4.944732739	-0.28665115	-4.168412594	-0.856174998
-3.669796664	-3.839949441	-5.294124086	-4.474009386	-2.729396504	-5.078411662	1.239263585	-4.229510368	-5.012123575	1.079491055	-5.447197122	-0.305483639	-4.512034842	-1.330200052
-3.877181701	-3.909510038	-5.258851155	-3.590983062	-2.079655634	-2.152194856	0.184927069	-1.586040833	-2.476108927	0.130574108	-4.505246237	-0.848533413	-4.967058768	-1.743374179
-3.594146393	-3.745825621	-4.924768723	-4.292919834	-1.671869754	-2.436124418	-0.119497157	-2.731471242	-4.23122461	0.296458953	-5.196175156	-0.876385802	-3.944740921	-1.52945456
-4.364152224	-3.893160359	-5.610264432	-4.437654491	-2.623844598	-5.126091487	0.758214043	-2.212709018	-3.081984168	-0.250982158	-5.430569451	-0.621330791	-4.679340844	-1.966947208
-3.809824839	-4.002413974	-6.388233031	-3.619765673	-1.328638296	-4.846023957	1.12374637	-2.490512572	-3.985604942	0.463381458	-4.431370857	-0.412561897	-4.548885237	-1.644034516
-3.675508854	-4.05091719	-5.613230588	-3.178259644	-2.000251393	-4.511484122	0.662944317	-2.73330363	-4.079551867	-0.015287359	-4.180333879	-0.783376662	-4.750924015	-1.000099297
-4.491032739	-4.759644503	-5.708381163	-3.465283768	-1.91264938	-4.264222096	-0.092184581	-1.368957283	-2.259846595	-0.169898588	-4.959804437	-1.006451578	-5.337736684	-1.957757356
-4.018890916	-4.271237762	-5.702785773	-3.463216045	-1.801865865	-4.587026073	1.146221091	-1.986235187	-2.915119852	0.130412417	-4.731011742	-0.883924363	-4.944466839	-1.203151064
-4.104862313	-4.143289651	-6.02348466	-4.753918675	-2.63174703	-4.604681198	0.577232085	-3.99878798	-5.039967313	-0.118787093	-6.872011477	-0.886232953	-4.863340561	-1.39042805
-4.083552357	-4.450633915	-6.12361865	-3.516789078	-1.889434091	-4.221284502	0.480700156	-2.883638783	-3.906413868	-0.040623106	-4.546573939	-1.049586361	-4.988386268	-1.469103212
-4.207284206	-5.351806289	-6.873722744	-3.852986995	-2.008430155	-3.048365169	-0.075172929	-2.752685181	-4.116523326	-0.085339268	-6.044721266	-1.068452102	-5.590780727	-1.82149915
-3.814262202	-4.067783781	-5.248136968	-4.317981473	-1.52319888	-3.228117326	-0.015509359	-1.113838256	-2.134682398	0.232857897	-5.204552835	-0.9865461	-4.913431004	-1.633644256
-3.875525013	-4.134127826	-6.473199314	-4.620150971	-1.651665954	-4.204539706	0.881025264	-0.417943589	-1.110751495	0.134551155	-5.524536534	-0.951599147	-5.005288793	-1.732966507
-3.936487857	-3.399932398	-5.87185479	-5.552735101	-2.636954972	-4.855836631	1.671787052	-4.230916565	-4.983716129	-0.436697957	-6.150147913	0.019082807	-4.225685498	-1.601049115
-3.640152322	-3.971838637	-5.786683626	-3.431092518	-2.169806748	-4.998464865	1.2332232	-4.257312827	-5.323823147	-0.013282882	-5.074701565	-0.298772269	-4.471300403	-0.1658063
-4.122855789	-4.339827233	-5.538867048	-3.070203104	-1.974337175	-4.185357899	0.938229364	-1.649620171	-2.161542571	-0.163214967	-4.498636905	-0.812445087	-5.102708476	-1.898341702
-3.786951682	-3.897143015	-4.951593355	-4.199006809	-1.768459802	-4.329688601	1.771207477	-2.61857452	-3.380408419	0.296627587	-5.559237435	-0.404076503	-4.436724446	-1.600965903
-4.582475326	-4.06979496	-4.429372725	-3.850773649	-1.916861131	-2.845750538	0.227294659	-1.365713943	-2.55894609	-1.123491607	-4.438581866	-0.873146042	-5.616475996	-2.779326675
-3.970097651	-4.627949975	-5.389144612	-4.564073394	-2.833815696	-5.062199245	0.154202532	-2.301634108	-3.778067496	-0.43565226	-2.19877214	-0.825071699	-5.454386535	-2.536008362
-4.299559779	-4.728446391	-6.00489055	-3.502343345	-1.574391124	-3.896425906	-0.418804247	-2.331260533	-3.408078386	-0.107267806	-4.862118556	-1.104794213	-6.302366834	-2.230255368
-4.17002033	-4.362846469	-5.411389212	-4.476033236	-1.837796265	-4.595001042	0.449497425	-1.423188336	-2.627701304	0.095793996	-4.650327335	-0.641261003	-5.311693474	-2.584902906
-4.392498754	-4.052677859	-4.971166233	-4.281795627	-1.378939469	-2.734178445	0.902538978	-1.793576573	-2.956594455	-0.894264376	-5.098394915	-0.955427239	-5.575812568	-2.414994911
-4.574858527	-3.590409629	-4.336042624	-3.98918059	-2.362928429	-2.095796831	-0.242974567	-1.901352701	-2.888148403	-1.146492672	-5.032978817	-0.753298302	-4.997223581	-2.299642109
-4.459090045	-3.812732928	-4.598055506	-4.508392478	-2.510774833	-2.393022287	0.258129069	-2.974710718	-3.653131937	-1.222469999	-5.430519265	-0.660307022	-5.089974182	-2.785238485
-4.448132828	-3.967465177	-4.752779319	-4.050772504	-1.84441126	-2.787439727	0.233294729	-1.69360965	-3.06357084	-0.935752517	-4.925471575	-0.672139358	-5.180609024	-2.284462713
-4.314419529	-3.861522598	-5.025649521	-4.128726923	-1.527866568	-3.105177296	0.975873905	-1.044356248	-2.454071376	-1.076610281	-4.144606636	-0.847607727	-5.330827663	-2.185581745
-4.596870501	-3.724742869	-4.474377047	-3.683867989	-2.106500478	-2.028168006	-0.236122629	-1.674976163	-3.133015772	-1.260845385	-5.259872764	-1.03653899	-5.314630603	-2.291351857
-4.055622106	-3.73805998	-4.787842619	-4.533714844	-1.996152205	-2.441816138	0.388107253	-0.917326691	-2.3647878	-0.930233157	-5.551733486	-0.583665719	-4.938965162	-2.39319979
-4.584456422	-3.737746336	-4.520412572	-3.683769068	-2.038763974	-2.070020508	0.087203795	-0.943539936	-2.027207185	-1.156790241	-4.371456336	-0.687216055	-5.349572585	-2.318799079
-4.418226455	-3.72546641	-4.209271871	-3.797921292	-1.896973855	-2.056395472	-0.137390073	-1.637624398	-2.988563585	-1.150532292	-4.770144811	-0.646161084	-5.314446338	-2.271212042
-3.953963578	-4.028668746	-6.032275053	-4.442943942	-1.848444007	-4.702575758	0.93818623	-2.490821263	-3.595374401	-0.185465937	-3.778528277	-0.405867364	-4.930665166	-1.392260844
-3.791060976	-3.862675587	-5.775109257	-4.192822874	-1.171117755	-4.415151416	1.472634511	-3.377568887	-4.549606899	0.433569266	-4.631818386	-0.348670006	-4.58420992	-1.090559503
-3.886767052	-4.080848222	-5.473123647	-3.463968483	-1.621215747	-3.274549904	0.487837992	-1.367027628	-2.511347636	0.110981353	-4.890798818	-0.993168662	-5.020234139	-1.455220688
-3.949508265	-4.159741374	-5.650305261	-3.950843989	-1.382589573	-3.823515721	0.872308741	-1.205729433	-2.401304963	0.49207002	-4.649972306	-0.986609372	-5.159518581	-1.393790364
-3.851891191	-4.079401736	-6.399516989	-4.696169794	-1.436221186	-4.51297081	0.78240169	-3.056521575	-4.0318337	0.296940096	-4.94930322	-1.091063607	-4.687563849	-2.322424386
-4.103031849	-3.819993661	-5.062630467	-3.890962853	-1.896354757	-4.307610702	-0.40014484	-1.962520055	-4.066241822	1.176103371	-6.763210882	-1.189241787	-4.689458027	-2.010019389
-3.743138862	-4.175016619	-5.330942296	-3.385632755	-1.370143954	-4.520790884	1.028665738	-1.454410559	-2.94937786	0.377323863	-4.350818797	-0.863957294	-4.809006827	-1.564754205
-4.021162466	-4.226300106	-5.78098044	-3.623932936	-2.115523219	-4.914727239	0.699304508	-2.37233577	-3.528338591	0.019753908	-4.933619537	-0.943142409	-4.9066366	-1.658788621
-4.172166282	-4.427967725	-5.940701395	-3.349412341	-2.873746982	-4.458093099	0.830346695	-2.685680402	-3.783328381	-0.243237648	-5.066454806	-0.67618311	-5.109823696	-0.846389001
-5.252458343	-4.438865906	-6.378501889	-4.158342584	-3.017245023	-5.183193086	-0.212638588	-3.045824316	-3.930470207	-0.780272602	-5.956540178	-1.013858488	-5.078430058	-1.793395309
-4.226962936	-4.200270531	-6.102224814	-4.590080843	-2.673025171	-4.183136987	0.531100219	-3.732338735	-4.72176864	-0.164591419	-6.387457103	-0.631524897	-5.088498589	-2.34392407
-3.902741459	-4.511308937	-6.18666989	-3.851132436	-2.023315075	-4.000491375	0.320682548	-1.646442405	-2.442569616	-0.014481905	-5.126097899	-1.245692053	-5.048440919	-1.774530957
-3.261645132	-4.275520115	-5.556046925	-5.134632189	-1.359238604	-3.831442251	0.297497477	-2.279599974	-3.841697447	0.44760563	-5.22279881	-0.925803818	-4.57029246	-1.976297744
-4.263061362	-4.233995808	-6.358098942	-3.638418065	-2.649775864	-4.44096472	1.091466069	-1.728211429	-3.0144251	-0.322991859	-4.91732097	-0.787342963	-4.794653337	-0.940082836
-4.16009299	-3.914883378	-5.485626817	-3.144717956	-1.296859064	-3.739390222	0.760852835	-1.056974768	-1.68588972	0.34353767	-4.683169245	-1.230835418	-4.846817125	-2.036402867

-4.216268272	-3.62345376	-4.189588842	-3.71117477	-1.80434874	-1.761276375	0.343688217	-1.309566079	-2.079500998	-1.061073333	-4.925671287	-0.649318766	-5.200563493	-2.04353601
-4.616164952	-3.660045892	-4.096508999	-3.642029939	-1.754884347	-1.780185996	-0.070541657	-1.363136692	-2.257470772	-0.969937286	-4.522704315	-0.737508913	-5.241913316	-2.085416158
-4.74715595	-3.676835777	-4.293578163	-3.399667796	-1.785051229	-2.013113012	0.245735052	-1.096996211	-1.934163508	-0.942694001	-4.249442874	-0.771048659	-4.738529297	-2.514542545
-4.677935544	-3.620036533	-4.595269851	-3.882684558	-1.680754257	-2.464061218	0.262260022	-1.196764439	-2.235233476	-1.175399657	-4.855277411	-0.746051663	-5.133554739	-2.533778885
-4.594566764	-4.149073979	-4.665106597	-3.866801147	-1.949795747	-3.04359598	0.458668423	-1.416992302	-2.609049436	-1.146734887	-4.604403153	-0.779301132	-5.435282889	-2.671126825

hsa-miR-940
-3.273063079
-2.37701516
-2.234913178
-2.502286266
-2.683373927
-2.067067156
-2.265438611
-1.68183235
-2.145326136
-1.897866083
-1.83842833
-2.067392563
-2.220176332
-2.16899927
-2.283196816
-1.630558764
-1.779119661
-1.493834931
-2.408021486
-1.896273139
-2.003622644
-1.877490534
-2.59630029
-2.780451192
-1.417996908
-2.850809247
-1.853628824
-2.581107405
-3.007137056
-2.527506576
-2.571106342
-3.874768367
-1.060401502
-0.91963789
-1.654588674
-1.4592927
-1.044551817
-0.796683777
-1.597272145
-1.36650458
-1.566570494
-0.833376833
-1.047709098
-2.907687875
-0.459141189
-0.860197009
-1.792851744

-1.250066843
-0.447858735
-0.432421169
-3.014943769
-3.171877215
-0.528888857
-0.37072735
-1.754362157
-0.296506463
-0.954413404
-1.510543275
-0.994795022
-2.618569633
-0.418611729
-0.72450206
-0.071531957
-0.387368777
-2.940630279
-1.968631504
-1.182967055
-2.548778928
-2.024669906
-3.068157992
-1.376093392
-2.308979383
-0.549631457
-2.572525942
-2.962417687
-1.877363643
-1.229389655
-1.791871268
-2.594405287
-1.68374772
-2.407506312
-1.997431772
-0.786588154
-0.476179887
-0.547873591
-0.470198274
-0.829508474
-0.732828584
-1.877830397
-1.468206145
-2.536163022
-2.72875562
-0.374112664
-0.306657654
-1.562887725
-0.902164846

-1.834543678
-1.86089175
-1.402317745
-1.911400404
-1.982620032

Supplementary Table2. Correlations of the Significant Down-regulated or Up-regulated miRNAs

	hsa-miR-223-3p	hsa-miR-22-3p	hsa-miR-221-3p	hsa-miR-4485-5p	hsa-miR-550a-5p	hsa-miR-423-3p	hsa-miR-23a-3p	hsa-miR-30d-5p	hsa-miR-873-3p
hsa-miR-223-3p		R=0.874,P=8.76x10^{-33}	R=0.902,P=8.76x10^{-38}	R=0.738,P=1.23x10^{-18}	R=0.630,P=1.75x10^{-12}	R=0.894,P=3.08x10^{-36}	R=0.881,P=5.41x10^{-34}	R=0.880,P=8.18x10^{-34}	R=-0.551,P=0.00000000231
hsa-miR-22-3p			R=0.965,P=3.56x10^{-59}	R=0.831,P=6.43x10^{-27}	R=0.639,P=6.37x10^{-13}	R=0.906,P=1.17x10^{-38}	R=0.883,P=2.31x10^{-33}	R=0.917,P=3.51x10^{-43}	R=-0394,P=0.0000459
hsa-miR-221-3p				R=0.843,P=1.96x10^{-28}	R=0.586,P=1.27x10^{-10}	R=0.910,P=1.96x10^{-39}	R=0.933,P=9.46x10^{-43}	R=0.954,P=1.74x10^{-53}	R=-0.345,P=0.000404
hsa-miR-4485-5p					R=0.698,P=4.72x10^{-16}	R=0.774,P=2.36x10^{-21}	R=0.717,P=3.19x10^{-17}	R=0.810,P=1.08x10^{-24}	R=-0.473,P=0.00000058
hsa-miR-550a-5p						R=0.621,P=4.25x10^{-12}	R=0.621,P=4.25x10^{-12}	R=0.575,P=3.22x10^{-10}	R=-0.766,P=1.13x10^{-20}
hsa-miR-423-3p							R=0.867,P=9.30x10^{-32}	R=0.923,P=8.84x10^{-43}	R=-0.465,P=0.000000991
hsa-miR-23a-3p								R=0.900,P=1.88x10^{-37}	R=-0.285,P=0.00389
hsa-miR-30d-5p									R=-0.322,P=0.00101
hsa-miR-873-3p									
